# Genome-wide evolutionary analysis of TKL_CTR1-DRK-2 gene family and functional characterization reveals that *TaCTR1* positively regulates flowering time in wheat

**DOI:** 10.1186/s12864-024-10383-2

**Published:** 2024-05-14

**Authors:** Peisen S. Su, Jingyu Li, Dongtian Zang, Zhiyu Wang, Yangyang Wu, Shatong Chi, Fanting Sun, Yufei Niu, Xuewen Hua, Jun Yan, Wenyang Ge

**Affiliations:** 1 College of Agronomy, Liaocheng University, Liaocheng, 252059 P.R. China; 2 Key Laboratory of Huang-Huai-Hai Smart Agricultural Technology of the Ministry of Agriculture and Rural Affairs, College of Information Science and Engineering, Shandong Agricultural University, Tai’an, Shandong 271018 P.R. China; 3 College of Agronomy, Anhui Agricultural University, Hefei, 230036 P.R. China

**Keywords:** Serine/threonine-protein kinases, *TaCTR1*, Flowering, Transcriptomics analysis

## Abstract

**Background:**

Flowering time has an important effect on regional adaptation and yields for crops. The tyrosine kinase-like (TKL) gene family is widely existed and participates in many biological processes in plants. Furthermore, only few TKLs have been characterized functions in controlling flowering time in wheat.

**Results:**

Here, we report that *TaCTR1*, a tyrosine kinase-like (TKL) gene, regulates flowering time in wheat. Based on identification and evolutionary analysis of TKL_CTR1-DRK-2 subfamily in 15 plants, we proposed an evolutionary model for *TaCTR1*, suggesting that occurrence of some exon fusion events during evolution. The overexpression of *TaCTR1* caused early flowering time in transgenic lines. Transcriptomics analysis enabled identification of mass differential expression genes including plant hormone (ET, ABA, IAA, BR) signaling, flavonoid biosynthesis, phenolamides and antioxidant, and flowering-related genes in *TaCTR1* overexpression transgenic lines compared with WT plants. qRT–PCR results showed that the expression levels of ethylene (ET) signal-related genes (*ETR*, *EIN*, *ERF*) and flowering-related genes (*FT*, *PPD1*, *CO, PRR, PHY*) were altered in *TaCTR1*-overexpressing wheat compared with WT plants. Metabonomics analysis showed that flavonoid contents were altered.

**Conclusions:**

Thus, the results show that *TaCTR1* plays a positive role in controlling flowering time by activating various signaling pathways and regulating flowering-related genes, and will provide new insights on the mechanisms of wheat flowering regulation.

**Supplementary Information:**

The online version contains supplementary material available at 10.1186/s12864-024-10383-2.

## Background

Vegetative and reproductive growth are the two major phases of the plant life cycle. The floral transition is one of the major transitions occurring between these two phases, and it determines plant environmental adaptation, survival, and grain yield. Plants have evolved complex regulatory networks in response to environmental signals, including vernalization, temperature, photoperiod, the phytohormone gibberellin (GA), and age, to tightly control flowering [1, 2]. In the last decade, many regulatory genes involved in plant flowering pathways, such as *CONSTANS* (*CO*), *FLOWERING LOCUS T* (*FT*), *PHOTOPERIOD1* (*PPD1*), *VRN*, and genes encoding protein kinases (PKs), have been reported [3–7]. Increasing evidence suggests that PKs are essential for regulating flowering [8–10].

Eukaryotic protein kinases belong to a large gene superfamily. They contain a protein kinase catalytic domain that phosphorylates the serine, threonine, or tyrosine residues of target proteins [11]. In plants, PKs are classified into seven large families, including AGC (PKA-PKG-PKC), CAMK (calcium- and calmodulin-regulated kinase), CMGC (cyclin-dependent kinases, mitogen-activated protein kinases, glycogen synthase kinases, and cyclin-dependent-like kinases), RLK (receptor-like kinase), STE (serine-threonine kinase), and TKL [12]. Some reports about the biological functions of PK in controlling flowering have recently been published. For example, a previous study demonstrated that the overexpression of the *Arabidopsis* CK1 family member MLK4 caused early flowering by repressing the expression of the negative flowering regulator FLC/MAF [13]. In soybean, CALCIUM-DEPENDENT PROTEIN KINASE38 (GmCDPK38), which contains different haplotypes, mediates flowering time and insect resistance [14]. The knockdown mutation of CPK32 has been shown to result in late flowering by altering *FLOWERING CONTROL LOCUS A* (*FCA*) alternative polyadenylation and *FLOWERING LOCUS C* (*FLC*) transcription [15].

TKLs belong to the serine/threonine kinase family in plants and are widespread among plants [16, 17] (Champion et al. 2004; Goldsmith et al. 2007). Yan et al. (2017) performed genome-wide analyses and classified PK genes into seven groups in *T. aestivum* (TKL 134), *Triticum urartu* (TKL 46), and *Aegilops tauschii* (TKL 48) [18]. The available literature has demonstrated that TKLs participate in various plant processes, including innate immune responses and embryonic development [19, 20]. The TKL constitutive triple response 1 (CTR1) has been widely studied. Its role in the negative regulation of ethylene (ET) signalling is well documented [21]. In *Arabidopsis*, the Raf-like protein kinase CTR1 (*AtCTR1*) negatively regulates ET responses by interacting with ethylene receptor 1 (ETR1) and the ethylene response sensor (ERS) [22]. The tomato CTR1-like protein kinase CTR2 (*LeCTR2)* is reportedly involved in defence responses and development [23]. In addition, previous studies demonstrated that an *osctr2* loss-of-function mutation and *OsCTR1* transgenic lines altered the flowering time and effective tiller number of rice [24].

Wheat is an important cereal crop worldwide. Flowering time affects regional wheat adaptation and yields. Here, we identified the function of one wheat TKL_CTR1-DRK-2 gene, *TaCTR1*, in controlling flowering time. Analysis of gene identification and evolution indicated that two exon fusion events occurred during the evolution of the TKL_CTR1-DRK-2 subfamilies I-IV. The overexpression of *TaCTR1* in wheat triggered early flowering through the activation of the ET signalling pathway and flowering-related genes. Our findings provide new insights into the regulatory roles of serine/threonine-protein kinases in determining wheat heading date.

## Results

### Identification and evolution of the TKL_CTR1-DRK-2 subfamily

**Fig. 1:**
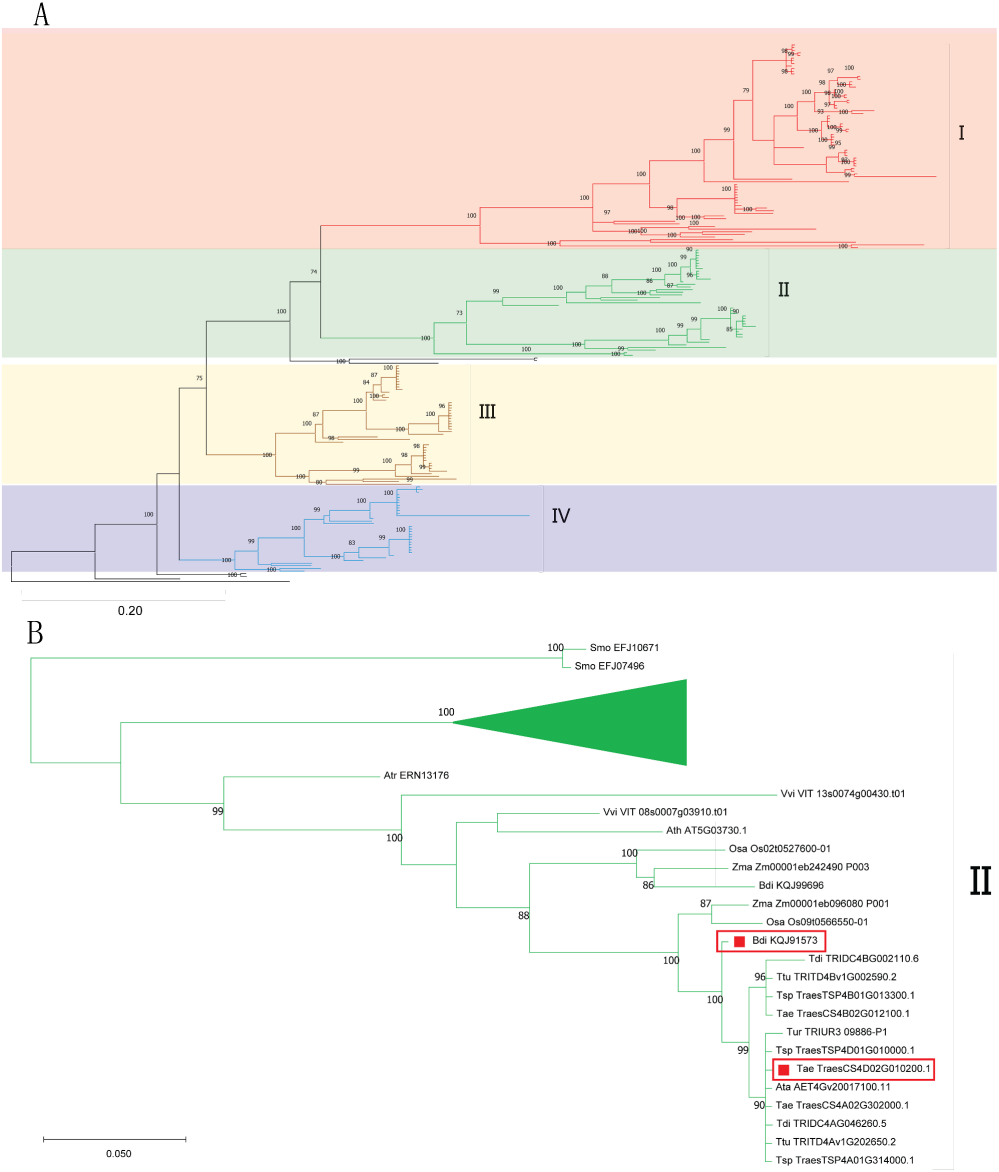
Classification and phylogenetic tree of the TKL_CTR1-DRK-2 in 15 plants

The TKL_CTR1-DRK-2 gene family belongs to the PK (protein kinase) gene superfamily studied in our previous article [18]. Here, we identified and classified the TKL_CTR1-DRK-2 gene family in 15 plants (Table S1). The other PK subfamilies were also re-identified and summarized in 15 plants (Table S2). Based on the four phylogenetic trees, we classified the TKL_CTR1-DRK-2 gene family into four subfamilies I-IV (Fig. 1 and Fig. S1). The results indicated that the classification was similar for the four phylogenetic trees (Table S3).

In order to obtain further insights into TKL_CTR1-DRK-2 evolution, we diagrammed the exon-intron structures within the kinase domain in the 15 investigated plants (Fig. S2). We found that TKL_CTR1-DRK-2 subfamilies I–IV contained a conserved exon-intron structure within the exon phases in the kinase domain (Fig. S3). For instance, the subfamily I contained conserved exon-intron structures within the “1002-0002-02” exon phases in the kinase domain. Subfamily II contained “1002-0000-2”, and subfamilies III and IV contained “1002-0002-2”. Interestingly, *P. patens* contained only one member (Pp3c12_3550V3.2) of TKL_CTR1-DRK-2, and its exon phases were “1002-0002-02,” hinting that subfamily I was the ancient subfamily. Indeed, Pp3c12_3550V3.2 was located in the root of subfamilies I and II clades among the bayesian and ML phylogenetic trees (Fig. S1).

We also studied the evolution of the TKL_CTR1-DRK-2 genes in *Triticum* species (Fig. S4). First, there was a “1-2-3-fold” trend in the distributions of members of the TKL_CTR1-DRK-2 subfamilies. In *Zea mays* and *Brachypodium distachyon*, the distributions of TKL_CTR1-DRK-2 subfamily members were the same (11 in total: 3 each in I, II, and III and two in IV). This distribution of subfamily III and IV members was similar in *Aegilops tauschii* (D genome, 12 in total: 5 in I, 2 in II, 3 in III and 2 in IV). Compared with those in *Ae. tauschii*, the number of members of subfamilies I-IV were twice as high in *Triticum dicoccoides* (AB subgenome, 25 in total: 11 in I, 4 in II, 6 in III and 4 in IV) and *Triticum turgidum* (AB subgenome, 26 in total: 12 in I, 4 in II, 6 in III and 4 in IV). Similarly, compared with those in *Ae. tauschii*, the number of members of subfamilies I-IV were three times as high in *Triticum aestivum* (ABD subgenome, 40 in total: 18 in I, 6 in II, 9 in III, 6 in IV, with 1 member excluded). The four phylogenetic trees also supported that the TKL_CTR1-DRK-2 genes of *Triticum* species underwent a genome triploidization event during their evolution (Fig. 1 and Fig. S1). For example, a subclade of subfamily I contained a “1-2-3-fold” trend with a bootstrap value of 100 in *Ae. tauschii* (D genome, Ata AET6Gv20792500.7), *Triticum urartu* (A genome, Tur TRIUR3 17,777-P1), *T. dicoccoides* (AB subgenome, Tdi TRIDC6BG056450.9 and Tdi TRIDC6AG048310.2), *T. turgidum* (AB subgenome, Ttu TRITD6Bv1G188650.3 and Ttu TRITD6Av1G195350.3), *T. spelta* (ABD subgenome, Tsp TraesTSP6D01G320200.1, Tsp TraesTSP6B01G378500.1 and Tsp TraesTSP6A01G304500.1) and *T. aestivum* (ABD subgenome, Tae TraesCS6D02G301700.1, Tae TraesCS6B02G352700.1 and Tae TraesCS6A02G321900.1).

**Fig. 2:**
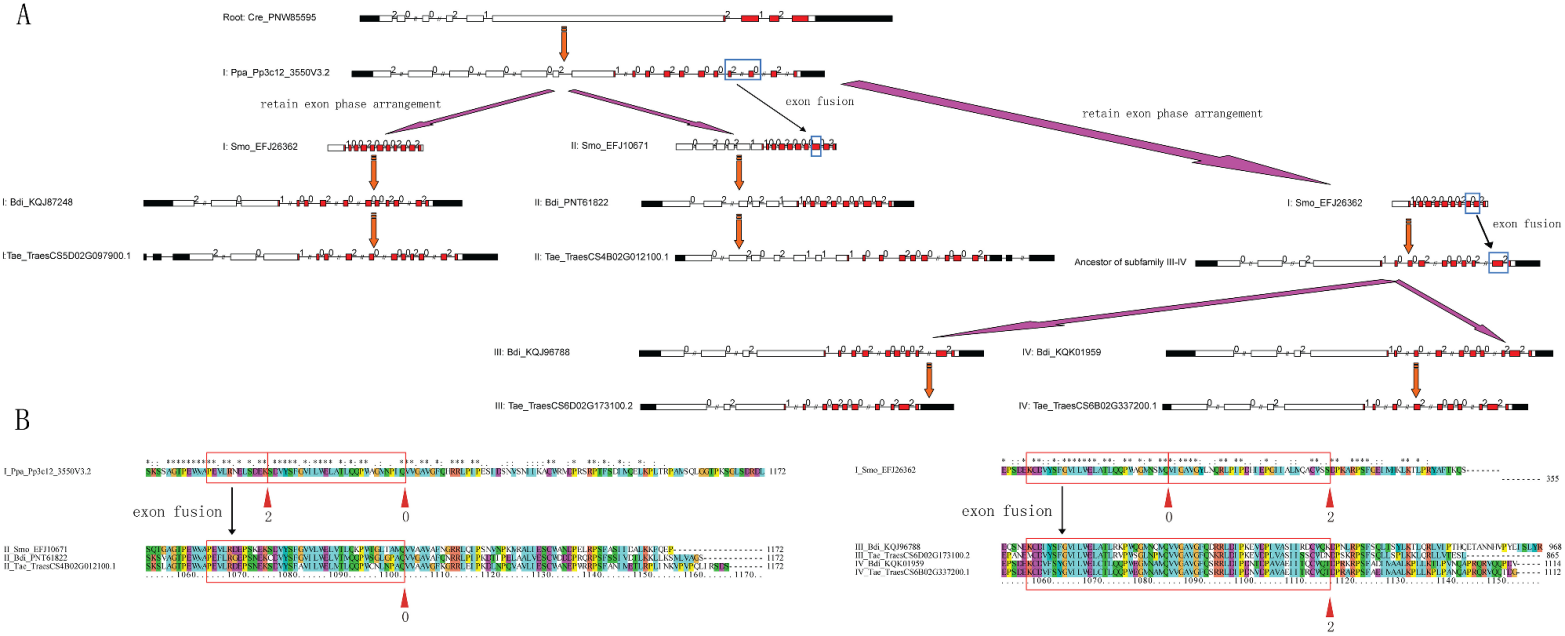
Exon − intron evolutionary model of TKL_CTR1-DRK-2 subfamilies I-IV. (**A**) Two exon fusion events of subfamily II and III-IV TKL_CTR1-DRK-2 in evolution. (**B**) Alightment of exon fusion blocks with amino acid sequences. Filled boxes: red represents the PK_Tyr_Ser-Thr domain; black boxes: untranslated regions (UTRs); white boxes: other exon regions; lines: introns; numbers 0, 1, and 2: exon phases. The lengths of the boxes and lines are scaled based on the lengths of the genes. The long introns are shorted by “//”. Exon fusion events are circled by blue boxes. Exon phases are circled by red boxes and arrows

How did these four types of exon phase arrangements of TKL_CTR1-DRK-2 evolve to form the subfamilies I–IV? Based on the analysis above, we inferred an exon-intron evolutionary model with two exon fusion events of TKL_CTR1-DRK-2 subfamilies I–IV (Fig. 2). This model suggests that two rounds of exon fusion events occurred during the emergence of Pteridophyta and Angiosperms (Fig. 2A). The first exon fusion event—two exons fused into one exon—emerged in subfamilies I and II during the *Pteridophyta* emergence. The exon-intron structure of *P. patens* TKL_CTR1-DRK-2 subfamily I (Pp3c12_3550V3.2) is “1002-0002-02”, while the exon-intron structure of the subfamily II of TKL_CTR1-DRK-2 genes from *S. moellendorffii* to Angiosperms evolved into “1002-0000-2”. Two exons behind within the exon phase “20” in the subfamily I fused into one exon within the exon phase “0” and formed the subfamily II of TKL_CTR1-DRK-2. The evidence of alignment within the breakpoints of exon fusion events between some subfamilies I and II genes supported our proposed evolutionary model (Fig. 2B). Similarly, the second exon fusion event—two exons fused into one exon—emerged in the subfamilies I, III, and IV during the Angiosperm emergence, or even earlier. The last two exons of the subfamily I within the exon phase “02” fused into one exon within the exon phase “2” and formed the ancestor of the TKL_CTR1-DRK-2 subfamilies III and IV. The exon-intron structure “1002-0002-2” of subfamilies III and IV remained in Angiosperms. We also aligned nucleotide sequences within the breakpoints of exon fusion events (Fig. S5).

**Fig. 3:**
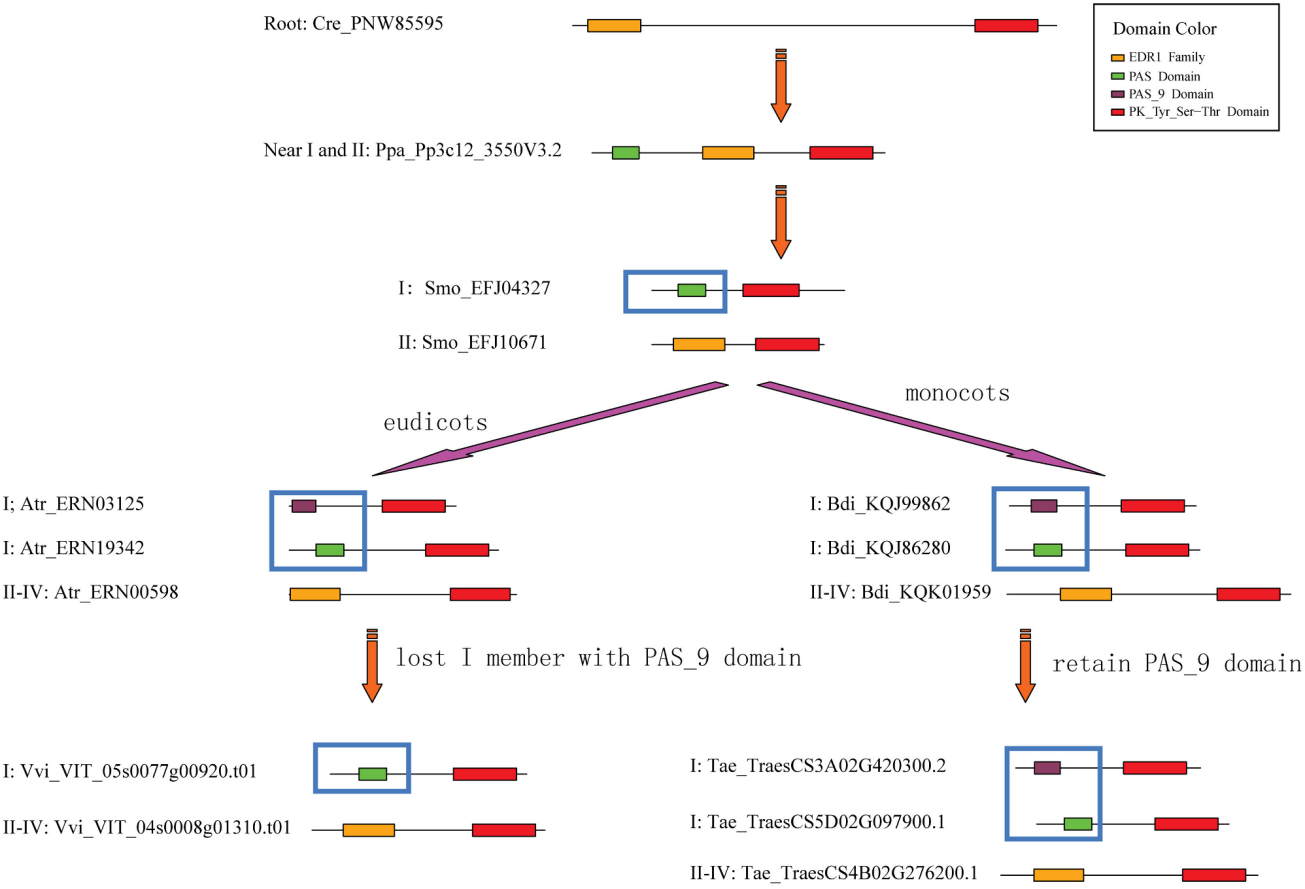
Domain evolutionary model of TKL_CTR1-DRK-2 subfamilies I-IV. Filled boxes: red represents PK_Tyr_Ser-Thr domain; green represents PAS domain; purple represents PAS_9 domain; orange represents EDR1 domain. PAS and PAS_9 domains were circled by blue box in subfamily I. The lengths of the boxes and lines are scaled based on the length of proteins (X axis)

We diagrammed the domain structures of the 15 investigated plants (Fig. S6). The evolution of domains in representative species is summarized in Fig. 3. *C. reinhardtii* TKL_CTR1-DRK-2 (PNW85595) contains two domains (EDR1 and PK_Tyr_Ser-Thr), and *P. patens* TKL_CTR1-DRK-2 (Pp3c12_3550V3.2) contains three domains (PAS, EDR1, and PK_Tyr_Ser-Thr). Subfamilies I and II had already appeared in *S. moellendorffii*, and they contain different domain compositions (I: PAS and PK_Tyr_Ser-Thr; II: EDR1 and PK_Tyr_Ser-Thr). Later, the PAS domain of one subfamily I member became the PAS_9 domain in the ancestor of eudicots and monocots. Surprisingly, the subfamily I member within the PAS_9 domain was lost in the eudicots *V. vinifera* and *A. thaliana* but was retained in the primitive eudicot *(A) trichopoda* and some monocots, such as *(B) distachyon* and *T. aestivum*. Subfamilies III and IV emerged in eudicots and monocots, and their domain compositions were the same as those of subfamily II (EDR1 and PK_Tyr_Ser-Thr).

### Analysis of functional divergence

Gene families are generated from gene duplication events on genome-wide or local chromosomal scales. Type I functional divergence indicates that the amino acid pattern is highly conserved in one duplicate cluster but highly variable in the other cluster [25, 26]. To determine the shift-selective constraints in the TKL_CTR1-DRK-2 gene family, the coefficients of functional divergence (θ) were calculated based on pairwise sequence comparisons (Fig. S7 and Table S4). The results showed that the θ values of four of the six combinations (I/II, I/III, II/III and III/IV) were slightly greater than zero, with *p* < 0.05 (LRT, df = 1, 3.841 at 5% for χ2), suggesting that a site-specific rate shift after gene duplication is not a common phenomenon in the evolution of the TKL_CTR1-DRK-2 gene family. Moreover, we noted that the p values of two combinations (I/IV and II/IV) differed. We also calculated the posterior probabilities (Qk) of each site from these 91 TKL_CTR1-DRK-2 combinations (Figure Sx3 and Table Sx2). The results showed that only a few sites had Qk values greater than 0.67, which supported that type I functional divergence is not a common phenomenon after gene duplications during the evolution of the TKL_CTR1-DRK-2 gene family.

### Chromosome location and duplication events in wheat

**Fig. 4:**
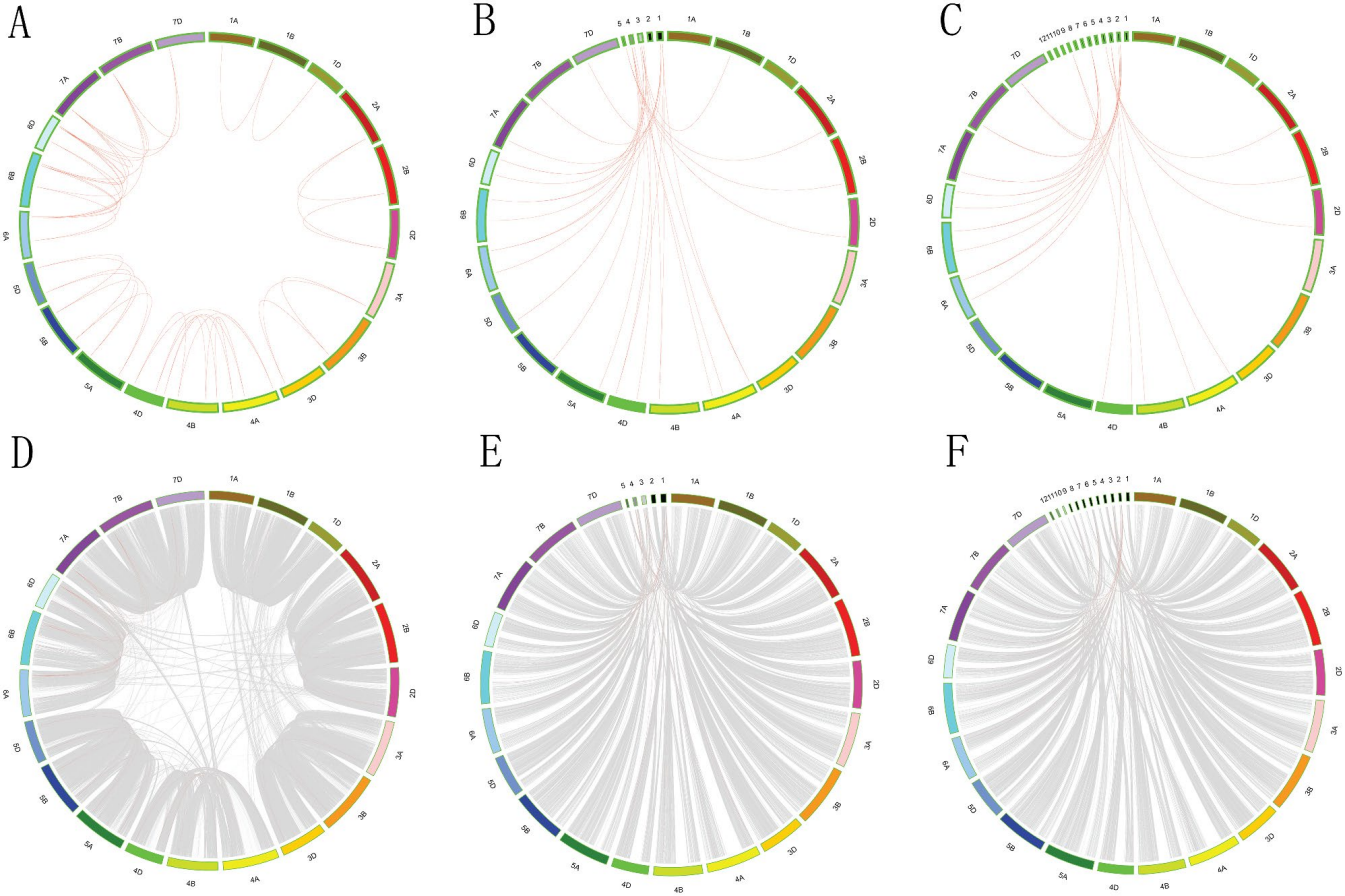
Synteny analysis of TKL_CTR1-DRK-2 genes. This graph displays syntenic maps of *T. aestivum* associated with two Graminaceae (*B. distachyon* and *O. sativa*). Red curves represent syntenic gene pairs between the TKL_CTR1-DRK-2 genes, and grey curves represent other genes. (**A**) Synteny of TKL_CTR1-DRK-2 genes in *T. aestivum*. (**B**) Synteny of TKL_CTR1-DRK-2 genes and other genes in *T. aestivum*. (**C**) Synteny of TKL_CTR1-DRK-2 genes in *T. aestivum* and *B. distachyon*. (**D**) Synteny of TKL_CTR1-DRK-2 genes and other genes in *T. aestivum* and *B. distachyon*. (**E**) Synteny of TKL_CTR1-DRK-2 genes in *T. aestivum* and *O. sativa*. (**F**) Synteny of TKL_CTR1-DRK-2 genes and other genes in *T. aestivum* and *O. sativa*

We mapped the locations of 40 TKL_CTR1-DRK-2 genes on 21 *T. aestivum* chromosomes (Fig. S9). About half of *T. aestivum* chromosomes contained only one TKL_CTR1-DRK-2. However, the other *T. aestivum* chromosomes, including 3 A (2 TKL_CTR1-DRK-2 genes), 3D (2), 4A (3), 4B (3), 4D (3), 5 A (2), 5B (2), 5D (3), 6 A (3), 6B (3), and 6D (3) contained more than one TKL_CTR1-DRK-2 (Table S6). In order to study the relationship between TKL_CTR1-DRK-2 expansion and *T. aestivum* polyploidization, we identified 42 collinearity events between A, B, and D sub-genomes of *T. aestivum* by using MCscanX (Fig. 4A and B). *Ks* values of TKL_CTR1-DRK-2 and all genes of *T. aestivum* collinearity events were also calculated using MCscanX (Table S6). A peak of *Ks* values (0.00–0.2) was observed in the collinearity events of *T. aestivum* TKL_CTR1-DRK-2 and all genes (Fig. S10A).

The allohexaploid bread wheat (*T. aestivum*) genome contains three subgenomes (A, B, and D), which are the products of polyploidization. The A and B genomes diverged 6.5 Mya, and they generated the D genome 1–2 million years later [27]. To determine the phylogenetic mechanism of the *T. aestivum* TKL_CTR1-DRK-2 genes, comparative syntenic maps of *T. aestivum* associated with two Graminaceae (*B. distachyon* and *O. sativa*) were also generated, and 28 collinearity events related to the TKL_CTR1-DRK-2 genes were found between the two Graminaceae (Fig. 4C and D). The *Ks* values of TKL_CTR1-DRK-2 and all genes between *T. aestivum* and *B. distachyon* were also calculated (Table S7). A peak in the *Ks* value (0.25–0.4) was observed in the collinearity events of TKL_CTR1-DRK-2 and all genes between *T. aestivum* and *B. distachyon* (Fig. S7B). Similar studies were also performed between *T. aestivum* and *O. sativa* (Fig. 4E and F, Table S7, and Fig. S10C).

### Expression pattern of wheat TKLs during different developmental stages

We assessed the expression patterns of wheat TKL genes from four public RNA-seq datasets from the NCBI SRA during development (Fig. S11). Based on the results of FastQC analysis, we performed a quality control assessment and excluded one RUN file from the following analysis (Table S8). (1) Grain development of wheat: The grain transcriptome at four developmental stages (5, 10, 15, and 20 days postanthesis) was investigated via RNA-Seq. The transcriptomes of additional wheat tissues, including roots, stems, leaves, flag leaves, grains, and spikes (from wheat plants at the booting or heading stage), were also studied via RNA-Seq. Three TKLs (III_TraesCS4D02G274400, III_TraesCS4B02G276200, and III_TraesCS4A02G029800) exhibited sustained high expression levels at the four developmental stages in the grain and other wheat tissues. (2) Early wheat (*T. aestivum*) spike development: Six stages of wheat early spike development (before elongation, elongation, single ridge, double ridge, glume primordium differentiation, and floret differentiation) were analysed via RNA-seq coupled with bioinformatics. We extracted TKL genes from genome-wide mRNA transcriptome profiling and noted that some wheat TKLs, such as II_TraesCS2A02G426200 and I_TraesCS5A02G085900, exhibited sustained high expression levels in these six stages of early spike development in wheat. On the other hand, some TKLs, such as IV_TraesCS6B02G337200 and III_TraesCS1D02G136400, exhibited sustained low expression levels in these six stages. (3) Four growth stages of wheat (Chinese Spring) flag leaves: Some TKLs, such as II_TraesCS4A02G302000, II_TraesCS4D02G010200, and III_TraesCS4A02G029800, exhibited high expression levels in the four growth stages. In contrast, other TKLs, such as I_TraesCS3B02G455600, I_TraesCS6D02G301700, and I_TraesCS4A02G171500, exhibited low expression levels in the four growth stages. (4) Wheat early and late heading date: Most TKLs, such as IV_TraesCS7D02G191400, IV_TraesCS7A02G190300, and IV_TraesCS7B02G095300, exhibited high expression levels in all eight investigated samples (Fig. S11). In addition, we measured the expression pattern of *TaCTR1* in different tissue including root, stem, leaf and spike. The results showed that the expression levels of *TaCTR1* in leaf were significantly higher than other tissues (Fig. S12). The expression levels of *TaCTR1* were performed among different wheat cultivars at the seedling, heading, and flowering stages. The results showed that expression level of *TaCTR1* was significantly higher in JM32, SuZhou 8332 and Xiaobaimai at the flowering stage (Fig. S13).

### *TaCTR1* overexpression causes early flowering in wheat

**Fig. 5:**
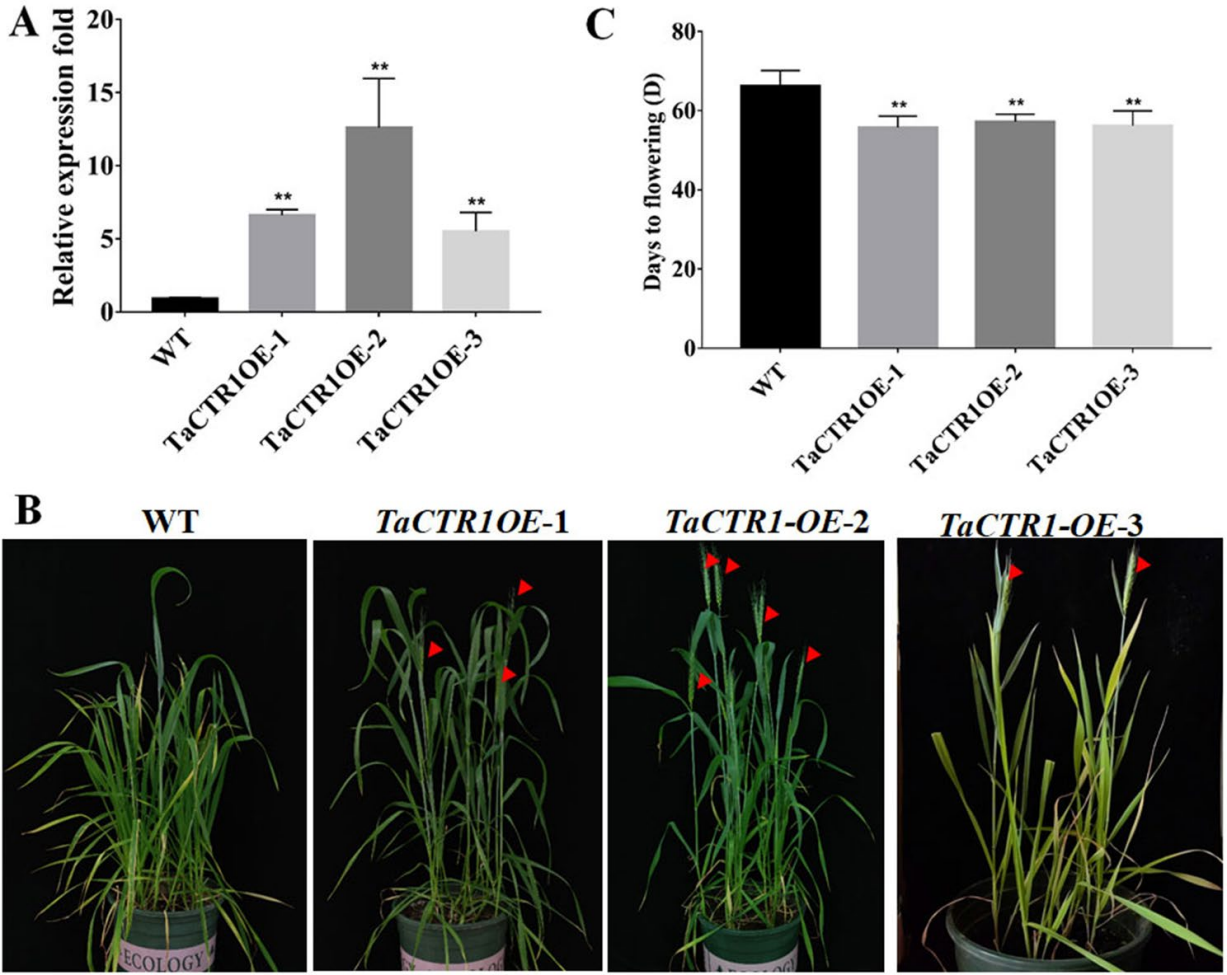
*TaCTR1* overexpression causes early flowering in wheat. (**A**) The expression levels of *TaCTR1* in transgenic lines and WT plants. (**B**) Comparison of flowering phenotypes among *TaCTR1* overexpression transgenic and wild-type (WT) plants grown in the greenhouse. (**C**) Flowering time of *TaCTR1* overexpression transgenic and WT plants. Data are means ± SD of 15 plants. (*P < 0.05; **P < 0.01; t-test)

Some *TaCTR* information, including protein sequence, coding sequence (CDS), chromosome information, GFF information, exon − intron and kinase domain diagrams, and BLAST results, are shown in Fig. S14. Analysis of public RNA-Seq data indicated that the expression of *TaCTR1* was upregulated at the heading stage, followed by a “maintained high expression level” (Fig. S11). To further elucidate the biological function of *TaCTR1*, we generated *TaCTR1*-overexpressing transgenic lines in the common wheat “JWI” background and successfully genotyped three independent homozygous overexpression transgenic lines. The transcript levels of *TaCTR1* in *TaCTR1*-overexpressing transgenic lines were analysed by qRT‒PCR. The results indicated that the expression levels of *TaCTR1* were greater in the transgenic lines than in the WT plants (Fig. 5A). Subsequently, the transgenic and WT plants were planted in a greenhouse and subjected to a detailed phenotypic analysis. The *TaCTR1* transgenic lines flowered seven days earlier than did the WT plants (Fig. 5B and C). In summary, the overexpression of *TaCTR1* promoted flowering time in wheat.

### *TaCTR1* overexpression alters gene profiles in wheat

In order to better understand the regulatory mechanisms by which *TaCTR1* affects wheat flowering, we performed a transcriptome analysis comparing *TaCTR1* overexpression transgenic lines and JWI wild-type plants. Our transcriptomic data indicated that 13,312 DEGs (8610 upregulated and 4702 downregulated) were identified between transgenic lines and JWI plants (Fig. S15A, Table S9). GO classification analyses revealed that molecular functions (lipase activity, glucosidase activity, chlorophyll binding, and microtubule binding), biological processes (cell tip growth, developmental cell growth, and post-embryonic plant organ morphogenesis), and cell components (photosystem II, secretory vesicle, and photosystem I) were highly enriched terms (Fig. S15B, Table S10). KEGG enrichment analyses indicated that DEGs were mainly enriched in the biosynthesis of secondary metabolites, plant hormone signal transduction, MAPK signaling pathways, carbon metabolism, and starch and sucrose metabolism (Fig. S15C, Table S10).

### *TaCTR1* overexpression stimulates the flavonoid biosynthesis pathway

**Fig. 6:**
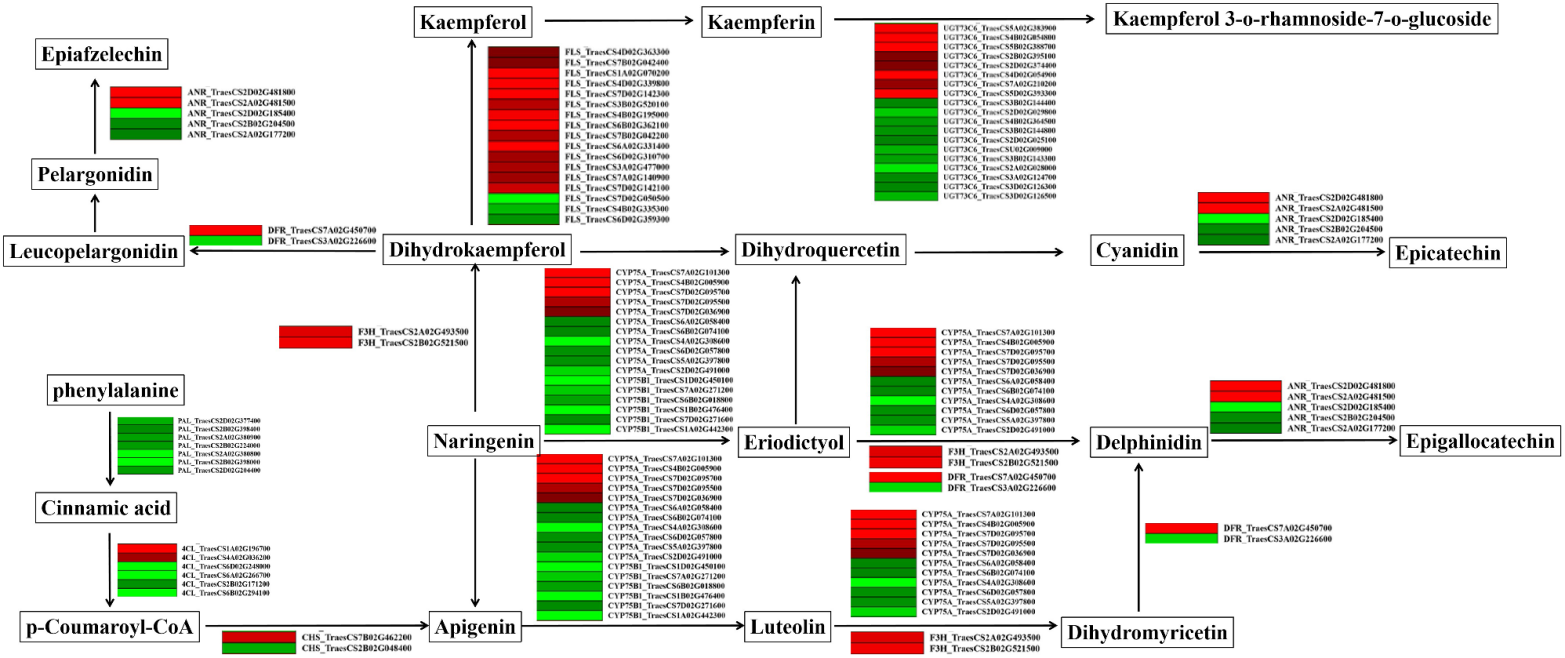
*TaCTR1* overexpression alters flavonoid biosynthesis pathway. The network of flavonoid biosynthesis pathway in *TaCTR1* overexpression transgenic lines, compared with WT plants. *PAL*, phenylalanine ammonia-lyase; *4CL*, 4-coumarate–CoA ligase; *CHS*, chalcone synthase; *DFR*, flavanone 4-reductase; *ANR*, anthocyanidin reductase; *FLS*, flavonol synthase; *UGT73C6*, flavonol-3-O-L-rhamnoside − 7-O-glucosyltransferase; *F3H*, naringenin 3-dioxygenase; *CYP75A*, flavonoid 3’,5’-hydroxylase; *CYP75B1*, flavonoid 3’-monooxygenase, *FG2*, flavonol-3-O-glucoside L-rhamnosyltransferase. The red and green indicate high and low expression level, respectively

To further study how *TaCTR1* affects plant flowering, we generated a network to illustrate the relationships between flavonoid biosynthesis and *TaCTR1* overexpression. We found that the transcript levels of 77 genes in the flavonoid biosynthesis pathway were altered, among which naringenin 3-dioxygenase (*F3H*) was upregulated compared to the control, while phenylalanine ammonia-lyase (*PAL*) and flavonoid 3’-monooxygenase (*CYP75B1*) were downregulated (Fig. 6, Table S11). In addition, the expression profiles of flavanone 4-reductase (*DFR*), flavonoid 3’,5’-hydroxylase (*CYP75A*), 4-coumarate–CoA ligase (*4CL*), anthocyanidin reductase (*ANR*), and flavonol-3-O-L-rhamnoside-7-O-glucosyltransferase (*UGT73C6*) were combined; detailed information is displayed in Table S11.

To further explore which type of flavonoid metabolites were altered due to *TaCTR1* overexpression, we measured the flavonoid content in *TaCTR1*-overexpressing lines and WT plants. The results showed that 8 flavonoid-related metabolites, including swertisin, apigenin-7-O-(2”-glucosyl)arabinoside, and apigenin-7-O-rutinoside-4’-O-rhamnoside, were upregulated. Eighty-nine flavonoid-related metabolites, including kaempferol-3,7-O-diglucoside, apigenin-7,4’-dimethyl ether, 6-hydroxy-2’-methoxyflavone, and abrusin, decreased in abundance (Table S12). *TaCTR1* overexpression promoted early flowering in the transgenic lines by stimulating flavonoid biosynthesis.

### Plant hormone signaling pathways induced by *TaCTR1* overexpression

**Fig. 7:**
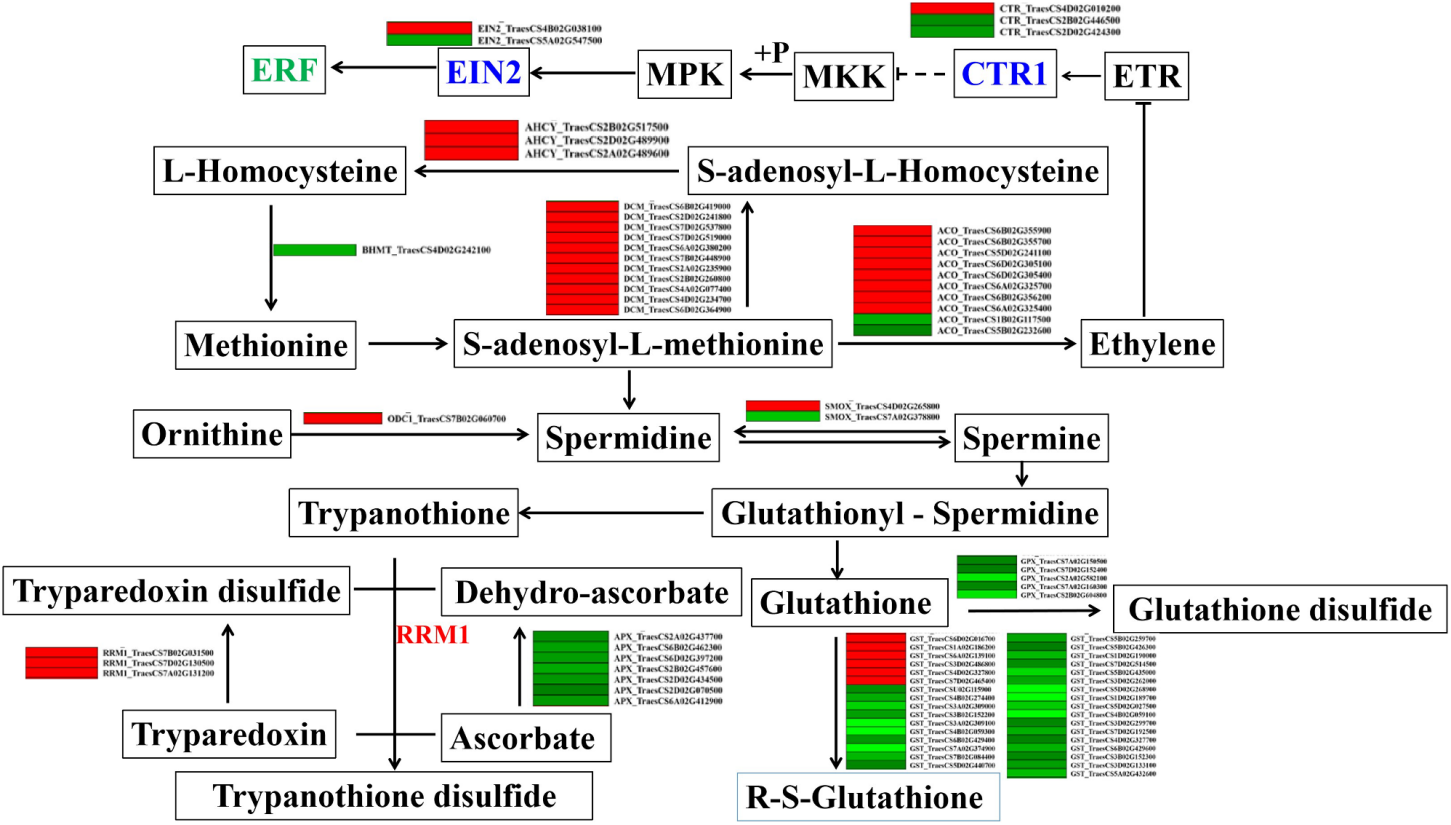
*TaCTR1* overexpression alters ethylene-related genes expression. The network of ET biosynthesis and signalling, phenolamides and antioxidant pathways in *TaCTR1* overexpression transgenic lines, compared with WT plants. *BHMT*, homocysteine S-methyltransferase; *DCM*, DNA (cytosine-5)-methyltransferase 1; *AHCY*, adenosylhomocysteinase; *ACO*, aminocyclopropanecarboxylate oxidase; *MKK*, mitogen-activated protein kinase kinase; *MPK*, mitogen-activated protein kinase; *EIN2*, ethylene-insensitive protein 2; *GPX*, glutathione peroxidase; *GST*, glutathione S-transferase; *APX*, ascorbate peroxidase; *RRM1*, ribonucleoside-diphosphate reductase subunit M1; *ODC1*, ornithine decarboxylase; *SMOX*, spermine oxidase; *ERF*, ethylene-responsive transcription factor. The red and green indicate high and low expression level, respectively

**Fig. 8:**
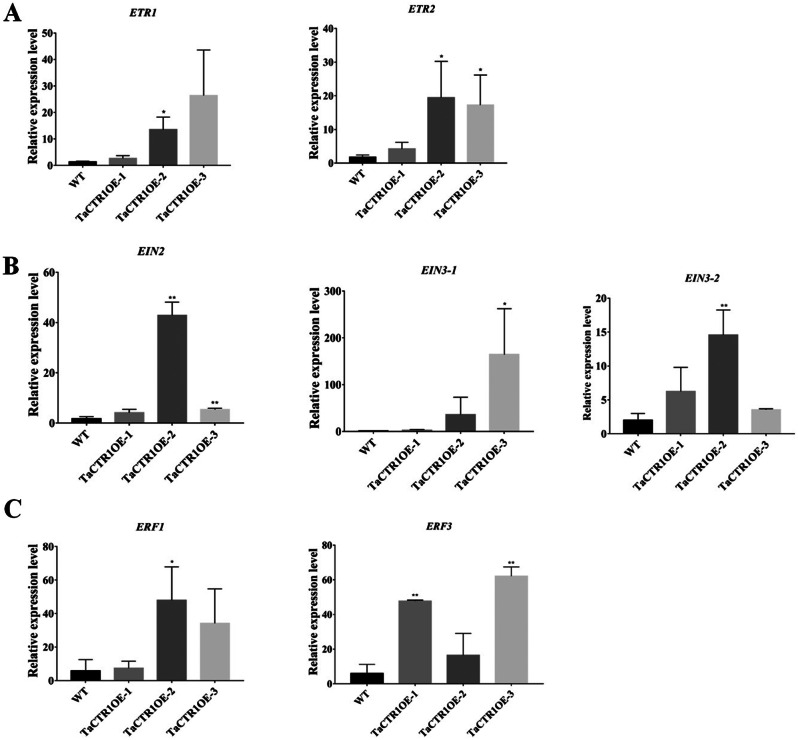
*TaCTR1* overexpression alters the relative expression of ET signal-related genes. (**A**) The expression profile of *ETR*. (**B**) The expression profile of *EIN*. (**C**) The expression profile of *ERF*. All experiments included three replicates. The wheat gene *18SrRNA* was used as an endogenous control. The gene expression profile were calculated using the 2^–ΔΔCT^ method

Our transcriptome data also indicated that phytohormone pathways were activated in *TaCTR1* overexpression transgenic lines. Therefore, we generated a network to explore the relationships between phytohormone pathways and *TaCTR1* overexpression. A large number of genes were enriched in plant hormone pathways: 32 ET-related genes were altered, among which adenosylhomocysteinase (*AHCY*) and DNA (cytosine-5)-methyltransferase 1 (*DCM*) were upregulated compared to the control, while ethylene-responsive transcription factors (*ERF*) were downregulated (Fig. 7, Table S11). We also performed qPCR to analyze the expression levels of known ET-related genes in transgenic lines and WT plants. The results indicated that the transcript levels of several ET-related genes, such as ethylene receptor (*ETR*), ethylene insensitive (*EIN*), and ethylene-responsive transcription factor (ERF), were significantly increased in *TaCTR1* overexpression lines compared with WT plants (Fig. 8).

**Fig. 9:**
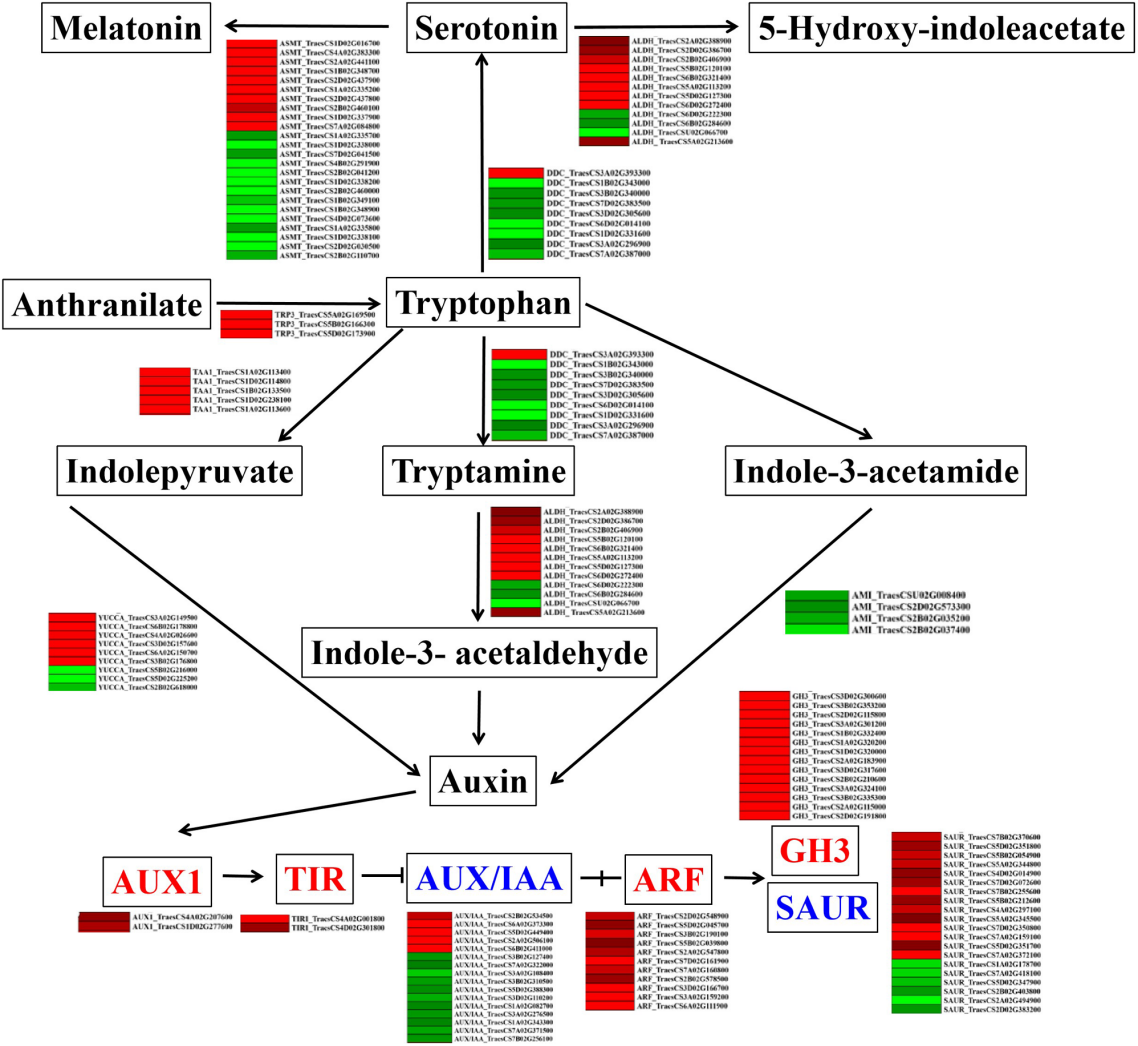
*TaCTR1* overexpression alters auxin signaling pathways. The network of auxin signaling pathways in *TaCTR1* overexpression transgenic lines, compared with WT plants. *TPR3*, anthranilate synthase; *DDC*, aromatic-L-amino-acid; *TAA1*, L-tryptophan—pyruvate aminotransferase;*ALDH*, aldehyde dehydrogenase (NAD+); *YUCCA*, indole-3-pyruvate monooxygenase; *AMI*, amidase; AUX/IAA, auxin-responsive protein. The red and green indicate high and low expression level, respectively

In addition, 64 ABA-related genes were found, among which abscisic-aldehyde oxidase (*AAO3*) and protein phosphatase 2 C (*PP2C*) were upregulated compared to the control, while 15-cis-phytoene synthase (*ctrB*), prolycopene isomerase (*crtH*), zeaxanthin epoxidase (*ABA1*), and 9-cis-epoxycarotenoid dioxygenase (*NCED*) were downregulated (Fig. S16, Table S11). 131 auxin-related genes—including anthranilate synthase (*TRP3*), L-tryptophan-pyruvate aminotransferase (*TAA1*), indole-3-pyruvate monooxygenase (*YUCCA*), aldehyde dehydrogenase (*ALDH*), and auxin-responsive GH3 gene family (*GH3*)—33 SA-related genes (*NPR1*, *TGA*, *PR-1*), 51 JA-related genes (hydroperoxide dehydratase (*AOS*), lipoxygenase (*LOX*), *COI1*, and *MYC2*), and 110 BR-related genes (steroid 5-alpha-reductase (*DET2*), brassinosteroid 6-oxygenase (*CYP85A1*), BR-signaling kinase (*BSK*), protein brassinosteroid insensitive 1 (*BRI1*)) were altered upon *TaCTR1* overexpression. The detailed information is displayed in Fig. 9, Table S11.

### *TaCTR1* overexpression alters phenolamides-related gene expressions

In plants, S-adenosyl-L-methionine (SAM) is a precursor of ET and polyamine biosynthesis [28]. Therefore, we analyzed polyamine biosynthesis pathways and found 51 altered genes, among which ornithine decarboxylase (*ODC1*), spermine oxidase (*SMOX*), and ribonucleoside-diphosphate reductase subunit M1 (*RRM1*) were upregulated compared to the control, while glutathione peroxidase (*GPX*) and ascorbate peroxidase (*APX*) were downregulated (Fig. 7, Table S11). The transcriptome analysis indicated that *TaCTR1* overexpression alters the expression of these genes involved in ET and polyamine biosynthesis pathways.

### *TaCTR1* overexpression alters flowering-related gene expression

**Fig. 10:**
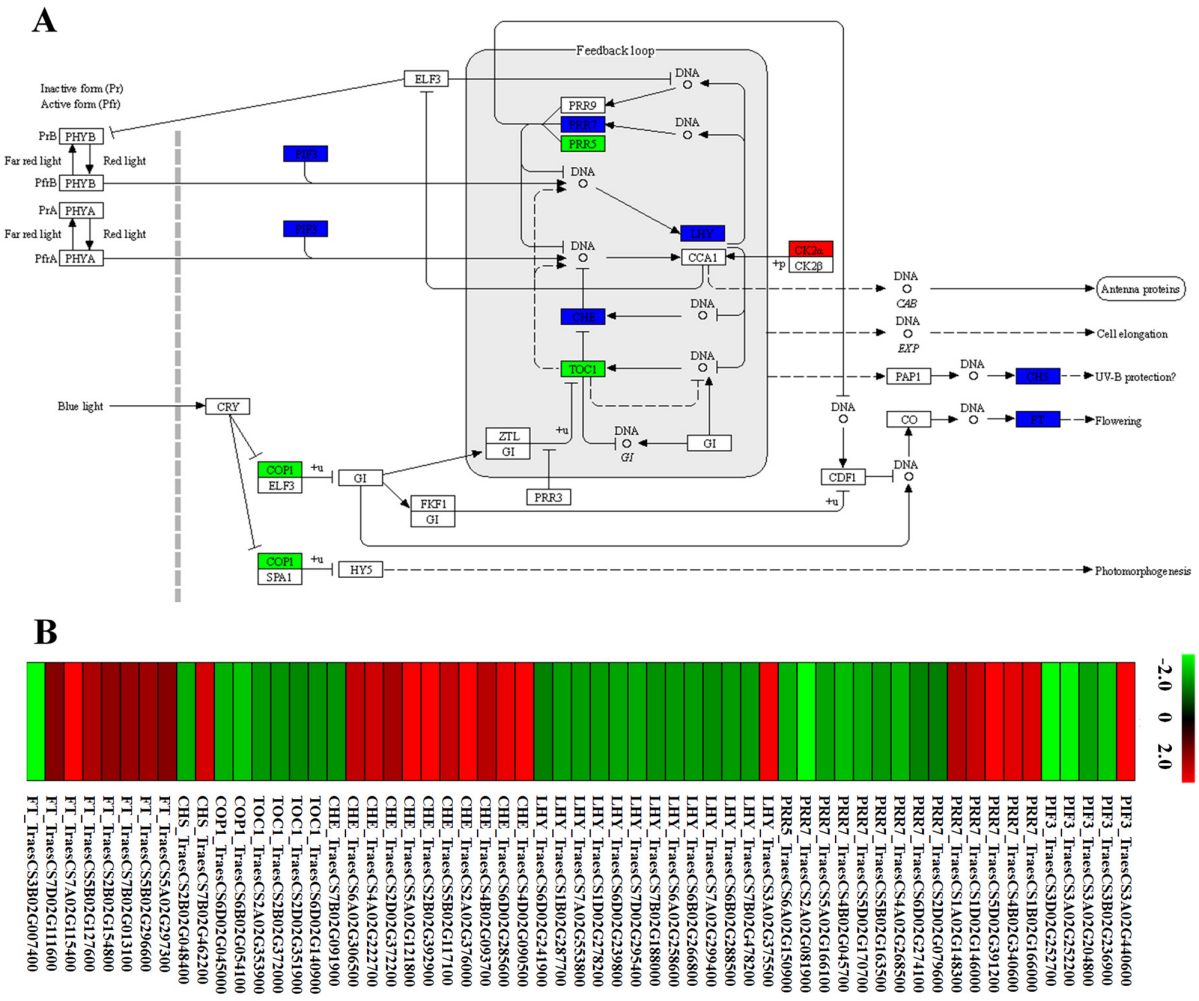
*TaCTR1* overexpression alters circadian rhythm pathway. (**A**) The network of circadian rhythm pathway in *TaCTR1* overexpression transgenic lines, compared with WT plants. The blue indicated significantly differences in gene expression levels, while red (up-regulated) and green (down-regulated) represented specific expression between *TaCTR1* overexpression transgenic lines and WT plants. *PIF3*, phytochrome-interacting factor 3; *PRR*, pseudo-response regulator; *LHY*, MYB-related transcription factor; *CHE*, protein CCA1 HIKING EXPEDITION; *TOC1*, pseudo-response regulator 1; *COP1*, E3 ubiquitin-protein ligase RFWD2; *CK2α*, casein kinase II subunit alpha; *CHS*, chalcone synthase; *FT*, FLOWERING LOCUS T. (**B**) The heatmap of DEGs in circadian rhythm pathway. The red and green indicate high and low expression level, respectively

**Fig. 11:**
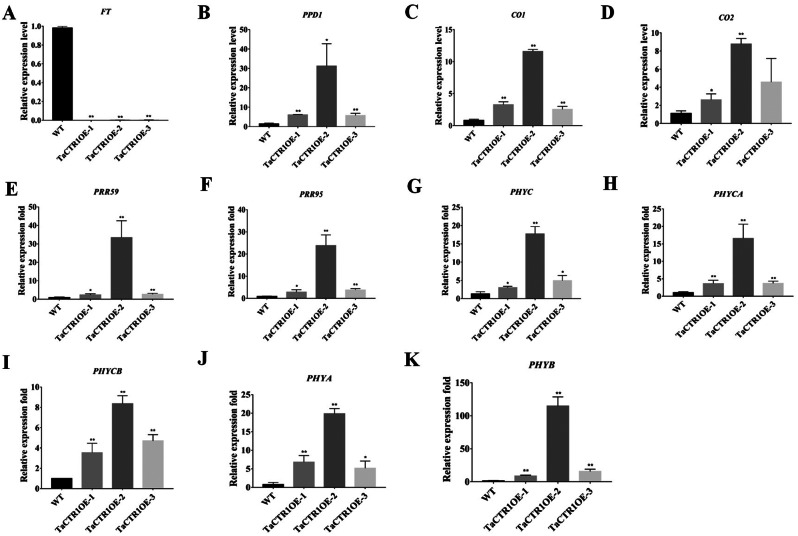
*TaCTR1* overexpression alters expression of flowering-related genes. (**A**) The expression profile of *FT*. (**B**) The expression profile of *PPD1*. (**C**-**D**) The expression profile of *CO*. (**E**-**F**) The expression profile of *PRRs*. (**G**-**K**) The expression profile of *PHY* genes. The wheat gene *18SrRNA* was used as an endogenous control. The gene expression profile were calculated using the 2^–ΔΔCT^ method. All experiments included three replicates

Flowering time is regulated by flowering-related genes, such as *CO*, *FT*, and *PPD1* [3–5]. Our transcriptome data indicated that circadian rhythm pathways were activated in *TaCTR1*-overexpressing transgenic lines, and the expression of 59 circadian rhythm genes, including phytochrome-interacting factor 3 (*PIF3*), pseudoresponse regulator (*PRR*), MYB-related transcription factor (*LHY*), protein CCA1 HIKING EXPEDITION (*CHE*), pseudoresponse regulator 1 (*TOC1*), E3 ubiquitin-protein ligase RFWD2 (*COP1*), casein kinase II subunit alpha (*CK2α*), chalcone synthase (*CHS*), and *FLOWERING LOCUS T* (*FT*), was altered (Fig. 10, Table S11). We also performed qRT‒PCR to analyse the expression levels of known flowering-related genes in the transgenic lines. The results indicated that *TaCTR1* overexpression regulates several flowering-related genes, such as *FT*, *PPD1*, *CO, PRR*, and *PHY*. Compared with those in the WT plants, the transcript levels of *FT* were significantly decreased by approximately 1000-fold in the *TaCTR1*-overexpressing lines (Fig. 11A), whereas the transcript levels of *PPD1*, *CO*, *PRR*, and *PHY* were significantly increased (Fig. 11B–K).

## Discussion

The CTR1-DRK-2 gene family belongs to the PK gene superfamily, which has expanded in the flowering plant lineage [12]. In our 2023 article, we also analysed the phylogeny and expression patterns of the RLK gene family, which belongs to the PK gene superfamily, in wheat and other plants [29]. Based on analyses of the exon‒intron structures and domains in 15 investigated plants, we proposed an evolutionary model of the CTR1-DRK-2 gene family. In brief, this model contains two points: (1) two exon fusion events occurred to form subfamilies II and III–IV during the emergence of Pteridophyta and Angiosperms (Fig. 2), and (2) subfamilies I and II–IV contained different domains (a PAS or PAS_9 domain and an EDR1 domain, respectively) in addition to a PK_Tyr_Ser-Thr domain (Fig. 3).

First, CTR1-DRK-2 subfamily I might have already appeared in a green algae-resembling ancestor. We identified one CTR1-DRK-2 gene (*PNW85595*) in green algae (*C. reinhardtii*), suggesting that the ancestral sequence of the CTR1-DRK-2 gene family might have arisen during the Phycophyta emergence. This sequence contained only two domains (EDR1 and PK_Tyr_Ser-Thr) (Fig. 3), and its exon‒intron structure was algae specific. As displayed in Fig. 3, P. *patens* had only one CTR1-DRK-2 gene (Ppa_Pp3c12_3550V3.2), but its domain arrangement had turned into three domains (PAS, EDR1, and PK_Tyr_Ser-Thr), suggesting that Ppa_Pp3c12_3550V3.2 obtained a new PAS domain during evolution. Moreover, the exon phases from the PK_Tyr_Ser-Thr domain changed to a “1002-0002-02” pattern, which was mostly retained during the evolution of eudicots and monocots. Based on the analysis of phylogenetic trees, the Ppa_Pp3c12_3550V3.2 sequence belongs to subfamily I, suggesting that subfamily I was the ancestral subfamily. Second, subfamilies I and II might have appeared earlier in a fern-resembling ancestor. Subfamily II may be the second subfamily because of its sequences (Smo_EFJ10671 and Smo_EFJ07496) in *S. moellendorffii*. These genes all contained two domains (EDR1 and PK_Tyr_Ser-Thr) that were retained in subfamilies II–IV during evolution. Moreover, the subfamily I sequence (Smo_EFJ04327) in *S. moellendorffii* contained two domains (PAS and PK_Tyr_Ser-Thr). Considering the three domains (PAS, EDR1, and PK_Tyr_Ser-Thr) in the *P. patens* sequence (Ppa_Pp3c12_3550V3.2), the subfamily I sequence (Smo_EFJ04327) in *S. moellendorffii* lost its EDR1 domain during evolution, and subfamily II (Smo_EFJ10671 and Smo_EFJ07496) lost its PAS domain. One exon fusion event occurred in *S. moellendorffii* subfamily II (Smo_EFJ10671 and Smo_EFJ07496) and changed to a “1002-000-02” structure. These structures were conserved from *S. moellendorffii* to *T. aestivum*. Third, the common ancestral sequence of subfamilies III and IV might have appeared during the emergence of angiosperms. Their two domain combinations (EDR1 and PK_Tyr_Ser-Thr) and “1002-0002-2” exon‒intron structure are conserved in both eudicots and monocots.

The PKs are known to function in plant development and growth. For example, the knockdown of *CPK32* has been shown to result in late flowering by altering FLOWERING CONTROL LOCUS A (*FCA*) alternative polyadenylation and FLOWERING LOCUS C (*FLC*) transcription [15]. Another study demonstrated that *CTR2* was involved in rice growth and development [24]. Ethylene plays a crucial role in plant development and growth. In *Arabidopsis*, the ETHYLENE RESPONSE FACTOR1 (*ERF1*) has been shown to delay flowering by inhibiting *FT* expression [30]. *CTR1* has also been reported to mediate the ethylene receptor signal output, and mutations in *CTR1* cause a constitutive ethylene response [31, 32]. In *Arabidopsis*, the Raf-like protein kinase CTR1 negatively regulates ET responses by interacting with the ET receptors ETR1 and ERS [22]. In addition, other phytohormones, such as ABA and IAA, reportedly affect flowering time. A previous study showed that *BrABF3* overexpression promotes flowering time by activating CONSTANS transcription [33]. Overexpression of the AUX/IAA protein TaIAA15-1 A promotes flowering time by activating the ABA signalling pathway and interacting with BdARF16 [34]. Consistent with these reports, our study demonstrated that the overexpression of *TaCTR1* results in early flowering by activating phytohormone pathways and altering the expression profiles of genes involved in those pathways, such as *ETR*, *EIN*, ABA1, AUX/IAA, ABF, ARF and *ERF*, suggesting that *TaCTR1* regulates phytohormone-related genes and thereby affects flowering time. Our functional diversity results showed that type I functional divergence was not a common phenomenon in the evolution of the TKL_CTR1-DRK-2 gene family (Table S4). This suggests that the functions of the TKL_CTR1-DRK-2 genes are conserved and may involve interactions with the ET receptors ETR1 and ERS [22].

Many studies have demonstrated that plant flowering time is regulated by various pathways, such as circadian rhythm and flavonoid biosynthesis [3–7]. A previous study demonstrated that the overexpression of *HvFT4* delays flowering and decreases floret fertility in barley [35]. *FT1* overexpression causes early flowering, and RNAi-mediated knockdown of *FT1* expression results in late flowering [36]. Other studies have shown that PKs result in late flowering by altering *FCA* alternative polyadenylation and *FLC* transcription [15]. In addition, the mutation of chalcone synthase (*CHS*) flavonoid 3’-hydroxylase (*F3’H*) altered the transcriptional activity of two key clock genes, CCA1 and TOC1, in *Arabidopsis* [37]. The results of our study also indicated that the overexpression of *TaCTR1* leads to early flowering by activating circadian rhythm pathways, including *FT*, *PPD1*, *CO*, *PRR*, and *PHY*, and flavonoid biosynthesis, including *CHS*, *F3’H, 4CL*, and *ANR*, suggesting that *TaCTR1* regulates flowering time-related genes and thereby affects flowering time.

## Conclusions

Here, we identified and characterized the role of the tyrosine kinase-like (TKL) gene *TaCTR1* in flowering time. Phylogenetic analysis revealed the occurrence of some exon fusion events during evolution. The overexpression of *TaCTR1* caused early flowering in transgenic wheat through activation of the plant hormone signaling, flavonoid biosynthesis, phenolamides and antioxidant, and flowering-related genes. This work and its findings provide insights on wheat controlling flowering time and the regulatory mechanism while illustrating the potential of *TaCTR1* overexpression in altering flowering time during crop improvement programs.

## Methods and materials

### Plant materials and growth conditions

The wheat (*Triticum aestivum* L.) cultivar “Sumai3” which was sourced from the College of Agronomy (Shandong Agricultural University), was used in this study. The cultivar “JWI” was used for transformation to construct overexpression lines. The transgenic wheat lines and “JWI” were grown at 20–25 °C with a photoperiod of 16 h light/8 h dark in a greenhouse.

### Identification and classification of TKL_CTR1-DRK-2 in plants

The genome and protein sequences of *T. aestivum*, *T. spelta*, *T. turgidum*, *T. dicoccoides*, *T. urartu*, *Ae. tauschii*, *B. distachyon*, *Z. mays*, *Oryza sativa*, *Arabidopsis thaliana*, *Vitis vinifera*, *Amborella trichopoda*, *Selaginella moellendorffii*, *Physcomitrella patens*, and *Chlamydomonas reinhardtii* were downloaded from Ensembl Plant release-51 (http://plants.ensembl.org/). To identify the PKs, all the proteomes of the fifteen plants were scanned by our local server HMMER3.1 (PK_Tyr_Ser-Thr.hmm pfam profile PF07714.19, Pkinase.hmm PF00069.27) and the website pfam 34.0 (http://pfam.xfam.org/) in batch mode with an E value of 0.01. Atypical PRXs with PK_Tyr_Ser-Thr or a Pkinase domain covering less than 50% alignment were excluded in the following analysis. Classifications of “typical” sequences of PK subfamilies were performed by HMMER 3.1 with HMM models developed by Legti-Shiu and Shui [12].

The alignment of truncated TKL_CTR1-DRK-2 sequences with the PK_Tyr_Ser-Thr domain was performed by ClustalX v2.0 [38]. A Bayesian phylogenetic tree was generated using MrBayes v3.2.7 [39] on our local server with the mixed amino acid substitution model. An MCMC chain with 10,000,000 generations was used. Markov chains were sampled every 100 generations, and the first 25% of the trees were discarded as burn-in. The result of MrBayes v3.2.7 was analyzed by TreeGraph v2.14 [40] and our Perl scripts. The ML (Maximum Likelihood) phylogenetic tree was performed using PhyML v3.1 [41] with 100 bootstrap replicates on our local server. The appropriate model of the ML method, including model parameters, was calculated using the Akaike Information Criterion (AIC) with ProtTest v3.4 [42]. The NJ (neighbor-joining) phylogenetic trees were constructed by MEGA-X with a *p*-distance, JTT model, and 1000 bootstrap repetitions.

### Domain and intron–exon structure diagram

The domain and intron–exon structures of TKL_CTR1-DRK-2 sequences in these fifteen plants were generated by our Perl and R scripts based on the corresponding GFF file information from Ensembl Plant release-51 (http://plants.ensembl.org/). The domain information of pfam-A models was downloaded from pfam 34.0 (http://pfam.xfam.org/), and then scanned in our local server.

### Analysis of functional divergence

Type I functional divergence analysis was performed by DIVERGE (version 2.0) software [43].

Type I functional divergence which resulted in altered functional constraints between duplicated genes, leads to one of the genes being conserved and the other gene varying highly. Coefficient of functional divergence (θ) is an indicator for the level of type I functional divergence among two homologous gene clusters. The sites (k) with contribution to the functional divergence were predicted according to their posterior probabilities (Qk). The sites with Qk > 0.67 were meaningful for the functional divergence. We chose 91 sequences (including Pkinase or PK_Tyr_Ser-Thr domain) of eight typical plants (including *C. reinhardtii*, *P. patens*, *S. moellendorffii*, *A. trichopoda*, *(A) thaliana*, *(B) distachyon*, *Ae. tauschii* and *T. aestivum*) to calculate the coefficients of functional divergence.

### Chromosome locations, duplication events and synthetic analysis of wheat TKL_CTR1- DRK-2 genes

Based on the extracted information of GFF files from Ensembl Plants release-51 (http://plants.ensembl.org/), the chromosome locations of *T. aestivum* TKL_CTR1-DRK-2 genes were diagrammed using the software GenomePixelizer [44]. BLASTP was performed against TKL_CTR1-DRK-2 genes and all genes of *T. aestivum*, *B. distachyon*, and *O. sativa* with an E-value of 100. Based on the GFF files and BLAST results, the *Ka* and *Ks* values were calculated by “add_ka_and_ks_to_collinearity.pl” in MCScanX [45]. Based on the GFF files and MCScanX results, synthetic diagrams between *T. aestivum*, *B. distachyon*, and *O. sativa* were generated using our perl scripts and the Circos software (http://circos.ca/).

### Public RNA-seq expression data analysis of wheat TKLs

Public wheat (*T. aestivum*) expression datasets were retrieved from NCBI’s Sequence Read Archive (SRA). The four categories identified from the RNA-seq data of wheat were grain development (Accession: PRJNA525250, ID: 525,250), early wheat spike development (Accession: PRJNA325489, ID: 325,489), four growth stages of wheat flag leaves (Accession: PRJNA656068, ID: 656,068), and wheat early and late heading date (Accession: PRJNA668815, ID: 668,815). Quality control assessment of raw data was performed using FastQC. High-quality RNA-seq reads were aligned to reference wheat (*T. aestivum*) genome of Ensembl Plants release-51 by software Hisat2. Counting of mapped reads was performed using Samtools and HTseq software. The reads per kilobase of exon model per million mapped reads (RPKM) algorithm was used to normalize the data. Heat maps of wheat TKL expression levels were generated using Mev4.9 [46]. The RPKM values of wheat TKLs from all samples are supplied in Table S13.

### Isolation and cloning of *TaCTR1* and its transformation

Total RNA from the wheat cultivar “Sumai 3” leaves was extracted with a TRIzol reagent (Transgen). The full-length cDNA sequence of *TaCTR1* (TraesCS4D01G010200) was obtained from Ensembl plants (http://plants.ensembl.org/index.html), and specific primers were designed based on the obtained sequences. The *TaCTR1* coding sequences were cloned and then sub-cloned into the PC186 vector. The genetic transformation of *TaCTR1* overexpression was performed by the Shandong Academy of Agricultural Sciences.

### Transcriptome sequencing and data analysis

The transcriptome sequencing was conducted on the leaves and stems of JWI and *TaCTR1* overexpression transgenic lines collected at elongation stages. The total RNA of samples was extracted using the RNAprep Pure Plant Kit (Transgen) and was sent to Wuhan Metware Biotechnology Co., Ltd. (Wuhan, China) for RNA sequencing. The transcriptome analysis was performed using an Illumina HiSeq™2000. The clean reads were mapped to the wheat reference genome (http://plants.ensembl.org/Triticum_aestivum/Info/Index) by HISAT2. The differentially expressed genes (DEGs) analysis between JWI and *TaCTR1* overexpression transgenic lines was performed using the DEGseq2 using the thresholds |log_2_ Fold Change| ≥ 1 and false discovery rate (FDR) < 0.05. Kyoto Encyclopedia of Genes and Genomes (KEGG) (https://www.genome.jp/kegg) pathway and Gene Ontology (GO) enrichment analyses were performed on the DEGs [47–49].

### RNA extraction and gene expression analysis

Total RNA of all samples was extracted using RNAprep Pure Plant Kit (Transgen), and then reverse-transcribed into cDNA. The cDNA was used as template for expression analysis. qRT–PCR were performed using the Roche LightCycler ®480 system (Roche, Germany). The wheat gene *18SrRNA* was used as an endogenous control. The gene expression profile were calculated using the 2^–ΔΔCT^ method. All qRT–PCR primers are listed in Table S14.

The bayesian phylogenetic tree was constructed by the amino acid sequences of the PK_Tyr_Ser-Thr domain using MrBayes v3.2.7. (A) Subfamilies I-IV. Detailed information is provided in Supplementary Fig. S1a. (B) Expanded subfamily II and other compressed subfamilies. Our two studied TKL_CTR1-DRK-2 sequences (TraesCS4D02G010200.1 and KQJ91573) in *T. aestivum* and *B. distachyon* were circled by red boxes.

### Electronic supplementary material

Below is the link to the electronic supplementary material.

Supplementary Material 1

Supplementary Material 2

Supplementary Material 3

Supplementary Material 4

Supplementary Material 5

Supplementary Material 6

Supplementary Material 7

Supplementary Material 8

Supplementary Material 9

Supplementary Material 10

Supplementary Material 11

Supplementary Material 12

Supplementary Material 13

Supplementary Material 14

Supplementary Material 15

Supplementary Material 16

Supplementary Material 17

Supplementary Material 18

Supplementary Material 19

Supplementary Material 20

Supplementary Material 21

Supplementary Material 22

Supplementary Material 23

Supplementary Material 24

Supplementary Material 25

Supplementary Material 26

Supplementary Material 27

Supplementary Material 28

Supplementary Material 29

Supplementary Material 30

Supplementary Material 31

## Data Availability

Data availabilityThe genomes, proteomes and GFF files of the investigated plants are available in Ensembl Plants 51-release (http://plants.ensembl.org). The accession numbers of plants are T. aestivum (IWGSC), T. spelta (PGSBv2.0), T. turgidum (Svevo.v1), T. dicoccoides (WEWSeq_v.1.0), T. urartu (ASM34745v1), Ae. tauschii (Aet_v4.0), B. distachyon (v3.0), Z. mays (Zm-B73-REFERENCE-NAM-5.0), O. sativa (IRGSP-1.0), A. thaliana (TAIR10), V. vinifera (12X), A. trichopoda (AMTR1.0), S. moellendorffii (v1.0), P. patens (Phypa_V3) and C. reinhardtii (v5.5). Public wheat (T. aestivum) transcriptome expression datasets were retrieved from the SRA database of NCBI. The SRA accession numbers of transcriptomes are 525250, 325489, 656068 and 668815. All data generated or analyzed during this study are included in this published article and its supplementary information files. The raw RNA-Seq data in this manuscript have been deposited in NCBI (BioProject: PRJNA1002999). All figure and additional files have been obtained appropriate copyright permission of KEGG.
