# Supplementary material for: Genome-wide evolutionary analysis of TKL_CTR1-DRK-2 gene family and functional characterization reveals that TaCTR1 positively regulates flowering time in wheat
Source: BMC Genomics. 2024 May 14;25:474. doi: 10.1186/s12864-024-10383-2 (PMC11092142; doi:10.1186/s12864-024-10383-2)
Supplement: Supplementary file 1 — Supplementary Material 1 [file 12864_2024_10383_MOESM1_ESM.docx]

**Additional file:**

**Fig. S1 Expanded phylogenetic classification of TKL_CTR1-DRK-2 using four phylogenetic method**

(A) Bayes; (B) ML with LG+G model; (C) NJ with JTT model; (D) NJ with p-distance model;

**Fig. S2 Exon−intron diagrams of TKL_CTR1-DRK-2 in 15 plants**

These diagrams indicated the exon–intron structures of TKL_CTR1-DRK-2 in 15 plants (see Method). The descriptions of domain and exon phases are the same as in Fig. 2.

**Fig. S3 Conserved exon−intron and domain diagrams of TKL_CTR1-DRK-2 subfamilies I-**I**V in *C. reinhardtii*, *P. patens, S. moellendorffii, A. trichopoda*, *V. vinifera*, *B. distachyon and T. aestivum***

The descriptions of domain and exon phases are the same as in Fig. 2. The lengths of the boxes and lines are scaled based on the length of the genes.

**Fig. S4. The member distributions of TKL_CTR1-DRK-2 I-IV subfamilies in Triticum species.**

The yellow boxes display one-fold relationship of TKL_CTR1-DRK-2 I-IV subfamilies in *Aegilops tauschii* and *Triticum urartu*. The blue boxes display two-fold relationship in *Triticum dicoccoides* and *Triticum turgidum*. Similarly, the red boxes display three-fold relationship in *Triticum spelta* and *Triticum aestivum*.

**Fig. S5 Alightment of exon fusion blocks with amino acid and nucleotide sequences**

1. Subfamily I to II. (B) Subfamily I to III-IV.

**Fig. S6 Domain diagrams of TKL_CTR1-DRK-2 in 15 plants**

Filled boxes: purple represents PK_Tyr_Ser-Thr domain; other colors represent various domains labeled in each page. The lengths of the boxes and lines are scaled based on the length of proteins (X axis).

**Fig. S7. Phylogenetic tree and alignment among Pkinase domain sequences of selected 91 TKL_CTR1-DRK-2 genes.**

(A) Phylogenetic neighbor-joining (NJ) tree with p-distance model. (B) Alignment.

**Fig. S8. Site-specific profile for evolution rate change.**

(A) I/II (B) I/III (C) I/IV (D) II/III (E) II/IV (F) III/IV

**Fig. S9 Chromosome locations of TKL_CTR1-DRK-2 genes in *T. aestivum*.**

**Fig. S10 Collinearity (*Ks* values) of TKL_CTR1-DRK-2 genes among *T. aestivum*, *B. distachyon* and *O. sativa*.**

1. Collinearity events of duplicated TKL_CTR1-DRK-2 or all genes between the *T. aestivum* ABD sub-genomes. The red bars denote the collinearity events contributed by polyploidizations (*Ks* values 0.0–0.2). (B) Collinearity events of duplicated TKL_CTR1-DRK-2 or all genes between *T. aestivum* and *B. distachyon.* The green bars denote the collinearity events contributed by polyploidizations (*Ks* values 0.25–0.4). (C) Collinearity events of duplicated TKL_CTR1-DRK-2 or all genes between *T. aestivum* and *O. sativa*. The pink bars denote the collinearity events contributed by polyploidizations (*Ks* values 0.45–0.6). The blue bars denote the other collinearity events.

**Fig. S11 Heat map of the expression patterns of all *T. aestivum* TKL genes during development stages.** Normalized gene expression RPKM values of all *T. aestivum* TKL genes. The descriptions of (A)-(D) are the same as in Table S6. Our studied TKL gene (II_TraesCS4D02G010200) is circled by red box.

**Fig. S12 The tissue expression pattern of *TaCTR1.*** The wheat gene *18SrRNA* was used as an endogenous control. The gene expression profile were calculated using the 2^–ΔΔCT^ method. All experiments included three replicates.

**Fig. S13 The expression profile of *TaCTR1* among different wheat cultivars at seeding stage, heading stage, flowering stage.**

**Fig. S14. Information of gene *TaCTR1* (Ensembl pep Id: TraesCS4D02G010200.1).**

(A)Protein sequence (B) Coding sequence (CDS) (C) Chromosome information (D) GFF information (E) Exon−intron and kinase domain diagram of T.ae TKL_CTR1−DRK−2 II subfamily (including TaCTR1) (F) BLAST result.

**Fig. S15 Transcriptom changes in *TaCTR1* overexpression transgenic lines and WT plants.** (A) Heatmap of DEGs in *TaCTR1* overexpression transgenic lines and WT plants. (B) GO enrichment. (C) KEGG classification.

**Fig. S16 *TaCTR1* overexpression alters ABA pathway.** The network of ABA pathway in *TaCTR1* overexpression transgenic lines, compared with WT plants. *ctrB*, 15-cis-phytoene synthase; *crtH*, prolycopene isomerase; *lcyB*, lycopene beta-cyclase; *ABA1*, zeaxanthin epoxidase; *ABA2*, xanthoxin dehydrogenase; *NCED*, 9-cis-epoxycarotenoid dioxygenase; *AAO3*, abscisic-aldehyde oxidase; *SnRK2*, serine/threonine-protein kinase SRK2; *PP2C*, protein phosphatase 2C; *PYL*, abscisic acid receptor PYR/PYL family.

**Table S1. HMR classification of TKL_CTR1-DRK-2 family proteins in 15 plants.**

**Table S2. Comparison of copy number of TKL_CTR1-DRK-2 family proteins in 15 plants.**

**Table S3. The classification of TKL_CTR1-DRK-2 subfamily of protein kinase gene family in 15 plants based on the four phylogenetic trees.**

**Table S4. Estimates of the coefficient of typeⅠfunctional divergence (θ) among Pkinase domain sequences of 91 TKL_CTR1-DRK-2 genes.**

Abbreviation, θ: coefficient of typeⅠfunctional divergence, S.E.(ML): standard error of the estimate theta, LRT: 2 log-likelihood-ratio against the null hypothesis of theta=0.

**Table S5. The posterior probabilities of sites for type I functional divergence among Pkinase domain sequences of 91 TKL_CTR1-DRK-2 genes.**

**Table S6. Chromosome locations of *T. aestivum* TKL_CTR1-DRK-2 genes.**

**Table S7. Collinearity events and *Ka/K*s values of TKL_CTR1-DRK-2 genes among *T. aestivum*, *B. distachyon* and *O. sativa*.**

Sheet 1 was *Ka/Ks* values of collinearity events in *T. aestivum* TKL_CTR1-DRK-2 genes; sheet 2 was *Ka/Ks* values of collinearity events in all *T. aestivum* genes. Similarly, sheets 3-4 showed *T. aestivum* and *B. distachyon*. Sheets 5-6 showed *T. aestivum* and *O. sativa*.

**Table S8. Public wheat RNA-seq expression data to use.**

**Table S9. Detailed information for all detected DEGs.**

**Table S10. GO and KEGG pathway analysis of DEGs.**

**Table S11. The gene information in different pathways.**

**Table S12. The flavonoid contents in *TaCTR1* overexpression lines and WT plants.**

**Table S13. Normalized gene expression RPKM values of TKL_CTR1-DRK-2 subfamily of protein kinase gene family in 4 transcriptomes of wheat.**

**Table S14. Primers used for analysis.**
