# Supplementary figures and images for: Genome-wide evolutionary analysis of TKL_CTR1-DRK-2 gene family and functional characterization reveals that TaCTR1 positively regulates flowering time in wheat
Source: BMC Genomics. 2024 May 14;25:474. doi: 10.1186/s12864-024-10383-2 (PMC11092142; doi:10.1186/s12864-024-10383-2)

A

Bayes

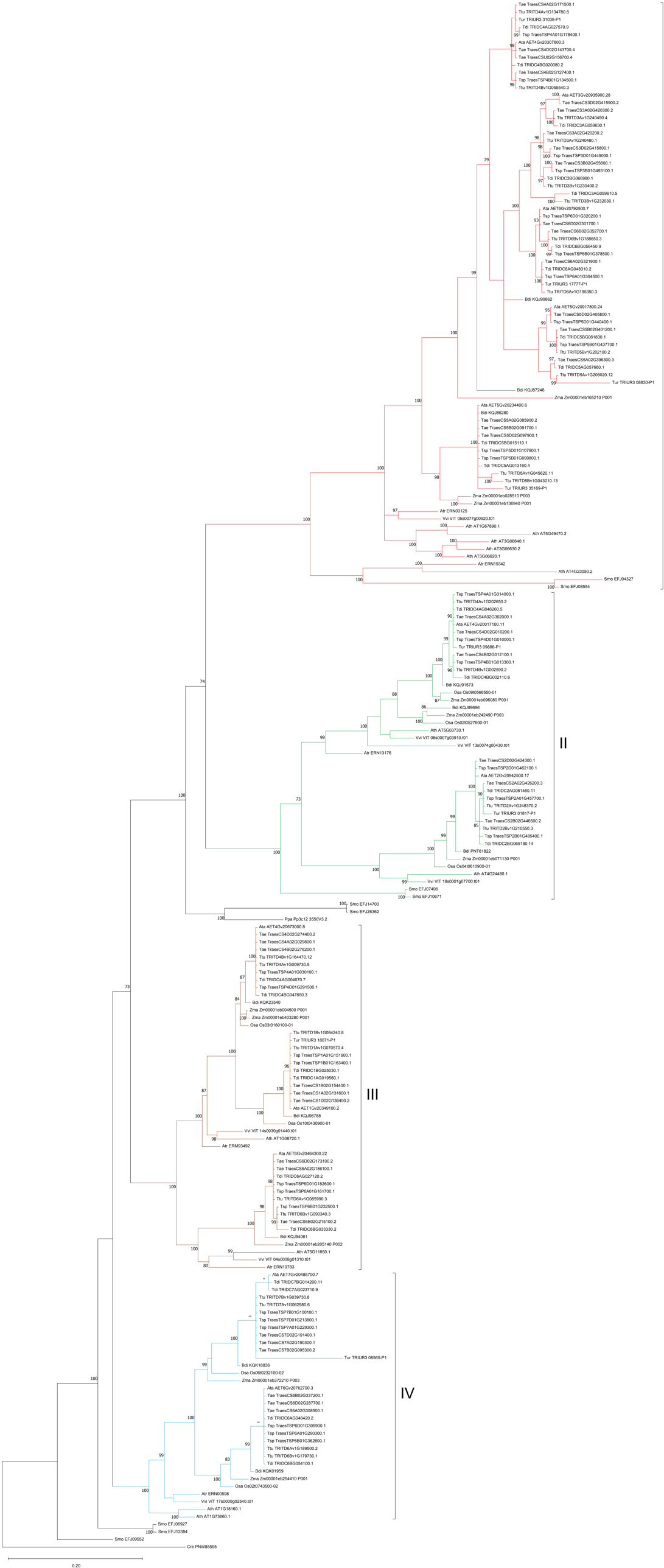

# B ML (LG+G)

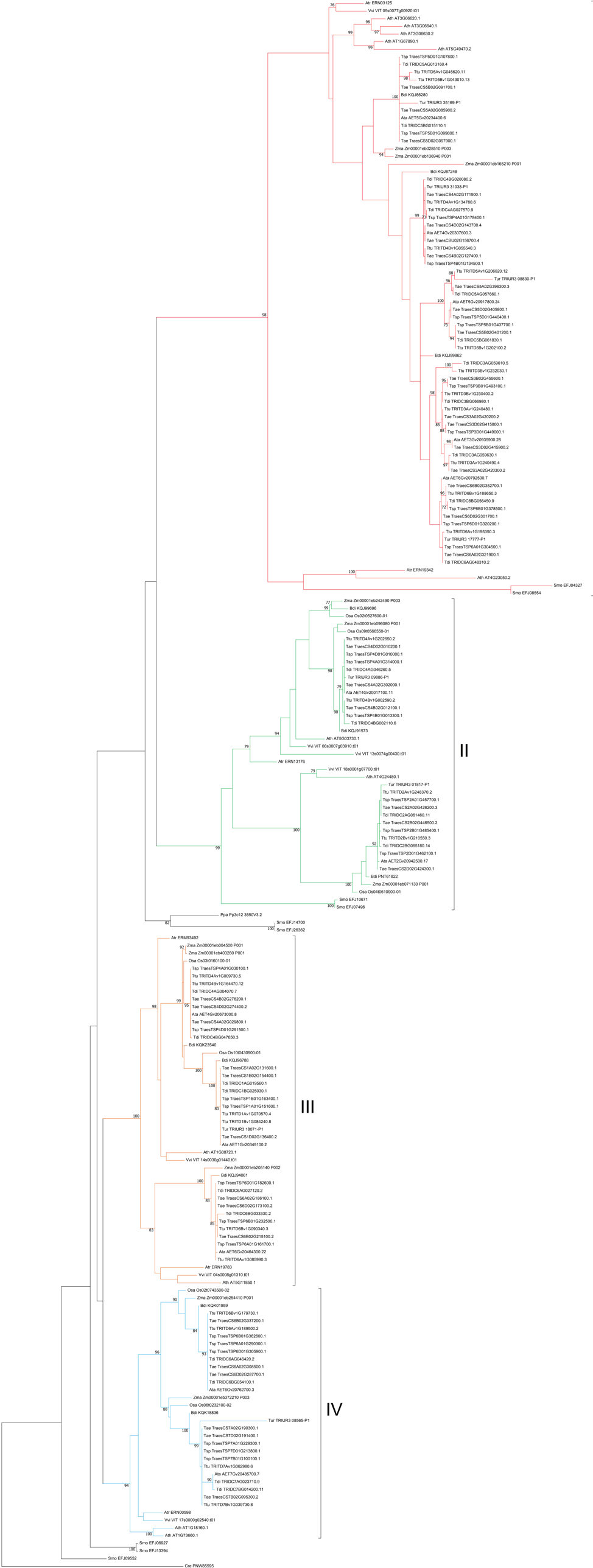

0.50

# C NJ (JTT)

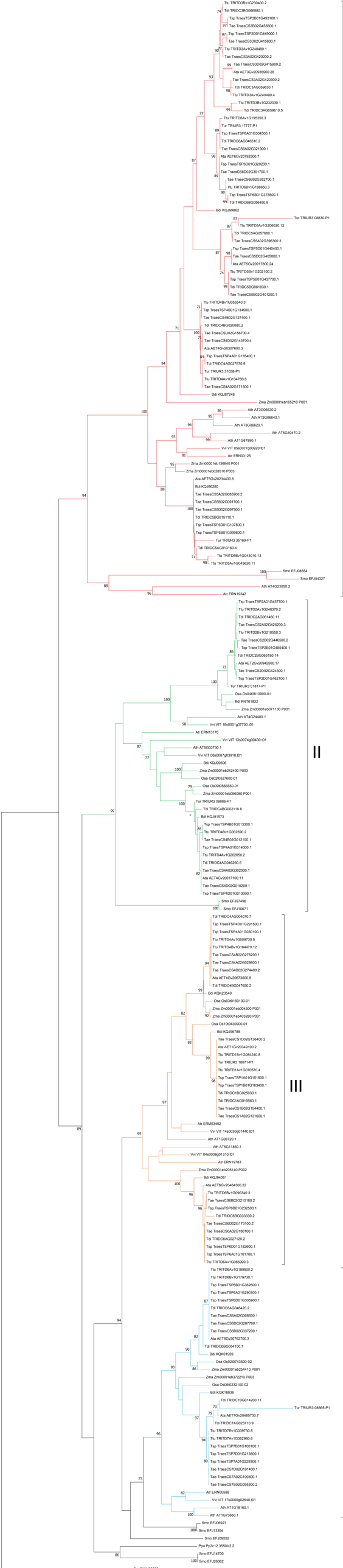

I

II

III

IV

# D NJ (p-distance)

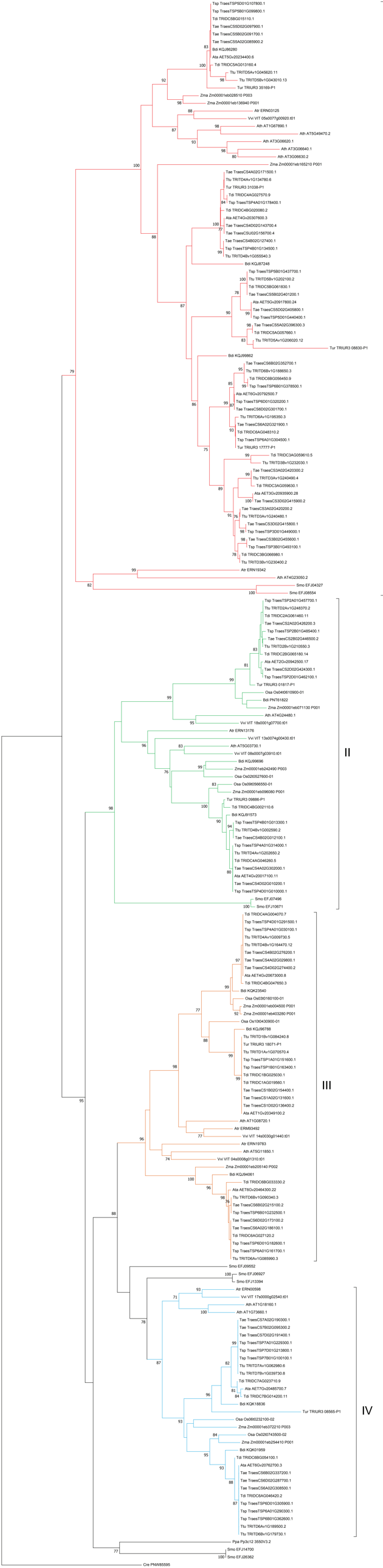

Supplement: Supplementary file 2 — Supplementary Material 2 [file 12864_2024_10383_MOESM2_ESM.pdf]

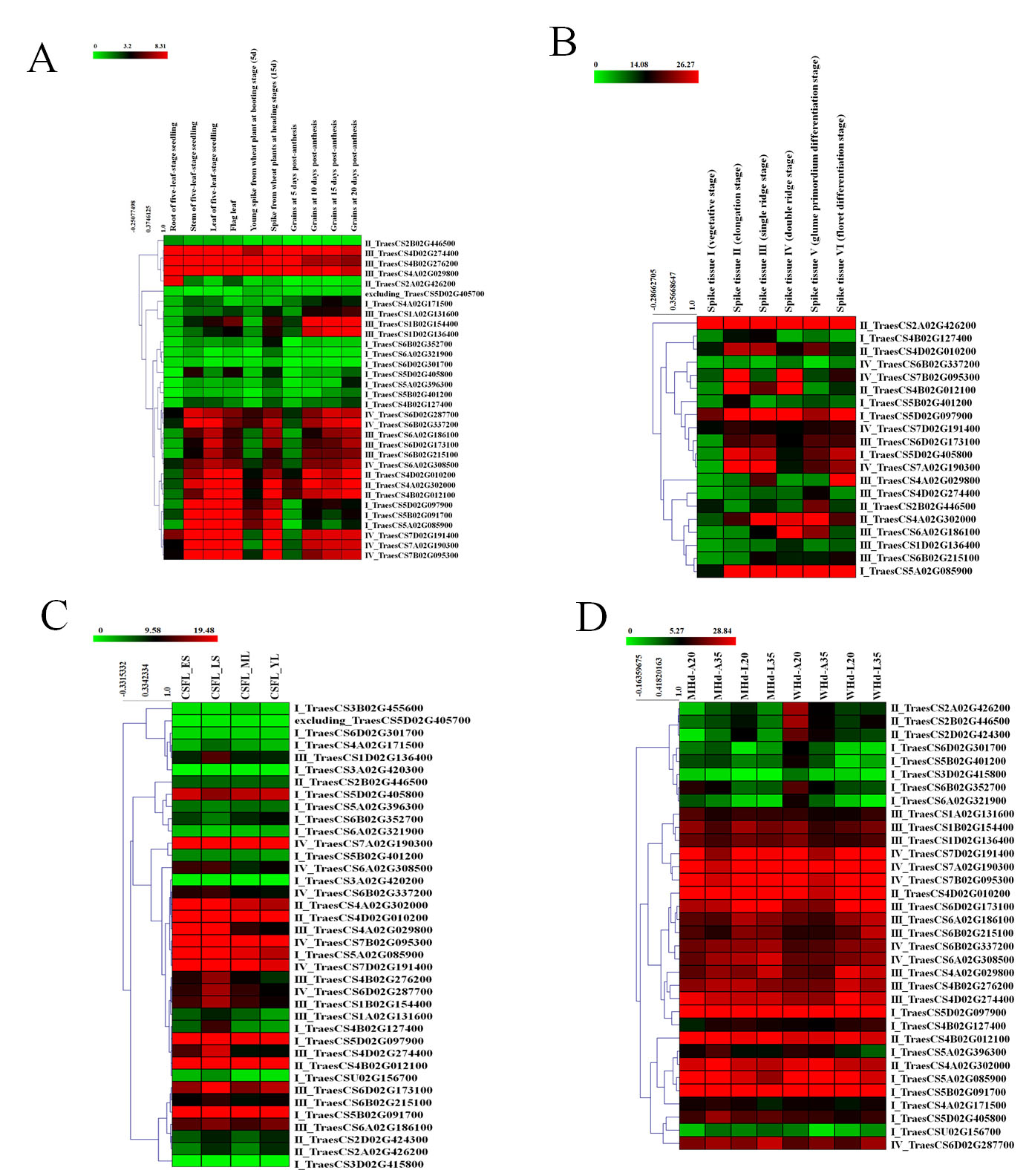

Supplement: Supplementary file 4 — Supplementary Material 4 [file 12864_2024_10383_MOESM4_ESM.jpg]

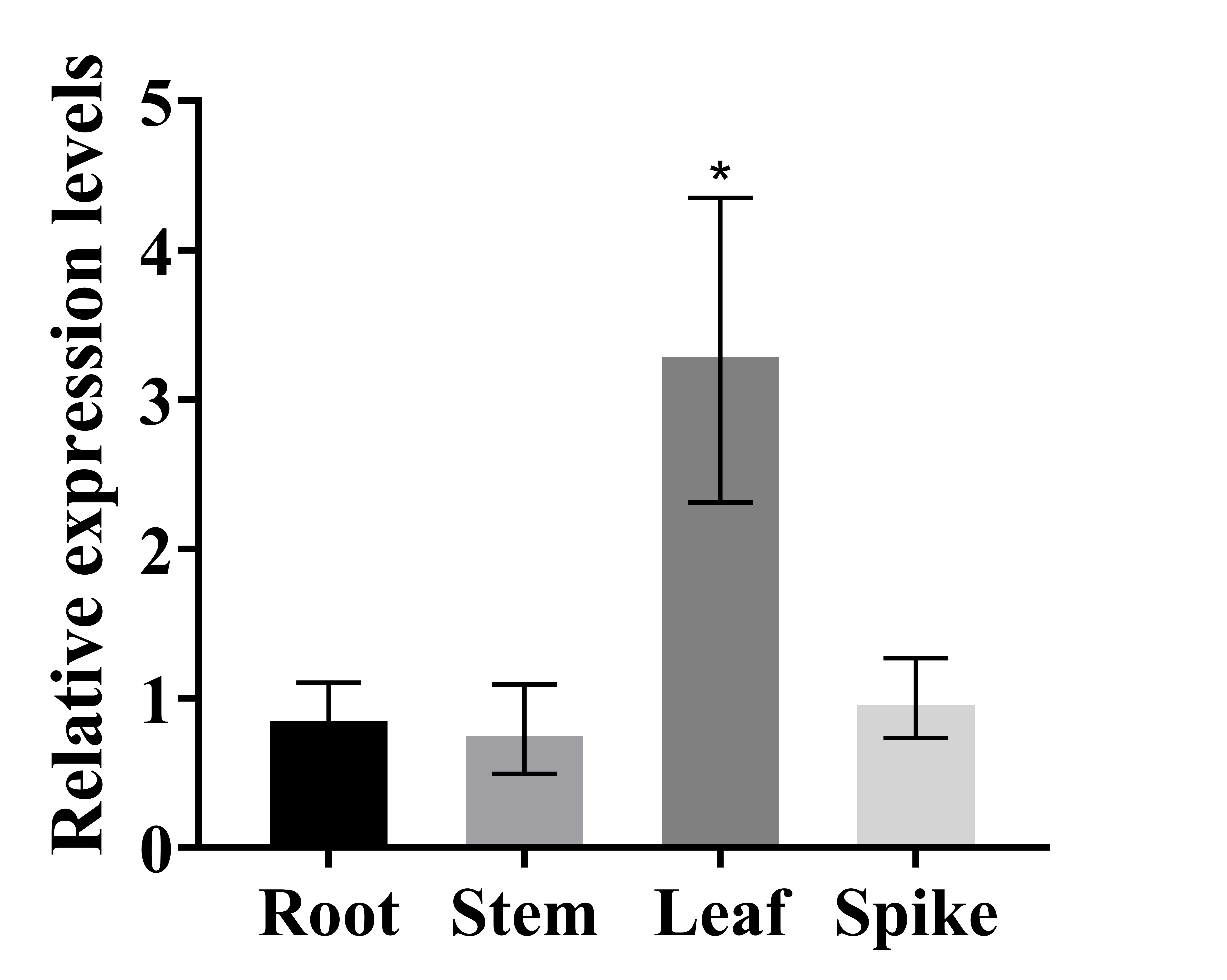

Supplement: Supplementary file 5 — Supplementary Material 5 [file 12864_2024_10383_MOESM5_ESM.jpg]

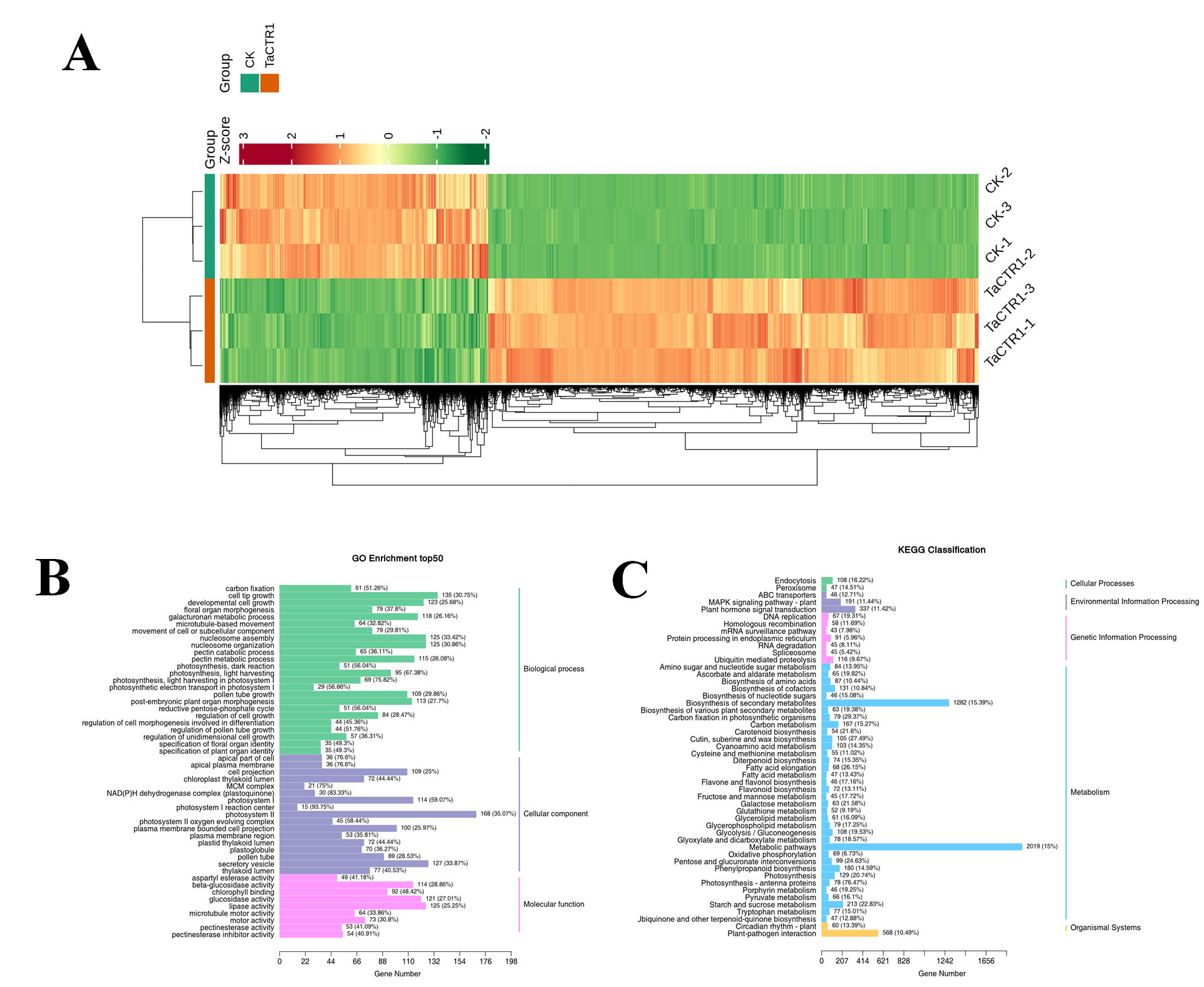

Supplement: Supplementary file 8 — Supplementary Material 8 [file 12864_2024_10383_MOESM8_ESM.jpg]

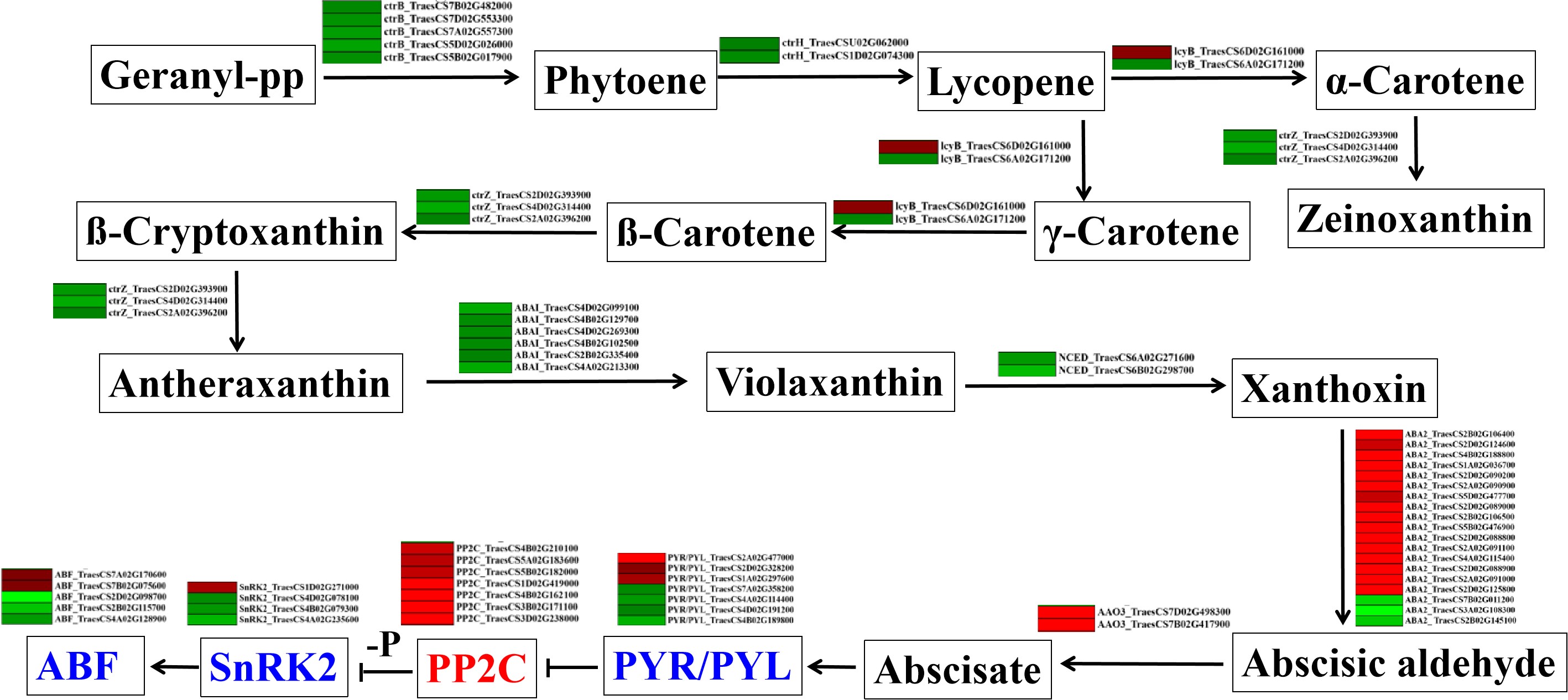

Supplement: Supplementary file 9 — Supplementary Material 9 [file 12864_2024_10383_MOESM9_ESM.jpg]

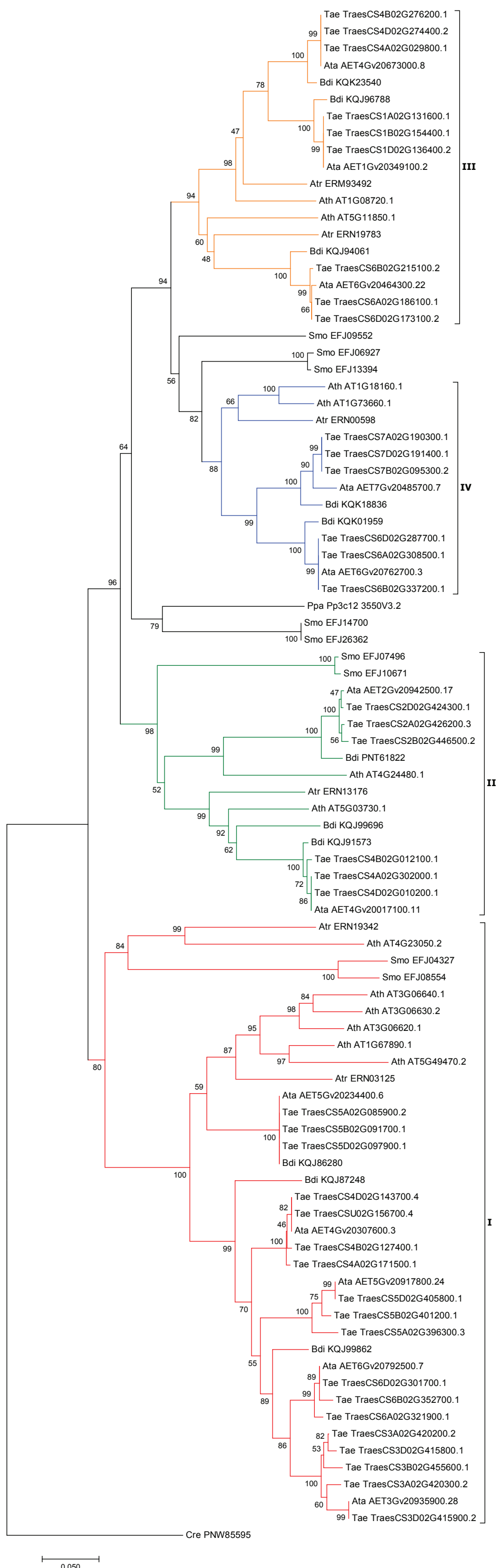



[illegible]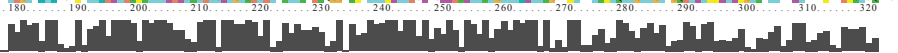

Supplement: Supplementary file 15 — Supplementary Material 15 [file 12864_2024_10383_MOESM15_ESM.pdf]

A

I/II

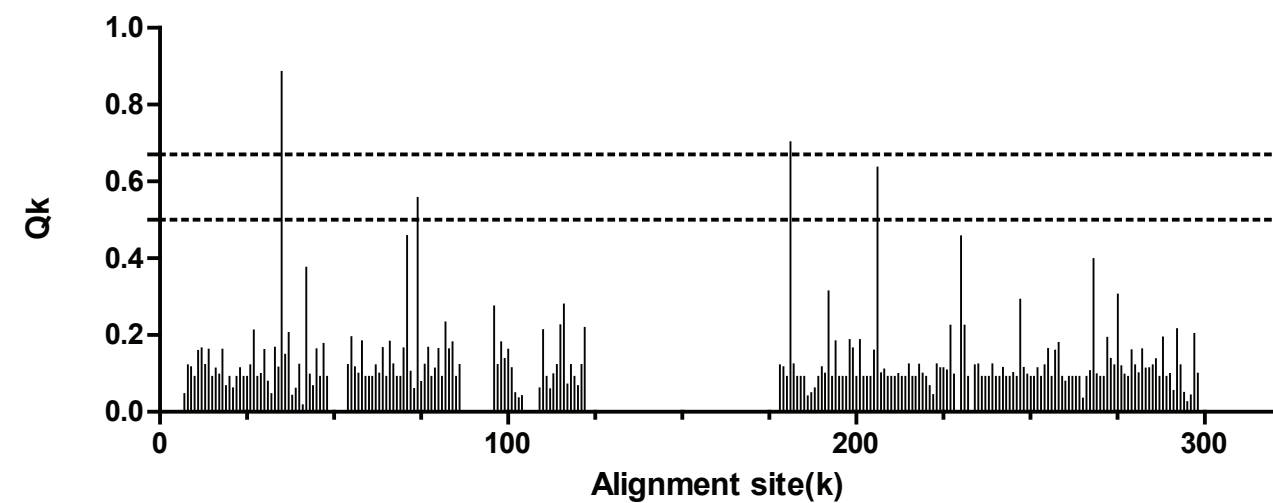

B

I/III

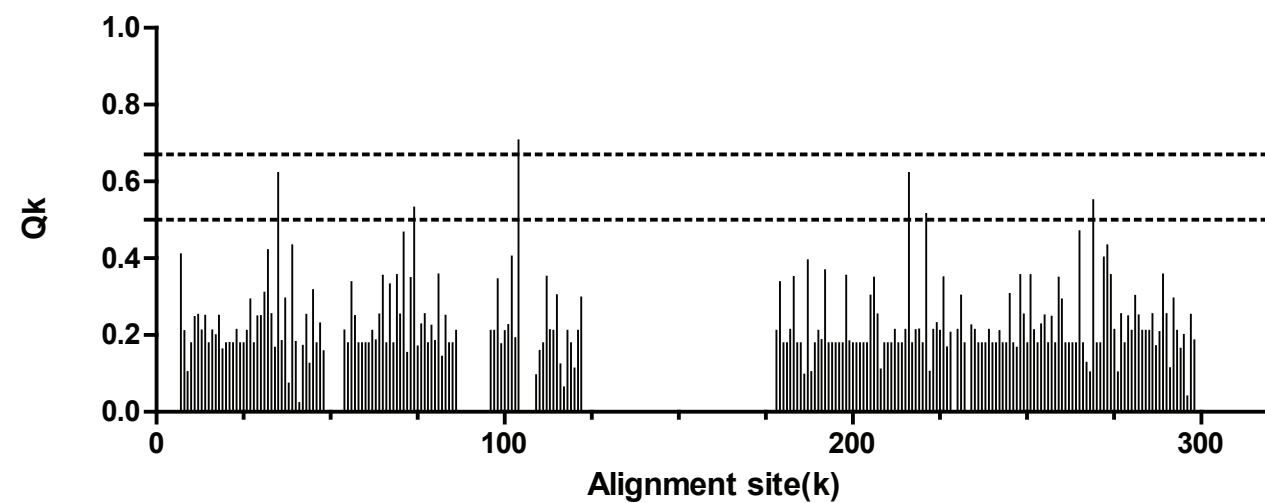

C

I/IV

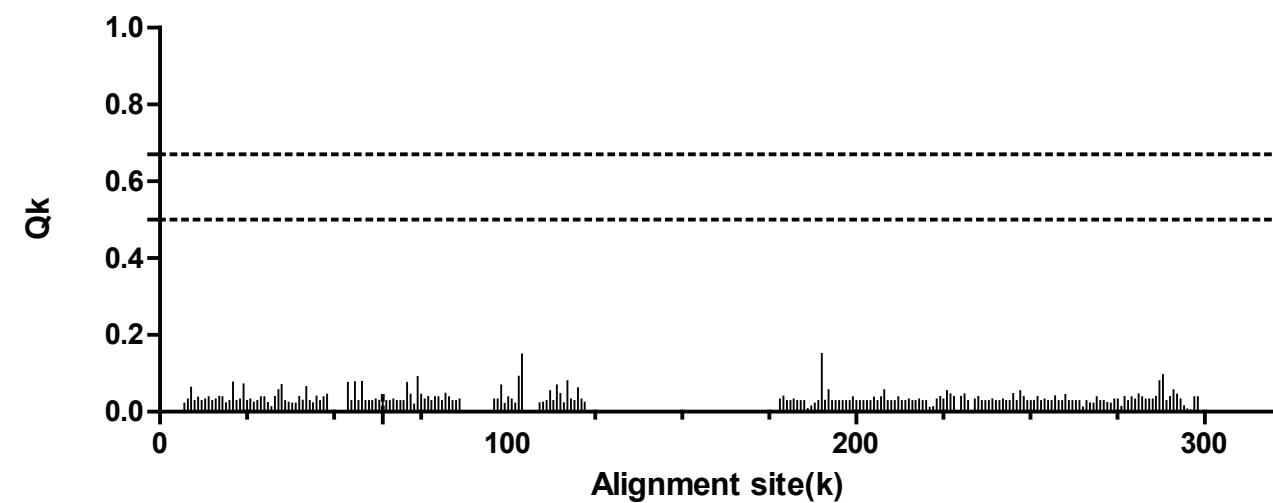

D

II/III

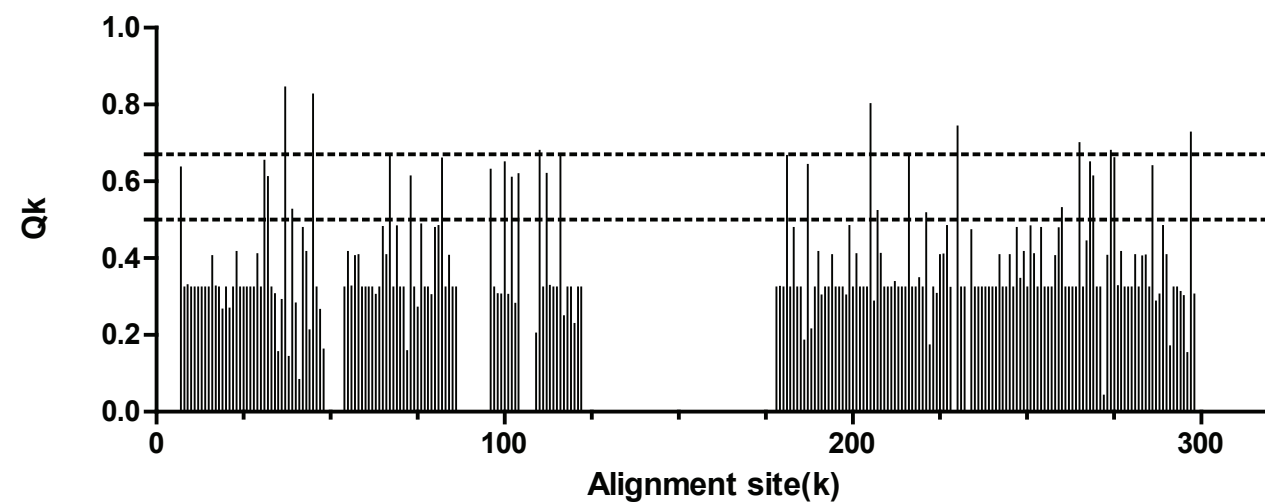

E

II/IV

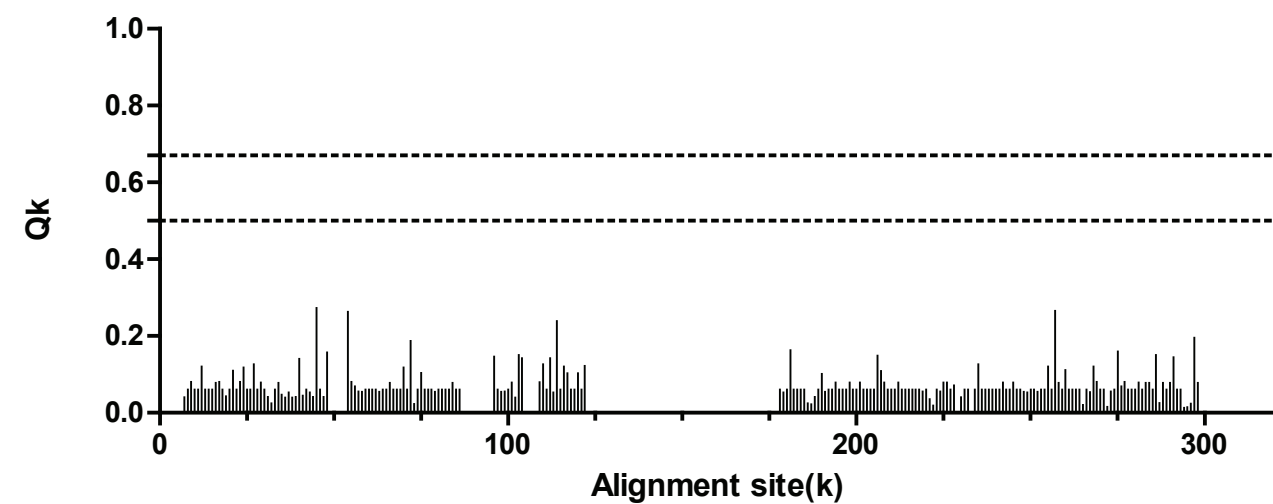

F

III/IV

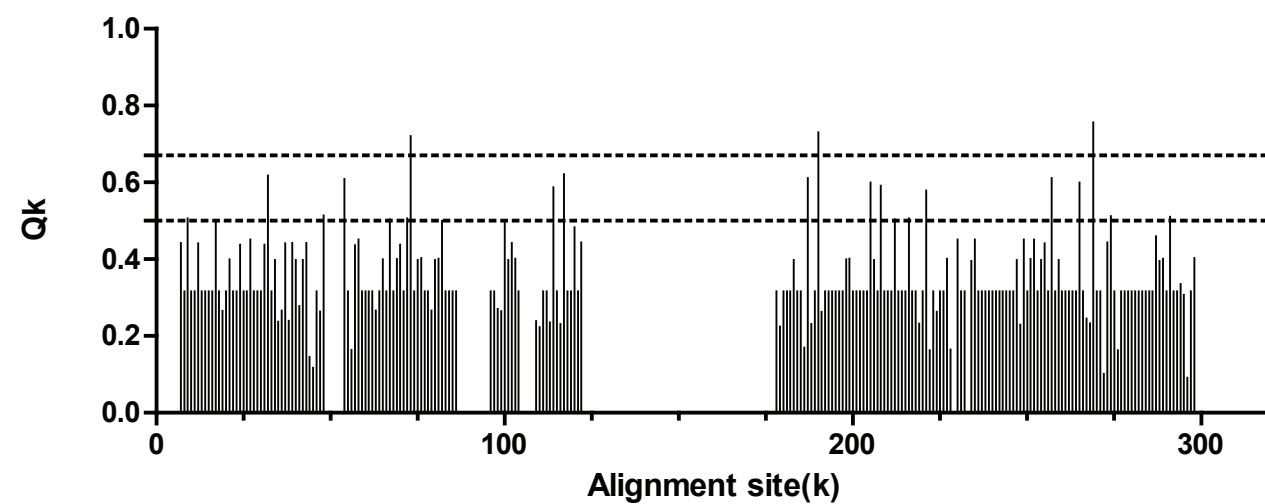

Supplement: Supplementary file 16 — Supplementary Material 16 [file 12864_2024_10383_MOESM16_ESM.pdf]

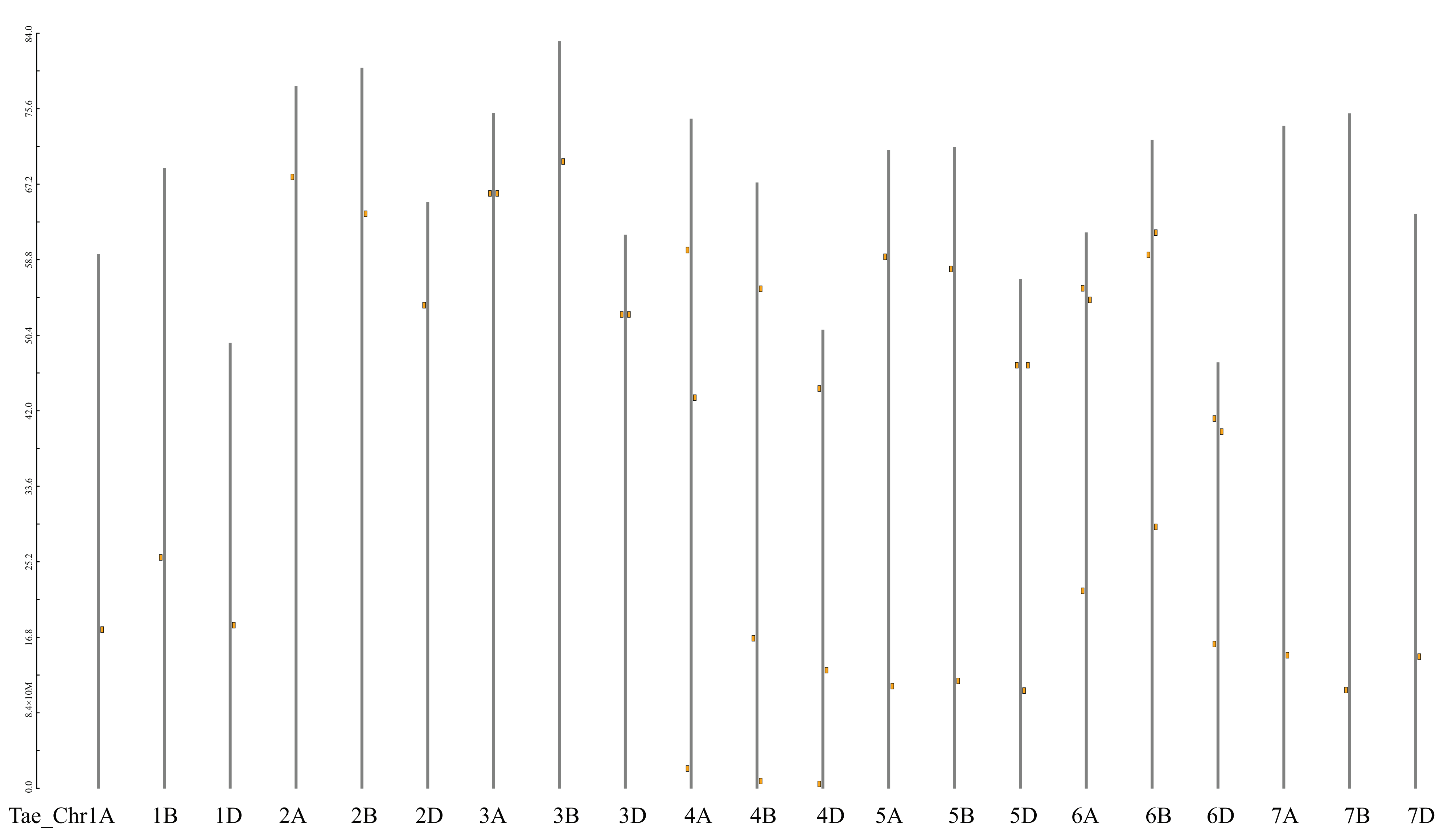

Supplement: Supplementary file 17 — Supplementary Material 17 [file 12864_2024_10383_MOESM17_ESM.pdf]
