# Supplementary material for: Genome-wide evolutionary analysis of TKL_CTR1-DRK-2 gene family and functional characterization reveals that TaCTR1 positively regulates flowering time in wheat
Source: BMC Genomics. 2024 May 14;25:474. doi: 10.1186/s12864-024-10383-2 (PMC11092142; doi:10.1186/s12864-024-10383-2)

A

Collinearity (*Ks* values) of TKL_CTR1-DRK-2 genes between *T. aestivum* ABD sub-gsenomes

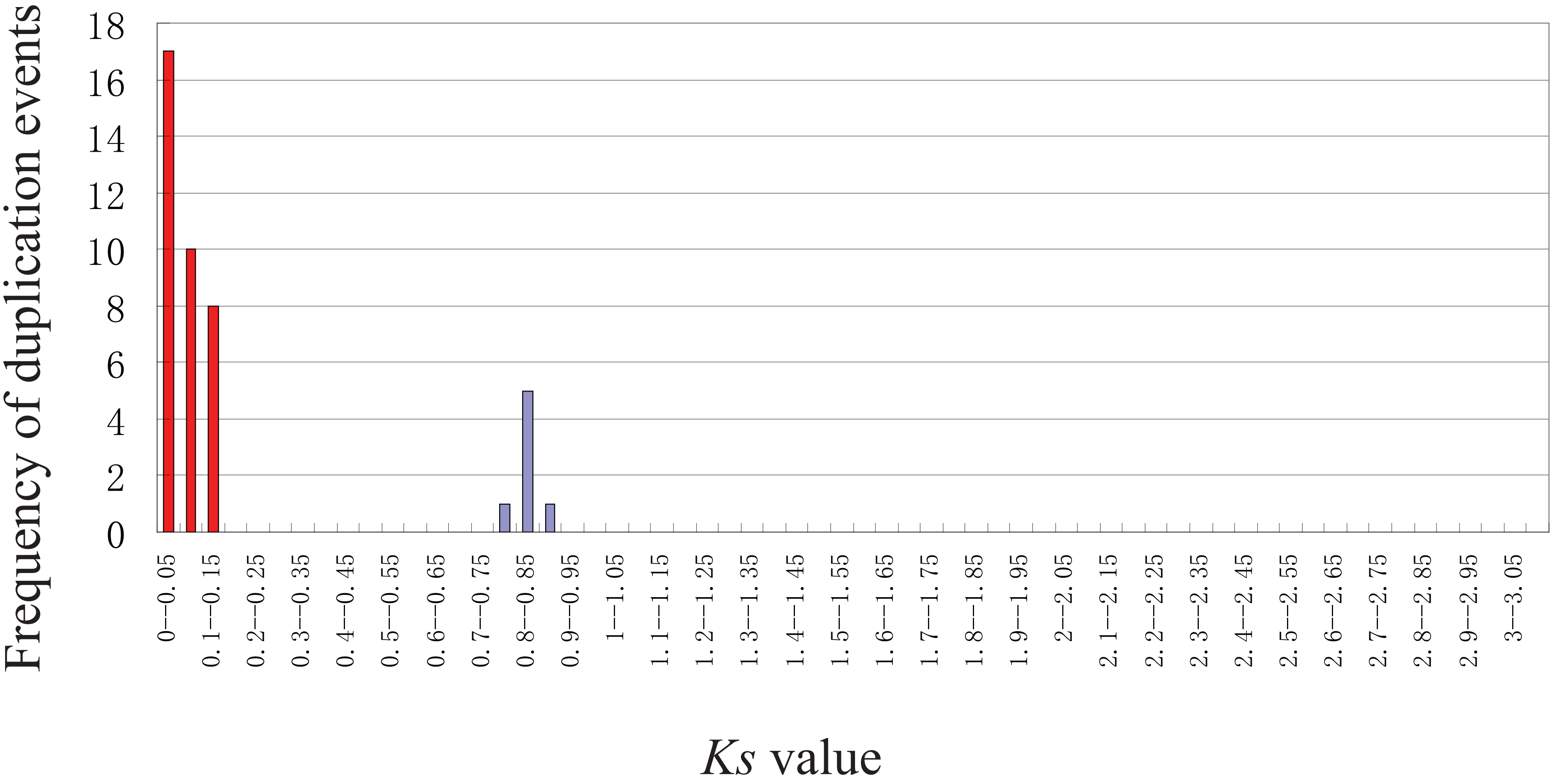

Collinearity (*Ks* values) of all genes between *T. aestivum* ABD sub-genomes

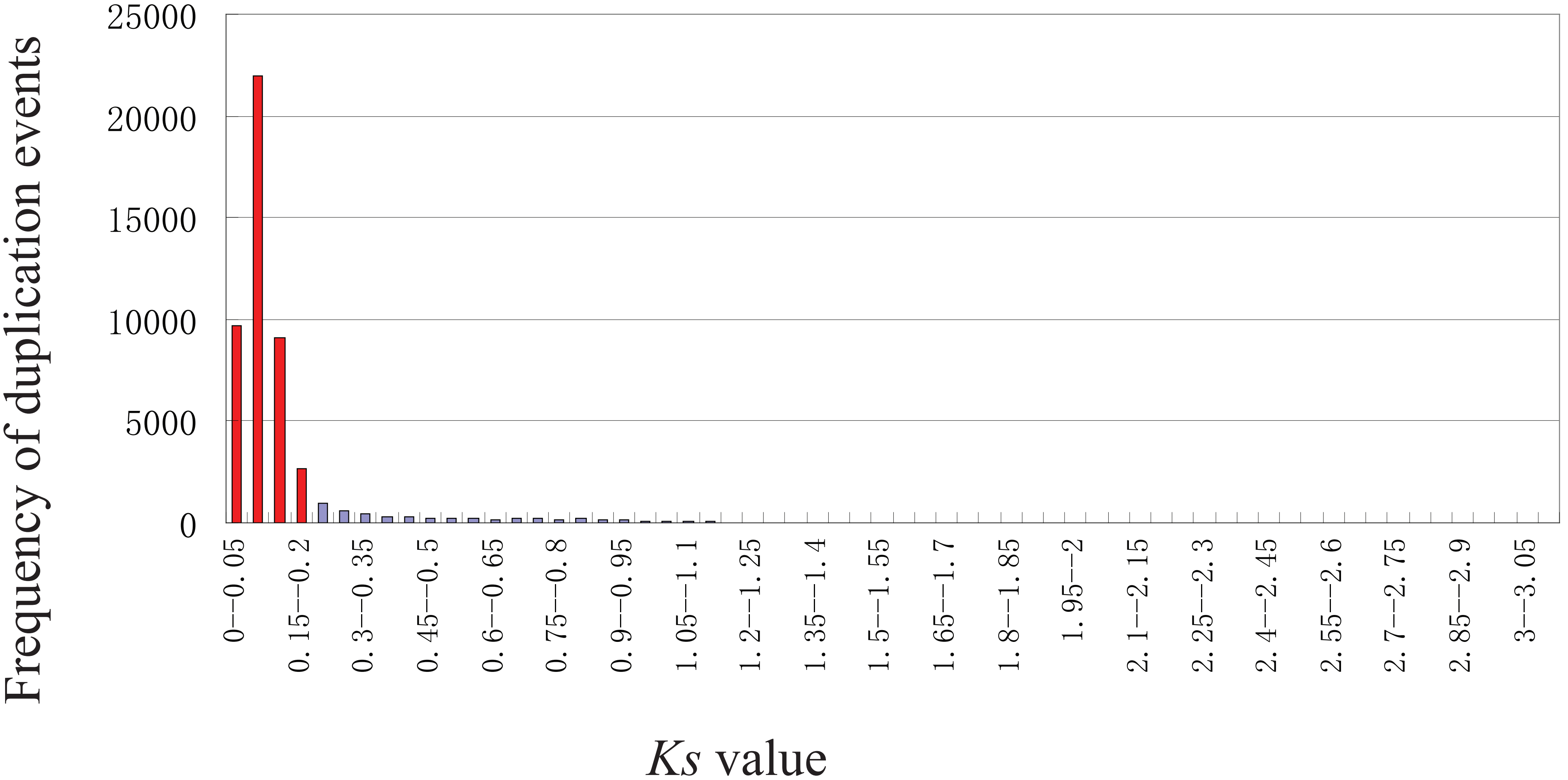

B

Collinearity (*Ks* values) of TKL_CTR1-DRK-2 genes between *T. aestivum* and *B. distachyon*

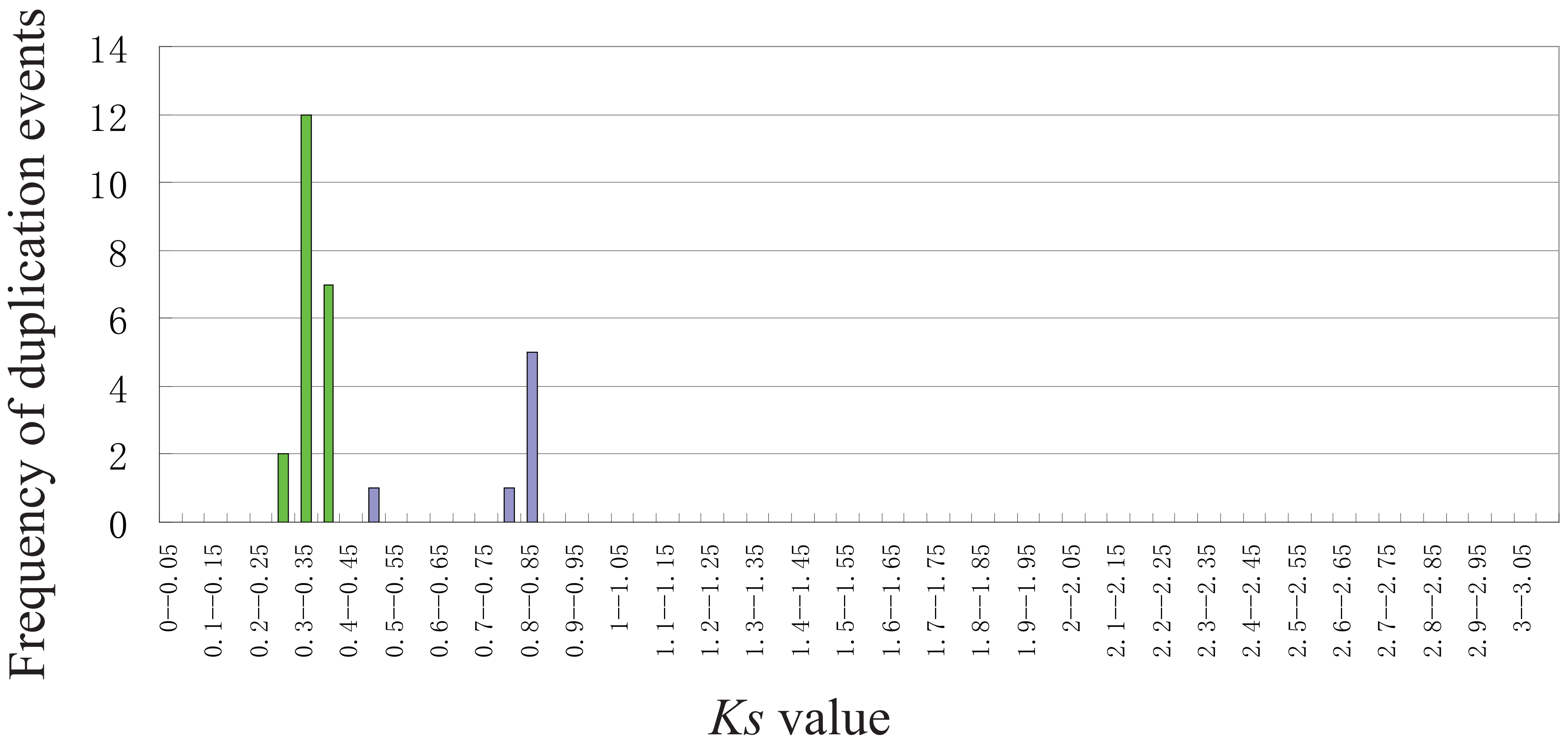

Collinearity (*Ks* values) of all genes between *T. aestivum* and *B. distachyon*

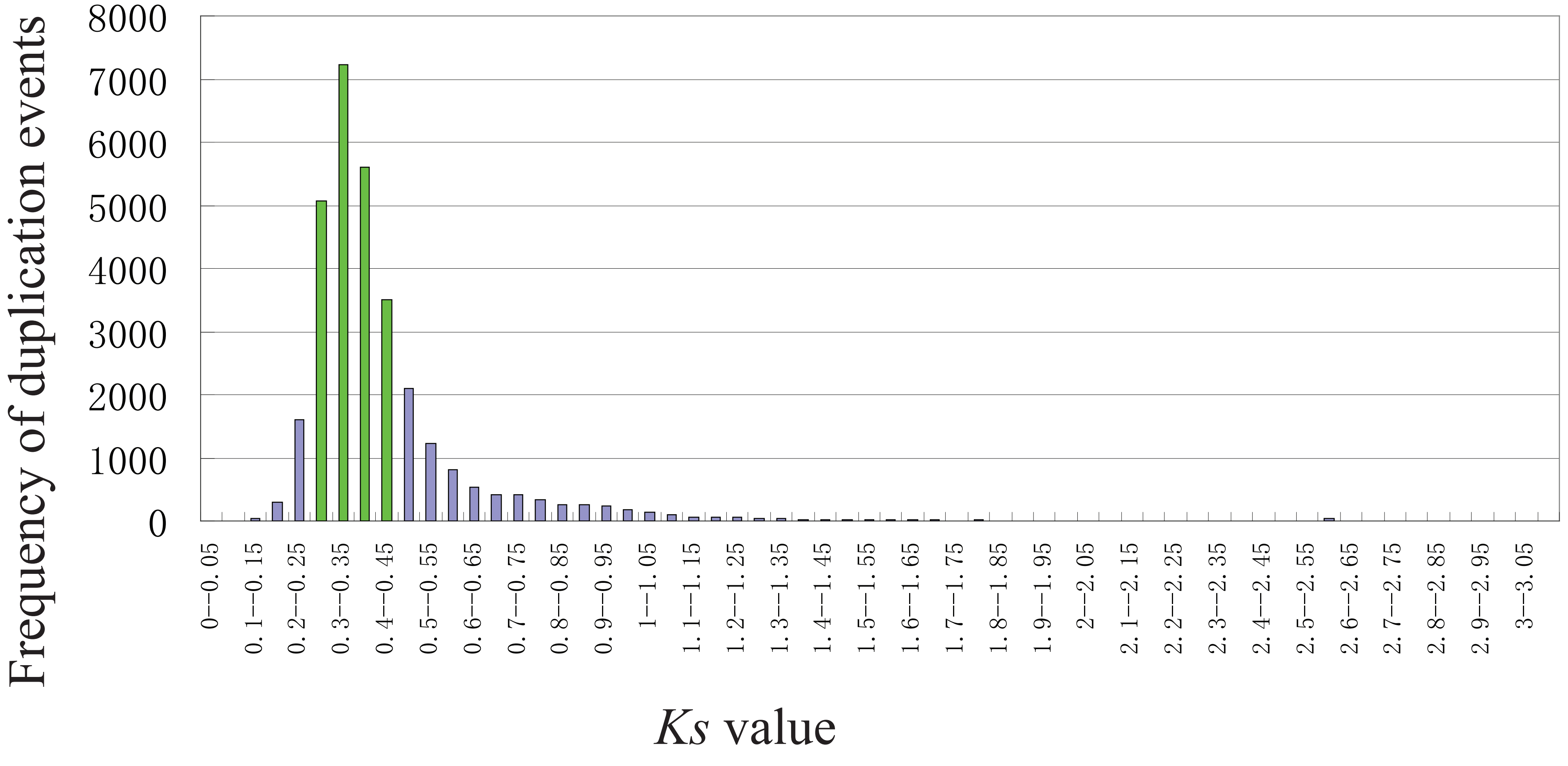

C

Collinearity (*Ks* values) of TKL_CTR1-DRK-2 genes between *T. aestivum* and *O. sativa*

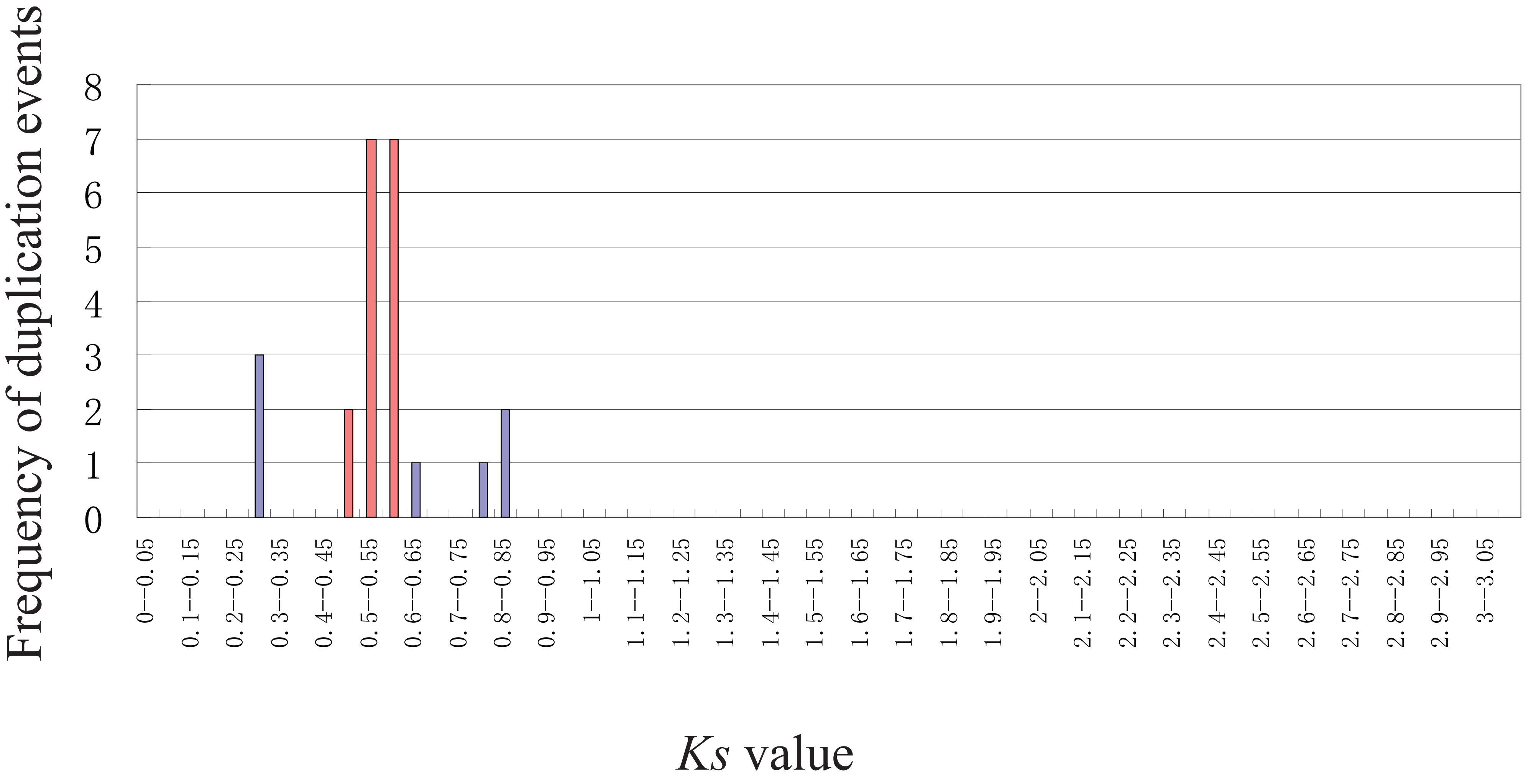

Collinearity (*Ks* values) of all genes between *T. aestivum* and *O. sativa*

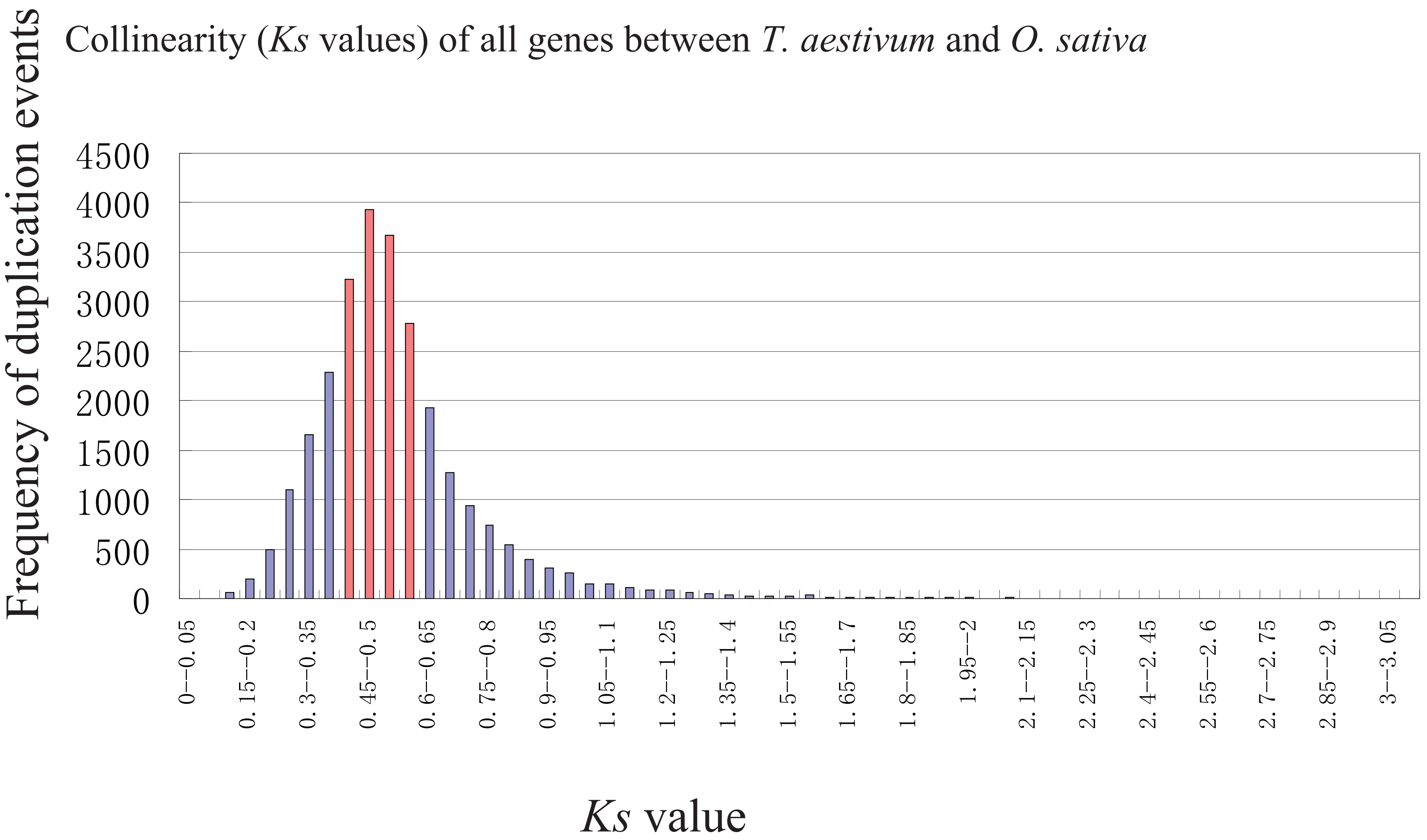

Supplement: Supplementary file 3 — Supplementary Material 3 [file 12864_2024_10383_MOESM3_ESM.pdf]
