# Supplementary material for: Genome-wide evolutionary analysis of TKL_CTR1-DRK-2 gene family and functional characterization reveals that TaCTR1 positively regulates flowering time in wheat
Source: BMC Genomics. 2024 May 14;25:474. doi: 10.1186/s12864-024-10383-2 (PMC11092142; doi:10.1186/s12864-024-10383-2)
Supplement: Supplementary file 7 — Supplementary Material 7 [file 12864_2024_10383_MOESM7_ESM.pdf]

A

Protein sequence of gene *TaCTR1* (Ensembl pep Id: TraesCS4D02G010200.1)

>TraesCS4D02G010200.1 pep chromosome:IWGSC:4D:4767194:4774274:-1 gene:TraesCS4D02G010200 transcript:TraesCS4D02G010200.1 gene_biotype:protein_coding transcript_biotype:protein_coding

MELPAAGGGRRTSYSLLSQFPDDAAAAGASPAVLQRQSSGGSSYGAGSSVSASSDYPFHL
PPAVAAAGGGGGTPSPCKSWAQQAEETYQLQLALALRLCADAACAADPGFLDPGDSGGS
K
MGGGGGGSGSGRAFPLAPPSPTAEALSHRFWVNGSLSYSNTIPDGFYLIQGMDPFVWSMC
TDVHEENRIPSVESLKSVRPDDSSIQVVLVDRRADFDLGMLENYASSFLSSSSDMKDVIN
QLAKLVSSRMGGTTSNEENLLPRWKESSEAIKSSAGSIVLHLGKLPIGLCKHRSLLFKML
ADKVNIPCRLVKGCKYCKAEDASSCVVRFGLEREYLVDLFGAPGQLSDPDSFVNGPYSLS
VPSPLRPPKFRSLEITSNFSSVAKQYFSDCHSLNLLFSDASTGASNGAAVAVDQMYSKKH
DAGDGIANSWVPVKGQAIANSDIILPEAPREVLPLMSPSNLTADKKKEFQLIEGNQYLRS
TVSDLSLAVDDLIIPWSELVLKEKIGAGSFGTVHRADWHGSDVAVKILMEQDYHLDRFKE
FMREVAIMKSLRHPNIVLFMGAVTEPPNLSIVTEYLSRGSLYKLLHRSGAREVLDERRRL
NMAFDVAKGMNYLHRRSPPIVHRDLKSPNLLVDKKYTVKVCDFGLSRLKANTYLSSKSL
A
GTPEWMAPEVLRDEPSNEKSDVYSFAVILWELMTLQQPWCNLNPAQVVAAVGFKGRRLEI
PKELNPQVAALIESCWANEPWRRPSFANIMETLRPLINKVPVPQLIRSDS

B

Coding sequence (CDS) of gene *TaCTR1* (Ensembl CDS Id: TraesCS4D02G010200.1)

>TraesCS4D02G010200.1 cds chromosome:IWGSC:4D:4767194:4774274:-1 gene:TraesCS4D02G010200 gene_biotype:protein_coding transcript_biotype:protein_coding
ATGGAGCTGCCGGCCGCCGGCGGGGGCCGGCGCACGAGCTACTCGCTGCTGTCCCAG
TTC
CCCGACGACGCGGCGGCCGCGGGGGCCTCCCCGGCGGTTCTGCAGCGGCAGTCCAGC
GGC
GGCAGCAGCTACGGCGCCGGGAGCTCCGTCTCGGCCTCCAGCGACTACCCGTTCCACC
TG
CCCCCGGCGGTCGCCGCGGCGGGAGGGGGAGGGGGCACGCCCTCCCCCTGCAAGAG
CTGG
GCGCAGCAGGCGGAGGAGACGTACCAGCTGCAGCTCGCCCTCGCGCTCCGCCTCTGC
GCC
GACGCCGCCTGCGCCGCCGACCCCGGGTTCCTCGACCCCGGCGACTCCGGGGGGTCC
AAG
ATGGGGGGCGGCGGGGGTGGCAGCGGCAGCGGCAGGGCCTTCCCGCTCGCTCCCCCC
TCC
CCCACCGCCGAGGCCCTCTCCCACCGCTTCTGGGTAAACGGCTCGTTATCATACAGCA
AC
ACGATACCGGATGGGTTCTACTTGATCCAAGGGATGGACCCCTTTGTGTGGTCGATGTG
C
ACCGACGTGCACGAGGAGAACCGCATACCCTCCGTGGAGTCGCTCAAGTCTGTCCGCC
CC
GACGATTCTTCCATCCAGGTGGTCCTCGTCGACAGGAGAGCTGATTTCGATCTTGGTAT
G
CTGGAGAACTACGCGTCCAGCTTTCTGTCCAGTTCCTCCGACATGAAAGATGTGATAAA
T
CAGCTAGCCAAACTTGTGTCTTCCAGAATGGGTGGTACAACCTCCAACGAGGAGAACT
TG
CTTCCACGCTGGAAAGAGAGCAGCGAGGCAATCAAATCAAGTGCAGGATCTATTGTGC
TT
CATCTGGGGAAGCTGCCGATTGGTCTCTGCAAGCACCGCTCACTGCTTTTTAAAATGTT
A
GCAGATAAAGTCAACATACCATGCAGATTAGTCAAGGGCTGTAAATACTGTAAAGCTG
AA
GACGCTTCCTCCTGTGTCGTACGTTTTGGGCTTGAAAGGGAATATCTGGTTGACTTATT
T
GGGGCTCCAGGCCAATTATCTGATCCTGATTCTTTCGTCAATGGTCCCTACTCGCTGTCG
GTTCCATCACCTCTCCGCCCACCAAAATTTAGGTCGTTAGAGATCACTTCAAATTTCAG
C
TCAGTTGCCAAGCAATACTTCTCAGACTGTCACTCGCTCAATCTATTGTTCAGTGATGC
T

TCCACAGGTGCTTCTAATGGTGCTGCAGTTGCTGTAGACCAAATGTATTCGAAGAAACA
T
GATGCAGGGGACGGAATTGCCAATAGCTGGGTGCCAGTGAAAGGGCAAGCAATTGCG
AAC
TCAGACATTATTCTGCCAGAAGCTCCTCGGGAAGTTTTGCCACTTATGTCACCGTCCAA
T
TTGACAGCTGATAAAAAGAAAGAGTTTCAGTTGATCGAGGGGAATCAGTATCTGCGAA
GT
ACTGTCAGTGATCTGTCACTTGCTGTGGATGATCTGATCATTCCATGGAGTGAGCTGGT
T
CTAAAGGAGAAGATTGGAGCAGGTTCTTTCGGAACAGTTCATCGTGCTGACTGGCATG
GA
TCGGATGTTGCTGTGAAGATACTGATGGAGCAGGATTATCATCTGGATCGCTTCAAGGA
A
TTTATGAGAGAGGTTGCAATAATGAAAAGTCTGAGGCATCCAAATATTGTTCTATTCATG
GGTGCTGTTACTGAACCCCCAAACCTATCAATAGTTACAGAGTACTTGTCGAGGGGTAG
T
TTGTATAAACTTTTGCATAGGAGTGGTGCAAGGGAGGTTCTGGATGAGAGACGCCGCT
TG
AACATGGCATTCGATGTGGCGAAGGGAATGAATTATCTACATAGACGTAGCCCTCCTAT
T
GTTCACCGTGATTTGAAATCTCCAAATCTTCTTGTCGACAAGAAGTATACTGTGAAGGT
A
TGCGACTTTGGGCTTTCGCGACTAAAAGCCAACACCTACCTGTCCTCGAAATCTTTGG
CA
GGAACACCTGAATGGATGGCGCCTGAAGTGCTTCGAGATGAGCCATCCAATGAGAAGT
CT
GATGTGTACAGTTTCGCTGTTATCCTGTGGGAACTTATGACATTACAACAACCATGGTG
T
AACTTGAATCCTGCCCAGGTAGTTGCTGCTGTTGGCTTTAAAGGAAGAAGACTTGAAA
TT
CCGAAAGAATTAAATCCTCAAGTAGCTGCATTAATCGAATCCTGCTGGGCAAATGAGCC
A
TGGCGGAGGCCCTCATTTGCTAATATCATGGAAACTCTGAGGCCTTTAATTAACAAGGT
C
CCGGTGCCGCAGTTGATCCGCTCAGACTCATAG

C

Chromosome information of gene *TaCTR1* (Ensembl pep Id: TraesCS4D02G010200.1)

Chromosome 4D

Location of Chromosome 4D: 4767194:4774274

Plus or minus chain:-1 stands for minus chain

D

GFF information of gene *TaCTR1* (Ensembl gene Id: TraesCS4D02G010200)

###
4D IWGSC gene 4767194 4774274 . - . ID=gene:TraesCS4D02G010200;biotype=protein_coding;gene_id=TraesCS4D02G010200;logic_name=iwgsc_high_conf

4D IWGSC mRNA 4767194 4774274 . - . ID=transcript:TraesCS4D02G010200.1;Parent=gene:TraesCS4D02G010200;biotype=protein_coding;tag=Ensembl_canonical;transcript_id=TraesCS4D02G010200.1

4D IWGSC exon 4767194 4767528 . - . Parent=transcript:TraesCS4D02G010200.1;Name=TraesCS4D02G010200.1-E17;constitutive=1;ensembl_end_phase=-1;ensembl_phase=-1;exon_id=TraesCS4D02G010200.1-E17;rank=17

4D IWGSC three_prime_UTR 4767194 4767528 . - . Parent=transcript:TraesCS4D02G010200.1

4D IWGSC three_prime_UTR 4767943 4768153 . - . Parent=transcript:TraesCS4D02G010200.1

4D IWGSC exon 4767943 4768253 . - . Parent=transcript:TraesCS4D02G010200.1;Name=TraesCS4D02G010200.1-E16;constitutive=1;ensembl_end_phase=-1;ensembl_phase=2;exon_id=TraesCS4D02G010200.1-E16;rank=16

4D IWGSC CDS 4768154 4768253 . - 1 ID=CDS:TraesCS4D02G010200.1;Parent=transcript:TraesCS4D02G010200.1;protein_id=TraesCS4D02G010200.1

4D IWGSC exon 4768344 4768438 . - . Parent=transcript:TraesCS4D02G010200.1;Name=TraesCS4D02G010200.1-E15;constitutive=1;ensembl_end_phase=2;ensembl_phase=0;exon_id=TraesCS4D02G010200.1-E15;rank=15

4D IWGSC CDS 4768344 4768438 . - 0 ID=CDS:TraesCS4D02G010200.1;Parent=transcript:TraesCS4D02G010200.1;protein_id=TraesCS4D02G010200.1

4D IWGSC exon 4768592 4768723 . - . Parent=transcript:TraesCS4D02G010200.1;Name=TraesCS4D02G010200.1-E14;constitutive=1;ensembl_end_phase=0;ensembl_phase=0;exon_id=TraesCS4D02G010200.1-E14;rank=14

4D IWGSC CDS 4768592 4768723 . - 0 ID=CDS:TraesCS4D02G010200.1;Parent=transcript:TraesCS4D02G010200.1;protein_id=TraesCS4D02G010200.1

4D IWGSC exon 4768801 4768869 . - . Parent=transcript:TraesCS4D02G010200.1;Name=TraesCS4D02G010200.1-E13;constitutive=1;ensembl_end_phase=0;ensembl_phase=0;exon_id=TraesCS4D02G010200.1-E13;rank=13

4D IWGSC CDS 4768801 4768869 . - 0 ID=CDS:TraesCS4D02G010200.1;Parent=transcript:TraesCS4D02G010200.1;protein_id=TraesCS4D02G010200.1

4D IWGSC exon 4769620 4769718 . - . Parent=transcript:TraesCS4D02G010200.1;Name=TraesCS4D02G010200.1-E12;constitutive

=1;ensembl_end_phase=0;ensembl_phase=0;exon_id=TraesCS4D02G010200.1-E12;rank=12
4D IWGSC CDS4769620 4769718 . - 0
ID=CDS:TraesCS4D02G010200.1;Parent=transcript:TraesCS4D02G010200.1;protein_id=TraesCS4D02G010200.1
4D IWGSC exon4769826 4769910 . - .
Parent=transcript:TraesCS4D02G010200.1;Name=TraesCS4D02G010200.1-E11;constitutive=1;ensembl_end_phase=0;ensembl_phase=2;exon_id=TraesCS4D02G010200.1-E11;rank=11
4D IWGSC CDS4769826 4769910 . - 1
ID=CDS:TraesCS4D02G010200.1;Parent=transcript:TraesCS4D02G010200.1;protein_id=TraesCS4D02G010200.1
4D IWGSC exon4770002 4770102 . - .
Parent=transcript:TraesCS4D02G010200.1;Name=TraesCS4D02G010200.1-E10;constitutive=1;ensembl_end_phase=2;ensembl_phase=0;exon_id=TraesCS4D02G010200.1-E10;rank=10
4D IWGSC CDS4770002 4770102 . - 0
ID=CDS:TraesCS4D02G010200.1;Parent=transcript:TraesCS4D02G010200.1;protein_id=TraesCS4D02G010200.1
4D IWGSC exon4770419 4770487 . - .
Parent=transcript:TraesCS4D02G010200.1;Name=TraesCS4D02G010200.1-E9;constitutive=1;ensembl_end_phase=0;ensembl_phase=0;exon_id=TraesCS4D02G010200.1-E9;rank=9
4D IWGSC CDS4770419 4770487 . - 0
ID=CDS:TraesCS4D02G010200.1;Parent=transcript:TraesCS4D02G010200.1;protein_id=TraesCS4D02G010200.1
4D IWGSC exon4770687 4770727 . - .
Parent=transcript:TraesCS4D02G010200.1;Name=TraesCS4D02G010200.1-E8;constitutive=1;ensembl_end_phase=0;ensembl_phase=1;exon_id=TraesCS4D02G010200.1-E8;rank=8
4D IWGSC CDS4770687 4770727 . - 2
ID=CDS:TraesCS4D02G010200.1;Parent=transcript:TraesCS4D02G010200.1;protein_id=TraesCS4D02G010200.1
4D IWGSC exon4770972 4771190 . - .
Parent=transcript:TraesCS4D02G010200.1;Name=TraesCS4D02G010200.1-E7;constitutive=1;ensembl_end_phase=1;ensembl_phase=1;exon_id=TraesCS4D02G010200.1-E7;rank=7
4D IWGSC CDS4770972 4771190 . - 2
ID=CDS:TraesCS4D02G010200.1;Parent=transcript:TraesCS4D02G010200.1;protein_id=TraesCS4D02G010200.1
4D IWGSC exon4771441 4771536 . - .
Parent=transcript:TraesCS4D02G010200.1;Name=TraesCS4D02G010200.1-E6;constitutive=1;ensembl_end_phase=1;ensembl_phase=1;exon_id=TraesCS4D02G010200.1-E6;rank=6
4D IWGSC CDS4771441 4771536 . - 2
ID=CDS:TraesCS4D02G010200.1;Parent=transcript:TraesCS4D02G010200.1;protein_id=TraesCS4D02G010200.1
4D IWGSC exon4771711 4771919 . - .
Parent=transcript:TraesCS4D02G010200.1;Name=TraesCS4D02G010200.1-E5;constitutive=1;ensembl_end_phase=1;ensembl_phase=2;exon_id=TraesCS4D02G010200.1-E5;rank=5
4D IWGSC CDS4771711 4771919 . - 1

ID=CDS:TraesCS4D02G010200.1;Parent=transcript:TraesCS4D02G010200.1;protein_id=TraesCS4D02G010200.1
4D IWGSC exon4772253 4772356 . - .
Parent=transcript:TraesCS4D02G010200.1;Name=TraesCS4D02G010200.1-E4;constitutive=1;ensembl_end_phase=2;ensembl_phase=0;exon_id=TraesCS4D02G010200.1-E4;rank=4
4D IWGSC CDS4772253 4772356 . - 0
ID=CDS:TraesCS4D02G010200.1;Parent=transcript:TraesCS4D02G010200.1;protein_id=TraesCS4D02G010200.1
4D IWGSC exon4772660 4772801 . - .
Parent=transcript:TraesCS4D02G010200.1;Name=TraesCS4D02G010200.1-E3;constitutive=1;ensembl_end_phase=0;ensembl_phase=2;exon_id=TraesCS4D02G010200.1-E3;rank=3
4D IWGSC CDS4772660 4772801 . - 1
ID=CDS:TraesCS4D02G010200.1;Parent=transcript:TraesCS4D02G010200.1;protein_id=TraesCS4D02G010200.1
4D IWGSC exon4772888 4773186 . - .
Parent=transcript:TraesCS4D02G010200.1;Name=TraesCS4D02G010200.1-E2;constitutive=1;ensembl_end_phase=2;ensembl_phase=0;exon_id=TraesCS4D02G010200.1-E2;rank=2
4D IWGSC CDS4772888 4773186 . - 0
ID=CDS:TraesCS4D02G010200.1;Parent=transcript:TraesCS4D02G010200.1;protein_id=TraesCS4D02G010200.1
4D IWGSC CDS4773585 4774037 . - 0
ID=CDS:TraesCS4D02G010200.1;Parent=transcript:TraesCS4D02G010200.1;protein_id=TraesCS4D02G010200.1
4D IWGSC exon4773585 4774274 . - .
Parent=transcript:TraesCS4D02G010200.1;Name=TraesCS4D02G010200.1-E1;constitutive=1;ensembl_end_phase=0;ensembl_phase=-1;exon_id=TraesCS4D02G010200.1-E1;rank=1
4D IWGSC five_prime_UTR 4774038 4774274 . - .
Parent=transcript:TraesCS4D02G010200.1
###

E

*T.*ae TKL_CTR1−DRK−2 II subfamily exon−intron and kinase domain diagram (including *TaCTR1*)

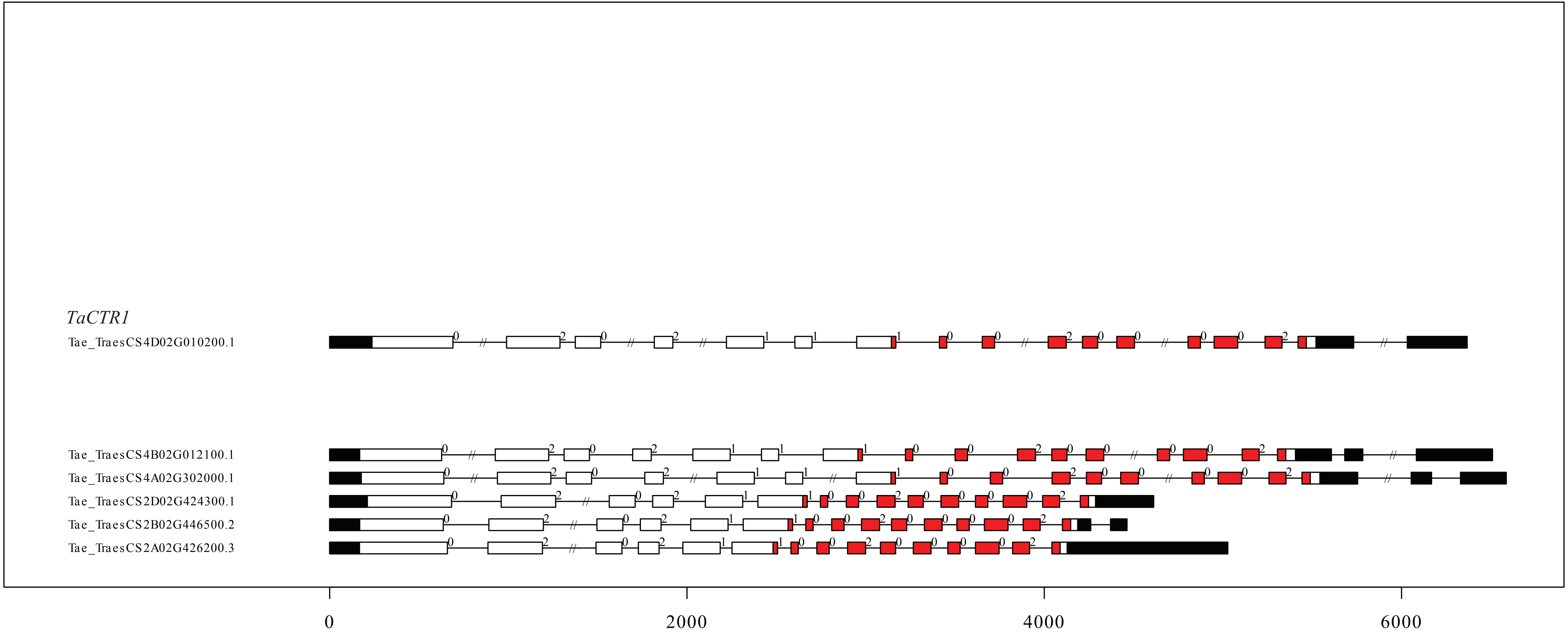

F BLAST result of gene *TaCTR1* (Ensembl pep Id: TraesCS4D02G010200.1)

Program: BLASTP
Database: nr All non-redundant GenBank CDS translations+PDB+SwissProt+PIR+PRF excluding environmental samples from WGS projects
Query #1: TraesCS4D02G010200.1 pep chromosome:IWGSC:4D:4767194:4774274:-1 gene:TraesCS4D02G010200 transcript:TraesCS4D02G010200.1 gene_biotype:protein_coding transcript_biotype:protein_coding Query ID: lcl|Query_3972404 Length: 770

Sequences producing significant alignments:

Description	Scientific Name	Common Name	Taxid	Max Score	Total Score	Query cover	E Value	Per. Ident	Acc. Len	Accession
serine/threonine-protein kinase CTR1 [Aegilops tauschii subsp....	Aegilops tau...	NA	200361	1578	1578	100%	0.0	100.00	770	XP_020183981.1
serine/threonine-protein kinase CTR1-like [Triticum urartu]	Triticum urartu	NA	4572	1566	1566	100%	0.0	99.35	773	XP_048551448.1
serine/threonine-protein kinase CTR1-like [Triticum dicoccoides]	Triticum dic...	NA	85692	1563	1563	100%	0.0	98.96	772	XP_037428624.1
uncharacterized protein LOC123095596 [Triticum aestivum]	Triticum aes...	bread wheat	4565	1560	1560	98%	0.0	99.61	1684	XP_044373050.1
hypothetical protein CFC21_053784 [Triticum aestivum]	Triticum aes...	bread wheat	4565	1540	1540	100%	0.0	98.06	763	KAF7044576.1
Serine/threonine-protein kinase CTR1 [Hordeum vulgare]	Hordeum vulgare	NA	4513	1536	1536	100%	0.0	97.42	773	KAE8817350.1
hypothetical protein ZWY2020_054149 [Hordeum vulgare]	Hordeum vulgare	NA	4513	1536	1536	100%	0.0	97.55	814	KAI4998807.1
unnamed protein product [Triticum turgidum subsp. durum]	Triticum tur...	durum wheat	4567	1495	1495	95%	0.0	99.32	755	VAH96052.1
unnamed protein product [Triticum turgidum subsp. durum]	Triticum tur...	durum wheat	4567	1467	1467	100%	0.0	93.77	728	VAI00708.1
unnamed protein product [Triticum turgidum subsp. durum]	Triticum tur...	durum wheat	4567	1467	1467	100%	0.0	93.77	726	VAI00706.1
unnamed protein product [Triticum turgidum subsp. durum]	Triticum tur...	durum wheat	4567	1466	1466	100%	0.0	93.90	729	VAH96051.1
unnamed protein product [Triticum turgidum subsp. durum]	Triticum tur...	durum wheat	4567	1435	1435	93%	0.0	97.10	725	VAI00710.1
uncharacterized protein LOC127304444 [Lolium perenne]	Lolium perenne	NA	4522	1354	1354	100%	0.0	92.39	1821	XP_051191091.1
uncharacterized protein LOC124657634 [Lolium rigidum]	Lolium rigidum	NA	89674	1350	1350	100%	0.0	92.12	1797	XP_047052109.1
serine/threonine-protein kinase CTR1-like [Lolium rigidum]	Lolium rigidum	NA	89674	1345	1345	100%	0.0	91.57	763	XP_047052104.1

hypothetical protein QYE76_006900 [Lolium multiflorum] Lolium multi... Italian ryeg... 4521 1342 1342 100% 0.0 92.13 768 KAK1632585.1
uncharacterized protein LOC100825661 [Brachypodium distachyon] Brachypodium... stiff brome 15368 1328 1328 100% 0.0 90.85 1820 XP_014757903.1
hypothetical protein BRADI_4g38400v3 [Brachypodium distachyon] Brachypodium... stiff brome 15368 1321 1321 100% 0.0 90.85 767 KQJ91573.1
hypothetical protein E2562_011176 [Oryza meyeriana var.... Oryza meyeri... NA 110450 1283 1283 100% 0.0 85.84 752 KAF0911546.1
serine/threonine-protein kinase CTR1-like [Oryza brachyantha] Oryza brachy... malo sina 4533 1260 1260 100% 0.0 84.16 739 XP_040383430.1
hypothetical protein ZWY2020_054148 [Hordeum vulgare] Hordeum vulgare NA 4513 1249 1456 81% 0.0 96.18 999 KAI4998806.1
serine/threonine-protein kinase CTR1-like isoform X2 [Oryza... Oryza glaber... African rice 4538 1234 1234 100% 0.0 84.18 751 XP_052166726.1
serine/threonine-protein kinase CTR1 [Oryza sativa Japonica... Oryza sativa... Japanese rice 39947 1231 1231 100% 0.0 83.79 751 XP_015611604.1
serine/threonine-protein kinase CTR1-like isoform X1 [Oryza... Oryza glaber... African rice 4538 1226 1226 100% 0.0 83.08 760 XP_052166725.1
hypothetical protein EJB05_44104 [Eragrostis curvula] Eragrostis c... weeping love... 38414 1224 1224 100% 0.0 81.71 762 TVU10563.1
hypothetical protein EJB05_44145 [Eragrostis curvula] Eragrostis c... weeping love... 38414 1223 1223 100% 0.0 81.67 762 TVU10602.1
putative serine/threonine-specific protein kinase [Oryza sativ... Oryza sativa... Japanese rice 39947 1221 1221 100% 0.0 82.69 760 BAD46244.1
hypothetical protein GUJ93_ZPchr0002g23815 [Zizania palustris] Zizania palu... NA 103762 1216 1216 98% 0.0 82.30 740 KAG8057783.1
hypothetical protein QOZ80_6AG0525500 [Eleusine coracana subsp... Eleusine cor... NA 191504 1212 1212 100% 0.0 81.39 762 KAK3132638.1
hypothetical protein U9M48_012797 [Paspalum notatum var. saurae] Paspalum not... NA 547442 1209 1209 99% 0.0 82.79 770 WVZ63138.1
serine/threonine-protein kinase CTR1 isoform X1 [Phragmites... Phragmites a... common reed 29695 1204 1204 100% 0.0 82.45 764 XP_062194396.1
Serine/threonine-protein kinase CTR1 [Dichanthelium oligosanthes] Dichantheliu... NA 888268 1201 1201 100% 0.0 83.21 769 OEL25444.1
hypothetical protein HU200_039538 [Digitaria exilis] Digitaria ex... NA 1010633 1199 1199 100% 0.0 84.44 762 KAF8692704.1
hypothetical protein GUJ93_ZPchr0009g1750 [Zizania palustris] Zizania palu... NA 103762 1196 1196 100% 0.0 81.63 752 KAG8050273.1
uncharacterized protein LOC101754395 [Setaria italica] Setaria italica foxtail millet 4555 1190 1190 100% 0.0 82.18 1851 XP_012699069.1
uncharacterized protein LOC117844130 [Setaria viridis] Setaria viridis NA 4556 1189 1189 100% 0.0 82.18 1851 XP_034580787.1
hypothetical protein SETIT_2G132000v2 [Setaria italica] Setaria italica foxtail millet 4555 1184 1184 100% 0.0 82.56 770 RCV10728.1

uncharacterized protein LOC120662481 [Panicum virgatum] Panicum virg... switchgrass 38727 1180 1180 100% 0.0 82.63 1896 XP_039797554.1
serine/threonine-protein kinase CTR1 [Sorghum bicolor] Sorghum bicolor sorghum 4558 1179 1179 100% 0.0 81.39 764 XP_002461985.1
hypothetical protein GQ55_2G122400 [Panicum hallii var. hallii] Panicum hall... NA 1504633 1179 1179 100% 0.0 83.18 769 PUZ69599.1
uncharacterized protein LOC112880881 [Panicum hallii] Panicum hallii NA 206008 1179 1179 100% 0.0 82.67 1965 XP_025801398.1
hypothetical protein PVAP13_2NG262500 [Panicum virgatum] Panicum virg... switchgrass 38727 1173 1173 100% 0.0 82.78 768 KAG2633570.1
hypothetical protein PAHAL_2G129500 [Panicum hallii] Panicum hallii NA 206008 1172 1172 100% 0.0 83.06 769 PAN10967.1
uncharacterized protein LOC120695190 [Panicum virgatum] Panicum virg... switchgrass 38727 1166 1166 99% 0.0 81.67 1912 XP_039834424.1
hypothetical protein PVAP13_2KG218100 [Panicum virgatum] Panicum virg... switchgrass 38727 1164 1164 99% 0.0 81.79 771 KAG2641912.1
serine/threonine-protein kinase CTR1 isoform X2 [Phragmites... Phragmites a... common reed 29695 1161 1161 100% 0.0 80.65 738 XP_062194397.1
hypothetical protein GUJ93_ZPchr0009g1750 [Zizania palustris] Zizania palu... NA 103762 1158 1158 100% 0.0 75.60 811 KAG8050274.1
serine/threonine-protein kinase CTR1 [Zea mays] Zea mays NA 4577 1156 1156 100% 0.0 80.82 769 XP_033915561.1
hypothetical protein BS78_02G130700 [Paspalum vaginatum] Paspalum vag... NA 158149 1149 1149 99% 0.0 80.34 737 KAJ1288982.1
Serine/threonine-protein kinase CTR1 [Triticum urartu] Triticum urartu NA 4572 1127 1127 76% 0.0 95.22 659 EMS46799.1
unnamed protein product [Miscanthus lutarioriparius] Miscanthus l... NA 422564 1113 1113 96% 0.0 80.37 758 CAD6220040.1
uncharacterized protein C2845_PM03G27530 [Panicum miliaceum] Panicum mili... NA 4540 1102 1102 95% 0.0 82.39 758 RLN35053.1
unnamed protein product [Miscanthus lutarioriparius] Miscanthus l... NA 422564 1100 1100 95% 0.0 80.61 760 CAD6225707.1
unnamed protein product [Triticum turgidum subsp. durum] Triticum tur... durum wheat 4567 1090 1090 73% 0.0 94.53 546 VAI00709.1
hypothetical protein PVAP13_2KG218100 [Panicum virgatum] Panicum virg... switchgrass 38727 1053 1053 92% 0.0 80.72 727 KAG2641915.1
hypothetical protein PVAP13_2KG218100 [Panicum virgatum] Panicum virg... switchgrass 38727 1050 1050 91% 0.0 80.92 749 KAG2641916.1

hypothetical protein OsJ_30383 [Oryza sativa Japonica Group] Oryza sativa... Japanese rice 39947 1048 1048 73% 0.0 87.50 710 EAZ45706.1
hypothetical protein SEVIR_2G137201v2 [Setaria viridis] Setaria viridis NA 4556 973 973 71% 0.0 87.66 550 TKW31910.1
serine/threonine-protein kinase CTR1 isoform X1 [Iris pallida] Iris pallida NA 29817 956 956 99% 0.0 63.79 744 KAJ6802525.1
PREDICTED: serine/threonine-protein kinase CTR1-like [Musa... Musa acumina... wild Malaysi... 214687 929 929 97% 0.0 63.11 799 XP_009383670.1
serine threonine-protein kinase [Musa troglodytarum] Musa troglod... NA 320322 921 921 97% 0.0 61.85 803 URD83379.1
hypothetical protein OPV22_000954 [Ensete ventricosum] Ensete ventr... NA 4639 919 919 97% 0.0 62.09 804 KAJ8510520.1
unnamed protein product [Musa acuminata subsp. malaccensis] Musa acumina... wild Malaysi... 214687 916 916 97% 0.0 62.39 805 CAG1864453.1
constitutive triple response 1-like protein [Musa acuminata AA... Musa acumina... dessert banana 214697 914 914 97% 0.0 62.27 805 AFA37962.1
PREDICTED: serine/threonine-protein kinase CTR1-like [Musa... Musa acumina... wild Malaysi... 214687 914 914 97% 0.0 62.27 805 XP_018676639.1
serine/threonine-protein kinase CTR1-like [Zingiber officinale] Zingiber off... NA 94328 911 911 99% 0.0 59.69 811 XP_042457633.1
serine/threonine-protein kinase CTR1-like [Zingiber officinale] Zingiber off... NA 94328 909 909 99% 0.0 59.69 811 XP_042462984.1
hypothetical protein OPV22_029832 [Ensete ventricosum] Ensete ventr... NA 4639 900 900 98% 0.0 60.51 795 KAJ8467280.1
serine/threonine-protein kinase CTR1 [Canna indica] Canna indica NA 4628 899 899 88% 0.0 65.85 797 WOL03471.1
serine/threonine-protein kinase CTR1 [Canna indica] Canna indica NA 4628 897 897 97% 0.0 59.28 826 WOL15672.1
serine/threonine-protein kinase CTR1 isoform X1 [Ananas comosus] Ananas comosus pineapple 4615 893 893 88% 0.0 63.19 821 XP_020111529.1
serine/threonine-protein kinase CTR1 isoform X1 [Magnolia sinica] Magnolia sinica NA 86752 879 879 89% 0.0 61.23 864 XP_058085947.1
serine/threonine-protein kinase CTR1 isoform X2 [Ananas comosus] Ananas comosus pineapple 4615 874 874 88% 0.0 62.48 811 XP_020111530.1
serine/threonine-protein kinase CTR1-like [Tripterygium... Tripterygium... NA 458696 870 870 92% 0.0 60.88 866 XP_038702140.1
hypothetical protein LUZ63_009036 [Rhynchospora breviuscula] Rhynchospora... NA 2022672 870 870 98% 0.0 61.33 742 KAJ1692338.1
hypothetical protein LUZ61_020752 [Rhynchospora tenuis] Rhynchospora... NA 198213 870 870 91% 0.0 62.96 751 KAJ3691588.1
protein kinase family protein [Rhynchospora pubera] Rhynchospora... NA 906938 869 869 98% 0.0 60.86 753 KAJ4783134.1

protein kinase family protein [Rhynchospora pubera] Rhynchospora... NA
906938 868 868 98% 0.0 60.86 749 KAJ4802344.1
Serine/threonine-protein kinase CTR1 [Cajanus cajan] Cajanus cajan pigeon pea 3821 868 868 99% 0.0 58.10 775 KYP73796.1
unnamed protein product [Vitis vinifera] Vitis vinifera wine grape 29760 867 867 98% 0.0 59.71 745 CBI30245.3
protein kinase family protein [Rhynchospora pubera] Rhynchospora... NA
906938 866 866 98% 0.0 61.41 806 KAJ4775647.1
protein kinase family protein [Rhynchospora pubera] Rhynchospora... NA
906938 865 865 98% 0.0 61.04 824 KAJ4776217.1
serine/threonine-protein kinase CTR1-like isoform X2 [Panicum... Panicum hallii NA
206008 864 864 95% 0.0 59.92 801 XP_025799085.1
serine/threonine-protein kinase CTR1-like [Ipomoea triloba] Ipomoea triloba trilobed mor... 35885 863 863 89% 0.0 60.41 850 XP_031131407.1
serine/threonine-protein kinase CTR1-like isoform X2 [Pistacia... Pistacia vera NA
55513 863 863 98% 0.0 55.26 832 XP_031261290.1
hypothetical protein ZIOFF_014783 [Zingiber officinale] Zingiber off... NA
94328 862 862 95% 0.0 58.82 802 KAG6524839.1
serine/threonine-protein kinase CTR1-like isoform X1 [Ipomoea... Ipomoea batatas sweet potato
4120 860 860 89% 0.0 60.86 839 GMC49546.1
hypothetical protein QOZ80_2BG0188030 [Eleusine coracana subsp... Eleusine cor... NA
191504 857 857 95% 0.0 60.48 777 KAK3154238.1
hypothetical protein KFK09_014274 [Dendrobium nobile] Dendrobium n... NA
94219 857 857 91% 0.0 59.68 822 KAI0508140.1
serine/threonine-protein kinase CTR1-like [Papaver somniferum] Papaver somn... opium poppy 3469 856 856 100% 0.0 55.52 812 XP_026425732.1
hypothetical protein BS78_04G149400 [Paspalum vaginatum] Paspalum vag... NA
158149 855 855 90% 0.0 62.02 797 KAJ1279357.1
serine/threonine-protein kinase CTR1 [Populus trichocarpa] Populus tric... black cotton...
3694 854 854 91% 0.0 63.84 821 XP_006381314.2
PREDICTED: serine/threonine-protein kinase CTR1 isoform X2... Populus euph... Euphrates po... 75702 854 854 93% 0.0 61.94 818
XP_011018886.1
hypothetical protein PHAVU_007G223500g [Phaseolus vulgaris] Phaseolus vu... NA
3885 854 854 99% 0.0 56.21 836 XP_007145256.1
Serine/threonine-protein kinase [Actinidia chinensis var.... Actinidia ch... NA
1590841 854 854 99% 0.0 55.92 824 PSS34902.1
serine/threonine-protein kinase CTR1-like isoform X1 [Lolium... Lolium perenne NA
4522 854 854 98% 0.0 56.91 773 XP_051217156.1
serine/threonine-protein kinase CTR1 [Citrus sinensis] Citrus sinensis sweet orange
2711 854 854 89% 0.0 61.70 838 KAH9738416.1
serine/threonine-protein kinase CTR1 isoform X3 [Ziziphus jujuba] Ziziphus jujuba common jujube 326968 853 853 99% 0.0 56.50 818 XP_048321301.1
protein kinase superfamily protein [Actinidia rufa] Actinidia rufa NA

165716 853 853 99% 0.0 55.92 824 GFY82708.1
serine/threonine-protein kinase CTR1 isoform X2 [Elaeis... Elaeis guine... African oil ...
51953 852 852 98% 0.0 58.45 754 XP_019703234.1

Alignments:

>serine/threonine-protein kinase CTR1 [Aegilops tauschii subsp. strangulata]
Sequence ID: XP_020183981.1 Length: 770
>hypothetical protein CFC21_060075 [Triticum aestivum]
Sequence ID: KAF7051885.1 Length: 770
>hypothetical protein CFC21_060076 [Triticum aestivum]
Sequence ID: KAF7051886.1 Length: 770
Range 1: 1 to 770

Score:1578 bits(4087), Expect:0.0,
Method:Compositional matrix adjust.,
Identities:770/770(100%), Positives:770/770(100%), Gaps:0/770(0%)

Query 1
MELPAAGGGRRTSYSLLSQFPDDAAAAGASPAVLQRQSSGGSSYGAGSSVSASSDYPFHL
60

MELPAAGGGRRTSYSLLSQFPDDAAAAGASPAVLQRQSSGGSSYGAGSSVSASSDYPFHL
Sbjct 1
MELPAAGGGRRTSYSLLSQFPDDAAAAGASPAVLQRQSSGGSSYGAGSSVSASSDYPFHL
60

Query 61
PPAVAAAGGGGGTPSPCKSWAQQAEETYQLQLALALRLCADAACAADPGFLDPGDSGGS
K 120

PPAVAAAGGGGGTPSPCKSWAQQAEETYQLQLALALRLCADAACAADPGFLDPGDSGGS
K
Sbjct 61
PPAVAAAGGGGGTPSPCKSWAQQAEETYQLQLALALRLCADAACAADPGFLDPGDSGGS
K 120

Query 121
MGGGGGGSGSGRAFPLAPPSPTAEALSHRFWVNGSLSYSNTIPDGFYLIQGMDPFVWSMC
180

MGGGGGGSGSGRAFPLAPPSPTAEALSHRFWVNGSLSYSNTIPDGFYLIQGMDPFVWSMC
Sbjct 121

MGGGGGGSGSGRAFPLAPPSPTAEALSHRFWVNGSLSYSNTIPDGFYLIQGMDPFVWSMC
180

Query 181
TDVHEENRIPSVESLKSVRPDDSSIQVVLVDRRADFDLGMLENYASSFLSSSSDMKDVIN
240

TDVHEENRIPSVESLKSVRPDDSSIQVVLVDRRADFDLGMLENYASSFLSSSSDMKDVIN
Sbjct 181
TDVHEENRIPSVESLKSVRPDDSSIQVVLVDRRADFDLGMLENYASSFLSSSSDMKDVIN
240

Query 241
QLAKLVSSRMGGTTSNEENLLPRWKESSEAIKSSAGSIVLHLGKLPIGLCKHRSLLFKML
300

QLAKLVSSRMGGTTSNEENLLPRWKESSEAIKSSAGSIVLHLGKLPIGLCKHRSLLFKML
Sbjct 241
QLAKLVSSRMGGTTSNEENLLPRWKESSEAIKSSAGSIVLHLGKLPIGLCKHRSLLFKML
300

Query 301
ADKVNIPCRLVKGCKYCKAEDASSCVVRFGLEREYLVDLFGAPGQLSDPDSFVNGPYSLS
360

ADKVNIPCRLVKGCKYCKAEDASSCVVRFGLEREYLVDLFGAPGQLSDPDSFVNGPYSLS
Sbjct 301
ADKVNIPCRLVKGCKYCKAEDASSCVVRFGLEREYLVDLFGAPGQLSDPDSFVNGPYSLS
360

Query 361
VPSPLRPPKFRSLEITSNFSSVAKQYFSDCHSLNLLFSDASTGASNGAAVAVDQMYSKKH
420

VPSPLRPPKFRSLEITSNFSSVAKQYFSDCHSLNLLFSDASTGASNGAAVAVDQMYSKKH
Sbjct 361
VPSPLRPPKFRSLEITSNFSSVAKQYFSDCHSLNLLFSDASTGASNGAAVAVDQMYSKKH
420

Query 421
DAGDGIANSWVPVKGQAIANSDIILPEAPREVLPLMSPSNLTADKKKEFQLIEGNQYLRS
480

DAGDGIANSWVPVKGQAIANSDIILPEAPREVLPLMSPSNLTADKKKEFQLIEGNQYLRS

Sbjct 421
DAGDGIANSWVPVKGQAIANSDIILPEAPREVLPLMSPSNLTADKKKEFQLIEGNQYLRS
480

Query 481
TVSDLSLAVDDLIIPWSELVLKEKIGAGSFGTVHRADWHGSDVAVKILMEQDYHLDRFKE
540

TVSDLSLAVDDLIIPWSELVLKEKIGAGSFGTVHRADWHGSDVAVKILMEQDYHLDRFKE
Sbjct 481
TVSDLSLAVDDLIIPWSELVLKEKIGAGSFGTVHRADWHGSDVAVKILMEQDYHLDRFKE
540

Query 541
FMREVAIMKSLRHPNIVLFMGAVTEPPNLSIVTEYLSRGSLYKLLHRSGAREVLDERRRL
600

FMREVAIMKSLRHPNIVLFMGAVTEPPNLSIVTEYLSRGSLYKLLHRSGAREVLDERRRL
Sbjct 541
FMREVAIMKSLRHPNIVLFMGAVTEPPNLSIVTEYLSRGSLYKLLHRSGAREVLDERRRL
600

Query 601
NMAFDVAKGMNYLHRRSPPIVHRDLKSPNLLVDKKYTVKVCDFGLSRLKANTYLSSKSL
A 660

NMAFDVAKGMNYLHRRSPPIVHRDLKSPNLLVDKKYTVKVCDFGLSRLKANTYLSSKSL
A
Sbjct 601
NMAFDVAKGMNYLHRRSPPIVHRDLKSPNLLVDKKYTVKVCDFGLSRLKANTYLSSKSL
A 660

Query 661
GTPEWMAPEVLRDEPSNEKSDVYSFAVILWELMTLQQPWCNLNPAQVVAAVGFKGRRLEI
720

GTPEWMAPEVLRDEPSNEKSDVYSFAVILWELMTLQQPWCNLNPAQVVAAVGFKGRRLEI
Sbjct 661
GTPEWMAPEVLRDEPSNEKSDVYSFAVILWELMTLQQPWCNLNPAQVVAAVGFKGRRLEI
720

Query 721 PKELNPQVAALIESCWANEPWRRPSFANIMETLRPLINKVPVPQLIRSDS
770

PKELNPQVAALIESCWANEPWRRPSFANIMETLRPLINKVPVPQLIRSDS

Sbjct 721 PKELNPQVAALIESCWANEPWRRPSFANIMETLRPLINKVPVPQLIRSDS 770

>serine/threonine-protein kinase CTR1-like [Triticum urartu]
Sequence ID: XP_048551448.1 Length: 773
>unnamed protein product [Triticum turgidum subsp. durum]
Sequence ID: VAH96050.1 Length: 773
Range 1: 1 to 773

Score:1566 bits(4055), Expect:0.0,
Method:Compositional matrix adjust.,
Identities:768/773(99%), Positives:769/773(99%), Gaps:3/773(0%)

Query 1
MELPAAGGGRRTSYSLLSQFPDDAAAAGASPAVLQRQSSGGSSYGAGSSVSASSDYPFHL
60

MELPAAGGGRRTSYSLLSQFPDDAAAAGASPAVLQRQSSGGSSYGAGSSVSASSDYPFHL
Sbjct 1
MELPAAGGGRRTSYSLLSQFPDDAAAAGASPAVLQRQSSGGSSYGAGSSVSASSDYPFHL
60

Query 61
PPAVAAAGGGGG--TPSPCKSWAQQAEETYQLQLALALRLCADAACAADPGFLDPGDSGG
118
PPA AAAGGGGG
TPSPCKSWAQQAEETYQLQLALALRLCADAACAADPGFLDPGDSGG
Sbjct 61
PPAAAAAGGGGGGGTPSPCKSWAQQAEETYQLQLALALRLCADAACAADPGFLDPGDS
GG 120

Query 119
SKMGGGGGG-SGSGRAFPLAPPSPTAEALSHRFWVNGSLSYSNTIPDGFYLIQGMDPFVW
177
SKMGGGGGG
SGSGRAFPLAPPSPTAEALSHRFWVNGSLSYSNTIPDGFYLIQGMDPFVW
Sbjct 121
SKMGGGGGGGSGSGRAFPLAPPSPTAEALSHRFWVNGSLSYSNTIPDGFYLIQGMDPFVW
180

Query 178
SMCTDVHEENRIPSVESLKSVRPDDSSIQVVLVDRRADFDLGMLENYASSFLSSSSDMKD

237

SMCTDVHEENRIPSVESLKSVRPDDSSIQVVLVDRRADFDLGMLENYASSFLSSSSDMKD
Sbjct 181
SMCTDVHEENRIPSVESLKSVRPDDSSIQVVLVDRRADFDLGMLENYASSFLSSSSDMKD
240

Query 238
VINQLAKLVSSRMGGTTSNEENLLPRWKESSEAIKSSAGSIVLHLGKLPIGLCKHRSLLF
297

VINQLAKLVSSRMGGTTSNEENLLPRWKESSEAIKSSAGSIVLHLGKLPIGLCKHRSLLF
Sbjct 241
VINQLAKLVSSRMGGTTSNEENLLPRWKESSEAIKSSAGSIVLHLGKLPIGLCKHRSLLF
300

Query 298
KMLADKVNIPCRLVKGCKYCKAEDASSCVVRFGLEREYLVDLFGAPGQLSDPDSFVNGP
Y 357

KMLADKVNIPCRLVKGCKYCKAEDASSCVVRFGLEREYLVDLFGAPGQLSDPDSFVNGP
Y
Sbjct 301
KMLADKVNIPCRLVKGCKYCKAEDASSCVVRFGLEREYLVDLFGAPGQLSDPDSFVNGP
Y 360

Query 358
SLSVPSPLRPPKFRSLEITSNFSSVAKQYFSDCHSLNLLFSDASTGASNGAAVAVDQMYS
417

SLSVPSPLRPPKFRSLEITSNFSSVAKQYFSDCHSLNLLFSDASTGASNGAAVAVDQMYS
Sbjct 361
SLSVPSPLRPPKFRSLEITSNFSSVAKQYFSDCHSLNLLFSDASTGASNGAAVAVDQMYS
420

Query 418
KKHDAGDGIANSWVPVKGQAIANSDIILPEAPREVLPLMSPSNLTADKKKEFQLIEGNQY
477

KKHDAGDGIANSWVPVKGQAIANSDIILPEAPREVLPLM+PSNLTADKKKEFQLIEGNQY
Sbjct 421
KKHDAGDGIANSWVPVKGQAIANSDIILPEAPREVLPLMTPSNLTADKKKEFQLIEGNQY
480

Query 478
LRSTVSDLSLAVDDLIIPWSELVLKEKIGAGSFGTVHRADWHGSDVAVKILMEQDYHLDR
537

LRSTVSDLSLAVDDLIIPWSELVLKEKIGAGSFGTVHRADWHGSDVAVKILMEQDYHLDR
Sbjct 481
LRSTVSDLSLAVDDLIIPWSELVLKEKIGAGSFGTVHRADWHGSDVAVKILMEQDYHLDR
540

Query 538
FKEFMREVAIMKSLRHPNIVLFMGAVTEPPNLSIVTEYLSRGSLYKLLHRSGAREVLDER
597

FKEFMREVAIMKSLRHPNIVLFMGAVTEPPNLSIVTEYLSRGSLYKLLHRSGAREVLDER
Sbjct 541
FKEFMREVAIMKSLRHPNIVLFMGAVTEPPNLSIVTEYLSRGSLYKLLHRSGAREVLDER
600

Query 598
RRLNMAFDVAKGMNYLHRRSPPIVHRDLKSPNLLVDKKYTVKVCDFGLSRLKANTYLSS
K 657

RRLNMAFDVAKGMNYLHRRSPPIVHRDLKSPNLLVDKKYTVKVCDFGLSRLKANTYLSS
K
Sbjct 601
RRLNMAFDVAKGMNYLHRRSPPIVHRDLKSPNLLVDKKYTVKVCDFGLSRLKANTYLSS
K 660

Query 658
SLAGTPEWMAPEVLRDEPSNEKSDVYSFAVILWELMTLQQPWCNLNPAQVVAAVGFKGR
R 717

SLAGTPEWMAPEVLRDEPSNEKSDVYSFAVILWELMTLQQPWCNLNPAQVVAAVGFKGR
R
Sbjct 661
SLAGTPEWMAPEVLRDEPSNEKSDVYSFAVILWELMTLQQPWCNLNPAQVVAAVGFKGR
R 720

Query 718 LEIPKELNPQVAALIESCWANEPWRRPSFANIMETLRPLINKVPVPQLIRSDS
770

LEIPKELNPQVAALIESCWANEPWRRPSFANIMETLRPLINKVPVPQLIRSDS
Sbjct 721 LEIPKELNPQVAALIESCWANEPWRRPSFANIMETLRPLINKVPVPQLIRSDS
773

>serine/threonine-protein kinase CTR1-like [Triticum dicoccoides]
Sequence ID: XP_037428624.1 Length: 772
>serine/threonine-protein kinase CTR1-like [Triticum dicoccoides]
Sequence ID: XP_037428625.1 Length: 772
>serine/threonine-protein kinase CTR1-like [Triticum aestivum]
Sequence ID: XP_044367797.1 Length: 772
>serine/threonine-protein kinase CTR1-like [Triticum aestivum]
Sequence ID: XP_044367798.1 Length: 772
>unnamed protein product [Triticum turgidum subsp. durum]
Sequence ID: VAI00707.1 Length: 772
Range 1: 1 to 772

Score:1563 bits(4048), Expect:0.0,
Method:Compositional matrix adjust.,
Identities:764/772(99%), Positives:766/772(99%), Gaps:2/772(0%)

Query 1
MELPAAGGGRRTSYSLLSQFPDDAAAAGASPAVLQRQSSGGSSYGAGSSVSASSDYPFHL
60
MELPAAGGGRRTSYSLLSQFPDDAA
AGASPAVLQRQSSGGSSYGAGSSVSASSDYPFHL
Sbjct 1
MELPAAGGGRRTSYSLLSQFPDDAAVAGASPAVLQRQSSGGSSYGAGSSVSASSDYPFHL
60

Query 61
PPAVAAA--GGGGGTPSPCKSWAQQAEETYQLQLALALRLCADAACAADPGFLDPGDSGG
118
PPA AA
GGGGGTPSPCKSWAQQAEETYQLQLALALRLCADAACAADPGFLDPGDSGG
Sbjct 61
PPAAAAGAGGGGGGTPSPCKSWAQQAEETYQLQLALALRLCADAACAADPGFLDPGDS
GG 120

Query 119
SKMGGGGGGSGSGRAFPLAPPSPTAEALSHRFWVNGSLSYSNTIPDGFYLIQGMDPFVWS
178

SKMGGGGGGSGSGRAFPLAPPSPTAEALSHRFWVNGSLSYSNTIPDGFYLIQGMDPFVWS
Sbjct 121
SKMGGGGGGSGSGRAFPLAPPSPTAEALSHRFWVNGSLSYSNTIPDGFYLIQGMDPFVWS

180

Query 179
MCTDVHEENRIPSVESLKSVRPDDSSIQVVLVDRRADFDLGMLENYASSFLSSSSDMKDV
238

MCTDVHEENRIPSVESLKSVRPDDSSIQVVLVDRRADFDLGMLENYASSFLSSSSDMKDV
Sbjct 181
MCTDVHEENRIPSVESLKSVRPDDSSIQVVLVDRRADFDLGMLENYASSFLSSSSDMKDV
240

Query 239
INQLAKLVSSRMGGTTSNEENLLPRWKESSEAIKSSAGSIVLHLGKLPIGLCKHRSLLFK
298

INQLAKLVSSRMGGTTSNEENLLPRWKESSEAIKSSAGSIVLHLGKLPIGLCKHRSLLFK
Sbjct 241
INQLAKLVSSRMGGTTSNEENLLPRWKESSEAIKSSAGSIVLHLGKLPIGLCKHRSLLFK
300

Query 299
MLADKVNIPCRLVKGCKYCKAEDASSCVVRFGLEREYLVDLFGAPGQLSDPDSFVNGPYS
358

MLADKVNIPCRLVKGCKYCKAEDASSCVVRFGLEREYLVDLFGAPGQLSDPDSFVNGPYS
Sbjct 301
MLADKVNIPCRLVKGCKYCKAEDASSCVVRFGLEREYLVDLFGAPGQLSDPDSFVNGPYS
360

Query 359
LSVPSPLRPPKFRSLEITSNFSSVAKQYFSDCHSLNLLFSDASTGASNGAAVAVDQMYSK
418

LSVPSPLRPPKFRSLEITSNFSSVAKQYFSDCHSLNLLFSDASTGASNGAAVAVDQMYSK
Sbjct 361
LSVPSPLRPPKFRSLEITSNFSSVAKQYFSDCHSLNLLFSDASTGASNGAAVAVDQMYSK
420

Query 419
KHDAGDGIANSWVPVKGQAIANSDIILPEAPREVLPLMSPSNLTADKKKEFQLIEGNQYL
478

KHDAGDGIANSWVPVKGQAIANSDIILPEAPREVLPLM+PSNLTADKKKEFQLIEGNQYL
Sbjct 421

KHDAGDGIANSWVPVKGQAIANSDIILPEAPREVLPLMTPSNLTADKKKEFQLIEGNQYL
480

Query 479
RSTVSDLSLAVDDLIIPWSELVLKEKIGAGSFGTVHRADWHGSDVAVKILMEQDYHLDRF
538

RSTVSDLSLAVDDLIIPWSELVLKEKIGAGSFGTVHRADWHGSDVAVKILMEQDYHLDRF
Sbjct 481
RSTVSDLSLAVDDLIIPWSELVLKEKIGAGSFGTVHRADWHGSDVAVKILMEQDYHLDRF
540

Query 539
KEFMREVAIMKSLRHPNIVLFMGAVTEPPNLSIVTEYLSRGSLYKLLHRSGAREVLDERR
598

KEFMREVAIMKSLRHPNIVLFMGAVTEPPNLSIVTEYLSRGSLYKLLHRSGAREVLDERR
Sbjct 541
KEFMREVAIMKSLRHPNIVLFMGAVTEPPNLSIVTEYLSRGSLYKLLHRSGAREVLDERR
600

Query 599
RLNMAFDVAKGMNYLHRRSPPIVHRDLKSPNLLVDKKYTVKVCDFGLSRLKANTYLSSK
S 658

RLNMAFDVAKGMNYLHRRSPPIVHRDLKSPNLLVDKKYTVKVCDFGLSRLKANTYLSSK
S
Sbjct 601
RLNMAFDVAKGMNYLHRRSPPIVHRDLKSPNLLVDKKYTVKVCDFGLSRLKANTYLSSK
S 660

Query 659
LAGTPEWMAPEVLRDEPSNEKSDVYSFAVILWELMTLQQPWCNLNPAQVVAAVGFKGRR
L 718

LAGTPEWMAPEVLRDEPSNEKSDVYSFAVILWELMTLQQPWCNLNPAQVVAAVGFKGRR
L
Sbjct 661
LAGTPEWMAPEVLRDEPSNEKSDVYSFAVILWELMTLQQPWCNLNPAQVVAAVGFKGRR
L 720

Query 719 EIPKELNPQVAALIESCWANEPWRRPSFANIMETLRPLINKVPVPQLIRSDS
770

EIPK+LNPQVA LIESCWANEPWRRPSFANIMETLRPLINKVPVPQLIRSDS

Sbjct 721 EIPKDLNPQVAVLIESCWANEPWRRPSFANIMETLRPLINKVPVPQLIRSDS 772

>uncharacterized protein LOC123095596 [Triticum aestivum]
Sequence ID: XP_044373050.1 Length: 1684
Range 1: 1 to 762

Score:1560 bits(4040), Expect:0.0,
Method:Compositional matrix adjust.,
Identities:759/762(99%), Positives:760/762(99%), Gaps:0/762(0%)

Query 1 MELPAAGGGRRTSYSLLSQFPDDAAAAGASPAVLQRQSSGGSSYGAGSSVSASSDYPFHL 60

MELPAAGGGRRTSYSLLSQFPDDAAAAGASPAVLQRQSSGGSSYGAGSSVSASSDYPFHL
Sbjct 1 MELPAAGGGRRTSYSLLSQFPDDAAAAGASPAVLQRQSSGGSSYGAGSSVSASSDYPFHL 60

Query 61 PPAVAAAGGGGGTPSPCKSWAQQAEETYQLQLALALRLCADAACAADPGFLDPGDSGGSK 120

PPAVAAAGGGGGTPSPCKSWAQQAEETYQLQLALALRLCADAACAADPGFLDPGDSGGSK
Sbjct 61 PPAVAAAGGGGGTPSPCKSWAQQAEETYQLQLALALRLCADAACAADPGFLDPGDSGGSK 120

Query 121 MGGGGGGSGSGRAFPLAPPSPTAEALSHRFWVNGSLSYSNTIPDGFYLIQGMDPFVWSMC 180

MGGGGGGSGSGRAFPLAPPSPTAEALSHRFWVNGSLSYSNTIPDGFYLIQGMDPFVWSMC
Sbjct 121 MGGGGGGSGSGRAFPLAPPSPTAEALSHRFWVNGSLSYSNTIPDGFYLIQGMDPFVWSMC 180

Query 181 TDVHEENRIPSVESLKSVRPDDSSIQVVLVDRRADFDLGMLENYASSFLSSSSDMKDVIN

240

TDVHEENRIPSVESLKSVRPDDSSIQVVLVDRRADFDLGMLENYASSFLSSSSDMKDVIN
Sbjct 181
TDVHEENRIPSVESLKSVRPDDSSIQVVLVDRRADFDLGMLENYASSFLSSSSDMKDVIN
240

Query 241
QLAKLVSSRMGGTTSNEENLLPRWKESSEAIKSSAGSIVLHLGKLPIGLCKHRSLLFKML
300

QLAKLVSSRMGGTTSNEENLLPRWKESSEAIKSSAGSIVLHLGKLPIGLCKHRSLLFKML
Sbjct 241
QLAKLVSSRMGGTTSNEENLLPRWKESSEAIKSSAGSIVLHLGKLPIGLCKHRSLLFKML
300

Query 301
ADKVNIPCRLVKGCKYCKAEDASSCVVRFGLEREYLVDLFGAPGQLSDPDSFVNGPYSLS
360

ADKVNIPCRLVKGCKYCKAEDASSCVVRFGLEREYLVDLFGAPGQLSDPDSFVNGPYSLS
Sbjct 301
ADKVNIPCRLVKGCKYCKAEDASSCVVRFGLEREYLVDLFGAPGQLSDPDSFVNGPYSLS
360

Query 361
VPSPLRPPKFRSLEITSNFSSVAKQYFSDCHSLNLLFSDASTGASNGAAVAVDQMYSKKH
420

VPSPLRPPKFRSLEITSNFSSVAKQYFSDCHSLNLLFSDASTGASNGAAVAVDQMYSKKH
Sbjct 361
VPSPLRPPKFRSLEITSNFSSVAKQYFSDCHSLNLLFSDASTGASNGAAVAVDQMYSKKH
420

Query 421
DAGDGIANSWVPVKGQAIANSDIILPEAPREVLPLMSPSNLTADKKKEFQLIEGNQYLRS
480

DAGDGIANSWVPVKGQAIANSDIILPEAPREVLPLMSPSNLTADKKKEFQLIEGNQYLRS
Sbjct 421
DAGDGIANSWVPVKGQAIANSDIILPEAPREVLPLMSPSNLTADKKKEFQLIEGNQYLRS
480

Query 481

TVSDLSLAVDDLIIPWSELVLKEKIGAGSFGTVHRADWHGSDVAVKILMEQDYHLDRFKE
540

TVSDLSLAVDDLIIPWSELVLKEKIGAGSFGTVHRADWHGSDVAVKILMEQDYHLDRFKE
Sbjct 481
TVSDLSLAVDDLIIPWSELVLKEKIGAGSFGTVHRADWHGSDVAVKILMEQDYHLDRFKE
540

Query 541
FMREVAIMKSLRHPNIVLFMGAVTEPPNLSIVTEYLSRGSLYKLLHRSGAREVLDERRRL
600

FMREVAIMKSLRHPNIVLFMGAVTEPPNLSIVTEYLSRGSLYKLLHRSGAREVLDERRRL
Sbjct 541
FMREVAIMKSLRHPNIVLFMGAVTEPPNLSIVTEYLSRGSLYKLLHRSGAREVLDERRRL
600

Query 601
NMAFDVAKGMNYLHRRSPPIVHRDLKSPNLLVDKKYTVKVCDFGLSRLKANTYLSSKSL
A 660

NMAFDVAKGMNYLHRRSPPIVHRDLKSPNLLVDKKYTVKVCDFGLSRLKANTYLSSKSL
A
Sbjct 601
NMAFDVAKGMNYLHRRSPPIVHRDLKSPNLLVDKKYTVKVCDFGLSRLKANTYLSSKSL
A 660

Query 661
GTPEWMAPEVLRDEPSNEKSDVYSFAVILWELMTLQQPWCNLNPAQVVAAVGFKGRRLEI
720

GTPEWMAPEVLRDEPSNEKSDVYSFAVILWELMTLQQPWCNLNPAQVVAAVGFKGRRLEI
Sbjct 661
GTPEWMAPEVLRDEPSNEKSDVYSFAVILWELMTLQQPWCNLNPAQVVAAVGFKGRRLEI
720

Query 721 PKELNPQVAALIESCWANEPWRRPSFANIMETLRPLINKVPV 762
PKELNPQVAALIESCWANEPWRRPSFANIMETLRPLINK +
Sbjct 721 PKELNPQVAALIESCWANEPWRRPSFANIMETLRPLINKCTI 762

>hypothetical protein CFC21_053784 [Triticum aestivum]

Sequence ID: KAF7044576.1 Length: 763
Range 1: 1 to 763

Score:1540 bits(3986), Expect:0.0,
Method:Compositional matrix adjust.,
Identities:758/773(98%), Positives:759/773(98%), Gaps:13/773(1%)

Query 1
MELPAAGGGRRTSYSLLSQFPDDAAAAGASPAVLQRQSSGGSSYGAGSSVSASSDYPFHL
60

MELPAAGGGRRTSYSLLSQFPDDAAAAGASPAVLQRQSSGGSSYGAGSSVSASSDYPFHL
Sbjct 1
MELPAAGGGRRTSYSLLSQFPDDAAAAGASPAVLQRQSSGGSSYGAGSSVSASSDYPFHL
60

Query 61
PPAVAAAGGGGG--TPSPCKSWAQQAEETYQLQLALALRLCADAACAADPGFLDPGDSGG
118
PPA AAAGGGGG
TPSPCKSWAQQAEETYQLQLALALRLCADAACAADPGFLDPGDSGG
Sbjct 61
PPAAAAAGGGGGGGTPSPCKSWAQQAEETYQLQLALALRLCADAACAADPGFLDPGDS
GG 120

Query 119
SKMGGGGGG-SGSGRAFPLAPPSPTAEALSHRFWVNGSLSYSNTIPDGFYLIQGMDPFVW
177
SKMGGGGGG SGSGRAFPLAPPSPTAEALSHRFW
IPDGFYLIQGMDPFVW
Sbjct 121
SKMGGGGGGGSGSGRAFPLAPPSPTAEALSHRFW----------IPDGFYLIQGMDPFVW 170

Query 178
SMCTDVHEENRIPSVESLKSVRPDDSSIQVVLVDRRADFDLGMLENYASSFLSSSSDMKD
237

SMCTDVHEENRIPSVESLKSVRPDDSSIQVVLVDRRADFDLGMLENYASSFLSSSSDMKD
Sbjct 171
SMCTDVHEENRIPSVESLKSVRPDDSSIQVVLVDRRADFDLGMLENYASSFLSSSSDMKD
230

Query 238
VINQLAKLVSSRMGGTTSNEENLLPRWKESSEAIKSSAGSIVLHLGKLPIGLCKHRSLLF

297

VINQLAKLVSSRMGGTTSNEENLLPRWKESSEAIKSSAGSIVLHLGKLPIGLCKHRSLLF
Sbjct 231
VINQLAKLVSSRMGGTTSNEENLLPRWKESSEAIKSSAGSIVLHLGKLPIGLCKHRSLLF
290

Query 298
KMLADKVNIPCRLVKGCKYCKAEDASSCVVRFGLEREYLVDLFGAPGQLSDPDSFVNGP
Y 357

KMLADKVNIPCRLVKGCKYCKAEDASSCVVRFGLEREYLVDLFGAPGQLSDPDSFVNGP
Y
Sbjct 291
KMLADKVNIPCRLVKGCKYCKAEDASSCVVRFGLEREYLVDLFGAPGQLSDPDSFVNGP
Y 350

Query 358
SLSVPSPLRPPKFRSLEITSNFSSVAKQYFSDCHSLNLLFSDASTGASNGAAVAVDQMYS
417

SLSVPSPLRPPKFRSLEITSNFSSVAKQYFSDCHSLNLLFSDASTGASNGAAVAVDQMYS
Sbjct 351
SLSVPSPLRPPKFRSLEITSNFSSVAKQYFSDCHSLNLLFSDASTGASNGAAVAVDQMYS
410

Query 418
KKHDAGDGIANSWVPVKGQAIANSDIILPEAPREVLPLMSPSNLTADKKKEFQLIEGNQY
477

KKHDAGDGIANSWVPVKGQAIANSDIILPEAPREVLPLM+PSNLTADKKKEFQLIEGNQY
Sbjct 411
KKHDAGDGIANSWVPVKGQAIANSDIILPEAPREVLPLMTPSNLTADKKKEFQLIEGNQY
470

Query 478
LRSTVSDLSLAVDDLIIPWSELVLKEKIGAGSFGTVHRADWHGSDVAVKILMEQDYHLDR
537

LRSTVSDLSLAVDDLIIPWSELVLKEKIGAGSFGTVHRADWHGSDVAVKILMEQDYHLDR
Sbjct 471
LRSTVSDLSLAVDDLIIPWSELVLKEKIGAGSFGTVHRADWHGSDVAVKILMEQDYHLDR
530

Query 538
FKEFMREVAIMKSLRHPNIVLFMGAVTEPPNLSIVTEYLSRGSLYKLLHRSGAREVLDER
597

FKEFMREVAIMKSLRHPNIVLFMGAVTEPPNLSIVTEYLSRGSLYKLLHRSGAREVLDER
Sbjct 531
FKEFMREVAIMKSLRHPNIVLFMGAVTEPPNLSIVTEYLSRGSLYKLLHRSGAREVLDER
590

Query 598
RRLNMAFDVAKGMNYLHRRSPPIVHRDLKSPNLLVDKKYTVKVCDFGLSRLKANTYLSS
K 657

RRLNMAFDVAKGMNYLHRRSPPIVHRDLKSPNLLVDKKYTVKVCDFGLSRLKANTYLSS
K
Sbjct 591
RRLNMAFDVAKGMNYLHRRSPPIVHRDLKSPNLLVDKKYTVKVCDFGLSRLKANTYLSS
K 650

Query 658
SLAGTPEWMAPEVLRDEPSNEKSDVYSFAVILWELMTLQQPWCNLNPAQVVAAVGFKGR
R 717

SLAGTPEWMAPEVLRDEPSNEKSDVYSFAVILWELMTLQQPWCNLNPAQVVAAVGFKGR
R
Sbjct 651
SLAGTPEWMAPEVLRDEPSNEKSDVYSFAVILWELMTLQQPWCNLNPAQVVAAVGFKGR
R 710

Query 718 LEIPKELNPQVAALIESCWANEPWRRPSFANIMETLRPLINKVPVPQLIRSDS
770

LEIPKELNPQVAALIESCWANEPWRRPSFANIMETLRPLINKVPVPQLIRSDS
Sbjct 711 LEIPKELNPQVAALIESCWANEPWRRPSFANIMETLRPLINKVPVPQLIRSDS
763

>Serine/threonine-protein kinase CTR1 [Hordeum vulgare]
Sequence ID: KAE8817350.1 Length: 773
Range 1: 1 to 773

Score:1536 bits(3977), Expect:0.0,
Method:Compositional matrix adjust.,

Identities:755/775(97%), Positives:759/775(97%), Gaps:7/775(0%)

Query 1
MELPAAGGGRRTSYSLLSQFPDDAAAA--GASPAVLQRQSSGGSSYGAGSSVSASSDYPF
58
MELPAAGGGRRTSYSLLSQFPDDA
GASPAVLQRQSSGGSSYGAGSSVSASSDYPF
Sbjct 1
MELPAAGGGRRTSYSLLSQFPDDAGGGTTGASPAVLQRQSSGGSSYGAGSSVSASSDYPF
60

Query 59
HLPPAVAA---AGGGGGTPSPCKSWAQQAEETYQLQLALALRLCADAACAADPGFLDPGD
115
HLPPA AA
GGGGGTPSPCKSWAQQAEETYQLQLALALRLCADAACAADPGFLDPGD
Sbjct 61
HLPPATAAVGGGGGGGGTPSPCKSWAQQAEETYQLQLALALRLCADAACAADPGFLDPG
D 120

Query 116
SGGSKMGGGGGGSGSGRAFPLAPPSPTAEALSHRFWVNGSLSYSNTIPDGFYLIQGMDPF
175
S GSKMGGGG
SGSGRAFPLAPPSPTAEALSHRFWVNGSLSYSNTIPDGFYLIQGMDPF
Sbjct 121
SAGSKMGGGG--SGSGRAFPLAPPSPTAEALSHRFWVNGSLSYSNTIPDGFYLIQGMDPF
178

Query 176
VWSMCTDVHEENRIPSVESLKSVRPDDSSIQVVLVDRRADFDLGMLENYASSFLSSSSDM
235

VWSMCTDVHEENRIPSVESLKSVRPDDSSIQVVLVDRRADFDLGMLENYASSFLSSSSDM
Sbjct 179
VWSMCTDVHEENRIPSVESLKSVRPDDSSIQVVLVDRRADFDLGMLENYASSFLSSSSDM
238

Query 236
KDVINQLAKLVSSRMGGTTSNEENLLPRWKESSEAIKSSAGSIVLHLGKLPIGLCKHRSL
295

KDVINQLAKLVSSRMGGTTSNEENLLPRWKESSEAIKSSAGSIVLHLGKLPIGLCKHRSL
Sbjct 239

KDVINQLAKLVSSRMGGTTSNEENLLPRWKESSEAIKSSAGSIVLHLGKLPIGLCKHRSL
298

Query 296
LFKMLADKVNIPCRLVKGCKYCKAEDASSCVVRFGLEREYLVDLFGAPGQLSDPDSFVN
G 355

LFKMLADKVNIPCRLVKGCKYCKAEDASSCVVRFGLEREYLVDLFGAPGQLSDPDSFVN
G
Sbjct 299
LFKMLADKVNIPCRLVKGCKYCKAEDASSCVVRFGLEREYLVDLFGAPGQLSDPDSFVN
G 358

Query 356
PYSLSVPSPLRPPKFRSLEITSNFSSVAKQYFSDCHSLNLLFSDASTGASNGAAVAVDQM
415

PYSLSVPSPLRPPKFRSLEITSNFSSVAKQYFSDCHSLNLLFSDASTGAS+GAAV VDQM
Sbjct 359
PYSLSVPSPLRPPKFRSLEITSNFSSVAKQYFSDCHSLNLLFSDASTGASSGAAVPVDQM
418

Query 416
YSKKHDAGDGIANSWVPVKGQAIANSDIILPEAPREVLPLMSPSNLTADKKKEFQLIEGN
475
YSKKHDAGDGIANSWVPVKGQAIAN
DIILPEAPRE+LPLM+PSNLTADKKKEFQLIEGN
Sbjct 419
YSKKHDAGDGIANSWVPVKGQAIANPDIILPEAPREILPLMTPSNLTADKKKEFQLIEGN
478

Query 476
QYLRSTVSDLSLAVDDLIIPWSELVLKEKIGAGSFGTVHRADWHGSDVAVKILMEQDYHL
535

QYLRSTVSDLSLAVDDLIIPWSELVLKEKIGAGSFGTVHRADWHGSDVAVKILMEQDYHL
Sbjct 479
QYLRSTVSDLSLAVDDLIIPWSELVLKEKIGAGSFGTVHRADWHGSDVAVKILMEQDYHL
538

Query 536
DRFKEFMREVAIMKSLRHPNIVLFMGAVTEPPNLSIVTEYLSRGSLYKLLHRSGAREVLD
595

DRFKEFMREVAIMKSLRHPNIVLFMGAVTEPPNLSIVTEYLSRGSLYKLLHRSGAREVLD
Sbjct 539
DRFKEFMREVAIMKSLRHPNIVLFMGAVTEPPNLSIVTEYLSRGSLYKLLHRSGAREVLD
598

Query 596
ERRRLNMAFDVAKGMNYLHRRSPPIVHRDLKSPNLLVDKKYTVKVCDFGLSRLKANTYL
S 655

ERRRLNMAFDVAKGMNYLHRRSPPIVHRDLKSPNLLVDKKYTVKVCDFGLSRLKANTYL
S
Sbjct 599
ERRRLNMAFDVAKGMNYLHRRSPPIVHRDLKSPNLLVDKKYTVKVCDFGLSRLKANTYL
S 658

Query 656
SKSLAGTPEWMAPEVLRDEPSNEKSDVYSFAVILWELMTLQQPWCNLNPAQVVAAVGFK
G 715

SKSLAGTPEWMAPEVLRDEPSNEKSDVYSFAVILWELMTLQQPWCNLNPAQVVAAVGFK
G
Sbjct 659
SKSLAGTPEWMAPEVLRDEPSNEKSDVYSFAVILWELMTLQQPWCNLNPAQVVAAVGFK
G 718

Query 716
RRLEIPKELNPQVAALIESCWANEPWRRPSFANIMETLRPLINKVPVPQLIRSDS 770
RRLEIPK+LNPQVAALIESCW
NEPWRRPSFANIMETLRPLINKVPVPQLIRSDS
Sbjct 719
RRLEIPKDLNPQVAALIESCWINEPWRRPSFANIMETLRPLINKVPVPQLIRSDS 773

>hypothetical protein ZWY2020_054149 [Hordeum vulgare]
Sequence ID: KAI4998807.1 Length: 814
Range 1: 1 to 773

Score:1536 bits(3977), Expect:0.0,
Method:Compositional matrix adjust.,
Identities:756/775(98%), Positives:760/775(98%), Gaps:7/775(0%)

Query 1

MELPAAGGGRRTSYSLLSQFPDDAAAA--GASPAVLQRQSSGGSSYGAGSSVSASSDYPF 58

MELPAAGGGRRTSYSLLSQFPDDA GASPAVLQRQSSGGSSYGAGSSVSASSDYPF

Sbjct 1 MELPAAGGGRRTSYSLLSQFPDDAGGGTTGASPAVLQRQSSGGSSYGAGSSVSASSDYPF 60

Query 59 HLPPAVAA---AGGGGGTPSPCKSWAQQAEETYQLQLALALRLCADAACAADPGFLDPGD 115

HLPPA AA GGGGGTPSPCKSWAQQAEETYQLQLALALRLCADAACAADPGFLDPGD

Sbjct 61 HLPPATAAVGGGGGGGGTPSPCKSWAQQAEETYQLQLALALRLCADAACAADPGFLDPGD 120

Query 116 SGGSKMGGGGGGSGSGRAFPLAPPSPTAEALSHRFWVNGSLSYSNTIPDGFYLIQGMDPF 175

S GSKMGGGG SGSGRAFPLAPPSPTAEALSHRFWVNGSLSYSNTIPDGFYLIQGMDPF

Sbjct 121 SAGSKMGGGG--SGSGRAFPLAPPSPTAEALSHRFWVNGSLSYSNTIPDGFYLIQGMDPF 178

Query 176 VWSMCTDVHEENRIPSVESLKSVRPDDSSIQVVLVDRRADFDLGMLENYASSFLSSSSDM 235

VWSMCTDVHEENRIPSVESLKSVRPDDSSIQVVLVDRRADFDLGMLENYASSFLSSSSDM

Sbjct 179 VWSMCTDVHEENRIPSVESLKSVRPDDSSIQVVLVDRRADFDLGMLENYASSFLSSSSDM 238

Query 236 KDVINQLAKLVSSRMGGTTSNEENLLPRWKESSEAIKSSAGSIVLHLGKLPIGLCKHRSL 295

KDVINQLAKLVSSRMGGTTSNEENLLPRWKESSEAIKSSAGSIVLHLGKLPIGLCKHRSL

Sbjct 239 KDVINQLAKLVSSRMGGTTSNEENLLPRWKESSEAIKSSAGSIVLHLGKLPIGLCKHRSL 298

Query 296
LFKMLADKVNIPCRLVKGCKYCKAEDASSCVVRFGLEREYLVDLFGAPGQLSDPDSFVN
G 355

LFKMLADKVNIPCRLVKGCKYCKAEDASSCVVRFGLEREYLVDLFGAPGQLSDPDSFVN
G
Sbjct 299
LFKMLADKVNIPCRLVKGCKYCKAEDASSCVVRFGLEREYLVDLFGAPGQLSDPDSFVN
G 358

Query 356
PYSLSVPSPLRPPKFRSLEITSNFSSVAKQYFSDCHSLNLLFSDASTGASNGAAVAVDQM
415

PYSLSVPSPLRPPKFRSLEITSNFSSVAKQYFSDCHSLNLLFSDASTGAS+GAAV VDQM
Sbjct 359
PYSLSVPSPLRPPKFRSLEITSNFSSVAKQYFSDCHSLNLLFSDASTGASSGAAVPVDQM
418

Query 416
YSKKHDAGDGIANSWVPVKGQAIANSDIILPEAPREVLPLMSPSNLTADKKKEFQLIEGN
475

YSKKHDAGDGIANSWVPVKGQAIAN
DIILPEAPRE+LPLM+PSNLTADKKKEFQLIEGN
Sbjct 419
YSKKHDAGDGIANSWVPVKGQAIANPDIILPEAPREILPLMTPSNLTADKKKEFQLIEGN
478

Query 476
QYLRSTVSDLSLAVDDLIIPWSELVLKEKIGAGSFGTVHRADWHGSDVAVKILMEQDYHL
535

QYLRSTVSDLSLAVDDLIIPWSELVLKEKIGAGSFGTVHRADWHGSDVAVKILMEQDYHL
Sbjct 479
QYLRSTVSDLSLAVDDLIIPWSELVLKEKIGAGSFGTVHRADWHGSDVAVKILMEQDYHL
538

Query 536
DRFKEFMREVAIMKSLRHPNIVLFMGAVTEPPNLSIVTEYLSRGSLYKLLHRSGAREVLD
595

DRFKEFMREVAIMKSLRHPNIVLFMGAVTEPPNLSIVTEYLSRGSLYKLLHRSGAREVLD
Sbjct 539
DRFKEFMREVAIMKSLRHPNIVLFMGAVTEPPNLSIVTEYLSRGSLYKLLHRSGAREVLD

598

Query 596
ERRRLNMAFDVAKGMNYLHRRSPPIVHRDLKSPNLLVDKKYTVKVCDFGLSRLKANTYL
S 655

ERRRLNMAFDVAKGMNYLHRRSPPIVHRDLKSPNLLVDKKYTVKVCDFGLSRLKANTYL
S
Sbjct 599
ERRRLNMAFDVAKGMNYLHRRSPPIVHRDLKSPNLLVDKKYTVKVCDFGLSRLKANTYL
S 658

Query 656
SKSLAGTPEWMAPEVLRDEPSNEKSDVYSFAVILWELMTLQQPWCNLNPAQVVAAVGFK
G 715

SKSLAGTPEWMAPEVLRDEPSNEKSDVYSFAVILWELMTLQQPWCNLNPAQVVAAVGFK
G
Sbjct 659
SKSLAGTPEWMAPEVLRDEPSNEKSDVYSFAVILWELMTLQQPWCNLNPAQVVAAVGFK
G 718

Query 716
RRLEIPKELNPQVAALIESCWANEPWRRPSFANIMETLRPLINKVPVPQLIRSDS 770

RRLEIPK+LNPQVAALIESCWANEPWRRPSFANIMETLRPLINKVPVPQLIRSDS
Sbjct 719
RRLEIPKDLNPQVAALIESCWANEPWRRPSFANIMETLRPLINKVPVPQLIRSDS 773

>unnamed protein product [Triticum turgidum subsp. durum]
Sequence ID: VAH96052.1 Length: 755
Range 1: 1 to 740

Score:1495 bits(3870), Expect:0.0,
Method:Compositional matrix adjust.,
Identities:735/740(99%), Positives:736/740(99%), Gaps:3/740(0%)

Query 1
MELPAAGGGRRTSYSLLSQFPDDAAAAGASPAVLQRQSSGGSSYGAGSSVSASSDYPFHL
60

MELPAAGGGRRTSYSLLSQFPDDAAAAGASPAVLQRQSSGGSSYGAGSSVSASSDYPFHL
Sbjct 1
MELPAAGGGRRTSYSLLSQFPDDAAAAGASPAVLQRQSSGGSSYGAGSSVSASSDYPFHL
60

Query 61
PPAVAAAGGGGG--TPSPCKSWAQQAEETYQLQLALALRLCADAACAADPGFLDPGDSGG
118
PPA AAAGGGGG
TPSPCKSWAQQAEETYQLQLALALRLCADAACAADPGFLDPGDSGG
Sbjct 61
PPAAAAAGGGGGGGTPSPCKSWAQQAEETYQLQLALALRLCADAACAADPGFLDPGDS
GG 120

Query 119
SKMGGGGGG-SGSGRAFPLAPPSPTAEALSHRFWVNGSLSYSNTIPDGFYLIQGMDPFVW
177
SKMGGGGGG
SGSGRAFPLAPPSPTAEALSHRFWVNGSLSYSNTIPDGFYLIQGMDPFVW
Sbjct 121
SKMGGGGGGGSGSGRAFPLAPPSPTAEALSHRFWVNGSLSYSNTIPDGFYLIQGMDPFVW
180

Query 178
SMCTDVHEENRIPSVESLKSVRPDDSSIQVVLVDRRADFDLGMLENYASSFLSSSSDMKD
237

SMCTDVHEENRIPSVESLKSVRPDDSSIQVVLVDRRADFDLGMLENYASSFLSSSSDMKD
Sbjct 181
SMCTDVHEENRIPSVESLKSVRPDDSSIQVVLVDRRADFDLGMLENYASSFLSSSSDMKD
240

Query 238
VINQLAKLVSSRMGGTTSNEENLLPRWKESSEAIKSSAGSIVLHLGKLPIGLCKHRSLLF
297

VINQLAKLVSSRMGGTTSNEENLLPRWKESSEAIKSSAGSIVLHLGKLPIGLCKHRSLLF
Sbjct 241
VINQLAKLVSSRMGGTTSNEENLLPRWKESSEAIKSSAGSIVLHLGKLPIGLCKHRSLLF
300

Query 298
KMLADKVNIPCRLVKGCKYCKAEDASSCVVRFGLEREYLVDLFGAPGQLSDPDSFVNGP
Y 357

KMLADKVNIPCRLVKGCKYCKAEDASSCVVRFGLEREYLVDLFGAPGQLSDPDSFVNGP
Y
Sbjct 301
KMLADKVNIPCRLVKGCKYCKAEDASSCVVRFGLEREYLVDLFGAPGQLSDPDSFVNGP
Y 360

Query 358
SLSVPSPLRPPKFRSLEITSNFSSVAKQYFSDCHSLNLLFSDASTGASNGAAVAVDQMYS
417

SLSVPSPLRPPKFRSLEITSNFSSVAKQYFSDCHSLNLLFSDASTGASNGAAVAVDQMYS
Sbjct 361
SLSVPSPLRPPKFRSLEITSNFSSVAKQYFSDCHSLNLLFSDASTGASNGAAVAVDQMYS
420

Query 418
KKHDAGDGIANSWVPVKGQAIANSDIILPEAPREVLPLMSPSNLTADKKKEFQLIEGNQY
477

KKHDAGDGIANSWVPVKGQAIANSDIILPEAPREVLPLM+PSNLTADKKKEFQLIEGNQY
Sbjct 421
KKHDAGDGIANSWVPVKGQAIANSDIILPEAPREVLPLMTPSNLTADKKKEFQLIEGNQY
480

Query 478
LRSTVSDLSLAVDDLIIPWSELVLKEKIGAGSFGTVHRADWHGSDVAVKILMEQDYHLDR
537

LRSTVSDLSLAVDDLIIPWSELVLKEKIGAGSFGTVHRADWHGSDVAVKILMEQDYHLDR
Sbjct 481
LRSTVSDLSLAVDDLIIPWSELVLKEKIGAGSFGTVHRADWHGSDVAVKILMEQDYHLDR
540

Query 538
FKEFMREVAIMKSLRHPNIVLFMGAVTEPPNLSIVTEYLSRGSLYKLLHRSGAREVLDER
597

FKEFMREVAIMKSLRHPNIVLFMGAVTEPPNLSIVTEYLSRGSLYKLLHRSGAREVLDER
Sbjct 541
FKEFMREVAIMKSLRHPNIVLFMGAVTEPPNLSIVTEYLSRGSLYKLLHRSGAREVLDER
600

Query 598

RRLNMAFDVAKGMNYLHRRSPPIVHRDLKSPNLLVDKKYTVKVCDFGLSRLKANTYLSS
K 657

RRLNMAFDVAKGMNYLHRRSPPIVHRDLKSPNLLVDKKYTVKVCDFGLSRLKANTYLSS
K
Sbjct 601
RRLNMAFDVAKGMNYLHRRSPPIVHRDLKSPNLLVDKKYTVKVCDFGLSRLKANTYLSS
K 660

Query 658
SLAGTPEWMAPEVLRDEPSNEKSDVYSFAVILWELMTLQQPWCNLNPAQVVAAVGFKGR
R 717

SLAGTPEWMAPEVLRDEPSNEKSDVYSFAVILWELMTLQQPWCNLNPAQVVAAVGFKGR
R
Sbjct 661
SLAGTPEWMAPEVLRDEPSNEKSDVYSFAVILWELMTLQQPWCNLNPAQVVAAVGFKGR
R 720

Query 718 LEIPKELNPQVAALIESCWA 737
LEIPKELNPQVAALIESCWA
Sbjct 721 LEIPKELNPQVAALIESCWA 740

>unnamed protein product [Triticum turgidum subsp. durum]
Sequence ID: VAI00708.1 Length: 728
Range 1: 1 to 728

Score:1467 bits(3799), Expect:0.0,
Method:Compositional matrix adjust.,
Identities:722/770(94%), Positives:724/770(94%), Gaps:42/770(5%)

Query 1
MELPAAGGGRRTSYSLLSQFPDDAAAAGASPAVLQRQSSGGSSYGAGSSVSASSDYPFHL
60
MELPAAGGGRRTSYSLLSQF
Sbjct 1 MELPAAGGGRRTSYSLLSQF-- 20

Query 61
PPAVAAAGGGGGTPSPCKSWAQQAEETYQLQLALALRLCADAACAADPGFLDPGDSGGS
K 120
A A

GGGGGTPSPCKSWAQQAEETYQLQLALALRLCADAACAADPGFLDPGDSGGSK
Sbjct 21
--AAGAGGGGGGTPSPCKSWAQQAEETYQLQLALALRLCADAACAADPGFLDPGDSGGS
K 78

Query 121
MGGGGGGSGSGRAFPLAPPSPTAEALSHRFWVNGSLSYSNTIPDGFYLIQGMDPFVWSMC
180

MGGGGGGSGSGRAFPLAPPSPTAEALSHRFWVNGSLSYSNTIPDGFYLIQGMDPFVWSMC
Sbjct 79
MGGGGGGSGSGRAFPLAPPSPTAEALSHRFWVNGSLSYSNTIPDGFYLIQGMDPFVWSMC
138

Query 181
TDVHEENRIPSVESLKSVRPDDSSIQVVLVDRRADFDLGMLENYASSFLSSSSDMKDVIN
240

TDVHEENRIPSVESLKSVRPDDSSIQVVLVDRRADFDLGMLENYASSFLSSSSDMKDVIN
Sbjct 139
TDVHEENRIPSVESLKSVRPDDSSIQVVLVDRRADFDLGMLENYASSFLSSSSDMKDVIN
198

Query 241
QLAKLVSSRMGGTTSNEENLLPRWKESSEAIKSSAGSIVLHLGKLPIGLCKHRSLLFKML
300

QLAKLVSSRMGGTTSNEENLLPRWKESSEAIKSSAGSIVLHLGKLPIGLCKHRSLLFKML
Sbjct 199
QLAKLVSSRMGGTTSNEENLLPRWKESSEAIKSSAGSIVLHLGKLPIGLCKHRSLLFKML
258

Query 301
ADKVNIPCRLVKGCKYCKAEDASSCVVRFGLEREYLVDLFGAPGQLSDPDSFVNGPYSLS
360

ADKVNIPCRLVKGCKYCKAEDASSCVVRFGLEREYLVDLFGAPGQLSDPDSFVNGPYSLS
Sbjct 259
ADKVNIPCRLVKGCKYCKAEDASSCVVRFGLEREYLVDLFGAPGQLSDPDSFVNGPYSLS
318

Query 361
VPSPLRPPKFRSLEITSNFSSVAKQYFSDCHSLNLLFSDASTGASNGAAVAVDQMYSKKH
420

VPSPLRPPKFRSLEITSNFSSVAKQYFSDCHSLNLLFSDASTGASNGAAVAVDQMYSKKH
Sbjct 319
VPSPLRPPKFRSLEITSNFSSVAKQYFSDCHSLNLLFSDASTGASNGAAVAVDQMYSKKH
378

Query 421
DAGDGIANSWVPVKGQAIANSDIILPEAPREVLPLMSPSNLTADKKKEFQLIEGNQYLRS
480

DAGDGIANSWVPVKGQAIANSDIILPEAPREVLPLM+PSNLTADKKKEFQLIEGNQYLRS
Sbjct 379
DAGDGIANSWVPVKGQAIANSDIILPEAPREVLPLMTPSNLTADKKKEFQLIEGNQYLRS
438

Query 481
TVSDLSLAVDDLIIPWSELVLKEKIGAGSFGTVHRADWHGSDVAVKILMEQDYHLDRFKE
540

TVSDLSLAVDDLIIPWSELVLKEKIGAGSFGTVHRADWHGSDVAVKILMEQDYHLDRFKE
Sbjct 439
TVSDLSLAVDDLIIPWSELVLKEKIGAGSFGTVHRADWHGSDVAVKILMEQDYHLDRFKE
498

Query 541
FMREVAIMKSLRHPNIVLFMGAVTEPPNLSIVTEYLSRGSLYKLLHRSGAREVLDERRRL
600

FMREVAIMKSLRHPNIVLFMGAVTEPPNLSIVTEYLSRGSLYKLLHRSGAREVLDERRRL
Sbjct 499
FMREVAIMKSLRHPNIVLFMGAVTEPPNLSIVTEYLSRGSLYKLLHRSGAREVLDERRRL
558

Query 601
NMAFDVAKGMNYLHRRSPPIVHRDLKSPNLLVDKKYTVKVCDFGLSRLKANTYLSSKSL
A 660

NMAFDVAKGMNYLHRRSPPIVHRDLKSPNLLVDKKYTVKVCDFGLSRLKANTYLSSKSL
A
Sbjct 559
NMAFDVAKGMNYLHRRSPPIVHRDLKSPNLLVDKKYTVKVCDFGLSRLKANTYLSSKSL
A 618

Query 661

GTPEWMAPEVLRDEPSNEKSDVYSFAVILWELMTLQQPWCNLNPAQVVAAVGFKGRRLEI 720

GTPEWMAPEVLRDEPSNEKSDVYSFAVILWELMTLQQPWCNLNPAQVVAAVGFKGRRLEI
Sbjct 619 GTPEWMAPEVLRDEPSNEKSDVYSFAVILWELMTLQQPWCNLNPAQVVAAVGFKGRRLEI 678

Query 721 PKELNPQVAALIESCWANEPWRRPSFANIMETLRPLINKVPVPQLIRSDS 770
PK+LNPQVA LIESCWANEPWRRPSFANIMETLRPLINKVPVPQLIRSDS
Sbjct 679 PKDLNPQVAVLIESCWANEPWRRPSFANIMETLRPLINKVPVPQLIRSDS 728

>unnamed protein product [Triticum turgidum subsp. durum]
Sequence ID: VAI00706.1 Length: 726
Range 1: 1 to 726

Score:1467 bits(3798), Expect:0.0,
Method:Compositional matrix adjust.,
Identities:722/770(94%), Positives:724/770(94%), Gaps:44/770(5%)

Query 1 MELPAAGGGRRTSYSLLSQFPDDAAAAGASPAVLQRQSSGGSSYGAGSSVSASSDYPFHL 60
MELPAAGGGRRTSYSLLSQFP
Sbjct 1 MELPAAGGGRRTSYSLLSQFP--------------------------------------- 21

Query 61 PPAVAAAGGGGGTPSPCKSWAQQAEETYQLQLALALRLCADAACAADPGFLDPGDSGGSK 120
A GGGGGTPSPCKSWAQQAEETYQLQLALALRLCADAACAADPGFLDPGDSGGSK
Sbjct 22 -----AGGGGGGTPSPCKSWAQQAEETYQLQLALALRLCADAACAADPGFLDPGDSGGSK 76

Query 121 MGGGGGGSGSGRAFPLAPPSPTAEALSHRFWVNGSLSYSNTIPDGFYLIQGMDPFVWSMC 180

MGGGGGGSGSGRAFPLAPPSPTAEALSHRFWVNGSLSYSNTIPDGFYLIQGMDPFVWSMC

Sbjct 77
MGGGGGGSGSGRAFPLAPPSPTAEALSHRFWVNGSLSYSNTIPDGFYLIQGMDPFVWSMC
136

Query 181
TDVHEENRIPSVESLKSVRPDDSSIQVVLVDRRADFDLGMLENYASSFLSSSSDMKDVIN
240

TDVHEENRIPSVESLKSVRPDDSSIQVVLVDRRADFDLGMLENYASSFLSSSSDMKDVIN
Sbjct 137
TDVHEENRIPSVESLKSVRPDDSSIQVVLVDRRADFDLGMLENYASSFLSSSSDMKDVIN
196

Query 241
QLAKLVSSRMGGTTSNEENLLPRWKESSEAIKSSAGSIVLHLGKLPIGLCKHRSLLFKML
300

QLAKLVSSRMGGTTSNEENLLPRWKESSEAIKSSAGSIVLHLGKLPIGLCKHRSLLFKML
Sbjct 197
QLAKLVSSRMGGTTSNEENLLPRWKESSEAIKSSAGSIVLHLGKLPIGLCKHRSLLFKML
256

Query 301
ADKVNIPCRLVKGCKYCKAEDASSCVVRFGLEREYLVDLFGAPGQLSDPDSFVNGPYSLS
360

ADKVNIPCRLVKGCKYCKAEDASSCVVRFGLEREYLVDLFGAPGQLSDPDSFVNGPYSLS
Sbjct 257
ADKVNIPCRLVKGCKYCKAEDASSCVVRFGLEREYLVDLFGAPGQLSDPDSFVNGPYSLS
316

Query 361
VPSPLRPPKFRSLEITSNFSSVAKQYFSDCHSLNLLFSDASTGASNGAAVAVDQMYSKKH
420

VPSPLRPPKFRSLEITSNFSSVAKQYFSDCHSLNLLFSDASTGASNGAAVAVDQMYSKKH
Sbjct 317
VPSPLRPPKFRSLEITSNFSSVAKQYFSDCHSLNLLFSDASTGASNGAAVAVDQMYSKKH
376

Query 421
DAGDGIANSWVPVKGQAIANSDIILPEAPREVLPLMSPSNLTADKKKEFQLIEGNQYLRS
480

DAGDGIANSWVPVKGQAIANSDIILPEAPREVLPLM+PSNLTADKKKEFQLIEGNQYLRS

Sbjct 377

DAGDGIANSWVPVKGQAIANSDIILPEAPREVLPLMTPSNLTADKKKEFQLIEGNQYLRS

436

Query 481

TVSDLSLAVDDLIIPWSELVLKEKIGAGSFGTVHRADWHGSDVAVKILMEQDYHLDRFKE

540

TVSDLSLAVDDLIIPWSELVLKEKIGAGSFGTVHRADWHGSDVAVKILMEQDYHLDRFKE

Sbjct 437

TVSDLSLAVDDLIIPWSELVLKEKIGAGSFGTVHRADWHGSDVAVKILMEQDYHLDRFKE

496

Query 541

FMREVAIMKSLRHPNIVLFMGAVTEPPNLSIVTEYLSRGSLYKLLHRSGAREVLDERRRL

600

FMREVAIMKSLRHPNIVLFMGAVTEPPNLSIVTEYLSRGSLYKLLHRSGAREVLDERRRL

Sbjct 497

FMREVAIMKSLRHPNIVLFMGAVTEPPNLSIVTEYLSRGSLYKLLHRSGAREVLDERRRL

556

Query 601

NMAFDVAKGMNYLHRRSPPIVHRDLKSPNLLVDKKYTVKVCDFGLSRLKANTYLSSKSL

A 660

NMAFDVAKGMNYLHRRSPPIVHRDLKSPNLLVDKKYTVKVCDFGLSRLKANTYLSSKSL

A

Sbjct 557

NMAFDVAKGMNYLHRRSPPIVHRDLKSPNLLVDKKYTVKVCDFGLSRLKANTYLSSKSL

A 616

Query 661

GTPEWMAPEVLRDEPSNEKSDVYSFAVILWELMTLQQPWCNLNPAQVVAAVGFKGRRLEI

720

GTPEWMAPEVLRDEPSNEKSDVYSFAVILWELMTLQQPWCNLNPAQVVAAVGFKGRRLEI

Sbjct 617

GTPEWMAPEVLRDEPSNEKSDVYSFAVILWELMTLQQPWCNLNPAQVVAAVGFKGRRLEI

676

Query 721 PKELNPQVAALIESCWANEPWRRPSFANIMETLRPLINKVPVPQLIRSDS

770

PK+LNPQVA LIESCWANEPWRRPSFANIMETLRPLINKVPVPQLIRSDS
Sbjct 677 PKDLNPQVAVLIESCWANEPWRRPSFANIMETLRPLINKVPVPQLIRSDS 726

>unnamed protein product [Triticum turgidum subsp. durum]
Sequence ID: VAH96051.1 Length: 729
Range 1: 1 to 729

Score:1466 bits(3795), Expect:0.0,
Method:Compositional matrix adjust.,
Identities:724/771(94%), Positives:725/771(94%), Gaps:43/771(5%)

Query 1
MELPAAGGGRRTSYSLLSQFPDDAAAAGASPAVLQRQSSGGSSYGAGSSVSASSDYPFHL
60
MELPAAGGGRRTSYSLLSQF
Sbjct 1 MELPAAGGGRRTSYSLLSQF-- 20

Query 61
PPAVAAAGGGGGTPSPCKSWAQQAEETYQLQLALALRLCADAACAADPGFLDPGDSGGS
K 120
A A
GGGGGTPSPCKSWAQQAEETYQLQLALALRLCADAACAADPGFLDPGDSGGSK
Sbjct 21
--AAAGGGGGGGTPSPCKSWAQQAEETYQLQLALALRLCADAACAADPGFLDPGDSGGS
K 78

Query 121
MGGGGGG-SGSGRAFPLAPPSPTAEALSHRFWVNGSLSYSNTIPDGFYLIQGMDPFVWSM
179
MGGGGGG
SGSGRAFPLAPPSPTAEALSHRFWVNGSLSYSNTIPDGFYLIQGMDPFVWSM
Sbjct 79
MGGGGGGGSGSGRAFPLAPPSPTAEALSHRFWVNGSLSYSNTIPDGFYLIQGMDPFVWSM
138

Query 180
CTDVHEENRIPSVESLKSVRPDDSSIQVVLVDRRADFDLGMLENYASSFLSSSSDMKDVI
239

CTDVHEENRIPSVESLKSVRPDDSSIQVVLVDRRADFDLGMLENYASSFLSSSSDMKDVI
Sbjct 139

CTDVHEENRIPSVESLKSVRPDDSSIQVVLVDRRADFDLGMLENYASSFLSSSSDMKDVI
198

Query 240
NQLAKLVSSRMGGTTSNEENLLPRWKESSEAIKSSAGSIVLHLGKLPIGLCKHRSLLFKM
299

NQLAKLVSSRMGGTTSNEENLLPRWKESSEAIKSSAGSIVLHLGKLPIGLCKHRSLLFKM
Sbjct 199
NQLAKLVSSRMGGTTSNEENLLPRWKESSEAIKSSAGSIVLHLGKLPIGLCKHRSLLFKM
258

Query 300
LADKVNIPCRLVKGCKYCKAEDASSCVVRFGLEREYLVDLFGAPGQLSDPDSFVNGPYSL
359

LADKVNIPCRLVKGCKYCKAEDASSCVVRFGLEREYLVDLFGAPGQLSDPDSFVNGPYSL
Sbjct 259
LADKVNIPCRLVKGCKYCKAEDASSCVVRFGLEREYLVDLFGAPGQLSDPDSFVNGPYSL
318

Query 360
SVPSPLRPPKFRSLEITSNFSSVAKQYFSDCHSLNLLFSDASTGASNGAAVAVDQMYSKK
419

SVPSPLRPPKFRSLEITSNFSSVAKQYFSDCHSLNLLFSDASTGASNGAAVAVDQMYSKK
Sbjct 319
SVPSPLRPPKFRSLEITSNFSSVAKQYFSDCHSLNLLFSDASTGASNGAAVAVDQMYSKK
378

Query 420
HDAGDGIANSWVPVKGQAIANSDIILPEAPREVLPLMSPSNLTADKKKEFQLIEGNQYLR
479

HDAGDGIANSWVPVKGQAIANSDIILPEAPREVLPLM+PSNLTADKKKEFQLIEGNQYLR
Sbjct 379
HDAGDGIANSWVPVKGQAIANSDIILPEAPREVLPLMTPSNLTADKKKEFQLIEGNQYLR
438

Query 480
STVSDLSLAVDDLIIPWSELVLKEKIGAGSFGTVHRADWHGSDVAVKILMEQDYHLDRFK
539

STVSDLSLAVDDLIIPWSELVLKEKIGAGSFGTVHRADWHGSDVAVKILMEQDYHLDRFK

Sbjct 439
STVSDLSLAVDDLIIPWSELVLKEKIGAGSFGTVHRADWHGSDVAVKILMEQDYHLDRFK
498

Query 540
EFMREVAIMKSLRHPNIVLFMGAVTEPPNLSIVTEYLSRGSLYKLLHRSGAREVLDERRR
599

EFMREVAIMKSLRHPNIVLFMGAVTEPPNLSIVTEYLSRGSLYKLLHRSGAREVLDERRR
Sbjct 499
EFMREVAIMKSLRHPNIVLFMGAVTEPPNLSIVTEYLSRGSLYKLLHRSGAREVLDERRR
558

Query 600
LNMAFDVAKGMNYLHRRSPPIVHRDLKSPNLLVDKKYTVKVCDFGLSRLKANTYLSSKS
L 659

LNMAFDVAKGMNYLHRRSPPIVHRDLKSPNLLVDKKYTVKVCDFGLSRLKANTYLSSKS
L
Sbjct 559
LNMAFDVAKGMNYLHRRSPPIVHRDLKSPNLLVDKKYTVKVCDFGLSRLKANTYLSSKS
L 618

Query 660
AGTPEWMAPEVLRDEPSNEKSDVYSFAVILWELMTLQQPWCNLNPAQVVAAVGFKGRRL
E 719

AGTPEWMAPEVLRDEPSNEKSDVYSFAVILWELMTLQQPWCNLNPAQVVAAVGFKGRRL
E
Sbjct 619
AGTPEWMAPEVLRDEPSNEKSDVYSFAVILWELMTLQQPWCNLNPAQVVAAVGFKGRRL
E 678

Query 720 IPKELNPQVAALIESCWANEPWRRPSFANIMETLRPLINKVPVPQLIRSDS
770

IPKELNPQVAALIESCWANEPWRRPSFANIMETLRPLINKVPVPQLIRSDS
Sbjct 679 IPKELNPQVAALIESCWANEPWRRPSFANIMETLRPLINKVPVPQLIRSDS
729

>unnamed protein product [Triticum turgidum subsp. durum]
Sequence ID: VAI00710.1 Length: 725

Range 1: 1 to 724

Score:1435 bits(3715), Expect:0.0,
Method:Compositional matrix adjust.,
Identities:703/724(97%), Positives:709/724(97%), Gaps:2/724(0%)

Query 1
MELPAAGGGRRTSYSLLSQFPDDAAAAGASPAVLQRQSSGGSSYGAGSSVSASSDYPFHL
60
MELPAAGGGRRTSYSLLSQFPDDAA
AGASPAVLQRQSSGGSSYGAGSSVSASSDYPFHL
Sbjct 1
MELPAAGGGRRTSYSLLSQFPDDAAVAGASPAVLQRQSSGGSSYGAGSSVSASSDYPFHL
60

Query 61
PPAVAAA--GGGGGTPSPCKSWAQQAEETYQLQLALALRLCADAACAADPGFLDPGDSGG
118
PPA AA
GGGGGTPSPCKSWAQQAEETYQLQLALALRLCADAACAADPGFLDPGDSGG
Sbjct 61
PPAAAAGAGGGGGGTPSPCKSWAQQAEETYQLQLALALRLCADAACAADPGFLDPGDS
GG 120

Query 119
SKMGGGGGGSGSGRAFPLAPPSPTAEALSHRFWVNGSLSYSNTIPDGFYLIQGMDPFVWS
178

SKMGGGGGGSGSGRAFPLAPPSPTAEALSHRFWVNGSLSYSNTIPDGFYLIQGMDPFVWS
Sbjct 121
SKMGGGGGGSGSGRAFPLAPPSPTAEALSHRFWVNGSLSYSNTIPDGFYLIQGMDPFVWS
180

Query 179
MCTDVHEENRIPSVESLKSVRPDDSSIQVVLVDRRADFDLGMLENYASSFLSSSSDMKDV
238

MCTDVHEENRIPSVESLKSVRPDDSSIQVVLVDRRADFDLGMLENYASSFLSSSSDMKDV
Sbjct 181
MCTDVHEENRIPSVESLKSVRPDDSSIQVVLVDRRADFDLGMLENYASSFLSSSSDMKDV
240

Query 239
INQLAKLVSSRMGGTTSNEENLLPRWKESSEAIKSSAGSIVLHLGKLPIGLCKHRSLLFK

298

INQLAKLVSSRMGGTTSNEENLLPRWKESSEAIKSSAGSIVLHLGKLPIGLCKHRSLLFK
Sbjct 241
INQLAKLVSSRMGGTTSNEENLLPRWKESSEAIKSSAGSIVLHLGKLPIGLCKHRSLLFK
300

Query 299
MLADKVNIPCRLVKGCKYCKAEDASSCVVRFGLEREYLVDLFGAPGQLSDPDSFVNGPYS
358

MLADKVNIPCRLVKGCKYCKAEDASSCVVRFGLEREYLVDLFGAPGQLSDPDSFVNGPYS
Sbjct 301
MLADKVNIPCRLVKGCKYCKAEDASSCVVRFGLEREYLVDLFGAPGQLSDPDSFVNGPYS
360

Query 359
LSVPSPLRPPKFRSLEITSNFSSVAKQYFSDCHSLNLLFSDASTGASNGAAVAVDQMYSK
418

LSVPSPLRPPKFRSLEITSNFSSVAKQYFSDCHSLNLLFSDASTGASNGAAVAVDQMYSK
Sbjct 361
LSVPSPLRPPKFRSLEITSNFSSVAKQYFSDCHSLNLLFSDASTGASNGAAVAVDQMYSK
420

Query 419
KHDAGDGIANSWVPVKGQAIANSDIILPEAPREVLPLMSPSNLTADKKKEFQLIEGNQYL
478

KHDAGDGIANSWVPVKGQAIANSDIILPEAPREVLPLM+PSNLTADKKKEFQLIEGNQYL
Sbjct 421
KHDAGDGIANSWVPVKGQAIANSDIILPEAPREVLPLMTPSNLTADKKKEFQLIEGNQYL
480

Query 479
RSTVSDLSLAVDDLIIPWSELVLKEKIGAGSFGTVHRADWHGSDVAVKILMEQDYHLDRF
538

RSTVSDLSLAVDDLIIPWSELVLKEKIGAGSFGTVHRADWHGSDVAVKILMEQDYHLDRF
Sbjct 481
RSTVSDLSLAVDDLIIPWSELVLKEKIGAGSFGTVHRADWHGSDVAVKILMEQDYHLDRF
540

Query 539

KEFMREVAIMKSLRHPNIVLFMGAVTEPPNLSIVTEYLSRGSLYKLLHRSGAREVLDERR
598

KEFMREVAIMKSLRHPNIVLFMGAVTEPPNLSIVTEYLSRGSLYKLLHRSGAREVLDERR
Sbjct 541
KEFMREVAIMKSLRHPNIVLFMGAVTEPPNLSIVTEYLSRGSLYKLLHRSGAREVLDERR
600

Query 599
RLNMAFDVAKGMNYLHRRSPPIVHRDLKSPNLLVDKKYTVKVCDFGLSRLKANTYLSSK
S 658

RLNMAFDVAKGMNYLHRRSPPIVHRDLKSPNLLVDKKYTVKVCDFGLSRLKANTYLSSK
S
Sbjct 601
RLNMAFDVAKGMNYLHRRSPPIVHRDLKSPNLLVDKKYTVKVCDFGLSRLKANTYLSSK
S 660

Query 659
LAGTPEWMAPEVLRDEPSNEKSDVYSFAVILWELMTLQQPWCNLNPAQVVAAVGFKGRR
L 718
LAGTPEWMAPEVLRDEPSNEKSDVYSFAVILWELMTLQQPWCNLNPA+ +
+ K L
Sbjct 661
LAGTPEWMAPEVLRDEPSNEKSDVYSFAVILWELMTLQQPWCNLNPARWLLLLALKEED
L 720

Query 719 EIPK 722
+ +
Sbjct 721 KFQR 724

>uncharacterized protein LOC127304444 [Lolium perenne]
Sequence ID: XP_051191091.1 Length: 1821
Range 1: 1 to 765

Score:1354 bits(3505), Expect:0.0,
Method:Compositional matrix adjust.,
Identities:716/775(92%), Positives:738/775(95%), Gaps:15/775(1%)

Query 1
MELPAA-GGGRRTSYSLLSQFPDDAAAAGASPAVLQRQSSGGSSYGAGSSVSASSDYPF-

58

M+LPAA GGGRRTSYSLLSQFPDD AA PAVLQRQSSGGSSYG GSSVSASSDYPF

Sbjct 1 MDLPAATGGGRRTSYSLLSQFPDDPAA----PAVLQRQSSGGSSYGPGSSVSASSDYPFN 56

Query 59 -HLPPAVAAAGGGGGTPSPCKSWAQQAEETYQLQLALALRLCADAACAADPGFLDPGDSG 117

HLPP+ A A GGTP KSWAQQAEETYQLQLALALRLCADAACAADPGFLDPGD+

Sbjct 57 HHLPPSAAPAAAAGGTP---KSWAQQAEETYQLQLALALRLCADAACAADPGFLDPGDA- 112

Query 118 GSKM--GGGGGGSGSGRAFPLAPPSPTAEALSHRFWVNGSLSYSNTIPDGFYLIQGMDPF 175

KM G G GGSGSGRAFPLAPP+PTAEALSHRFWVNGSLSYSNTIPDGFYLIQGMDPF

Sbjct 113 --KMMGGPGPGGSGSGRAFPLAPPTPTAEALSHRFWVNGSLSYSNTIPDGFYLIQGMDPF 170

Query 176 VWSMCTDVHEENRIPSVESLKSVRPDDSSIQVVLVDRRADFDLGMLENYASSFLSSSSDM 235

VWSMCTDVHEENRIPSVESL+SVRP DSSIQVVLVDRRADFDLGMLENYA+SFLSSS+D+

Sbjct 171 VWSMCTDVHEENRIPSVESLRSVRPGDSSIQVVLVDRRADFDLGMLENYAASFLSSSADI 230

Query 236 KDVINQLAKLVSSRMGGTTSNEENLLPRWKESSEAIKSSAGSIVLHLGKLPIGLCKHRSL 295

KDVINQLAKLVSSRMGGT SNEENLLPRWKESSEAI SS+GSIVLHLGKLPIGLCK RSL

Sbjct 231 KDVINQLAKLVSSRMGGTASNEENLLPRWKESSEAITSSSGSIVLHLGKLPIGLCKQRSL 290

Query 296 LFKMLADKVNIPCRLVKGCKYCKAEDASSCVVRFGLEREYLVDLFGAPGQLSDPDSFVN

G 355

LFKMLADKVN+PCRLVKGCKYCKAEDASSCVVRFGLEREYLVDL G PGQLSDPDSFVNG

Sbjct 291 LFKMLADKVNVPCRLVKGCKYCKAEDASSCVVRFGLEREYLVDLIGEPGQLSDPDSFVNG 350

Query 356 PYSLSVPSPLRPPKFRSLEITSNFSSVAKQYFSDCHSLNLLFSDASTGASNGAAVAVDQM 415

PYSLSVPSPLRPPKFRSLEITSNFSSVAKQYFSDCHSLNLLFSDASTGAS+GAAVAVDQM

Sbjct 351 PYSLSVPSPLRPPKFRSLEITSNFSSVAKQYFSDCHSLNLLFSDASTGASSGAAVAVDQM 410

Query 416 YSKKHDAGDGIANSWVPVKGQAIANSDIILPEAPREVLPLMSPSNLTADKKKEFQLIEGN 475

YSKKHDAGDGIA+ WVPVKGQ NSDIILPEAPRE+LPLM+ SNLTADKKKEFQL+EGN

Sbjct 411 YSKKHDAGDGIASGWVPVKGQPTINSDIILPEAPREILPLMTSSNLTADKKKEFQLLEGN 470

Query 476 QYLRSTVSDLSLAVDDLIIPWSELVLKEKIGAGSFGTVHRADWHGSDVAVKILMEQDYHL 535

QYLRSTVSDLSLAVDDLIIPW+ELVLKEKIGAGSFGTVHRADWHGSDVAVKILMEQDYHL

Sbjct 471 QYLRSTVSDLSLAVDDLIIPWNELVLKEKIGAGSFGTVHRADWHGSDVAVKILMEQDYHL 530

Query 536 DRFKEFMREVAIMKSLRHPNIVLFMGAVTEPPNLSIVTEYLSRGSLYKLLHRSGAREVLD 595

DRF+EFMREVAIMKSLRHPNIVLFMGAVTEPPNLSIVTEYLSRGSLYKLLHR+GAREVLD

Sbjct 531 DRFREFMREVAIMKSLRHPNIVLFMGAVTEPPNLSIVTEYLSRGSLYKLLHRTGAREVLD 590

Query 596

ERRRLNMAFDVAKGMNYLHRRSPPIVHRDLKSPNLLVDKKYTVKVCDFGLSRLKANTYL
S 655

ERRRLNMAFDVAKGMNYLHRRSPPIVHRDLKSPNLLVDKKYTVKVCDFGLSRLKANTYL
S
Sbjct 591
ERRRLNMAFDVAKGMNYLHRRSPPIVHRDLKSPNLLVDKKYTVKVCDFGLSRLKANTYL
S 650

Query 656
SKSLAGTPEWMAPEVLRDEPSNEKSDVYSFAVILWELMTLQQPWCNLNPAQVVAAVGFK
G 715

SKSLAGTPEWMAPEVLRDEPSNEKSDVYSFAVILWELMTLQQPWCNLNPAQVVAAVGFK
G
Sbjct 651
SKSLAGTPEWMAPEVLRDEPSNEKSDVYSFAVILWELMTLQQPWCNLNPAQVVAAVGFK
G 710

Query 716
RRLEIPKELNPQVAALIESCWANEPWRRPSFANIMETLRPLINKVPVPQLIRSDS 770
RRLEIP +LNPQVAALIE+CWANEPW+RPSFANIMETLRPLINKV
VPQL+RSDS
Sbjct 711
RRLEIPIDLNPQVAALIEACWANEPWKRPSFANIMETLRPLINKVAVPQLLRSDS 765

>uncharacterized protein LOC124657634 [Lolium rigidum]
Sequence ID: XP_047052109.1 Length: 1797
Range 1: 1 to 767

Score:1350 bits(3495), Expect:0.0,
Method:Compositional matrix adjust.,
Identities:713/774(92%), Positives:731/774(94%), Gaps:11/774(1%)

Query 1
MELPAA-GGGRRTSYSLLSQFPDDAAAAGASPAVLQRQSSGGSSYGAGSSVSASSDYPF-
58
M+LPAA GGGRRTSYSLLSQFPDD AA PAVLQRQSSGGSSYG GSSVS
SSDYPF
Sbjct 1
MDLPAATGGGRRTSYSLLSQFPDDPAA----PAVLQRQSSGGSSYGPGSSVSGSSDYPFN 56

Query 59
-HLPPAVAAAGGG-GGTPSPCKSWAQQAEETYQLQLALALRLCADAACAADPGFLDPGDS
116
HLPPA AA GGTP
KSWAQQAEETYQLQLALALRLCADAACAADPGFLDPGD
Sbjct 57
HHLPPASAAPAAASGGTP---KSWAQQAEETYQLQLALALRLCADAACAADPGFLDPGDG
113

Query 117
GGSKMGGGGGGSGSGRAFPLAPPSPTAEALSHRFWVNGSLSYSNTIPDGFYLIQGMDPFV
176
G GG
SGRAFPLAPP+PTAEALSHRFWVNGSLSYSNTIPDGFYLIQGMDPFV
Sbjct 114
KMMGGPGPGGSGSSGRAFPLAPPTPTAEALSHRFWVNGSLSYSNTIPDGFYLIQGMDPFV
173

Query 177
WSMCTDVHEENRIPSVESLKSVRPDDSSIQVVLVDRRADFDLGMLENYASSFLSSSSDMK
236
WSMCTDVHEENRIPSVESL+SVRP
DSSIQVVLVDRRADFDLGMLENYA+SFLSSS+D+K
Sbjct 174
WSMCTDVHEENRIPSVESLRSVRPGDSSIQVVLVDRRADFDLGMLENYAASFLSSSADIK
233

Query 237
DVINQLAKLVSSRMGGTTSNEENLLPRWKESSEAIKSSAGSIVLHLGKLPIGLCKHRSLL
296
DVINQLAKLVSSRMGGT SNEENLLPRWKESSEAI
SS+GSIVLHLGKLPIGLCK RSLL
Sbjct 234
DVINQLAKLVSSRMGGTASNEENLLPRWKESSEAITSSSGSIVLHLGKLPIGLCKQRSLL
293

Query 297
FKMLADKVNIPCRLVKGCKYCKAEDASSCVVRFGLEREYLVDLFGAPGQLSDPDSFVNGP
356
FKMLADKVN+PCRLVKGCKYCKAEDASSCVVRFGLEREYLVDL G
PGQLSDPDSFVNGP
Sbjct 294
FKMLADKVNVPCRLVKGCKYCKAEDASSCVVRFGLEREYLVDLIGEPGQLSDPDSFVNG

P 353

Query 357
YSLSVPSPLRPPKFRSLEITSNFSSVAKQYFSDCHSLNLLFSDASTGASNGAAVAVDQMY
416

YSLSVPSPLRPPKFRSLEITSNFSSVAKQYFSDCHSLNLLFSDASTGAS+GAAVAVDQMY
Sbjct 354
YSLSVPSPLRPPKFRSLEITSNFSSVAKQYFSDCHSLNLLFSDASTGASSGAAVAVDQMY
413

Query 417
SKKHDAGDGIANSWVPVKGQAIANSDIILPEAPREVLPLMSPSNLTADKKKEFQLIEGNQ
476
SKKHDAGDGIA+ WVPVKGQ NSDIILPEAPRE+LPLM+
SNLTADKKKEFQL+EGNQ
Sbjct 414
SKKHDAGDGIASGWVPVKGQPTINSDIILPEAPREILPLMTSSNLTADKKKEFQLLEGNQ
473

Query 477
YLRSTVSDLSLAVDDLIIPWSELVLKEKIGAGSFGTVHRADWHGSDVAVKILMEQDYHLD
536

YLRSTVSDLSLAVDDLIIPWSELVLKEKIGAGSFGTVHRADWHGSDVAVKILMEQDYHLD
Sbjct 474
YLRSTVSDLSLAVDDLIIPWSELVLKEKIGAGSFGTVHRADWHGSDVAVKILMEQDYHLD
533

Query 537
RFKEFMREVAIMKSLRHPNIVLFMGAVTEPPNLSIVTEYLSRGSLYKLLHRSGAREVLDE
596

RF+EFMREVAIMKSLRHPNIVLFMGAVTEPPNLSIVTEYLSRGSLYKLLHR+GAREVLDE
Sbjct 534
RFREFMREVAIMKSLRHPNIVLFMGAVTEPPNLSIVTEYLSRGSLYKLLHRTGAREVLDE
593

Query 597
RRRLNMAFDVAKGMNYLHRRSPPIVHRDLKSPNLLVDKKYTVKVCDFGLSRLKANTYLS
S 656

RRRLNMAFDVAKGMNYLHRRSPPIVHRDLKSPNLLVDKKYTVKVCDFGLSRLKANTYLS
S

Sbjct 594
RRRLNMAFDVAKGMNYLHRRSPPIVHRDLKSPNLLVDKKYTVKVCDFGLSRLKANTYLS
S 653

Query 657
KSLAGTPEWMAPEVLRDEPSNEKSDVYSFAVILWELMTLQQPWCNLNPAQVVAAVGFKG
R 716

KSLAGTPEWMAPEVLRDEPSNEKSDVYSFAVILWELMTLQQPWCNLNPAQVVAAVGFKG
R
Sbjct 654
KSLAGTPEWMAPEVLRDEPSNEKSDVYSFAVILWELMTLQQPWCNLNPAQVVAAVGFKG
R 713

Query 717
RLEIPKELNPQVAALIESCWANEPWRRPSFANIMETLRPLINKVPVPQLIRSDS 770
RLEIP +LNPQVAALIESCWANEPW+RPSFANIMETLRPLINKV VPQL+RSDS
Sbjct 714 RLEIPIDLNPQVAALIESCWANEPWKRPSFANIMETLRPLINKVAVPQLLRSDS
767

>serine/threonine-protein kinase CTR1-like [Lolium rigidum]
Sequence ID: XP_047052104.1 Length: 763
Range 1: 1 to 763

Score:1345 bits(3481), Expect:0.0,
Method:Compositional matrix adjust.,
Identities:706/771(92%), Positives:725/771(94%), Gaps:9/771(1%)

Query 1
MELPAA-GGGRRTSYSLLSQFPDDAAAAGASPAVLQRQSSGGSSYGAGSSVSASSDYPFH
59
M+LPAA GGGRRTSYSLLSQFPDD AA PAVLQRQSSGGSSYG GSSVS
SSDYPF+
Sbjct 1
MDLPAATGGGRRTSYSLLSQFPDDPAA----PAVLQRQSSGGSSYGPGSSVSGSSDYPFN 56

Query 60
LPPAVAAAGGGGGTPSPCKSWAQQAEETYQLQLALALRLCADAACAADPGFLDPGDSGG
S 119
AAA GGTP
KSWAQQAEETYQLQLALALRLCADAACAADPGFLDPGD

Sbjct 57
HHLPPAAAPAAGGTP---KSWAQQAEETYQLQLALALRLCADAACAADPGFLDPGDGKM
M 113

Query 120
KMGGGGGGSGSGRAFPLAPPSPTAEALSHRFWVNGSLSYSNTIPDGFYLIQGMDPFVWSM
179
G GG
RAFPLAPP+PTAEALSHRFWVNGSLSYSNTIPDGFYLIQGMDPFVWSM
Sbjct 114
GGPGAGGSGSG-RAFPLAPPTPTAEALSHRFWVNGSLSYSNTIPDGFYLIQGMDPFVWSM
172

Query 180
CTDVHEENRIPSVESLKSVRPDDSSIQVVLVDRRADFDLGMLENYASSFLSSSSDMKDVI
239
CTDVHEENRIPSVESL+SVRP
DSSIQVVLVDRRADFDLGMLENYA+SFLSSS+D+KDVI
Sbjct 173
CTDVHEENRIPSVESLRSVRPGDSSIQVVLVDRRADFDLGMLENYAASFLSSSADIKDVI
232

Query 240
NQLAKLVSSRMGGTTSNEENLLPRWKESSEAIKSSAGSIVLHLGKLPIGLCKHRSLLFKM
299
NQLAKLVSSRMGGT SNEENLLPRWKESSE I SS+GSIVLHLGKLPIGLCK
RSLLFKM
Sbjct 233
NQLAKLVSSRMGGTASNEENLLPRWKESSEVITSSSGSIVLHLGKLPIGLCKQRSLLFKM
292

Query 300
LADKVNIPCRLVKGCKYCKAEDASSCVVRFGLEREYLVDLFGAPGQLSDPDSFVNGPYSL
359
LADKVN+PCRLVKGCKYCKAEDASSCVVRFGLEREYLVDL G
PGQLSDPDSFVNGPYSL
Sbjct 293
LADKVNVPCRLVKGCKYCKAEDASSCVVRFGLEREYLVDLIGEPGQLSDPDSFVNGPYSL
352

Query 360
SVPSPLRPPKFRSLEITSNFSSVAKQYFSDCHSLNLLFSDASTGASNGAAVAVDQMYSKK
419

SVPSPLRPPKFRSLEITSNFSSVAKQYFSDCHSLNLLFSDASTGAS+GAAVAVDQMYSKK
Sbjct 353
SVPSPLRPPKFRSLEITSNFSSVAKQYFSDCHSLNLLFSDASTGASSGAAVAVDQMYSKK
412

Query 420
HDAGDGIANSWVPVKGQAIANSDIILPEAPREVLPLMSPSNLTADKKKEFQLIEGNQYLR
479
HDAGDGIA+ WVPVKGQ NSDIILPEAPRE+LPLM+
SNLTADKKKEFQL+EGNQYLR
Sbjct 413
HDAGDGIASGWVPVKGQPTINSDIILPEAPREILPLMTSSNLTADKKKEFQLLEGNQYLR
472

Query 480
STVSDLSLAVDDLIIPWSELVLKEKIGAGSFGTVHRADWHGSDVAVKILMEQDYHLDRFK
539

STVSDLSLAVDDLIIPWSELVLKEKIGAGSFGTVHRADWHGSDVAVKILMEQDYHLDRF+
Sbjct 473
STVSDLSLAVDDLIIPWSELVLKEKIGAGSFGTVHRADWHGSDVAVKILMEQDYHLDRFR
532

Query 540
EFMREVAIMKSLRHPNIVLFMGAVTEPPNLSIVTEYLSRGSLYKLLHRSGAREVLDERRR
599

EFMREVAIMKSLRHPNIVLFMGAVTEPPNLSIVTEYLSRGSLYKLLHR+GAREVLDERRR
Sbjct 533
EFMREVAIMKSLRHPNIVLFMGAVTEPPNLSIVTEYLSRGSLYKLLHRTGAREVLDERRR
592

Query 600
LNMAFDVAKGMNYLHRRSPPIVHRDLKSPNLLVDKKYTVKVCDFGLSRLKANTYLSSKS
L 659

LNMAFDVAKGMNYLHRRSPPIVHRDLKSPNLLVDKKYTVKVCDFGLSRLKANTYLSSKS
L
Sbjct 593
LNMAFDVAKGMNYLHRRSPPIVHRDLKSPNLLVDKKYTVKVCDFGLSRLKANTYLSSKS
L 652

Query 660
AGTPEWMAPEVLRDEPSNEKSDVYSFAVILWELMTLQQPWCNLNPAQVVAAVGFKGRRL

E 719

AGTPEWMAPEVLRDEPSNEKSDVYSFAVILWELMTLQQPWCNLNPAQVVAAVGFKGRRL
E
Sbjct 653
AGTPEWMAPEVLRDEPSNEKSDVYSFAVILWELMTLQQPWCNLNPAQVVAAVGFKGRRL
E 712

Query 720 IPKELNPQVAALIESCWANEPWRRPSFANIMETLRPLINKVPVPQLIRSDS
770
IP +LNPQVAALIESCWANEPW+RPSFANIMETLRPLINKV VPQL+RSDS
Sbjct 713 IPIDLNPQVAALIESCWANEPWKRPSFANIMETLRPLINKVAVPQLLRSDS
763

>hypothetical protein QYE76_006900 [Lolium multiflorum]
Sequence ID: KAK1632585.1 Length: 768
Range 1: 1 to 768

Score:1342 bits(3474), Expect:0.0,
Method:Compositional matrix adjust.,
Identities:714/775(92%), Positives:734/775(94%), Gaps:12/775(1%)

Query 1
MELPAA-GGGRRTSYSLLSQFPDDAAAAGASPAVLQRQSSGGSSYGAGSSVSASSDYPF-
58
M+LPAA GGGRRTSYSLLSQFPDD AA PA LQRQSSGGSSYG
GSS+SASSDYPF
Sbjct 1
MDLPAATGGGRRTSYSLLSQFPDDPAA----PAGLQRQSSGGSSYGPGSSLSASSDYPFN 56

Query 59
-HLPPAVAAAGGGGGTPSPCKSWAQQAEETYQLQLALALRLCADAACAADPGFLDPGDS
G 117
HLPPA AA G
KSWAQQAEETYQLQLALALRLCADAACAADPGFLDPGD+
Sbjct 57
HHLPPAAAAGPGSAAAGGTPKSWAQQAEETYQLQLALALRLCADAACAADPGFLDPGD
A- 115

Query 118
GSKM--GGGGGGSGSGRAFPLAPPSPTAEALSHRFWVNGSLSYSNTIPDGFYLIQGMDPF

175
KM GGG
GGSGSGRAFPLAPP+PTAEALSHRFWVNGSLSYSNTIPDGFYLIQGMDPF
Sbjct 116
--KMMGGGGPGGSGSGRAFPLAPPTPTAEALSHRFWVNGSLSYSNTIPDGFYLIQGMDPF
173

Query 176
VWSMCTDVHEENRIPSVESLKSVRPDDSSIQVVLVDRRADFDLGMLENYASSFLSSSSDM
235
VWSMCTDVHEENRIPSVESL+SVRP
DSSIQVVLVDRRADFDLGMLENYASSFLSSS+D+
Sbjct 174
VWSMCTDVHEENRIPSVESLRSVRPGDSSIQVVLVDRRADFDLGMLENYASSFLSSSADI
233

Query 236
KDVINQLAKLVSSRMGGTTSNEENLLPRWKESSEAIKSSAGSIVLHLGKLPIGLCKHRSL
295
KDVINQLAKLVSSRMGGT SNEENLLPRWKESSEAI
SS+GSIVLHLGKLPIGLCK RSL
Sbjct 234
KDVINQLAKLVSSRMGGTASNEENLLPRWKESSEAITSSSGSIVLHLGKLPIGLCKQRSL
293

Query 296
LFKMLADKVNIPCRLVKGCKYCKAEDASSCVVRFGLEREYLVDLFGAPGQLSDPDSFVN
G 355
LFKMLADKVN+PCRLVKGCKYCKAEDASSCVVRFGLEREYLVDL G
PGQLSDPDSFVNG
Sbjct 294
LFKMLADKVNVPCRLVKGCKYCKAEDASSCVVRFGLEREYLVDLIGEPGQLSDPDSFVN
G 353

Query 356
PYSLSVPSPLRPPKFRSLEITSNFSSVAKQYFSDCHSLNLLFSDASTGASNGAAVAVDQM
415
PYSLSVPSPLRPPKFRSLEITSNFSSVAKQYFSDCHSLNLLFSDASTGAS
GAAVAVDQM
Sbjct 354
PYSLSVPSPLRPPKFRSLEITSNFSSVAKQYFSDCHSLNLLFSDASTGASRGAAVAVDQM
413

Query 416

YSKKHDAGDGIANSWVPVKGQAIANSDIILPEAPREVLPLMSPSNLTADKKKEFQLIEGN 475

YSKKHDAGDGIA+ WVPVKGQ NS+IILPEAPRE+LPLM+ SNLTADKKKEFQL+EGN

Sbjct 414 YSKKHDAGDGIASGWVPVKGQPTINSEIILPEAPREILPLMTSSNLTADKKKEFQLLEGN 473

Query 476 QYLRSTVSDLSLAVDDLIIPWSELVLKEKIGAGSFGTVHRADWHGSDVAVKILMEQDYHL 535

QYLRSTVSDLSLAVDDLIIPW+ELVLKEKIGAGSFGTVHRADWHGSDVAVKILMEQDYHL

Sbjct 474 QYLRSTVSDLSLAVDDLIIPWNELVLKEKIGAGSFGTVHRADWHGSDVAVKILMEQDYHL 533

Query 536 DRFKEFMREVAIMKSLRHPNIVLFMGAVTEPPNLSIVTEYLSRGSLYKLLHRSGAREVLD 595

DRF+EFMREVAIMKSLRHPNIVLFMGAVTEPPNLSIVTEYLSRGSLYKLLHR+GAREVLD

Sbjct 534 DRFREFMREVAIMKSLRHPNIVLFMGAVTEPPNLSIVTEYLSRGSLYKLLHRTGAREVLD 593

Query 596 ERRRLNMAFDVAKGMNYLHRRSPPIVHRDLKSPNLLVDKKYTVKVCDFGLSRLKANTYLS 655

ERRRLNMAFDVAKGMNYLHRRSPPIVHRDLKSPNLLVDKKYTVKVCDFGLSRLKANTYLS

Sbjct 594 ERRRLNMAFDVAKGMNYLHRRSPPIVHRDLKSPNLLVDKKYTVKVCDFGLSRLKANTYLS 653

Query 656 SKSLAGTPEWMAPEVLRDEPSNEKSDVYSFAVILWELMTLQQPWCNLNPAQVVAAVGFKG 715

SKSLAGTPEWMAPEVLRDEPSNEKSDVYSFAVILWELMTLQQPWCNLNPAQVVAAVGFKG

Sbjct 654 SKSLAGTPEWMAPEVLRDEPSNEKSDVYSFAVILWELMTLQQPWCNLNPAQVVAAVGFK

G 713

Query 716
RRLEIPKELNPQVAALIESCWANEPWRRPSFANIMETLRPLINKVPVPQLIRSDS 770
RRLEIP +LNPQVAALIESCWANEPW+RPSFANIMETLRPLINKV
VPQL+RSDS
Sbjct 714
RRLEIPIDLNPQVAALIESCWANEPWKRPSFANIMETLRPLINKVAVPQLLRSDS 768

>uncharacterized protein LOC100825661 [Brachypodium distachyon]
Sequence ID: XP_014757903.1 Length: 1820
Range 1: 1 to 767

Score:1328 bits(3437), Expect:0.0,
Method:Compositional matrix adjust.,
Identities:705/776(91%), Positives:728/776(93%), Gaps:15/776(1%)

Query 1
MELPAAGGGRRTSYSLLSQFPDDAAAAGASPA--VLQRQSSGGSSYGAGSSVSASSDYPF
58
M+LPAAGG RRTSYSLLSQFPDDA+A+ A+
VLQRQSSGGSSYGAGSS+SASSDYPF
Sbjct 1
MDLPAAGG-RRTSYSLLSQFPDDASASSAAATAAVLQRQSSGGSSYGAGSSISASSDYPF
59

Query 59
HLPPAVAAAGGGGGTP----SPCKSWAQQAEETYQLQLALALRLCADAACAADPGFLDPG
114
HLPP A
SPCKSWAQQAEETYQLQLALALRLCADAACAADPGFLDPG
Sbjct 60
HLPPPAAGPAAAAAATGGTPSPCKSWAQQAEETYQLQLALALRLCADAACAADPGFLDP
G 119

Query 115
DSGGSKMGGGGGGSGSGRAFPLAPPSPTAEALSHRFWVNGSLSYSNTIPDGFYLIQGMDP
174
DSGG+ G RAFPLAPP+PTAEALSHRFWVNGSLSYSNTIPDGFYLI
GMDP
Sbjct 120

DSGGNNNSGS---GSGRRAFPLAPPTPTAEALSHRFWVNGSLSYSNTIPDGFYLIHGMDP
176

Query 175
FVWSMCTDVHEENRIPSVESLKSVRPDDSSIQVVLVDRRADFDLGMLENYASSFLSSSSD
234
FVWS+CTDVHEENRIPS+ESLKSV
PDDSSIQVVL+DRRADFDLGMLENYASSFLSSS+D
Sbjct 177
FVWSLCTDVHEENRIPSMESLKSVCPDDSSIQVVLIDRRADFDLGMLENYASSFLSSSAD
236

Query 235
MKDVINQLAKLVSSRMGGTTSNEENLLPRWKESSEAIKSSAGSIVLHLGKLPIGLCKHRS
294

MKDVINQLAKLVSSRMGGTTSNEENLLPRWKESSEAIKSSAGSIVLHLGKLPIGLCKHRS
Sbjct 237
MKDVINQLAKLVSSRMGGTTSNEENLLPRWKESSEAIKSSAGSIVLHLGKLPIGLCKHRS
296

Query 295
LLFKMLADKVNIPCRLVKGCKYCKAEDASSCVVRFGLEREYLVDLFGAPGQLSDPDSFVN
354
LLFKMLADKVN+PCRLVKGCKYCKA+DASSCVVRFGLEREYLVDL G
PGQLSDPDSFVN
Sbjct 297
LLFKMLADKVNVPCRLVKGCKYCKADDASSCVVRFGLEREYLVDLIGDPGQLSDPDSFV
N 356

Query 355
GPYSLSVPSPLRPPKFRSLEITSNFSSVAKQYFSDCHSLNLLFSDASTGASNGAAVAVDQ
414

GPYSLSVPSPLRPPKFRSLEITSNFSSVAKQYFSDCHSLNLLFSDASTGAS+GAAVAVDQ
Sbjct 357
GPYSLSVPSPLRPPKFRSLEITSNFSSVAKQYFSDCHSLNLLFSDASTGASSGAAVAVDQ
416

Query 415
MYSKKHDAGDGIANSWVPVKGQAIANSDIILPEAPREVLPLMSPSNLTADKKKEFQLIEG
474
MYSKKHDA +W+PVKGQA N DIILPEAPREVLPLMS +NL
ADKKKE+QLIEG

Sbjct 417
MYSKKHDA----VGAWMPVKGQATTNPDIILPEAPREVLPLMS-ANLAADKKKEYQLIEG
471

Query 475
NQYLRSTVSDLSLAVDDLIIPWSELVLKEKIGAGSFGTVHRADWHGSDVAVKILMEQDYH
534

NQYLRSTVSDLSLAVDDLIIPW+ELVLKEKIGAGSFGTVHRADWHGSDVAVKILMEQDYH
Sbjct 472
NQYLRSTVSDLSLAVDDLIIPWNELVLKEKIGAGSFGTVHRADWHGSDVAVKILMEQDYH
531

Query 535
LDRFKEFMREVAIMKSLRHPNIVLFMGAVTEPPNLSIVTEYLSRGSLYKLLHRSGAREVL
594

LDRF+EFMREVAIMKSLRHPNIVLFMGAVTEPPNLSIVTEYLSRGSLYKLLHRSGAREVL
Sbjct 532
LDRFREFMREVAIMKSLRHPNIVLFMGAVTEPPNLSIVTEYLSRGSLYKLLHRSGAREVL
591

Query 595
DERRRLNMAFDVAKGMNYLHRRSPPIVHRDLKSPNLLVDKKYTVKVCDFGLSRLKANTY
L 654

DERRRLNMAFDVAKGMNYLHRRSPPIVHRDLKSPNLLVDKKYTVKVCDFGLSRLKANT+
L
Sbjct 592
DERRRLNMAFDVAKGMNYLHRRSPPIVHRDLKSPNLLVDKKYTVKVCDFGLSRLKANTF
L 651

Query 655
SSKSLAGTPEWMAPEVLRDEPSNEKSDVYSFAVILWELMTLQQPWCNLNPAQVVAAVGF
K 714

SSKSLAGTPEWMAPEVLRDEPSNEKSDVYSFAVILWELMTLQQPWCNLNPAQVVAAVGF
K
Sbjct 652
SSKSLAGTPEWMAPEVLRDEPSNEKSDVYSFAVILWELMTLQQPWCNLNPAQVVAAVGF
K 711

Query 715
GRRLEIPKELNPQVAALIESCWANEPWRRPSFANIMETLRPLINKVPVPQLIRSDS 770

GRRLEIPK+LNPQVAALIESCWANEPWRRPSFANIMETLRPLINKV VPQL+RSDS
Sbjct 712
GRRLEIPKDLNPQVAALIESCWANEPWRRPSFANIMETLRPLINKVSVPQLLRSDS 767

>hypothetical protein BRADI_4g38400v3 [Brachypodium distachyon]
Sequence ID: KQJ91573.1 Length: 767
Range 1: 1 to 767

Score:1321 bits(3419), Expect:0.0,
Method:Compositional matrix adjust.,
Identities:705/776(91%), Positives:728/776(93%), Gaps:15/776(1%)

Query 1
MELPAAGGGRRTSYSLLSQFPDDAAAAGASPA--VLQRQSSGGSSYGAGSSVSASSDYPF 58
M+LPAAGG RRTSYSLLSQFPDDA+A+ A+ VLQRQSSGGSSYGAGSS+SASSDYPF
Sbjct 1
MDLPAAGG-RRTSYSLLSQFPDDASASSAAATAAVLQRQSSGGSSYGAGSSISASSDYPF 59

Query 59
HLPPAVAAAGGGGGTP----SPCKSWAQQAEETYQLQLALALRLCADAACAADPGFLDPG 114
HLPP A SPCKSWAQQAEETYQLQLALALRLCADAACAADPGFLDPG
Sbjct 60
HLPPPAAGPAAAAAATGGTPSPCKSWAQQAEETYQLQLALALRLCADAACAADPGFLDPG 119

Query 115
DSGGSKMGGGGGGSGSGRAFPLAPPSPTAEALSHRFWVNGSLSYSNTIPDGFYLIQGMDP 174
DSGG+ G RAFPLAPP+PTAEALSHRFWVNGSLSYSNTIPDGFYLI GMDP
Sbjct 120
DSGGNNNSGS---GSGRRAFPLAPPTPTAEALSHRFWVNGSLSYSNTIPDGFYLIHGMDP 176

Query 175

FVWSMCTDVHEENRIPSVESLKSVRPDDSSIQVVLVDRRADFDLGMLENYASSFLSSSSD 234
FVWS+CTDVHEENRIPS+ESLKSV PDDSSIQVVL+DRRADFDLGMLENYASSFLSSS+D
Sbjct 177 FVWSLCTDVHEENRIPSMESLKSVCPDDSSIQVVLIDRRADFDLGMLENYASSFLSSSAD 236

Query 235 MKDVINQLAKLVSSRMGGTTSNEENLLPRWKESSEAIKSSAGSIVLHLGKLPIGLCKHRS 294

MKDVINQLAKLVSSRMGGTTSNEENLLPRWKESSEAIKSSAGSIVLHLGKLPIGLCKHRS
Sbjct 237 MKDVINQLAKLVSSRMGGTTSNEENLLPRWKESSEAIKSSAGSIVLHLGKLPIGLCKHRS 296

Query 295 LLFKMLADKVNIPCRLVKGCKYCKAEDASSCVVRFGLEREYLVDLFGAPGQLSDPDSFVN 354
LLFKMLADKVN+PCRLVKGCKYCKA+DASSCVVRFGLEREYLVDL G PGQLSDPDSFVN
Sbjct 297 LLFKMLADKVNVPCRLVKGCKYCKADDASSCVVRFGLEREYLVDLIGDPGQLSDPDSFVN 356

Query 355 GPYSLSVPSPLRPPKFRSLEITSNFSSVAKQYFSDCHSLNLLFSDASTGASNGAAVAVDQ 414

GPYSLSVPSPLRPPKFRSLEITSNFSSVAKQYFSDCHSLNLLFSDASTGAS+GAAVAVDQ
Sbjct 357 GPYSLSVPSPLRPPKFRSLEITSNFSSVAKQYFSDCHSLNLLFSDASTGASSGAAVAVDQ 416

Query 415 MYSKKHDAGDGIANSWVPVKGQAIANSDIILPEAPREVLPLMSPSNLTADKKKEFQLIEG 474
MYSKKHDA +W+PVKGQA N DIILPEAPREVLPLMS +NL ADKKKE+QLIEG
Sbjct 417 MYSKKHDA----VGAWMPVKGQATTNPDIILPEAPREVLPLMS-ANLAADKKKEYQLIEG 471

Query 475
NQYLRSTVSDLSLAVDDLIIPWSELVLKEKIGAGSFGTVHRADWHGSDVAVKILMEQDYH
534

NQYLRSTVSDLSLAVDDLIIPW+ELVLKEKIGAGSFGTVHRADWHGSDVAVKILMEQDYH
Sbjct 472
NQYLRSTVSDLSLAVDDLIIPWNELVLKEKIGAGSFGTVHRADWHGSDVAVKILMEQDYH
531

Query 535
LDRFKEFMREVAIMKSLRHPNIVLFMGAVTEPPNLSIVTEYLSRGSLYKLLHRSGAREVL
594

LDRF+EFMREVAIMKSLRHPNIVLFMGAVTEPPNLSIVTEYLSRGSLYKLLHRSGAREVL
Sbjct 532
LDRFREFMREVAIMKSLRHPNIVLFMGAVTEPPNLSIVTEYLSRGSLYKLLHRSGAREVL
591

Query 595
DERRRLNMAFDVAKGMNYLHRRSPPIVHRDLKSPNLLVDKKYTVKVCDFGLSRLKANTY
L 654

DERRRLNMAFDVAKGMNYLHRRSPPIVHRDLKSPNLLVDKKYTVKVCDFGLSRLKANT+
L
Sbjct 592
DERRRLNMAFDVAKGMNYLHRRSPPIVHRDLKSPNLLVDKKYTVKVCDFGLSRLKANTF
L 651

Query 655
SSKSLAGTPEWMAPEVLRDEPSNEKSDVYSFAVILWELMTLQQPWCNLNPAQVVAAVGF
K 714

SSKSLAGTPEWMAPEVLRDEPSNEKSDVYSFAVILWELMTLQQPWCNLNPAQVVAAVGF
K
Sbjct 652
SSKSLAGTPEWMAPEVLRDEPSNEKSDVYSFAVILWELMTLQQPWCNLNPAQVVAAVGF
K 711

Query 715
GRRLEIPKELNPQVAALIESCWANEPWRRPSFANIMETLRPLINKVPVPQLIRSDS 770
GRRLEIPK+LNPQVAALIESCWANEPWRRPSFANIMETLRPLINKV
VPQL+RSDS
Sbjct 712
GRRLEIPKDLNPQVAALIESCWANEPWRRPSFANIMETLRPLINKVSVPQLLRSDS 767

>hypothetical protein E2562_011176 [Oryza meyeriana var. granulata]
Sequence ID: KAF0911546.1 Length: 752
>hypothetical protein E2562_011176 [Oryza meyeriana var. granulata]
Sequence ID: KAF0911547.1 Length: 752
>hypothetical protein E2562_011176 [Oryza meyeriana var. granulata]
Sequence ID: KAF0911548.1 Length: 752
Range 1: 1 to 752

Score:1283 bits(3319), Expect:0.0,
Method:Compositional matrix adjust.,
Identities:661/770(86%), Positives:709/770(92%), Gaps:18/770(2%)

Query 1
MELPAAGGGRRTSYSLLSQFPDDAAAAGASPAVLQRQSSGGSSYGAGSSVSASSDYPFHL
60
M+LPA G R T+YSLLSQFPDDAAA VLQRQSSG S
GAGSS+SASSD+PFHL
Sbjct 1
MDLPAVAGRRTTTYSLLSQFPDDAAA------VLQRQSSGSSY-GAGSSLSASSDFPFHL 53

Query 61
PPAVAAAGGGGGTPSPCKSWAQQAEETYQLQLALALRLCADAACAADPGFLDPGDSGGS
K 120
P A A + G
SPCKSWAQQAEETYQLQLALALRLCADAACAADPGFLDPGDSG
Sbjct 54
PSAAAGSAAAGAGGSPCKSWAQQAEETYQLQLALALRLCADAACAADPGFLDPGDSG---
110

Query 121
MGGGGGGSGSGRAFPLAPPSPTAEALSHRFWVNGSLSYSNTIPDGFYLIQGMDPFVWSMC
180
SGSGRAFPL PP+ +AE+LSHRFWVNGSLSY NTIPDGFYLI
GMDPFVWS+C
Sbjct 111
-------SGSGRAFPLPPPTASAESLSHRFWVNGSLSYRNTIPDGFYLIHGMDPFVWSLC 163

Query 181
TDVHEENRIPSVESLKSVRPDDSSIQVVLVDRRADFDLGMLENYASSFLSSSSDMKDVIN
240

TDV EENRIPS+ESLKS+RPDDSSIQ +L+DRR DFDLGMLENYASSFLSSS+DMKDVIN
Sbjct 164 TDVLEENRIPSMESLKSIRPDDSSIQAILIDRRTDFDLGMLENYASSFLSSSADMKDVIN 223

Query 241 QLAKLVSSRMGGTTSNEENLLPRWKESSEAIKSSAGSIVLHLGKLPIGLCKHRSLLFKML 300
QLAKLVSSRMGGTTSNEE+ LPRWKE +AIKSS GSIVLHLGKLPIG CKHRSLLFKML
Sbjct 224 QLAKLVSSRMGGTTSNEESFLPRWKECRDAIKSSTGSIVLHLGKLPIGFCKHRSLLFKML 283

Query 301 ADKVNIPCRLVKGCKYCKAEDASSCVVRFGLEREYLVDLFGAPGQLSDPDSFVNGPYSLS 360
ADKVN+PCR+VKGCKYCK++DA+SC+VRFGLEREYLVDL G PGQLSDPDSFVNGPYSLS
Sbjct 284 ADKVNVPCRVVKGCKYCKSDDATSCLVRFGLEREYLVDLIGDPGQLSDPDSFVNGPYSLS 343

Query 361 VPSPLRPPKFRSLEITSNFSSVAKQYFSDCHSLNLLFSDASTGASNGAAVAVDQMYSKKH 420
VPSPLRPPKFRSLEITSNFSSVAKQYFSDCHSLNLLF++ASTGA++ AA+A+DQ+YS+KH
Sbjct 344 VPSPLRPPKFRSLEITSNFSSVAKQYFSDCHSLNLLFNEASTGATSSAAIAMDQLYSRKH 403

Query 421 DAGDGIANSWVPVKGQAIANSDIILPEAPREVLPLMSPSNLTADKKKEFQLIEGNQYLRS 480
D D +SWVPVKGQA +SD+ILPEAPREVLPL++ SNL ADKKKEF+LIEGNQYLRS
Sbjct 404 DTRDDTMSSWVPVKGQAAVSSDVILPEAPREVLPLITSSNLKADKKKEFKLIEGNQYLRS 463

Query 481 TVSDLSLAVDDLIIPWSELVLKEKIGAGSFGTVHRADWHGSDVAVKILMEQDYHLDRFKE

540

TVSDLSLAVDDLIIPW+ELVLKEKIGAGSFGTVHRADW+GSDVAVKILMEQD+H DRF+E
Sbjct 464
TVSDLSLAVDDLIIPWNELVLKEKIGAGSFGTVHRADWNGSDVAVKILMEQDFHPDRFRE
523

Query 541
FMREVAIMKSLRHPNIVLFMGAVTEPPNLSIVTEYLSRGSLYKLLHRSGAREVLDERRRL
600

FMREVAIMKSLRHPNIVLFMGAVTEPPNLSIVTEYLSRGSLYKLLHRSGA+EVLDERRRL
Sbjct 524
FMREVAIMKSLRHPNIVLFMGAVTEPPNLSIVTEYLSRGSLYKLLHRSGAKEVLDERRRL
583

Query 601
NMAFDVAKGMNYLHRRSPPIVHRDLKSPNLLVDKKYTVKVCDFGLSRLKANTYLSSKSL
A 660

NMAFDVAKGMNYLH+RSPPIVHRDLKSPNLLVDKKYTVKVCDFGLSRLKANT+LSSKSL
A
Sbjct 584
NMAFDVAKGMNYLHKRSPPIVHRDLKSPNLLVDKKYTVKVCDFGLSRLKANTFLSSKSL
A 643

Query 661
GTPEWMAPEVLRDEPSNEKSDVYSFAVILWELMTLQQPWCNLNPAQVVAAVGFKGRRLEI
720
GTPEWMAPEVLRDE SNEKSDVYSF
VILWELMT+QQPWCNLNPAQVVAAVGFKGRRL+I
Sbjct 644
GTPEWMAPEVLRDELSNEKSDVYSFGVILWELMTMQQPWCNLNPAQVVAAVGFKGRRL
DI 703

Query 721 PKELNPQVAALIESCWANEPWRRPSFANIMETLRPLINKVPVPQLIRSDS
770
PK+LNPQVAALIESCWANEPWRRPSFANIME+LR LI KVP+PQLIRSDS
Sbjct 704 PKDLNPQVAALIESCWANEPWRRPSFANIMESLRSLI-KVPLPQLIRSDS 752

>serine/threonine-protein kinase CTR1-like [Oryza brachyantha]

Sequence ID: XP_040383430.1 Length: 739
Range 1: 1 to 739

Score:1260 bits(3260), Expect:0.0,
Method:Compositional matrix adjust.,
Identities:648/770(84%), Positives:703/770(91%), Gaps:31/770(4%)

Query 1
MELPAAGGGRRTSYSLLSQFPDDAAAAGASPAVLQRQSSGGSSYGAGSSVSASSDYPFHL
60
M+LPA+G RR++YSLLSQFPDDAA +LQRQSSG S
GAGSS+SASSD+PFHL
Sbjct 1
MDLPASG--RRSTYSLLSQFPDDAA-------LLQRQSSGSSY-GAGSSLSASSDFPFHL 50

Query 61
PPAVAAAGGGGGTPSPCKSWAQQAEETYQLQLALALRLCADAACAADPGFLDPGDSGGS
K 120
P +A G SPCKSWAQQAEETYQLQLALALRLCADAA
AADPGFLDPGDSG +
Sbjct 51
P----SAAAAGAGGSPCKSWAQQAEETYQLQLALALRLCADAARAADPGFLDPGDSGSA-
105

Query 121
MGGGGGGSGSGRAFPLAPPSPTAEALSHRFWVNGSLSYSNTIPDGFYLIQGMDPFVWSMC
180
L PP+P+A++LSHRFWVNGSLSY+NTIPDGFYLI
GMDPFVWS+C
Sbjct 106 ---------------LPPPAPSADSLSHRFWVNGSLSYNNTIPDGFYLIHGMDPFVWSLC
150

Query 181
TDVHEENRIPSVESLKSVRPDDSSIQVVLVDRRADFDLGMLENYASSFLSSSDMKDVIN
240
TDV EENRIPS+ESLKSVRPDDSSIQ +L+DRR
DFDLGMLENYASSFLSSS+DMKDVIN
Sbjct 151
TDVLEENRIPSMESLKSVRPDDSSIQAILIDRRTDFDLGMLENYASSFLSSSADMKDVIN
210

Query 241
QLAKLVSSRMGGTTSNEENLLPRWKESSEAIKSSAGSIVLHLGKLPIGLCKHRSLLFKML
300

QLAKLVSSRMGGTTSNE++ LPRWKE S+AIKSS GSIVLHLGKLPIG CKHRSLLFKML
Sbjct 211
QLAKLVSSRMGGTTSNEDSFLPRWKECSDAIKSSTGSIVLHLGKLPIGFCKHRSLLFKML
270

Query 301
ADKVNIPCRLVKGCKYCKAEDASSCVVRFGLEREYLVDLFGAPGQLSDPDSFVNGPYSLS
360
ADKVN+PCR+VKGCKYCK++DA+SC+VRFGLEREYLVDL G PGQLSDPDSFVNGPYSLS
Sbjct 271
ADKVNVPCRVVKGCKYCKSDDATSCLVRFGLEREYLVDLIGDPGQLSDPDSFVNGPYSLS
330

Query 361
VPSPLRPPKFRSLEITSNFSSVAKQYFSDCHSLNLLFSDASTGASNGAAVAVDQMYSKKH
420
VPSPLRPPKFRSL I+SNFSSVAKQYFSDCHSLNLLF++ASTGA++GAA+A+DQ YS+KH
Sbjct 331
VPSPLRPPKFRSLMISSNFSSVAKQYFSDCHSLNLLFNEASTGATSGAAIAIDQPYSRKH
390

Query 421
DAGDGIANSWVPVKGQAIANSDIILPEAPREVLPLMSPSNLTADKKKEFQLIEGNQYLRS
480
D D +SWVPVKGQA +SD ILPEAPREVLPL++ SNL ADKKKEF+LIEGNQ+LRS
Sbjct 391
DTRDDTMSSWVPVKGQAAVSSDAILPEAPREVLPLITSSNLKADKKKEFKLIEGNQHLRS
450

Query 481
TVSDLSLAVDDLIIPWSELVLKEKIGAGSFGTVHRADWHGSDVAVKILMEQDYHLDRFKE
540

TVSDLSLAVDDLIIPW+EL+LKEKIGAGSFGTVHRADW+GSDVAVKILMEQD+H DRF+E
Sbjct 451
TVSDLSLAVDDLIIPWNELILKEKIGAGSFGTVHRADWNGSDVAVKILMEQDFHPDRFRE
510

Query 541
FMREVAIMKSLRHPNIVLFMGAVTEPPNLSIVTEYLSRGSLYKLLHRSGAREVLDERRRL

600

FMREVAIMKSLRHPNIVLFMGAVTEPPNLSIVTEYLSRGSLYKLLHRSGAREVLDERRRL
Sbjct 511
FMREVAIMKSLRHPNIVLFMGAVTEPPNLSIVTEYLSRGSLYKLLHRSGAREVLDERRRL
570

Query 601
NMAFDVAKGMNYLHRRSPPIVHRDLKSPNLLVDKKYTVKVCDFGLSRLKANTYLSSKSL
A 660

NMAFDVAKGMNYLH+RSPPIVHRDLKSPNLLVDKKYTVKVCDFGLSRLKANT+LSSKSL
A
Sbjct 571
NMAFDVAKGMNYLHKRSPPIVHRDLKSPNLLVDKKYTVKVCDFGLSRLKANTFLSSKSL
A 630

Query 661
GTPEWMAPEVLRDEPSNEKSDVYSFAVILWELMTLQQPWCNLNPAQVVAAVGFKGRRLEI
720
GTPEWMAPEVLRDEPSNEKSDVYSF
VILWELMT+QQPWCNLNPAQVVAAVGFKGRRL+I
Sbjct 631
GTPEWMAPEVLRDEPSNEKSDVYSFGVILWELMTMQQPWCNLNPAQVVAAVGFKGRRL
DI 690

Query 721 PKELNPQVAALIESCWANEPWRRPSFANIMETLRPLINKVPVPQLIRSDS
770
PK+LNPQVAALIESCWANEPWRRPSFANIME+LR LI KVP+PQL+RSDS
Sbjct 691 PKDLNPQVAALIESCWANEPWRRPSFANIMESLRSLI-KVPLPQLLRSDS 739

>hypothetical protein ZWY2020_054148 [Hordeum vulgare]
Sequence ID: KAI4998806.1 Length: 999
Range 1: 371 to 999

Score:1249 bits(3232), Expect:0.0,
Method:Compositional matrix adjust.,
Identities:605/629(96%), Positives:616/629(97%), Gaps:3/629(0%)

Query 145
ALSHRFW---VNGSLSYSNTIPDGFYLIQGMDPFVWSMCTDVHEENRIPSVESLKSVRPD

201

+ H W

VNGSLSY+NTIPDGFYLIQGMDPFVWSMCTDVHEENRIPSVESLKSVRPD

Sbjct 371

TVHHADWHGSVNGSLSYNNTIPDGFYLIQGMDPFVWSMCTDVHEENRIPSVESLKSVRP

D 430

Query 202

DSSIQVVLVDRRADFDLGMLENYASSFLSSSSDMKDVINQLAKLVSSRMGGTTSNEENLL

261

DSSIQVVLVDRRADFDLGMLENY

S+FLSSSSDMKDVINQLAKLVSSRMGGTTSNEENLL

Sbjct 431

DSSIQVVLVDRRADFDLGMLENYVSTFLSSSSDMKDVINQLAKLVSSRMGGTTSNEENLL

490

Query 262

PRWKESSEAIKSSAGSIVLHLGKLPIGLCKHRSLLFKMLADKVNIPCRLVKGCKYCKAED

321

PRWKESSEAIKSSAGSIVLHLGKLPIGLCKHRSLLFKMLADKVNIPCRLVKGCKYCKAED

Sbjct 491

PRWKESSEAIKSSAGSIVLHLGKLPIGLCKHRSLLFKMLADKVNIPCRLVKGCKYCKAED

550

Query 322

ASSCVVRFGLEREYLVDLFGAPGQLSDPDSFVNGPYSLSVPSPLRPPKFRSLEITSNFSS

381

ASSCVVRFGLEREYLVDLFGAPGQLSDPD

FVNGPYSLSVPSPLRPPKFRSLEITSNFSS

Sbjct 551

ASSCVVRFGLEREYLVDLFGAPGQLSDPDYFVNGPYSLSVPSPLRPPKFRSLEITSNFSS

610

Query 382

VAKQYFSDCHSLNLLFSDASTGASNGAAVAVDQMYSKKHDAGDGIANSWVPVKGQAIAN

S 441

VAKQYFSDCHSLNLLFSDASTGAS+GAAVAVDQMYSKKHDAGDGIANSWVPVKGQAIAN

Sbjct 611

VAKQYFSDCHSLNLLFSDASTGASSGAAVAVDQMYSKKHDAGDGIANSWVPVKGQAIAN

P 670

Query 442

DIILPEAPREVLPLMSPSNLTADKKKEFQLIEGNQYLRSTVSDLSLAVDDLIIPWSELVL
501

DIILPEAPRE+LPLM+PSNLTADKKKEFQLI
GNQYLRSTVSDLS+AVDDLIIPWSELVL
Sbjct 671
DIILPEAPREILPLMTPSNLTADKKKEFQLIGGNQYLRSTVSDLSIAVDDLIIPWSELVL 730

Query 502
KEKIGAGSFGTVHRADWHGSDVAVKILMEQDYHLDRFKEFMREVAIMKSLRHPNIVLFM
G 561

KEKIGAGSFGTVHRADWHGSDVAVKILMEQDYHLDRFKEFMREVAIMKSLR+PNIV+FMG
Sbjct 731
KEKIGAGSFGTVHRADWHGSDVAVKILMEQDYHLDRFKEFMREVAIMKSLRYPNIVVFM
G 790

Query 562
AVTEPPNLSIVTEYLSRGSLYKLLHRSGAREVLDERRRLNMAFDVAKGMNYLHRRSPPIV
621

AVTEPPNLSIVTEYLSRGSLYKLLH
SGAREVLDERRRLNMAFDVAKGMNYLHRRSPPIV
Sbjct 791
AVTEPPNLSIVTEYLSRGSLYKLLHMSGAREVLDERRRLNMAFDVAKGMNYLHRRSPPIV
850

Query 622
HRDLKSPNLLVDKKYTVKVCDFGLSRLKANTYLSSKSLAGTPEWMAPEVLRDEPSNEKS
D 681

HRD+KSPNLLVDKKYTVKVCDFGLSRLKANTYLSSKSLAGTPEWMAPEVLRDEPSNEKS
D
Sbjct 851
HRDMKSPNLLVDKKYTVKVCDFGLSRLKANTYLSSKSLAGTPEWMAPEVLRDEPSNEKS
D 910

Query 682
VYSFAVILWELMTLQQPWCNLNPAQVVAAVGFKGRRLEIPKELNPQVAALIESCWANEPW
741

VYSFAVILWELMTLQQPWCNLNPAQVVAAVGFKGRRLEIPK+LNPQVAALIESCW NEPW
Sbjct 911
VYSFAVILWELMTLQQPWCNLNPAQVVAAVGFKGRRLEIPKDLNPQVAALIESCWINEPW
970

Query 742 RRPSFANIMETLRPLINKVPVPQLIRSDS 770
RRPSFANIMETLRPLINKVPVPQLIRSDS
Sbjct 971 RRPSFANIMETLRPLINKVPVPQLIRSDS 999

Range 2: 306 to 380

Score:126 bits(317), Expect:2e-25,
Method:Compositional matrix adjust.,
Identities:60/75(80%), Positives:68/75(90%), Gaps:0/75(0%)

Query 447
EAPREVLPLMSPSNLTADKKKEFQLIEGNQYLRSTVSDLSLAVDDLIIPWSELVLKEKIG
506
E+ +EVLPLM+ S+LT+DKKKEFQLIEGN YL+S+
SDLSLAVDDLIIPW+ELVLKEKIG
Sbjct 306
ESIQEVLPLMTSSSLTSDKKKEFQLIEGNHYLQSSASDLSLAVDDLIIPWNELVLKEKIG
365

Query 507 AGSFGTVHRADWHGS 521
AG GTVH ADWHGS
Sbjct 366 AGFVGTVHHADWHGS 380

Range 3: 42 to 247

Score:80.5 bits(197), Expect:5e-11,
Method:Compositional matrix adjust.,
Identities:66/219(30%), Positives:102/219(46%), Gaps:35/219(15%)

Query 503
EKIGAGSFGTVHRADWHGSDVAVKILMEQDYHLDRFKEFMREVAIMKSLRHPNIVLFMG
A 562
+IG+G F V++ VAVK L + Y D EF REV + ++H N+V F+G
Sbjct 42
HEIGSGGFAVVYKGILGERAVAVKRL-NKAYMQD--TEFHREVQCLMQVKHKNVVRFLGY
98

Query 563

VT------EPPNLSIVT----------EYLSRGSLYKLLHRSGAREVLDERRRLNMAFDV 606
E N +V EY+ RGSL R + D + +
Sbjct 99
CADTQGNMERYNGKLVMADVQQRLLCFEYMPRGSL--------DRYIRDAYHEWKTCYKI
150

Query 607
AKGM----NYLHRRSPPIVHRDLKSPNLLVDKKYTVKVCDFGLSRL--KANTYLSSKSLA
660
KG+ YLH I+H DLK N+L+D ++ DFGLSR + T +K+++
Sbjct 151
IKGICEGIQYLHENR--IIHLDLKPANILLDDNMVPRITDFGLSRYFDENQTRDITKNIS 208

Query 661 GTPEWMAPEVLRDEPSNEKSDVYSFAVILWELMTLQQPW 699
GT ++APE+ +D+ S VI+ E++T Q+ +
Sbjct 209 GTMGYLAPELCEGGVIARSADLCSLGVIVIEILTGQKGY 247

>serine/threonine-protein kinase CTR1-like isoform X2 [Oryza glaberrima]
Sequence ID: XP_052166726.1 Length: 751
Range 1: 1 to 751

Score:1234 bits(3192), Expect:0.0,
Method:Compositional matrix adjust.,
Identities:649/771(84%), Positives:696/771(90%), Gaps:21/771(2%)

Query 1
MELPAAGGGRRTSYSLLSQFPDDAAAAGASPAVLQRQSSGGSSYGAGSSVSASSDYPFHL
60
M+LPA G R TSYSLLSQFPDDAA VLQRQSSG S
GAGSS+SASSD+PFHL
Sbjct 1
MDLPAVAGRRTTSYSLLSQFPDDAA-------VLQRQSSGSSY-GAGSSLSASSDFPFHL 52

Query 61
PPAVAAAGGGGGTPSPCKSWAQQAEETYQLQLALALRLCADAACAADPGFLDPGDSGGS
K 120
P A A A G G CKSWAQQAEETYQLQLALALRLCADAA AADP FLDPG S
+
Sbjct 53
PSAAAPAAGAPGGSP-CKSWAQQAEETYQLQLALALRLCADAASAADPAFLDPGHSATAT
111

Query 121
MGGGGGGSGSGRAFPLAPPSPTAEALSHRFWVNGSLSYSNTIPDGFYLIQGMDPFVWSMC
180
G + +A++LSHRFWVNGSLSYSNTIPDGFYLI
GMDPFVWS+C
Sbjct 112
TGPFPLPPPTP----------SADSLSHRFWVNGSLSYSNTIPDGFYLIHGMDPFVWSLC 161

Query 181
TDVHEENRIPSVESLKSVRPDDSSIQVVLVDRRADFDLGMLENYASSFLSSSDMKDVIN
240
TD+ EENRIPS+ESLKSVRPDDSSIQ +L+DRR
DFDLGMLENYASSFLSSS+DMKDVIN
Sbjct 162
TDLLEENRIPSIESLKSVRPDDSSIQAILIDRRTDFDLGMLENYASSFLSSSADMKDVIN
221

Query 241
QLAKLVSSRMGGTTSNEENLLPRWKESSEAIKSSAGSIVLHLGKLPIGLCKHRSLLFKML
300
QLAKLVSSRMGGTTSNEE+ LPRWKE S+AIKSS GSIVLHLGKLPIG
CKHRSLLFKML
Sbjct 222
QLAKLVSSRMGGTTSNEESFLPRWKECSDAIKSSTGSIVLHLGKLPIGFCKHRSLLFKML
281

Query 301
ADKVNIPCRLVKGCKYCKAEDASSCVVRFGLEREYLVDLFGAPGQLSDPDSFVNGPYSLS
360
ADKVN+PCR+VKGCKYCK++DA+SC+VRFGLEREYLVDL G
PGQLSDPDSFVNGPYSLS
Sbjct 282
ADKVNVPCRVVKGCKYCKSDDATSCLVRFGLEREYLVDLIGDPGQLSDPDSFVNGPYSLS
341

Query 361
VPSPLRPPKFRSLEITSNFSSVAKQYFSDCHSLNLLFSDASTGASNGAAVAVDQMYS-KK
419
VPSPLRPPKFRSLEITSNFSSVAKQYFSDCHSLNLLF++ASTGA++ AAVA+DQ
YS +K
Sbjct 342
VPSPLRPPKFRSLEITSNFSSVAKQYFSDCHSLNLLFNEASTGATSNAAVAMDQPYSTRK
401

Query 420
HDAGDGIANSWVPVKGQAIANSDIILPEAPREVLPLMSPSNLTADKKKEFQLIEGNQYLR
479
HD D I +SWVPVKGQA +SD ILPEAPREVLPL++ SNL
ADKKKEF+LIEGNQYLR
Sbjct 402
HDTRDDIMSSWVPVKGQAAVSSDAILPEAPREVLPLITSSNLKADKKKEFKLIEGNQYLR
461

Query 480
STVSDLSLAVDDLIIPWSELVLKEKIGAGSFGTVHRADWHGSDVAVKILMEQDYHLDRFK
539

STVSDLSLAVDDLIIPW+EL+LKEKIGAGSFGTVHRADW+GSDVAVKILMEQD+H DRF+
Sbjct 462
STVSDLSLAVDDLIIPWNELILKEKIGAGSFGTVHRADWNGSDVAVKILMEQDFHPDRFR
521

Query 540
EFMREVAIMKSLRHPNIVLFMGAVTEPPNLSIVTEYLSRGSLYKLLHRSGAREVLDERRR
599

EFMREVAIMKSLRHPNIVLFMGAVTEPPNLSIVTEYLSRGSLYKLLHRSGA+EVLDERRR
Sbjct 522
EFMREVAIMKSLRHPNIVLFMGAVTEPPNLSIVTEYLSRGSLYKLLHRSGAKEVLDERRR
581

Query 600
LNMAFDVAKGMNYLHRRSPPIVHRDLKSPNLLVDKKYTVKVCDFGLSRLKANTYLSSKS
L 659

LNMAFDVAKGMNYLH+RSPPIVHRDLKSPNLLVDKKYTVKVCDFGLSRLKANT+LSSKSL
Sbjct 582
LNMAFDVAKGMNYLHKRSPPIVHRDLKSPNLLVDKKYTVKVCDFGLSRLKANTFLSSKS
L 641

Query 660
AGTPEWMAPEVLRDEPSNEKSDVYSFAVILWELMTLQQPWCNLNPAQVVAAVGFKGRRL
E 719
AGTPEWMAPEVLRDEPSNEKSDVYSF
VILWELMT+QQPWCNLNPAQVVAAVGFKGRRL+
Sbjct 642
AGTPEWMAPEVLRDEPSNEKSDVYSFGVILWELMTMQQPWCNLNPAQVVAAVGFKGRR

LD 701

Query 720 IPKELNPQVAALIESCWANEPWRRPSFANIMETLRPLINKVPVPQLIRSDS 770
IPK+LNPQVAALIESCWANEPWRRPSFANIM++LR LI KVP+PQLIRSDS
Sbjct 702 IPKDLNPQVAALIESCWANEPWRRPSFANIMDSLRSLI-KVPLPQLIRSDS 751

>serine/threonine-protein kinase CTR1 [Oryza sativa Japonica Group]
Sequence ID: XP_015611604.1 Length: 751
>hypothetical protein EE612_049533 [Oryza sativa]
Sequence ID: KAB8111757.1 Length: 751
>hypothetical protein DAI22_09g203500 [Oryza sativa Japonica Group]
Sequence ID: KAF2917579.1 Length: 751
>Os09g0566550 [Oryza sativa Japonica Group]
Sequence ID: BAT09465.1 Length: 751
Range 1: 1 to 751

Score:1231 bits(3185), Expect:0.0,
Method:Compositional matrix adjust.,
Identities:646/771(84%), Positives:696/771(90%), Gaps:21/771(2%)

Query 1 MELPAAGGGRRTSYSLLSQFPDDAAAAGASPAVLQRQSSGGSSYGAGSSVSASSDYPFHL 60
M+LPA G R TSYSLLSQFPDDAA VLQRQSSG S GAGSS+SASSD+PFHL
Sbjct 1 MDLPAVTGRRTTSYSLLSQFPDDAA-------VLQRQSSGSSY-GAGSSLSASSDFPFHL 52

Query 61 PPAVAAAGGGGGTPSPCKSWAQQAEETYQLQLALALRLCADAACAADPGFLDPGDSGGSK 120
P A A A G G CKSWAQQAEETYQLQLALALRLCADAA AADP FLDPG S +
Sbjct 53 PSAAAPAAGAPGGSP-CKSWAQQAEETYQLQLALALRLCADAASAADPAFLDPGHSATAT 111

Query 121 MGGGGGGSGSGRAFPLAPPSPTAEALSHRFWVNGSLSYSNTIPDGFYLIQGMDPFVWSMC 180

G + +A++LSHRFWVNGSLSYSNTIPDGFYLI GMDPFVWS+C

Sbjct 112 AGPFPLPPPTP----------SADSLSHRFWVNGSLSYSNTIPDGFYLIHGMDPFVWSLC 161

Query 181 TDVHEENRIPSVESLKSVRPDDSSIQVVLVDRRADFDLGMLENYASSFLSSSSDMKDVIN 240

TD+ EENRIPS++SLKSVRPDDSS+Q +L+DRR DFDLGMLENYASSFLSSS+DMKDVIN

Sbjct 162 TDLLEENRIPSIDSLKSVRPDDSSMQAILIDRRTDFDLGMLENYASSFLSSSADMKDVIN 221

Query 241 QLAKLVSSRMGGTTSNEENLLPRWKESSEAIKSSAGSIVLHLGKLPIGLCKHRSLLFKML 300

QLAKLVSSRMGGTTSNEE+ LPRWKE S+AIKSS GSIVLHLGKLPIG CKHRSLLFKML

Sbjct 222 QLAKLVSSRMGGTTSNEESFLPRWKECSDAIKSSTGSIVLHLGKLPIGFCKHRSLLFKML 281

Query 301 ADKVNIPCRLVKGCKYCKAEDASSCVVRFGLEREYLVDLFGAPGQLSDPDSFVNGPYSLS 360

ADKVN+PCR+VKGCKYCK++DA+SC+VRFGLEREYLVDL G PGQLSDPDSFVNGPYSLS

Sbjct 282 ADKVNVPCRVVKGCKYCKSDDATSCLVRFGLEREYLVDLIGDPGQLSDPDSFVNGPYSLS 341

Query 361 VPSPLRPPKFRSLEITSNFSSVAKQYFSDCHSLNLLFSDASTGASNGAAVAVDQMYS-KK 419

VPSPLRPPKFRSLEITSNFSSVAKQYFSDCHSLNLLF++ASTGA++ AAVA+DQ YS +K

Sbjct 342 VPSPLRPPKFRSLEITSNFSSVAKQYFSDCHSLNLLFNEASTGANSNAAVAMDQPYSTRK 401

Query 420 HDAGDGIANSWVPVKGQAIANSDIILPEAPREVLPLMSPSNLTADKKKEFQLIEGNQYLR 479

HD D I +SWVPVKGQA +SD ILPEAPREVLPL++ SNL A+KKKEF+LIEGNQYLR
Sbjct 402 HDTRDDIMSSWVPVKGQAAVSSDAILPEAPREVLPLITSSNLKAEKKKEFKLIEGNQYLR 461

Query 480 STVSDLSLAVDDLIIPWSELVLKEKIGAGSFGTVHRADWHGSDVAVKILMEQDYHLDRFK 539

STVSDLSLAVDDLIIPW+EL+LKEKIGAGSFGTVHRADW+GSDVAVKILMEQD+H DRF+
Sbjct 462 STVSDLSLAVDDLIIPWNELILKEKIGAGSFGTVHRADWNGSDVAVKILMEQDFHPDRFR 521

Query 540 EFMREVAIMKSLRHPNIVLFMGAVTEPPNLSIVTEYLSRGSLYKLLHRSGAREVLDERRR 599

EFMREVAIMKSLRHPNIVLFMGAVTEPPNLSIVTEYLSRGSLYKLLHRSGA+EVLDERRR
Sbjct 522 EFMREVAIMKSLRHPNIVLFMGAVTEPPNLSIVTEYLSRGSLYKLLHRSGAKEVLDERRR 581

Query 600 LNMAFDVAKGMNYLHRRSPPIVHRDLKSPNLLVDKKYTVKVCDFGLSRLKANTYLSSKSL 659

LNMAFDVAKGMNYLH+RSPPIVHRDLKSPNLLVDKKYTVKVCDFGLSRLKANT+LSSKSL
Sbjct 582 LNMAFDVAKGMNYLHKRSPPIVHRDLKSPNLLVDKKYTVKVCDFGLSRLKANTFLSSKSL 641

Query 660 AGTPEWMAPEVLRDEPSNEKSDVYSFAVILWELMTLQQPWCNLNPAQVVAAVGFKGRRLE 719
AGTPEWMAPEVLRDEPSNEKSDVYSF VILWELMT+QQPWCNLNPAQVVAAVGFKGRRL+
Sbjct 642 AGTPEWMAPEVLRDEPSNEKSDVYSFGVILWELMTMQQPWCNLNPAQVVAAVGFKGRRLD 701

Query 720 IPKELNPQVAALIESCWANEPWRRPSFANIMETLRPLINKVPVPQLIRSDS 770

IPK+LNPQVAALIESCWANEPWRRPSFANIM++LR LI KVP+PQLIRSDS
Sbjct 702 IPKDLNPQVAALIESCWANEPWRRPSFANIMDSLRSLI-KVPLPQLIRSDS 751

>serine/threonine-protein kinase CTR1-like isoform X1 [Oryza glaberrima]
Sequence ID: XP_052166725.1 Length: 760
Range 1: 1 to 760

Score:1226 bits(3171), Expect:0.0,
Method:Compositional matrix adjust.,
Identities:648/780(83%), Positives:695/780(89%), Gaps:30/780(3%)

Query 1
MELPAAGGGRRTSYSLLSQFPDDAAAAGASPAVLQRQSSGGSSYGAGSSVSASSDYPFHL
60
M+LPA G R TSYSLLSQFPDDAA VLQRQSSG S
GAGSS+SASSD+PFHL
Sbjct 1
MDLPAVAGRRTTSYSLLSQFPDDAA-------VLQRQSSGSSY-GAGSSLSASSDFPFHL 52

Query 61
PPAVAAAGGGGGTPSPCKSWAQQAEETYQLQLALALRLCADAACAADPGFLDPGDSGGS
K 120
P A A A G G CKSWAQQAEETYQLQLALALRLCADAA AADP FLDPG S
+
Sbjct 53
PSAAAPAAGAPGGSP-CKSWAQQAEETYQLQLALALRLCADAASAADPAFLDPGHSATAT
111

Query 121
MGGGGGGSGSGRAFPLAPPSPTAEALSHRFWVNGSLSYSNTIPDGFYLIQGMDPFVWSMC
180
G + +A++LSHRFWVNGSLSYSNTIPDGFYLI
GMDPFVWS+C
Sbjct 112
TGPFPLPPPTP----------SADSLSHRFWVNGSLSYSNTIPDGFYLIHGMDPFVWSLC 161

Query 181
TDVHEENRIPSVESLKSVRPDDSSIQVVLVDRRADFDLGMLENYASSFLSSSSDMKDVIN
240
TD+ EENRIPS+ESLKSVRPDDSSIQ +L+DRR
DFDLGMLENYASSFLSSS+DMKDVIN

Sbjct 162
TDLLEENRIPSIESLKSVRPDDSSIQAILIDRRTDFDLGMLENYASSFLSSSADMKDVIN
221

Query 241
QLAKLVSSRMGGTTSNEENLLPRWKESSEAIKSSAGSIVLHLGKLPIGLCKHRSLLFKML
300
QLAKLVSSRMGGTTSNEE+ LPRWKE S+AIKSS GSIVLHLGKLPIG CKHRSLLFKML
Sbjct 222
QLAKLVSSRMGGTTSNEESFLPRWKECSDAIKSSTGSIVLHLGKLPIGFCKHRSLLFKML
281

Query 301
ADKVNIPCRLVKGCKYCKAEDASSCVVRFGLEREYLVDLFGAPGQLSDPDSFVNGPYSLS
360
ADKVN+PCR+VKGCKYCK++DA+SC+VRFGLEREYLVDL G PGQLSDPDSFVNGPYSLS
Sbjct 282
ADKVNVPCRVVKGCKYCKSDDATSCLVRFGLEREYLVDLIGDPGQLSDPDSFVNGPYSLS
341

Query 361
VPSPLRPPKFRSLEITSNFSSVAKQYFSDCHSLNLLFSDASTGASNGAAVAVDQMYS-KK
419
VPSPLRPPKFRSLEITSNFSSVAKQYFSDCHSLNLLF++ASTGA++ AAVA+DQ YS +K
Sbjct 342
VPSPLRPPKFRSLEITSNFSSVAKQYFSDCHSLNLLFNEASTGATSNAAVAMDQPYSTRK
401

Query 420
HDAGDGIANSWVPVKG---------QAIANSDIILPEAPREVLPLMSPSNLTADKKKEFQ 470
HD D I +SWVPVK QA +SD ILPEAPREVLPL++ SNL ADKKKEF+
Sbjct 402
HDTRDDIMSSWVPVKAYIHIMAQQSQAAVSSDAILPEAPREVLPLITSSNLKADKKKEFK
461

Query 471
LIEGNQYLRSTVSDLSLAVDDLIIPWSELVLKEKIGAGSFGTVHRADWHGSDVAVKILME
530

LIEGNQYLRSTVSDLSLAVDDLIIPW+EL+LKEKIGAGSFGTVHRADW+GSDVAVKILME

Sbjct 462
LIEGNQYLRSTVSDLSLAVDDLIIPWNELILKEKIGAGSFGTVHRADWNGSDVAVKILME
521

Query 531
QDYHLDRFKEFMREVAIMKSLRHPNIVLFMGAVTEPPNLSIVTEYLSRGSLYKLLHRSGA
590
QD+H
DRF+EFMREVAIMKSLRHPNIVLFMGAVTEPPNLSIVTEYLSRGSLYKLLHRSGA
Sbjct 522
QDFHPDRFREFMREVAIMKSLRHPNIVLFMGAVTEPPNLSIVTEYLSRGSLYKLLHRSGA
581

Query 591
REVLDERRRLNMAFDVAKGMNYLHRRSPPIVHRDLKSPNLLVDKKYTVKVCDFGLSRLK
A 650

+EVLDERRRLNMAFDVAKGMNYLH+RSPPIVHRDLKSPNLLVDKKYTVKVCDFGLSRLK
A
Sbjct 582
KEVLDERRRLNMAFDVAKGMNYLHKRSPPIVHRDLKSPNLLVDKKYTVKVCDFGLSRLK
A 641

Query 651
NTYLSSKSLAGTPEWMAPEVLRDEPSNEKSDVYSFAVILWELMTLQQPWCNLNPAQVVA
A 710
NT+LSSKSLAGTPEWMAPEVLRDEPSNEKSDVYSF
VILWELMT+QQPWCNLNPAQVVAA
Sbjct 642
NTFLSSKSLAGTPEWMAPEVLRDEPSNEKSDVYSFGVILWELMTMQQPWCNLNPAQVVA
A 701

Query 711
VGFKGRRLEIPKELNPQVAALIESCWANEPWRRPSFANIMETLRPLINKVPVPQLIRSDS
770
VGFKGRRL+IPK+LNPQVAALIESCWANEPWRRPSFANIM++LR LI
KVP+PQLIRSDS
Sbjct 702
VGFKGRRLDIPKDLNPQVAALIESCWANEPWRRPSFANIMDSLRSLI-KVPLPQLIRSDS
760

>hypothetical protein EJB05_44104, partial [Eragrostis curvula]
Sequence ID: TVU10563.1 Length: 762
Range 1: 1 to 762

Score:1224 bits(3166), Expect:0.0,
Method:Compositional matrix adjust.,
Identities:639/782(82%), Positives:682/782(87%), Gaps:32/782(4%)

Query 1
MELPAAGGGRRTSYSLLSQFPDDAAAAGASPAVLQRQSSGGSSYGAGSSVSASSDYPFHL
60
MELP G GRRT+YSLLSQ+PDDA P VLQRQSSG S SS+S
SSDYPFHL
Sbjct 1
MELPP-GAGRRTTYSLLSQYPDDAP-----PNVLQRQSSGSSYGAG-SSLSGSSDYPFHL 53

Query 61
PPAVAAAGG-----------GGGTPSPCKSWAQQAEETYQLQLALALRLCADAACAADPG 109
P + + GG S CKSWAQQAEETYQLQLALALRLCADAA
AADPG
Sbjct 54
QPPASISAAAAAALPVAGAATGG--SVCKSWAQQAEETYQLQLALALRLCADAASAADPG
111

Query 110
FLDPGDSGGSKMGGGGGGSGSGRAFPLAPPSPTAEALSHRFWVNGSLSYSNTIPDGFYLI
169
FLDPGDSG SG+ RAFPLA P+P+AE++SHRFWVNGSLSY+
TIPDGFYLI
Sbjct 112
FLDPGDSG----------SGNSRAFPLAQPTPSAESISHRFWVNGSLSYNTTIPDGFYLI 161

Query 170
QGMDPFVWSMCTDVHEENRIPSVESLKSVRPDDSSIQVVLVDRRADFDLGMLENYASSFL
229
GMDPFVWS+CTD HEE+RIPS+ESLKSVRPDDSSIQ +LVDRR DF+LGMLE
YA+S L
Sbjct 162
HGMDPFVWSLCTDAHEESRIPSMESLKSVRPDDSSIQAILVDRRTDFELGMLEGYAASLL
221

Query 230
SSSSDMKDVINQLAKLVSSRMGGTTSNEENLLPRWKESSEAIKSSAGSIVLHLGKLPIGL
289

SS+D KDV+NQLAKLVSSRMGGTTSNEENLLP W E EAIKSS GS+VLHLGKLPIGL
Sbjct 222
PSSADAKDVVNQLAKLVSSRMGGTTSNEENLLPGWNECIEAIKSSTGSVVLHLGKLPIGL
281

Query 290
CKHRSLLFKMLADKVNIPCRLVKGCKYCKAEDASSCVVRFGLEREYLVDLFGAPGQLSDP
349
CKHR+LLFKMLADKVNIPCR+VKGCKYCK++DASSC+VRFGLEREYLVDL G PGQL+DP
Sbjct 282
CKHRALLFKMLADKVNIPCRVVKGCKYCKSDDASSCLVRFGLEREYLVDLIGDPGQLADP
341

Query 350
DSFVNGPYSLSVPSPLRPPKFRSLEITSNFSSVAKQYFSDCHSLNLLFSDASTG-ASNGA
408
DSFVNGPYSLSV SPL PPKFRSLEITSNFSSVAKQYFSDCHSLNLLFSD+STG A+N
Sbjct 342
DSFVNGPYSLSVSSPLHPPKFRSLEITSNFSSVAKQYFSDCHSLNLLFSDSSTGCATNST
401

Query 409
AVAVDQMYSKKHDAGDGIANSWVPVKGQAIANSDIILPEAPREVLPLMSPSNLTADKKKE
468
A DQ +SKK AGD I +SWVP KGQ DII+PEAPREVLPL++ SNL DKK+E
Sbjct 402
VAASDQHFSKKPVAGDEILSSWVPGKGQPAMKPDIIVPEAPREVLPLITSSNLKLDKKQE
461

Query 469
FQLIEGNQYLRSTVSDLSLAVDDLIIPWSELVLKEKIGAGSFGTVHRADWHGSDVAVKIL
528
LIEG Q LR+TVSDLSLA DDLIIPWSELVLKEKIGAGSFG VHRADWHGSDVAVKIL
Sbjct 462
LALIEGTQQLRNTVSDLSLAADDLIIPWSELVLKEKIGAGSFGIVHRADWHGSDVAVKIL
521

Query 529
MEQDYHLDRFKEFMREVAIMKSLRHPNIVLFMGAVTEPPNLSIVTEYLSRGSLYKLLHRS

588

MEQD+H
+RF+EFMREVAIMKSLRHPNIVLFMGAVTEPPNLSIVTEYLSRGSLYKLLHRS
Sbjct 522
MEQDFHPERFREFMREVAIMKSLRHPNIVLFMGAVTEPPNLSIVTEYLSRGSLYKLLHRS
581

Query 589
GAREVLDERRRLNMAFDVAKGMNYLHRRSPPIVHRDLKSPNLLVDKKYTVKVCDFGLSR
L 648

GA+EVLDERRRLNMAFDVAKGMNYLHRRSPPIVHRDLKSPNLLVDKKYTVKVCDFGLSR
L
Sbjct 582
GAKEVLDERRRLNMAFDVAKGMNYLHRRSPPIVHRDLKSPNLLVDKKYTVKVCDFGLSR
L 641

Query 649
KANTYLSSKSLAGTPEWMAPEVLRDEPSNEKSDVYSFAVILWELMTLQQPWCNLNPAQV
V 708

KANT+LSSKSLAGTPEWMAPEVLRDEPSNEKSDVYSF
VILWELMTLQQPWCNLNPAQVV
Sbjct 642
KANTFLSSKSLAGTPEWMAPEVLRDEPSNEKSDVYSFGVILWELMTLQQPWCNLNPAQV
V 701

Query 709
AAVGFKGRRLEIPKELNPQVAALIESCWANEPWRRPSFANIMETLRPLINKVPVPQLIRS
768

AAVGFKGRRL+ PK+LNP VAALIESCWANEPWRRPSFANIMETL+PLI
KVPVPQLIRS
Sbjct 702
AAVGFKGRRLDNPKDLNPLVAALIESCWANEPWRRPSFANIMETLKPLI-KVPVPQLIRS
760

Query 769 DS 770

DS
Sbjct 761 DS 762

>hypothetical protein EJB05_44145, partial [Eragrostis curvula]
Sequence ID: TVU10602.1 Length: 762

Range 1: 1 to 762

Score:1223 bits(3164), Expect:0.0,
Method:Compositional matrix adjust.,
Identities:637/780(82%), Positives:679/780(87%), Gaps:28/780(3%)

Query 1
MELPAAGGGRRTSYSLLSQFPDDAAAAGASPAVLQRQSSGGSSYGAGSSVSASSDYPFHL
60
MELP G GRRT+YSLLSQ+PDDA P VLQRQSSG S SS+S
SSDYPFHL
Sbjct 1
MELPP-GAGRRTTYSLLSQYPDDAP-----PNVLQRQSSGSSYGAG-SSLSGSSDYPFHL 53

Query 61
PPAVAAAGGGGGTPSP---------CKSWAQQAEETYQLQLALALRLCADAACAADPGFL 111
P + + CKSWAQQAEETYQLQLALALRLCADAA
AADPGFL
Sbjct 54
QPPASISAAAAAALPAAGAATGGSACKSWAQQAEETYQLQLALALRLCADAASAADPGF
L 113

Query 112
DPGDSGGSKMGGGGGGSGSGRAFPLAPPSPTAEALSHRFWVNGSLSYSNTIPDGFYLIQG
171
DPGDSG SG+ RAFPLA P+P+AE++SHRFWVNGSLSY+
TIPDGFYLI G
Sbjct 114
DPGDSG----------SGNSRAFPLAQPTPSAESISHRFWVNGSLSYNTTIPDGFYLIHG 163

Query 172
MDPFVWSMCTDVHEENRIPSVESLKSVRPDDSSIQVVLVDRRADFDLGMLENYASSFLSS
231
MDPFVWS+CTD HEE+RIPS+ESLKSVRPDDSSIQ +LVDRR DF+LGMLE
YA+S L S
Sbjct 164
MDPFVWSLCTDAHEESRIPSMESLKSVRPDDSSIQAILVDRRTDFELGMLEGYAASLLPS
223

Query 232
SSDMKDVINQLAKLVSSRMGGTTSNEENLLPRWKESSEAIKSSAGSIVLHLGKLPIGLCK
291
S+D KDV+NQLAKLVSSRMGGTTSNEENLLP W E EAIKSS
GS+VLHLGKLPIGLCK

Sbjct 224
SADAKDVVNQLAKLVSSRMGGTTSNEENLLPGWNECIEAIKSSTGSVVLHLGKLPIGLCK
283

Query 292
HRSLLFKMLADKVNIPCRLVKGCKYCKAEDASSCVVRFGLEREYLVDLFGAPGQLSDPDS
351
HR+LLFKMLADKVNIPCR+VKGCKYCK++DASSC+VRFGLEREYLVDL G
PGQL+DPDS
Sbjct 284
HRALLFKMLADKVNIPCRVVKGCKYCKSDDASSCLVRFGLEREYLVDLIGDPGQLADPDS
343

Query 352
FVNGPYSLSVPSPLRPPKFRSLEITSNFSSVAKQYFSDCHSLNLLFSDASTG-ASNGAAV
410
FVNGPYSLSV SPL PPKFRSLEITSNFSSVAKQYFSDCHSLNLLFSD+STG
A+N
Sbjct 344
FVNGPYSLSVSSPLHPPKFRSLEITSNFSSVAKQYFSDCHSLNLLFSDSSTGCATNSTVA
403

Query 411
AVDQMYSKKHDAGDGIANSWVPVKGQAIANSDIILPEAPREVLPLMSPSNLTADKKKEFQ
470
A DQ +SKK AGD I NSWVP KGQ DII+PEAPREVLPL++ SNL DKK+E
Sbjct 404
ASDQHFSKKPVAGDEILNSWVPGKGQPAMKPDIIVPEAPREVLPLITSSNLKLDKKQELA
463

Query 471
LIEGNQYLRSTVSDLSLAVDDLIIPWSELVLKEKIGAGSFGTVHRADWHGSDVAVKILME
530
LIEG Q LR+TVSDLSLA DDLIIPWSELVLKEKIGAGSFG
VHRADWHGSDVAVKILME
Sbjct 464
LIEGTQQLRNTVSDLSLAADDLIIPWSELVLKEKIGAGSFGIVHRADWHGSDVAVKILME
523

Query 531
QDYHLDRFKEFMREVAIMKSLRHPNIVLFMGAVTEPPNLSIVTEYLSRGSLYKLLHRSGA
590
QD+H
+RF+EFMREVAIMKSLRHPNIVLFMGAVTEPPNLSIVTEYLSRGSLYKLLHRSGA

Sbjct 524
QDFHPERFREFMREVAIMKSLRHPNIVLFMGAVTEPPNLSIVTEYLSRGSLYKLLHRSGA
583

Query 591
REVLDERRRLNMAFDVAKGMNYLHRRSPPIVHRDLKSPNLLVDKKYTVKVCDFGLSRLK
A 650

+EVLDERRRLNMAFDVAKGMNYLHRRSPPIVHRDLKSPNLLVDKKYTVKVCDFGLSRLK
A
Sbjct 584
KEVLDERRRLNMAFDVAKGMNYLHRRSPPIVHRDLKSPNLLVDKKYTVKVCDFGLSRLK
A 643

Query 651
NTYLSSKSLAGTPEWMAPEVLRDEPSNEKSDVYSFAVILWELMTLQQPWCNLNPAQVVA
A 710
NT+LSSKSLAGTPEWMAPEVLRDEPSNEKSDVYSF
VILWELMTLQQPWCNLNPAQVVAA
Sbjct 644
NTFLSSKSLAGTPEWMAPEVLRDEPSNEKSDVYSFGVILWELMTLQQPWCNLNPAQVVA
A 703

Query 711
VGFKGRRLEIPKELNPQVAALIESCWANEPWRRPSFANIMETLRPLINKVPVPQLIRSDS
770
VGFKGRRL+ PK+LNP VAALIESCWANEPWRRPSFANIMETL+PLI
KVPVPQLIRSDS
Sbjct 704
VGFKGRRLDNPKDLNPLVAALIESCWANEPWRRPSFANIMETLKPLI-KVPVPQLIRSDS
762

>putative serine/threonine-specific protein kinase [Oryza sativa Japonica Group]
Sequence ID: BAD46244.1 Length: 760
>putative serine/threonine-specific protein kinase [Oryza sativa Japonica Group]
Sequence ID: BAD46666.1 Length: 760
Range 1: 1 to 760

Score:1221 bits(3160), Expect:0.0,
Method:Compositional matrix adjust.,
Identities:645/780(83%), Positives:695/780(89%), Gaps:30/780(3%)

Query 1
MELPAAGGGRRTSYSLLSQFPDDAAAAGASPAVLQRQSSGGSSYGAGSSVSASSDYPFHL
60
M+LPA G R TSYSLLSQFPDDAA VLQRQSSG S
GAGSS+SASSD+PFHL
Sbjct 1
MDLPAVTGRRTTSYSLLSQFPDDAA-------VLQRQSSGSSY-GAGSSLSASSDFPFHL 52

Query 61
PPAVAAAGGGGGTPSPCKSWAQQAEETYQLQLALALRLCADAACAADPGFLDPGDSGGS
K 120
P A A A G G CKSWAQQAEETYQLQLALALRLCADAA AADP FLDPG S
+
Sbjct 53
PSAAAPAAGAPGGSP-CKSWAQQAEETYQLQLALALRLCADAASAADPAFLDPGHSATAT
111

Query 121
MGGGGGGSGSGRAFPLAPPSPTAEALSHRFWVNGSLSYSNTIPDGFYLIQGMDPFVWSMC
180
G + +A++LSHRFWVNGSLSYSNTIPDGFYLI
GMDPFVWS+C
Sbjct 112
AGPFPLPPPTP----------SADSLSHRFWVNGSLSYSNTIPDGFYLIHGMDPFVWSLC 161

Query 181
TDVHEENRIPSVESLKSVRPDDSSIQVVLVDRRADFDLGMLENYASSFLSSSSDMKDVIN
240
TD+ EENRIPS++SLKSVRPDDSS+Q +L+DRR
DFDLGMLENYASSFLSSS+DMKDVIN
Sbjct 162
TDLLEENRIPSIDSLKSVRPDDSSMQAILIDRRTDFDLGMLENYASSFLSSSADMKDVIN
221

Query 241
QLAKLVSSRMGGTTSNEENLLPRWKESSEAIKSSAGSIVLHLGKLPIGLCKHRSLLFKML
300
QLAKLVSSRMGGTTSNEE+ LPRWKE S+AIKSS GSIVLHLGKLPIG
CKHRSLLFKML
Sbjct 222
QLAKLVSSRMGGTTSNEESFLPRWKECSDAIKSSTGSIVLHLGKLPIGFCKHRSLLFKML
281

Query 301
ADKVNIPCRLVKGCKYCKAEDASSCVVRFGLEREYLVDLFGAPGQLSDPDSFVNGPYSLS
360
ADKVN+PCR+VKGCKYCK++DA+SC+VRFGLEREYLVDL G
PGQLSDPDSFVNGPYSLS
Sbjct 282
ADKVNVPCRVVKGCKYCKSDDATSCLVRFGLEREYLVDLIGDPGQLSDPDSFVNGPYSLS
341

Query 361
VPSPLRPPKFRSLEITSNFSSVAKQYFSDCHSLNLLFSDASTGASNGAAVAVDQMYS-KK
419
VPSPLRPPKFRSLEITSNFSSVAKQYFSDCHSLNLLF++ASTGA++ AAVA+DQ
YS +K
Sbjct 342
VPSPLRPPKFRSLEITSNFSSVAKQYFSDCHSLNLLFNEASTGANSNAAVAMDQPYSTRK
401

Query 420
HDAGDGIANSWVPVKG---------QAIANSDIILPEAPREVLPLMSPSNLTADKKKEFQ 470
HD D I +SWVPVK QA +SD ILPEAPREVLPL++ SNL
A+KKKEF+
Sbjct 402
HDTRDDIMSSWVPVKAYIHIMAQQSQAAVSSDAILPEAPREVLPLITSSNLKAEKKKEFK
461

Query 471
LIEGNQYLRSTVSDLSLAVDDLIIPWSELVLKEKIGAGSFGTVHRADWHGSDVAVKILME
530

LIEGNQYLRSTVSDLSLAVDDLIIPW+EL+LKEKIGAGSFGTVHRADW+GSDVAVKILME
Sbjct 462
LIEGNQYLRSTVSDLSLAVDDLIIPWNELILKEKIGAGSFGTVHRADWNGSDVAVKILME
521

Query 531
QDYHLDRFKEFMREVAIMKSLRHPNIVLFMGAVTEPPNLSIVTEYLSRGSLYKLLHRSGA
590
QD+H
DRF+EFMREVAIMKSLRHPNIVLFMGAVTEPPNLSIVTEYLSRGSLYKLLHRSGA
Sbjct 522
QDFHPDRFREFMREVAIMKSLRHPNIVLFMGAVTEPPNLSIVTEYLSRGSLYKLLHRSGA
581

Query 591
REVLDERRRLNMAFDVAKGMNYLHRRSPPIVHRDLKSPNLLVDKKYTVKVCDFGLSRLK
A 650

+EVLDERRRLNMAFDVAKGMNYLH+RSPPIVHRDLKSPNLLVDKKYTVKVCDFGLSRLK
A
Sbjct 582
KEVLDERRRLNMAFDVAKGMNYLHKRSPPIVHRDLKSPNLLVDKKYTVKVCDFGLSRLK
A 641

Query 651
NTYLSSKSLAGTPEWMAPEVLRDEPSNEKSDVYSFAVILWELMTLQQPWCNLNPAQVVA
A 710
NT+LSSKSLAGTPEWMAPEVLRDEPSNEKSDVYSF
VILWELMT+QQPWCNLNPAQVVAA
Sbjct 642
NTFLSSKSLAGTPEWMAPEVLRDEPSNEKSDVYSFGVILWELMTMQQPWCNLNPAQVVA
A 701

Query 711
VGFKGRRLEIPKELNPQVAALIESCWANEPWRRPSFANIMETLRPLINKVPVPQLIRSDS
770
VGFKGRRL+IPK+LNPQVAALIESCWANEPWRRPSFANIM++LR LI
KVP+PQLIRSDS
Sbjct 702
VGFKGRRLDIPKDLNPQVAALIESCWANEPWRRPSFANIMDSLRSLI-KVPLPQLIRSDS
760

>hypothetical protein GUJ93_ZPchr0002g23815 [Zizania palustris]
Sequence ID: KAG8057783.1 Length: 740
Range 1: 1 to 736

Score:1216 bits(3146), Expect:0.0,
Method:Compositional matrix adjust.,
Identities:623/757(82%), Positives:679/757(89%), Gaps:21/757(2%)

Query 1
MELPAAGGGRRTSYSLLSQFPDDAAAAGASPAVLQRQSSGGSSYGAGSSVSASSDYPFHL
60
M+LP AG RRT+YSL+SQFPDD A VLQRQSSG S SS+SASSDYP
H+

Sbjct 1
MDLPVAG--RRTTYSLISQFPDDGA-------VLQRQSSGSSYGAG-SSLSASSDYPLHI 50

Query 61
PPAVAAAGGGGGTPSPCKSWAQQAEETYQLQLALALRLCADAACAADPGFLDPGDSGGS
K 120
P A AAA G +
KSWAQQAEETYQLQLALALRLCADAAC+ADPGFLDPGDSG
Sbjct 51
PAAAAAAAGSATAVA-GKSWAQQAEETYQLQLALALRLCADAACSADPGFLDPGDSG---
106

Query 121
MGGGGGGSGSGRAFPLAPPSPTAEALSHRFWVNGSLSYSNTIPDGFYLIQGMDPFVWSMC
180
SGSGRA P PP+P+AE+LSHRFWVNGSLSY NTI DGFYLI G+DP
VWS+C
Sbjct 107
-------SGSGRASPFPPPTPSAESLSHRFWVNGSLSYINTILDGFYLIHGLDPLVWSLC 159

Query 181
TDVHEENRIPSVESLKSVRPDDSSIQVVLVDRRADFDLGMLENYASSFLSSSSDMKDVIN
240
TDVHEENRIPS+ESLKSVRPD+SSIQV+L+DRR
DFDLGMLENYASSFLSSS+DM+DVIN
Sbjct 160
TDVHEENRIPSIESLKSVRPDESSIQVILIDRRTDFDLGMLENYASSFLSSSADMRDVIN
219

Query 241
QLAKLVSSRMGGTTSNEENLLPRWKESSEAIKSSAGSIVLHLGKLPIGLCKHRSLLFKML
300
QLAKLVSSRMGGTTSNEEN LP WKE +A+KSS GSIVLHLGKLPIG
CKHRSLLFKML
Sbjct 220
QLAKLVSSRMGGTTSNEENFLPHWKECIDAMKSSTGSIVLHLGKLPIGFCKHRSLLFKML
279

Query 301
ADKVNIPCRLVKGCKYCKAEDASSCVVRFGLEREYLVDLFGAPGQLSDPDSFVNGPYSLS
360
ADKVN+ CR+VKGCKYCK++D +SC+VRFGLEREYLVDL G
PG+LSDPDSFVNGPYSLS
Sbjct 280

ADKVNVSCRVVKGCKYCKSDDGTSCLVRFGLEREYLVDLIGDPGKLSDPDSFVNGPYSLS
339

Query 361
VPSPLRPPKFRSLEITSNFSSVAKQYFSDCHSLNLLFSDASTGASNGAAVAVDQMYSKKH
420
VPSPLRPPKFRS +ITSNFSSVAKQYFSDCHSL LLF++ASTGA++ A VA
DQ+YS+KH
Sbjct 340
VPSPLRPPKFRSSQITSNFSSVAKQYFSDCHSLILLFNEASTGAASSAEVAADQLYSRKH
399

Query 421
DAGDGIANSWVPVKGQAIANSDIILPEAPREVLPLMSPSNLTADKKKEFQLIEGNQYLRS
480
D D I +SWVPVKGQA NS+++LPEAPREVLPL++ S L ADKKKEF+LIEGNQ
LRS
Sbjct 400
DVRDDIMSSWVPVKGQATVNSNVVLPEAPREVLPLIASSKLKADKKKEFKLIEGNQQLRS
459

Query 481
TVSDLSLAVDDLIIPWSELVLKEKIGAGSFGTVHRADWHGSDVAVKILMEQDYHLDRFKE
540

TVSD++LAVDDLIIPW+ELVLKEKIGAGSFGTVHRADW+GSDVAVKILMEQD+H DRF+E
Sbjct 460
TVSDITLAVDDLIIPWNELVLKEKIGAGSFGTVHRADWNGSDVAVKILMEQDFHPDRFRE
519

Query 541
FMREVAIMKSLRHPNIVLFMGAVTEPPNLSIVTEYLSRGSLYKLLHRSGAREVLDERRRL
600
F+REVAIMKSLRHPNIVLFMGAVTEPPNLSIVTEYLSRGSLYKLLHRSGA+E
LDERRRL
Sbjct 520
FLREVAIMKSLRHPNIVLFMGAVTEPPNLSIVTEYLSRGSLYKLLHRSGAKEALDERRRL
579

Query 601
NMAFDVAKGMNYLHRRSPPIVHRDLKSPNLLVDKKYTVKVCDFGLSRLKANTYLSSKSL
A 660

NMAFDVAKGMNYLH+RSPPIVHRDLKSPNLLVDKKYTVKVCDFGLSRLKANT+LSSKSL

A
Sbjct 580
NMAFDVAKGMNYLHKRSPPIVHRDLKSPNLLVDKKYTVKVCDFGLSRLKANTFLSSKSL
A 639

Query 661
GTPEWMAPEVLRDEPSNEKSDVYSFAVILWELMTLQQPWCNLNPAQVVAAVGFKGRRLEI
720
GTPEWMAPEVLRDEPSNEKSDVYSF
VILWELMT+QQPWCNLNPAQVVAAVGFK RRL+I
Sbjct 640
GTPEWMAPEVLRDEPSNEKSDVYSFGVILWELMTMQQPWCNLNPAQVVAAVGFKCRRL
DI 699

Query 721 PKELNPQVAALIESCWANEPWRRPSFANIMETLRPLI 757
PK+LN QVA LIESCWANEPWRRPSFANIM++LR LI
Sbjct 700 PKDLNHQVAVLIESCWANEPWRRPSFANIMDSLRSLI 736

>hypothetical protein QOZ80_6AG0525500 [Eleusine coracana subsp. coracana]
Sequence ID: KAK3132638.1 Length: 762
Range 1: 1 to 762

Score:1212 bits(3135), Expect:0.0,
Method:Compositional matrix adjust.,
Identities:634/779(81%), Positives:679/779(87%), Gaps:26/779(3%)

Query 1
MELPAAGGGRRTSYSLLSQFPDDAAAAGASPAVLQRQSSGGSSYGAGSSVSASSDYPFHL
60
M+LP G RRT+YSLLSQ+P D A VLQRQSSG S
GAGSSVSASSDYPFHL
Sbjct 1
MDLPP-GAARRTTYSLLSQYPPDDGPAN----VLQRQSSGSSY-GAGSSVSASSDYPFHL 54

Query 61
PPAVAAAGGGGGTPSP---------CKSWAQQAEETYQLQLALALRLCADAACAADPGFL 111
P + + CKSWAQQAEETYQLQLALALRLCADAA
AADPGFL
Sbjct 55
QPPASVSAAAAAALPSAAAPAAGSSCKSWAQQAEETYQLQLALALRLCADAASAADPGF
L 114

Query 112
DPGDSGGSKMGGGGGGSGSGRAFPLAPPSPTAEALSHRFWVNGSLSYSNTIPDGFYLIQG
171
DPG+ G SGSGRAFPLA
P+P+AE++SHRFWVNGSLSY++TIPDGFYLI G
Sbjct 115
DPGEPG----------SGSGRAFPLAQPTPSAESVSHRFWVNGSLSYNSTIPDGFYLIHG 164

Query 172
MDPFVWSMCTDVHEENRIPSVESLKSVRPDDSSIQVVLVDRRADFDLGMLENYASSFLSS
231
MDPFVWS+CTD HEENRIPS+ESL SVRPDDS IQ +LVDRR DF+LGMLE YA+S
LSS
Sbjct 165
MDPFVWSLCTDAHEENRIPSMESLMSVRPDDSPIQAILVDRRTDFELGMLEGYAASLLSS
224

Query 232
SSDMKDVINQLAKLVSSRMGGTTSNEENLLPRWKESSEAIKSSAGSIVLHLGKLPIGLCK
291
S+D KDV+NQLAKLVS+RMGG TS+EENLLPRW E SEAIKSS
GS+VLHLGKLP+GLCK
Sbjct 225
SADAKDVVNQLAKLVSTRMGGATSSEENLLPRWNECSEAIKSSTGSVVLHLGKLPVGLCK
284

Query 292
HRSLLFKMLADKVNIPCRLVKGCKYCKAEDASSCVVRFGLEREYLVDLFGAPGQLSDPDS
351
HRSLLFK+LADKVNIPCR+VKGCKYCK++DASSC+VRFGLEREYLVDL G PG
L+DPDS
Sbjct 285
HRSLLFKILADKVNIPCRVVKGCKYCKSDDASSCLVRFGLEREYLVDLIGDPGHLADPDS
344

Query 352
FVNGPYSLSVPSPLRPPKFRSLEITSNFSSVAKQYFSDCHSLNLLFSDASTGASNGAAVA
411
FVNGPYSLSV SPL PPKFRSL+ITSNFSSVAKQYFSDCHSLNLLFSD+STGA++
A
Sbjct 345
FVNGPYSLSVCSPLHPPKFRSLDITSNFSSVAKQYFSDCHSLNLLFSDSSTGAASNTVAA
404

Query 412
VDQMYSKKHDAGDGIANSWVPVKGQAIANSDIILPEAPREVLPLMSPSNLTADKKKEFQL
471
DQ YSKK AGD I NSWVP KGQ DII+PEAPREVLPL++ SNL DKK+E
L
Sbjct 405
SDQHYSKKPVAGDEIMNSWVPGKGQPAIKPDIIVPEAPREVLPLITSSNLKLDKKQELAL
464

Query 472
IEGNQYLRSTVSDLSLAVDDLIIPWSELVLKEKIGAGSFGTVHRADWHGSDVAVKILMEQ
531
IEG Q LR+TVSDLSLA
DDLIIPWSELVLKEKIGAGSFGTVHRADWHGSDVAVKILMEQ
Sbjct 465
IEGTQQLRNTVSDLSLAADDLIIPWSELVLKEKIGAGSFGTVHRADWHGSDVAVKILMEQ
524

Query 532
DYHLDRFKEFMREVAIMKSLRHPNIVLFMGAVTEPPNLSIVTEYLSRGSLYKLLHRSGAR
591
D+H +RF+EFMREVAIMKSLRHPNIVLFMGAVTEPP
LSIVTEYLSRGSLYKLLHRSGA+
Sbjct 525
DFHPERFREFMREVAIMKSLRHPNIVLFMGAVTEPPKLSIVTEYLSRGSLYKLLHRSGAK
584

Query 592
EVLDERRRLNMAFDVAKGMNYLHRRSPPIVHRDLKSPNLLVDKKYTVKVCDFGLSRLKA
N 651

EVLDERRRLNMAFDVAKGMNYLHRRSPPIVHRDLKSPNLLVDKKYTVKVCDFGLSRLKA
N
Sbjct 585
EVLDERRRLNMAFDVAKGMNYLHRRSPPIVHRDLKSPNLLVDKKYTVKVCDFGLSRLKA
N 644

Query 652
TYLSSKSLAGTPEWMAPEVLRDEPSNEKSDVYSFAVILWELMTLQQPWCNLNPAQVVAAV
711
T+LSSKSLAGTPEWMAPEVLRDEPSNEKSDVYSF
VILWELMTLQQPWCNLNPAQVVAAV
Sbjct 645

TFLSSKSLAGTPEWMAPEVLRDEPSNEKSDVYSFGVILWELMTLQQPWCNLNPAQVVAA
V 704

Query 712
GFKGRRLEIPKELNPQVAALIESCWANEPWRRPSFANIMETLRPLINKVPVPQLIRSDS
770
GFK RRL+IPK+LNP VAALIESCWANEPWRRPSFANIME LRPLI
KVPVPQLIRSDS
Sbjct 705
GFKARRLDIPKDLNPLVAALIESCWANEPWRRPSFANIMEALRPLI-KVPVPQLIRSDS 762

>hypothetical protein U9M48_012797 [Paspalum notatum var. saurae]
Sequence ID: WVZ63138.1 Length: 770
Range 1: 6 to 770

Score:1209 bits(3128), Expect:0.0,
Method:Compositional matrix adjust.,
Identities:640/773(83%), Positives:690/773(89%), Gaps:17/773(2%)

Query 7
GGGRRTSYSLLSQFPDDAAAAGASPAVLQRQSSGGSSYGAGSSVSASSDYPFHLPPAVAA
66
G RRT+YSLLSQFPDDAAA G + VLQRQSSG S GAGSS+SASSDYPFHL P
+A
Sbjct 6
GAARRTTYSLLSQFPDDAAAPGPA-NVLQRQSSGSSY-GAGSSISASSDYPFHLQPPASA
63

Query 67
AGGG---------GGTPSPCKSWAQQAEETYQLQLALALRLCADAACAADPGFLDPGDSG
117
AG G
SPCKSWAQQAEETYQLQLALALRLCADAACAADPGFLDPGDSG
Sbjct 64
AGVAQPGSAAAPPGAAGSPCKSWAQQAEETYQLQLALALRLCADAACAADPGFLDPGDS
G 123

Query 118
GSKMGGGGGGSGSGRAFPLAPPSPTAEALSHRFWVNGSLSYSNTIPDGFYLIQGMDPFVW
177
+ G G+GRAFPLA P+P+AE+LSHRFWVNGSLSY++TIPDGFYLI

GMDPFVW
Sbjct 124
SGRGSGN---GGNGRAFPLAHPAPSAESLSHRFWVNGSLSYNSTIPDGFYLIHGMDPFVW
180

Query 178
SMCTDVHEENRIPSVESLKSVRPDDSSIQVVLVDRRADFDLGMLENYASSFLSSSSDMKD
237
S+CTD+HEENRIPS+ESLKSVRPDDSSIQ +L+DRR DF+LGMLE+YAS
SSS+D KD
Sbjct 181
SLCTDIHEENRIPSMESLKSVRPDDSSIQAILIDRRNDFELGMLESYASR--SSSADAKD 238

Query 238
VINQLAKLVSSRMGGTTSNEENLLPRWKESSEAIKSSAGSIVLHLGKLPIGLCKHRSLLF
297
V+ QLAKL+SSRMGGT SNEENLL +WKE +EAI+SS
GS+VLHLGKLPIGLCKHRSLLF
Sbjct 239
VVIQLAKLISSRMGGTASNEENLLQQWKECTEAIRSSTGSVVLHLGKLPIGLCKHRSLLF
298

Query 298
KMLADKVNIPCRLVKGCKYCKAEDASSCVVRFGLEREYLVDLFGAPGQLSDPDSFVNGP
Y 357
K+LADKVNIPCR+VKGCKYCK++DASSC+VRFGLEREYLVDL G
PGQL+DPDSFVNGPY
Sbjct 299
KILADKVNIPCRVVKGCKYCKSDDASSCLVRFGLEREYLVDLIGDPGQLTDPDSFVNGPY
358

Query 358
SLSVPSPLRPPKFRSLEITSNFSSVAKQYFSDCHSLNLLFSDASTGASNGAAVAVDQMYS
417
SLSV SPL PPKFRSLEITSNFSSVAKQYFSDCHSL LLFSD+STG +N V++DQ
Y
Sbjct 359
SLSVSSPLHPPKFRSLEITSNFSSVAKQYFSDCHSLILLFSDSSTGVANSTVVSLDQPYY
418

Query 418
KKHDAGDGIANSWVPVKGQAIANSDIILPEAPREVLPLMSPSNLTADKKKEFQLIEGNQY
477
KKH AG+ + NSWVP KGQA+ DII+PEAPREVLPL++ SN+ DKKKE LI+

Q
Sbjct 419
KKHVAGNDVINSWVPGKGQAVMKPDIIVPEAPREVLPLITSSNMKPDKKKELTLIDETQP
478

Query 478
LRSTVSDLSLAVDDLIIPWSELVLKEKIGAGSFGTVHRADWHGSDVAVKILMEQDYHLDR
537
LR+ VSDLSLA
DDLIIPWSELVLKEKIGAGSFGTVHRADWHGSDVAVKILMEQD+H +R
Sbjct 479
LRNAVSDLSLAADDLIIPWSELVLKEKIGAGSFGTVHRADWHGSDVAVKILMEQDFHPER
538

Query 538
FKEFMREVAIMKSLRHPNIVLFMGAVTEPPNLSIVTEYLSRGSLYKLLHRSGAREVLDER
597

F+EFMREVAIMKSLRHPNIVLFMGAVTEPPNLSIVTEYLSRGSLYKLLHRSGA+EVLDER
Sbjct 539
FREFMREVAIMKSLRHPNIVLFMGAVTEPPNLSIVTEYLSRGSLYKLLHRSGAKEVLDER
598

Query 598
RRLNMAFDVAKGMNYLHRRSPPIVHRDLKSPNLLVDKKYTVKVCDFGLSRLKANTYLSS
K 657

RRLNMAFDVAKGMNYLHRRSPPIVHRDLKSPNLLVDKKYTVKVCDFGLSRLKANT+LSS
K
Sbjct 599
RRLNMAFDVAKGMNYLHRRSPPIVHRDLKSPNLLVDKKYTVKVCDFGLSRLKANTFLSS
K 658

Query 658
SLAGTPEWMAPEVLRDEPSNEKSDVYSFAVILWELMTLQQPWCNLNPAQVVAAVGFKGR
R 717
SLAGTPEWMAPEVLRDEPSNEKSDVYSF
VILWELMTLQQPWCNLNPAQVVAAVGFKGRR
Sbjct 659
SLAGTPEWMAPEVLRDEPSNEKSDVYSFGVILWELMTLQQPWCNLNPAQVVAAVGFKGR
R 718

Query 718 LEIPKELNPQVAALIESCWANEPWRRPSFANIMETLRPLINKVPVPQLIRSDS
770

LEIPK+LNP VAALIESCWANEPWRRPSF NIMETLR LI KVPV QLIRSDS
Sbjct 719 LEIPKDLNPLVAALIESCWANEPWRRPSFTNIMETLRSLI-KVPVAQLIRSDS 770

>serine/threonine-protein kinase CTR1 isoform X1 [Phragmites australis]
Sequence ID: XP_062194396.1 Length: 764
Range 1: 1 to 764

Score:1204 bits(3115), Expect:0.0,
Method:Compositional matrix adjust.,
Identities:639/775(82%), Positives:692/775(89%), Gaps:16/775(2%)

Query 1 MELPAAGGGRRTSYSLLSQFPDDAAAAGASPAVLQRQSSGGSSYGAGSSVSASSDYPFHL 60
M+LP G RRT+YSLLSQFP+DA VLQRQSSG SSYGAGSS+SASSDYPFHL
Sbjct 1 MDLPP-GAARRTTYSLLSQFPEDAPGPAN---VLQRQSSGSSSYGAGSSISASSDYPFHL 56

Query 61 PPAVAAAGGGGGTPS-----PCKSWAQQAEETYQLQLALALRLCADAACAADPGFLDPGD 115
P VA + + PCK+WAQQAEETYQLQLALALRLCADAACAADPGFLDPGD
Sbjct 57 QPPVAGSATTTAAAAASGGSPCKTWAQQAEETYQLQLALALRLCADAACAADPGFLDPGD 116

Query 116 SGGSKMGGGGGGSGSGRAFPLAPPSPTAEALSHRFWVNGSLSYSNTIPDGFYLIQGMDPF 175
SG + SG+GRAFPLA P+P+AE+LSHRFWVNGSLSY++TIPDGFYLI GMDPF
Sbjct 117 SGSGRG------SGNGRAFPLAQPAPSAESLSHRFWVNGSLSYNSTIPDGFYLIHGMDPF 170

Query 176 VWSMCTDVHEENRIPSVESLKSVRPDDSSIQVVLVDRRADFDLGMLENYASSFLSSSSDM 235
VWS+CTDVH+ENRIPS+ESLKSVRPDDSSIQ +L+DRR DF+LGMLE+YASS

LSS +D
Sbjct 171
VWSLCTDVHDENRIPSMESLKSVRPDDSSIQAILIDRRTDFELGMLESYASSLLSSFADA
230

Query 236
KDVINQLAKLVSSRMGGTTSNEENLLPRWKESSEAIKSSAGSIVLHLGKLPIGLCKHRSL
295
KDV+ QLAKL+SSR GGTTS+EENLLP+WKE EAIKSS
GS+VLHLGKLPIGLCKHRSL
Sbjct 231
KDVVKQLAKLISSRTGGTTSDEENLLPQWKECCEAIKSSTGSVVLHLGKLPIGLCKHRSL
290

Query 296
LFKMLADKVNIPCRLVKGCKYCKAEDASSCVVRFGLEREYLVDLFGAPGQLSDPDSFVN
G 355
LFKMLADKVN+ CR+VKGCKYCK++DASSC+VRFGLEREYLVDL G
PGQL+DPDSFVNG
Sbjct 291
LFKMLADKVNVRCRVVKGCKYCKSDDASSCLVRFGLEREYLVDLIGDPGQLTDPDSFVN
G 350

Query 356
PYSLSVPSPLRPPKFRSLEITSNFSSVAKQYFSDCHSLNLLFSDASTGASNGAAVAVDQM
415
PYSLSV SPL PPKFRSLEITSNFSSVAKQYFSDCHSLNLLFSD+STGA+N
AAVA+DQ
Sbjct 351
PYSLSVSSPLHPPKFRSLEITSNFSSVAKQYFSDCHSLNLLFSDSSTGAANSAAVALDQP
410

Query 416
YSKKHDAGDGIANSWVPVKGQAIANSDIILPEAPREVLPLMSPSNLTADKKKEFQLIEGN
475
YSKKH AGD I N WVP KGQA II+PE PR+VLPL++ SNL DKK++ LIEG
Sbjct 411
YSKKHVAGDDIINRWVPGKGQAAMKPAIIVPEDPRKVLPLITSSNLKLDKKQQLALIEGT
470

Query 476
QYLRSTVSDLSLAVDDLIIPWSELVLKEKIGAGSFGTVHRADWHGSDVAVKILMEQDYHL
535
+ L+ TVS LSLA

DDLIIPW+ELVLKEKIGAGSFGTVHRADWHGSDVAVKILMEQD+H
Sbjct 471
KQLQDTVSGLSLAADDLIIPWNELVLKEKIGAGSFGTVHRADWHGSDVAVKILMEQDFHP
530

Query 536
DRFKEFMREVAIMKSLRHPNIVLFMGAVTEPPNLSIVTEYLSRGSLYKLLHRSGAREVLD
595

+RF+EFMREVAIMKSLRHPNIVLFMGAVTEPPNLSIVTEYLSRGSLYKLLHRSGA+EVLD
Sbjct 531
ERFREFMREVAIMKSLRHPNIVLFMGAVTEPPNLSIVTEYLSRGSLYKLLHRSGAKEVLD
590

Query 596
ERRRLNMAFDVAKGMNYLHRRSPPIVHRDLKSPNLLVDKKYTVKVCDFGLSRLKANTYL
S 655

ERRRLNMAFDVAKGMNYLHRR+PPIVHRDLKSPNLLVDKKYTVKVCDFGLSRLKANT+L
S
Sbjct 591
ERRRLNMAFDVAKGMNYLHRRNPPIVHRDLKSPNLLVDKKYTVKVCDFGLSRLKANTFL
S 650

Query 656
SKSLAGTPEWMAPEVLRDEPSNEKSDVYSFAVILWELMTLQQPWCNLNPAQVVAAVGFK
G 715
SKSLAGTPEWMAPEVLRDE SNEKSDVYS+
VILWELMTLQQPWCNLNPAQVVAAVGFKG
Sbjct 651
SKSLAGTPEWMAPEVLRDELSNEKSDVYSYGVILWELMTLQQPWCNLNPAQVVAAVGFK
G 710

Query 716
RRLEIPKELNPQVAALIESCWANEPWRRPSFANIMETLRPLINKVPVPQLIRSDS 770
RRLEIPK+LNP VAALIESCWANEPWRRPSFANIM TLRPLI KVPVPQLIRSDS
Sbjct 711 RRLEIPKDLNPLVAALIESCWANEPWRRPSFANIMVTLRPLI-KVPVPQLIRSDS
764

>Serine/threonine-protein kinase CTR1 [Dichanthelium oligosanthes]
Sequence ID: OEL25444.1 Length: 769

Range 1: 1 to 769

Score:1201 bits(3107), Expect:0.0,
Method:Compositional matrix adjust.,
Identities:649/780(83%), Positives:698/780(89%), Gaps:21/780(2%)

Query 1
MELPAAGGGRRTSYSLLSQFPDDAAAAGASPAVLQRQSSGGSSYGAGSSVSASSDYPFHL
60
M+LP G RRT+YSLLSQFPDDAAA A+ VLQRQSSG S
GAGSS+SASSDYPFHL
Sbjct 1
MDLPP-GAARRTTYSLLSQFPDDAAAGPAN--VLQRQSSGSSY-GAGSSISASSDYPFHL 56

Query 61
-PPA---------VAAAGGGGGTPSPCKSWAQQAEETYQLQLALALRLCADAACAADPGF 110
PPA AAA G G
SPCKSWAQQAEETYQLQLALALRLCADAACAADPGF
Sbjct 57
QPPASVAGVAPAGSAAAAPGAGGSSPCKSWAQQAEETYQLQLALALRLCADAACAADPG
F 116

Query 111
LDPGDSGGSKMGGGGGGSGSGRAFPLAPPSPTAEALSHRFWVNGSLSYSNTIPDGFYLIQ
170
LDPGDSG + SG+GRAFPLA
P+P+AE+LSHRFWVNGSLSY++TIPDGFY+I
Sbjct 117
LDPGDSGSGRG------SGNGRAFPLAQPAPSAESLSHRFWVNGSLSYNSTIPDGFYMIH 170

Query 171
GMDPFVWSMCTDVHEENRIPSVESLKSVRPDDSSIQVVLVDRRADFDLGMLENYASSFLS
230
GMDP+VWS+CTDVHEENRIPS+ESLKSVRPDDSSI+ +L+DRR
DFDLGMLE+YASS LS
Sbjct 171
GMDPYVWSLCTDVHEENRIPSMESLKSVRPDDSSIEAILIDRRTDFDLGMLESYASSLLS
230

Query 231
SSSDMKDVINQLAKLVSSRMGGTTSNEENLLPRWKESSEAIKSSAGSIVLHLGKLPIGLC
290
SS+D KDV+ QLAK+VSSRMGGT SNEENLLPRWKE +EAIKSS
GS++LHLGKLP+GLC

Sbjct 231
SSTDAKDVVMQLAKVVSSRMGGTISNEENLLPRWKECNEAIKSSTGSVLLHLGKLPVGLC
290

Query 291
KHRSLLFKMLADKVNIPCRLVKGCKYCKAEDASSCVVRFGLEREYLVDLFGAPGQLSDP
D 350
KHRSLLFKMLADKV+IPCR+VKGCKYCK++DASSC+VRFGLEREYLVDL G
PGQLSDPD
Sbjct 291
KHRSLLFKMLADKVSIPCRVVKGCKYCKSDDASSCLVRFGLEREYLVDLIGDPGQLSDPD
350

Query 351
SFVNGPYSLSVPSPLRPPKFRSLEITSNFSSVAKQYFSDCHSLNLLFSDASTGASNGAAV
410
SFVNGPYSLSV SPL PPKFRSLEITSNFSSVAKQYFSDCHSLNLLFSD+STGA+
V
Sbjct 351
SFVNGPYSLSVSSPLHPPKFRSLEITSNFSSVAKQYFSDCHSLNLLFSDSSTGAATSTVV
410

Query 411
AVDQMYSKKHDAGDGIANSWVPVKGQAIANSDIILPEAPREVLPLMSPSNLTADKKKEFQ
470
++DQ Y KKH AGD NSW+P KGQA DII+PE PREVLPL++ SNL
DKKKE
Sbjct 411
SLDQPYCKKHVAGDETMNSWMPGKGQAAMKPDIIVPEVPREVLPLITSSNLKLDKKKEL
A 470

Query 471
LIEGNQYLRSTVSDLSLAVDDLIIPWSELVLKEKIGAGSFGTVHRADWHGSDVAVKILME
530
LIEG Q LR TVSDLSLA DDLIIPW+EL+LKEKIGAGSFGTVH
ADWHGSDVAVKILME
Sbjct 471
LIEGTQQLRHTVSDLSLAADDLIIPWNELILKEKIGAGSFGTVHHADWHGSDVAVKILME
530

Query 531
QDYHLDRFKEFMREVAIMKSLRHPNIVLFMGAVTEPPNLSIVTEYLSRGSLYKLLHRSGA
590
QD H

+RF+EFMREVAIMKSLRHPNIVLFMGAVTEPPNLSIVTEYLSRGSLYKLLHRSGA
Sbjct 531
QDLHPERFREFMREVAIMKSLRHPNIVLFMGAVTEPPNLSIVTEYLSRGSLYKLLHRSGA
590

Query 591
REVLDERRRLNMAFDVAKGMNYLHRRSPPIVHRDLKSPNLLVDKKYTVKVCDFGLSRLK
A 650

+EVLDERRRLNMAFDVAKGMNYLHRRSPPIVHRDLKSPNLLVDKKYTVKVCDFGLSRLK
A
Sbjct 591
KEVLDERRRLNMAFDVAKGMNYLHRRSPPIVHRDLKSPNLLVDKKYTVKVCDFGLSRLK
A 650

Query 651
NTYLSSKSLAGTPEWMAPEVLRDEPSNEKSDVYSFAVILWELMTLQQPWCNLNPAQVVA
A 710
NT+LSSKSLAGTPEWMAPEVLRDEPSNEKSDVYSF
VILWELMTLQQPWCNLNPAQVVAA
Sbjct 651
NTFLSSKSLAGTPEWMAPEVLRDEPSNEKSDVYSFGVILWELMTLQQPWCNLNPAQVVA
A 710

Query 711
VGFKGRRLEIPKELNPQVAALIESCWANEPWRRPSFANIMETLRPLINKVPVPQLIRSDS
770
VGFKGRRLEIPK+LNP VA LIESCWANEPWRRPSFANIM+TL+PLI
KVPVPQL+RSDS
Sbjct 711
VGFKGRRLEIPKDLNPLVATLIESCWANEPWRRPSFANIMDTLKPLI-KVPVPQLLRSDS
769

>hypothetical protein HU200_039538 [Digitaria exilis]
Sequence ID: KAF8692704.1 Length: 762
Range 1: 1 to 762

Score:1199 bits(3102), Expect:0.0,
Method:Compositional matrix adjust.,
Identities:651/771(84%), Positives:698/771(90%), Gaps:10/771(1%)

Query 1
MELPAAGGGRRTSYSLLSQFPDDAAAAGASPA-VLQRQSSGGSSYGAGSSVSASSDYPFH
59
M+LP G RR++YSLLSQFPDDAA A A VLQRQSSG S
GAGSS+SASSDYPFH
Sbjct 1
MDLPP-GAARRSTYSLLSQFPDDAAGPAAPAANVLQRQSSGSSY-GAGSSISASSDYPFH
58

Query 60
LPPAVAAAGGGGGTPSPCKSWAQQAEETYQLQLALALRLCADAACAADPGFLDPGDSGG
S 119
L P +AA G
SPCKSWAQQAEETYQLQLALALRLCADAACAADPGFLDPGD G
Sbjct 59
LAPPASAAAAAPGGSSPCKSWAQQAEETYQLQLALALRLCADAACAADPGFLDPGDPGS
G 118

Query 120
KMGGGGGGSGSGRAFPLAPPSPTAEALSHRFWVNGSLSYSNTIPDGFYLIQGMDPFVWSM
179
+ SG+GRAFPLA P+PTAE+LSHRFWVNGSLSY++TIPDGFY+I
GMDPFVWS+
Sbjct 119
RG------SGNGRAFPLAQPAPTAESLSHRFWVNGSLSYNSTIPDGFYVIHGMDPFVWSL 172

Query 180
CTDVHEENRIPSVESLKSVRPDDSSIQVVLVDRRADFDLGMLENYASSFLSSSSDMKDVI
239
CTDVHEENRIPS+ESLKSVRPDDS IQ +L+DRR DFDLGMLE+YASS LSS +D
KDV+
Sbjct 173
CTDVHEENRIPSMESLKSVRPDDSPIQAILIDRRTDFDLGMLESYASSLLSSCTDAKDVV
232

Query 240
NQLAKLVSSRMGGTTSNEENLLPRWKESSEAIKSSAGSIVLHLGKLPIGLCKHRSLLFKM
299
QLAK+VSSRMGGTTSNE+NLLPRWKE +EAIKSS
GS+VLHLGKLP+GLCKHRSLLFKM
Sbjct 233
IQLAKVVSSRMGGTTSNEDNLLPRWKECNEAIKSSTGSVVLHLGKLPVGLCKHRSLLFKM
292

Query 300
LADKVNIPCRLVKGCKYCKAEDASSCVVRFGLEREYLVDLFGAPGQLSDPDSFVNGPYSL
359
LADKVNIPCR+VKGCKYCK++DASSC+VRFGLEREYLVDL G
PGQLSDPDSFVNGPYSL
Sbjct 293
LADKVNIPCRVVKGCKYCKSDDASSCLVRFGLEREYLVDLIGDPGQLSDPDSFVNGPYSL
352

Query 360
SVPSPLRPPKFRSLEITSNFSSVAKQYFSDCHSLNLLFSDASTGASNGAAVAVDQMYSKK
419
SV SPL PPKFRSLEITSNFSSVAKQYFSDCHSLNLLFSD+STGA+ A V++DQ
YSKK
Sbjct 353
SVSSPLHPPKFRSLEITSNFSSVAKQYFSDCHSLNLLFSDSSTGAATSAVVSLDQPYSKK
412

Query 420
HDAGDGIANSWVPVKGQAIANSDIILPEAPREVLPLMSPSNLTADKKKEFQLIEGNQYLR
479
+ AGD NSW+P KGQA DII+PEAPREVLPL++ SN+ DKKKE +LIE
Q+LR
Sbjct 413
YVAGDENINSWMPGKGQASMKPDIIVPEAPREVLPLITSSNMKLDKKKELKLIEETQHLR
472

Query 480
STVSDLSLAVDDLIIPWSELVLKEKIGAGSFGTVHRADWHGSDVAVKILMEQDYHLDRFK
539
TVSDLSLA
DDLIIPW+ELVLKEKIGAGSFGTVHRADWHGSDVAVKILMEQD+H +RF+
Sbjct 473
HTVSDLSLAADDLIIPWNELVLKEKIGAGSFGTVHRADWHGSDVAVKILMEQDFHPERFR
532

Query 540
EFMREVAIMKSLRHPNIVLFMGAVTEPPNLSIVTEYLSRGSLYKLLHRSGAREVLDERRR
599

EFMREVAIMKSLRHPNIVLFMGAVTEPPNLSIVTEYLSRGSLYKLLHRSGAREVLDERRR
Sbjct 533
EFMREVAIMKSLRHPNIVLFMGAVTEPPNLSIVTEYLSRGSLYKLLHRSGAREVLDERRR
592

Query 600
LNMAFDVAKGMNYLHRRSPPIVHRDLKSPNLLVDKKYTVKVCDFGLSRLKANTYLSSKS
L 659

LNMAFDVAKGMNYLHRRSPPIVHRDLKSPNLLVDKKYTVKVCDFGLSRLKANT+LSSKS
L
Sbjct 593
LNMAFDVAKGMNYLHRRSPPIVHRDLKSPNLLVDKKYTVKVCDFGLSRLKANTFLSSKSL
652

Query 660
AGTPEWMAPEVLRDEPSNEKSDVYSFAVILWELMTLQQPWCNLNPAQVVAAVGFKGRRL
E 719
AGTPEWMAPEVLRDEPSNEKSDVYSF
VILWELMTLQQPWCNLNPAQVVAAVGFKGRRLE
Sbjct 653
AGTPEWMAPEVLRDEPSNEKSDVYSFGVILWELMTLQQPWCNLNPAQVVAAVGFKGRRL
E 712

Query 720 IPKELNPQVAALIESCWANEPWRRPSFANIMETLRPLINKVPVPQLIRSDS
770
IPK+LNP VAALIESCWANEPWRRPSF NIMETLRPLI KVPVPQL+RSDS
Sbjct 713 IPKDLNPLVAALIESCWANEPWRRPSFTNIMETLRPLI-KVPVPQLVRSDS
762

>hypothetical protein GUJ93_ZPchr0009g1750 [Zizania palustris]
Sequence ID: KAG8050273.1 Length: 752
Range 1: 1 to 752

Score:1196 bits(3095), Expect:0.0,
Method:Compositional matrix adjust.,
Identities:631/773(82%), Positives:685/773(88%), Gaps:24/773(3%)

Query 1
MELPAAGGGRRTSYSLLSQFPDDAAAAGASPAVLQRQSSGGSSYGAGSSVSASSDYPFHL
60
M+LP GGRRT+YSLLSQ P+DAA V+QR SSG S
GAGSS+SASS+YPFHL
Sbjct 1
MDLPV--GGRRTTYSLLSQLPEDAA-------VVQRHSSGSSY-GAGSSLSASSEYPFHL 50

Query 61
PPAVAAAGGGGGTPS---PCKSWAQQAEETYQLQLALALRLCADAACAADPGFLDPGDSG
117
P A + G +
PCKSWAQQAEETYQLQLALALRLCADAAC+ADPGFLD
Sbjct 51
PATAAGSTAAAGAGAGGSPCKSWAQQAEETYQLQLALALRLCADAACSADPGFLD-----
105

Query 118
GSKMGGGGGGSGSGRAFPLAPPSPTAEALSHRFWVNGSLSYSNTIPDGFYLIQGMDPFVW
177
GG GSGSGRAFPL P P+AE+LSHRFWVNGSLSYSNT+PDGFYLI
GMDPFVW
Sbjct 106
-----TGGSGSGSGRAFPLPPLIPSAESLSHRFWVNGSLSYSNTVPDGFYLIHGMDPFVW 160

Query 178
SMCTDVHEENRIPSVESLKSVRPDDSSIQVVLVDRRADFDLGMLENYASSFLSSSSDMKD
237
S+CTDV EENRIPS+ESLKSVRPDD SIQ +L+DRR
DFDLGMLENYASSFLSSSSDM+D
Sbjct 161
SLCTDVQEENRIPSLESLKSVRPDDCSIQAILIDRRTDFDLGMLENYASSFLSSSSDMRD
220

Query 238
VINQLAKLVSSRMGGTTSNEENLLPRWKESSEAIKSSAGSIVLHLGKLPIGLCKHRSLLF
297
VINQLAKLVSSRMGGT SNE+ LP WKE +AIKSS GSIVLHLGKLPIG
CKHRSLLF
Sbjct 221
VINQLAKLVSSRMGGTASNEDTFLPYWKECGDAIKSSTGSIVLHLGKLPIGFCKHRSLLF
280

Query 298
KMLADKVNIPCRLVKGCKYCKAEDASSCVVRFGLEREYLVDLFGAPGQLSDPDSFVNGP
Y 357
K+LADKVN+PCR+VKGCKYCK++DA+SC+VRFGLEREYLVDL G PG
LSDPDSF+NGPY
Sbjct 281
KILADKVNVPCRVVKGCKYCKSDDATSCLVRFGLEREYLVDLIGDPGHLSDPDSFLNGPY
340

Query 358
SLSVPSPLRPPKFRSLEITSNFSSVAKQYFSDCHSLNLLFSDASTGASNGAAVAVDQMYS
417
SLSVPSPLRPPKFRSLEITSNFSSVAKQYFSDCHSLNLLF++ STGA +
AVAVDQ+Y
Sbjct 341
SLSVPSPLRPPKFRSLEITSNFSSVAKQYFSDCHSLNLLFNETSTGAVSSTAVAVDQLYY
400

Query 418
KKHDAGDGIANSWVPVKGQAIANSDIILPEAPREVLPLMSPSNLTADKKKEFQLIEGNQY
477
KKHD+ D I +SWVPVKGQA NSD ILPEAPREVLPL++ +NL A K
EF+LIEGNQ
Sbjct 401
KKHDSRDDIMSSWVPVKGQAAVNSDAILPEAPREVLPLITSANLEAGKNNEFKLIEGNQQ
460

Query 478
LRSTVSDLSLAVDDLIIPWSELVLKEKIGAGSFGTVHRADWHGSDVAVKILMEQDYHLDR
537
LRSTVS S
AVDDLIIPW+ELVLKEKIGAGSFGTVHRADW+GSDVAVKILMEQD+H D
Sbjct 461
LRSTVSSFSFAVDDLIIPWNELVLKEKIGAGSFGTVHRADWNGSDVAVKILMEQDFHPDH
520

Query 538
FKEFMREVAIMKSLRHPNIVLFMGAVTEPPNLSIVTEYLSRGSLYKLLHRSGAREVLDER
597

F+EFMREVAIMKSLRHPNIVLFMGAVTEPPNLSI+TEYLSRGSLYKLLHRSGA+EVLDER
Sbjct 521
FREFMREVAIMKSLRHPNIVLFMGAVTEPPNLSIITEYLSRGSLYKLLHRSGAKEVLDER
580

Query 598
RRLNMAFDVAKGMNYLHRRSPPIVHRDLKSPNLLVDKKYTVKVCDFGLSRLKANTYLSS
K 657

RRLNMAFDVAKGMNYLH+R+PPIVHRDLKSPNLLVDKKYTVK+CDFGLSRLKANT+LSSK
Sbjct 581
RRLNMAFDVAKGMNYLHKRNPPIVHRDLKSPNLLVDKKYTVKICDFGLSRLKANTFLSS

K 640

Query 658
SLAGTPEWMAPEVLRDEPSNEKSDVYSFAVILWELMTLQQPWCNLNPAQVVAAVGFKGR
R 717
SLAGTPEWMAPEVLRDEPSNEKSDVYSF VILWE+MT+QQPW
N+NPAQVVAAVGFKGRR
Sbjct 641
SLAGTPEWMAPEVLRDEPSNEKSDVYSFGVILWEIMTMQQPWFNMNPAQVVAAVGFKGR
R 700

Query 718 LEIPKELNPQVAALIESCWANEPWRRPSFANIMETLRPLINKVPVPQLIRSDS
770
L+IPK+LNPQV ALIE CWANEPWRRPSFANIME+LR I KVP+PQLIRSDS
Sbjct 701 LDIPKDLNPQVTALIELCWANEPWRRPSFANIMESLRS-ITKVPLPQLIRSDS
752

>uncharacterized protein LOC101754395 [Setaria italica]
Sequence ID: XP_012699069.1 Length: 1851
Range 1: 1 to 770

Score:1190 bits(3078), Expect:0.0,
Method:Compositional matrix adjust.,
Identities:641/780(82%), Positives:691/780(88%), Gaps:20/780(2%)

Query 1
MELPAAGGGRRTSYSLLSQFPDDAAAAGASPAVLQRQSSGGSSYGAGSSVSASSDYPFHL
60
M+LP G RRT+YSLLSQFPDDA + VLQRQSSG S
SS+SASSDYPFHL
Sbjct 1
MDLPP-GAARRTTYSLLSQFPDDAPGPPPA-TVLQRQSSGSSYGAG-SSISASSDYPFHL 57

Query 61
PPAVAAAG----------GGGGTPSPCKSWAQQAEETYQLQLALALRLCADAACAADPGF 110
+AAG G
SPCKSWAQQAEETYQLQLALALRLCADAACAADPGF
Sbjct 58
QAPASAAGVAPPGSAAAAAAPGGSSPCKSWAQQAEETYQLQLALALRLCADAACAADP
GF 117

Query 111
LDPGDSGGSKMGGGGGGSGSGRAFPLAPPSPTAEALSHRFWVNGSLSYSNTIPDGFYLIQ
170
LDPG++G + SG+GRAFPLA P+P+
E+LSHRFWVNGSLSY++TIPDGFY+I
Sbjct 118
LDPGETGSGRG------SGNGRAFPLAHPAPSPESLSHRFWVNGSLSYNSTIPDGFYVIH 171

Query 171
GMDPFVWSMCTDVHEENRIPSVESLKSVRPDDSSIQVVLVDRRADFDLGMLENYASSFLS
230
GMDPFVWS+CTD+HEENRIPS+ESLKSV PDDSSIQ +L+DRR
DFDLGMLE+YASS S
Sbjct 172
GMDPFVWSLCTDIHEENRIPSMESLKSVLPDDSSIQAILIDRRTDFDLGMLESYASSLSS
231

Query 231
SSSDMKDVINQLAKLVSSRMGGTTSNEENLLPRWKESSEAIKSSAGSIVLHLGKLPIGLC
290
S +D KDV+ QLAK+VSSRMGGTTSNEENLLPRWKE
+EAIKSSAGS+VLHLGKLP+GLC
Sbjct 232
SCTDAKDVVTQLAKVVSSRMGGTTSNEENLLPRWKECNEAIKSSAGSVVLHLGKLPVGL
C 291

Query 291
KHRSLLFKMLADKVNIPCRLVKGCKYCKAEDASSCVVRFGLEREYLVDLFGAPGQLSDP
D 350
KHRSLLFKMLADKV+IPCR+VKGCKYCK++DASSC+VRFGLEREYLVDL G
PGQLSDPD
Sbjct 292
KHRSLLFKMLADKVSIPCRVVKGCKYCKSDDASSCLVRFGLEREYLVDLIGDPGQLSDPD
351

Query 351
SFVNGPYSLSVPSPLRPPKFRSLEITSNFSSVAKQYFSDCHSLNLLFSDASTGASNGAAV
410
SFVNGPYSLSV SPL PPKFRSLEITSNFSSVAKQYFSDCHSLNLLFSD+STGA+
A
Sbjct 352
SFVNGPYSLSVSSPLHPPKFRSLEITSNFSSVAKQYFSDCHSLNLLFSDSSTGAATSAVT
411

Query 411
AVDQMYSKKHDAGDGIANSWVPVKGQAIANSDIILPEAPREVLPLMSPSNLTADKKKEFQ
470
+DQ YSKKH AGD I NSW+P KGQA DII+PEAPREVLPL++ SN+
DKKKE +
Sbjct 412
TLDQPYSKKHIAGDEIINSWMPGKGQAAIKPDIIVPEAPREVLPLITSSNIKLDKKKELK
471

Query 471
LIEGNQYLRSTVSDLSLAVDDLIIPWSELVLKEKIGAGSFGTVHRADWHGSDVAVKILME
530
LIE Q+LR TVSDLSLA
DDLIIPW+EL+LKEKIGAGSFGTVHRADWHGSDVAVKILME
Sbjct 472
LIEETQHLRHTVSDLSLAADDLIIPWNELILKEKIGAGSFGTVHRADWHGSDVAVKILME
531

Query 531
QDYHLDRFKEFMREVAIMKSLRHPNIVLFMGAVTEPPNLSIVTEYLSRGSLYKLLHRSGA
590
QD+H
+RF+EFMREVAIMKSLRHPNIVLFMGAVTEPPNLSIVTEYLSRGSLYKLLHRSGA
Sbjct 532
QDFHPERFREFMREVAIMKSLRHPNIVLFMGAVTEPPNLSIVTEYLSRGSLYKLLHRSGA
591

Query 591
REVLDERRRLNMAFDVAKGMNYLHRRSPPIVHRDLKSPNLLVDKKYTVKVCDFGLSRLK
A 650

+EVLDERRRLNMAFDVAKGMNYLHRRSPPIVHRDLKSPNLLVDKKYTVKVCDFGLSRLK
A
Sbjct 592
KEVLDERRRLNMAFDVAKGMNYLHRRSPPIVHRDLKSPNLLVDKKYTVKVCDFGLSRLK
A 651

Query 651
NTYLSSKSLAGTPEWMAPEVLRDEPSNEKSDVYSFAVILWELMTLQQPWCNLNPAQVVA
A 710
NT+LSSKSLAGTPEWMAPEVLRDEPSNEKSDVYSF
VILWELMTLQQPWCNLNPAQVVAA
Sbjct 652
NTFLSSKSLAGTPEWMAPEVLRDEPSNEKSDVYSFGVILWELMTLQQPWCNLNPAQVVA

A 711

Query 711
VGFKGRRLEIPKELNPQVAALIESCWANEPWRRPSFANIMETLRPLINKVPVPQLIRSDS
770
VGFKGRRLEIPK+LNP VAALIESCWANEPWRRPSF NIME LRPLI
KVPVPQLIRSDS
Sbjct 712
VGFKGRRLEIPKDLNPLVAALIESCWANEPWRRPSFTNIMEALRPLI-KVPVPQLIRSDS
770

>uncharacterized protein LOC117844130 [Setaria viridis]
Sequence ID: XP_034580787.1 Length: 1851
Range 1: 1 to 770

Score:1189 bits(3077), Expect:0.0,
Method:Compositional matrix adjust.,
Identities:641/780(82%), Positives:691/780(88%), Gaps:20/780(2%)

Query 1
MELPAAGGGRRTSYSLLSQFPDDAAAAGASPAVLQRQSSGGSSYGAGSSVSASSDYPFHL
60
M+LP G RRT+YSLLSQFPDDA + VLQRQSSG S
SS+SASSDYPFHL
Sbjct 1
MDLPP-GAARRTTYSLLSQFPDDAPGPPPA-TVLQRQSSGSSYGAG-SSISASSDYPFHL 57

Query 61
PPAVAAAG----------GGGGTPSPCKSWAQQAEETYQLQLALALRLCADAACAADPGF 110
+AAG G
SPCKSWAQQAEETYQLQLALALRLCADAACAADPGF
Sbjct 58
QAPASAAGVAPPGSAAAAAAPGGSSPCKSWAQQAEETYQLQLALALRLCADAACAADP
GF 117

Query 111
LDPGDSGGSKMGGGGGGSGSGRAFPLAPPSPTAEALSHRFWVNGSLSYSNTIPDGFYLIQ
170
LDPG++G + SG+GRAFPLA P+P+
E+LSHRFWVNGSLSY++TIPDGFY+I
Sbjct 118

LDPGETGSGRG------SGNGRAFPLAHPAPSPESLSHRFWVNGSLSYNSTIPDGFYVIH 171

Query 171
GMDPFVWSMCTDVHEENRIPSVESLKSVRPDDSSIQVVLVDRRADFDLGMLENYASSFLS
230
GMDPFVWS+CTD+HEENRIPS+ESLKSV PDDSSIQ +L+DRR
DFDLGMLE+YASS S
Sbjct 172
GMDPFVWSLCTDIHEENRIPSMESLKSVLPDDSSIQAILIDRRTDFDLGMLESYASSLSS
231

Query 231
SSSDMKDVINQLAKLVSSRMGGTTSNEENLLPRWKESSEAIKSSAGSIVLHLGKLPIGLC
290
S +D KDV+ QLAK+VSSRMGGTTSNEENLLPRWKE
+EAIKSSAGS+VLHLGKLP+GLC
Sbjct 232
SCTDAKDVVTQLAKVVSSRMGGTTSNEENLLPRWKECNEAIKSSAGSVVLHLGKLPVGL
C 291

Query 291
KHRSLLFKMLADKVNIPCRLVKGCKYCKAEDASSCVVRFGLEREYLVDLFGAPGQLSDP
D 350
KHRSLLFKMLADKV+IPCR+VKGCKYCK++DASSC+VRFGLEREYLVDL G
PGQLSDPD
Sbjct 292
KHRSLLFKMLADKVSIPCRVVKGCKYCKSDDASSCLVRFGLEREYLVDLIGDPGQLSDPD
351

Query 351
SFVNGPYSLSVPSPLRPPKFRSLEITSNFSSVAKQYFSDCHSLNLLFSDASTGASNGAAV
410
SFVNGPYSLSV SPL PPKFRSLEITSNFSSVAKQYFSDCHSLNLLFSD+STGA+
A
Sbjct 352
SFVNGPYSLSVSSPLHPPKFRSLEITSNFSSVAKQYFSDCHSLNLLFSDSSTGAATSAVT
411

Query 411
AVDQMYSKKHDAGDGIANSWVPVKGQAIANSDIILPEAPREVLPLMSPSNLTADKKKEFQ
470
+DQ YSKKH AGD I NSW+P KGQA DII+PEAPREVLPL++ SN+
DKKKE +
Sbjct 412

TLDQPYSKKHIAGDEIINSWMPGKGQAAIKPDIIVPEAPREVLPLITSSNIKLDKKKELK
471

Query 471
LIEGNQYLRSTVSDLSLAVDDLIIPWSELVLKEKIGAGSFGTVHRADWHGSDVAVKILME
530
LIE Q+LR TVSDLSLA
DDLIIPW+EL+LKEKIGAGSFGTVHRADWHGSDVAVKILME
Sbjct 472
LIEETQHLRHTVSDLSLAADDLIIPWNELILKEKIGAGSFGTVHRADWHGSDVAVKILME
531

Query 531
QDYHLDRFKEFMREVAIMKSLRHPNIVLFMGAVTEPPNLSIVTEYLSRGSLYKLLHRSGA
590
QD+H
+RF+EFMREVAIMKSLRHPNIVLFMGAVTEPPNLSIVTEYLSRGSLYKLLHRSGA
Sbjct 532
QDFHPERFREFMREVAIMKSLRHPNIVLFMGAVTEPPNLSIVTEYLSRGSLYKLLHRSGA
591

Query 591
REVLDERRRLNMAFDVAKGMNYLHRRSPPIVHRDLKSPNLLVDKKYTVKVCDFGLSRLK
A 650

+EVLDERRRLNMAFDVAKGMNYLHRRSPPIVHRDLKSPNLLVDKKYTVKVCDFGLSRLK
A
Sbjct 592
KEVLDERRRLNMAFDVAKGMNYLHRRSPPIVHRDLKSPNLLVDKKYTVKVCDFGLSRLK
A 651

Query 651
NTYLSSKSLAGTPEWMAPEVLRDEPSNEKSDVYSFAVILWELMTLQQPWCNLNPAQVVA
A 710
NT+LSSKSLAGTPEWMAPEVLRDEPSNEKSDVYSF
VILWELMTLQQPWCNLNPAQVVAA
Sbjct 652
NTFLSSKSLAGTPEWMAPEVLRDEPSNEKSDVYSFGVILWELMTLQQPWCNLNPAQVVA
A 711

Query 711
VGFKGRRLEIPKELNPQVAALIESCWANEPWRRPSFANIMETLRPLINKVPVPQLIRSDS
770
VGFKGRRLEIPK+LNP VAALIESCWANEPWRRPSF NIME LRPLI

KVPVPQLIRSDS
Sbjct 712
VGFKGRRLEIPKDLNPLVAALIESCWANEPWRRPSFTNIMEALRPLI-KVPVPQLIRSDS
770

>hypothetical protein SETIT_2G132000v2 [Setaria italica]
Sequence ID: RCV10728.1 Length: 770
>hypothetical protein SEVIR_2G137201v2 [Setaria viridis]
Sequence ID: TKW31909.1 Length: 770
Range 1: 1 to 770

Score:1184 bits(3064), Expect:0.0,
Method:Compositional matrix adjust.,
Identities:644/780(83%), Positives:694/780(88%), Gaps:20/780(2%)

Query 1
MELPAAGGGRRTSYSLLSQFPDDAAAAGASPAVLQRQSSGGSSYGAGSSVSASSDYPFHL
60
M+LP G RRT+YSLLSQFPDDA + VLQRQSSG S
GAGSS+SASSDYPFHL
Sbjct 1
MDLPP-GAARRTTYSLLSQFPDDAPGPPPA-TVLQRQSSGSSY-GAGSSISASSDYPFHL 57

Query 61
PPAVAAAG----------GGGGTPSPCKSWAQQAEETYQLQLALALRLCADAACAADPGF 110
+AAG G
SPCKSWAQQAEETYQLQLALALRLCADAACAADPGF
Sbjct 58
QAPASAAGVAPPGSAAAAAAPGGSSPCKSWAQQAEETYQLQLALALRLCADAACAADP
GF 117

Query 111
LDPGDSGGSKMGGGGGGSGSGRAFPLAPPSPTAEALSHRFWVNGSLSYSNTIPDGFYLIQ
170
LDPG++G + SG+GRAFPLA P+P+
E+LSHRFWVNGSLSY++TIPDGFY+I
Sbjct 118
LDPGETGSGRG------SGNGRAFPLAHPAPSPESLSHRFWVNGSLSYNSTIPDGFYVIH 171

Query 171
GMDPFVWSMCTDVHEENRIPSVESLKSVRPDDSSIQVVLVDRRADFDLGMLENYASSFLS

230
GMDPFVWS+CTD+HEENRIPS+ESLKSV PDDSSIQ +L+DRR DFDLGMLE+YASS S
Sbjct 172
GMDPFVWSLCTDIHEENRIPSMESLKSVLPDDSSIQAILIDRRTDFDLGMLESYASSLSS
231

Query 231
SSSDMKDVINQLAKLVSSRMGGTTSNEENLLPRWKESSEAIKSSAGSIVLHLGKLPIGLC
290
S +D KDV+ QLAK+VSSRMGGTTSNEENLLPRWKE +EAIKSSAGS+VLHLGKLP+GLC
Sbjct 232
SCTDAKDVVTQLAKVVSSRMGGTTSNEENLLPRWKECNEAIKSSAGSVVLHLGKLPVGL
C 291

Query 291
KHRSLLFKMLADKVNIPCRLVKGCKYCKAEDASSCVVRFGLEREYLVDLFGAPGQLSDP
D 350
KHRSLLFKMLADKV+IPCR+VKGCKYCK++DASSC+VRFGLEREYLVDL G PGQLSDPD
Sbjct 292
KHRSLLFKMLADKVSIPCRVVKGCKYCKSDDASSCLVRFGLEREYLVDLIGDPGQLSDPD
351

Query 351
SFVNGPYSLSVPSPLRPPKFRSLEITSNFSSVAKQYFSDCHSLNLLFSDASTGASNGAAV
410
SFVNGPYSLSV SPL PPKFRSLEITSNFSSVAKQYFSDCHSLNLLFSD+STGA+ A
Sbjct 352
SFVNGPYSLSVSSPLHPPKFRSLEITSNFSSVAKQYFSDCHSLNLLFSDSSTGAATSAVT
411

Query 411
AVDQMYSKKHDAGDGIANSWVPVKGQAIANSDIILPEAPREVLPLMSPSNLTADKKKEFQ
470
+DQ YSKKH AGD I NSW+P KGQA DII+PEAPREVLPL++ SN+ DKKKE +
Sbjct 412
TLDQPYSKKHIAGDEIINSWMPGKGQAAIKPDIIVPEAPREVLPLITSSNIKLDKKKELK
471

Query 471

LIEGNQYLRSTVSDLSLAVDDLIIPWSELVLKEKIGAGSFGTVHRADWHGSDVAVKILME
530

LIE Q+LR TVSDLSLA
DDLIIPW+EL+LKEKIGAGSFGTVHRADWHGSDVAVKILME

Sbjct 472
LIEETQHLRHTVSDLSLAADDLIIPWNELILKEKIGAGSFGTVHRADWHGSDVAVKILME
531

Query 531
QDYHLDRFKEFMREVAIMKSLRHPNIVLFMGAVTEPPNLSIVTEYLSRGSLYKLLHRSGA
590

QD+H
+RF+EFMREVAIMKSLRHPNIVLFMGAVTEPPNLSIVTEYLSRGSLYKLLHRSGA

Sbjct 532
QDFHPERFREFMREVAIMKSLRHPNIVLFMGAVTEPPNLSIVTEYLSRGSLYKLLHRSGA
591

Query 591
REVLDERRRLNMAFDVAKGMNYLHRRSPPIVHRDLKSPNLLVDKKYTVKVCDFGLSRLK
A 650

+EVLDERRRLNMAFDVAKGMNYLHRRSPPIVHRDLKSPNLLVDKKYTVKVCDFGLSRLK
A

Sbjct 592
KEVLDERRRLNMAFDVAKGMNYLHRRSPPIVHRDLKSPNLLVDKKYTVKVCDFGLSRLK
A 651

Query 651
NTYLSSKSLAGTPEWMAPEVLRDEPSNEKSDVYSFAVILWELMTLQQPWCNLNPAQVVA
A 710

NT+LSSKSLAGTPEWMAPEVLRDEPSNEKSDVYSF
VILWELMTLQQPWCNLNPAQVVAA

Sbjct 652
NTFLSSKSLAGTPEWMAPEVLRDEPSNEKSDVYSFGVILWELMTLQQPWCNLNPAQVVA
A 711

Query 711
VGFKGRRLEIPKELNPQVAALIESCWANEPWRRPSFANIMETLRPLINKVPVPQLIRSDS
770

VGFKGRRLEIPK+LNP VAALIESCWANEPWRRPSF NIME LRPLI
KVPVPQLIRSDS

Sbjct 712
VGFKGRRLEIPKDLNPLVAALIESCWANEPWRRPSFTNIMEALRPLI-KVPVPQLIRSDS
770

>uncharacterized protein LOC120662481 [Panicum virgatum]
Sequence ID: XP_039797554.1 Length: 1896
Range 1: 1 to 767

Score:1180 bits(3052), Expect:0.0,
Method:Compositional matrix adjust.,
Identities:642/777(83%), Positives:693/777(89%), Gaps:17/777(2%)

Query 1
MELPAAGGGRRTSYSLLSQFPDDAAAAGASPA-VLQRQSSGGSSYGAGSSVSASSDYPFH
59
MELP G RRT+YSLLSQFPDDAAAAG PA LQRQSSG S G
SSVSASSDYPFH
Sbjct 1
MELPP-GAARRTNYSLLSQFPDDAAAAGPGPANALQRQSSGSSYGGG-SSVSASSDYPFH
58

Query 60
LPPAVAAAGGGGGTPSP------CKSWAQQAEETYQLQLALALRLCADAACAADPGFLDP
113
LPP +AA G G +
CKSWAQQAEETYQLQLALALRLCADAACAADPGFLD
Sbjct 59
LPPPASAAAGPGSAAAAPGGSSPCKSWAQQAEETYQLQLALALRLCADAACAADPGFLD-
117

Query 114
GDSGGSKMGGGGGGSGSGRAFPLAPPSPTAEALSHRFWVNGSLSYSNTIPDGFYLIQGMD
173
G GG GSG+GRAFPLA P+P+AE+LSHRFWVNGSLSY++TIPDGFY+I
G+D
Sbjct 118
----PGDPGSGGRGSGNGRAFPLAQPAPSAESLSHRFWVNGSLSYNSTIPDGFYVIHGLD
173

Query 174
PFVWSMCTDVHEENRIPSVESLKSVRPDDSSIQVVLVDRRADFDLGMLENYASSFLSSSS
233
PFVWS+CTDVHEENRIPS+ESLKSVRPDDSSIQ +L+DRR DFDLGMLE+YASS
LSS +

Sbjct 174
PFVWSLCTDVHEENRIPSMESLKSVRPDDSSIQAILIDRRTDFDLGMLESYASSLLSSCT
233

Query 234
DMKDVINQLAKLVSSRMGGTTSNEENLLPRWKESSEAIKSSAGSIVLHLGKLPIGLCKHR
293
D KDV+ QLAK+VSSRMGGT SNE+NL PRWKE +EAIKSS
GS+VLHLGKLP+GLCKHR
Sbjct 234
DAKDVVIQLAKVVSSRMGGTASNEDNLFPRWKECNEAIKSSTGSVVLHLGKLPVGLCKH
R 293

Query 294
SLLFKMLADKVNIPCRLVKGCKYCKAEDASSCVVRFGLEREYLVDLFGAPGQLSDPDSFV
353
SLLFK+LADKV+IPCR+VKGCKYCK++DASSC+VRFGLE+EYLVDL G
PGQLSDPDSFV
Sbjct 294
SLLFKILADKVSIPCRVVKGCKYCKSDDASSCLVRFGLEKEYLVDLIGDPGQLSDPDSFV
353

Query 354
NGPYSLSVPSPLRPPKFRSLEITSNFSSVAKQYFSDCHSLNLLFSDASTGASNGAAVAVD
413
NGPYSLSV SPL PPKFRSLEITSNFSSVAKQYFSDCHSLNLLFSD+STGA++
+D
Sbjct 354
NGPYSLSVSSPLHPPKFRSLEITSNFSSVAKQYFSDCHSLNLLFSDSSTGATS-VITTLD 412

Query 414
QMYSKKHDAGDGIANSWVPVKGQAIANSDIILPEAPREVLPLMSPSNLTADKKKEFQLIE
473
Q YSKKH AGD N+W+P KGQA DI++PEAPREVLPL+S SN+ D+KKE
+LIE
Sbjct 413
QPYSKKHVAGDETMNNWMPGKGQAAMKPDIVVPEAPREVLPLIS-SNMKLDRKKELKLI
E 471

Query 474
GNQYLRSTVSDLSLAVDDLIIPWSELVLKEKIGAGSFGTVHRADWHGSDVAVKILMEQDY
533
Q LR TVSDLSLA
+DLIIPW+ELVLKEKIGAGSFGTVHRADWHGSDVAVKILMEQD+

Sbjct 472
ETQQLRHTVSDLSLAANDLIIPWNELVLKEKIGAGSFGTVHRADWHGSDVAVKILMEQDF
531

Query 534
HLDRFKEFMREVAIMKSLRHPNIVLFMGAVTEPPNLSIVTEYLSRGSLYKLLHRSGAREV
593
H
+RF+EFMREVAIMKSLRHPNIVLFMGAVTEPPNLSIVTEYLSRGSLYKLLHRSGA+EV
Sbjct 532
HPERFREFMREVAIMKSLRHPNIVLFMGAVTEPPNLSIVTEYLSRGSLYKLLHRSGAKEV
591

Query 594
LDERRRLNMAFDVAKGMNYLHRRSPPIVHRDLKSPNLLVDKKYTVKVCDFGLSRLKANT
Y 653

LDERRRLNMAFDVAKGMNYLHRRSPP+VHRDLKSPNLLVDKKYTVKVCDFGLSRLKAN
T+
Sbjct 592
LDERRRLNMAFDVAKGMNYLHRRSPPVVHRDLKSPNLLVDKKYTVKVCDFGLSRLKAN
TF 651

Query 654
LSSKSLAGTPEWMAPEVLRDEPSNEKSDVYSFAVILWELMTLQQPWCNLNPAQVVAAVGF
713
LSSKSLAGTPEWMAPEVLRDEPSNEKSDVYSF
VILWELMTLQQPWCNLNPAQVVAAVGF
Sbjct 652
LSSKSLAGTPEWMAPEVLRDEPSNEKSDVYSFGVILWELMTLQQPWCNLNPAQVVAAVG
F 711

Query 714
KGRRLEIPKELNPQVAALIESCWANEPWRRPSFANIMETLRPLINKVPVPQLIRSDS 770
KGRRLEIPK+LNP VAALIESCWANEPWRRPSF NIMETLRPLI KVPVPQL
RSDS
Sbjct 712
KGRRLEIPKDLNPLVAALIESCWANEPWRRPSFTNIMETLRPLI-KVPVPQLSRSDS 767

>serine/threonine-protein kinase CTR1 [Sorghum bicolor]
Sequence ID: XP_002461985.1 Length: 764

>serine/threonine-protein kinase CTR1 [Sorghum bicolor]
Sequence ID: XP_021309754.1 Length: 764
>hypothetical protein BDA96_02G140100 [Sorghum bicolor]
Sequence ID: KAG0542857.1 Length: 764
>hypothetical protein BDA96_02G140100 [Sorghum bicolor]
Sequence ID: KAG0542858.1 Length: 764
>hypothetical protein SORBI_3002G133400 [Sorghum bicolor]
Sequence ID: OQU89015.1 Length: 764
>hypothetical protein SORBI_3002G133400 [Sorghum bicolor]
Sequence ID: OQU89016.1 Length: 764
>hypothetical protein SORBI_3002G133400 [Sorghum bicolor]
Sequence ID: OQU89017.1 Length: 764
Range 1: 1 to 764

Score:1179 bits(3050), Expect:0.0,
Method:Compositional matrix adjust.,
Identities:634/779(81%), Positives:686/779(88%), Gaps:24/779(3%)

Query 1
MELPAAGGGRRTSYSLLSQFPDDAAAAGASPAVLQRQSSGGSSYGAGSSVSASSDYPFHL
60
M+LP G RRT+YSLL QFPDDAA A A+ VLQRQSSG S S +
SSDYPFHL
Sbjct 1
MDLPP-GAARRTTYSLLPQFPDDAAPAPAN--VLQRQSSGSSYGAGSSIST-SSDYPFHL 56

Query 61
PPAVAAAG---------GGGGTPSPCKSWAQQAEETYQLQLALALRLCADAACAADPGFL 111
P +AAG G
SPCKSWAQQAEETYQLQLALALRLCADAACAADPGFL
Sbjct 57
QPPASAAGVAPPGSAAAPPGAAGSPCKSWAQQAEETYQLQLALALRLCADAACAADPGF
L 116

Query 112
DPGDSGGSKMGGGGGGSGSGRAFPLAPPSPTAEALSHRFWVNGSLSYSNTIPDGFYLIQG
171
DPGDSG + SG+GRAFPLA P+P+AE+LSHRFWVNGSLSY++TI
DGFYLI G
Sbjct 117
DPGDSGSGRG------SGNGRAFPLAQPAPSAESLSHRFWVNGSLSYNSTISDGFYLIHG 170

Query 172
MDPFVWSMCTDVHEENRIPSVESLKSVRPDDSSIQVVLVDRRADFDLGMLENYASSFLSS

231

MDPFVWS+CTDV EENRIPS+ESLKSVRPDDSSIQ +L+DRR DF+LGMLE+YASS LSS

Sbjct 171 MDPFVWSLCTDVQEENRIPSMESLKSVRPDDSSIQAILIDRRTDFELGMLESYASSILSS 230

Query 232 SSDMKDVINQLAKLVSSRMGGTTSNEENLLPRWKESSEAIKSSAGSIVLHLGKLPIGLCK 291

S+D KDV+ QLAKL+SSRMGGTTSNEENLL RWKE EAIKSS GS+VLHLGKLPIGLCK

Sbjct 231 SADAKDVVIQLAKLISSRMGGTTSNEENLLQRWKECIEAIKSSTGSVVLHLGKLPIGLCK 290

Query 292 HRSLLFKMLADKVNIPCRLVKGCKYCKAEDASSCVVRFGLEREYLVDLFGAPGQLSDPDS 351

HRSLLFK+LADKV+IPCR+VKGCKYCK++DASSC+VRFGLERE+LVDL G PGQL+DPDS

Sbjct 291 HRSLLFKVLADKVSIPCRVVKGCKYCKSDDASSCLVRFGLEREFLVDLIGDPGQLTDPDS 350

Query 352 FVNGPYSLSVPSPLRPPKFRSLEITSNFSSVAKQYFSDCHSLNLLFSDASTGASNGAAVA 411

FVNGPYSLSV SPL PPKFRSLEITSNF SVAKQYFSDCHSLNLLFSD+STG +N V+

Sbjct 351 FVNGPYSLSVSSPLHPPKFRSLEITSNFGSVAKQYFSDCHSLNLLFSDSSTGVANSTVVS 410

Query 412 VDQMYSKKHDAGDGIANSWVPVKGQAIANSDIILPEAPREVLPLMSPSNLTADKKKEFQL 471

+D YS+KH AGD + NSWVP KGQAI DI++PEAPREVLPL++ SN+ DKKKE

Sbjct 411 LDHPYSRKHVAGDDVMNSWVPGKGQAIMKPDIMVPEAPREVLPLITSSNVKPDKKKELVT 470

Query 472 IEGNQYLRSTVSDLSLAVDDLIIPWSELVLKEKIGAGSFGTVHRADWHGSDVAVKILMEQ

531

+ LR+TVSDLSLA DDLIIPW+EL+LKEKIGAGSFGTVHRADWHGSDVAVKILMEQ

Sbjct 471 PQ----LRNTVSDLSLAADDLIIPWNELILKEKIGAGSFGTVHRADWHGSDVAVKILMEQ 526

Query 532 DYHLDRFKEFMREVAIMKSLRHPNIVLFMGAVTEPPNLSIVTEYLSRGSLYKLLHRSGAR 591

D+H +RF+EFMREVAIMKSLRHPNIVLFMGAVTEPPNLSIVTEYLSRGSLYKLLHRSGA+

Sbjct 527 DFHPERFREFMREVAIMKSLRHPNIVLFMGAVTEPPNLSIVTEYLSRGSLYKLLHRSGAK 586

Query 592 EVLDERRRLNMAFDVAKGMNYLHRRSPPIVHRDLKSPNLLVDKKYTVKVCDFGLSRLKAN 651

EVLDERRRLNMAFDVAKGMNYLHRRSPPIVHRDLKSPNLLVDKKYTVKVCDFGLSRLKAN

Sbjct 587 EVLDERRRLNMAFDVAKGMNYLHRRSPPIVHRDLKSPNLLVDKKYTVKVCDFGLSRLKAN 646

Query 652 TYLSSKSLAGTPEWMAPEVLRDEPSNEKSDVYSFAVILWELMTLQQPWCNLNPAQVVAAV 711

T+LSSKSLAGTPEWMAPEVLRDEPSNEKSDVYSF VILWELMTLQQPWCNLNPAQVVAAV

Sbjct 647 TFLSSKSLAGTPEWMAPEVLRDEPSNEKSDVYSFGVILWELMTLQQPWCNLNPAQVVAAV 706

Query 712 GFKGRRLEIPKELNPQVAALIESCWANEPWRRPSFANIMETLRPLINKVPVPQLIRSDS 770

GFKGRRLEIPK+LNP VAALIESCWANEPWRRPSFANIM+TLRPLINK P QLIRSDS

Sbjct 707 GFKGRRLEIPKDLNPLVAALIESCWANEPWRRPSFANIMDTLRPLINKGPA-QLIRSDS 764

>hypothetical protein GQ55_2G122400 [Panicum hallii var. hallii]
Sequence ID: PUZ69599.1 Length: 769
Range 1: 1 to 769

Score:1179 bits(3049), Expect:0.0,
Method:Compositional matrix adjust.,
Identities:648/779(83%), Positives:694/779(89%), Gaps:19/779(2%)

Query 1
MELPAAGGGRRTSYSLLSQFPDDAAAAGASPA---VLQRQSSGGSSYGAGSSVSASSDYP
57
MELP G RRT+YSLLSQFPDDAAA G PA VLQRQSSG S
GAGSSVSASSDYP
Sbjct 1
MELPP-GAARRTTYSLLSQFPDDAAAGGPGPAPANVLQRQSSGSSY-GAGSSVSASSDYP
58

Query 58
FHLPPAVAAAGGGGGTPSP------CKSWAQQAEETYQLQLALALRLCADAACAADPGFL
111
FHLPP +AA G G +
CKSWAQQAEETYQLQLALALRLCADAACAADPGFL
Sbjct 59
FHLPPPASAAAGPGSAAAAPGGSSPCKSWAQQAEETYQLQLALALRLCADAACAADPGF
L 118

Query 112
DPGDSGGSKMGGGGGGSGSGRAFPLAPPSPTAEALSHRFWVNGSLSYSNTIPDGFYLIQG
171
D G GG GSG+GRAFPLA
P+P+AE+LSHRFWVNGSLSY++TIPDGFY+I G
Sbjct 119
D-----PGDPGSGGKGSGNGRAFPLAQPAPSAESLSHRFWVNGSLSYNSTIPDGFYVIHG
173

Query 172
MDPFVWSMCTDVHEENRIPSVESLKSVRPDDSSIQVVLVDRRADFDLGMLENYASSFLSS
231
+DPFVWS+CTDVHEENRIP++ESLKSVRPDDSSIQ +L+DRR
DFDLGMLE+YASS LSS
Sbjct 174
LDPFVWSLCTDVHEENRIPTMESLKSVRPDDSSIQAILIDRRTDFDLGMLESYASSLLSS

233

Query 232
SSDMKDVINQLAKLVSSRMGGTTSNEENLLPRWKESSEAIKSSAGSIVLHLGKLPIGLCK
291
+D KDV+ QLAK+VSSRMGGT SNEENL PRWKE +EAIKSS GS+
LHLGKLP+GLCK
Sbjct 234
CTDAKDVVIQLAKVVSSRMGGTASNEENLFPRWKECNEAIKSSTGSVALHLGKLPVGLCK
293

Query 292
HRSLLFKMLADKVNIPCRLVKGCKYCKAEDASSCVVRFGLEREYLVDLFGAPGQLSDPDS
351
HRSLLFKMLADKV+IPCR+VKGCKYCK++DASSC+VRFGLEREYLVDL G
PGQLSDPDS
Sbjct 294
HRSLLFKMLADKVSIPCRVVKGCKYCKSDDASSCLVRFGLEREYLVDLIGDPGQLSDPDS
353

Query 352
FVNGPYSLSVPSPLRPPKFRSLEITSNFSSVAKQYFSDCHSLNLLFSDASTGASNGAAVA
411
FVNGPYSLSV SPL PPKFRSLEITSNFSSVAKQYF+DCHSLNLLFSD+STGA++
A
Sbjct 354
FVNGPYSLSVSSPLHPPKFRSLEITSNFSSVAKQYFADCHSLNLLFSDSSTGATS-AVTT 412

Query 412
VDQMYSKKHDAGDGIANSWVPVKGQAIANSDIILPEAPREVLPLMSPSNLTADKKKEFQL
471
+DQ YSKKH AGD N+W+P KGQA DII+PEAPREVLPL+S SN+
DKKKE +L
Sbjct 413
LDQPYSKKHVAGDETMNNWMPGKGQAAMKPDIIVPEAPREVLPLIS-SNMKLDKKKELK
L 471

Query 472
IEGNQYLRSTVSDLSLAVDDLIIPWSELVLKEKIGAGSFGTVHRADWHGSDVAVKILMEQ
531
IE Q LR TVSDLSLA
DDLIIPW+ELVLKEKIGAGSFGTVHRADWHGSDVAVKILMEQ
Sbjct 472
IEETQQLRHTVSDLSLAADDLIIPWNELVLKEKIGAGSFGTVHRADWHGSDVAVKILMEQ

531

Query 532
DYHLDRFKEFMREVAIMKSLRHPNIVLFMGAVTEPPNLSIVTEYLSRGSLYKLLHRSGAR
591
D+H
+RF+EFMREVAIMKSLRHPNIVLFMGAVTEPPNLSIVTEYLSRGSLYKLLHRSGA+
Sbjct 532
DFHPERFREFMREVAIMKSLRHPNIVLFMGAVTEPPNLSIVTEYLSRGSLYKLLHRSGAK
591

Query 592
EVLDERRRLNMAFDVAKGMNYLHRRSPPIVHRDLKSPNLLVDKKYTVKVCDFGLSRLKA
N 651

EVLDERRRLNMAFDVAKGMNYLHRRSPPIVHRDLKSPNLLVDKKYTVKVCDFGLSRLKA
N
Sbjct 592
EVLDERRRLNMAFDVAKGMNYLHRRSPPIVHRDLKSPNLLVDKKYTVKVCDFGLSRLKA
N 651

Query 652
TYLSSKSLAGTPEWMAPEVLRDEPSNEKSDVYSFAVILWELMTLQQPWCNLNPAQVVAAV
711
T+LSSKSLAGTPEWMAPEVLRDEPSNEKSDVYSF
VILWELMTLQQPWCNLNPAQVVAAV
Sbjct 652
TFLSSKSLAGTPEWMAPEVLRDEPSNEKSDVYSFGVILWELMTLQQPWCNLNPAQVVAA
V 711

Query 712
GFKGRRLEIPKELNPQVAALIESCWANEPWRRPSFANIMETLRPLINKVPVPQLIRSDS
770
GFKGRRLEIPK+LNP VAALIESCWANEPWRRPSF NIME LRPLI KVPVPQL
RSDS
Sbjct 712
GFKGRRLEIPKDLNPLVAALIESCWANEPWRRPSFTNIMEALRPLI-KVPVPQLSRSDS 769

>uncharacterized protein LOC112880881 [Panicum hallii]
Sequence ID: XP_025801398.1 Length: 1965
Range 1: 1 to 769

Score:1179 bits(3049), Expect:0.0,
Method:Compositional matrix adjust.,
Identities:644/779(83%), Positives:690/779(88%), Gaps:19/779(2%)

Query 1
MELPAAGGGRRTSYSLLSQFPDDAAAAGASPA---VLQRQSSGGSSYGAGSSVSASSDYP
57
MELP G RRT+YSLLSQFPDDAAA G PA VLQRQSSG S
SSVSASSDYP
Sbjct 1
MELPP-GAARRTTYSLLSQFPDDAAAGGPGPAPANVLQRQSSGSSYGAG-SSVSASSDYP
58

Query 58
FHLPPAVAAAGGGGGTPSP------CKSWAQQAEETYQLQLALALRLCADAACAADPGFL
111
FHLPP +AA G G +
CKSWAQQAEETYQLQLALALRLCADAACAADPGFL
Sbjct 59
FHLPPPASAAAGPGSAAAAPGGSSPCKSWAQQAEETYQLQLALALRLCADAACAADPGF
L 118

Query 112
DPGDSGGSKMGGGGGGSGSGRAFPLAPPSPTAEALSHRFWVNGSLSYSNTIPDGFYLIQG
171
D G GG GSG+GRAFPLA
P+P+AE+LSHRFWVNGSLSY++TIPDGFY+I G
Sbjct 119
D-----PGDPGSGGRGSGNGRAFPLAQPAPSAESLSHRFWVNGSLSYNSTIPDGFYVIHG
173

Query 172
MDPFVWSMCTDVHEENRIPSVESLKSVRPDDSSIQVVLVDRRADFDLGMLENYASSFLSS
231
+DPFVWS+CTDVHEENRIP++ESLKSVRPDDSSIQ +L+DRR
DFDLGMLE+YASS LSS
Sbjct 174
LDPFVWSLCTDVHEENRIPTMESLKSVRPDDSSIQAILIDRRTDFDLGMLESYASSLLSS
233

Query 232
SSDMKDVINQLAKLVSSRMGGTTSNEENLLPRWKESSEAIKSSAGSIVLHLGKLPIGLCK
291

+D KDV+ QLAK+VSSRMGGT SNEENL PRWKE +EAIKSS GS+ LHLGKLP+GLCK
Sbjct 234 CTDAKDVVIQLAKVVSSRMGGTASNEENLFPRWKECNEAIKSSTGSVALHLGKLPVGLCK 293

Query 292 HRSLLFKMLADKVNIPCRLVKGCKYCKAEDASSCVVRFGLEREYLVDLFGAPGQLSDPDS 351
HRSLLFKMLADKV+IPCR+VKGCKYCK++DASSC+VRFGLEREYLVDL G PGQLSDPDS
Sbjct 294 HRSLLFKMLADKVSIPCRVVKGCKYCKSDDASSCLVRFGLEREYLVDLIGDPGQLSDPDS 353

Query 352 FVNGPYSLSVPSPLRPPKFRSLEITSNFSSVAKQYFSDCHSLNLLFSDASTGASNGAAVA 411
FVNGPYSLSV SPL PPKFRSLEITSNFS VAKQYF+DCHSLNLLFSD+STGA++ A
Sbjct 354 FVNGPYSLSVSSPLHPPKFRSLEITSNFSLVAKQYFADCHSLNLLFSDSSTGATS-AVTT 412

Query 412 VDQMYSKKHDAGDGIANSWVPVKGQAIANSDIILPEAPREVLPLMSPSNLTADKKKEFQL 471
+DQ YSKKH AGD N+W+P KGQA DII+PEAPREVLPL+S SN+ DKKKE +L
Sbjct 413 LDQPYSKKHVAGDETMNNWMPGKGQAAMKPDIIVPEAPREVLPLIS-SNMKLDKKKELKL 471

Query 472 IEGNQYLRSTVSDLSLAVDDLIIPWSELVLKEKIGAGSFGTVHRADWHGSDVAVKILMEQ 531
IE Q LR TVSDLSLA DDLIIPW+ELVLKEKIGAGSFGTVHRADWHGSDVAVKILMEQ
Sbjct 472 IEETQQLRHTVSDLSLAADDLIIPWNELVLKEKIGAGSFGTVHRADWHGSDVAVKILMEQ 531

Query 532 DYHLDRFKEFMREVAIMKSLRHPNIVLFMGAVTEPPNLSIVTEYLSRGSLYKLLHRSGAR 591

D+H
+RF+EFMREVAIMKSLRHPNIVLFMGAVTEPPNLSIVTEYLSRGSLYKLLHRSGA+
Sbjct 532
DFHPERFREFMREVAIMKSLRHPNIVLFMGAVTEPPNLSIVTEYLSRGSLYKLLHRSGAK
591

Query 592
EVLDERRRLNMAFDVAKGMNYLHRRSPPIVHRDLKSPNLLVDKKYTVKVCDFGLSRLKA
N 651

EVLDERRRLNMAFDVAKGMNYLHRRSPPIVHRDLKSPNLLVDKKYTVKVCDFGLSRLKA
N
Sbjct 592
EVLDERRRLNMAFDVAKGMNYLHRRSPPIVHRDLKSPNLLVDKKYTVKVCDFGLSRLKA
N 651

Query 652
TYLSSKSLAGTPEWMAPEVLRDEPSNEKSDVYSFAVILWELMTLQQPWCNLNPAQVVAAV
711
T+LSSKSLAGTPEWMAPEVLRDEPSNEKSDVYSF
VILWELMTLQQPWCNLNPAQVVAAV
Sbjct 652
TFLSSKSLAGTPEWMAPEVLRDEPSNEKSDVYSFGVILWELMTLQQPWCNLNPAQVVAA
V 711

Query 712
GFKGRRLEIPKELNPQVAALIESCWANEPWRRPSFANIMETLRPLINKVPVPQLIRSDS
770
GFKGRRLEIPK+LNP VAALIESCWANEPWRRPSF NIME LRPLI KVPVPQL
RSDS
Sbjct 712
GFKGRRLEIPKDLNPLVAALIESCWANEPWRRPSFTNIMEALRPLI-KVPVPQLSRSDS 769

>hypothetical protein PVAP13_2NG262500 [Panicum virgatum]
Sequence ID: KAG2633570.1 Length: 768
Range 1: 1 to 768

Score:1173 bits(3034), Expect:0.0,
Method:Compositional matrix adjust.,
Identities:644/778(83%), Positives:694/778(89%), Gaps:18/778(2%)

Query 1
MELPAAGGGRRTSYSLLSQFPDDAAAAGASPA-VLQRQSSGGSSYGAGSSVSASSDYPFH
59
MELP G RRT+YSLLSQFPDDAAAAG PA LQRQSSG S G
GSSVSASSDYPFH
Sbjct 1
MELPP-GAARRTNYSLLSQFPDDAAAAGPGPANALQRQSSGSSY-GGGSSVSASSDYPFH
58

Query 60
LPPAVAAAGGGGGTPSP------CKSWAQQAEETYQLQLALALRLCADAACAADPGFLDP
113
LPP +AA G G +
CKSWAQQAEETYQLQLALALRLCADAACAADPGFLD
Sbjct 59
LPPPASAAAGPGSAAAAPGGSSPCKSWAQQAEETYQLQLALALRLCADAACAADPGFLD-
117

Query 114
GDSGGSKMGGGGGGSGSGRAFPLAPPSPTAEALSHRFWVNGSLSYSNTIPDGFYLIQGMD
173
G GG GSG+GRAFPLA P+P+AE+LSHRFWVNGSLSY++TIPDGFY+I
G+D
Sbjct 118
----PGDPGSGGRGSGNGRAFPLAQPAPSAESLSHRFWVNGSLSYNSTIPDGFYVIHGLD
173

Query 174
PFVWSMCTDVHEENRIPSVESLKSVRPDDSSIQVVLVDRRADFDLGMLENYASSFLSSSS
233
PFVWS+CTDVHEENRIPS+ESLKSVRPDDSSIQ +L+DRR DFDLGMLE+YASS
LSS +
Sbjct 174
PFVWSLCTDVHEENRIPSMESLKSVRPDDSSIQAILIDRRTDFDLGMLESYASSLLSSCT
233

Query 234
DMKDVINQLAKLVSSRMGGTTSNEENLLPRWKESSEAIKSSAGSIVLHLGKLPIGLCKHR
293
D KDV+ QLAK+VSSRMGGT SNE+NL PRWKE +EAIKSS
GS+VLHLGKLP+GLCKHR
Sbjct 234
DAKDVVIQLAKVVSSRMGGTASNEDNLFPRWKECNEAIKSSTGSVVLHLGKLPVGLCKH
R 293

Query 294
SLLFKMLADKVNIPCRLVKGCKYCKAEDASSCVVRFGLE-REYLVDLFGAPGQLSDPDSF
352
SLLFK+LADKV+IPCR+VKGCKYCK++DASSC+VRFGLE REYLVDL G
PGQLSDPDSF
Sbjct 294
SLLFKILADKVSIPCRVVKGCKYCKSDDASSCLVRFGLENREYLVDLIGDPGQLSDPDSF
353

Query 353
VNGPYSLSVPSPLRPPKFRSLEITSNFSSVAKQYFSDCHSLNLLFSDASTGASNGAAVAV
412
VNGPYSLSV SPL PPKFRSLEITSNFSSVAKQYFSDCHSLNLLFSD+STGA++
+
Sbjct 354
VNGPYSLSVSSPLHPPKFRSLEITSNFSSVAKQYFSDCHSLNLLFSDSSTGATS-VITTL 412

Query 413
DQMYSKKHDAGDGIANSWVPVKGQAIANSDIILPEAPREVLPLMSPSNLTADKKKEFQLI
472
DQ YSKKH AGD N+W+P KGQA DI++PEAPREVLPL+S SN+ D+KKE
+LI
Sbjct 413
DQPYSKKHVAGDETMNNWMPGKGQAAMKPDIVVPEAPREVLPLIS-SNMKLDRKKELKL
I 471

Query 473
EGNQYLRSTVSDLSLAVDDLIIPWSELVLKEKIGAGSFGTVHRADWHGSDVAVKILMEQD
532
E Q LR TVSDLSLA
+DLIIPW+ELVLKEKIGAGSFGTVHRADWHGSDVAVKILMEQD
Sbjct 472
EETQQLRHTVSDLSLAANDLIIPWNELVLKEKIGAGSFGTVHRADWHGSDVAVKILMEQD
531

Query 533
YHLDRFKEFMREVAIMKSLRHPNIVLFMGAVTEPPNLSIVTEYLSRGSLYKLLHRSGARE
592
+H
+RF+EFMREVAIMKSLRHPNIVLFMGAVTEPPNLSIVTEYLSRGSLYKLLHRSGA+E
Sbjct 532
FHPERFREFMREVAIMKSLRHPNIVLFMGAVTEPPNLSIVTEYLSRGSLYKLLHRSGAKE
591

Query 593
VLDERRRLNMAFDVAKGMNYLHRRSPPIVHRDLKSPNLLVDKKYTVKVCDFGLSRLKAN
T 652

VLDERRRLNMAFDVAKGMNYLHRRSPP+VHRDLKSPNLLVDKKYTVKVCDFGLSRLKA
NT
Sbjct 592
VLDERRRLNMAFDVAKGMNYLHRRSPPVVHRDLKSPNLLVDKKYTVKVCDFGLSRLKA
NT 651

Query 653
YLSSKSLAGTPEWMAPEVLRDEPSNEKSDVYSFAVILWELMTLQQPWCNLNPAQVVAAV
G 712
+LSSKSLAGTPEWMAPEVLRDEPSNEKSDVYSF
VILWELMTLQQPWCNLNPAQVVAAVG
Sbjct 652
FLSSKSLAGTPEWMAPEVLRDEPSNEKSDVYSFGVILWELMTLQQPWCNLNPAQVVAAV
G 711

Query 713
FKGRRLEIPKELNPQVAALIESCWANEPWRRPSFANIMETLRPLINKVPVPQLIRSDS 770
FKGRRLEIPK+LNP VAALIESCWANEPWRRPSF NIMETLRPLI KVPVPQL
RSDS
Sbjct 712
FKGRRLEIPKDLNPLVAALIESCWANEPWRRPSFTNIMETLRPLI-KVPVPQLSRSDS 768

>hypothetical protein PAHAL_2G129500 [Panicum hallii]
Sequence ID: PAN10967.1 Length: 769
Range 1: 1 to 769

Score:1172 bits(3032), Expect:0.0,
Method:Compositional matrix adjust.,
Identities:647/779(83%), Positives:693/779(88%), Gaps:19/779(2%)

Query 1
MELPAAGGGRRTSYSLLSQFPDDAAAAGASPA---VLQRQSSGGSSYGAGSSVSASSDYP
57
MELP G RRT+YSLLSQFPDDAAA G PA VLQRQSSG S
GAGSSVSASSDYP
Sbjct 1

MELPP-GAARRTTYSLLSQFPDDAAAGGPGPAPANVLQRQSSGSSY-GAGSSVSASSDYP 58

Query 58 FHLPPAVAAAGGGGGTPSP------CKSWAQQAEETYQLQLALALRLCADAACAADPGFL 111

FHLPP +AA G G + CKSWAQQAEETYQLQLALALRLCADAACAADPGFL

Sbjct 59 FHLPPPASAAAGPGSAAAAPGGSSPCKSWAQQAEETYQLQLALALRLCADAACAADPGFL 118

Query 112 DPGDSGGSKMGGGGGGSGSGRAFPLAPPSPTAEALSHRFWVNGSLSYSNTIPDGFYLIQG 171

D G GG GSG+GRAFPLA P+P+AE+LSHRFWVNGSLSY++TIPDGFY+I G

Sbjct 119 D-----PGDPGSGGRGSGNGRAFPLAQPAPSAESLSHRFWVNGSLSYNSTIPDGFYVIHG 173

Query 172 MDPFVWSMCTDVHEENRIPSVESLKSVRPDDSSIQVVLVDRRADFDLGMLENYASSFLSS 231

+DPFVWS+CTDVHEENRIP++ESLKSVRPDDSSIQ +L+DRR DFDLGMLE+YASS LSS

Sbjct 174 LDPFVWSLCTDVHEENRIPTMESLKSVRPDDSSIQAILIDRRTDFDLGMLESYASSLLSS 233

Query 232 SSDMKDVINQLAKLVSSRMGGTTSNEENLLPRWKESSEAIKSSAGSIVLHLGKLPIGLCK 291

+D KDV+ QLAK+VSSRMGGT SNEENL PRWKE +EAIKSS GS+ LHLGKLP+GLCK

Sbjct 234 CTDAKDVVIQLAKVVSSRMGGTASNEENLFPRWKECNEAIKSSTGSVALHLGKLPVGLCK 293

Query 292 HRSLLFKMLADKVNIPCRLVKGCKYCKAEDASSCVVRFGLEREYLVDLFGAPGQLSDPDS 351

HRSLLFKMLADKV+IPCR+VKGCKYCK++DASSC+VRFGLEREYLVDL G PGQLSDPDS

Sbjct 294
HRSLLFKMLADKVSIPCRVVKGCKYCKSDDASSCLVRFGLEREYLVDLIGDPGQLSDPDS
353

Query 352
FVNGPYSLSVPSPLRPPKFRSLEITSNFSSVAKQYFSDCHSLNLLFSDASTGASNGAAVA
411
FVNGPYSLSV SPL PPKFRSLEITSNFS VAKQYF+DCHSLNLLFSD+STGA++
A
Sbjct 354
FVNGPYSLSVSSPLHPPKFRSLEITSNFSLVAKQYFADCHSLNLLFSDSSTGATS-AVTT 412

Query 412
VDQMYSKKHDAGDGIANSWVPVKGQAIANSDIILPEAPREVLPLMSPSNLTADKKKEFQL
471
+DQ YSKKH AGD N+W+P KGQA DII+PEAPREVLPL+S SN+
DKKKE +L
Sbjct 413
LDQPYSKKHVAGDETMNNWMPGKGQAAMKPDIIVPEAPREVLPLIS-SNMKLDKKKELK
L 471

Query 472
IEGNQYLRSTVSDLSLAVDDLIIPWSELVLKEKIGAGSFGTVHRADWHGSDVAVKILMEQ
531
IE Q LR TVSDLSLA
DDLIIPW+ELVLKEKIGAGSFGTVHRADWHGSDVAVKILMEQ
Sbjct 472
IEETQQLRHTVSDLSLAADDLIIPWNELVLKEKIGAGSFGTVHRADWHGSDVAVKILMEQ
531

Query 532
DYHLDRFKEFMREVAIMKSLRHPNIVLFMGAVTEPPNLSIVTEYLSRGSLYKLLHRSGAR
591
D+H
+RF+EFMREVAIMKSLRHPNIVLFMGAVTEPPNLSIVTEYLSRGSLYKLLHRSGA+
Sbjct 532
DFHPERFREFMREVAIMKSLRHPNIVLFMGAVTEPPNLSIVTEYLSRGSLYKLLHRSGAK
591

Query 592
EVLDERRRLNMAFDVAKGMNYLHRRSPPIVHRDLKSPNLLVDKKYTVKVCDFGLSRLKA
N 651

EVLDERRRLNMAFDVAKGMNYLHRRSPPIVHRDLKSPNLLVDKKYTVKVCDFGLSRLKA

N
Sbjct 592
EVLDERRRLNMAFDVAKGMNYLHRRSPPIVHRDLKSPNLLVDKKYTVKVCDFGLSRLKA
N 651

Query 652
TYLSSKSLAGTPEWMAPEVLRDEPSNEKSDVYSFAVILWELMTLQQPWCNLNPAQVVAAV
711
T+LSSKSLAGTPEWMAPEVLRDEPSNEKSDVYSF
VILWELMTLQQPWCNLNPAQVVAAV
Sbjct 652
TFLSSKSLAGTPEWMAPEVLRDEPSNEKSDVYSFGVILWELMTLQQPWCNLNPAQVVAA
V 711

Query 712
GFKGRRLEIPKELNPQVAALIESCWANEPWRRPSFANIMETLRPLINKVPVPQLIRSDS
770
GFKGRRLEIPK+LNP VAALIESCWANEPWRRPSF NIME LRPLI KVPVPQL
RSDS
Sbjct 712
GFKGRRLEIPKDLNPLVAALIESCWANEPWRRPSFTNIMEALRPLI-KVPVPQLSRSDS 769

>uncharacterized protein LOC120695190 [Panicum virgatum]
Sequence ID: XP_039834424.1 Length: 1912
Range 1: 1 to 770

Score:1166 bits(3017), Expect:0.0,
Method:Compositional matrix adjust.,
Identities:637/780(82%), Positives:688/780(88%), Gaps:21/780(2%)

Query 1
MELPAAGGGRRTSYSLLSQFPDDAAAAGASP-----AVLQRQSSGGSSYGAGSSVSASSD
55
MELP G RRT+YSLLSQFPDDAA AG P + LQRQSSG S G
SSVSASSD
Sbjct 1
MELPP-GAARRTNYSLLSQFPDDAAGAGPGPGPAPASALQRQSSGSSYGGG-SSVSASSD
58

Query 56
YPFHLPPAVAAAGGGGGTPSP------CKSWAQQAEETYQLQLALALRLCADAACAADPG

109

YPFHLPP +AA G G + CKSWAQQAEETYQLQLALALRLCADAACAADPG

Sbjct 59

YPFHLPPPASAAAGPGSAAAAPGGSSPCKSWAQQAEETYQLQLALALRLCADAACAADPG 118

Query 110

FLDPGDSGGSKMGGGGGGSGSGRAFPLAPPSPTAEALSHRFWVNGSLSYSNTIPDGFYLI 169

FLD G GG GSG+GRAFPLA P+P+AE+LSHRFWVNGSLSY++TIPDGFY+I

Sbjct 119

FLD-----PGDPGSGGRGSGNGRAFPLAQPAPSAESLSHRFWVNGSLSYNSTIPDGFYVI 173

Query 170

QGMDPFVWSMCTDVHEENRIPSVESLKSVRPDDSSIQVVLVDRRADFDLGMLENYASSFL 229

G+DPFVWS+CTDVHEEN IPS+ESLKSVRPDDSSIQ +L+DRR DFDLGMLE+YASS L

Sbjct 174

HGLDPFVWSLCTDVHEENHIPSMESLKSVRPDDSSIQAILIDRRTDFDLGMLESYASSLL 233

Query 230

SSSSDMKDVINQLAKLVSSRMGGTTSNEENLLPRWKESSEAIKSSAGSIVLHLGKLPIGL 289

SS +D KDV+ QLAK+VSSRMGGT SNE++L +WKE +EAIKSS GS+VLHLGKLP+GL

Sbjct 234

SSCTDAKDVVIQLAKVVSSRMGGTASNEDSLFQKWKECNEAIKSSTGSVVLHLGKLPVGL 293

Query 290

CKHRSLLFKMLADKVNIPCRLVKGCKYCKAEDASSCVVRFGLEREYLVDLFGAPGQLSDP 349

CKHRSLLFK+LADKV+IPCR+VKGCKYCK++DASSC+VRFGLEREYLVDL G PGQLSDP

Sbjct 294

CKHRSLLFKILADKVSIPCRVVKGCKYCKSDDASSCLVRFGLEREYLVDLIGDPGQLSDP 353

Query 350

DSFVNGPYSLSVPSPLRPPKFRSLEITSNFSSVAKQYFSDCHSLNLLFSDASTGASNGAA

409

DSFVNGPYSLSV SPL PPKFRSLEITSNFSSVAKQYFSDCHSLNLLFSD+STGA++ A

Sbjct 354 DSFVNGPYSLSVSSPLHPPKFRSLEITSNFSSVAKQYFSDCHSLNLLFSDSSTGATS-AV 412

Query 410 VAVDQMYSKKHDAGDGIANSWVPVKGQAIANSDIILPEAPREVLPLMSPSNLTADKKKEF 469

+DQ YSKKH AGD N+W+P KGQA DII+ EAPREVLPL+S SN+ D+KKE

Sbjct 413 TTLDQPYSKKHVAGDETMNNWMPGKGQAAIKPDIIVQEAPREVLPLIS-SNMKLDRKKEL 471

Query 470 QLIEGNQYLRSTVSDLSLAVDDLIIPWSELVLKEKIGAGSFGTVHRADWHGSDVAVKILM 529

+LIE Q LR TVSDLSLA DDLIIPW+ELVLKEKIGAGSFGTVHRADWHGSDVAVKILM

Sbjct 472 KLIEETQQLRHTVSDLSLAADDLIIPWNELVLKEKIGAGSFGTVHRADWHGSDVAVKILM 531

Query 530 EQDYHLDRFKEFMREVAIMKSLRHPNIVLFMGAVTEPPNLSIVTEYLSRGSLYKLLHRSG 589

EQD+H +RF+EFMREVAIMKSLRHPNIVLFMGAVTEPPNLSIVTEYLSRGSLYKLLHRSG

Sbjct 532 EQDFHPERFREFMREVAIMKSLRHPNIVLFMGAVTEPPNLSIVTEYLSRGSLYKLLHRSG 591

Query 590 AREVLDERRRLNMAFDVAKGMNYLHRRSPPIVHRDLKSPNLLVDKKYTVKVCDFGLSRLK 649

A+EVLDERRRLNMAFDVAKGMNYLHRRSPPIVHRDLKSPNLLVDKKYTVKVCDFGLSRLK

Sbjct 592 AKEVLDERRRLNMAFDVAKGMNYLHRRSPPIVHRDLKSPNLLVDKKYTVKVCDFGLSRLK 651

Query 650

ANTYLSSKSLAGTPEWMAPEVLRDEPSNEKSDVYSFAVILWELMTLQQPWCNLNPAQVV A 709
NT+LSSKSLAGTPEWMAPEVLRDEPSNEKSDVYSF VILWELMTLQQPWCNLNPAQVVA
Sbjct 652 PNTFLSSKSLAGTPEWMAPEVLRDEPSNEKSDVYSFGVILWELMTLQQPWCNLNPAQVVA 711

Query 710 AVGFKGRRLEIPKELNPQVAALIESCWANEPWRRPSFANIMETLRPLINKVPVPQLIRSD 769
AVGFKGRRLEIPK+LNP VAALIESCWA+EPWRRPSF NIMETLRPLI KVPVPQL RSD
Sbjct 712 AVGFKGRRLEIPKDLNPLVAALIESCWASEPWRRPSFTNIMETLRPLI-KVPVPQLSRSD 770

>hypothetical protein PVAP13_2KG218100 [Panicum virgatum]
Sequence ID: KAG2641912.1 Length: 771
>hypothetical protein PVAP13_2KG218100 [Panicum virgatum]
Sequence ID: KAG2641914.1 Length: 771
Range 1: 1 to 770

Score:1164 bits(3012), Expect:0.0,
Method:Compositional matrix adjust.,
Identities:638/780(82%), Positives:689/780(88%), Gaps:21/780(2%)

Query 1 MELPAAGGGRRTSYSLLSQFPDDAAAAGASP-----AVLQRQSSGGSSYGAGSSVSASSD 55
MELP G RRT+YSLLSQFPDDAA AG P + LQRQSSG S G GSSVSASSD
Sbjct 1 MELPP-GAARRTNYSLLSQFPDDAAGAGPGPGPAPASALQRQSSGSSY-GGGSSVSASSD 58

Query 56 YPFHLPPAVAAAGGGGGTPSP------CKSWAQQAEETYQLQLALALRLCADAACAADPG 109
YPFHLPP +AA G G + CKSWAQQAEETYQLQLALALRLCADAACAADPG

Sbjct 59
YPFHLPPPASAAAGPGSAAAAPGGSSPCKSWAQQAEETYQLQLALALRLCADAACAADP
G 118

Query 110
FLDPGDSGGSKMGGGGGGSGSGRAFPLAPPSPTAEALSHRFWVNGSLSYSNTIPDGFYLI
169
FLD G GG GSG+GRAFPLA
P+P+AE+LSHRFWVNGSLSY++TIPDGFY+I
Sbjct 119
FLD-----PGDPGSGGRGSGNGRAFPLAQPAPSAESLSHRFWVNGSLSYNSTIPDGFYVI 173

Query 170
QGMDPFVWSMCTDVHEENRIPSVESLKSVRPDDSSIQVVLVDRRADFDLGMLENYASSFL
229
G+DPFVWS+CTDVHEEN IPS+ESLKSVRPDDSSIQ +L+DRR
DFDLGMLE+YASS L
Sbjct 174
HGLDPFVWSLCTDVHEENHIPSMESLKSVRPDDSSIQAILIDRRTDFDLGMLESYASSLL
233

Query 230
SSSSDMKDVINQLAKLVSSRMGGTTSNEENLLPRWKESSEAIKSSAGSIVLHLGKLPIGL
289
SS +D KDV+ QLAK+VSSRMGGT SNE++L +WKE +EAIKSS
GS+VLHLGKLP+GL
Sbjct 234
SSCTDAKDVVIQLAKVVSSRMGGTASNEDSLFQKWKECNEAIKSSTGSVVLHLGKLPVGL
293

Query 290
CKHRSLLFKMLADKVNIPCRLVKGCKYCKAEDASSCVVRFGLEREYLVDLFGAPGQLSDP
349
CKHRSLLFK+LADKV+IPCR+VKGCKYCK++DASSC+VRFGLEREYLVDL G
PGQLSDP
Sbjct 294
CKHRSLLFKILADKVSIPCRVVKGCKYCKSDDASSCLVRFGLEREYLVDLIGDPGQLSDP
353

Query 350
DSFVNGPYSLSVPSPLRPPKFRSLEITSNFSSVAKQYFSDCHSLNLLFSDASTGASNGAA
409
DSFVNGPYSLSV SPL
PPKFRSLEITSNFSSVAKQYFSDCHSLNLLFSD+STGA++ A

Sbjct 354
DSFVNGPYSLSVSSPLHPPKFRSLEITSNFSSVAKQYFSDCHSLNLLFSDSSTGATS-AV 412

Query 410
VAVDQMYSKKHDAGDGIANSWVPVKGQAIANSDIILPEAPREVLPLMSPSNLTADKKKEF
469
+DQ YSKKH AGD N+W+P KGQA DII+ EAPREVLPL+S SN+
D+KKE
Sbjct 413
TTLDQPYSKKHVAGDETMNNWMPGKGQAAIKPDIIVQEAPREVLPLIS-SNMKLDRKKEL
471

Query 470
QLIEGNQYLRSTVSDLSLAVDDLIIPWSELVLKEKIGAGSFGTVHRADWHGSDVAVKILM
529
+LIE Q LR TVSDLSLA
DDLIIPW+ELVLKEKIGAGSFGTVHRADWHGSDVAVKILM
Sbjct 472
KLIEETQQLRHTVSDLSLAADDLIIPWNELVLKEKIGAGSFGTVHRADWHGSDVAVKILM
531

Query 530
EQDYHLDRFKEFMREVAIMKSLRHPNIVLFMGAVTEPPNLSIVTEYLSRGSLYKLLHRSG
589
EQD+H
+RF+EFMREVAIMKSLRHPNIVLFMGAVTEPPNLSIVTEYLSRGSLYKLLHRSG
Sbjct 532
EQDFHPERFREFMREVAIMKSLRHPNIVLFMGAVTEPPNLSIVTEYLSRGSLYKLLHRSG
591

Query 590
AREVLDERRRLNMAFDVAKGMNYLHRRSPPIVHRDLKSPNLLVDKKYTVKVCDFGLSRL
K 649

A+EVLDERRRLNMAFDVAKGMNYLHRRSPPIVHRDLKSPNLLVDKKYTVKVCDFGLSRL
K
Sbjct 592
AKEVLDERRRLNMAFDVAKGMNYLHRRSPPIVHRDLKSPNLLVDKKYTVKVCDFGLSRL
K 651

Query 650
ANTYLSSKSLAGTPEWMAPEVLRDEPSNEKSDVYSFAVILWELMTLQQPWCNLNPAQVV
A 709
NT+LSSKSLAGTPEWMAPEVLRDEPSNEKSDVYSF

VILWELMTLQQPWCNLNPAQVVA

Sbjct 652
PNTFLSSKSLAGTPEWMAPEVLRDEPSNEKSDVYSFGVILWELMTLQQPWCNLNPAQVVA
711

Query 710
AVGFKGRRLEIPKELNPQVAALIESCWANEPWRRPSFANIMETLRPLINKVPVPQLIRSD
769
AVGFKGRRLEIPK+LNP VAALIESCWA+EPWRRPSF NIMETLRPLI KVPVPQL RSD
Sbjct 712
AVGFKGRRLEIPKDLNPLVAALIESCWASEPWRRPSFTNIMETLRPLI-KVPVPQLSRSD
770

>serine/threonine-protein kinase CTR1 isoform X2 [Phragmites australis]
Sequence ID: XP_062194397.1 Length: 738
Range 1: 1 to 738

Score:1161 bits(3003), Expect:0.0,
Method:Compositional matrix adjust.,
Identities:625/775(81%), Positives:673/775(86%), Gaps:42/775(5%)

Query 1
MELPAAGGGRRTSYSLLSQFPDDAAAAGASPAVLQRQSSGGSSYGAGSSVSASSDYPFHL
60
M+LP G RRT+YSLLSQFP+DA VLQRQSSG SSYGAGSS+SASSDYPFHL
Sbjct 1
MDLPP-GAARRTTYSLLSQFPEDAPGPAN---VLQRQSSGSSSYGAGSSISASSDYPFHL 56

Query 61
PPAVAAAGGGGGTPS-----PCKSWAQQAEETYQLQLALALRLCADAACAADPGFLDPGD
115
P VA + + PCK+WAQQAEETYQLQLALALRLCADAACAADPGFLDPGD
Sbjct 57
QPPVAGSATTTAAAAASGGSPCKTWAQQAEETYQLQLALALRLCADAACAADPGFLDPG
D 116

Query 116
SGGSKMGGGGGGSGSGRAFPLAPPSPTAEALSHRFWVNGSLSYSNTIPDGFYLIQGMDPF

175

SG + SG+GRAFPLA P+P+AE+LSHRFWVNGSLSY++TIPDGFYLI GMDPF

Sbjct 117
SGSGRG------SGNGRAFPLAQPAPSAESLSHRFWVNGSLSYNSTIPDGFYLIHGMDPF 170

Query 176
VWSMCTDVHEENRIPSVESLKSVRPDDSSIQVVLVDRRADFDLGMLENYASSFLSSSSDM
235

VWS+CTDVH+ENRIPS+ESLKSVRPDDSSIQ +L+DRR DF+LGMLE+YASS LSS +D

Sbjct 171
VWSLCTDVHDENRIPSMESLKSVRPDDSSIQAILIDRRTDFELGMLESYASSLLSSFADA
230

Query 236
KDVINQLAKLVSSRMGGTTSNEENLLPRWKESSEAIKSSAGSIVLHLGKLPIGLCKHRSL
295

KDV+ QLAKL+SSR GGTTS+EENLLP+WKE EAIKSS GS+VLHLGKLPIGLCKHRSL

Sbjct 231
KDVVKQLAKLISSRTGGTTSDEENLLPQWKECCEAIKSSTGSVVLHLGKLPIGLCKHRSL
290

Query 296
LFKMLADKVNIPCRLVKGCKYCKAEDASSCVVRFGLEREYLVDLFGAPGQLSDPDSFVN
G 355

LFKMLADKVN+ CR+VKGCKYCK++DASSC+VRFGLEREYLVDL G PGQL+DPDSFVNG

Sbjct 291
LFKMLADKVNVRCRVVKGCKYCKSDDASSCLVRFGLEREYLVDLIGDPGQLTDPDSFVN
G 350

Query 356
PYSLSVPSPLRPPKFRSLEITSNFSSVAKQYFSDCHSLNLLFSDASTGASNGAAVAVDQM
415

PYSLSV SPL PPKFRSLEITSNFSSVAKQYFSDCHSLNLLFSD+STGA+N AAVA+DQ

Sbjct 351
PYSLSVSSPLHPPKFRSLEITSNFSSVAKQYFSDCHSLNLLFSDSSTGAANSAAVALDQP
410

Query 416
YSKKHDAGDGIANSWVPVKGQAIANSDIILPEAPREVLPLMSPSNLTADKKKEFQLIEGN

475
YSKKH AGD I N WVP KGQA II+PE PR+
Sbjct 411 YSKKHVAGDDIINRWVPGKGQAAMKPAIIVPEDPRK----------------------- 446

Query 476
QYLRSTVSDLSLAVDDLIIPWSELVLKEKIGAGSFGTVHRADWHGSDVAVKILMEQDYHL
535
L+ TVS LSLA
DDLIIPW+ELVLKEKIGAGSFGTVHRADWHGSDVAVKILMEQD+H
Sbjct 447
--LQDTVSGLSLAADDLIIPWNELVLKEKIGAGSFGTVHRADWHGSDVAVKILMEQDFHP
504

Query 536
DRFKEFMREVAIMKSLRHPNIVLFMGAVTEPPNLSIVTEYLSRGSLYKLLHRSGAREVLD
595

+RF+EFMREVAIMKSLRHPNIVLFMGAVTEPPNLSIVTEYLSRGSLYKLLHRSGA+EVLD
Sbjct 505
ERFREFMREVAIMKSLRHPNIVLFMGAVTEPPNLSIVTEYLSRGSLYKLLHRSGAKEVLD
564

Query 596
ERRRLNMAFDVAKGMNYLHRRSPPIVHRDLKSPNLLVDKKYTVKVCDFGLSRLKANTYL
S 655

ERRRLNMAFDVAKGMNYLHRR+PPIVHRDLKSPNLLVDKKYTVKVCDFGLSRLKANT+L
S
Sbjct 565
ERRRLNMAFDVAKGMNYLHRRNPPIVHRDLKSPNLLVDKKYTVKVCDFGLSRLKANTFL
S 624

Query 656
SKSLAGTPEWMAPEVLRDEPSNEKSDVYSFAVILWELMTLQQPWCNLNPAQVVAAVGFK
G 715
SKSLAGTPEWMAPEVLRDE SNEKSDVYS+
VILWELMTLQQPWCNLNPAQVVAAVGFKG
Sbjct 625
SKSLAGTPEWMAPEVLRDELSNEKSDVYSYGVILWELMTLQQPWCNLNPAQVVAAVGFK
G 684

Query 716
RRLEIPKELNPQVAALIESCWANEPWRRPSFANIMETLRPLINKVPVPQLIRSDS 770
RRLEIPK+LNP VAALIESCWANEPWRRPSFANIM TLRPLI KVPVPQLIRSDS

Sbjct 685 RRLEIPKDLNPLVAALIESCWANEPWRRPSFANIMVTLRPLI-KVPVPQLIRSDS 738

>hypothetical protein GUJ93_ZPchr0009g1750 [Zizania palustris]
Sequence ID: KAG8050274.1 Length: 811
Range 1: 1 to 811

Score:1158 bits(2995), Expect:0.0,
Method:Compositional matrix adjust.,
Identities:629/832(76%), Positives:682/832(81%), Gaps:83/832(9%)

Query 1 MELPAAGGGRRTSYSLLSQFPDDAAAAGASPAVLQRQSSGGSSYGAGSSVSASSDYPFHL 60
M+LP GGRRT+YSLLSQ P+DAA V+QR SSG S GAGSS+SASS+YPFHL
Sbjct 1 MDLPV--GGRRTTYSLLSQLPEDAA-------VVQRHSSGSSY-GAGSSLSASSEYPFHL 50

Query 61 PPAVAAAGGGGGTPS---PCKSWAQQAEETYQLQLALALRLCADAACAADPGFLDPGDSG 117
P A + G + PCKSWAQQAEETYQLQLALALRLCADAAC+ADPGFLD
Sbjct 51 PATAAGSTAAAGAGAGGSPCKSWAQQAEETYQLQLALALRLCADAACSADPGFLD----- 105

Query 118 GSKMGGGGGGSGSGRAFPLAPPSPTAEALSHRFWVNGSLSYSNTIPDGFYLIQGMDPFVW 177
GG GSGSGRAFPL P P+AE+LSHRFWVNGSLSYSNT+PDGFYLI GMDPFVW
Sbjct 106 -----TGGSGSGSGRAFPLPPLIPSAESLSHRFWVNGSLSYSNTVPDGFYLIHGMDPFVW 160

Query 178 SMCTDVHEENRIPSVESLKSVRPDDSSIQVVLVDRRADFDLGMLENYASSFLSSSSDMKD 237
S+CTDV EENRIPS+ESLKSVRPDD SIQ +L+DRR DFDLGMLENYASSFLSSSSDM+D

Sbjct 161
SLCTDVQEENRIPSLESLKSVRPDDCSIQAILIDRRTDFDLGMLENYASSFLSSSSDMRD
220

Query 238
VINQLAKLVSSRMGGTTSNEENLLPRWKESSEAIKSSAGSIVLHLGKLPIGLCKHRSLLF
297
VINQLAKLVSSRMGGT SNE+ LP WKE +AIKSS GSIVLHLGKLPIG
CKHRSLLF
Sbjct 221
VINQLAKLVSSRMGGTASNEDTFLPYWKECGDAIKSSTGSIVLHLGKLPIGFCKHRSLLF
280

Query 298
KMLADKVNIPCRLVKGCKYCKAEDASSCVVRFGLEREYLVDLFGAPGQLSDPDSFVNGP
Y 357
K+LADKVN+PCR+VKGCKYCK++DA+SC+VRFGLEREYLVDL G PG
LSDPDSF+NGPY
Sbjct 281
KILADKVNVPCRVVKGCKYCKSDDATSCLVRFGLEREYLVDLIGDPGHLSDPDSFLNGPY
340

Query 358
SLSVPSPLRPPKFRSLEITSNFSSVAKQYFSDCHSLNLLFSDASTGASNGAAVAVDQMYS
417
SLSVPSPLRPPKFRSLEITSNFSSVAKQYFSDCHSLNLLF++ STGA +
AVAVDQ+Y
Sbjct 341
SLSVPSPLRPPKFRSLEITSNFSSVAKQYFSDCHSLNLLFNETSTGAVSSTAVAVDQLYY
400

Query 418
KKHDAGDGIANSWVPVKGQAIANSDIILPEAPREVLPLMSPSNLTADKKKEFQLIEGNQY
477
KKHD+ D I +SWVPVKGQA NSD ILPEAPREVLPL++ +NL A K
EF+LIEGNQ
Sbjct 401
KKHDSRDDIMSSWVPVKGQAAVNSDAILPEAPREVLPLITSANLEAGKNNEFKLIEGNQQ
460

Query 478
LRSTVSDLSLAVDDLIIPWSELVLKEKIGAGSFGTVHRADWHGSDVAVKILMEQDYHLDR
537
LRSTVS S

AVDDLIIPW+ELVLKEKIGAGSFGTVHRADW+GSDVAVKILMEQD+H D
Sbjct 461
LRSTVSSFSFAVDDLIIPWNELVLKEKIGAGSFGTVHRADWNGSDVAVKILMEQDFHPDH
520

Query 538
FKEFMREVAIMKSLRHPNIVLFMGAVTEPPNLSIVTEYLSRGSLYKLLHRSGAREVLDER
597

F+EFMREVAIMKSLRHPNIVLFMGAVTEPPNLSI+TEYLSRGSLYKLLHRSGA+EVLDER
Sbjct 521
FREFMREVAIMKSLRHPNIVLFMGAVTEPPNLSIITEYLSRGSLYKLLHRSGAKEVLDER
580

Query 598
RRLNMAFDVAKGMNYLHRRSPPIVHRDLKSPNLLVDKKYTVKVCDFGLSRLKANTYLSS
K 657

RRLNMAFDVAKGMNYLH+R+PPIVHRDLKSPNLLVDKKYTVK+CDFGLSRLKANT+LSSK
Sbjct 581
RRLNMAFDVAKGMNYLHKRNPPIVHRDLKSPNLLVDKKYTVKICDFGLSRLKANTFLSS
K 640

Query 658
SLAGTPEWMAPEVLRDEPSNEKSDVYSFAVILWELMTLQQP---------------WCNL 702
SLAGTPEWMAPEVLRDEPSNEKSDVYSF VILWE+MT+QQP
WC L
Sbjct 641
SLAGTPEWMAPEVLRDEPSNEKSDVYSFGVILWEIMTMQQPWFNMNPAQVISLYLIWCVL
700

Query 703 NPA--QVVAAVGFKGRRL 718
QVVAAVGFKGRRL
Sbjct 701
ADEVVRLFLDIIQCMYDIFCFSRSKYTPEVVQIIILFCHFIWWYAFPQVVAAVGFKGRRL
760

Query 719 EIPKELNPQVAALIESCWANEPWRRPSFANIMETLRPLINKVPVPQLIRSDS
770
+IPK+LNPQV ALIE CWANEPWRRPSFANIME+LR I KVP+PQLIRSDS
Sbjct 761 DIPKDLNPQVTALIELCWANEPWRRPSFANIMESLRS-ITKVPLPQLIRSDS
811

>serine/threonine-protein kinase CTR1 [Zea mays]
Sequence ID: XP_033915561.1 Length: 769
>serine/threonine-protein kinase CTR1 [Zea mays]
Sequence ID: XP_033915562.1 Length: 769
>serine/threonine-protein kinase CTR1 [Zea mays]
Sequence ID: XP_033915563.1 Length: 769
>serine/threonine-protein kinase CTR1 [Zea mays]
Sequence ID: XP_033915564.1 Length: 769
>Serine/threonine-protein kinase CTR1 [Zea mays]
Sequence ID: ONM20883.1 Length: 769
>Serine/threonine-protein kinase CTR1 [Zea mays]
Sequence ID: PWZ37428.1 Length: 769
>hypothetical protein Zm00014a_012110 [Zea mays]
Sequence ID: PWZ37429.1 Length: 769
>hypothetical protein Zm00014a_012110 [Zea mays]
Sequence ID: PWZ37430.1 Length: 769
>hypothetical protein Zm00014a_012110 [Zea mays]
Sequence ID: PWZ37431.1 Length: 769
Range 1: 1 to 769

Score:1156 bits(2991), Expect:0.0,
Method:Compositional matrix adjust.,
Identities:632/782(81%), Positives:684/782(87%), Gaps:25/782(3%)

Query 1
MELPAAGGGRRTSYSLLSQFPDDAAAAGASPA-VLQRQSSGGSSYGAGSSVSASSDYPFH
59
M+LP G GRRT+YSLL QFPDDAAA +PA +LQRQSSG S S +
SSDYPFH
Sbjct 1
MDLPP-GAGRRTTYSLLPQFPDDAAAPAPAPANILQRQSSGSSYGAGSSIST-SSDYPFH 58

Query 60
LPPAVAAAG---------GGGGTPSPCKSWAQQAEETYQLQLALALRLCADAACAADPGF 110
L P +AAG G
SPCKSWAQQAEETYQLQLALALRLCADAACAADPGF
Sbjct 59
LQPPASAAGVAPPGSAAAPPGAAGSPCKSWAQQAEETYQLQLALALRLCADAACAADPG
F 118

Query 111
LDPGDSGGSKMGGGGGGSGSGRAFPLAPPSPTAEALSHRFWVNGSLSYSNTIPDGFYLIQ

170

LDPGDSG + SG+GRAFPLA P+P+AE+LSHRFWVNGSLSY++TIPDGFYLI

Sbjct 119 LDPGDSGSGRG------SGNGRAFPLAQPAPSAESLSHRFWVNGSLSYNSTIPDGFYLIH 172

Query 171 GMDPFVWSMCTDVHEENRIPSVESLKSVRPDDSSIQVVLVDRRADFDLGMLENYASSFLS 230

GMDPFVWS+CTDV EEN IPS+ESLKSVRPDD SIQ +L+DRR DF+LGMLE+YASS LS

Sbjct 173 GMDPFVWSLCTDVQEENHIPSMESLKSVRPDDPSIQAILIDRRTDFELGMLESYASSILS 232

Query 231 SSSDMKDVINQLAKLVSSRMGGTTSNEENLLPRWKESSEAIKSSAGSIVLHLGKLPIGLC 290

SS+D KDV+ QLAKL+SSRMGGTTSNEENLL RWKE EAIKSS GS+VLHLGKLPIGLC

Sbjct 233 SSADAKDVVIQLAKLISSRMGGTTSNEENLLQRWKECIEAIKSSTGSVVLHLGKLPIGLC 292

Query 291 KHRSLLFKMLADKVNIPCRLVKGCKYCKAEDASSCVVRFGLEREYLVDLFGAPGQLSDPD 350

KHRSLLFK+LADKVNIPCR+VKGCKYCK++DASSC+VRFGLERE+LVDL PGQL+DPD

Sbjct 293 KHRSLLFKVLADKVNIPCRVVKGCKYCKSDDASSCLVRFGLEREFLVDLIRDPGQLTDPD 352

Query 351 SFVNGPYSLSVPSPLRPPKFRSLEITSNFSSVAKQYFSDCHSLNLLFSDASTGASNGAAV 410

SFVNGPYSLSV SPL PPKFRSLEITSNF SVAKQYFSDCHSLNLLFSD+STG +N +

Sbjct 353 SFVNGPYSLSVSSPLHPPKFRSLEITSNFGSVAKQYFSDCHSLNLLFSDSSTGVANSTVI 412

Query 411 AVDQMYSKKHDAGDGIANSWVPVKGQAIANSDIILPEAPREVLPLMSPSN--LTADKKKE

468

++D YSKKH AGD + NSWVP KGQ I DI++PEAPR VLPL++ SN L DKKKE

Sbjct 413 SLDHPYSKKHVAGDDVINSWVPGKGQGIMKPDIMVPEAPRVVLPLVTSSNIKLELDKKKE 472

Query 469 FQLIEGNQYLRSTVSDLSLAVDDLIIPWSELVLKEKIGAGSFGTVHRADWHGSDVAVKIL 528

+ LR+TVSDLSLA DDLIIPW+EL+LKEKIGAGSFGTVHRADWHGSDVAVKIL

Sbjct 473 LVTTQ----LRNTVSDLSLAADDLIIPWNELILKEKIGAGSFGTVHRADWHGSDVAVKIL 528

Query 529 MEQDYHLDRFKEFMREVAIMKSLRHPNIVLFMGAVTEPPNLSIVTEYLSRGSLYKLLHRS 588

MEQD+H +RF+EFMREVAIMKSLRHPNIVLFMGAVTEPPNLSIVTEYLSRGSLYKLLHRS

Sbjct 529 MEQDFHPERFREFMREVAIMKSLRHPNIVLFMGAVTEPPNLSIVTEYLSRGSLYKLLHRS 588

Query 589 GAREVLDERRRLNMAFDVAKGMNYLHRRSPPIVHRDLKSPNLLVDKKYTVKVCDFGLSRL 648

GA+EVLDERRRLNMAFDVAKGMNYLHRRSPPIVHRDLKSPNLLVDKKYTVKVCDFGLSRL

Sbjct 589 GAKEVLDERRRLNMAFDVAKGMNYLHRRSPPIVHRDLKSPNLLVDKKYTVKVCDFGLSRL 648

Query 649 KANTYLSSKSLAGTPEWMAPEVLRDEPSNEKSDVYSFAVILWELMTLQQPWCNLNPAQVV 708

KANT+LSSKSLAGTPEWMAPEVLRDEPSNEKSDVYSF VILWELMTLQQPWCNLNPAQVV

Sbjct 649 KANTFLSSKSLAGTPEWMAPEVLRDEPSNEKSDVYSFGVILWELMTLQQPWCNLNPAQVV 708

Query 709
AAVGFKGRRLEIPKELNPQVAALIESCWANEPWRRPSFANIMETLRPLINKVPVPQLIRS
768
AAVGFKGRRLEIPK+LNP VA LIESCWANEPWRRPSFANIM+TL+PLINKVP
QLIRS
Sbjct 709
AAVGFKGRRLEIPKDLNPLVAVLIESCWANEPWRRPSFANIMDTLKPLINKVPA-QLIRS
767

Query 769 DS 770
DS
Sbjct 768 DS 769

>hypothetical protein BS78_02G130700 [Paspalum vaginatum]
Sequence ID: KAJ1288982.1 Length: 737
Range 1: 6 to 737

Score:1149 bits(2973), Expect:0.0,
Method:Compositional matrix adjust.,
Identities:621/773(80%), Positives:667/773(86%), Gaps:50/773(6%)

Query 7
GGGRRTSYSLLSQFPDDAAAAGASPAVLQRQSSGGSSYGAGSSVSASSDYPFHLPPAVAA
66
G RRT+YSLLSQFPDDAAA G + VLQRQSSG S GAGSS+SASSDYPFH P
+A
Sbjct 6
GAARRTTYSLLSQFPDDAAAPGPA-NVLQRQSSGSSY-GAGSSISASSDYPFHFQPPASA 63

Query 67
AG---------GGGGTPSPCKSWAQQAEETYQLQLALALRLCADAACAADPGFLDPGDSG
117
AG G
SPCKSWAQQAEETYQLQLALALRLC+DAACAADPGFLDPGDSG
Sbjct 64
AGVAPPGSAAAPPGAAGSPCKSWAQQAEETYQLQLALALRLCSDAACAADPGFLDPGDS
G 123

Query 118
GSKMGGGGGGSGSGRAFPLAPPSPTAEALSHRFWVNGSLSYSNTIPDGFYLIQGMDPFVW
177

+ SG+GRAFPLA P+P+AE+LSHRFWVNGSLSY++TIPDGFYLI GMDPFVW
Sbjct 124 SGRG------SGNGRAFPLAHPAPSAESLSHRFWVNGSLSYNSTIPDGFYLIHGMDPFVW 177

Query 178 SMCTDVHEENRIPSVESLKSVRPDDSSIQVVLVDRRADFDLGMLENYASSFLSSSDMKD 237
S+CTD+HEENRIPS+ESLKSV PDDSSIQ +L+DRRADF+LGMLE+YASS LSSS+D KD
Sbjct 178 SLCTDIHEENRIPSMESLKSVCPDDSSIQAILIDRRADFELGMLESYASSLLSSSADAKD 237

Query 238 VINQLAKLVSSRMGGTTSNEENLLPRWKESSEAIKSSAGSIVLHLGKLPIGLCKHRSLLF 297
V+ QLAK +SSRMGGT SNEENLL +WKE +EAIKSS GS+V HLGKLPIGLCKHR+LLF
Sbjct 238 VVIQLAKFISSRMGGTASNEENLLQQWKECTEAIKSSTGSVVFHLGKLPIGLCKHRTLLF 297

Query 298 KMLADKVNIPCRLVKGCKYCKAEDASSCVVRFGLEREYLVDLFGAPGQLSDPDSFVNGP Y 357
KMLADKVNIPCR+VKGCKYCK++DASSC+VRFGLEREYLVDL G PGQL+DPDSFVNGPY
Sbjct 298 KMLADKVNIPCRVVKGCKYCKSDDASSCLVRFGLEREYLVDLIGDPGQLTDPDSFVNGPY 357

Query 358 SLSVPSPLRPPKFRSLEITSNFSSVAKQYFSDCHSLNLLFSDASTGASNGAAVAVDQMYS 417
SLSV SPL P KFRSLEITSNFSSVAKQYFSDCHSLNLLFSD+S G
Sbjct 358 SLSVSSPLHPSKFRSLEITSNFSSVAKQYFSDCHSLNLLFSDSSIG-------------- 403

Query 418 KKHDAGDGIANSWVPVKGQAIANSDIILPEAPREVLPLMSPSNLTADKKKEFQLIEGNQY 477
QA+ DII+PEAPREVLPL++ SN+ DKKKE LI+G Q

Sbjct 404 ------------------QAVMKPDIIVPEAPREVLPLITSSNMKLDKKKELALIDGTQP 445

Query 478 LRSTVSDLSLAVDDLIIPWSELVLKEKIGAGSFGTVHRADWHGSDVAVKILMEQDYHLDR 537

LR+ VSDLSLA DDLIIPWSELVLKEKIGAGSFGTVHRADWHGSDVAVKILMEQD+H +

Sbjct 446 LRNAVSDLSLAADDLIIPWSELVLKEKIGAGSFGTVHRADWHGSDVAVKILMEQDFHPEH 505

Query 538 FKEFMREVAIMKSLRHPNIVLFMGAVTEPPNLSIVTEYLSRGSLYKLLHRSGAREVLDER 597

F+EFMREVAIMKSLRHPNIVLFMGAVTEPPNLSIVTEYLSRGSLYKLLHRSGA+EVLDER

Sbjct 506 FREFMREVAIMKSLRHPNIVLFMGAVTEPPNLSIVTEYLSRGSLYKLLHRSGAKEVLDER 565

Query 598 RRLNMAFDVAKGMNYLHRRSPPIVHRDLKSPNLLVDKKYTVKVCDFGLSRLKANTYLSSK 657

RRLNMAFDVAKGMNYLHRRSPPIVHRDLKSPNLLVDKKYTVKVCDFGLSRLKANT+LSSK

Sbjct 566 RRLNMAFDVAKGMNYLHRRSPPIVHRDLKSPNLLVDKKYTVKVCDFGLSRLKANTFLSSK 625

Query 658 SLAGTPEWMAPEVLRDEPSNEKSDVYSFAVILWELMTLQQPWCNLNPAQVVAAVGFKGRR 717

SLAGTPEWMAPEVLRDEPSNEKSDVYSF VILWELMTLQQPWCNLNPAQVVAAVGFKGRR

Sbjct 626 SLAGTPEWMAPEVLRDEPSNEKSDVYSFGVILWELMTLQQPWCNLNPAQVVAAVGFKGRR 685

Query 718 LEIPKELNPQVAALIESCWANEPWRRPSFANIMETLRPLINKVPVPQLIRSDS 770

LEIPK+LNP VAALIESCWANEPWRRPSF NIMETLRPL+ KVPV QLIRSDS

Sbjct 686 LEIPKDLNPLVAALIESCWANEPWRRPSFTNIMETLRPLL-KVPVAQLIRSDS

737

>Serine/threonine-protein kinase CTR1 [Triticum urartu]
Sequence ID: EMS46799.1 Length: 659
Range 1: 94 to 658

Score:1127 bits(2915), Expect:0.0,
Method:Compositional matrix adjust.,
Identities:558/586(95%), Positives:560/586(95%), Gaps:21/586(3%)

Query 152
VNGSLSYSNTIPDGFYLIQGMDPFVWSMCTDVHEENRIPSVESLKSVRPDDSSIQVVLVD
211

VNGSLSYSNTIPDGFYLIQGMDPFVWSMCTDVHEENRIPSVESLKSVRPDDSSIQVVLVD
Sbjct 94
VNGSLSYSNTIPDGFYLIQGMDPFVWSMCTDVHEENRIPSVESLKSVRPDDSSIQVVLVD
153

Query 212
RRADFDLGMLENYASSFLSSSSDMKDVINQLAKLVSSRMGGTTSNEENLLPRWKESSEAI
271

RRADFDLGMLENYASSFLSSSSDMKDVINQLAKLVSSRMGGTTSNEENLLPRWKESSEAI
Sbjct 154
RRADFDLGMLENYASSFLSSSSDMKDVINQLAKLVSSRMGGTTSNEENLLPRWKESSEAI
213

Query 272
KSSAGSIVLHLGKLPIGLCKHRSLLFKMLADKVNIPCRLVKGCKYCKAEDASSCVVRFGL
331

KSSAGSIVLHLGKLPIGLCKHRSLLFKMLADKVNIPCRLVKGCKYCKAEDASSCVVRFGL
Sbjct 214
KSSAGSIVLHLGKLPIGLCKHRSLLFKMLADKVNIPCRLVKGCKYCKAEDASSCVVRFGL
273

Query 332
EREYLVDLFGAPGQLSDPDSFVNGPYSLSVPSPLRPPKFRSLEITSNFSSVAKQYFSDCH
391
EREYLVDLFGAPGQLSDPDSFVNGPYSLSVPSPLRPPKFR +

Sbjct 274 EREYLVDLFGAPGQLSDPDSFVNGPYSLSVPSPLRPPKFRGVT----------------
316

Query 392
SLNLLFSDASTGASNGAAVAVDQMYSKKHDAGDGIANSWVPVKGQAIANSDIILPEAPRE
451
NL FS
GASNGAAVAVDQMYSKKHDAGDGIANSWVPVKGQAIANSDIILPEAPRE
Sbjct 317
EANLSFS----GASNGAAVAVDQMYSKKHDAGDGIANSWVPVKGQAIANSDIILPEAPRE
372

Query 452
VLPLMSPSNLTADKKKEFQLIEGNQYLRSTVSDLSLAVDDLIIPWSELVLKEKIGAGSFG
511

VLPLM+PSNLTADKKKEFQLIEGNQYLRSTVSDLSLAVDDLIIPWSELVLKEKIGAGSFG
Sbjct 373
VLPLMTPSNLTADKKKEFQLIEGNQYLRSTVSDLSLAVDDLIIPWSELVLKEKIGAGSFG
432

Query 512
TVHRADWHGSDVAVKILMEQDYHLDRFKEFMREVAIMKSLRHPNIVLFMGAVTEPPNLSI
571

TVHRADWHGSDVAVKILMEQDYHLDRFKEFMREVAIMKSLRHPNIVLFMGAVTEPPNLSI
Sbjct 433
TVHRADWHGSDVAVKILMEQDYHLDRFKEFMREVAIMKSLRHPNIVLFMGAVTEPPNLSI
492

Query 572
VTEYLSRGSLYKLLHRSGAREVLDERRRLNMAFDVAKGMNYLHRRSPPIVHRDLKSPNLL
631

VTEYLSRGSLYKLLHRSGAREVLDERRRLNMAFDVAKGMNYLHRRSPPIVHRDLKSPNLL
Sbjct 493
VTEYLSRGSLYKLLHRSGAREVLDERRRLNMAFDVAKGMNYLHRRSPPIVHRDLKSPNLL
552

Query 632
VDKKYTVKVCDFGLSRLKANTYLSSKSLAGTPEWMAPEVLRDEPSNEKSDVYSFAVILW
E 691

VDKKYTVKVCDFGLSRLKANTYLSSKSLAGTPEWMAPEVLRDEPSNEKSDVYSFAVILW

E
Sbjct 553
VDKKYTVKVCDFGLSRLKANTYLSSKSLAGTPEWMAPEVLRDEPSNEKSDVYSFAVILW
E 612

Query 692 LMTLQQPWCNLNPAQVVAAVGFKGRRLEIPKELNPQVAALIESCWA 737
LMTLQQPWCNLNPAQVVAAVGFKGRRLEIPKELNPQVAALIESCWA
Sbjct 613 LMTLQQPWCNLNPAQVVAAVGFKGRRLEIPKELNPQVAALIESCWA 658

>unnamed protein product [Miscanthus lutarioriparius]
Sequence ID: CAD6220040.1 Length: 758
Range 1: 1 to 742

Score:1113 bits(2879), Expect:0.0,
Method:Compositional matrix adjust.,
Identities:606/754(80%), Positives:656/754(87%), Gaps:22/754(2%)

Query 1
MELPAAGGGRRTSYSLLSQFPDDAAAAGASPA-VLQRQSSGGSSYGAGSSVSASSDYPFH
59
M+LP G RRT+YSLL QFPD AAA PA VLQRQSSG S S +
SSDYPFH
Sbjct 1
MDLPP-GAARRTTYSLLPQFPDAAAAPAPVPANVLQRQSSGSSYGAGSSIST-SSDYPFH
58

Query 60
LPPAVAAAG---------GGGGTPSPCKSWAQQAEETYQLQLALALRLCADAACAADPGF 110
L P +AAG G
SPCKSWAQQAEETYQLQLALALRLCADAACAADPGF
Sbjct 59
LQPPASAAGVAPPGSAAAPPGAAGSPCKSWAQQAEETYQLQLALALRLCADAACAADPG
F 118

Query 111
LDPGDSGGSKMGGGGGGSGSGRAFPLAPPSPTAEALSHRFWVNGSLSYSNTIPDGFYLIQ
170
LDPGDSG + SG+GRAFPLA
P+P+AE+LSHRFWVNGSLSY++TIPDGFYLI
Sbjct 119
LDPGDSGSGRG------SGNGRAFPLAQPAPSAESLSHRFWVNGSLSYNSTIPDGFYLIH 172

Query 171
GMDPFVWSMCTDVHEENRIPSVESLKSVRPDDSSIQVVLVDRRADFDLGMLENYASSFLS
230
GMDPFVWS+CTDV EENRIPS+ESLKSVRPDDSSIQ +L+DRR
DF+LGMLE+YASS LS
Sbjct 173
GMDPFVWSLCTDVQEENRIPSMESLKSVRPDDSSIQAILIDRRTDFELGMLESYASSILS
232

Query 231
SSSDMKDVINQLAKLVSSRMGGTTSNEENLLPRWKESSEAIKSSAGSIVLHLGKLPIGLC
290
SS+D KDV+ QLAKL+SSRMGGTTSNEENLL RWKE EAIKSS
GS+VLHLGKLPIGLC
Sbjct 233
SSADAKDVVIQLAKLISSRMGGTTSNEENLLQRWKECIEAIKSSTGSVVLHLGKLPIGLC
292

Query 291
KHRSLLFKMLADKVNIPCRLVKGCKYCKAEDASSCVVRFGLEREYLVDLFGAPGQLSDP
D 350
KHRSLLFK+LADKV+IPCR+VKGCKYCK++DASSC+VRFGLERE+LVDL G
PGQL+DPD
Sbjct 293
KHRSLLFKVLADKVSIPCRVVKGCKYCKSDDASSCLVRFGLEREFLVDLIGDPGQLTDPD
352

Query 351
SFVNGPYSLSVPSPLRPPKFRSLEITSNFSSVAKQYFSDCHSLNLLFSDASTGASNGAAV
410
SFVNGPYSLSV SPL PPKFRSLEITSNF SVAKQ FSDCHSLNLLFSD+STG +N
V
Sbjct 353
SFVNGPYSLSVSSPLHPPKFRSLEITSNFGSVAKQCFSDCHSLNLLFSDSSTGVANSTVV
412

Query 411
AVDQMYSKKHDAGDGIANSWVPVKGQAIANSDIILPEAPREVLPLMSPSNLTADKKKEFQ
470
++D YSKKH AGD + NSWVP KGQA+ DI++PEAPREVLPL++ SN+
DKKKE
Sbjct 413
SLDHPYSKKHVAGDDVMNSWVPGKGQAVMKPDIMVPEAPREVLPLITSSNMKLDKKKEL

V 472

Query 471
LIEGNQYLRSTVSDLSLAVDDLIIPWSELVLKEKIGAGSFGTVHRADWHGSDVAVKILME
530
+ + LR+TVSDLSLA
DDLIIPW+ELVLKEKIGAGSFGTVHRADWHGSDVAVKILME
Sbjct 473
ITQ----LRNTVSDLSLAADDLIIPWNELVLKEKIGAGSFGTVHRADWHGSDVAVKILME
528

Query 531
QDYHLDRFKEFMREVAIMKSLRHPNIVLFMGAVTEPPNLSIVTEYLSRGSLYKLLHRSGA
590
QD+H +RF+EFMREVAIMKSLRHPNIVLFMGAVTEPPNLSIVTEYLSRGSLYK
LHR GA
Sbjct 529
QDFHPERFREFMREVAIMKSLRHPNIVLFMGAVTEPPNLSIVTEYLSRGSLYKFLHRCGA
588

Query 591
REVLDERRRLNMAFDVAKGMNYLHRRSPPIVHRDLKSPNLLVDKKYTVKVCDFGLSRLK
A 650

+EVLDERRRLNMAFDVAKGMNYLHRRSPPIVHRDLKSPNLLVDKKYTVKVCDFGLSRLK
A
Sbjct 589
KEVLDERRRLNMAFDVAKGMNYLHRRSPPIVHRDLKSPNLLVDKKYTVKVCDFGLSRLK
A 648

Query 651
NTYLSSKSLAGTPEWMAPEVLRDEPSNEKSDVYSFAVILWELMTLQQPWCNLNPAQVVA
A 710
NT+LSSKSLAGTPEWMAPEVLRDEPSNEKSDVYSF
VILWELMTLQQPWCNLNPAQVVAA
Sbjct 649
NTFLSSKSLAGTPEWMAPEVLRDEPSNEKSDVYSFGVILWELMTLQQPWCNLNPAQVVA
A 708

Query 711 VGFKGRRLEIPKELNPQVAALIESCWANEPWRRP 744
VGFKGRRLEIPK+LNP VAALIESCWA W P
Sbjct 709 VGFKGRRLEIPKDLNPLVAALIESCWATSAWTSP 742

>uncharacterized protein C2845_PM03G27530 [Panicum miliaceum]
Sequence ID: RLN35053.1 Length: 758
Range 1: 1 to 730

Score:1102 bits(2850), Expect:0.0,
Method:Compositional matrix adjust.,
Identities:613/744(82%), Positives:660/744(88%), Gaps:21/744(2%)

Query 1
MELPAAGGGRRTSYSLLSQFPDDAAAAGASPA-VLQRQSSGGSSYGAGSSVSASSDYPFH
59
MELP G RRT+YS LSQFPDDAAAAG +PA VLQRQSSG S GAGSSVS
SSDYPFH
Sbjct 1
MELPP-GAARRTTYSPLSQFPDDAAAAGPAPANVLQRQSSGSSY-GAGSSVSGSSDYPFH
58

Query 60
LPPAVAAAGGGGGTPSP------CKSWAQQAEETYQLQLALALRLCADAACAADPGFLDP
113
LPP +AA G G +P
CKSWAQQAEETYQLQLALALRLCADAACAADPGFLD
Sbjct 59
LPPPASAAAGPGSAAAPPGGSSPCKSWAQQAEETYQLQLALALRLCADAACAADPGFLD-
117

Query 114
GDSGGSKMGGGGGGGSGSGRAFPLAPPSPTAEALSHRFWVNGSLSYSNTIPDGFYLIQGMD
173
G GG GSG+GRAFPLA P+P+AE+LSHRFWVNGSLSY++TIPDGFY+I
G+D
Sbjct 118
----PGDPGSGGRGSGNGRAFPLAQPAPSAESLSHRFWVNGSLSYNSTIPDGFYVIHGLD
173

Query 174
PFVWSMCTDVHEENRIPSVESLKSVRPDDSSIQVVLVDRRADFDLGMLENYASSFLSSSS
233
PFVWS+CTDVHEENRIP++ESLKSV PDDSSIQ +L+DRR DFDLGMLE+YASS
LSS +
Sbjct 174
PFVWSLCTDVHEENRIPTMESLKSVHPDDSSIQAILIDRRTDFDLGMLESYASSLLSSCT

233

Query 234
DMKDVINQLAKLVSSRMGGTTSNEENLLPRWKESSEAIKSSAGSIVLHLGKLPIGLCKHR
293
D KDV+ QLAK+VSSRMGGT SNEENL PRWKE +EAIKSS
GS+VLHLGKLP+GLCKHR
Sbjct 234
DAKDVVIQLAKVVSSRMGGTASNEENLFPRWKECNEAIKSSTGSVVLHLGKLPVGLCKH
R 293

Query 294
SLLFKMLADKVNIPCRLVKGCKYCKAEDASSCVVRFGLEREYLVDLFGAPGQLSDPDSFV
353
SLLFK+LADKV+IPCR+VKGCKYCK++DASSC+VRFGLEREYLVDL G
PGQLSDPDSFV
Sbjct 294
SLLFKILADKVSIPCRVVKGCKYCKSDDASSCLVRFGLEREYLVDLIGDPGQLSDPDSFV
353

Query 354
NGPYSLSVPSPLRPPKFRSLEITSNFSSVAKQYFSDCHSLNLLFSDASTGASNGAAVAVD
413
NGPYSLSV SPL PPKFRSLEITSNFSSVAKQYFSDCHSLNLLFSD+STGA++ A
+D
Sbjct 354
NGPYSLSVSSPLHPPKFRSLEITSNFSSVAKQYFSDCHSLNLLFSDSSTGATS-AVTTLD 412

Query 414
QMYSKKHDAGDGIANSWVPVKGQAIANSDIILPEAPREVLPLMSPSNLTADKKKEFQLIE
473
Q YSKKH AGD N+W+ KGQA II+PEAPREVLPL+S SN+ KE
+LIE
Sbjct 413
QPYSKKHVAGDETMNNWMTGKGQAAMKPGIIVPEAPREVLPLIS-SNM-----KELKLIE
466

Query 474
GNQYLRSTVSDLSLAVDDLIIPWSELVLKEKIGAGSFGTVHRADWHGSDVAVKILMEQDY
533
Q LR TVSDLSLA
DDLIIPW+ELVLKEKIGAGSFGTVHRADWHGSDVAVKILMEQD+
Sbjct 467
ETQQLRHTVSDLSLAADDLIIPWNELVLKEKIGAGSFGTVHRADWHGSDVAVKILMEQDF

526

Query 534
HLDRFKEFMREVAIMKSLRHPNIVLFMGAVTEPPNLSIVTEYLSRGSLYKLLHRSGAREV
593
H +
F+EFMREVAIMKSLRHPNIVLFMGAVTEPPNLSIVTEYLSRGSLYKLLHRSGAREV
Sbjct 527
HPECFREFMREVAIMKSLRHPNIVLFMGAVTEPPNLSIVTEYLSRGSLYKLLHRSGAREV
586

Query 594
LDERRRLNMAFDVAKGMNYLHRRSPPIVHRDLKSPNLLVDKKYTVKVCDFGLSRLKANT
Y 653

LDERRRLNMAFDVAKGMNYLHRRSPP+VHRDLKSPNLLVDKKYTVKVCDFGLSRLKAN
T+
Sbjct 587
LDERRRLNMAFDVAKGMNYLHRRSPPVVHRDLKSPNLLVDKKYTVKVCDFGLSRLKAN
TF 646

Query 654
LSSKSLAGTPEWMAPEVLRDEPSNEKSDVYSFAVILWELMTLQQPWCNLNPAQVVAAVGF
713
LSSKSLAGTPEWMAPEVLRDEPSNEKSDVYSF
VILWELMTLQQPWCNLNPAQVVAAVGF
Sbjct 647
LSSKSLAGTPEWMAPEVLRDEPSNEKSDVYSFGVILWELMTLQQPWCNLNPAQVVAAVG
F 706

Query 714 KGRRLEIPKELNPQVAALIESCWA 737
KGRRLEIPK+LNP VAALIESCWA
Sbjct 707 KGRRLEIPKDLNPLVAALIESCWA 730

>unnamed protein product [Miscanthus lutarioriparius]
Sequence ID: CAD6225707.1 Length: 760
Range 1: 1 to 736

Score:1100 bits(2844), Expect:0.0,
Method:Compositional matrix adjust.,
Identities:603/748(81%), Positives:654/748(87%), Gaps:22/748(2%)

Query 1
MELPAAGGGRRTSYSLLSQFPDDAAAAGASPA-VLQRQSSGGSSYGAGSSVSASSDYPFH
59
M+LP G RRT+YSLL QFPDDAAA +PA VLQRQSSG S S +
SSDYPFH
Sbjct 1
MDLPP-GAARRTTYSLLPQFPDDAAAPAPAPANVLQRQSSGSSYGAGSSIST-SSDYPFH
58

Query 60
LPPAVAAAG---------GGGGTPSPCKSWAQQAEETYQLQLALALRLCADAACAADPGF 110
L P + AG G
SPCKSWAQQAEETYQLQLALALRLCADAACAADPGF
Sbjct 59
LQPPASPAGVAPPGSAAAPPGAAGSPCKSWAQQAEETYQLQLALALRLCADAACAADPG
F 118

Query 111
LDPGDSGGSKMGGGGGGGSGSGRAFPLAPPSPTAEALSHRFWVNGSLSYSNTIPDGFYLIQ
170
LDPGDSG + SG+GRAFPLA
P+P+AE+LSHRFWVNGSLSY++TIPDGFYLI
Sbjct 119
LDPGDSGSGRG------SGNGRAFPLAQPAPSAESLSHRFWVNGSLSYNSTIPDGFYLIH 172

Query 171
GMDPFVWSMCTDVHEENRIPSVESLKSVRPDDSSIQVVLVDRRADFDLGMLENYASSFLS
230
GMDPFVWS+CTDV EENRIPS+ESLKSVRPDDSSIQ +L+DRR
DF+LGMLE+YASS LS
Sbjct 173
GMDPFVWSLCTDVQEENRIPSMESLKSVRPDDSSIQAILIDRRTDFELGMLESYASSILS
232

Query 231
SSSDMKDVINQLAKLVSSRMGGTTSNEENLLPRWKESSEAIKSSAGSIVLHLGKLPIGLC
290
SS+D KDV+ QLAKL+SSRMGGTTSNEENLL RWKE EAIKSS
GS+VLHLGKLPIGLC
Sbjct 233
SSADAKDVVIQLAKLISSRMGGTTSNEENLLQRWKECIEAIKSSTGSVVLHLGKLPIGLC
292

Query 291
KHRSLLFKMLADKVNIPCRLVKGCKYCKAEDASSCVVRFGLEREYLVDLFGAPGQLSDP
D 350
KHRSLLFK+LADKV+IPCR+VKGCKYCK++DASSC+VRFGLERE+LVDL G
G L+DPD
Sbjct 293
KHRSLLFKVLADKVSIPCRVVKGCKYCKSDDASSCLVRFGLEREFLVDLIGDRGPLTDPD
352

Query 351
SFVNGPYSLSVPSPLRPPKFRSLEITSNFSSVAKQYFSDCHSLNLLFSDASTGASNGAAV
410
SFVNGPYSLSV SPL PPKFRSLEITSNF SVAKQYFSDCHSLNLLF+D+STG
+NG V
Sbjct 353
SFVNGPYSLSVSSPLHPPKFRSLEITSNFGSVAKQYFSDCHSLNLLFNDSSTGVANGTVV
412

Query 411
AVDQMYSKKHDAGDGIANSWVPVKGQAIANSDIILPEAPREVLPLMSPSNLTADKKKEFQ
470
++D YSKKH AGD + NSWVP KGQA+ DI++PEAPREVLPL++ SN+
DKKKE
Sbjct 413
SLDHPYSKKHVAGDDVMNSWVPGKGQAVMKPDIMVPEAPREVLPLITSSNMKLDKKKEL
V 472

Query 471
LIEGNQYLRSTVSDLSLAVDDLIIPWSELVLKEKIGAGSFGTVHRADWHGSDVAVKILME
530
+ L +TVSDLSLA
DDLIIPW+ELVLKEKIGAGSFGTVHRADWHGSDVAVKILME
Sbjct 473
TPQ----LWNTVSDLSLAADDLIIPWNELVLKEKIGAGSFGTVHRADWHGSDVAVKILME
528

Query 531
QDYHLDRFKEFMREVAIMKSLRHPNIVLFMGAVTEPPNLSIVTEYLSRGSLYKLLHRSGA
590
QD+H
+RF+EFMREVAIMKSLRHPNIVLFMGAVTEPPNLSIVTEYLSRGSLYKLLHRSGA
Sbjct 529
QDFHPERFREFMREVAIMKSLRHPNIVLFMGAVTEPPNLSIVTEYLSRGSLYKLLHRSGA
588

Query 591
REVLDERRRLNMAFDVAKGMNYLHRRSPPIVHRDLKSPNLLVDKKYTVKVCDFGLSRLK
A 650

+EVLDERRRLNMAFDVAKGMNYLHRRSPPIVHRDLKSPNLLVDKKYTVKVCDFGLSRLK
A
Sbjct 589
KEVLDERRRLNMAFDVAKGMNYLHRRSPPIVHRDLKSPNLLVDKKYTVKVCDFGLSRLK
A 648

Query 651
NTYLSSKSLAGTPEWMAPEVLRDEPSNEKSDVYSFAVILWELMTLQQPWCNLNPAQVVA
A 710
NT+LSSKSLAGTPEWMAPEVLRDEPSNEKSDVYSF
VILWELMTLQQPWCNLNPAQVVAA
Sbjct 649
NTFLSSKSLAGTPEWMAPEVLRDEPSNEKSDVYSFGVILWELMTLQQPWCNLNPAQVVA
A 708

Query 711 VGFKGRRLEIPKELNPQVAALIESCWAN 738
VGFKGRRLEIPK+LNP VAALIE CWA
Sbjct 709 VGFKGRRLEIPKDLNPLVAALIECCWAT 736

>unnamed protein product [Triticum turgidum subsp. durum]
Sequence ID: VAI00709.1 Length: 546
Range 1: 1 to 546

Score:1090 bits(2818), Expect:0.0,
Method:Compositional matrix adjust.,
Identities:536/567(95%), Positives:541/567(95%), Gaps:21/567(3%)

Query 172
MDPFVWSMCTDVHEENRIPSVESLKSVRPDDSSIQVVLVDRRADFDLGMLENYASSFLSS
231

MDPFVWSMCTDVHEENRIPSVESLKSVRPDDSSIQVVLVDRRADFDLGMLENYASSFLSS
Sbjct 1
MDPFVWSMCTDVHEENRIPSVESLKSVRPDDSSIQVVLVDRRADFDLGMLENYASSFLSS
60

Query 232
SSDMKDVINQLAKLVSSRMGGTTSNEENLLPRWKESSEAIKSSAGSIVLHLGKLPIGLCK
291

SSDMKDVINQLAKLVSSRMGGTTSNEENLLPRWKESSEAIKSSAGSIVLHLGKLPIGLCK
Sbjct 61
SSDMKDVINQLAKLVSSRMGGTTSNEENLLPRWKESSEAIKSSAGSIVLHLGKLPIGLCK
120

Query 292
HRSLLFKMLADKVNIPCRLVKGCKYCKAEDASSCVVRFGLEREYLVDLFGAPGQLSDPDS
351

HRSLLFKMLADKVNIPCRLVKGCKYCKAEDASSCVVRFGLEREYLVDLFGAPGQLSDPDS
Sbjct 121
HRSLLFKMLADKVNIPCRLVKGCKYCKAEDASSCVVRFGLEREYLVDLFGAPGQLSDPDS
180

Query 352
FVNGPYSLSVPSPLRPPKFRSLEITSNFSSVAKQYFSDCHSLNLLFSDASTGASNGAAVA
411
FVNGPYSLSVPSPLRPPKFR + T+ HS
+GASNGAAVA
Sbjct 181 FVNGPYSLSVPSPLRPPKFRGVTKTN-------------HSF--------SGASNGAAVA
219

Query 412
VDQMYSKKHDAGDGIANSWVPVKGQAIANSDIILPEAPREVLPLMSPSNLTADKKKEFQL
471

VDQMYSKKHDAGDGIANSWVPVKGQAIANSDIILPEAPREVLPLM+PSNLTADKKKEFQL
Sbjct 220
VDQMYSKKHDAGDGIANSWVPVKGQAIANSDIILPEAPREVLPLMTPSNLTADKKKEFQL
279

Query 472
IEGNQYLRSTVSDLSLAVDDLIIPWSELVLKEKIGAGSFGTVHRADWHGSDVAVKILMEQ
531

IEGNQYLRSTVSDLSLAVDDLIIPWSELVLKEKIGAGSFGTVHRADWHGSDVAVKILMEQ
Sbjct 280
IEGNQYLRSTVSDLSLAVDDLIIPWSELVLKEKIGAGSFGTVHRADWHGSDVAVKILMEQ
339

Query 532
DYHLDRFKEFMREVAIMKSLRHPNIVLFMGAVTEPPNLSIVTEYLSRGSLYKLLHRSGAR
591

DYHLDRFKEFMREVAIMKSLRHPNIVLFMGAVTEPPNLSIVTEYLSRGSLYKLLHRSGAR
Sbjct 340
DYHLDRFKEFMREVAIMKSLRHPNIVLFMGAVTEPPNLSIVTEYLSRGSLYKLLHRSGAR
399

Query 592
EVLDERRRLNMAFDVAKGMNYLHRRSPPIVHRDLKSPNLLVDKKYTVKVCDFGLSRLKA
N 651

EVLDERRRLNMAFDVAKGMNYLHRRSPPIVHRDLKSPNLLVDKKYTVKVCDFGLSRLKA
N
Sbjct 400
EVLDERRRLNMAFDVAKGMNYLHRRSPPIVHRDLKSPNLLVDKKYTVKVCDFGLSRLKA
N 459

Query 652
TYLSSKSLAGTPEWMAPEVLRDEPSNEKSDVYSFAVILWELMTLQQPWCNLNPAQVVAAV
711

TYLSSKSLAGTPEWMAPEVLRDEPSNEKSDVYSFAVILWELMTLQQPWCNLNPAQVVAAV
Sbjct 460
TYLSSKSLAGTPEWMAPEVLRDEPSNEKSDVYSFAVILWELMTLQQPWCNLNPAQVVAAV
519

Query 712 GFKGRRLEIPKELNPQVAALIESCWAN 738
GFKGRRLEIPK+LNPQVA LIESCWAN
Sbjct 520 GFKGRRLEIPKDLNPQVAVLIESCWAN 546

>hypothetical protein PVAP13_2KG218100 [Panicum virgatum]
Sequence ID: KAG2641915.1 Length: 727
Range 1: 1 to 712

Score:1053 bits(2723), Expect:0.0,
Method:Compositional matrix adjust.,
Identities:582/721(81%), Positives:631/721(87%), Gaps:20/721(2%)

Query 1

MELPAAGGGRRTSYSLLSQFPDDAAAAGASP-----AVLQRQSSGGSSYGAGSSVSASSD 55

MELP G RRT+YSLLSQFPDDAA AG P + LQRQSSG S G GSSVSASSD

Sbjct 1 MELPP-GAARRTNYSLLSQFPDDAAGAGPGPGPAPASALQRQSSGSSY-GGGSSVSASSD 58

Query 56 YPFHLPPAVAAAGGGGGTPSP------CKSWAQQAEETYQLQLALALRLCADAACAADPG 109

YPFHLPP +AA G G + CKSWAQQAEETYQLQLALALRLCADAACAADPG

Sbjct 59 YPFHLPPPASAAAGPGSAAAAPGGSSPCKSWAQQAEETYQLQLALALRLCADAACAADPG 118

Query 110 FLDPGDSGGSKMGGGGGGSGSGRAFPLAPPSPTAEALSHRFWVNGSLSYSNTIPDGFYLI 169

FLD G GG GSG+GRAFPLA P+P+AE+LSHRFWVNGSLSY++TIPDGFY+I

Sbjct 119 FLD-----PGDPGSGGRGSGNGRAFPLAQPAPSAESLSHRFWVNGSLSYNSTIPDGFYVI 173

Query 170 QGMDPFVWSMCTDVHEENRIPSVESLKSVRPDDSSIQVVLVDRRADFDLGMLENYASSFL 229

G+DPFVWS+CTDVHEEN IPS+ESLKSVRPDDSSIQ +L+DRR DFDLGMLE+YASS L

Sbjct 174 HGLDPFVWSLCTDVHEENHIPSMESLKSVRPDDSSIQAILIDRRTDFDLGMLESYASSLL 233

Query 230 SSSSDMKDVINQLAKLVSSRMGGTTSNEENLLPRWKESSEAIKSSAGSIVLHLGKLPIGL 289

SS +D KDV+ QLAK+VSSRMGGT SNE++L +WKE +EAIKSS GS+VLHLGKLP+GL

Sbjct 234 SSCTDAKDVVIQLAKVVSSRMGGTASNEDSLFQKWKECNEAIKSSTGSVVLHLGKLPVGL 293

Query 290

CKHRSLLFKMLADKVNIPCRLVKGCKYCKAEDASSCVVRFGLEREYLVDLFGAPGQLSDP
349
CKHRSLLFK+LADKV+IPCR+VKGCKYCK++DASSC+VRFGLEREYLVDL G
PGQLSDP
Sbjct 294
CKHRSLLFKILADKVSIPCRVVKGCKYCKSDDASSCLVRFGLEREYLVDLIGDPGQLSDP
353

Query 350
DSFVNGPYSLSVPSPLRPPKFRSLEITSNFSSVAKQYFSDCHSLNLLFSDASTGASNGAA
409
DSFVNGPYSLSV SPL
PPKFRSLEITSNFSSVAKQYFSDCHSLNLLFSD+STGA++ A
Sbjct 354
DSFVNGPYSLSVSSPLHPPKFRSLEITSNFSSVAKQYFSDCHSLNLLFSDSSTGATS-AV 412

Query 410
VAVDQMYSKKHDAGDGIANSWVPVKGQAIANSDIILPEAPREVLPLMSPSNLTADKKKEF
469
+DQ YSKKH AGD N+W+P KGQA DII+ EAPREVLPL+S SN+
D+KKE
Sbjct 413
TTLDQPYSKKHVAGDETMNNWMPGKGQAAIKPDIIVQEAPREVLPLIS-SNMKLDRKKEL
471

Query 470
QLIEGNQYLRSTVSDLSLAVDDLIIPWSELVLKEKIGAGSFGTVHRADWHGSDVAVKILM
529
+LIE Q LR TVSDLSLA
DDLIIPW+ELVLKEKIGAGSFGTVHRADWHGSDVAVKILM
Sbjct 472
KLIEETQQLRHTVSDLSLAADDLIIPWNELVLKEKIGAGSFGTVHRADWHGSDVAVKILM
531

Query 530
EQDYHLDRFKEFMREVAIMKSLRHPNIVLFMGAVTEPPNLSIVTEYLSRGSLYKLLHRSG
589
EQD+H
+RF+EFMREVAIMKSLRHPNIVLFMGAVTEPPNLSIVTEYLSRGSLYKLLHRSG
Sbjct 532
EQDFHPERFREFMREVAIMKSLRHPNIVLFMGAVTEPPNLSIVTEYLSRGSLYKLLHRSG
591

Query 590

AREVLDERRRLNMAFDVAKGMNYLHRRSPPIVHRDLKSPNLLVDKKYTVKVCDFGLSRL
K 649

A+EVLDERRRLNMAFDVAKGMNYLHRRSPPIVHRDLKSPNLLVDKKYTVKVCDFGLSRL
K
Sbjct 592
AKEVLDERRRLNMAFDVAKGMNYLHRRSPPIVHRDLKSPNLLVDKKYTVKVCDFGLSRL
K 651

Query 650
ANTYLSSKSLAGTPEWMAPEVLRDEPSNEKSDVYSFAVILWELMTLQQPWCNLNPAQVV
A 709
NT+LSSKSLAGTPEWMAPEVLRDEPSNEKSDVYSF
VILWELMTLQQPWCNLNPAQ
Sbjct 652
PNTFLSSKSLAGTPEWMAPEVLRDEPSNEKSDVYSFGVILWELMTLQQPWCNLNPAQRA
M 711

Query 710 A 710
A
Sbjct 712 A 712

>hypothetical protein PVAP13_2KG218100 [Panicum virgatum]
Sequence ID: KAG2641916.1 Length: 749
Range 1: 1 to 709

Score:1050 bits(2714), Expect:0.0,
Method:Compositional matrix adjust.,
Identities:581/718(81%), Positives:631/718(87%), Gaps:20/718(2%)

Query 1
MELPAAGGGRRTSYSLLSQFPDDAAAAGASP-----AVLQRQSSGGSSYGAGSSVSASSD
55
MELP G RRT+YSLLSQFPDDAA AG P + LQRQSSG S G
GSSVSASSD
Sbjct 1
MELPP-GAARRTNYSLLSQFPDDAAGAGPGPGPAPASALQRQSSGSSY-GGGSSVSASSD
58

Query 56
YPFHLPPAVAAAGGGGGTPSP------CKSWAQQAEETYQLQLALALRLCADAACAADPG

109

YPFHLPP +AA G G + CKSWAQQAEETYQLQLALALRLCADAACAADPG

Sbjct 59 YPFHLPPPASAAAGPGSAAAAPGGSSPCKSWAQQAEETYQLQLALALRLCADAACAADPG 118

Query 110 FLDPGDSGGSKMGGGGGGSGSGRAFPLAPPSPTAEALSHRFWVNGSLSYSNTIPDGFYLI 169

FLD G GG GSG+GRAFPLA P+P+AE+LSHRFWVNGSLSY++TIPDGFY+I

Sbjct 119 FLD-----PGDPGSGGRGSGNGRAFPLAQPAPSAESLSHRFWVNGSLSYNSTIPDGFYVI 173

Query 170 QGMDPFVWSMCTDVHEENRIPSVESLKSVRPDDSSIQVVLVDRRADFDLGMLENYASSFL 229

G+DPFVWS+CTDVHEEN IPS+ESLKSVRPDDSSIQ +L+DRR DFDLGMLE+YASS L

Sbjct 174 HGLDPFVWSLCTDVHEENHIPSMESLKSVRPDDSSIQAILIDRRTDFDLGMLESYASSLL 233

Query 230 SSSSDMKDVINQLAKLVSSRMGGTTSNEENLLPRWKESSEAIKSSAGSIVLHLGKLPIGL 289

SS +D KDV+ QLAK+VSSRMGGT SNE++L +WKE +EAIKSS GS+VLHLGKLP+GL

Sbjct 234 SSCTDAKDVVIQLAKVVSSRMGGTASNEDSLFQKWKECNEAIKSSTGSVVLHLGKLPVGL 293

Query 290 CKHRSLLFKMLADKVNIPCRLVKGCKYCKAEDASSCVVRFGLEREYLVDLFGAPGQLSDP 349

CKHRSLLFK+LADKV+IPCR+VKGCKYCK++DASSC+VRFGLEREYLVDL G PGQLSDP

Sbjct 294 CKHRSLLFKILADKVSIPCRVVKGCKYCKSDDASSCLVRFGLEREYLVDLIGDPGQLSDP 353

Query 350 DSFVNGPYSLSVPSPLRPPKFRSLEITSNFSSVAKQYFSDCHSLNLLFSDASTGASNGAA

409

DSFVNGPYSLSV SPL PPKFRSLEITSNFSSVAKQYFSDCHSLNLLFSD+STGA++ A

Sbjct 354 DSFVNGPYSLSVSSPLHPPKFRSLEITSNFSSVAKQYFSDCHSLNLLFSDSSTGATS-AV 412

Query 410 VAVDQMYSKKHDAGDGIANSWVPVKGQAIANSDIILPEAPREVLPLMSPSNLTADKKKEF 469

+DQ YSKKH AGD N+W+P KGQA DII+ EAPREVLPL+S SN+ D+KKE

Sbjct 413 TTLDQPYSKKHVAGDETMNNWMPGKGQAAIKPDIIVQEAPREVLPLIS-SNMKLDRKKEL 471

Query 470 QLIEGNQYLRSTVSDLSLAVDDLIIPWSELVLKEKIGAGSFGTVHRADWHGSDVAVKILM 529

+LIE Q LR TVSDLSLA DDLIIPW+ELVLKEKIGAGSFGTVHRADWHGSDVAVKILM

Sbjct 472 KLIEETQQLRHTVSDLSLAADDLIIPWNELVLKEKIGAGSFGTVHRADWHGSDVAVKILM 531

Query 530 EQDYHLDRFKEFMREVAIMKSLRHPNIVLFMGAVTEPPNLSIVTEYLSRGSLYKLLHRSG 589

EQD+H +RF+EFMREVAIMKSLRHPNIVLFMGAVTEPPNLSIVTEYLSRGSLYKLLHRSG

Sbjct 532 EQDFHPERFREFMREVAIMKSLRHPNIVLFMGAVTEPPNLSIVTEYLSRGSLYKLLHRSG 591

Query 590 AREVLDERRRLNMAFDVAKGMNYLHRRSPPIVHRDLKSPNLLVDKKYTVKVCDFGLSRLK 649

A+EVLDERRRLNMAFDVAKGMNYLHRRSPPIVHRDLKSPNLLVDKKYTVKVCDFGLSRLK

Sbjct 592 AKEVLDERRRLNMAFDVAKGMNYLHRRSPPIVHRDLKSPNLLVDKKYTVKVCDFGLSRLK 651

Query 650

ANTYLSSKSLAGTPEWMAPEVLRDEPSNEKSDVYSFAVILWELMTLQQPWCNLNPAQV 707
NT+LSSKSLAGTPEWMAPEVLRDEPSNEKSDVYSF VILWELMTLQQPWCNLNPAQ+
Sbjct 652 PNTFLSSKSLAGTPEWMAPEVLRDEPSNEKSDVYSFGVILWELMTLQQPWCNLNPAQL 709

>hypothetical protein OsJ_30383 [Oryza sativa Japonica Group]
Sequence ID: EAZ45706.1 Length: 710
Range 1: 1 to 576

Score:1048 bits(2710), Expect:0.0,
Method:Compositional matrix adjust.,
Identities:504/576(88%), Positives:543/576(94%), Gaps:10/576(1%)

Query 172 MDPFVWSMCTDVHEENRIPSVESLKSVRPDDSSIQVVLVDRRADFDLGMLENYASSFLSS 231
MDPFVWS+CTD+ EENRIPS++SLKSVRPDDSS+Q +L+DRR DFDLGMLENYASSFLSS
Sbjct 1 MDPFVWSLCTDLLEENRIPSIDSLKSVRPDDSSMQAILIDRRTDFDLGMLENYASSFLSS 60

Query 232 SSDMKDVINQLAKLVSSRMGGTTSNEENLLPRWKESSEAIKSSAGSIVLHLGKLPIGLCK 291
S+DMKDVINQLAKLVSSRMGGTTSNEE+ LPRWKE S+AIKSS GSIVLHLGKLPIG CK
Sbjct 61 SADMKDVINQLAKLVSSRMGGTTSNEESFLPRWKECSDAIKSSTGSIVLHLGKLPIGFCK 120

Query 292 HRSLLFKMLADKVNIPCRLVKGCKYCKAEDASSCVVRFGLEREYLVDLFGAPGQLSDPDS 351
HRSLLFKMLADKVN+PCR+VKGCKYCK++DA+SC+VRFGLEREYLVDL G PGQLSDPDS
Sbjct 121 HRSLLFKMLADKVNVPCRVVKGCKYCKSDDATSCLVRFGLEREYLVDLIGDPGQLSDPDS

180

Query 352
FVNGPYSLSVPSPLRPPKFRSLEITSNFSSVAKQYFSDCHSLNLLFSDASTGASNGAAVA
411

FVNGPYSLSVPSPLRPPKFRSLEITSNFSSVAKQYFSDCHSLNLLF++ASTGA++ AAVA
Sbjct 181
FVNGPYSLSVPSPLRPPKFRSLEITSNFSSVAKQYFSDCHSLNLLFNEASTGANSNAAVA
240

Query 412
VDQMYS-KKHDAGDGIANSWVPVKG---------QAIANSDIILPEAPREVLPLMSPSNL 461
+DQ YS +KHD D I +SWVPVK QA +SD ILPEAPREVLPL++
SNL
Sbjct 241
MDQPYSTRKHDTRDDIMSSWVPVKAYIHIMAQQSQAAVSSDAILPEAPREVLPLITSSNL
300

Query 462
TADKKKEFQLIEGNQYLRSTVSDLSLAVDDLIIPWSELVLKEKIGAGSFGTVHRADWHGS
521

A+KKKEF+LIEGNQYLRSTVSDLSLAVDDLIIPW+EL+LKEKIGAGSFGTVHRADW+GS
Sbjct 301
KAEKKKEFKLIEGNQYLRSTVSDLSLAVDDLIIPWNELILKEKIGAGSFGTVHRADWNGS
360

Query 522
DVAVKILMEQDYHLDRFKEFMREVAIMKSLRHPNIVLFMGAVTEPPNLSIVTEYLSRGSL
581
DVAVKILMEQD+H
DRF+EFMREVAIMKSLRHPNIVLFMGAVTEPPNLSIVTEYLSRGSL
Sbjct 361
DVAVKILMEQDFHPDRFREFMREVAIMKSLRHPNIVLFMGAVTEPPNLSIVTEYLSRGSL
420

Query 582
YKLLHRSGAREVLDERRRLNMAFDVAKGMNYLHRRSPPIVHRDLKSPNLLVDKKYTVKV
C 641

YKLLHRSGA+EVLDERRRLNMAFDVAKGMNYLH+RSPPIVHRDLKSPNLLVDKKYTVKV
C
Sbjct 421

YKLLHRSGAKEVLDERRRLNMAFDVAKGMNYLHKRSPPIVHRDLKSPNLLVDKKYTVK
VC 480

Query 642
DFGLSRLKANTYLSSKSLAGTPEWMAPEVLRDEPSNEKSDVYSFAVILWELMTLQQPWC
N 701
DFGLSRLKANT+LSSKSLAGTPEWMAPEVLRDEPSNEKSDVYSF
VILWELMT+QQPWCN
Sbjct 481
DFGLSRLKANTFLSSKSLAGTPEWMAPEVLRDEPSNEKSDVYSFGVILWELMTMQQPWC
N 540

Query 702 LNPAQVVAAVGFKGRRLEIPKELNPQVAALIESCWA 737
LNPAQVVAAVGFKGRRL+IPK+LNPQVAALIESCWA
Sbjct 541 LNPAQVVAAVGFKGRRLDIPKDLNPQVAALIESCWA 576

>hypothetical protein SEVIR_2G137201v2 [Setaria viridis]
Sequence ID: TKW31910.1 Length: 550
Range 1: 1 to 550

Score:973 bits(2516), Expect:0.0,
Method:Compositional matrix adjust.,
Identities:483/551(88%), Positives:513/551(93%), Gaps:1/551(0%)

Query 220
MLENYASSFLSSSSDMKDVINQLAKLVSSRMGGTTSNEENLLPRWKESSEAIKSSAGSIV
279
MLE+YASS SS +D KDV+ QLAK+VSSRMGGTTSNEENLLPRWKE
+EAIKSSAGS+V
Sbjct 1
MLESYASSLSSSCTDAKDVVTQLAKVVSSRMGGTTSNEENLLPRWKECNEAIKSSAGSVV
60

Query 280
LHLGKLPIGLCKHRSLLFKMLADKVNIPCRLVKGCKYCKAEDASSCVVRFGLEREYLVDL
339

LHLGKLP+GLCKHRSLLFKMLADKV+IPCR+VKGCKYCK++DASSC+VRFGLEREYLVDL
Sbjct 61
LHLGKLPVGLCKHRSLLFKMLADKVSIPCRVVKGCKYCKSDDASSCLVRFGLEREYLVDL
120

Query 340
FGAPGQLSDPDSFVNGPYSLSVPSPLRPPKFRSLEITSNFSSVAKQYFSDCHSLNLLFSD
399
G PGQLSDPDSFVNGPYSLSV SPL
PPKFRSLEITSNFSSVAKQYFSDCHSLNLLFSD
Sbjct 121
IGDPGQLSDPDSFVNGPYSLSVSSPLHPPKFRSLEITSNFSSVAKQYFSDCHSLNLLFSD
180

Query 400
ASTGASNGAAVAVDQMYSKKHDAGDGIANSWVPVKGQAIANSDIILPEAPREVLPLMSPS
459
+STGA+ A +DQ YSKKH AGD I NSW+P KGQA DII+PEAPREVLPL++
S
Sbjct 181
SSTGAATSAVTTLDQPYSKKHIAGDEIINSWMPGKGQAAIKPDIIVPEAPREVLPLITSS
240

Query 460
NLTADKKKEFQLIEGNQYLRSTVSDLSLAVDDLIIPWSELVLKEKIGAGSFGTVHRADWH
519
N+ DKKKE +LIE Q+LR TVSDLSLA
DDLIIPW+EL+LKEKIGAGSFGTVHRADWH
Sbjct 241
NIKLDKKKELKLIEETQHLRHTVSDLSLAADDLIIPWNELILKEKIGAGSFGTVHRADWH
300

Query 520
GSDVAVKILMEQDYHLDRFKEFMREVAIMKSLRHPNIVLFMGAVTEPPNLSIVTEYLSRG
579
GSDVAVKILMEQD+H
+RF+EFMREVAIMKSLRHPNIVLFMGAVTEPPNLSIVTEYLSRG
Sbjct 301
GSDVAVKILMEQDFHPERFREFMREVAIMKSLRHPNIVLFMGAVTEPPNLSIVTEYLSRG
360

Query 580
SLYKLLHRSGAREVLDERRRLNMAFDVAKGMNYLHRRSPPIVHRDLKSPNLLVDKKYTV
K 639

SLYKLLHRSGA+EVLDERRRLNMAFDVAKGMNYLHRRSPPIVHRDLKSPNLLVDKKYTV
K
Sbjct 361

SLYKLLHRSGAKEVLDERRRLNMAFDVAKGMNYLHRRSPPIVHRDLKSPNLLVDKKYTV
K 420

Query 640
VCDFGLSRLKANTYLSSKSLAGTPEWMAPEVLRDEPSNEKSDVYSFAVILWELMTLQQPW
699
VCDFGLSRLKANT+LSSKSLAGTPEWMAPEVLRDEPSNEKSDVYSF
VILWELMTLQQPW
Sbjct 421
VCDFGLSRLKANTFLSSKSLAGTPEWMAPEVLRDEPSNEKSDVYSFGVILWELMTLQQP
W 480

Query 700
CNLNPAQVVAAVGFKGRRLEIPKELNPQVAALIESCWANEPWRRPSFANIMETLRPLINK
759
CNLNPAQVVAAVGFKGRRLEIPK+LNP VAALIESCWANEPWRRPSF NIME
LRPLI K
Sbjct 481
CNLNPAQVVAAVGFKGRRLEIPKDLNPLVAALIESCWANEPWRRPSFTNIMEALRPLI-K
539

Query 760 VPVPQLIRSDS 770
VPVPQLIRSDS
Sbjct 540 VPVPQLIRSDS 550

>serine/threonine-protein kinase CTR1 isoform X1 [Iris pallida]
Sequence ID: KAJ6802525.1 Length: 744
>serine/threonine-protein kinase CTR1 isoform X1 [Iris pallida]
Sequence ID: KAJ6827320.1 Length: 744
Range 1: 1 to 739

Score:956 bits(2472), Expect:0.0,
Method:Compositional matrix adjust.,
Identities:495/776(64%), Positives:594/776(76%), Gaps:44/776(5%)

Query 1
MELPAAGGGRRTSYSLLSQFPDDAAAAGASPAVLQRQSSGGSSYGAGSSVSASSDYPFHL
60
ME+P RR++YSLLS +PDD LQRQSSG S + +++DY F
Sbjct 1
MEIP----NRRSNYSLLSHYPDDPPHQKFDS--LQRQSSGSSFGESSLLSLSAADYCF-- 52

Query 61
PPAVAAAGGGGGTP-------SPCKSWAQQAEETYQLQLALALRLCADAACAADPGFLDP
113
PP +A GG +P S KSWAQQAEE YQLQLALALRLC+DAACA DP
LD
Sbjct 53
PPTDPSAPVGGPSPAAPASYPSSVKSWAQQAEEAYQLQLALALRLCSDAACATDPNLLDS
112

Query 114
GDSGGSKMGGGGGGSGSGRAFPLAPPSPTAEALSHRFWVNGSLSYSNTIPDGFYLIQGMD
173
+G + G +P+AEA+SHRFWVNG LSY +
+PDGFYLIQGMD
Sbjct 113 SPAG--EFG----------------APSAEAISHRFWVNGCLSYHDRVPDGFYLIQGMD
153

Query 174
PFVWSMCTDVHEENRIPSVESLKSVRPDDSSIQVVLVDRRADFDLGMLENYASSFLSSSS
233
PFVW++CTDV EE+RIPS++SLK+VRP +SSI+V L+DR +D DL L+N S +
Sbjct 154
PFVWTLCTDVQEESRIPSLQSLKTVRPSESSIEVALIDRNSDPDLRQLQNIVLGLSRSCA
213

Query 234
DMKDVINQLAKLVSSRMGGTTSNEENLLPRWKESSEAIKSSAGSIVLHLGKLPIGLCKHR
293
D++ QLAKLV S++GG+ +NE+ LLPRWK+ SE +K+ +GS+V+ +GKL
+GLC+HR
Sbjct 214
TTNDMVEQLAKLVCSQLGGSATNEDFLLPRWKDRSEILKAISGSVVISMGKLSVGLCRHR
273

Query 294
SLLFKMLADKVNIPCRLVKGCKYCKAEDASSCVVRFGLEREYLVDLFGAPGQLSDPDSFV
353
SL+FKMLAD +N+PCR+ KGCKYCK+EDASSC+VRFG EREYLVDL PG L
+PDS +
Sbjct 274
SLMFKMLADAMNLPCRIAKGCKYCKSEDASSCLVRFGSEREYLVDLIENPGFLCEPDSLL
333

Query 354
NGPYSLSVPSPLRPPKFRSLEITSNFSSVAKQYFSDCHSLNLLFSDASTGASNGAAVAVD
413
NGPYS+S+ SPLRPPKF+S+E +F ++AKQYF DC SLN++F+DAS GA + +
Sbjct 334
NGPYSISISSPLRPPKFKSVESKGDFRALAKQYFLDCQSLNIMFNDASAGAGQKDTICLP
393

Query 414
QMYSKKHDAGDGIANSWVPVKGQAIANSDIILPEAPREVLPLMSPSNLTADKKKEFQLIE
473
SK +D VP+ A +II +A R + +++ + DK + + IE
Sbjct 394
S--SKHYD------EKAVPIVKDA-GQPNIIAQDARRGIASILNSFDPKVDKNNDLRFIE 444

Query 474
GNQYLRSTVSDLSLAVDDLIIPWSELVLKEKIGAGSFGTVHRADWHGSDVAVKILMEQDY
533
+Q S ++LAVDDL
IPWSELVLKE+IGAGSFGTVHRADW+GSDVAVKILMEQD+
Sbjct 445
EHQGRYRPTSTITLAVDDLAIPWSELVLKERIGAGSFGTVHRADWNGSDVAVKILMEQDF
504

Query 534
HLDRFKEFMREVAIMKSLRHPNIVLFMGAVTEPPNLSIVTEYLSRGSLYKLLHRSGAREV
593
H +RFKEF+REVAIMKSLRHPNIVL+MGAVT+PPNLSIVTEYLSRGSLY+LLHR
GARE+
Sbjct 505
HQERFKEFLREVAIMKSLRHPNIVLYMGAVTQPPNLSIVTEYLSRGSLYRLLHRHGAREI
564

Query 594
LDERRRLNMAFDVAKGMNYLHRRSPPIVHRDLKSPNLLVDKKYTVKVCDFGLSRLKANT
Y 653

LDERRRL+MAFDVAKGMNYLH+R+PPIVHRDLKSPNLLVDKKYTVKVCDFGLSRLKANT
+
Sbjct 565
LDERRRLSMAFDVAKGMNYLHKRNPPIVHRDLKSPNLLVDKKYTVKVCDFGLSRLKANT
F 624

Query 654

LSSKSLAGTPEWMAPEVLRDEPSNEKSDVYSFAVILWELMTLQQPWCNLNPAQVVAAVGF 713
LSSKSLAGTPEWMAPEVLRDEPSNEKSDVYSF VILWELMTLQQPW LNPAQVVAAVGF
Sbjct 625 LSSKSLAGTPEWMAPEVLRDEPSNEKSDVYSFGVILWELMTLQQPWTTLNPAQVVAAVGF 684

Query 714 KGRRLEIPKELNPQVAALIESCWANEPWRRPSFANIMETLRPLINKVPVPQLIRSD 769
KGRRLEIP ++NPQVAA+IESCWANE W+RPSF NIME L+P+I K P PQ RSD
Sbjct 685 KGRRLEIPSDVNPQVAAIIESCWANESWKRPSFFNIMELLKPMI-KPPAPQPTRSD 739

>PREDICTED: serine/threonine-protein kinase CTR1-like [Musa acuminata subsp. malaccensis]
Sequence ID: XP_009383670.1 Length: 799
>unnamed protein product [Musa acuminata subsp. malaccensis]
Sequence ID: CAG1864442.1 Length: 799
Range 1: 4 to 780

Score:929 bits(2402), Expect:0.0,
Method:Compositional matrix adjust.,
Identities:503/797(63%), Positives:585/797(73%), Gaps:70/797(8%)

Query 8 GGRRTSYSLLSQFPDDA-------AAAGASP---------AVLQRQSSGGSS-------- 43
GRR++YSLLSQ PDD+ A A ASP A LQRQSSG S
Sbjct 4 AGRRSNYSLLSQSPDDSSPPPTDKARARASPFDWSIAPAVAPLQRQSSGSSYGESSLSGG 63

Query 44 --YGAGSSVSASSDY-PFHLPPAVAAAGGGGGTPS-----------PCKSWAQQAEETYQ 89
Y + SA+ D F A +AGGG G + KSWAQQAEETYQ
Sbjct 64 DFYVPATISSATVDADAFSRMAARTSAGGGEGRTTDGAVAEASSLLSAKSWAQQAEETYQ 123

Query 90 LQLALALRLCADAACAADPGFLDPGDSGGSKMGGGGGGSGSGRAFPLAPPSPTAEALSHR 149

LQLALALRLC++AACA DP FLD D + P + ++SHR
Sbjct 124 LQLALALRLCSEAACADDPNFLDALDQT-----------------VLPDRVSPTSISHR
165

Query 150
FWVNGSLSYSNTIPDGFYLIQGMDPFVWSMCTDVHEENRIPSVESLKSVRPDDSSIQVVL
209
FWVNG LSY + IPDGFYLIQGMDPFVW++CTDV EENRIPSVESLK+V P
DSSI+V L
Sbjct 166
FWVNGCLSYHDKIPDGFYLIQGMDPFVWTLCTDVEEENRIPSVESLKTVHPCDSSIEVAL
225

Query 210
VDRRADFDLGMLENYASSFLSSSSDMKDVINQLAKLVSSRMGGTTSNEENLLPRWKESSE
269
VDR+ D DL L+N A+ S + KD+++QLA LV S MGGT NEE+LL RWKE
SE
Sbjct 226
VDRQYDPDLRQLQNVAAGLSCSCATPKDMVDQLANLVCSHMGGTAFNEEDLLHRWKEC
SE 285

Query 270
AIKSSAGSIVLHLGKLPIGLCKHRSLLFKMLADKVNIPCRLVKGCKYCKAEDASSCVVRF
329
A+K+++GS+VL +GKL +GLC+HR+LLFKMLAD +N+PCR+ KGCKYCK
DASSC+VRF
Sbjct 286
ALKATSGSVVLPIGKLSVGLCRHRALLFKMLADTINLPCRVAKGCKYCKTGDASSCLVRF
345

Query 330
GLEREYLVDLFGAPGQLSDPDSFVNGPYSLSVPSPLRPPKFRSLEITSNFSSVAKQYFSD
389
GLEREYLVDL PG L +PDS +NGPYS+S+ SPLRPPK +S E+T NF ++AKQY
D
Sbjct 346
GLEREYLVDLIRNPGNLCEPDSLLNGPYSISISSPLRPPKAKSTEVTVNFRTLAKQYLLD
405

Query 390
CHSLNLLFSDASTGASNGAAVAVDQMYSKKHDAGDGIANSWVPVKGQAIANSDIILPEAP
449
C SLNL F+DAS GA VD S+ D S P++ ++ LP

Sbjct 406
CQSLNLFFNDASAGAVVAQGDVVDLSSSRPLDEKSVEVIS-SPLEATGAELCELPLPHIQ
464

Query 450
REVLPLMS-----------PSNLTADKKKEFQLI-EGNQYLRSTVSDLSLAVDDLIIPWS 497
+ P S P + ADKK +F+LI + Q +++SLA+DDL IPWS
Sbjct 465
KVARPEPSKAGHKDVLHIIPPDPKADKK-DFRLIKDSKQGHNRPNNEISLAIDDLNIPWS
523

Query 498
ELVLKEKIGAGSFGTVHRADWHGSDVAVKILMEQDYHLDRFKEFMREVAIMKSLRHPNIV
557
ELVLKE+IGAGSFGTVHRA+WHGSDVAVKILMEQD H +R
KEF+REVAIMKSLRHPNIV
Sbjct 524
ELVLKERIGAGSFGTVHRAEWHGSDVAVKILMEQDLHPERLKEFLREVAIMKSLRHPNIV
583

Query 558
LFMGAVTEPPNLSIVTEYLSRGSLYKLLHRSGAREVLDERRRLNMAFDVAKGMNYLHRRS
617
LFMGAVTEP
NLSIVTEYLSRGSLY+LLHR+GAREVLDERRRL+MAFDVAKGMNYLH+R+
Sbjct 584
LFMGAVTEPRNLSIVTEYLSRGSLYRLLHRNGAREVLDERRRLSMAFDVAKGMNYLHKR
N 643

Query 618
PPIVHRDLKSPNLLVDKKYTVKVCDFGLSRLKANTYLSSKSLAGTPEWMAPEVLRDEPSN
677

PPIVHRDLKSPNLLVDKKYTVKVCDFGLSRLKANT+LSSKSLAGTPEWMAPEVLRDEPSN
Sbjct 644
PPIVHRDLKSPNLLVDKKYTVKVCDFGLSRLKANTFLSSKSLAGTPEWMAPEVLRDEPSN
703

Query 678
EKSDVYSFAVILWELMTLQQPWCNLNPAQVVAAVGFKGRRLEIPKELNPQVAALIESCWA
737
EKSDVYSF VILWELMTLQQPW NLNPAQVVAAVGFKGR LEIP
++NPQVAA+IESCWA
Sbjct 704

EKSDVYSFGVILWELMTLQQPWSNLNPAQVVAAVGFKGRWLEIPSDVNPQVAAIIESCWA
763

Query 738 NEPWRRPSFANIMETLR 754
NEPW+RP+F++IM++L+
Sbjct 764 NEPWKRPAFSSIMDSLK 780

>serine threonine-protein kinase [Musa troglodytarum]
Sequence ID: URD83379.1 Length: 803
Range 1: 4 to 784

Score:921 bits(2381), Expect:0.0,
Method:Compositional matrix adjust.,
Identities:496/802(62%), Positives:582/802(72%), Gaps:76/802(9%)

Query 8 GGRRTSYSLLSQFPDDAAAA--------------------GASPAV--LQRQSSGGSS-- 43
GRR++YSLLSQ P+D+ +PAV LQRQSSG S
Sbjct 4
AGRRSTYSLLSQSPNDSPPPPKFESPPNDKARPRASHFDWSIAPAVAPLQRQSSGSSYGE
63

Query 44 --------YGAGSSVSASSDY-PFHLPPAVAAAGGGGG---------TPSPCKSWAQQAE
85
Y + SA+ D F A+ +AGGG G S KSWAQQAE
Sbjct 64
SSLSGGDFYVPATISSATVDADSFSRMAALTSAGGGEGRTKDGAVVEASSSAKSWAQQAE
123

Query 86
ETYQLQLALALRLCADAACAADPGFLDPGDSGGSKMGGGGGGSGSGRAFPLAPPSPTAE
A 145
ETYQLQLALALRLC++AACA DP FLD D + P +
Sbjct 124 ETYQLQLALALRLCSEAACADDPNFLDALDQT------------------VLPERVAPTS
165

Query 146
LSHRFWVNGSLSYSNTIPDGFYLIQGMDPFVWSMCTDVHEENRIPSVESLKSVRPDDSSI
205
+SHRFWVNG LSY + IPDGFYLIQGMDPFVW++CTDV EENRIPS+ESLK+V P
DSSI
Sbjct 166

ISHRFWVNGCLSYHDKIPDGFYLIQGMDPFVWTLCTDVEEENRIPSIESLKTVHPCDSSI 225

Query 206 QVVLVDRRADFDLGMLENYASSFLSSSSDMKDVINQLAKLVSSRMGGTTSNEENLLPRWK 265
+V LVDR+ D +L L+N + S KD++ QLA LV S MGGT NE++LL WK
Sbjct 226 EVALVDRQYDPNLRQLQNMVAGLSCSCVTPKDMVVQLANLVCSHMGGTAFNEDDLLRDWK 285

Query 266 ESSEAIKSSAGSIVLHLGKLPIGLCKHRSLLFKMLADKVNIPCRLVKGCKYCKAEDASSC 325
E SEA+K+++GS+VL +GKL +GLC+HR+LLFKMLAD +N+PCR+ KGCKYCK DASSC
Sbjct 286 ECSEALKATSGSVVLPIGKLSVGLCRHRALLFKMLADTINLPCRVAKGCKYCKTGDASSC 345

Query 326 VVRFGLEREYLVDLFGAPGQLSDPDSFVNGPYSLSVPSPLRPPKFRSLEITSNFSSVAKQ 385
+VRFGLEREYLVDL PG L +PDS +NGPYS+S+ SPLRPP +S E+T NF ++AKQ
Sbjct 346 LVRFGLEREYLVDLIRNPGNLCEPDSLLNGPYSISISSPLRPPTAKSTEVTVNFRTLAKQ 405

Query 386 YFSDCHSLNLLFSDASTGASNGAAVAVDQMYSKKHDAGDGIANSWVPVKGQAIANSDIIL 445
Y DC SLNL F+DASTGA VD S+ D S P++ ++ L
Sbjct 406 YLLDCQSLNLFFNDASTGAVVAQGDVVDLSSSRPLDEKSVEVIS-SPLEATRTELCELPL 464

Query 446 PE------------APREVLPLMSPSNLTADKKKEFQLI-EGNQYLRSTVSDLSLAVDDL 492
P ++VL ++ P + ADK +F+LI + Q +++SLA+DDL
Sbjct 465 PHIQEVSRPEPSKAVHKDVLHIIPP-DPKADKN-DFRLIKDSKQSHNRPNNEISLAIDDL 522

Query 493 IIPWSELVLKEKIGAGSFGTVHRADWHGSDVAVKILMEQDYHLDRFKEFMREVAIMKSLR 552
IPWSELVLKE+IGAGSFGTVHRA+WHGSDVAVKILMEQD H +R KEF+REVAIMKSLR
Sbjct 523 NIPWSELVLKERIGAGSFGTVHRAEWHGSDVAVKILMEQDLHPERLKEFLREVAIMKSLR 582

Query 553 HPNIVLFMGAVTEPPNLSIVTEYLSRGSLYKLLHRSGAREVLDERRRLNMAFDVAKGMNY 612
HPNIVLFMGAVTEP NLSIVTEYLSRGSLY+LLHR+GAREVLDERRRL+MAFDVAKGMNY
Sbjct 583 HPNIVLFMGAVTEPRNLSIVTEYLSRGSLYRLLHRNGAREVLDERRRLSMAFDVAKGMNY 642

Query 613 LHRRSPPIVHRDLKSPNLLVDKKYTVKVCDFGLSRLKANTYLSSKSLAGTPEWMAPEVLR 672
LH+R+PPIVHRDLKSPNLLVDKKYTVKVCDFGLSRLKANT+LSSKSLAGTPEWMAPEVLR
Sbjct 643 LHKRNPPIVHRDLKSPNLLVDKKYTVKVCDFGLSRLKANTFLSSKSLAGTPEWMAPEVLR 702

Query 673 DEPSNEKSDVYSFAVILWELMTLQQPWCNLNPAQVVAAVGFKGRRLEIPKELNPQVAALI 732
DEPSNEKSDVYSF VILWELMTLQQPW NLNPAQVVAAVGFKGRRLEIP ++NPQVAA+I
Sbjct 703 DEPSNEKSDVYSFGVILWELMTLQQPWSNLNPAQVVAAVGFKGRRLEIPSDVNPQVAAII 762

Query 733 ESCWANEPWRRPSFANIMETLR 754
ESCWANEPW+RP+F++IME+L+
Sbjct 763 ESCWANEPWKRPAFSSIMESLK 784

>hypothetical protein OPV22_000954 [Ensete ventricosum]

Sequence ID: KAJ8510520.1 Length: 804
Range 1: 4 to 785

Score:919 bits(2374), Expect:0.0,
Method:Compositional matrix adjust.,
Identities:498/802(62%), Positives:581/802(72%), Gaps:75/802(9%)

Query 8 GGRRTSYSLLSQFP-------------DDAAAAGASP---------AVLQRQSSGGSS-- 43
GRR++YSLLSQ P D A A ASP A LQRQSSG S
Sbjct 4
AGRRSTYSLLSQSPDDSPPPPKFESPPTDKARARASPFDWSIAPAVAPLQRQSSGSSYGE
63

Query 44 --------YGAGSSVSASSDY-PFHLPPAVAAAGGGGGTP-----------SPCKSWAQQ
83
Y + SA+ D F A+ +AGGG G S KSWAQQ
Sbjct 64
SSLSGGDFYVPATISSATVDADAFSRMAALTSAGGGEGRTKDGAVVEASLSSSAKSWAQQ
123

Query 84
AEETYQLQLALALRLCADAACAADPGFLDPGDSGGSKMGGGGGGSGSGRAFPLAPPSPT
A 143
EETYQLQLALALRLC++AACA DP FLD D + P
Sbjct 124 TEETYQLQLALALRLCSEAACADDPNFLDALDQT------------------VLPERVAP
165

Query 144
EALSHRFWVNGSLSYSNTIPDGFYLIQGMDPFVWSMCTDVHEENRIPSVESLKSVRPDDS
203
++SHRFWVNG LSY + I DGFYLIQGMDPFVW++CTDV EENRIPS+ESLK+V
P DS
Sbjct 166
ASISHRFWVNGCLSYHDKILDGFYLIQGMDPFVWTLCTDVEEENRIPSIESLKTVHPCDS
225

Query 204
SIQVVLVDRRADFDLGMLENYASSFLSSSSDMKDVINQLAKLVSSRMGGTTSNEENLLPR
263
SI+V LVDR+ D DL L+N + S + KD+++QLA LV SRMGG NEE+LL R
Sbjct 226
SIEVALVDRQYDPDLRQLQNVVAGLSCSCATPKDMVDQLANLVCSRMGGIAFNEEDLLRR
285

Query 264
WKESSEAIKSSAGSIVLHLGKLPIGLCKHRSLLFKMLADKVNIPCRLVKGCKYCKAEDAS
323
WKE SEA+K+++GS+VL +GKL +GLC+HR+LLFKMLAD +N+PCR+
KGCKYCK DAS
Sbjct 286
WKECSEALKATSGSVVLPIGKLSVGLCRHRALLFKMLADTINLPCRIAKGCKYCKTGDAS
345

Query 324
SCVVRFGLEREYLVDLFGAPGQLSDPDSFVNGPYSLSVPSPLRPPKFRSLEITSNFSSVA
383
SC+VRFGLEREYLVDL PG L +PDS +NGPYS+S+ SPLRPPK +S E+T NF
++A
Sbjct 346
SCLVRFGLEREYLVDLIRNPGNLCEPDSLLNGPYSISISSPLRPPKAKSTEVTVNFRTLA
405

Query 384
KQYFSDCHSLNLLFSDASTGASNGAAVAVDQMYSKKHDAGDGIANSWVPVKGQAIAN---
440
KQY DC SLNL F+DAS GA VD ++ + +S + G +
Sbjct 406
KQYLLDCQSLNLFFNDASAGAVVAQGDVVDFSSRPFNEKSVEVISSPLAAAGTELCELPL
465

Query 441
---SDIILPE----APREVLPLMSPSNLTADKKKEFQLI-EGNQYLRSTVSDLSLAVDDL 492
+ PE R+VL ++ P + ADKK +F+L + Q +++S AVDDL
Sbjct 466
PNIQKVARPEPSKAVHRDVLHIIPP-DPKADKK-DFRLTKDSKQGHNRPNNEISFAVDDL
523

Query 493
IIPWSELVLKEKIGAGSFGTVHRADWHGSDVAVKILMEQDYHLDRFKEFMREVAIMKSLR
552
IPWSELVLKE+IGAGSFGTVHRA+WHGSDVAVKILMEQD H +R
KEF+REVAIMKSLR
Sbjct 524
NIPWSELVLKERIGAGSFGTVHRAEWHGSDVAVKILMEQDLHPERLKEFLREVAIMKSLR
583

Query 553
HPNIVLFMGAVTEPPNLSIVTEYLSRGSLYKLLHRSGAREVLDERRRLNMAFDVAKGMNY

612
HPNIVLFMGAVTEP
NLSIVTEYLSRGSLY+LLHR+GAREVLDERRRL+MAFDVAKGMNY
Sbjct 584
HPNIVLFMGAVTEPRNLSIVTEYLSRGSLYRLLHRNGAREVLDERRRLSMAFDVAKGMNY
643

Query 613
LHRRSPPIVHRDLKSPNLLVDKKYTVKVCDFGLSRLKANTYLSSKSLAGTPEWMAPEVLR
672

LHRR+PPIVHRDLKSPNLLVDKKYTVKVCDFGLSRLKANT+LSSKSLAGTPEWMAPEVLR
Sbjct 644
LHRRNPPIVHRDLKSPNLLVDKKYTVKVCDFGLSRLKANTFLSSKSLAGTPEWMAPEVLR
703

Query 673
DEPSNEKSDVYSFAVILWELMTLQQPWCNLNPAQVVAAVGFKGRRLEIPKELNPQVAALI
732
DEPSNEKSD+YSF VILWELMTLQQPW NLNPAQVVAAVGFKGRRLEIP
++NPQVAA+I
Sbjct 704
DEPSNEKSDIYSFGVILWELMTLQQPWSNLNPAQVVAAVGFKGRRLEIPSDVNPQVAAII
763

Query 733 ESCWANEPWRRPSFANIMETLR 754
ESCWANEPW+RP F++IM++L+
Sbjct 764 ESCWANEPWKRPPFSSIMDSLK 785

>unnamed protein product [Musa acuminata subsp. malaccensis]
Sequence ID: CAG1864453.1 Length: 805
Range 1: 4 to 786

Score:916 bits(2368), Expect:0.0,
Method:Compositional matrix adjust.,
Identities:501/803(62%), Positives:581/803(72%), Gaps:76/803(9%)

Query 8 GGRRTSYSLLSQFP-------------DDAAAAGASP---------AVLQRQSSGGSS-- 43
GRR++YSLLSQ P D A A ASP A LQRQSSG S
Sbjct 4
AGRRSTYSLLSQSPDDSPPPPKFDSPPTDKARARASPFDWSIAPAVAPLQRQSSGSSYGE

63

Query 44 --------YGAGSSVSASSDY-PFHLPPAVAAAGGGGGTPS-----------PCKSWAQQ
83
Y + SA+ D F A+ +AGGG G KSWAQQ
Sbjct 64
SSLSGGDFYAPATISSATVDADAFSRMTALTSAGGGEGRTKDGAVAEASSSSSAKSWAQQ
123

Query 84
AEETYQLQLALALRLCADAACAADPGFLDPGDSGGSKMGGGGGGSGSGRAFPLAPPSPT
A 143
AEETYQLQLALALRLC++AACA DP FLD D + P +
Sbjct 124 AEETYQLQLALALRLCSEAACADDPNFLDALDQT------------------VLPERVSP
165

Query 144
EALSHRFWVNGSLSYSNTIPDGFYLIQGMDPFVWSMCTDVHEENRIPSVESLKSVRPDDS
203
+SHRFWVNG LSY + IPDGFYLIQGMDPFVW++C DV EENRIPSVESLK+V
P DS
Sbjct 166
TTISHRFWVNGCLSYHDKIPDGFYLIQGMDPFVWTLCADVEEENRIPSVESLKTVHPCDS
225

Query 204
SIQVVLVDRRADFDLGMLENYASSFLSSSSDMKDVINQLAKLVSSRMGGTTSNEENLLPR
263
SI+V LVDR+ D DL L+N + S + KD+++QLA LV S MGGT NEE+LL R
Sbjct 226
SIEVALVDRQYDPDLRQLQNVVAGLSCSCATPKDMVDQLASLVCSHMGGTAFNEEDLLRR
285

Query 264
WKESSEAIKSSAGSIVLHLGKLPIGLCKHRSLLFKMLADKVNIPCRLVKGCKYCKAEDAS
323
WKE SEA+K+++GS+VL +GKL +GLC+HR+LLFKMLAD +N+PCR+
KGCKYCK DAS
Sbjct 286
WKECSEALKATSGSVVLPIGKLSVGLCRHRALLFKMLADTINLPCRVAKGCKYCKTGDAS
345

Query 324
SCVVRFGLEREYLVDLFGAPGQLSDPDSFVNGPYSLSVPSPLRPPKFRSLEITSNFSSVA

383

SC+VRFGLEREYLVDL PG L +PDS +NGPYS+S+ SPLRPPK +S E+T NF ++A

Sbjct 346

SCLVRFGLEREYLVDLIRNPGNLCEPDSLLNGPYSISISSPLRPPKAKSTEVTVNFRTLA

405

Query 384

KQYFSDCHSLNLLFSDASTGASNGAAVAVDQMYSKKHDAGDGIANSWVPVKGQAIANSD I 443

KQY DC SLNL F+DAS GA VD S+ D S P++ ++

Sbjct 406

KQYLLDCQSLNLFFNDASAGAVVAQGDVVDLSSSRPLDEKSVEVIS-SPLEATGTELCEL

464

Query 444

ILPEAPREVLPLMS-----------PSNLTADKKKEFQLI-EGNQYLRSTVSDLSLAVDD 491

LP + P+ S P + ADKK +F+LI + Q +++SLA+DD

Sbjct 465

PLPHIQKVARPVPSKAVQKDVLHIIPPDPKADKK-DFRLIKDSKQGHNRPNNEISLAIDD

523

Query 492

LIIPWSELVLKEKIGAGSFGTVHRADWHGSDVAVKILMEQDYHLDRFKEFMREVAIMKSL

551

L IPWSELVLKE+IGAGSFGTVHRA+WHGSDVAVKILMEQD H +R KEF+REVAIMKSL

Sbjct 524

LNIPWSELVLKERIGAGSFGTVHRAEWHGSDVAVKILMEQDLHPERLKEFLREVAIMKSL

583

Query 552

RHPNIVLFMGAVTEPPNLSIVTEYLSRGSLYKLLHRSGAREVLDERRRLNMAFDVAKGMN

611

RHPNIVLFMGAVTEP NLSIVTEYLSRGSLY+LLHR+GAREVLDERRRL+MAFDVAKGMN

Sbjct 584

RHPNIVLFMGAVTEPRNLSIVTEYLSRGSLYRLLHRNGAREVLDERRRLSMAFDVAKGMN

643

Query 612

YLHRRSPPIVHRDLKSPNLLVDKKYTVKVCDFGLSRLKANTYLSSKSLAGTPEWMAPEVL

671

YLH+R+PPIVHRDLKSPNLLVDKKYTVKVCDFGLSRLKANT+LSSKSLAGTPEWMAPEVL
Sbjct 644
YLHKRNPPIVHRDLKSPNLLVDKKYTVKVCDFGLSRLKANTFLSSKSLAGTPEWMAPEVL
703

Query 672
RDEPSNEKSDVYSFAVILWELMTLQQPWCNLNPAQVVAAVGFKGRRLEIPKELNPQVAAL
731
RDEPSNEKSDVYSF VILWE MTLQQPW NLNPAQVVAAVGFKGRRLEIP
++NPQVAA+
Sbjct 704
RDEPSNEKSDVYSFGVILWEFMTLQQPWSNLNPAQVVAAVGFKGRRLEIPSDVNPQVAAI
763

Query 732 IESCWANEPWRRPSFANIMETLR 754
IESCWANEPW+RP+F++IME+L+
Sbjct 764 IESCWANEPWKRPAFSSIMESLK 786

>constitutive triple response 1-like protein [Musa acuminata AAA Group]
Sequence ID: AFA37962.1 Length: 805
Range 1: 4 to 786

Score:914 bits(2363), Expect:0.0,
Method:Compositional matrix adjust.,
Identities:500/803(62%), Positives:581/803(72%), Gaps:76/803(9%)

Query 8 GGRRTSYSLLSQFP-------------DDAAAAGASP---------AVLQRQSSGGSS-- 43
GRR++YSLLSQ P D A A ASP A LQRQSSG S
Sbjct 4
AGRRSTYSLLSQSPDDSPPPPKFDSPPTDKARARASPFDWSIAPAVAPLQRQSSGSSYGE
63

Query 44 --------YGAGSSVSASSDY-PFHLPPAVAAAGGGGGTPS-----------PCKSWAQQ
83
Y + SA+ D F A+ +AGGG G KSWAQQ
Sbjct 64
SSLSGGDFYAPATISSATVDADAFSRMTALTSAGGGEGRTKDGAVAEASSSSSAKSWAQQ
123

Query 84
AEETYQLQLALALRLCADAACAADPGFLDPGDSGGSKMGGGGGGSGSGRAFPLAPPSPT

A 143
AEETYQLQLALALRLC++AACA DP FLD D + P +
Sbjct 124 AEETYQLQLALALRLCSEAACADDPNFLDALDQT------------------VLPERVSP
165

Query 144
EALSHRFWVNGSLSYSNTIPDGFYLIQGMDPFVWSMCTDVHEENRIPSVESLKSVRPDDS
203
+SHRFWVNG LSY + IPDGFYLIQGMDPFVW++C DV EENRIPSVESLK+V
P DS
Sbjct 166
TTISHRFWVNGCLSYHDKIPDGFYLIQGMDPFVWTLCADVEEENRIPSVESLKTVHPCDS
225

Query 204
SIQVVLVDRRADFDLGMLENYASSFLSSSSDMKDVINQLAKLVSSRMGGTTSNEENLLPR
263
SI+V LVDR+ D DL L+N + S + KD+++QLA LV S MGGT NEE+LL R
Sbjct 226
SIEVALVDRQYDPDLRQLQNVVAGLSCSCATPKDMVDQLASLVCSHMGGTAFNEEDLLRR
285

Query 264
WKESSEAIKSSAGSIVLHLGKLPIGLCKHRSLLFKMLADKVNIPCRLVKGCKYCKAEDAS
323
WKE SEA+K+++GS+VL +GKL +GLC+HR+LLFKMLAD +N+PCR+
KGCKYCK DAS
Sbjct 286
WKECSEALKATSGSVVLPIGKLSVGLCRHRALLFKMLADTINLPCRVAKGCKYCKTGDAS
345

Query 324
SCVVRFGLEREYLVDLFGAPGQLSDPDSFVNGPYSLSVPSPLRPPKFRSLEITSNFSSVA
383
SC+VRFGLEREYLVDL PG L +PDS +NGPYS+S+ SPLRPPK +S E+T NF
++A
Sbjct 346
SCLVRFGLEREYLVDLIRNPGNLCEPDSLLNGPYSISISSPLRPPKAKSTEVTVNFRTLA
405

Query 384
KQYFSDCHSLNLLFSDASTGASNGAAVAVDQMYSKKHDAGDGIANSWVPVKGQAIANSD
I 443
KQY DC SLNL F+DAS GA VD S+ D S P++ ++

Sbjct 406
KQYLLDCQSLNLFFNDASAGAVVAQGDVVDLSSSRPLDEKSVEVIS-SPLEATGTELCEL
464

Query 444
ILPEAPREVLPLMS-----------PSNLTADKKKEFQLI-EGNQYLRSTVSDLSLAVDD 491
LP + P+ S P + ADKK +F+LI + Q +++SLA+DD
Sbjct 465
PLPHIQKVARPVPSKAVQKDVLHIIPPDPKADKK-DFRLIKDSKQGHNRPNNEISLAIDD
523

Query 492
LIIPWSELVLKEKIGAGSFGTVHRADWHGSDVAVKILMEQDYHLDRFKEFMREVAIMKSL
551
L IPWSELVLKE+IGAGSFGTVHRA+WHGSDVAVKILMEQD H +R
KEF+REVAIMKSL
Sbjct 524
LNIPWSELVLKERIGAGSFGTVHRAEWHGSDVAVKILMEQDLHPERLKEFLREVAIMKSL
583

Query 552
RHPNIVLFMGAVTEPPNLSIVTEYLSRGSLYKLLHRSGAREVLDERRRLNMAFDVAKGMN
611
RHPNIVLFMGAVTEP
NLSIVTEYLSRGSLY+LLHR+GAREVLDERRRL+MAFDVAKGMN
Sbjct 584
RHPNIVLFMGAVTEPRNLSIVTEYLSRGSLYRLLHRNGAREVLDERRRLSMAFDVAKGMN
643

Query 612
YLHRRSPPIVHRDLKSPNLLVDKKYTVKVCDFGLSRLKANTYLSSKSLAGTPEWMAPEVL
671

YLH+R+PPIVHRDLKSPNLLVDKKYTVKVCDFGLSRLKANT+LSSKSLAGTPEWMAPEVL
Sbjct 644
YLHKRNPPIVHRDLKSPNLLVDKKYTVKVCDFGLSRLKANTFLSSKSLAGTPEWMAPEVL
703

Query 672
RDEPSNEKSDVYSFAVILWELMTLQQPWCNLNPAQVVAAVGFKGRRLEIPKELNPQVAAL
731
RDEPSNEKSDVYSF VILWE MTLQQPW NLNPAQVVAAVGFKGRRLEIP
++NPQVAA+
Sbjct 704

RDEPSNEKSDVYSFGVILWEFMTLQQPWSNLNPAQVVAAVGFKGRRLEIPSDVNPQVAAI 763

Query 732 IESCWANEPWRRPSFANIMETLR 754
IESCWANEPW+RP+F++IM++L+
Sbjct 764 IESCWANEPWKRPAFSSIMDSLK 786

>PREDICTED: serine/threonine-protein kinase CTR1-like [Musa acuminata subsp. malaccensis]
Sequence ID: XP_018676639.1 Length: 805
Range 1: 4 to 786

Score:914 bits(2363), Expect:0.0,
Method:Compositional matrix adjust.,
Identities:500/803(62%), Positives:580/803(72%), Gaps:76/803(9%)

Query 8 GGRRTSYSLLSQFP-------------DDAAAAGASP---------AVLQRQSSGGSS-- 43
GRR++YSLLSQ P D A A ASP A LQRQSSG S
Sbjct 4 AGRRSTYSLLSQSPDDSPPPPKFDSPPTDKARARASPFDWSIAPAVAPLQRQSSGSSYGE 63

Query 44 --------YGAGSSVSASSDY-PFHLPPAVAAAGGGGGTPS-----------PCKSWAQQ 83
Y + SA+ D F A+ +AGGG G KSWAQQ
Sbjct 64 SSLSGGDFYAPATISSATVDADAFSRMTALTSAGGGEGRTKDGAVAEASSSSSAKSWAQQ 123

Query 84 AEETYQLQLALALRLCADAACAADPGFLDPGDSGGSKMGGGGGGSGSGRAFPLAPPSPTA 143
AEETYQLQLALALRLC++AACA DP FLD D + P +
Sbjct 124 AEETYQLQLALALRLCSEAACADDPNFLDALDQT------------------VLPERVSP 165

Query 144 EALSHRFWVNGSLSYSNTIPDGFYLIQGMDPFVWSMCTDVHEENRIPSVESLKSVRPDDS 203
+SHRFWVNG LSY + IPDGFYLIQGMDPFVW++C DV EENRIPSVESLK+VP DS
Sbjct 166

TTISHRFWVNGCLSYHDKIPDGFYLIQGMDPFVWTLCADVEEENRIPSVESLKTVHPCDS
225

Query 204
SIQVVLVDRRADFDLGMLENYASSFLSSSSDMKDVINQLAKLVSSRMGGTTSNEENLLPR
263
SI+V LVDR+ D DL L+N + S + KD+++QLA LV S MGGT NEE+LL R
Sbjct 226
SIEVALVDRQYDPDLRQLQNVVAGLSCSCATPKDMVDQLASLVCSHMGGTAFNEEDLLRR
285

Query 264
WKESSEAIKSSAGSIVLHLGKLPIGLCKHRSLLFKMLADKVNIPCRLVKGCKYCKAEDAS
323
WKE SEA+K+++GS+VL +GKL +GLC+HR+LLFKMLAD +N+PCR+
KGCKYCK DAS
Sbjct 286
WKECSEALKATSGSVVLPIGKLSVGLCRHRALLFKMLADTINLPCRVAKGCKYCKTGDAS
345

Query 324
SCVVRFGLEREYLVDLFGAPGQLSDPDSFVNGPYSLSVPSPLRPPKFRSLEITSNFSSVA
383
SC+VRFGLEREYLVDL PG L +PDS +NGPYS+S+ SPLRPPK +S E+T NF
++A
Sbjct 346
SCLVRFGLEREYLVDLIRNPGNLCEPDSLLNGPYSISISSPLRPPKAKSTEVTVNFRTLA
405

Query 384
KQYFSDCHSLNLLFSDASTGASNGAAVAVDQMYSKKHDAGDGIANSWVPVKGQAIANSD
I 443
KQY DC SLNL F+DAS GA VD S+ D S P++ ++
Sbjct 406
KQYLLDCQSLNLFFNDASAGAVVAQGDVVDLSSSRPLDEKSVEVIS-SPLEATGTELCEL
464

Query 444
ILPEAPREVLPLMS-----------PSNLTADKKKEFQLI-EGNQYLRSTVSDLSLAVDD 491
LP + P+ S P + ADKK +F+LI + Q +++SLA+DD
Sbjct 465
PLPHIQKVARPVPSKAVQKDVLHIIPPDPKADKK-DFRLIKDSKQGHNRPNNEISLAIDD
523

Query 492
LIIPWSELVLKEKIGAGSFGTVHRADWHGSDVAVKILMEQDYHLDRFKEFMREVAIMKSL
551
L IPWSELVLKE+IGAGSFGTVHRA+WHGSDVAVKILMEQD H +R
KEF+REVAIMKSL
Sbjct 524
LNIPWSELVLKERIGAGSFGTVHRAEWHGSDVAVKILMEQDLHPERLKEFLREVAIMKSL
583

Query 552
RHPNIVLFMGAVTEPPNLSIVTEYLSRGSLYKLLHRSGAREVLDERRRLNMAFDVAKGMN
611
RHPNIVLFMGAVTEP
NLSIVTEYLSRGSLY+LLHR+GAREVLDERRRL+MAFDVAKGMN
Sbjct 584
RHPNIVLFMGAVTEPRNLSIVTEYLSRGSLYRLLHRNGAREVLDERRRLSMAFDVAKGMN
643

Query 612
YLHRRSPPIVHRDLKSPNLLVDKKYTVKVCDFGLSRLKANTYLSSKSLAGTPEWMAPEVL
671
YLH+R+PPIVHRDLKSPNLLVDKKY
VKVCDFGLSRLKANT+LSSKSLAGTPEWMAPEVL
Sbjct 644
YLHKRNPPIVHRDLKSPNLLVDKKYAVKVCDFGLSRLKANTFLSSKSLAGTPEWMAPEVL
703

Query 672
RDEPSNEKSDVYSFAVILWELMTLQQPWCNLNPAQVVAAVGFKGRRLEIPKELNPQVAAL
731
RDEPSNEKSDVYSF VILWE MTLQQPW NLNPAQVVAAVGFKGRRLEIP
++NPQVAA+
Sbjct 704
RDEPSNEKSDVYSFGVILWEFMTLQQPWSNLNPAQVVAAVGFKGRRLEIPSDVNPQVAAI
763

Query 732 IESCWANEPWRRPSFANIMETLR 754
IESCWANEPW+RP+F++IME+L+
Sbjct 764 IESCWANEPWKRPAFSSIMESLK 786

>serine/threonine-protein kinase CTR1-like [Zingiber officinale]

Sequence ID: XP_042457633.1 Length: 811
>serine/threonine-protein kinase CTR1-like [Zingiber officinale]
Sequence ID: XP_042457634.1 Length: 811
Range 1: 1 to 806

Score:911 bits(2354), Expect:0.0,
Method:Compositional matrix adjust.,
Identities:499/836(60%), Positives:592/836(70%), Gaps:97/836(11%)

Query 1 MELPAAGGGRRTSYSLLSQFPDDA----------------------AAAGASPAVLQRQ 37
ME+P GRR+SYSLL Q PD+A A++ A+P LQRQ
Sbjct 1 MEMP----GRRSSYSLLGQNPDEALPPLFDTPPSDKARAARFEWPINASSVAAPPPLQRQ 56

Query 38 SSGGS--------SYGAGSSVSASSDYPFHLPPAVAAAGGGGGTPSPC------KSWAQQ 83
SSG S Y +++S+S+ P G G + KSWAQQ
Sbjct 57 SSGSSYGGSSFSGDYYLPATISSSNVDSEGFNPLAGGEGRGQDGAAAGLSSSSAKSWAQQ 116

Query 84 AEETYQLQLALALRLCADAACAADPGFLDPGDSGGSKMGGGGGGSGSGRAFPLAPPSPTA 143
AEETYQLQLALALRLC++AACA DP FLD D + P
Sbjct 117 AEETYQLQLALALRLCSEAACAEDPNFLDAADQM------------------VLPERAAP 158

Query 144 EALSHRFWVNGSLSYSNTIPDGFYLIQGMDPFVWSMCTDVHEENRIPSVESLKSVRPDDS 203
+LSHRFWVNG LSY + IPDGFYLIQGMDPFVW++CTDV EENRIPS+ESLK+VP+DS
Sbjct 159 TSLSHRFWVNGCLSYHDKIPDGFYLIQGMDPFVWTLCTDVEEENRIPSIESLKTVHPNDS 218

Query 204 SIQVVLVDRRADFDLGMLENYASSFLSSSSDMKDVINQLAKLVSSRMGGTTSN-EENLLP 262
SI+VVL+DR+ D DL LE + S + KDV+ QLAKLV +RMGG N E+ LL
Sbjct 219 SIEVVLIDRQHDPDLRHLETIVTGLSCSCATAKDVVEQLAKLVCTRMGGIAFNGEDALLH

278

Query 263
RWKESSEAIKSSAGSIVLHLGKLPIGLCKHRSLLFKMLADKVNIPCRLVKGCKYCKAEDA
322
WKE E IK+S+ SIVL +GKL GLC+HR+LLFKMLAD +N+PCR+ KGCKYCK
+DA
Sbjct 279
CWKECGETIKASSCSIVLPMGKLSFGLCRHRALLFKMLADTINLPCRVAKGCKYCKTDDA
338

Query 323
SSCVVRFGLEREYLVDLFGAPGQLSDPDSFVNGPYSLSVPSPLRPPKFRSLEITSNFSSV
382
SSC+VRFGLEREYLVDL PG L +PDS +NGPYS+S+ SPLRPPK +S EI NF ++
Sbjct 339
SSCLVRFGLEREYLVDLIKNPGNLCEPDSLLNGPYSVSIASPLRPPKVKSSEINVNFRTL
398

Query 383
AKQYFSDCHSLNLLFSDASTGASNGAAVAVDQMYSKKHDAG--------------DG-IA 427
AKQYF DC SLNL+F DAS G S A+D SK D G DG I
Sbjct 399
AKQYFQDCQSLNLIFKDASAGTSIAEGDAIDPSSSKPSDGGTESLQPCIQKVTWPDGNIV
458

Query 428
NS---WVPVKGQAIANSDIILPEAPREVLPL----------MSPSNLTADKKKEFQLIEG 474
N+ + P++ N D++ RE+ P+ +P AD K +F+L E
Sbjct 459
NANKMFNPLRNAR--NPDLV----ERELGPMKLTAQDGCGNTAPCEPNADMK-DFKLTED
511

Query 475
NQYLRSTVS-DLSLAVDDLIIPWSELVLKEKIGAGSFGTVHRADWHGSDVAVKILMEQDY
533
++++ + S D+ LA DDL IPW
EL+LKE+IGAGSFGTVHRA+WHGS+VAVKILMEQD+
Sbjct 512
SKHIGNRPSNDIRLAEDDLNIPWRELILKERIGAGSFGTVHRAEWHGSEVAVKILMEQDF
571

Query 534
HLDRFKEFMREVAIMKSLRHPNIVLFMGAVTEPPNLSIVTEYLSRGSLYKLLHRSGAREV

593
H R
+EF+REVAIMKSLRHPNIVLFMGAVTEPPNLSIVTEYLSRGSLY+LLH++GA+E
Sbjct 572
HPGRLREFLREVAIMKSLRHPNIVLFMGAVTEPPNLSIVTEYLSRGSLYRLLHKNGAKET
631

Query 594
LDERRRLNMAFDVAKGMNYLHRRSPPIVHRDLKSPNLLVDKKYTVKVCDFGLSRLKANT
Y 653

++ERRRL+MAFDVAKGMNYLH+R+PPIVHRDLKSPNLLVDKKYTVKVCDFGLSRLKANT+
Sbjct 632
IEERRRLSMAFDVAKGMNYLHKRNPPIVHRDLKSPNLLVDKKYTVKVCDFGLSRLKANT
F 691

Query 654
LSSKSLAGTPEWMAPEVLRDEPSNEKSDVYSFAVILWELMTLQQPWCNLNPAQVVAAVGF
713
LSSKSLAGTPEWMAPEVLRDEPSNEKSD+YSF VILWELMTLQQPW
NLNPAQVVAAVGF
Sbjct 692
LSSKSLAGTPEWMAPEVLRDEPSNEKSDIYSFGVILWELMTLQQPWSNLNPAQVVAAVGF
751

Query 714
KGRRLEIPKELNPQVAALIESCWANEPWRRPSFANIMETLRPLINKVPVPQLIRSD 769
K RR EIP +N VAA+IESCWA+EPW+RPSF++IME+L+PLI K PQ + SD
Sbjct 752
KARRPEIPSTVNRHVAAIIESCWASEPWKRPSFSSIMESLKPLI-KTQSPQPLHSD 806

>serine/threonine-protein kinase CTR1-like [Zingiber officinale]
Sequence ID: XP_042462984.1 Length: 811
>serine/threonine-protein kinase CTR1-like [Zingiber officinale]
Sequence ID: XP_042462985.1 Length: 811
Range 1: 1 to 806

Score:909 bits(2350), Expect:0.0,
Method:Compositional matrix adjust.,
Identities:499/836(60%), Positives:591/836(70%), Gaps:97/836(11%)

Query 1 MELPAAGGGRRTSYSLLSQFPDDA----------------------AAAGASPAVLQRQ 37
ME+P GRR+SYSLL Q PD+A A++ A+P LQRQ
Sbjct 1 MEMP----GRRSSYSLLGQNPDEATPPLFDTPPSDKARAARFEWPINASSVAAPPPLQRQ 56

Query 38 SSGGS--------SYGAGSSVSASSDYPFHLPPAVAAAGGGGGTPSPC------KSWAQQ 83
SSG S Y +++S+S+ P G G + KSWAQQ
Sbjct 57 SSGSSYGGSSFSGDYYLPATISSSNVDSEGFNPLAGGEGRGQDGAAAGLSSSSAKSWAQQ 116

Query 84 AEETYQLQLALALRLCADAACAADPGFLDPGDSGGSKMGGGGGGSGSGRAFPLAPPSPTA 143
AEETYQLQLALALRLC++AACA DP FLD D + P
Sbjct 117 AEETYQLQLALALRLCSEAACAEDPNFLDAADQM------------------VLPERAAP 158

Query 144 EALSHRFWVNGSLSYSNTIPDGFYLIQGMDPFVWSMCTDVHEENRIPSVESLKSVRPDDS 203
+LSHRFWVNG LSY + IPDGFYLIQGMDPFVW++CTDV EENRIPS+ESLK+V P+DS
Sbjct 159 TSLSHRFWVNGCLSYHDKIPDGFYLIQGMDPFVWTLCTDVEEENRIPSIESLKTVHPNDS 218

Query 204 SIQVVLVDRRADFDLGMLENYASSFLSSSSDMKDVINQLAKLVSSRMGGTTSN-EENLLP 262
SI+VVL+DR+ D DL LE + S + KDV+ QLAKLV +RMGG N E+ LL
Sbjct 219 SIEVVLIDRQHDPDLRHLETIVTGLSCSCATAKDVVEQLAKLVCTRMGGIAFNGEDALLH 278

Query 263 RWKESSEAIKSSAGSIVLHLGKLPIGLCKHRSLLFKMLADKVNIPCRLVKGCKYCKAEDA 322
WKE E IK+S+ SIVL +GKL GLC+HR+LLFKMLAD +N+PCR+ KGCKYCK +DA
Sbjct 279 CWKECGETIKASSCSIVLPMGKLSFGLCRHRALLFKMLADTINLPCRVAKGCKYCKTDDA

338

Query 323
SSCVVRFGLEREYLVDLFGAPGQLSDPDSFVNGPYSLSVPSPLRPPKFRSLEITSNFSSV
382
SSC+VRFGLEREYLVDL PG L +PDS +NGPYS+S+ SPLRPPK +S EI NF
+
Sbjct 339
SSCLVRFGLEREYLVDLIKNPGNLCEPDSLLNGPYSVSIASPLRPPKVKSSEINVNFRIL
398

Query 383
AKQYFSDCHSLNLLFSDASTGASNGAAVAVDQMYSKKHDAG--------------DG-IA 427
AKQYF DC SLNL+F DAS G S A+D SK D G DG I
Sbjct 399
AKQYFQDCQSLNLIFKDASAGTSIAEGDAIDPSSSKPSDGGTESLQPCIQKVTWPDGNIV
458

Query 428
NS---WVPVKGQAIANSDIILPEAPREVLPL----------MSPSNLTADKKKEFQLIEG 474
N+ + P++ N D++ RE+ P+ +P AD K +F+L E
Sbjct 459
NANKMFNPLRNAR--NPDLV----ERELGPMKLTAQDGCGNTAPCEPNADMK-DFKLTED
511

Query 475
NQYLRSTVS-DLSLAVDDLIIPWSELVLKEKIGAGSFGTVHRADWHGSDVAVKILMEQDY
533
++++ + S D+ LA DDL IPW
EL+LKE+IGAGSFGTVHRA+WHGS+VAVKILMEQD+
Sbjct 512
SKHIGNRPSNDIRLAEDDLNIPWRELILKERIGAGSFGTVHRAEWHGSEVAVKILMEQDF
571

Query 534
HLDRFKEFMREVAIMKSLRHPNIVLFMGAVTEPPNLSIVTEYLSRGSLYKLLHRSGAREV
593
H R
+EF+REVAIMKSLRHPNIVLFMGAVTEPPNLSIVTEYLSRGSLY+LLH++GA+E
Sbjct 572
HPGRLREFLREVAIMKSLRHPNIVLFMGAVTEPPNLSIVTEYLSRGSLYRLLHKNGAKET
631

Query 594

LDERRRLNMAFDVAKGMNYLHRRSPPIVHRDLKSPNLLVDKKYTVKVCDFGLSRLKANTY 653

++ERRRL+MAFDVAKGMNYLH+R+PPIVHRDLKSPNLLVDKKYTVKVCDFGLSRLKANT+
Sbjct 632
IEERRRLSMAFDVAKGMNYLHKRNPPIVHRDLKSPNLLVDKKYTVKVCDFGLSRLKANTF 691

Query 654
LSSKSLAGTPEWMAPEVLRDEPSNEKSDVYSFAVILWELMTLQQPWCNLNPAQVVAAVGF 713
LSSKSLAGTPEWMAPEVLRDEPSNEKSD+YSF VILWELMTLQQPW NLNPAQVVAAVGF
Sbjct 692
LSSKSLAGTPEWMAPEVLRDEPSNEKSDIYSFGVILWELMTLQQPWSNLNPAQVVAAVGF 751

Query 714
KGRRLEIPKELNPQVAALIESCWANEPWRRPSFANIMETLRPLINKVPVPQLIRSD 769
K RR EIP +N VAA+IESCWA+EPW+RPSF++IME+L+PLI K PQ + SD
Sbjct 752
KARRPEIPSTVNRHVAAIIESCWASEPWKRPSFSSIMESLKPLI-KTQSPQPLHSD 806

>hypothetical protein OPV22_029832 [Ensete ventricosum]
Sequence ID: KAJ8467280.1 Length: 795
Range 1: 1 to 789

Score:900 bits(2327), Expect:0.0,
Method:Compositional matrix adjust.,
Identities:495/818(61%), Positives:586/818(71%), Gaps:89/818(10%)

Query 1 MELPAAGGGRRTSYSLLSQFPDDA------------AAAGASPAV--------------- 33
ME+P GRR+ YSLL Q DDA A SP+
Sbjct 1
MEMP----GRRSGYSLLGQSSDDAQLPKFESPPSDKARPRPSPSPFDWPMAPAVAAAVTH 56

Query 34 LQRQSSGGSS---------YGAGSSVSASSDYPFHLPPAVAAAGGGGGTPS---------
75
LQRQSSG S Y + SA+ D P +AAAGG G +
Sbjct 57

LQRQSSGSSYGGSSLSGDYYVPTTLASATVDSDAFNP--MAAAGGEGRSKDGAAAAEAAA
114

Query 76
------PCKSWAQQAEETYQLQLALALRLCADAACAADPGFLDPGDSGGSKMGGGGGGSG
129
KSWAQQAEETYQLQLALALRLC++AACA DP FLD D
Sbjct 115
VGSSSSSAKSWAQQAEETYQLQLALALRLCSEAACADDPNFLDAVDQM------------ 162

Query 130
SGRAFPLAPPSPTAEALSHRFWVNGSLSYSNTIPDGFYLIQGMDPFVWSMCTDVHEENRI
189
+ P T ++SHRFWVNG LSY + IPDGFYLIQGMDPFVW++C DV
EENRI
Sbjct 163
------VLPERATPASMSHRFWVNGCLSYHDKIPDGFYLIQGMDPFVWTLCADVGEENRI
216

Query 190
PSVESLKSVRPDDSSIQVVLVDRRADFDLGMLENYASSFLSSSSDMKDVINQLAKLVSSR
249
PS+ESLK+V P DSSI+V L+DR+ D DL L++ + + + K+++ QLAKLV +
Sbjct 217
PSIESLKTVHPSDSSIEVALIDRQDDPDLRQLQSLVAGISRTCTMPKEMVEQLAKLVCTC
276

Query 250
MGGTTSNEEN-LLPRWKESSEAIKSSAGSIVLHLGKLPIGLCKHRSLLFKMLADKVNIPC
308
MGGT NEE+ LL RWKE SEA+K+S+GS+VL +GKL GLC+HR+LLFKMLAD
+ +PC
Sbjct 277
MGGTAYNEEDGLLHRWKECSEALKASSGSVVLPIGKLSAGLCRHRALLFKMLADTIKLPC
336

Query 309
RLVKGCKYCKAEDASSCVVRFGLEREYLVDLFGAPGQLSDPDSFVNGPYSLSVPSPLRPP
368
R+ KGCKYCK++ SSC+V G EREYLVDL +PG L +P+S +NGPYS+S+
SPLRPP
Sbjct 337
RVAKGCKYCKSDSGSSCLVDLGQEREYLVDLIRSPGNLFEPNSLLNGPYSISISSPLRPP
396

Query 369
KFRSLEITSNFSSVAKQYFSDCHSLNLLFSDASTGASNGAAVAVDQMYSKKHDAGDGIAN
428
K RS +T +F ++AKQYF DC SLNL F+D S GA+ A D S+ D +
Sbjct 397
KIRSTTVTVDFRTLAKQYFLDCQSLNLFFNDPSAGAAVAQGDATDPPSSRPFDE-KSMEM
455

Query 429
SWVPVKG---QAIANSDIILPEAPREVLPLMSPSNLTADKKKEFQLIE-----GNQYLRS 480
S P++ Q +A + R+V ++ PS+L A+KKK+F+ IE GN+
Sbjct 456
SSSPLRALLEQDVAQLKLTSQAGCRKVPQIIPPSDLKAEKKKDFRFIEDSRKGGNR---- 511

Query 481
TVSDLSLAVDDLIIPWSELVLKEKIGAGSFGTVHRADWHGSDVAVKILMEQDYHLDRFKE
540
+ +D+SLAVDDL
IPWSEL+LKE+IGAGSFGTVHRA+WHGSDVAVKILMEQD+H +R KE
Sbjct 512
SNNDISLAVDDLSIPWSELILKERIGAGSFGTVHRAEWHGSDVAVKILMEQDFHPERLKE
571

Query 541
FMREVAIMKSLRHPNIVLFMGAVTEPPNLSIVTEYLSRGSLYKLLHRSGAREVLDERRRL
600
F REVAIMKSLRHPNIVLFMGAVTEPP
LSIVTEYLSRGSLY++LHR+GARE+LDERRRL
Sbjct 572
FRREVAIMKSLRHPNIVLFMGAVTEPPKLSIVTEYLSRGSLYRILHRNGAREILDERRRL
631

Query 601
NMAFDVAKGMNYLHRRSPPIVHRDLKSPNLLVDKKYTVKVCDFGLSRLKANTYLSSKSL
A 660
+MAFDVAKGMNYLH RS
PIVHRDLKSPNLLVDKKYTVKVCDFGLSRLKANT+LSSKSLA
Sbjct 632
SMAFDVAKGMNYLHNRSTPIVHRDLKSPNLLVDKKYTVKVCDFGLSRLKANTFLSSKSL
A 691

Query 661
GTPEWMAPEVLRDEPSNEKSDVYSFAVILWELMTLQQPWCNLNPAQVVAAVGFKGRRLEI

720
GTPEWMAPEVLRDEPSNEKSDVYSF VILWELMTLQQPW NLNPAQVVAAVGFKGRRLEI
Sbjct 692
GTPEWMAPEVLRDEPSNEKSDVYSFGVILWELMTLQQPWSNLNPAQVVAAVGFKGRRLE
I 751

Query 721 PKELNPQVAALIESCWANEPWRRPSFANIMETLRPLIN 758
P ++NP VAA+IESCWANEPW+RPSF++I E+L+PLI
Sbjct 752 PSDVNPHVAAMIESCWANEPWKRPSFSSITESLKPLIK 789

>serine/threonine-protein kinase CTR1 [Canna indica]
Sequence ID: WOL03471.1 Length: 797
Range 1: 120 to 784

Score:899 bits(2324), Expect:0.0,
Method:Compositional matrix adjust.,
Identities:455/691(66%), Positives:544/691(78%), Gaps:38/691(5%)

Query 82
QQAEETYQLQLALALRLCADAACAADPGFLDPGDSGGSKMGGGGGGSGSGRAFPLAPPS
P 141
QQAEETYQLQLALALRLC++AACA DP FLD D + P
Sbjct 120
QQAEETYQLQLALALRLCSEAACADDPNFLDAADEM------------------VQPERA 161

Query 142
TAEALSHRFWVNGSLSYSNTIPDGFYLIQGMDPFVWSMCTDVHEENRIPSVESLKSVRPD
201
++SHRFWVNG LSY + IPDGFYLIQGMDPFVW++CT+V
EENRIPS+ESLK+V P
Sbjct 162
APASMSHRFWVNGCLSYHDKIPDGFYLIQGMDPFVWTLCTNVEEENRIPSIESLKAVHPS
221

Query 202
DSSIQVVLVDRRADFDLGMLENYASSFLSSSSDMKDVINQLAKLVSSRMGGTTSNEEN-L
260
+SSI+V L+DR+ D DL LE+ F S + KD+++QLAKLV ++MGGT NEE+
L
Sbjct 222

ESSIEVALMDRQDDPDLRHLESVVGGFSCSCATAKDLVDQLAKLVCTQMGGTVFNEEDTL
281

Query 261
LPRWKESSEAIKSSAGSIVLHLGKLPIGLCKHRSLLFKMLADKVNIPCRLVKGCKYCKAE
320
L RWK+ SE +K+S+GS+V+ +GKL +GLC+ RSLLFKMLAD VN+PCR+
KGCKYCK++
Sbjct 282
LDRWKKCSETLKASSGSVVVPIGKLSVGLCRQRSLLFKMLADTVNLPCRVAKGCKYCKS
D 341

Query 321
DASSCVVRFGLEREYLVDLFGAPGQLSDPDSFVNGPYSLSVPSPLRPPKFRSLEITSNFS
380
DASSC+VRFGLEREYLVDL PG L +PDS VNGPYSLS+ SPLRPP+ + E+T
NF
Sbjct 342
DASSCLVRFGLEREYLVDLIRNPGNLCEPDSLVNGPYSLSISSPLRPPRVKVSELTVNFR
401

Query 381
SVAKQYFSDCHSLNLLFSDASTGASNGAAVAVDQMYSKKHDAGDGIANSWVPVKGQAIA
N 440
++AKQYF DC SLNL+F +AS A ++D S+ D S +P G ++
Sbjct 402
TLAKQYFLDCQSLNLIFKNAS-------ADSMDPSSSRPFDEKSAQIIS-LPSSGIQMSE 453

Query 441
S------DIILPEAPREV----LPLMSPSNLTADKKKEFQLIE-GNQYLRSTVSDLSLAV 489
S + P+ ++V L ++ PS+ A+ KK+F+L+E Q +D+ LAV
Sbjct 454
SPQSRIQKVARPDGYKDVANKFLQIIPPSDPKANMKKDFRLMEDSKQGGNKPSNDIGLAV
513

Query 490
DDLIIPWSELVLKEKIGAGSFGTVHRADWHGSDVAVKILMEQDYHLDRFKEFMREVAIMK
549
DDL IPW+ELVLKE+IGAGSFGTVHRADWHGSDVAVKILMEQD+H +R
+EF+REVAIMK
Sbjct 514
DDLEIPWTELVLKERIGAGSFGTVHRADWHGSDVAVKILMEQDFHPERLREFLREVAIMK
573

Query 550
SLRHPNIVLFMGAVTEPPNLSIVTEYLSRGSLYKLLHRSGAREVLDERRRLNMAFDVAKG
609
SLRHPNIVLFMGAVTEPP LSIVTEYLSRGSLY+LLH++G RE
LDERRRL+MAFDVAKG
Sbjct 574
SLRHPNIVLFMGAVTEPPKLSIVTEYLSRGSLYRLLHKTGGRETLDERRRLSMAFDVAKG
633

Query 610
MNYLHRRSPPIVHRDLKSPNLLVDKKYTVKVCDFGLSRLKANTYLSSKSLAGTPEWMAP
E 669

MNYLHRR+PPIVHRDLKSPNLLVDKKYTVKVCDFGLSRLKANT+LSSKSLAGTPEWMAPE
Sbjct 634
MNYLHRRNPPIVHRDLKSPNLLVDKKYTVKVCDFGLSRLKANTFLSSKSLAGTPEWMAP
E 693

Query 670
VLRDEPSNEKSDVYSFAVILWELMTLQQPWCNLNPAQVVAAVGFKGRRLEIPKELNPQVA
729
VLRDEPSNEKSDVYSF VILWELMTLQQPW NLN AQVVAAVGFKGRRLEIP
++NP+VA
Sbjct 694
VLRDEPSNEKSDVYSFGVILWELMTLQQPWSNLNSAQVVAAVGFKGRRLEIPSDVNPKVA
753

Query 730 ALIESCWANEPWRRPSFANIMETLRPLINKV 760
A+IESCW +EPW+RPSF++IME+++PLI +
Sbjct 754 AIIESCWTSEPWKRPSFSSIMESMKPLIKSL 784

>serine/threonine-protein kinase CTR1 [Canna indica]
Sequence ID: WOL15672.1 Length: 826
Range 1: 4 to 814

Score:897 bits(2319), Expect:0.0,
Method:Compositional matrix adjust.,
Identities:492/830(59%), Positives:591/830(71%), Gaps:95/830(11%)

Query 8 GGRRTSYSLLSQFPDD--------------AAAAGASP-------AVLQRQSSGGS---- 42
GRR++YSLLSQ PDD A A A P A LQRQSSG S

Sbjct 4
AGRRSTYSLLSQSPDDPPSPNFESPPISDKARAPRAPPFDWAPAAAPLQRQSSGSSFGES
63

Query 43
-----SYGAGSSVSASSDYPFHLPPA---------VAAAGGGGGTPSPCKSWAQQAEETY 88
Y A +++S+++D P AAA G + S +SWAQQAEETY
Sbjct 64
SLSGGDYYAPATLSSAADVDAFNPTVPRAKNSAAEAAAAVGSSSSSSSARSWAQQAEETY
123

Query 89
QLQLALALRLCADAACAADPGFLDPGDSGGSKMGGGGGGSGSGRAFPLAPPSPTAEALS
H 148
QLQLALALRLC++AACA DP FLD D + P ++SH
Sbjct 124 QLQLALALRLCSEAACADDPNFLDAVDQT------------------VLPEHAAPASISH
165

Query 149
RFWVNGSLSYSNTIPDGFYLIQGMDPFVWSMCTDVHEENRIPSVESLKSVRPDDSSIQVV
208
RFWVNG LSY + IPDGFYLIQGMDPF+W++CTDV EENRIPS+E+LK+V P
DSSI+V
Sbjct 166
RFWVNGCLSYHDKIPDGFYLIQGMDPFIWTLCTDVEEENRIPSIETLKTVHPSDSSIEVA
225

Query 209
LVDRRADFDLGMLENYASSFLSSSSDMKDVINQLAKLVSSRMGGTTSNEENLLPRWKESS
268
L+DR+ D DL L+ + SS+ +D+++QLA LV RMGGT S EE+LL RWK
S
Sbjct 226
LIDRQYDPDLRQLQTMVTGLSCSSATPEDIVDQLANLVCIRMGGTASREEDLLHRWKACS
285

Query 269
EAIKSSAGSIVLHLGKLPIGLCKHRSLLFKMLADKVNIPCRLVKGCKYCKAEDASSCVVR
328
EA+K S GS+VL +GKL +GLC+HRSLLFKMLAD +N+PCR+ KGC
YCKA+DAS+C+VR
Sbjct 286
EALKFSLGSVVLPIGKLSVGLCRHRSLLFKMLADTINLPCRIAKGCNYCKADDASACLVR
345

Query 329
FGLEREYLVDLFGAPGQLSDPDSFVNGPYSLSVPSPLRPPKFRSLEITSNFSSVAKQYFS
388
FG EREYLVDL G PG LS+P+S +NGP S+ +PSPLRPPK +S EIT NF ++AKQY
Sbjct 346
FGFEREYLVDLIGNPGNLSEPNSLLNGPNSILIPSPLRPPKVKSTEITVNFRALAKQYLL
405

Query 389 DCHSLNLLFSDASTGASNGAAV---AVDQMYSKKHDAGDGIA------------------
427
DC SLNL F+DAS S ++D + S +G ++
Sbjct 406
DCQSLNLFFNDASADPSLSRPFDEKSIDMISSPPTVSGTDVSELSQSQPHIQKVARPDSN
465

Query 428 NSWVPVKGQAIANSDIILPE---------------APREVLPLMSPSNLTADKKKEFQLI
472
++ +K ++I PE R+VL ++ PS+ K++F I
Sbjct 466
KDFIKLKNLLNPLQNVISPELLQQEMAQLKLTSQAGCRDVLQVIPPSDPNP-IKRDFGFI
524

Query 473
EGNQYLRST-VSDLSLAVDDLIIPWSELVLKEKIGAGSFGTVHRADWHGSDVAVKILMEQ
531
E + R+ +D+SLA+DDL IPWSEL
LKE+IGAGSFGTVHRA+WHGSDVAVKILMEQ
Sbjct 525
EDPRQGRNRHNNDISLAIDDLNIPWSELNLKERIGAGSFGTVHRAEWHGSDVAVKILMEQ
584

Query 532
DYHLDRFKEFMREVAIMKSLRHPNIVLFMGAVTEPPNLSIVTEYLSRGSLYKLLHRSGAR
591
D+H +R
+EF+REVAIMKSLRHPNIVLFMGAVTEPPNLSIVTEYLSRGSLY+LLHR+GAR
Sbjct 585
DFHPERLREFLREVAIMKSLRHPNIVLFMGAVTEPPNLSIVTEYLSRGSLYRLLHRNGAR
644

Query 592
EVLDERRRLNMAFDVAKGMNYLHRRSPPIVHRDLKSPNLLVDKKYTVKVCDFGLSRLKA
N 651

EVLDE+RRL+MAFDVAKGMNYLH+R+PPIVHRDLKSPNLLVDKKYTVKVCDFGLSRLKA
N
Sbjct 645
EVLDEKRRLSMAFDVAKGMNYLHKRNPPIVHRDLKSPNLLVDKKYTVKVCDFGLSRLKA
N 704

Query 652
TYLSSKSLAGTPEWMAPEVLRDEPSNEKSDVYSFAVILWELMTLQQPWCNLNPAQVVAAV
711
T+LSSKSLAGTPEWMAPEVLRDEPSNEKSDVYSF VILWELMTLQQPW
NLNPAQVVAAV
Sbjct 705
TFLSSKSLAGTPEWMAPEVLRDEPSNEKSDVYSFGVILWELMTLQQPWSNLNPAQVVAAV
764

Query 712 GFKGRRLEIPKELNPQVAALIESCWANEPWRRPSFANIMETLRPLINKVP
761
GFKGRRLEIP ++NP VAA+I+SCWANEPW+RP F++IM+T++ LI +P
Sbjct 765 GFKGRRLEIPSDVNPHVAAIIQSCWANEPWKRPPFSSIMDTVKHLIKSLP 814

>serine/threonine-protein kinase CTR1 isoform X1 [Ananas comosus]
Sequence ID: XP_020111529.1 Length: 821
Range 1: 118 to 809

Score:893 bits(2308), Expect:0.0,
Method:Compositional matrix adjust.,
Identities:448/709(63%), Positives:551/709(77%), Gaps:46/709(6%)

Query 78
KSWAQQAEETYQLQLALALRLCADAACAADPGFLDPGDSGGSKMGGGGGGSGSGRAFP
LA 137
KSWAQQAEETYQLQLALALRLC++AACA DP FLD GD ++ G
PLA
Sbjct 118
KSWAQQAEETYQLQLALALRLCSEAACATDPNFLDAGD----QIVGLQ---------PLA 164

Query 138
PPSPTAEALSHRFWVNGSLSYSNTIPDGFYLIQGMDPFVWSMCTDVHEENRIPSVESLKS
197
P AE+LSHRFWVNG LSY + +PDGFYLIQG+DPFVW++C DV +

NRIPS+ESLK+
Sbjct 165
P----AESLSHRFWVNGCLSYYDKVPDGFYLIQGIDPFVWTLCNDVQDGNRIPSIESLKA
220

Query 198
VRPDDSSIQVVLVDRRADFDLGMLENYASSFLSSSSDMKDVINQLAKLVSSRMGGTTSNE
257
+RP D+SI+VV++D+ DFDL +L+ A S+ KD ++ LA LV +R+GG S+E
Sbjct 221
IRPGDTSIEVVVIDKMGDFDLKLLQKMAVDISSTRPLSKDDVDLLASLVCTRLGGVASSE
280

Query 258
EN-LLPRWKESSEAIKSSAGSIVLHLGKLPIGLCKHRSLLFKMLADKVNIPCRLVKGCKY
316
E+ LLP WKES+E +K+S+ S+VL +GKL +GLC+HR+LLFK LAD +N+PCR+
+GC+Y
Sbjct 281
EHELLPLWKESNEILKASSASVVLPIGKLSVGLCRHRALLFKTLADSINLPCRVARGCRY
340

Query 317
CKAEDASSCVVRFGLEREYLVDLFGAPGQLSDPDSFVNGPYSLSVPSPLRPPKFRSLEIT
376
CK++DA+SC+VRFGLEREYL+DL G PG + +PDS NG S+ + SPLRPPK +++
IT
Sbjct 341
CKSDDAASCLVRFGLEREYLIDLIGNPGSVCEPDSLFNGLSSILISSPLRPPKHKAVGIT 400

Query 377 SNFSSVAKQYFSDCHSLNLLFSDAS----------------------TGASNGAAVAVD
413
NF S+A QYF DC SLN +FSDAS T + + A +
Sbjct 401
DNFRSLAAQYFLDCQSLNAMFSDASAAAVVDQEDTMCSFLDPNSFLHATNSDSEATHPRN
460

Query 414
QMYSKKHDAGDGIANSWVPVKGQAIANSDIILPEAPR----EVLPLMSPSNLTADKKKEF
469
Q ++ H + + Q I +S+ I + P V P++ ++ ADK F
Sbjct 461
QRIAQPHGQHGDLQPKKLCNSSQNIVSSEQIAQDQPHADVMNVSPILPFKDIKADKNTNF
520

Query 470
QLIEGNQYLR-STVSDLSLAVDDLIIPWSELVLKEKIGAGSFGTVHRADWHGSDVAVKIL
528
+ E N + + S
++D+SLAVDDL+IPWSELVLKEKIGAGSFGTVHRADW+GSDVAVKIL
Sbjct 521
DVREENHFGQCSALNDISLAVDDLMIPWSELVLKEKIGAGSFGTVHRADWNGSDVAVKIL
580

Query 529
MEQDYHLDRFKEFMREVAIMKSLRHPNIVLFMGAVTEPPNLSIVTEYLSRGSLYKLLHRS
588
MEQD+H +R EF+REVAIMKSLRHPNIVL
MGAVT+PPNLSIVTEYLSRGSLY+LLHR
Sbjct 581
MEQDFHPERLNEFLREVAIMKSLRHPNIVLLMGAVTQPPNLSIVTEYLSRGSLYRLLHRP
640

Query 589
GAREVLDERRRLNMAFDVAKGMNYLHRRSPPIVHRDLKSPNLLVDKKYTVKVCDFGLSR
L 648
GA+E
LDE+RRL+MAFDVAKGMNYLH+R+PPIVHRDLKSPNLLVDKKYTVKVCDFGLSRL
Sbjct 641
GAKETLDEKRRLSMAFDVAKGMNYLHKRNPPIVHRDLKSPNLLVDKKYTVKVCDFGLSR
L 700

Query 649
KANTYLSSKSLAGTPEWMAPEVLRDEPSNEKSDVYSFAVILWELMTLQQPWCNLNPAQV
V 708
KANT+LSSK+ AGTPEWMAPEVLRDEPSNEKSDVYSF+VILWELMTLQQPW
NLNPAQVV
Sbjct 701
KANTFLSSKTAAGTPEWMAPEVLRDEPSNEKSDVYSFSVILWELMTLQQPWSNLNPAQV
V 760

Query 709 AAVGFKGRRLEIPKELNPQVAALIESCWANEPWRRPSFANIMETLRPLI 757
AAVGFKGRRL+IP +++PQVAA+I+SCWANEPW+RPSF++IM++L+PLI
Sbjct 761 AAVGFKGRRLDIPSDVDPQVAAIIQSCWANEPWKRPSFSSIMKSLKPLI 809

>serine/threonine-protein kinase CTR1 isoform X1 [Magnolia sinica]
Sequence ID: XP_058085947.1 Length: 864
Range 1: 147 to 859

Score:879 bits(2271), Expect:0.0,
Method:Compositional matrix adjust.,
Identities:447/730(61%), Positives:546/730(74%), Gaps:59/730(8%)

Query 82
QQAEETYQLQLALALRLCADAACAADPGFLDPGDSGGSKMGGGGGGSGSGRAFPLAPPS
P 141
QQ EE+YQLQLALALRL ++A CA DP FL PG S + P
Sbjct 147 QQTEESYQLQLALALRLSSEATCADDPNFLCPGSEDSS----------------IGSPIA
190

Query 142
TAEALSHRFWVNGSLSYSNTIPDGFYLIQGMDPFVWSMCTDVHEENRIPSVESLKSVRPD
201
++E++SHRFWVNG LSY + +PDGFYLIQGMDP+VW++CTD+ E
RIPS+ESLK++ P
Sbjct 191
SSESVSHRFWVNGCLSYYDKVPDGFYLIQGMDPYVWTVCTDLQENGRIPSIESLKAMSPS
250

Query 202
DSSIQVVLVDRRADFDLGMLENYASSFLSSSSDMKDVINQLAKLVSSRMGGTTS-NEENL
260
DSS++VVL+D+R D L LEN S K+V+++LAKL+ RMGG S E
+L
Sbjct 251
DSSVEVVLIDKRGDPGLKELENKVVSLSCGCFTTKEVVDELAKLICRRMGGAASAGEGSL
310

Query 261
LPRWKESSEAIKSSAGSIVLHLGKLPIGLCKHRSLLFKMLADKVNIPCRLVKGCKYCKAE
320
+PRW+ S+ +K GSIV +G L +GLC+HR+LLFK+LAD +++PCR+
KGCKYC+ +
Sbjct 311
VPRWQHCSDTLKDCLGSIVFPVGSLSVGLCRHRALLFKVLADAIDLPCRIAKGCKYCRRD
370

Query 321
DASSCVVRFGLEREYLVDLFGAPGQLSDPDSFVNGPYSLSVPSPLRPPKFRSLEITSNFS

380
DA+SC+VRFG EREYLVDL G PG L +PDS +NGP S+ + SPLR P+F+++ I NF
Sbjct 371
DATSCLVRFGFEREYLVDLIGNPGSLCEPDSLLNGPSSILISSPLRLPRFKTVGIAQNFR 430

Query 381
SVAKQYFSDCHSLNLLFSDASTGASNGAAVAVDQMY----SKKHD--------AGDGIAN 428
S+AKQYFSDC SLNL+F DAS G+++G A D + +KH+ + + N
Sbjct 431
SLAKQYFSDCQSLNLIFDDASAGSASGQGDATDPSFPGPLDRKHEDMNYSLPVSINNDEN
490

Query 429 SWVPVKGQAIANSD---------------------IILPEAP------REVLPLMSPSN 460
S +P Q +A D + +P RE+ P+++ SN
Sbjct 491
SLLPKPNQGVARPDSRDDETQFQKWQNSQNGMVPQRFVEDHSPKNVQRQREIPPMIAISN
550

Query 461
LTADKKKEFQLIEGNQYL-RSTVSDLSLAVDDLIIPWSELVLKEKIGAGSFGTVHRADWH
519
DK KE +LI +Q + + + S+LSL VDDL
IPWS+LVLKE+IGAGSFGTVHRADWH
Sbjct 551
PRTDKNKELRLIGSSQVVPKRSDSELSLEVDDLDIPWSDLVLKERIGAGSFGTVHRADWH
610

Query 520
GSDVAVKILMEQDYHLDRFKEFMREVAIMKSLRHPNIVLFMGAVTEPPNLSIVTEYLSRG
579
GS+VAVKILMEQD+H ++FKEF+REVAIMK LRHPNIVLFMGAVT
PPNLSIVTEYLSRG
Sbjct 611
GSEVAVKILMEQDFHAEKFKEFLREVAIMKRLRHPNIVLFMGAVTRPPNLSIVTEYLSRG
670

Query 580
SLYKLLHRSGAREVLDERRRLNMAFDVAKGMNYLHRRSPPIVHRDLKSPNLLVDKKYTV
K 639
SLY+LLHR
GARE+LDERRRLNMA+DVAKGMNYLH+R+PPIVHRDLKSPNLLVD+KYTVK
Sbjct 671
SLYRLLHRPGAREMLDERRRLNMAYDVAKGMNYLHKRNPPIVHRDLKSPNLLVDRKYTV
K 730

Query 640
VCDFGLSRLKANTYLSSKSLAGTPEWMAPEVLRDEPSNEKSDVYSFAVILWELMTLQQPW
699
VCDFGLSRLK NT+LSSKSLAGTPEWMAPEVLRDEPSNEKSDVYSF
VILWEL+TLQQPW
Sbjct 731
VCDFGLSRLKENTFLSSKSLAGTPEWMAPEVLRDEPSNEKSDVYSFGVILWELLTLQQPW
790

Query 700
CNLNPAQVVAAVGFKGRRLEIPKELNPQVAALIESCWANEPWRRPSFANIMETLRPLINK
759
NLNPAQVVAAVGFK RRLEIP ++NPQVAALIESCW NEPW+RPSFA
IME+L+PL+ K
Sbjct 791
TNLNPAQVVAAVGFKCRRLEIPSDVNPQVAALIESCWINEPWKRPSFATIMESLKPLV-K
849

Query 760 VPVPQLIRSD 769
P PQ R++
Sbjct 850 PPTPQPGRAE 859

>serine/threonine-protein kinase CTR1 isoform X2 [Ananas comosus]
Sequence ID: XP_020111530.1 Length: 811
Range 1: 118 to 799

Score:874 bits(2258), Expect:0.0,
Method:Compositional matrix adjust.,
Identities:443/709(62%), Positives:544/709(76%), Gaps:56/709(7%)

Query 78
KSWAQQAEETYQLQLALALRLCADAACAADPGFLDPGDSGGSKMGGGGGGSGSGRAFP
LA 137
KSWAQQAEETYQLQLALALRLC++AACA DP FLD GD ++ G
PLA
Sbjct 118
KSWAQQAEETYQLQLALALRLCSEAACATDPNFLDAGD----QIVGLQ---------PLA 164

Query 138
PPSPTAEALSHRFWVNGSLSYSNTIPDGFYLIQGMDPFVWSMCTDVHEENRIPSVESLKS

197

P AE+LSHRFWV PDGFYLIQG+DPFVW++C DV + NRIPS+ESLK+

Sbjct 165 P----AESLSHRFWV----------PDGFYLIQGIDPFVWTLCNDVQDGNRIPSIESLKA 210

Query 198 VRPDDSSIQVVLVDRRADFDLGMLENYASSFLSSSSDMKDVINQLAKLVSSRMGGTTSNE 257

+RP D+SI+VV++D+ DFDL +L+ A S+ KD ++ LA LV +R+GG S+E

Sbjct 211 IRPGDTSIEVVVIDKMGDFDLKLLQKMAVDISSTRPLSKDDVDLLASLVCTRLGGVASSE 270

Query 258 EN-LLPRWKESSEAIKSSAGSIVLHLGKLPIGLCKHRSLLFKMLADKVNIPCRLVKGCKY 316

E+ LLP WKES+E +K+S+ S+VL +GKL +GLC+HR+LLFK LAD +N+PCR+ +GC+Y

Sbjct 271 EHELLPLWKESNEILKASSASVVLPIGKLSVGLCRHRALLFKTLADSINLPCRVARGCRY 330

Query 317 CKAEDASSCVVRFGLEREYLVDLFGAPGQLSDPDSFVNGPYSLSVPSPLRPPKFRSLEIT 376

CK++DA+SC+VRFGLEREYL+DL G PG + +PDS NG S+ + SPLRPPK +++ IT

Sbjct 331 CKSDDAASCLVRFGLEREYLIDLIGNPGSVCEPDSLFNGLSSILISSPLRPPKHKAVGIT 390

Query 377 SNFSSVAKQYFSDCHSLNLLFSDAS----------------------TGASNGAAVAVD 413

NF S+A QYF DC SLN +FSDAS T + + A +

Sbjct 391 DNFRSLAAQYFLDCQSLNAMFSDASAAAVVDQEDTMCSFLDPNSFLHATNSDSEATHPRN 450

Query 414 QMYSKKHDAGDGIANSWVPVKGQAIANSDIILPEAPR----EVLPLMSPSNLTADKKKEF 469

Q ++ H + + Q I +S+ I + P V P++ ++ ADK F

Sbjct 451 QRIAQPHGQHGDLQPKKLCNSSQNIVSSEQIAQDQPHADVMNVSPILPFKDIKADKNTNF

510

Query 470
QLIEGNQYLR-STVSDLSLAVDDLIIPWSELVLKEKIGAGSFGTVHRADWHGSDVAVKIL
528
+ E N + + S
++D+SLAVDDL+IPWSELVLKEKIGAGSFGTVHRADW+GSDVAVKIL
Sbjct 511
DVREENHFGQCSALNDISLAVDDLMIPWSELVLKEKIGAGSFGTVHRADWNGSDVAVKIL
570

Query 529
MEQDYHLDRFKEFMREVAIMKSLRHPNIVLFMGAVTEPPNLSIVTEYLSRGSLYKLLHRS
588
MEQD+H +R EF+REVAIMKSLRHPNIVL
MGAVT+PPNLSIVTEYLSRGSLY+LLHR
Sbjct 571
MEQDFHPERLNEFLREVAIMKSLRHPNIVLLMGAVTQPPNLSIVTEYLSRGSLYRLLHRP
630

Query 589
GAREVLDERRRLNMAFDVAKGMNYLHRRSPPIVHRDLKSPNLLVDKKYTVKVCDFGLSR
L 648
GA+E
LDE+RRL+MAFDVAKGMNYLH+R+PPIVHRDLKSPNLLVDKKYTVKVCDFGLSRL
Sbjct 631
GAKETLDEKRRLSMAFDVAKGMNYLHKRNPPIVHRDLKSPNLLVDKKYTVKVCDFGLSR
L 690

Query 649
KANTYLSSKSLAGTPEWMAPEVLRDEPSNEKSDVYSFAVILWELMTLQQPWCNLNPAQV
V 708
KANT+LSSK+ AGTPEWMAPEVLRDEPSNEKSDVYSF+VILWELMTLQQPW
NLNPAQVV
Sbjct 691
KANTFLSSKTAAGTPEWMAPEVLRDEPSNEKSDVYSFSVILWELMTLQQPWSNLNPAQV
V 750

Query 709 AAVGFKGRRLEIPKELNPQVAALIESCWANEPWRRPSFANIMETLRPLI 757
AAVGFKGRRL+IP +++PQVAA+I+SCWANEPW+RPSF++IM++L+PLI
Sbjct 751 AAVGFKGRRLDIPSDVDPQVAAIIQSCWANEPWKRPSFSSIMKSLKPLI 799

>serine/threonine-protein kinase CTR1-like [Tripterygium wilfordii]
Sequence ID: XP_038702140.1 Length: 866
>serine/threonine-protein kinase CTR1 [Tripterygium wilfordii]
Sequence ID: KAF5747830.1 Length: 866
Range 1: 135 to 861

Score:870 bits(2249), Expect:0.0,
Method:Compositional matrix adjust.,
Identities:456/749(61%), Positives:556/749(74%), Gaps:61/749(8%)

Query 60
LPPAVAAAGGGGGTPSPCKSWAQQAEETYQLQLALALRLCADAACAADPGFLDPGDSGG
S 119
P AVA G G S KSWAQQ EE+YQ+QL LALRL +D ACA DP FLDPG
Sbjct 135
FPEAVA----GTGVSSSGKSWAQQTEESYQIQLELALRLSSDVACADDPNFLDPGPD--- 187

Query 120
KMGGGGGGSGSGRAFPLAPPSPTAEALSHRFWVNGSLSYSNTIPDGFYLIQGMDPFVWSM
179
+ R+ L +AEA+SHRFWVNG LSY + IPDGFYLI
GMDP+VW++
Sbjct 188
--------ESAVRSLSLR----SAEAVSHRFWVNGCLSYFDKIPDGFYLIHGMDPYVWTV 235

Query 180
CTDVHEENRIPSVESLKSVRPD-DSSIQVVLVDRRADFDLGMLENYASSFLSSSSDMKDV
238
C D+ E RIPS+ESLKS+ P D+SI+VVL+D D L L+N S S+ ++V
Sbjct 236
CIDLQENGRIPSLESLKSIDPRMDTSIEVVLMDHHTDSSLKELQNRVLSISSNCITTQEV
295

Query 239
INQLAKLVSSRMGGT-TSNEENLLPRWKESSEAIKSSAGSIVLHLGKLPIGLCKHRSLLF
297
+QLAKLV + MGG+ T+ E + + W+E+ + +K SIVL LG L +GLC+HR+LLF
Sbjct 296
ADQLAKLVCNHMGGSVTTGEHDFISIWRENIDDLKDRLRSIVLPLGSLSVGLCRHRALLF
355

Query 298
KMLADKVNIPCRLVKGCKYCKAEDASSCVVRFGLEREYLVDLFGAPGQLSDPDSFVNGP

Y 357
K+LAD +++PCR+ KGCKYCK DASSC+VRFGL+REYLVDL G PG L +PDS
+NGP
Sbjct 356
KVLADTIDLPCRIAKGCKYCKRADASSCLVRFGLDREYLVDLIGKPGHLWEPDSLLNGPS
415

Query 358
SLSVPSPLRPPKFRSLEITSNFSSVAKQYFSDCHSLNLLFSDASTGASNGAAVAVDQ--- 414
S+S+ SPLR P+ +S+E T +F S+A+ YFSDC SLNL+F +AS AS G A+ +
Sbjct 416
SISISSPLRFPRLQSVEPTIDFRSLARTYFSDCQSLNLVFDEAS--ASAGTALNKEDPGF 473

Query 415 -MYSKKHDA--------------GDGIANSWVPVK----GQAIANSDII--------LPE 447
+Y K++D G+ I+ PV+ G +A+S ++ P
Sbjct 474
SLYPKQYDKMGRDRNNVVQIPRNGNEISQLTPPVRVGLPGDQVADSRLLNLKNLIKDFPL
533

Query 448
APREVL------PLMSPSNLTADKKKEFQLIEGNQYLRSTVS-DLSLAVDDLIIPWSELV 500
P ++ P+++ S+ D K+ + EG+Q + S S +LS V+DL IPWS+LV
Sbjct 534
KPIQLTAHCDGQPILTSSDQRVDATKDLKFAEGSQLVSSRPSKELSFDVEDLDIPWSDLV
593

Query 501
LKEKIGAGSFGTVHRADWHGSDVAVKILMEQDYHLDRFKEFMREVAIMKSLRHPNIVLF
M 560
LKE+IGAGSFGTVHRADWHGS+VAVKILMEQD+H +RFKEF+REVAIMK
LRHPNIVLFM
Sbjct 594
LKERIGAGSFGTVHRADWHGSEVAVKILMEQDFHAERFKEFLREVAIMKRLRHPNIVLFM
653

Query 561
GAVTEPPNLSIVTEYLSRGSLYKLLHRSGAREVLDERRRLNMAFDVAKGMNYLHRRSPPI
620

GAVT+PPNLSIVTEYLSRGSLY+LLH+SGAREVLDERRRL+MA+DVAKG+NYLHRR+PPI
Sbjct 654
GAVTQPPNLSIVTEYLSRGSLYRLLHKSGAREVLDERRRLSMAYDVAKGLNYLHRRNPPI
713

Query 621
VHRDLKSPNLLVDKKYTVKVCDFGLSRLKANTYLSSKSLAGTPEWMAPEVLRDEPSNEK
S 680
VHRDLKSPNLLVDKKYTVKVCDFGLSR KANT+LSSKS
AGTPEWMAPEVLRDEPSNEKS
Sbjct 714
VHRDLKSPNLLVDKKYTVKVCDFGLSRFKANTFLSSKSAAGTPEWMAPEVLRDEPSNEK
S 773

Query 681
DVYSFAVILWELMTLQQPWCNLNPAQVVAAVGFKGRRLEIPKELNPQVAALIESCWANEP
740
DVYSF VILWEL TLQQPW NLNPAQVVAAVGFKG+R EIP+
LNPQV+A+IE+CWANEP
Sbjct 774
DVYSFGVILWELATLQQPWGNLNPAQVVAAVGFKGKRPEIPRNLNPQVSAIIETCWANEP
833

Query 741 WRRPSFANIMETLRPLINKVPVPQLIRSD 769
W+RPSFA+IME+LRPLI K P PQ R+D
Sbjct 834 WKRPSFASIMESLRPLI-KPPTPQPGRAD 861

>hypothetical protein LUZ63_009036 [Rhynchospora breviuscula]
Sequence ID: KAJ1692338.1 Length: 742
Range 1: 1 to 732

Score:870 bits(2248), Expect:0.0,
Method:Compositional matrix adjust.,
Identities:479/781(61%), Positives:566/781(72%), Gaps:69/781(8%)

Query 1
MELPAAGGGRRTSYSLLSQFPDD-----AAAAGASPAVLQRQSSGGSSYGAGSSVSASSD 55
ME+P G +YS+L+Q DD P LQRQSSG S SS+S S D
Sbjct 1
MEIPKRGTN---TYSVLNQLSDDYDPSLPPLPLPLPLQLQRQSSGSSY--GDSSLSLSGD 55

Query 56
YPFHLPPAVAAAGGGG--------GTPSPCKSWAQQAEETYQLQLALALRLCADAACAAD
107
F PP A G KSWAQQAEETYQLQLALALRLC++AA
+D

Sbjct 56
CCFFAPPLDAITGAKSEPITGGSGSGSGSGKSWAQQAEETYQLQLALALRLCSEAASVSD
115

Query 108
PGFLDPGDSGGSKMGGGGGGSGSGRAFPLAPPSPTAEALSHRFWVNGSLSYSNTIPDGFY
167
PG ++ GD+ + P+ + +AEALSH FWVNGSLSY N+I DGFY
Sbjct 116 PGLMELGDASTA---------------PVTSSAVSAEALSHCFWVNGSLSYINSIQDGFY
160

Query 168
LIQGMDPFVWSMCTDVHEENRIPSVESLKSVRPDDSSIQVVLVDRRADFDLGMLENYASS
227
+I+GMDPFVWS+CTDV EE+R+P+++SLK+V DSSI+VVL D++ D L L +
S
Sbjct 161
VIRGMDPFVWSLCTDVQEESRMPTLDSLKNVDWSDSSIEVVLFDKKIDSYLRQLHSSVVS
220

Query 228
FLS---SSSDMKDVINQLAKLVSSRMGGTTSN---EENLLPRWKESSEAIKSSAGSIVLH 281
S +++ KD++ QLAKLVSSRMGG N EE LL RW++S+ + S S+V+
Sbjct 221
TFSSFSATASTKDMVEQLAKLVSSRMGGAALNLKDEEALLNRWRDSNSTARMSTHSVVIP
280

Query 282
LGKLPIGLCKHRSLLFKMLADKVNIPCRLVKGCKYCKAEDASSCVVRFGLEREYLVDLFG
341
LG+L +GLC HRSLLFKMLAD VN+PCR+VKGCKYC
+DA+SC+VRFGLEREYLVDL G
Sbjct 281
LGQLSVGLCIHRSLLFKMLADSVNVPCRVVKGCKYCTRDDATSCLVRFGLEREYLVDLIG
340

Query 342
APGQLSDPDSFVNGPYSLSVPSPLRPPKFRSLEITSNFSSVAKQYFSDCHSLNLLFSDAS
401
PG LS+PDS +NGP+S+ V SPL PP+F+S+EI NF S+AKQYFS+C SLNL+ D
Sbjct 341
DPGHLSEPDSQLNGPHSMFVSSPLHPPRFKSMEINGNFKSLAKQYFSECQSLNLILKDTK
400

Query 402
TGASNGAAVAVDQMYSKKHDAGDGIANSWVPVKGQAIANSDIILPEAP-REVLPLMSPSN
460
T + V+ DQ G + VP ++ S++ P+ + L ++PS
Sbjct 401 TTSD---LVSSDQ-------TGAYV----VPAAAPSLPFSNLRPPDMKITDSLARINPSR
446

Query 461
LTADKKKEFQLIEGNQYLRSTVSDLSLAVDDLIIPWSELVLKEKIGAGSFGTVHRADWHG
520
T S D+ +VDDL IPW
EL+LKEKIGAGSFGTVHRADWHG
Sbjct 447
TTNP---------------SPNRDVVFSVDDLAIPWRELILKEKIGAGSFGTVHRADWHG 491

Query 521
SDVAVKILMEQDYHLDRFKEFMREVAIMKSLRHPNIVLFMGAVTEPPNLSIVTEYLSRGS
580
SDVAVKIL+EQD+H DR KEFMREV +MKSLRHPNIVLFMG
VTEPPNLSIVTEYLSRGS
Sbjct 492
SDVAVKILIEQDFHADRLKEFMREVVMMKSLRHPNIVLFMGVVTEPPNLSIVTEYLSRGS
551

Query 581
LYKLLHRSGAREVLDERRRLNMAFDVAKGMNYLHRRSPPIVHRDLKSPNLLVDKKYTVK
V 640
LYKLLHRS RE
LDERRRL+MAFDVAKGMNYLHRR+PPIVHRDLKSPNLLVD+KYTVKV
Sbjct 552
LYKLLHRSSVREYLDERRRLHMAFDVAKGMNYLHRRNPPIVHRDLKSPNLLVDRKYTVK
V 611

Query 641
CDFGLSRLKANTYLSSKSLAGTPEWMAPEVLRDEPSNEKSDVYSFAVILWELMTLQQPWC
700
CDFGLSRLKA+TYLSSKSLAGTPEWMAPEVLRDEPSNEKSDVYSF
V+LWELMTLQQPW
Sbjct 612
CDFGLSRLKASTYLSSKSLAGTPEWMAPEVLRDEPSNEKSDVYSFGVVLWELMTLQQPW
S 671

Query 701
NLNPAQVVAAVGFKGRRLEIPKELNPQVAALIESCWANEPWRRPSFANIMETLRPLINKV

760
NLNPAQVVAAVGFKGRRLEIPKELNP+VA+LIESCWA+EPW+RPSF
+IME+L+PL+ K
Sbjct 672
NLNPAQVVAAVGFKGRRLEIPKELNPRVASLIESCWADEPWKRPSFGSIMESLKPLLAKS
731

Query 761 P 761
P
Sbjct 732 P 732

>hypothetical protein LUZ61_020752 [Rhynchospora tenuis]
Sequence ID: KAJ3691588.1 Length: 751
Range 1: 57 to 741

Score:870 bits(2247), Expect:0.0,
Method:Compositional matrix adjust.,
Identities:459/729(63%), Positives:537/729(73%), Gaps:66/729(9%)

Query 55
DYPFHLPPAVAAAGGGGGTP---------------SPCKSWAQQAEETYQLQLALALRLC 99
D F PP + A G P
KSWAQQAEETYQLQLALALRLC
Sbjct 57
DCCFFAPPPLDAFTGAKSEPITGGCEMGGGGGSGSGSSKSWAQQAEETYQLQLALALRLC
116

Query 100
ADAACAADPGFLDPGDSGGSKMGGGGGGSGSGRAFPLAPPSPTAEALSHRFWVNGSLSY
S 159
++AA +DPG ++ GD+ + + G
+AEALSHRFWVNGSLSY
Sbjct 117
SEAASVSDPGLMESGDASSAPVTSSGA---------------SAEALSHRFWVNGSLSYI 161

Query 160
NTIPDGFYLIQGMDPFVWSMCTDVHEENRIPSVESLKSVRPDDSSIQVVLVDRRADFDLG
219
+ I DGFY+I+GMDPFVWS+CTDV EE+R+PS++SLK+V DSSI+VVL D++ D
L
Sbjct 162

DAIQDGFYVIRGMDPFVWSLCTDVQEESRMPSLDSLKNVDWSDSSIEVVLFDKKIDSYLR
221

Query 220
MLENYASSFLS---SSSDMKDVINQLAKLVSSRMGGTTSN---EENLLPRWKESSEAIKS 273
L + S S +++ +D++ QLAKLVSSRMGG N EE LL RW+ES+ +
Sbjct 222
QLHSSVVSTFSSFSATASTQDMVEQLAKLVSSRMGGAALNSIDEETLLSRWRESNSTARM
281

Query 274
SAGSIVLHLGKLPIGLCKHRSLLFKMLADKVNIPCRLVKGCKYCKAEDASSCVVRFGLER
333
S S+V+ LG+L +GLC HRSLLFKMLAD VN+PCR+VKGCKYC +DA+SC+VRFGLER
Sbjct 282
STHSVVIPLGQLSVGLCIHRSLLFKMLADSVNVPCRVVKGCKYCTRDDATSCLVRFGLER
341

Query 334
EYLVDLFGAPGQLSDPDSFVNGPYSLSVPSPLRPPKFRSLEITSNFSSVAKQYFSDCHSL
393
EYLVDL G PG LS+PDS +NGP+S+ V SPL PP+ +S+EI NF S+AKQYF +C SL
Sbjct 342
EYLVDLIGDPGHLSEPDSQLNGPHSICVSSPLHPPRSKSMEINGNFKSLAKQYFLECQSL
401

Query 394
NLLFSDASTGASNGAAVAVDQMYSKKHDAGDGIANSWVPVKGQAIANSDIILPEAP-REV
452
NL+F DA T + V+ DQ + VP ++ S++ P+ E
Sbjct 402 NLIFKDAKTTSD---LVSSDQTSAYV-----------VPAAAPSLPFSNLRPPDVKITES
447

Query 453
LPLMSPSNLTADKKKEFQLIEGNQYLRSTVSDLSLAVDDLIIPWSELVLKEKIGAGSFGT
512
L ++PS T ST D+ +VDDL IPW EL+LKEKIGAGSFGT
Sbjct 448 LARINPSRTTNP---------------STNRDIVFSVDDLTIPWRELILKEKIGAGSFGT
492

Query 513
VHRADWHGSDVAVKILMEQDYHLDRFKEFMREVAIMKSLRHPNIVLFMGAVTEPPNLSIV
572

VHRADWHGSDVAVKIL+EQD+H DR KEFMREV IMKSLRHPNIVLFMG VTEPPNLSIV
Sbjct 493 VHRADWHGSDVAVKILIEQDFHADRIKEFMREVVIMKSLRHPNIVLFMGVVTEPPNLSIV 552

Query 573 TEYLSRGSLYKLLHRSGAREVLDERRRLNMAFDVAKGMNYLHRRSPPIVHRDLKSPNLLV 632
TEYLSRGSLYKLLH+S RE LDERRRL+MAFDVAKGMNYLHRR+PPIVHRDLKSPNLLV
Sbjct 553 TEYLSRGSLYKLLHKSSVREYLDERRRLHMAFDVAKGMNYLHRRNPPIVHRDLKSPNLLV 612

Query 633 DKKYTVKVCDFGLSRLKANTYLSSKSLAGTPEWMAPEVLRDEPSNEKSDVYSFAVILWEL 692
D+KYTVKVCDFGLSRLKA+TYLSSKSLAGTPEWMAPEVLRDE SNEKSDVYSF VILWEL
Sbjct 613 DRKYTVKVCDFGLSRLKASTYLSSKSLAGTPEWMAPEVLRDELSNEKSDVYSFGVILWEL 672

Query 693 MTLQQPWCNLNPAQVVAAVGFKGRRLEIPKELNPQVAALIESCWANEPWRRPSFANIMET 752
MTLQQPW NLNPAQVVAAVGFKGRRLEIPKELNP+VA+LIESCWANE +RPSFA+IME+
Sbjct 673 MTLQQPWSNLNPAQVVAAVGFKGRRLEIPKELNPRVASLIESCWANEIRKRPSFASIMES 732

Query 753 LRPLINKVP 761
L+PL+ K P
Sbjct 733 LKPLLVKSP 741

>protein kinase family protein [Rhynchospora pubera]
Sequence ID: KAJ4783134.1 Length: 753
Range 1: 1 to 743

Score:869 bits(2246), Expect:0.0,
Method:Compositional matrix adjust.,
Identities:482/792(61%), Positives:565/792(71%), Gaps:80/792(10%)

Query 1
MELPAAGGGRRTSYSLLSQFPDD-------AAAAGASPAVLQRQSSGGSSYGAGSSVSAS 53
ME+P G +YS+L+Q DD P LQRQSSG S SS+S S
Sbjct 1
MEIPKRGTN---TYSVLNQLSDDYDPSLPPLPLPLPLPLQLQRQSSGSSY--GDSSLSLS 55

Query 54
SDYPFHLPPAVAAAGGGGGTP-----------------SPCKSWAQQAEETYQLQLALAL 96
D F PP + A G P
KSWAQQAEETYQLQLALAL
Sbjct 56
GDCCFFAPPPLDAITGAKSEPITGGCEMGGGGGSGSGSGSGKSWAQQAEETYQLQLALAL
115

Query 97
RLCADAACAADPGFLDPGDSGGSKMGGGGGGSGSGRAFPLAPPSPTAEALSHRFWVNGS
L 156
RLC++AA +DPG ++ GD+ + P+ +
+AEALSHRFWVNGSL
Sbjct 116
RLCSEAASVSDPGLMESGDASTA---------------PVTSSAVSAEALSHRFWVNGSL 160

Query 157
SYSNTIPDGFYLIQGMDPFVWSMCTDVHEENRIPSVESLKSVRPDDSSIQVVLVDRRADF
216
SY ++I DGFY+I+GMDPFVWS+CTDV EE+R+P++ SLK+V DSSI+VVL D++
D
Sbjct 161
SYIDSIQDGFYVIRGMDPFVWSLCTDVQEESRMPTLVSLKNVDWSDSSIEVVLFDKKIDS
220

Query 217
DLGMLENYASSFLS---SSSDMKDVINQLAKLVSSRMGGTTSN---EENLLPRWKESSEA
270
L L N S S +++ KD++ QLAKLVSSRMGG N EE LL RW++S+
Sbjct 221
YLRQLHNSVVSTFSSFSATASTKDMVEQLAKLVSSRMGGAAPNLKDEEALLSRWRDSNST
280

Query 271

IKSSAGSIVLHLGKLPIGLCKHRSLLFKMLADKVNIPCRLVKGCKYCKAEDASSCVVRFG 330
+ S S+V+ LG+L +GLC HRSLLFKMLAD VN+PCR+VKGCKYC +DA+SC+VRFG
Sbjct 281 ARMSTHSVVIPLGQLSVGLCIHRSLLFKMLADSVNVPCRVVKGCKYCTRDDATSCLVRFG 340

Query 331 LEREYLVDLFGAPGQLSDPDSFVNGPYSLSVPSPLRPPKFRSLEITSNFSSVAKQYFSDC 390
LEREYLVDL G PG LS+PDS +NGP S+ V SPL PP+F+S+E NF S+AKQYFS+C
Sbjct 341 LEREYLVDLIGDPGHLSEPDSQLNGPNSMFVSSPLHPPRFKSMERNGNFKSLAKQYFSEC 400

Query 391 HSLNLLFSDASTGASNGAAVAVDQMYSKKHDAGDGIANSWVPVKGQAIANSDIILPEAP- 449
SLNL F D T + V+ DQ G + VP ++ S++ P+
Sbjct 401 QSLNLCFKDTKTTSD---LVSSDQ-------TGACV----VPAAAPSLPFSNLRPPDMKI 446

Query 450 REVLPLMSPSNLTADKKKEFQLIEGNQYLRSTVSDLSLAVDDLIIPWSELVLKEKIGAGS 509
E L ++PS T S D+ +VDDL IPW EL+LKEKIGAGS
Sbjct 447 TESLARINPSRTTNP---------------SPNRDIVFSVDDLTIPWRELILKEKIGAGS 491

Query 510 FGTVHRADWHGSDVAVKILMEQDYHLDRFKEFMREVAIMKSLRHPNIVLFMGAVTEPPNL 569
FGTVHRADWHGSDVAVKIL+EQD+H DR KEFMREV IMKSLRHPNIVLFMG VTEPPNL
Sbjct 492 FGTVHRADWHGSDVAVKILIEQDFHADRLKEFMREVIIMKSLRHPNIVLFMGVVTEPPNL 551

Query 570 SIVTEYLSRGSLYKLLHRSGAREVLDERRRLNMAFDVAKGMNYLHRRSPPIVHRDLKSPN 629
SIVTEYLSRGSLYKLLH+S RE LDERRRL+MAFDVAKGMNYLHRR+PPIVHRDLKSPN

Sbjct 552
SIVTEYLSRGSLYKLLHKSTVREYLDERRRLHMAFDVAKGMNYLHRRNPPIVHRDLKSPN
611

Query 630
LLVDKKYTVKVCDFGLSRLKANTYLSSKSLAGTPEWMAPEVLRDEPSNEKSDVYSFAVIL
689
LLVD+KYTVKVCDFGLSRLKA+TYLSSKSLAGTPEWMAPEVLRDE
SNEKSDVYSF VIL
Sbjct 612
LLVDRKYTVKVCDFGLSRLKASTYLSSKSLAGTPEWMAPEVLRDELSNEKSDVYSFGVIL
671

Query 690
WELMTLQQPWCNLNPAQVVAAVGFKGRRLEIPKELNPQVAALIESCWANEPWRRPSFANI
749
WELMTLQQPW NLNPAQVVAAVGFKGRRLEIPKELNP+V
+LIESCWA+EPW+RPSFA+I
Sbjct 672
WELMTLQQPWSNLNPAQVVAAVGFKGRRLEIPKELNPRVTSLIESCWADEPWKRPSFASI
731

Query 750 METLRPLINKVP 761
ME+L+PL+ K P
Sbjct 732 MESLKPLLVKSP 743

>protein kinase family protein [Rhynchospora pubera]
Sequence ID: KAJ4802344.1 Length: 749
Range 1: 1 to 739

Score:868 bits(2242), Expect:0.0,
Method:Compositional matrix adjust.,
Identities:482/792(61%), Positives:563/792(71%), Gaps:84/792(10%)

Query 1
MELPAAGGGRRTSYSLLSQFPDD-------AAAAGASPAVLQRQSSGGSSYGAGSSVSAS 53
ME+P G +YS+L+Q DD P LQRQSSG S SS+S S
Sbjct 1
MEIPKRGTN---TYSVLNQLSDDYDPSLPPLPLPLPLPLQLQRQSSGSSY--GDSSLSLS 55

Query 54

SDYPFHLPPAVAAAGGGGGTP-----------------SPCKSWAQQAEETYQLQLALAL 96
D F PP + A G P KSWAQQAEETYQLQLALAL
Sbjct 56 GDCCFFAPPPLDAITGAKSEPITGGCEMGGGGGSGSGSGSGKSWAQQAEETYQLQLALAL 115

Query 97 RLCADAACAADPGFLDPGDSGGSKMGGGGGGSGSGRAFPLAPPSPTAEALSHRFWVNGSL 156
RLC++AA +DPG ++ GD+ S + +AEALSHRFWVNGSL
Sbjct 116 RLCSEAASVSDPGLMESGDASTSS-------------------AVSAEALSHRFWVNGSL 156

Query 157 SYSNTIPDGFYLIQGMDPFVWSMCTDVHEENRIPSVESLKSVRPDDSSIQVVLVDRRADF 216
SY ++I DGFY+I+GMDPFVWS+CTDV EE+R+P++ SLK+V DSSI+VVL D++ D
Sbjct 157 SYIDSIQDGFYVIRGMDPFVWSLCTDVQEESRMPTLVSLKNVDWSDSSIEVVLFDKKIDS 216

Query 217 DLGMLENYASSFLS---SSSDMKDVINQLAKLVSSRMGGTTSN---EENLLPRWKESSEA 270
L L N S S +++ KD++ QLAKLVSSRMGG N EE LL RW++S+
Sbjct 217 YLRQLHNSVVSTFSSFSATASTKDMVEQLAKLVSSRMGGAAPNLKDEEALLSRWRDSNST 276

Query 271 IKSSAGSIVLHLGKLPIGLCKHRSLLFKMLADKVNIPCRLVKGCKYCKAEDASSCVVRFG 330
+ S S+V+ LG+L +GLC HRSLLFKMLAD VN+PCR+VKGCKYC +DA+SC+VRFG
Sbjct 277 ARMSTHSVVIPLGQLSVGLCIHRSLLFKMLADSVNVPCRVVKGCKYCTRDDATSCLVRFG 336

Query 331 LEREYLVDLFGAPGQLSDPDSFVNGPYSLSVPSPLRPPKFRSLEITSNFSSVAKQYFSDC 390

LEREYLVDL G PG LS+PDS +NGP S+ V SPL PP+F+S+E NF S+AKQYFS+C

Sbjct 337
LEREYLVDLIGDPGHLSEPDSHLNGPNSMFVSSPLHPPRFKSMERNGNFKSLAKQYFSEC
396

Query 391
HSLNLLFSDASTGASNGAAVAVDQMYSKKHDAGDGIANSWVPVKGQAIANSDIILPEAP-
449

SLNL F D T + V+ DQ G + VP ++ S++ P+

Sbjct 397 QSLNLSFKDTKTTSD---LVSSDQ-------TGACV----VPAAAPSLPFSNLRPPDMKI
442

Query 450
REVLPLMSPSNLTADKKKEFQLIEGNQYLRSTVSDLSLAVDDLIIPWSELVLKEKIGAGS
509

E L ++PS T S D+ +VDDL IPW EL+LKEKIGAGS

Sbjct 443 TESLARINPSRTTNP---------------SPNRDIVFSVDDLTIPWRELILKEKIGAGS
487

Query 510
FGTVHRADWHGSDVAVKILMEQDYHLDRFKEFMREVAIMKSLRHPNIVLFMGAVTEPPN
L 569

FGTVHRADWHGSDVAVKIL+EQD+H DR KEFMREV IMKSLRHPNIVLFMG
VTEPPNL

Sbjct 488
FGTVHRADWHGSDVAVKILIEQDFHADRLKEFMREVIIMKSLRHPNIVLFMGVVTEPPNL
547

Query 570
SIVTEYLSRGSLYKLLHRSGAREVLDERRRLNMAFDVAKGMNYLHRRSPPIVHRDLKSPN
629

SIVTEYLSRGSLYKLLH+S RE
LDERRRL+MAFDVAKGMNYLHRR+PPIVHRDLKSPN

Sbjct 548
SIVTEYLSRGSLYKLLHKSTVREYLDERRRLHMAFDVAKGMNYLHRRNPPIVHRDLKSPN
607

Query 630
LLVDKKYTVKVCDFGLSRLKANTYLSSKSLAGTPEWMAPEVLRDEPSNEKSDVYSFAVIL
689

LLVD+KYTVKVCDFGLSRLKA+TYLSSKSLAGTPEWMAPEVLRDE
SNEKSDVYSF VIL

Sbjct 608
LLVDRKYTVKVCDFGLSRLKASTYLSSKSLAGTPEWMAPEVLRDELSNEKSDVYSFGVIL

667

Query 690
WELMTLQQPWCNLNPAQVVAAVGFKGRRLEIPKELNPQVAALIESCWANEPWRRPSFANI
749
WELMTLQQPW NLNPAQVVAAVGFKGRRLEIPKELNP+V
+LIESCWA+EPW+RPSFA+I
Sbjct 668
WELMTLQQPWSNLNPAQVVAAVGFKGRRLEIPKELNPRVTSLIESCWADEPWKRPSFASI
727

Query 750 METLRPLINKVP 761
ME+L+PL+ K P
Sbjct 728 MESLKPLLVKSP 739

>Serine/threonine-protein kinase CTR1 [Cajanus cajan]
Sequence ID: KYP73796.1 Length: 775
Range 1: 1 to 766

Score:868 bits(2242), Expect:0.0,
Method:Compositional matrix adjust.,
Identities:466/802(58%), Positives:577/802(71%), Gaps:74/802(9%)

Query 1
MELPAAGGGRRTSYSLLSQFPDDAAAAGASPAVLQRQSSGGSSYGAGSSVSASSDYPFHL
60
ME+PA RR++YSLLSQ PDD + A+P+ SSG G G + D+
Sbjct 1
MEMPA----RRSNYSLLSQIPDDQFSGPAAPS-----SSGDGKNGRGGKADRAFDWDLVA 51

Query 61
PPAVAAAG----GGGGTPSPCKSWAQQAEETYQLQLALALRLCADAACAADPGFLDP-GD
115
A G G + S KSWAQQ EE+YQLQLALALRL +DA CA DP FLDP
D
Sbjct 52
DHRAAQQGTNRIGNLISGSSGKSWAQQTEESYQLQLALALRLSSDATCADDPNFLDPVPD
111

Query 116
SGGSKMGGGGGGSGSGRAFPLAPPSPTAEALSHRFWVNGSLSYSNTIPDGFYLIQGMDPF

175

++ S +AEA+SHRFWVNG LSYS+ IPDGFYLI GMD +

Sbjct 112 DSSMRL-----------------SSSAEAVSHRFWVNGCLSYSDKIPDGFYLIHGMDSY
153

Query 176
VWSMCTDVHEENRIPSVESLKSVRP-DDSSIQVVLVDRRADFDLGMLENYASSFLSSSSD
234

VW++CTD+HE RIPSV++LKSV P SS++VVLVDRR+D L L+N S
SS

Sbjct 154
VWTVCTDLHENGRIPSVDTLKSVNPCIISSLEVVLVDRRSDPSLRDLQNRVHSISCSSIT
213

Query 235
MKDVINQLAKLVSSRMGGTTS-NEENLLPRWKESSEAIKSSAGSIVLHLGKLPIGLCKHR
293

DV++QL+KL+ +RMGG+ S E+N W++ S +K GS+V+ +G L
+GLC+HR

Sbjct 214
TTDVVDQLSKLICNRMGGSASIGEDNFFSIWRDCSNDLKDCLGSVVIPIGSLSVGLCRHR
273

Query 294
SLLFKMLADKVNIPCRLVKGCKYCKAEDASSCVVRFGLEREYLVDLFGAPGQLSDPDSFV
353

++LFK+LAD +++PCR+ KGCKYCK +DA+SC+VRFGLEREYLVDL G PG
LS+PDS +

Sbjct 274
AVLFKVLADAIDLPCRIAKGCKYCKRDDAASCLVRFGLEREYLVDLIGKPGSLSEPDSLL
333

Query 354
NGPYSLSVPSPLRPPKFRSLEITSNFSSVAKQYFSDCHSLNLLFSDASTGASN---GAAV 410

NGP S+S SPLR P+ + + T +F S+AKQYFSD SL L+F ++S SN G ++

Sbjct 334
NGPSSISFSSPLRFPRLKPADPTIDFRSLAKQYFSDNLSLELVFDNSSAAVSNEDSGFSI 393

Query 411
AVDQMYSKKHDAGDGIANSWVPVKGQAIANSDIILPEAPREVLPLMSPSNLTA------- 463

S++ DA N+ P+ + +N LP P++ P +LT

Sbjct 394
------SEQFDAKCKDRNNTRPISTE--SNRSSHLPLHPQDSTPGTHEQDLTTVGKYPPP 445

Query 464 -------------------DKKKEFQLIEGNQYL-RSTVSDLSLAVDDLIIPWSELVLK
502
D + + +EG+Q + + +L+L ++DL IPWS+LVL+
Sbjct 446
IKHKRPVGIPTPLAFPNTNDDMIEGKRFVEGSQLIPNKSTRELTLDMEDLDIPWSDLVLR
505

Query 503
EKIGAGSFGTVHRADWHGSDVAVKILMEQDYHLDRFKEFMREVAIMKSLRHPNIVLFMG
A 562
EKIG+GSFGTVHRA+W+GSDVAVKILMEQD+H +RFKEF+REVAIMK
LRHPNIVLFMGA
Sbjct 506
EKIGSGSFGTVHRAEWNGSDVAVKILMEQDFHAERFKEFLREVAIMKRLRHPNIVLFMGA
565

Query 563
VTEPPNLSIVTEYLSRGSLYKLLHRSGAREVLDERRRLNMAFDVAKGMNYLHRRSPPIVH
622

VT+PPNLSIVTEYLSRGSLY+LLHRSGA+EVLDERRRL+MA+DVAKGMNYLH+R+PPIVH
Sbjct 566
VTQPPNLSIVTEYLSRGSLYRLLHRSGAKEVLDERRRLSMAYDVAKGMNYLHKRNPPIVH
625

Query 623
RDLKSPNLLVDKKYTVKVCDFGLSRLKANTYLSSKSLAGTPEWMAPEVLRDEPSNEKSD
V 682
RDLKSPNLLVDKKYTVKVCDFGLSRLKANT+LSSKS
AGTPEWMAPEVLRDEPSNEKSD+
Sbjct 626
RDLKSPNLLVDKKYTVKVCDFGLSRLKANTFLSSKSAAGTPEWMAPEVLRDEPSNEKSDI
685

Query 683
YSFAVILWELMTLQQPWCNLNPAQVVAAVGFKGRRLEIPKELNPQVAALIESCWANEPWR
742
YSF VILWEL TLQQPW
NLNPAQVVAAVGFKG+RLEIP+++NPQVAALIE+CWA+EPW+
Sbjct 686
YSFGVILWELATLQQPWVNLNPAQVVAAVGFKGKRLEIPRDVNPQVAALIEACWASEPWK
745

Query 743 RPSFANIMETLRPLINKVPVPQ 764

RPSFA+IM++LRPL+ K P PQ
Sbjct 746 RPSFASIMDSLRPLL-KPPTPQ 766

>unnamed protein product, partial [Vitis vinifera]
Sequence ID: CBI30245.3 Length: 745
Range 1: 3 to 740

Score:867 bits(2239), Expect:0.0,
Method:Compositional matrix adjust.,
Identities:458/767(60%), Positives:552/767(71%), Gaps:35/767(4%)

Query 9
GRRTSYSLLSQFPDD---AAAAGASPAVLQRQSSGGSSYGAGSSVSASSDYPFHLPPAVA 65
G+R++YSLLSQFPDD AAG P L SG S G G F A
Sbjct 3
GKRSNYSLLSQFPDDQFVGGAAGNQPP-LYESLSGEKSKGKG----------FDWDELRA 51

Query 66
AAGGGGGTPSPCKSWAQQAEETYQLQLALALRLCADAACAADPGFLDPGDSGGSKMGG
GG 125
A G G S KSWAQQ EE+YQLQLALALRL ++A CA DP FLDP +
Sbjct 52
KAVTGTGDSSSSKSWAQQTEESYQLQLALALRLSSEATCADDPNFLDPVPDDSASRSLSS
111

Query 126
GGSGSGRAFPLAPPSPTAEALSHRFWVNGSLSYSNTIPDGFYLIQGMDPFVWSMCTDVHE
185
GS + EA+SHRFWV+G LSY + +PDGFYLI GMDP+VW++C D+
E
Sbjct 112
SGS-------------SVEAMSHRFWVSGCLSYFDKVPDGFYLIHGMDPYVWTVCNDLRE 158

Query 186
ENRIPSVESLKSVRPD-DSSIQVVLVDRRADFDLGMLENYASSFLSSSSDMKDVINQLAK
244
RIPS+ESLK P DS I+VVL+DRR D L L+N S
K+V++QLAK
Sbjct 159
NGRIPSIESLKHAEPSADSPIEVVLIDRRTDPTLKELQNKVHGISCSCMTTKEVVDQLAK
218

Query 245
LVSSRMGGTTSN-EENLLPRWKESSEAIKSSAGSIVLHLGKLPIGLCKHRSLLFKMLADK
303
LV + MGG S E++ + W+E S+ K GSIV+ +G L GLC+HR+LLFK+LAD
Sbjct 219
LVCNCMGGAASTGEDDFVSIWRECSDDQKDCLGSIVVPIGSLSFGLCRHRALLFKVLADT
278

Query 304
VNIPCRLVKGCKYCKAEDASSCVVRFGLEREYLVDLFGAPGQLSDPDSFVNGPYSLSVPS
363
+++ CR+ KGCKYC +DASSC+VR G +RE+LVDL G PG L +PDS +NGP S+S+ S
Sbjct 279
IDLRCRIAKGCKYCTRDDASSCLVRVGPDREFLVDLVGKPGCLCEPDSLLNGPASISISS
338

Query 364
PLRPPKFRSLEITSNFSSVAKQYFSDCHSLNLLFSDASTGASNGAAVAVDQMYSKKHDAG
423
PLR P+ + +E +F S+AKQYFS+C SLNL+F D S G A D MY KK D
Sbjct 339
PLRFPRSKPVETNIDFRSLAKQYFSECQSLNLVFEDTSVGVIVDEADGGDSMYPKKFDRK
398

Query 424
DGIANSWVPVKGQAIANSDIILPEAPREVLPLMSPSNLTADKKKEFQLIEGNQ-YLRSTV
482
VP+ + +P P+ P +S +L D K+ + +G Q Y
Sbjct 399
CTDRTHLVPISRNRGETPQLPMP--PKVAWPTLS--DLRGDTIKDMRFTDGGQLYPNKPC
454

Query 483
SDLSLAVDDLIIPWSELVLKEKIGAGSFGTVHRADWHGSDVAVKILMEQDYHLDRFKEFM
542
+LSL V+DL IPWS+LVLKE+IGAGSFGTVHRADW+GSDVAVK+LMEQD+H
+RFKEF+
Sbjct 455
KELSLDVEDLDIPWSDLVLKERIGAGSFGTVHRADWNGSDVAVKVLMEQDFHAERFKEF
L 514

Query 543
REVAIMKSLRHPNIVLFMGAVTEPPNLSIVTEYLSRGSLYKLLHRSGAREVLDERRRLNM

602
REV+IMK LRHPNIVLFMGAVT+PPNLSIVTEYLSRGSLY+LLH+
GARE+LDERRRL+M
Sbjct 515
REVSIMKRLRHPNIVLFMGAVTQPPNLSIVTEYLSRGSLYRLLHKPGAREMLDERRRLSM
574

Query 603
AFDVAKGMNYLHRRSPPIVHRDLKSPNLLVDKKYTVKVCDFGLSRLKANTYLSSKSLAGT
662
A+DVAKGMNYLH+R+PPIVHRDLKSPNLLVDKKYTVKVCDFGLSR
KANT+LSSKS AGT
Sbjct 575
AYDVAKGMNYLHKRNPPIVHRDLKSPNLLVDKKYTVKVCDFGLSRFKANTFLSSKSAAG
T 634

Query 663
PEWMAPEVLRDEPSNEKSDVYSFAVILWELMTLQQPWCNLNPAQVVAAVGFKGRRLEIPK
722
PEWMAPEVLRDE SNEKSD+YSF +ILWEL TLQQPW
NLNPAQVVAAVGFKG+RLEIP+
Sbjct 635
PEWMAPEVLRDEASNEKSDIYSFGIILWELATLQQPWSNLNPAQVVAAVGFKGKRLEIPR
694

Query 723 ELNPQVAALIESCWANEPWRRPSFANIMETLRPLINKVPVPQLIRSD 769
+LNPQVA++IE+CWANEPW+RPSF NIME+L+PLI K P PQ +R+D
Sbjct 695 DLNPQVASIIEACWANEPWKRPSFFNIMESLKPLI-KPPTPQPVRAD 740

>protein kinase family protein [Rhynchospora pubera]
Sequence ID: KAJ4775647.1 Length: 806
Range 1: 1 to 731

Score:866 bits(2237), Expect:0.0,
Method:Compositional matrix adjust.,
Identities:479/780(61%), Positives:562/780(72%), Gaps:72/780(9%)

Query 1
MELPAAGGGRRTSYSLLSQFPDD-------AAAAGASPAVLQRQSSGGSSYGAGSSVSAS 53
ME+P G +YS+L+Q DD P LQRQSSG S SS+S S
Sbjct 1

MEIPKRGTN---TYSVLNQLSDDYDPSLPPLPLPLPLPLQLQRQSSGSSY--GDSSLSLS 55

Query 54
SDYPFHLPPAVAAAGGGGGTP---------SPCKSWAQQAEETYQLQLALALRLCADAAC 104
D F PP + A G P
KSWAQQAEETYQLQLALALRLC++AA
Sbjct 56
GDCCFFAPPPLDAITGAKSEPITGGSGSGSGSGKSWAQQAEETYQLQLALALRLCSEAAS
115

Query 105
AADPGFLDPGDSGGSKMGGGGGGSGSGRAFPLAPPSPTAEALSHRFWVNGSLSYSNTIPD
164
+DPG ++ GD+ + P+ + +AEALSHRFWVNGSLSY ++I D
Sbjct 116 VSDPGLMESGDASTA---------------PVTSSAVSAEALSHRFWVNGSLSYIDSIQD
160

Query 165
GFYLIQGMDPFVWSMCTDVHEENRIPSVESLKSVRPDDSSIQVVLVDRRADFDLGMLENY
224
GFY+I+GMDPFVWS+CTDV EE+R+P++ SLK+V DSSI+VVL D++ D L L
N
Sbjct 161
GFYVIRGMDPFVWSLCTDVQEESRMPTLVSLKNVDWSDSSIEVVLFDKKIDSYLRQLHNS
220

Query 225
ASSFLS---SSSDMKDVINQLAKLVSSRMGGTTSN---EENLLPRWKESSEAIKSSAGSI 278
S S +++ KD++ QLAKLVSSRMGG N EE LL RW++S+ + S
S+
Sbjct 221
VVSTFSSFSATASTKDMVEQLAKLVSSRMGGAALNLKDEEALLSRWRDSNSTARMSTHSV
280

Query 279
VLHLGKLPIGLCKHRSLLFKMLADKVNIPCRLVKGCKYCKAEDASSCVVRFGLEREYLVD
338
V+ LG+L +GLC HRSLLFKMLAD VN+PCR+VKGCKYC
+DA+SC+VRFGLEREYLVD
Sbjct 281
VIPLGQLSVGLCIHRSLLFKMLADSVNVPCRVVKGCKYCTRDDATSCLVRFGLEREYLVD
340

Query 339

LFGAPGQLSDPDSFVNGPYSLSVPSPLRPPKFRSLEITSNFSSVAKQYFSDCHSLNLLFS 398

L G PG LS+PDS +NGP S+ V SPL PP+F+S+E NF S+AKQYFS+C SLNL F

Sbjct 341 LIGDPGHLSEPDSQLNGPNSMFVSSPLHPPRFKSMERNGNFKSLAKQYFSECQSLNLSFK 400

Query 399 DASTGASNGAAVAVDQMYSKKHDAGDGIANSWVPVKGQAIANSDIILPEAP-REVLPLMS 457

D T + V+ DQ G + VP ++ S++ P+ E L ++

Sbjct 401 DTKTTSD---LVSSDQ-------TGAYV----VPAAAPSLPFSNLRPPDMKITESLARIN 446

Query 458 PSNLTADKKKEFQLIEGNQYLRSTVSDLSLAVDDLIIPWSELVLKEKIGAGSFGTVHRAD 517

PS T S D+ +VDD IPW EL+LKEKIGAGSFGTVHRAD

Sbjct 447 PSRTTNP---------------SPNRDIVFSVDDYTIPWRELILKEKIGAGSFGTVHRAD 491

Query 518 WHGSDVAVKILMEQDYHLDRFKEFMREVAIMKSLRHPNIVLFMGAVTEPPNLSIVTEYLS 577

WHGSDVAVKIL+EQD+H DR KEFMREVAIMKSLRHPNIVL MG VTEPPNLSIVTEYLS

Sbjct 492 WHGSDVAVKILIEQDFHADRLKEFMREVAIMKSLRHPNIVLLMGVVTEPPNLSIVTEYLS 551

Query 578 RGSLYKLLHRSGAREVLDERRRLNMAFDVAKGMNYLHRRSPPIVHRDLKSPNLLVDKKYT 637

RGSLYKLLH+S RE LDERRRL+MAFDVAKGMNYLHRR+PPIVHRDLKSPNLLVD+KYT

Sbjct 552 RGSLYKLLHKSTVREYLDERRRLHMAFDVAKGMNYLHRRNPPIVHRDLKSPNLLVDRKYT 611

Query 638 VKVCDFGLSRLKANTYLSSKSLAGTPEWMAPEVLRDEPSNEKSDVYSFAVILWELMTLQQ 697

VKVCDFGLSRLKA+TYLSSKSLAGTPEWMAPEVLRDE SNEKSDVYSF

VILWELMTLQQ
Sbjct 612
VKVCDFGLSRLKASTYLSSKSLAGTPEWMAPEVLRDELSNEKSDVYSFGVILWELMTLQ
Q 671

Query 698
PWCNLNPAQVVAAVGFKGRRLEIPKELNPQVAALIESCWANEPWRRPSFANIMETLRPLI
757
PW NLNPAQVVAAVGFKGRRLEIPKELNP+V
+LIESCWA+EPW+RPSFA+IME+L+PL+
Sbjct 672
PWSNLNPAQVVAAVGFKGRRLEIPKELNPRVTSLIESCWADEPWKRPSFASIMESLKPLL
731

>protein kinase family protein [Rhynchospora pubera]
Sequence ID: KAJ4776217.1 Length: 824
Range 1: 76 to 814

Score:865 bits(2236), Expect:0.0,
Method:Compositional matrix adjust.,
Identities:481/788(61%), Positives:565/788(71%), Gaps:76/788(9%)

Query 1
MELPAAGGGRRTSYSLLSQFPDD-------AAAAGASPAVLQRQSSGGSSYGAGSSVSAS 53
ME+P G +YS+L+Q DD P LQRQSSG S SS+S S
Sbjct 76
MEIPKRGTN---TYSVLNQLSDDYDPSLPPLPLPLPLPLQLQRQSSGSSY--GDSSLSLS 130

Query 54
SDYPFHLPPAVAAAGGGGGTP-------------SPCKSWAQQAEETYQLQLALALRLCA 100
D F PP + A G P
KSWAQQAEETYQLQLALALRLC+
Sbjct 131
GDCCFFAPPPLDAITGAKSEPITGGCEMGGGSGSGSGKSWAQQAEETYQLQLALALRLCS
190

Query 101
DAACAADPGFLDPGDSGGSKMGGGGGGSGSGRAFPLAPPSPTAEALSHRFWVNGSLSYS
N 160
+AA +DPG ++ GD+ + P+ + +AEALSHRFWVNGSLSY
+

Sbjct 191
EAASVSDPGLMESGDASTA---------------PVTSSAVSAEALSHRFWVNGSLSYID 235

Query 161
TIPDGFYLIQGMDPFVWSMCTDVHEENRIPSVESLKSVRPDDSSIQVVLVDRRADFDLGM
220
+I DGFY+I+GMDPFVWS+CTDV EE+R+P++ SLK+V DSSI+VVL D++ D L
Sbjct 236
SIQDGFYVIRGMDPFVWSLCTDVQEESRMPTLVSLKNVDWSDSSIEVVLFDKKIDSYLRQ
295

Query 221
LENYASSFLS---SSSDMKDVINQLAKLVSSRMGGTTSN---EENLLPRWKESSEAIKSS 274
L N S S +++ KD++ QLAKLVSSRMGG N EE LL RW++S+ +
S
Sbjct 296
LHNSVVSTFSSFSATASTKDMVEQLAKLVSSRMGGAAPNLKDEEALLSRWRDSNSTARMS
355

Query 275
AGSIVLHLGKLPIGLCKHRSLLFKMLADKVNIPCRLVKGCKYCKAEDASSCVVRFGLERE
334
S+V+ LG+L +GLC HRSLLFKMLAD VN+PCR+VKGCKYC
+DA+SC+VRFGLERE
Sbjct 356
THSVVIPLGQLSVGLCIHRSLLFKMLADSVNVPCRVVKGCKYCTRDDATSCLVRFGLERE
415

Query 335
YLVDLFGAPGQLSDPDSFVNGPYSLSVPSPLRPPKFRSLEITSNFSSVAKQYFSDCHSLN
394
YLVDL G PG L++PDS +NGP S+ V SPL PP+F+S+E NF S+AKQYFS+C SLN
Sbjct 416
YLVDLIGDPGHLTEPDSQLNGPNSMFVSSPLHPPRFKSMERNGNFKSLAKQYFSECQSLN
475

Query 395
LLFSDASTGASNGAAVAVDQMYSKKHDAGDGIANSWVPVKGQAIANSDIILPEAP-REVL
453
L F D T + V+ DQ G + VP ++ S++ P+ E L
Sbjct 476 LSFKDTKTTSD---LVSSDQ-------TGACV----VPAAAPSLPFSNLRPPDMKITESL
521

Query 454

PLMSPSNLTADKKKEFQLIEGNQYLRSTVSDLSLAVDDLIIPWSELVLKEKIGAGSFGTV
513
++PS T S D+ +VDDL IPW EL+LKEKIGAGSFGTV
Sbjct 522 ARINPSRTTNP---------------SPNRDIVFSVDDLTIPWRELILKEKIGAGSFGTV
566

Query 514
HRADWHGSDVAVKILMEQDYHLDRFKEFMREVAIMKSLRHPNIVLFMGAVTEPPNLSIVT
573
HRADWHGSDVAVKIL+EQD+H DR KEFMREV IMKSLRHPNIVLFMG
VTEPPNLSIVT
Sbjct 567
HRADWHGSDVAVKILIEQDFHADRLKEFMREVIIMKSLRHPNIVLFMGVVTEPPNLSIVT
626

Query 574
EYLSRGSLYKLLHRSGAREVLDERRRLNMAFDVAKGMNYLHRRSPPIVHRDLKSPNLLVD
633
EYLSRGSLYKLLH+S RE
LDERRRL+MAFDVAKGMNYLHRR+PPIVHRDLKSPNLLVD
Sbjct 627
EYLSRGSLYKLLHKSTVREYLDERRRLHMAFDVAKGMNYLHRRNPPIVHRDLKSPNLLV
D 686

Query 634
KKYTVKVCDFGLSRLKANTYLSSKSLAGTPEWMAPEVLRDEPSNEKSDVYSFAVILWEL
M 693
+KYTVKVCDFGLSRLKA+TYLSSKSLAGTPEWMAPEVLRDE
SNEKSDVYSF VILWELM
Sbjct 687
RKYTVKVCDFGLSRLKASTYLSSKSLAGTPEWMAPEVLRDELSNEKSDVYSFGVILWEL
M 746

Query 694
TLQQPWCNLNPAQVVAAVGFKGRRLEIPKELNPQVAALIESCWANEPWRRPSFANIMETL
753
TLQQPW NLNPAQVVAAVGFKGRRLEIPKELNP+V
+LIESCWA+EPW+RPSFA+IME+L
Sbjct 747
TLQQPWSNLNPAQVVAAVGFKGRRLEIPKELNPRVTSLIESCWADEPWKRPSFASIMESL
806

Query 754 RPLINKVP 761
+PL+ K P

Sbjct 807 KPLLVKSP 814

>serine/threonine-protein kinase CTR1-like isoform X2 [Panicum hallii]
Sequence ID: XP_025799085.1 Length: 801
>hypothetical protein PAHAL_1G251900 [Panicum hallii]
Sequence ID: PAN06278.1 Length: 801
Range 1: 94 to 797

Score:864 bits(2233), Expect:0.0,
Method:Compositional matrix adjust.,
Identities:447/746(60%), Positives:544/746(72%), Gaps:55/746(7%)

Query 33
VLQRQSSG---GSSYGAGSSVSASSDYPFHLPPAVAAAGGGGGTPSP-----CKSWAQQA 84
+LQRQSSG G G GSS +++ L A A G P KSWAQQA
Sbjct 94
LLQRQSSGSSVGGDDGEGSSTAST------LANAAAEYRDRGDADRPPSSSGGKSWAQQA
147

Query 85
EETYQLQLALALRLCADAACAADPGFLDPGDSGGSKMGGGGGGSGSGRAFPLAPPSPTA
E 144
EE Y LQLALALRLC++A+ AADP FLD + + +A P +
Sbjct 148
EEAYHLQLALALRLCSEASSAADPNFLD-----------SSSNAAADHLQHIASP----Q 192

Query 145
ALSHRFWVNGSLSYSNTIPDGFYLIQGMDPFVWSMCTDVHEENRIPSVESLKSVRPDDSS
204
+LS+RFWVNGSLSYS+ +PDGFYLIQGM+PF+W++C DVH+ R+PS+ESLK+V
P +SS
Sbjct 193
SLSYRFWVNGSLSYSDKVPDGFYLIQGMNPFIWTLCNDVHDGGRVPSIESLKAVNPTESS
252

Query 205
IQVVLVDRRADFDLGMLENYASSFLSSSSDMKDVINQLAKLVSSRMGGTT--SNEENLLP
262
I+ V+VD+ AD++L L + A + +D K++ +LA +VS++MGG+ + E L P
Sbjct 253
IEAVIVDKVADYELRQLISMAIDVSRNRADSKEIATRLAGVVSAKMGGSVAATEEHGLGP

312

Query 263
RWKESSEAIKSSAGSIVLHLGKLPIGLCKHRSLLFKMLADKVNIPCRLVKGCKYCKAEDA
322
RW+E+ +K S+GS+VL +GKL IG C HR+LLFK LAD +N+ CR+VKGCKYCKA
A
Sbjct 313
RWRETVGFLKISSGSVVLPIGKLSIGFCCHRALLFKTLADSINLLCRIVKGCKYCKAGAA
372

Query 323
SSCVVRFGLEREYLVDLFGAPGQLSDPDSFVNGPYSLSVPSPLRPPKFRSLEITSNFSSV
382
+SC+VRF +REYL+DL G PG LS+PDS +NG S+SV SPLRPPK S++I NF S+
Sbjct 373
ASCLVRFDHDREYLIDLIGNPGFLSEPDSLLNGLSSISVSSPLRPPKHNSVDIADNFKSL
432

Query 383
AKQYFSDCHSLNLLFSDASTGASNGAAVAVDQMYSKKHDAGDGIANSWVPVKGQAIANS
- 441
AKQYF DC +LNL+FSD + + AV + + H ++ +K A S
Sbjct 433
AKQYFLDCQALNLMFSDPAAVINLDEAVGSNLGPNSSHGTNSDCQATFPHLKAGAQLGSQ
492

Query 442
--DIILPEAPREVLPLMSPSNLTADKKKEFQLIEGNQYLRSTVSDLSLAVDDLIIPWSEL 499
+ I+ R P + S L+ SD+SL ++DLIIPWSEL
Sbjct 493 DENFIM----RRSFPEDTQSGLS-----------------DPFSDMSLDIEDLIIPWSEL 531

Query 500
VLKEKIGAGSFGTVHRADWHGSDVAVKILMEQDYHLDRFKEFMREVAIMKSLRHPNIVLF
559
VLKEKIGAGSFGTVHRADW+GSDVAVKILMEQD+H +R
KEF+REVAIM+SLRHPNIVL
Sbjct 532
VLKEKIGAGSFGTVHRADWNGSDVAVKILMEQDFHPERLKEFLREVAIMRSLRHPNIVLL
591

Query 560
MGAVTEPPNLSIVTEYLSRGSLYKLLHRSGAREVLDERRRLNMAFDVAKGMNYLHRRSPP
619

MGAVT+PPNLSIVTEYLSRGSLY+LLHR ARE LDERRRL+MAFDVAKGMNYLH+R+PP
Sbjct 592 MGAVTQPPNLSIVTEYLSRGSLYRLLHRHSARENLDERRRLSMAFDVAKGMNYLHKRNPP 651

Query 620 IVHRDLKSPNLLVDKKYTVKVCDFGLSRLKANTYLSSKSLAGTPEWMAPEVLRDEPSNEK 679
IVHRDLKSPNLLVDKKYTVKVCDFGLSRLKANT+LSSK+ AGTPEWMAPEVLRDEPSNEK
Sbjct 652 IVHRDLKSPNLLVDKKYTVKVCDFGLSRLKANTFLSSKTAAGTPEWMAPEVLRDEPSNEK 711

Query 680 SDVYSFAVILWELMTLQQPWCNLNPAQVVAAVGFKGRRLEIPKELNPQVAALIESCWANE 739
SDVYSF VILWELMTLQQPW NLNPAQVVAAVGFKGRRLEIP ++P+VAALI+SCW E
Sbjct 712 SDVYSFGVILWELMTLQQPWSNLNPAQVVAAVGFKGRRLEIPSSIDPKVAALIDSCWVRE 771

Query 740 PWRRPSFANIMETLRPLINKVPVPQL 765
PWRRPSFA+IME+L+PLI +P +L
Sbjct 772 PWRRPSFASIMESLKPLIKTLPPNEL 797

>serine/threonine-protein kinase CTR1-like [Ipomoea triloba]
Sequence ID: XP_031131407.1 Length: 850
Range 1: 139 to 845

Score:863 bits(2231), Expect:0.0,
Method:Compositional matrix adjust.,
Identities:438/725(60%), Positives:532/725(73%), Gaps:51/725(7%)

Query 78 KSWAQQAEETYQLQLALALRLCADAACAADPGFLDP-GDSGGSKMGGGGGGSGSGRAFPL 136
KSWAQQ EE+YQ+QLALALRL ++A CA DP FLDP D S+
Sbjct 139

KSWAQQTEESYQMQLALALRLSSEATCAEDPNFLDPVPDEAASRSSAL------------ 186

Query 137
APPSPTAEALSHRFWVNGSLSYSNTIPDGFYLIQGMDPFVWSMCTDVHEENRIPSVESLK
196
+ +AEA+SHRFWVNG LSY + IPDGFYLI GMDP+VW +CTD+ E
RIPS+ESLK
Sbjct 187
---TVSAEAMSHRFWVNGCLSYFDKIPDGFYLIHGMDPYVWIVCTDLQESGRIPSLESLK
243

Query 197
SVRPDD-SSIQVVLVDRRADFDLGMLENYASSFLSSSSDMKDVINQLAKLVSSRMGGTTS
255
+ P S++V L+DRR D L L+N + S K+V++QLAKLV + MGG
S
Sbjct 244
AADPSILPSVEVTLIDRRTDPSLKELQNRIHNLSPSCITTKEVVDQLAKLVCNHMGGAAS
303

Query 256
-NEENLLPRWKESSEAIKSSAGSIVLHLGKLPIGLCKHRSLLFKMLADKVNIPCRLVKGC
314
E +L+P WKE + +K GSIV+ +G L +GLC+HRSLLFK+LAD +++PCR+
KGC
Sbjct 304
AGETDLVPIWKECRDDLKDCLGSIVIPIGNLSVGLCRHRSLLFKVLADTIDLPCRIAKGC
363

Query 315
KYCKAEDASSCVVRFGLEREYLVDLFGAPGQLSDPDSFVNGPYSLSVPSPLRPPKFRSLE
374
+YC +DASSC+VRFGL+REYLVDL G PG L +PDS +NGP S+++ SPLR P+F+ +E
Sbjct 364
RYCNRDDASSCLVRFGLDREYLVDLVGMPGCLCEPDSMLNGPSSIAISSPLRFPRFKQVE
423

Query 375
ITSNFSSVAKQYFSDCHSLNLLFSDASTGASNGAAVAVDQMYSKKHDAGDGIANSWVPVK
434
+F +AKQYFSDC SLNL+F D+STG + V +Y K+ D+G N ++P
Sbjct 424
PRIDFKLLAKQYFSDCQSLNLVFIDSSTGTTVSGDAGV-PVYPKQADSGGMDRNCFIPSS
482

Query 435 GQAIANSDIILP---------------------------EAPREV-LPLMSPSNLTADK 465
S LP R+V LP++ P + D
Sbjct 483
SNQEEISRFPLPPTNVQRMGGGLESQRAGMYGPSNIINSMNVGRDVQLPIIRP-DPRLDS
541

Query 466
KKEFQLIEGNQYLRSTVSDLSLAVDDLIIPWSELVLKEKIGAGSFGTVHRADWHGSDVAV
525
K+ + +G Q +L++ V+DL
IPW++LVLKE+IGAGSFGTVHRADWHGSDVAV
Sbjct 542
TKDTRFTDGGQLALRKPKELAIDVEDLDIPWTDLVLKERIGAGSFGTVHRADWHGSDVAV
601

Query 526
KILMEQDYHLDRFKEFMREVAIMKSLRHPNIVLFMGAVTEPPNLSIVTEYLSRGSLYKLL
585
KILMEQD+H +R+ EF+REVAIMK
LRHPNIVLFMGAVT+PPNLSIVTEYLSRGSLY+LL
Sbjct 602
KILMEQDFHPERYHEFLREVAIMKRLRHPNIVLFMGAVTQPPNLSIVTEYLSRGSLYRLL
661

Query 586
HRSGAREVLDERRRLNMAFDVAKGMNYLHRRSPPIVHRDLKSPNLLVDKKYTVKVCDFG
L 645
H+ GARE LDE+RRL
MA+DVAKGMNYLH+R+PPIVHRDLKSPNLLVDKKYTVKVCDFGL
Sbjct 662
HKHGAREALDEKRRLCMAYDVAKGMNYLHKRNPPIVHRDLKSPNLLVDKKYTVKVCDF
GL 721

Query 646
SRLKANTYLSSKSLAGTPEWMAPEVLRDEPSNEKSDVYSFAVILWELMTLQQPWCNLNPA
705
SRLKANT+LSSKS AGTPEWMAPEVLRDEPSNEKSD+YSF VILWEL
TLQQPW NLNPA
Sbjct 722
SRLKANTFLSSKSAAGTPEWMAPEVLRDEPSNEKSDLYSFGVILWELATLQQPWGNLNPA
781

Query 706

QVVAAVGFKGRRLEIPKELNPQVAALIESCWANEPWRRPSFANIMETLRPLINKVPV-PQ
764
QVVAAVGFKG+RLEIP +LNPQVAA+IE+CWANEPW+RPSF+ IM+ LRPLI
K+PV PQ
Sbjct 782
QVVAAVGFKGKRLEIPHDLNPQVAAIIEACWANEPWKRPSFSTIMDLLRPLI-KLPVTPQ
840

Query 765 LIRSD 769
R+D
Sbjct 841 PGRTD 845

>serine/threonine-protein kinase CTR1-like isoform X2 [Pistacia vera]
Sequence ID: XP_031261290.1 Length: 832
Range 1: 1 to 813

Score:863 bits(2229), Expect:0.0,
Method:Compositional matrix adjust.,
Identities:462/836(55%), Positives:575/836(68%), Gaps:102/836(12%)

Query 1
MELPAAGGGRRTSYSLLSQFPDDAAAAGASPAVLQRQSSGGSSYGAGS-----SVSASSD 55
ME+P GRR++YSLLSQ+PDD +SP G S+ + S+ D
Sbjct 1
MEMP----GRRSNYSLLSQYPDDQLPVASSPFYESHSGDGKSNINSSKPKLDWESSSGGD
56

Query 56 YPF---------------------------------HLPPAVAAA-------------- 67
+ F + P +++A
Sbjct 57
HKFSQQSNRIGNLYTSSMGLQRQSSGSSFGESSMSGDYYAPTLSSAAANEIDSFGDVYKV
116

Query 68
GGGGGTPSP-------CKSWAQQAEETYQLQLALALRLCADAACAADPGFLDPGDSGGSK
120
GGG P KSWAQQ EE+YQLQLALALRL ++A CA DP FLDP
+
Sbjct 117
GGGDYKAKPPKEGSSSGKSWAQQTEESYQLQLALALRLSSEATCADDPNFLDPVPDESAL
176

Query 121
MGGGGGGSGSGRAFPLAPPSPTAEALSHRFWVNGSLSYSNTIPDGFYLIQGMDPFVWSMC
180
G +AEA+SHRFWV G LSY + +PDGFYL+
G+DP+VW++C
Sbjct 177
RSGSAS---------------SAEAVSHRFWVTGCLSYFDKVPDGFYLVHGVDPYVWTVC 221

Query 181
TDVHEENRIPSVESLKSVRPD-DSSIQVVLVDRRADFDLGMLENYASSFLSSSSDMKDVI
239
TD++E RIPS+ESL+SV P D+SI+V+L+DRR+D L L+N + S
K+V+
Sbjct 222
TDLNENGRIPSIESLRSVDPSVDTSIEVLLIDRRSDPSLKELQNRVMNISCSCITTKEVV 281

Query 240
NQLAKLVSSRMGGTTS-NEENLLPRWKESSEAIKSSAGSIVLHLGKLPIGLCKHRSLLFK
298
+QLAKLV +RMGG+ + E++ + W+E S+ IK GS+V+ +G L
+GLC+HR+LLFK
Sbjct 282
DQLAKLVCNRMGGSAAMAEDDFVSIWRECSDDIKDCLGSVVVPMGSLSVGLCRHRTLLF
K 341

Query 299
MLADKVNIPCRLVKGCKYCKAEDASSCVVRFGLEREYLVDLFGAPGQLSDPDSFVNGPYS
358
+LAD +++PCR+ KGCKYCK +DASSC+VRFGL+REYLVDL G PGQL +PDS
+NGP S
Sbjct 342
LLADAIDLPCRIAKGCKYCKRDDASSCLVRFGLDREYLVDLIGKPGQLCEPDSLLNGPSS
401

Query 359
LSVPSPLRPPKFRSLEITSNFSSVAKQYFSDCHSLNLLFSDASTGASNGAAVAVDQMYSK
418
+++ SPLR P+ +S E T +F +AKQYFSDC S NL+F D+S G++ MY K
Sbjct 402
ITIASPLRFPRLKSAEPTMDFRLLAKQYFSDCQSHNLVFDDSSAGSAVEEEDNKFSMYPK
461

Query 419 KHDAGDGIANSWVPVKGQAIANSDIILPEAPREV----------------LPLMSPSNLT

462

+ D G + V +I S + LP P+ V P ++ ++

Sbjct 462

QFDKM-GTERNLVQF-SSSINESQLALP--PKGVRPSAHDRDSELFKSYGQPCLALTDQI

517

Query 463

ADKKKEFQLIEGNQYLRSTVS-DLSLAVDDLIIPWSELVLKEKIGAGSFGTVHRADWHGS

521

D K+ + EG Q + + S DLSL V++L

IPW++LVLKE+IGAGSFGTVHRADWHGS

Sbjct 518

VDTAKDLRFPEGAQLIPTKPSKDLSLYVEELDIPWNDLVLKERIGAGSFGTVHRADWHGS

577

Query 522

DVAVKILMEQDYHLDRFKEFMREVAIMKSLRHPNIVLFMGAVTEPPNLSIVTEYLSRGSL

581

DVAVKILMEQD+H +RF+EF+REVAIMK

LRHPNIVLFMGAVT+PPNLSIVTEYLSRGSL

Sbjct 578

DVAVKILMEQDFHGERFQEFLREVAIMKRLRHPNIVLFMGAVTQPPNLSIVTEYLSRGSL

637

Query 582

YKLLHRSGAREVLDERRRLNMAFDVAKGMNYLHRRSPPIVHRDLKSPNLLVDKKYTVKV

C 641

Y+LLH+

GAREVLDERRRLNMA+DVAKGMNYLHRR+PPIVHRDLKSPNLLVDKKYTVKVC

Sbjct 638

YRLLHKPGAREVLDERRRLNMAYDVAKGMNYLHRRNPPIVHRDLKSPNLLVDKKYTVK

VC 697

Query 642

DFGLSRLKANTYLSSKSLAGTPEWMAPEVLRDEPSNEKSDVYSFAVILWELMTLQQPWC

N 701

DFGLSRLKANT+LSSKS AGTPEWMAPEVL DEPSNEKSDVYSF VILWEL

TLQQPW N

Sbjct 698

DFGLSRLKANTFLSSKSAAGTPEWMAPEVLCDEPSNEKSDVYSFGVILWELATLQQPWGN

757

Query 702

LNPAQVVAAVGFKGRRLEIPKELNPQVAALIESCWANEPWRRPSFANIMETLRPLI 757

LNPAQVVAAVGFKG+RLEIP++LNP+VA++IE+CWANEPW+RPSF+ IM+
LRPLI
Sbjct 758
LNPAQVVAAVGFKGKRLEIPRDLNPRVASIIEACWANEPWKRPSFSTIMDLLRPLI 813

>hypothetical protein ZIOFF_014783 [Zingiber officinale]
Sequence ID: KAG6524839.1 Length: 802
Range 1: 1 to 787

Score:862 bits(2228), Expect:0.0,
Method:Compositional matrix adjust.,
Identities:480/816(59%), Positives:565/816(69%), Gaps:108/816(13%)

Query 1 MELPAAGGGRRTSYSLLSQFPDDA-----------------------AAAGASPAVLQRQ
37
ME+P GRR+SYSLL Q PD+A A++ A+P LQRQ
Sbjct 1
MEMP----GRRSSYSLLGQNPDEATPPLFDTPPSDKARAARFEWPINASSVAAPPPLQRQ 56

Query 38
SSGGS--------SYGAGSSVSASSDYPFHLPPAVAAAGGGGGTPSPC------KSWAQQ 83
SSG S Y +++S+S+ P G G + KSWAQQ
Sbjct 57
SSGSSYGGSSFSGDYYLPATISSSNVDSEGFNPLAGGEGRGQDGAAAGLSSSSAKSWAQQ
116

Query 84
AEETYQLQLALALRLCADAACAADPGFLDPGDSGGSKMGGGGGGSGSGRAFPLAPPSPT
A 143
AEETYQLQLALALRLC++AACA DP FLD D + P
Sbjct 117 AEETYQLQLALALRLCSEAACAEDPNFLDAADQM------------------VLPERAAP
158

Query 144
EALSHRFWVNGSLSYSNTIPDGFYLIQGMDPFVWSMCTDVHEENRIPSVESLKSVRPDDS
203
+LSHRFWVNG LSY + IPDGFYLIQGMDPFVW++CTDV EENRIPS+ESLK+V
P+DS
Sbjct 159
TSLSHRFWVNGCLSYHDKIPDGFYLIQGMDPFVWTLCTDVEEENRIPSIESLKTVHPNDS
218

Query 204
SIQVVLVDRRADFDLGMLENYASSFLSSSSDMKDVINQLAKLVSSRMGGTTSN-EENLLP
262
SI+VVL+DR+ D DL LE + S + KDV+ QLAKLV +RMGG N E+ LL
Sbjct 219
SIEVVLIDRQHDPDLRHLETIVTGLSCSCATAKDVVEQLAKLVCTRMGGIAFNGEDALLH
278

Query 263
RWKESSEAIKSSAGSIVLHLGKLPIGLCKHRSLLFKMLADKVNIPCRLVKGCKYCKAEDA
322
WKE E IK+S+ SIVL +GKL GLC+HR+LLFKMLAD +N+PCR+ KGCKYCK
+DA
Sbjct 279
CWKECGETIKASSCSIVLPMGKLSFGLCRHRALLFKMLADTINLPCRVAKGCKYCKTDDA
338

Query 323
SSCVVRFGLEREYLVDLFGAPGQLSDPDSFVNGPYSLSVPSPLRPPKFRSLEITSNFSSV
382
SSC+VRFGLEREYLVDL PG L +PDS +NGPYS+S+ SPLRPPK +S EI NF
+
Sbjct 339
SSCLVRFGLEREYLVDLIKNPGNLCEPDSLLNGPYSVSIASPLRPPKVKSSEINVNFRIL
398

Query 383
AKQYFSDCHSLNLLFSDASTGASNGAAVAVDQMYSKKHDAG--------------DG-IA 427
AKQYF DC SLNL+F DAS G S A+D SK D G DG I
Sbjct 399
AKQYFQDCQSLNLIFKDASAGTSIAEGDAIDPSSSKPSDGGTESLQPCIQKVTWPDGNIV
458

Query 428
NS---WVPVKGQAIANSDIILPEAPREVLPL----------MSPSNLTADKKKEFQLIEG 474
N+ + P++ N D++ RE+ P+ +P AD K +F+L E
Sbjct 459
NANKMFNPLRNAR--NPDLV----ERELGPMKLTAQDGCGNTAPCEPNADMK-DFKLTED
511

Query 475
NQYLRSTVS-DLSLAVDDLIIPWSELVLKEKIGAGSFGTVHRADWHGSDVAVKILMEQDY
533

++++ + S D+ LA DDL IPW
EL+LKE+IGAGSFGTVHRA+WHGS+VAVKILMEQD+
Sbjct 512
SKHIGNRPSNDIRLAEDDLNIPWRELILKERIGAGSFGTVHRAEWHGSEVAVKILMEQDF
571

Query 534
HLDRFKEFMREVAIMKSLRHPNIVLFMGAVTEPPNLSIVTEYLSRGSLYKLLHRSGAREV
593
H R
+EF+REVAIMKSLRHPNIVLFMGAVTEPPNLSIVTEYLSRGSLY+LLH++GA+E
Sbjct 572
HPGRLREFLREVAIMKSLRHPNIVLFMGAVTEPPNLSIVTEYLSRGSLYRLLHKNGAKET
631

Query 594
LDERRRLNMAFDVAKGMNYLHRRSPPIVHRDLKSPNLLVDKKYTVK------------VC 641
++ERRRL+MAFDVAKGMNYLH+R+PPIVHRDLKSPNLLVDKKYTVK
VC
Sbjct 632
IEERRRLSMAFDVAKGMNYLHKRNPPIVHRDLKSPNLLVDKKYTVKLKSDSAHFAVRTVC
691

Query 642
DFGLSRLKANTYLSSKSLAGTPEWMAPEVLRDEPSNEKSDVYSFAVILWELMTLQQPWC
N 701
DFGLSRLKANT+LSSKSLAGTPEWMAPEVLRDEPSNEKSD+YSF
VILWELMTLQQPW N
Sbjct 692
DFGLSRLKANTFLSSKSLAGTPEWMAPEVLRDEPSNEKSDIYSFGVILWELMTLQQPWSN
751

Query 702 LNPAQVVAAVGFKGRRLEIPKELNPQVAALIESCWA 737
LNPAQVVAAVGFK RR EIP +N VAA+IESCWA
Sbjct 752 LNPAQVVAAVGFKARRPEIPSTVNRHVAAIIESCWA 787

>serine/threonine-protein kinase CTR1-like isoform X1 [Ipomoea batatas]
Sequence ID: GMC49546.1 Length: 839
Range 1: 139 to 834

Score:860 bits(2222), Expect:0.0,

Method:Compositional matrix adjust.,
Identities:437/718(61%), Positives:534/718(74%), Gaps:48/718(6%)

Query 78
KSWAQQAEETYQLQLALALRLCADAACAADPGFLDP-GDSGGSKMGGGGGGSGSGRAF
PL 136
KSWAQQ EE+YQ+QLALALRL ++A CA DP FLDP D S+
Sbjct 139
KSWAQQTEESYQMQLALALRLSSEATCAEDPNFLDPVPDEAASRSSAL------------ 186

Query 137
APPSPTAEALSHRFWVNGSLSYSNTIPDGFYLIQGMDPFVWSMCTDVHEENRIPSVESLK
196
+ +AEA+SHRFWVNG LSY + IPDGFYLI GMDP+VW +C+D+ E
RIPS+ESLK
Sbjct 187
---TVSAEAMSHRFWVNGCLSYFDKIPDGFYLIHGMDPYVWIVCSDLQESGRIPSLESLK
243

Query 197
SVRPDD-SSIQVVLVDRRADFDLGMLENYASSFLSSSSDMKDVINQLAKLVSSRMGGTTS
255
+ P S++V L+DRR D L L+N + S K+V++QLAKLV + MGG
S
Sbjct 244
AADPSILPSVEVTLIDRRTDPSLKELQNRIHNLSPSCITTKEVVDQLAKLVCNHMGGAAS
303

Query 256
-NEENLLPRWKESSEAIKSSAGSIVLHLGKLPIGLCKHRSLLFKMLADKVNIPCRLVKGC
314
E +L+P WKE + +K GSIV+ +G L +GLC+HRSLLFK+LAD +++PCR+
KGC
Sbjct 304
AGETDLIPIWKECRDDLKDCLGSIVIPIGNLSVGLCRHRSLLFKVLADTIDLPCRIAKGC
363

Query 315
KYCKAEDASSCVVRFGLEREYLVDLFGAPGQLSDPDSFVNGPYSLSVPSPLRPPKFRSLE
374
+YC +DASSC+VRFGL+REYLVDL G PG L +PDS +NGP S+++ SPLR P+F+ +E
Sbjct 364
RYCNRDDASSCLVRFGLDREYLVDLVGMPGYLCEPDSMLNGPSSIAISSPLRFPRFKQVE
423

Query 375
ITSNFSSVAKQYFSDCHSLNLLFSDASTGASNGAAVAVDQMYSKKHDA------------ 422
+F +AKQYFSDC SLNL+F D+STG + V +Y K+ D+
Sbjct 424
PRIDFKLLAKQYFSDCQSLNLVFIDSSTGTTVSGDAGV-PVYPKQADSSSNQEEISRFPL
482

Query 423
--------GDGIANSWVPVKGQA-IANSDIILPEAPREV-LPLMSPSNLTADKKKEFQLI 472
G G+ + + G + I NS R+V LP++ P + D K+ +
Sbjct 483
PPTNAQRMGGGLESQRAGMYGPSNIINS----MNVGRDVQLPIIRP-DPRLDSTKDTRFT
537

Query 473
EGNQYLRSTVSDLSLAVDDLIIPWSELVLKEKIGAGSFGTVHRADWHGSDVAVKILMEQD
532
+G Q +L++ V+DL
IPW++LVLKE+IGAGSFGTVHRADWHGSDVAVKILMEQD
Sbjct 538
DGGQLALRKPKELAIDVEDLDIPWTDLVLKERIGAGSFGTVHRADWHGSDVAVKILMEQ
D 597

Query 533
YHLDRFKEFMREVAIMKSLRHPNIVLFMGAVTEPPNLSIVTEYLSRGSLYKLLHRSGARE
592
+H +R+ EF+REVAIMK LRHPNIVLFMGAVT+PPNLSIVTEYLSRGSLY+LLH+
GARE
Sbjct 598
FHPERYHEFLREVAIMKRLRHPNIVLFMGAVTQPPNLSIVTEYLSRGSLYRLLHKHGARE
657

Query 593
VLDERRRLNMAFDVAKGMNYLHRRSPPIVHRDLKSPNLLVDKKYTVKVCDFGLSRLKAN
T 652
LDE+RRL
MA+DVAKGMNYLH+R+PPIVHRDLKSPNLLVDKKYTVKVCDFGLSRLKANT
Sbjct 658
ALDEKRRLCMAYDVAKGMNYLHKRNPPIVHRDLKSPNLLVDKKYTVKVCDFGLSRLKA
NT 717

Query 653
YLSSKSLAGTPEWMAPEVLRDEPSNEKSDVYSFAVILWELMTLQQPWCNLNPAQVVAAV

G 712
+LSSKS AGTPEWMAPEVLRDEPSNEKSD+YSF VILWEL TLQQPW NLNPAQVVAAVG
Sbjct 718
FLSSKSAAGTPEWMAPEVLRDEPSNEKSDLYSFGVILWELATLQQPWGNLNPAQVVAAVG
777

Query 713
FKGRRLEIPKELNPQVAALIESCWANEPWRRPSFANIMETLRPLINKVPV-PQLIRSD 769
FKG+RLEIP +LNPQVAA+IE+CWANEPW+RPSF+ IM+ LRPLI K+PV PQ R+D
Sbjct 778
FKGKRLEIPHDLNPQVAAIIEACWANEPWKRPSFSTIMDLLRPLI-KLPVTPQPGRTD 834

>hypothetical protein QOZ80_2BG0188030 [Eleusine coracana subsp. coracana]
Sequence ID: KAK3154238.1 Length: 777
Range 1: 77 to 773

Score:857 bits(2215), Expect:0.0,
Method:Compositional matrix adjust.,
Identities:450/744(60%), Positives:548/744(73%), Gaps:59/744(7%)

Query 34
LQRQSSGGSSYG----AGSSVS--ASSDYPFHLPPAVAAAGGGGGTPSPCKSWAQQAEET 87
LQRQSSG S G S+VS A+ +Y PA G T KSWAQQAEE
Sbjct 77
LQRQSSGSSVGGDEMEGSSTVSTLANGEYRDADRPA------GSNT---SKSWAQQAEEA 127

Query 88
YQLQLALALRLCADAACAADPGFLDPGDSGGSKMGGGGGGSGSGRAFPLAPPSPTAEAL
S 147
Y LQLALALRLC++A+ AADP FL+ S + R +A P ++LS
Sbjct 128 YHLQLALALRLCSEASSAADPNFLE--------------SSSTDRFQQIASP----QSLS
169

Query 148
HRFWVNGSLSYSNTIPDGFYLIQGMDPFVWSMCTDVHEENRIPSVESLKSVRPDDSSIQV
207
HRFWVNGSLSYS+ +PDGFY IQGMDPF+W++C DVH+ R+PS+ESLK+V P
+S+I+V
Sbjct 170

HRFWVNGSLSYSDKVPDGFYHIQGMDPFIWTLCNDVHDGGRVPSIESLKAVDPAESAIEV 229

Query 208 VLVDRRADFDLGMLENYASSFLSSSSDMKDVINQLAKLVSSRMGGT--TSNEENLLPRWK 265
V+VD+ AD+DL L + +D K++ +LA +VS++MGG+ ++ E L PRW+
Sbjct 230 VIVDKVADYDLRQLIGMVIDVSRTRADFKEIATRLAGIVSAKMGGSVASTEEHELGPRWR 289

Query 266 ESSEAIKSSAGSIVLHLGKLPIGLCKHRSLLFKMLADKVNIPCRLVKGCKYCKAEDASSC 325
+S +K S+GS+VL +GKL +G C HR+LLFK LAD +N+PCR+VKGCKYC A A+SC
Sbjct 290 DSVGFLKISSGSVVLPIGKLSVGCCWHRALLFKTLADSINLPCRVVKGCKYCAAGAAASC 349

Query 326 VVRFGLEREYLVDLFGAPGQLSDPDSFVNGPYSLSVPSPLRPPKFRSLEITSNFSSVAKQ 385
+VRFG +REY +DL G PG LS+PDS +NG S+S+ SPLRPPK S++I NF S+AKQ
Sbjct 350 LVRFGHDREYQIDLIGNPGFLSEPDSLLNGLSSISISSPLRPPKHYSVDIADNFKSLAKQ 409

Query 386 YFSDCHSLNLLFSDASTGASNGAAVAVDQMYSKKHDAGDGIANSWVPVKGQAIANSDIIL 445
YF DC SLNL+F+D + G + +D+ + ++ P A ANSD
Sbjct 410 YFVDCQSLNLMFNDPAAGT----VIDLDET----------MGSNLGPNSSHA-ANSDCQA 454

Query 446 ----PEAPREVLPLMSPSNLTADKKKEFQLIEGNQYLRSTVSDLSLAVDDLIIPWSELVL 501
P+A + S + ++ Q + N + SD+SL ++DLIIPWSELVL
Sbjct 455 AFPHPKAGAQPNSQEGHSIMQRSFSEDAQSGQSNPF-----SDMSLDIEDLIIPWSELVL 509

Query 502 KEKIGAGSFGTVHRADWHGSDVAVKILMEQDYHLDRFKEFMREVAIMKSLRHPNIVLFMG 561

+EKIGAGSFGTVHRADW+GSDVAVKILMEQD+H +R
KEF+REVAIMKSLRHPNIVL MG
Sbjct 510
REKIGAGSFGTVHRADWNGSDVAVKILMEQDFHPERLKEFLREVAIMKSLRHPNIVLLMG
569

Query 562
AVTEPPNLSIVTEYLSRGSLYKLLHRSGAREVLDERRRLNMAFDVAKGMNYLHRRSPPIV
621
AV +PPNLSIVTEYLSRGSLY+LLHR G RE
LDERRRL+MAFDVAKGMNYLH+R+PPIV
Sbjct 570
AVAQPPNLSIVTEYLSRGSLYRLLHRHGTRESLDERRRLSMAFDVAKGMNYLHKRNPPIV
629

Query 622
HRDLKSPNLLVDKKYTVKVCDFGLSRLKANTYLSSKSLAGTPEWMAPEVLRDEPSNEKS
D 681
HRDLKSPNLLVDKKYTVKVCDFGLSRLKANT+LSSK+
AGTPEWMAPEVLRDEPSNEKSD
Sbjct 630
HRDLKSPNLLVDKKYTVKVCDFGLSRLKANTFLSSKTAAGTPEWMAPEVLRDEPSNEKS
D 689

Query 682
VYSFAVILWELMTLQQPWCNLNPAQVVAAVGFKGRRLEIPKELNPQVAALIESCWANEPW
741
VYSF VILWELMTLQQPW NLNPAQVVAAVGFKGRRLEIP
++P+VAA+IESCW EPW
Sbjct 690
VYSFGVILWELMTLQQPWSNLNPAQVVAAVGFKGRRLEIPSGVDPKVAAVIESCWVREPW
749

Query 742 RRPSFANIMETLRPLINKVPVPQL 765
RRPSFA+IME+L+PLI +P QL
Sbjct 750 RRPSFASIMESLKPLIKTLPPHQL 773

>hypothetical protein KFK09_014274 [Dendrobium nobile]
Sequence ID: KAI0508140.1 Length: 822
Range 1: 107 to 819

Score:857 bits(2214), Expect:0.0,
Method:Compositional matrix adjust.,
Identities:441/739(60%), Positives:543/739(73%), Gaps:59/739(7%)

Query 64
VAAAGGG------GGTP----SPCKSWAQQAEETYQLQLALALRLCADAACAADPGFLDP 113
+AA GGG GG S KSWAQQAEE YQLQLALALRLC++A+CA DP
+LDP
Sbjct 107
IAAIGGGEVRLKEGGEAVVLSSSSKSWAQQAEEAYQLQLALALRLCSEASCANDPNYLDP
166

Query 114
GDSGGSKMGGGGGGSGSGRAFPLAPPSPTAEALSHRFWVNGSLSYSNTIPDGFYLIQGMD
173
GD L + E SHRFWVNGSLSY++
+PDGFY+IQGMD
Sbjct 167
GDHM------------------LLAERASPETTSHRFWVNGSLSYNDKVPDGFYMIQGMD 208

Query 174
PFVWSMCTDVHEENRIPSVESLKSVRPDDSSIQVVLVDRRADFDLGMLENYASSFLSSSS
233
PFVW++CTDV EE+RIP+VESLK ++P +S I V+LVDR D +L L N A+ S S
Sbjct 209
PFVWTLCTDVQEESRIPTVESLKIIQPSESLITVILVDRCNDPELKQLLNLATGISSRFS 268

Query 234
DMKDVINQLAKLVSSRMGGTTSNEEN-LLPRWKESSEAIKSSAGSIVLHLGKLPIGLCKH
292
K+++ Q+AKLV ++MGG +EE+ LLPRWK S+A+K S+ S+V+ GKL
IGLC+H
Sbjct 269
TTKEIVEQIAKLVCTQMGGAAMDEEHGLLPRWKVGSKALKDSSRSVVISAGKLSIGLCRH
328

Query 293
RSLLFKMLADKVNIPCRLVKGCKYCKAEDASSCVVRFGLEREYLVDLFGAPGQLSDPDSF
352
R+LLFK+LAD V++PCR+ KGCK+CK ED+SSC+V FGLEREYLVDL G PG L
+P+S
Sbjct 329
RALLFKILADAVHLPCRIAKGCKFCKREDSSSCLVHFGLEREYLVDLIGTPGFLCEPNSL
388

Query 353
VNGPYSLSVPSPLRPPKFRSLEITSNFSSVAKQYFSDCHSLNLLFSDASTGASNGAAVAV
412
+NGPYS+++ SPLRPP +S ++ NF S+AKQYF DC SLNL+F+D S +
Sbjct 389
INGPYSVTILSPLRPPNLKSGDVAENFKSLAKQYFVDCRSLNLMFTDPSKENVTSHEDNI
448

Query 413
DQMYSKKHDAGDGIANSWVPVKGQAIANSDIILPEAPREVLPLMSP-------------- 458
D + K ++ A S A SD L E P+ + +++P
Sbjct 449
DNVLLKHYNGNYAEATS-------PAAMSDPELSEVPQRYVEMVAPPDGQHRASQLQIWN
501

Query 459
----SNLTADKKKEFQLI---EGNQYLRSTVSDLSLAV-DDLIIPWSELVLKEKIGAGSF 510
S+ A + + +L E L + D+ + +DL IPWS+LVLKE+IGAGSF
Sbjct 502
NHMISDQAATEATQLKLNPRDEQKDALPNLAYDVKANITNDLTIPWSDLVLKERIGAGSF
561

Query 511
GTVHRADWHGSDVAVKILMEQDYHLDRFKEFMREVAIMKSLRHPNIVLFMGAVTEPPNL
S 570
GTVH A+WHGS+VAVKILMEQD+H
+RFKEF+REV+IMKSLRHPNIVLFMGAVT+PP+LS
Sbjct 562
GTVHHAEWHGSEVAVKILMEQDFHPERFKEFLREVSIMKSLRHPNIVLFMGAVTQPPHLS
621

Query 571
IVTEYLSRGSLYKLLHRSGAREVLDERRRLNMAFDVAKGMNYLHRRSPPIVHRDLKSPNL
630
IVTEYLSRGSLY LH++G E LDERRRLNMA
DVAKGMNYLH+R+PPIVHRDLKSPNL
Sbjct 622
IVTEYLSRGSLYSFLHKTGVGETLDERRRLNMALDVAKGMNYLHKRNPPIVHRDLKSPNL
681

Query 631
LVDKKYTVKVCDFGLSRLKANTYLSSKSLAGTPEWMAPEVLRDEPSNEKSDVYSFAVILW
690

LVDKKYTVKVCDFGLSRLKANTYLSSKSLAGTPEWMAPEVLRDEPSNEKSDVYSF VILW
Sbjct 682
LVDKKYTVKVCDFGLSRLKANTYLSSKSLAGTPEWMAPEVLRDEPSNEKSDVYSFGVIL
W 741

Query 691
ELMTLQQPWCNLNPAQVVAAVGFKGRRLEIPKELNPQVAALIESCWANEPWRRPSFANIM
750
ELMTLQQPW NLNPA+VV AVGFKGRRL+IP ++NP
+A++IESCWA+EPW+RPSF++IM
Sbjct 742
ELMTLQQPWSNLNPAKVVYAVGFKGRRLDIPSDMNPVIASIIESCWASEPWKRPSFSSIM
801

Query 751 ETLRPLINKVPVPQLIRSD 769
+L+ L+N P Q +++D
Sbjct 802 VSLKQLLNSRP-SQALQTD 819

>serine/threonine-protein kinase CTR1-like [Papaver somniferum]
Sequence ID: XP_026425732.1 Length: 812
>hypothetical protein C5167_045108 [Papaver somniferum]
Sequence ID: RZC82322.1 Length: 812
Range 1: 1 to 808

Score:856 bits(2211), Expect:0.0,
Method:Compositional matrix adjust.,
Identities:463/834(56%), Positives:580/834(69%), Gaps:90/834(10%)

Query 1 MELPAAGGGRRTSYSLLSQFP--DDA--------------------------------- 24
ME+P GRRT+YSLL+QFP DD
Sbjct 1
MEMP----GRRTNYSLLNQFPPEDDKKFLPPLGVGGGNQFYESLSGEKNKGNWDGNGGDQ
56

Query 25 ---AAAGASPAVL--QRQSSGGSSYG------------AGSSVSASSDYPFHLPPAVAAA
67
G P+ L QRQSSG SS+G + S V+ L + AA
Sbjct 57
RRMGGGGLFPSSLGLQRQSSG-SSFGETLSGEYYIPTMSNSMVNDPEIDVLRLKSGIEAA
115

Query 68
GGGGGTPSPCKSWAQQAEETYQLQLALALRLCADAACAADPGFLDPGDSGGSKMGGGG
GG 127
G + + KSWAQQ EE+YQLQLALALRL ++A CA DP FLDP +
Sbjct 116
ATNGSSSTTSKSWAQQTEESYQLQLALALRLSSEATCADDPNFLDPVPDESNIRNSTN--
173

Query 128
SGSGRAFPLAPPSPTAEALSHRFWVNGSLSYSNTIPDGFYLIQGMDPFVWSMCTDVHEEN
187
S + EA+SHRFWVNG LSY + +PDGFYLI GMD +VW+MCTD+ E
Sbjct 174
------------SSSPEAMSHRFWVNGCLSYFDKVPDGFYLIHGMDSYVWTMCTDIQEHG 221

Query 188
RIPSVESLKSVRPDDSSIQVVLVDRRADFDLGMLENYASSFLSSSSDMKDVINQLAKLVS
247
R+P++ESLK+V DSSI+VVL+D+ +D L L+N S S
K+V++QLAKLV
Sbjct 222
RVPTIESLKAVD-SDSSIEVVLIDKSSDPSLIELQNRVISLSRSCVTTKEVVDQLAKLVC 280

Query 248
SRMGGTTSNEEN-LLPRWKESSEAIKSSAGSIVLHLGKLPIGLCKHRSLLFKMLADKVNI
306
MG T SN E+ L+ RWKE S+ +K GS+V+ +G L +G+C+HR+LLFK+LAD +++
Sbjct 281
IHMGDTASNGEDFLVSRWKECSQVLKDCLGSVVIPMGSLSVGVCRHRALLFKILADTIDL
340

Query 307
PCRLVKGCKYCKAEDASSCVVRFGLEREYLVDLFGAPGQLSDPDSFVNGPYSLSVPSPLR
366
PCR+ KGCKYC+ DA+SC+VRFGL+REYLVDL G PG L +PDS +NGP S+S+
SPLR
Sbjct 341
PCRIAKGCKYCRRNDAASCLVRFGLDREYLVDLLGKPGCLCEPDSLLNGPSSISIASPLR
400

Query 367
PPKFRSLEITSNFSSVAKQYFSDCHSLNLLFSDASTGASNGAAVA-VDQMYSK--KHDAG
423

P+++ E+T +F+S+AKQYF DC S+NL+FSDA TG ++G++ + Q +S+ D G
Sbjct 401
LPRYKPFEMTESFTSLAKQYFQDCQSINLIFSDAPTGVNDGSSTPPLHQGFSRPSSRDRG
460

Query 424
D-------GIANSWVPVKGQAIANSDIILPEAPREVLPLMSPSNLTADKKKEFQLIEGNQ 476
D G A S + V + P R+ ++ ++ D ++ + ++G
Sbjct 461
DIMLQSSSGPAESVISVDLRDPMLLKHTPPTGNRDGQAIVPVADPRIDMNRDLRYMQG--
518

Query 477
YLRSTVSDLSLAVDDLIIPWSELVLKEKIGAGSFGTVHRADWHGSDVAVKILMEQDYHLD
536
RS+ +DLS ++DL IPWSEL LKEKIGAGSFGTVHRADW+ S+VAVKILMEQD+H++
Sbjct 519
--RSS-NDLSFDIEDLDIPWSELELKEKIGAGSFGTVHRADWNSSEVAVKILMEQDFHVE
575

Query 537
RFKEFMREVAIMKSLRHPNIVLFMGAVTEPPNLSIVTEYLSRGSLYKLLHRSGAREVLDE
596
RFKEF+REVAIM+ LRHPNIVLFMGAVT PPNLSIVTEYLSRGSLY+LLH+SG+RE+LDE
Sbjct 576
RFKEFIREVAIMRRLRHPNIVLFMGAVTRPPNLSIVTEYLSRGSLYRLLHKSGSRELLDE
635

Query 597
RRRLNMAFDVAKGMNYLHRRSPPIVHRDLKSPNLLVDKKYTVKVCDFGLSRLKANTYLS
S 656
+RRL+MAFDVAKGMNYLHR +PPIVHRDLKSPNLLVDKKYTVKVCDFGLSRLKANT+LSS
Sbjct 636
KRRLSMAFDVAKGMNYLHRHNPPIVHRDLKSPNLLVDKKYTVKVCDFGLSRLKANTFLS
S 695

Query 657
KSLAGTPEWMAPEVLRDEPSNEKSDVYSFAVILWELMTLQQPWCNLNPAQVVAAVGFKG
R 716
KS+AGTPEWMAPEVLRDEPSNEKSD+YSF VILWEL+T+Q PW NLN AQVVAAVGFK R

Sbjct 696
KSVAGTPEWMAPEVLRDEPSNEKSDIYSFGVILWELVTMQPPWVNLNAAQVVAAVGFKN
R 755

Query 717
RLEIPKELNPQVAALIESCWANEPWRRPSFANIMETLRPLINKVPVPQLIRSDS 770
RL+IP +NPQVA LIE+CWA EPW+RPSFA+IMETL+ +I K P P +RSD+
Sbjct 756 RLDIPNNVNPQVAELIEACWAYEPWKRPSFASIMETLKAMI-KPPTPPSLRSDA
808

>hypothetical protein BS78_04G149400 [Paspalum vaginatum]
Sequence ID: KAJ1279357.1 Length: 797
Range 1: 129 to 793

Score:855 bits(2209), Expect:0.0,
Method:Compositional matrix adjust.,
Identities:436/703(62%), Positives:519/703(73%), Gaps:44/703(6%)

Query 69
GGGGTPSPC--KSWAQQAEETYQLQLALALRLCADAACAADPGFLDPGDSGGSKMGGGG
G 126
G P C KSWAQQAEE Y LQLA ALRLC++A+ AADP FLD
Sbjct 129
GDADRPPSCSSKSWAQQAEEAYHLQLAFALRLCSEASSAADPHFLD-------------- 174

Query 127
GSGSGRAFPLAPPSPTAEALSHRFWVNGSLSYSNTIPDGFYLIQGMDPFVWSMCTDVHEE
186
S + A P ++LSHRFWVNGSLSYS+ +PDGFYLIQGMDPF+W++C
DVH+
Sbjct 175
-SSADHLQHTASP----QSLSHRFWVNGSLSYSDKLPDGFYLIQGMDPFIWTLCNDVHDG
229

Query 187
NRIPSVESLKSVRPDDSSIQVVLVDRRADFDLGMLENYASSFLSSSSDMKDVINQLAKLV
246
R+PS+ESLK+V P +S+I+VV+VD+ AD+DL L + A + ++ K++ +LA +V
Sbjct 230
GRVPSIESLKAVNPTESAIEVVVVDKAADYDLRKLISMAIDVSRNRAESKEIATRLAGVV
289

Query 247
SSRMGGTT--SNEENLLPRWKESSEAIKSSAGSIVLHLGKLPIGLCKHRSLLFKMLADKV
304
S++MGG+ + E L PRW++S +K S+GS+V +GKL IG C HR+LLFK LAD +
Sbjct 290
SAKMGGSVAATEEHELGPRWRDSVGFLKISSGSVVFPIGKLSIGFCCHRALLFKTLADSI
349

Query 305
NIPCRLVKGCKYCKAEDASSCVVRFGLEREYLVDLFGAPGQLSDPDSFVNGPYSLSVPSP
364
N+PCR+VKGCKYCKA A+SC+VRFG +REYL+DL G PG LS+PDS +NG
S+SV SP
Sbjct 350
NLPCRIVKGCKYCKAAAAASCLVRFGHDREYLIDLIGNPGLLSEPDSLLNGLSSISVSSP
409

Query 365
LRPPKFRSLEITSNFSSVAKQYFSDCHSLNLLFSDASTGASNGAAVAVDQMYSKKHDAGD
424
LRPPK +I+ NF S+AKQYF DC SLNL+FSD + G D +
Sbjct 410
LRPPKHIVTDISDNFKSLAKQYFLDCQSLNLMFSDPAAGTVI--------------DLDE 455

Query 425
GIANSWVPVKGQAIANSDI--ILPEAPREVLPLMSPSNLTADKKKEFQLIEGNQYLRSTV
482
+ ++ P A NSD P LP S N + G L
Sbjct 456
AMGSNLGPNSSHA-TNSDCQATFPHLKAGALP-DSQGNFIMQRSFPEDTQSG---LSDPF
510

Query 483
SDLSLAVDDLIIPWSELVLKEKIGAGSFGTVHRADWHGSDVAVKILMEQDYHLDRFKEFM
542
SD+SL ++DLIIPW+EL LKEKIGAGSFGTVHRADW+GSDVAVKILMEQD H +R
KEF+
Sbjct 511
SDMSLEIEDLIIPWNELALKEKIGAGSFGTVHRADWNGSDVAVKILMEQDLHPERLKEFL
570

Query 543
REVAIMKSLRHPNIVLFMGAVTEPPNLSIVTEYLSRGSLYKLLHRSGAREVLDERRRLNM

602

REVAIM+SLRHPNIVL MGAVT+PPNLSIVTEYLSRGSLY+LLHR G +E LDERRRL+M

Sbjct 571 REVAIMRSLRHPNIVLLMGAVTQPPNLSIVTEYLSRGSLYRLLHRHGTKENLDERRRLSM 630

Query 603 AFDVAKGMNYLHRRSPPIVHRDLKSPNLLVDKKYTVKVCDFGLSRLKANTYLSSKSLAGT 662

AFDVAKGMNYLH+R+PPIVHRDLKSPNLLVDKKYTVKVCDFGLSRLKANT+LSSK+ AGT

Sbjct 631 AFDVAKGMNYLHKRNPPIVHRDLKSPNLLVDKKYTVKVCDFGLSRLKANTFLSSKTAAGT 690

Query 663 PEWMAPEVLRDEPSNEKSDVYSFAVILWELMTLQQPWCNLNPAQVVAAVGFKGRRLEIPK 722

PEWMAPEVLRDEPSNEKSDVYSF VILWELMTLQQPW NLNPAQVVAAVGFKGRRLEIP

Sbjct 691 PEWMAPEVLRDEPSNEKSDVYSFGVILWELMTLQQPWSNLNPAQVVAAVGFKGRRLEIPS 750

Query 723 ELNPQVAALIESCWANEPWRRPSFANIMETLRPLINKVPVPQL 765

++P+VAA+IESCW EPWRRPSF NIME+L+PLI + QL

Sbjct 751 SVDPKVAAVIESCWVREPWRRPSFFNIMESLKPLIKTLSPNQL 793

>serine/threonine-protein kinase CTR1 [Populus trichocarpa]

Sequence ID: XP_006381314.2 Length: 821

>hypothetical protein BDE02_06G102800 [Populus trichocarpa]

Sequence ID: KAI5584755.1 Length: 821

>hypothetical protein POPTR_006G115800v4 [Populus trichocarpa]

Sequence ID: PNT31071.1 Length: 821

Range 1: 131 to 811

Score:854 bits(2207), Expect:0.0,

Method:Compositional matrix adjust.,

Identities:452/708(64%), Positives:534/708(75%), Gaps:31/708(4%)

Query 61
PPAVAAAGGGGGTPSPCKSWAQQAEETYQLQLALALRLCADAACAADPGFLDP-GDSGGS
119
PP +A G S KSWAQQ EE+YQLQLALALRL ++A CA DP FLDP D
Sbjct 131
PPVDVSANNG----SSGKSWAQQTEESYQLQLALALRLSSEATCADDPHFLDPVPDESAL
186

Query 120
KMGGGGGGSGSGRAFPLAPPSPTAEALSHRFWVNGSLSYSNTIPDGFYLIQGMDPFVWSM
179
+ + S + EALSHRFWVNG LSY N IPDGFYLI
GMDP+VW++
Sbjct 187
R----------------SSTSNSPEALSHRFWVNGCLSYFNKIPDGFYLIHGMDPYVWTV 230

Query 180
CTDVHEENRIPSVESLKSVRPD-DSSIQVVLVDRRADFDLGMLENYASSFLSSSSDMKDV
238
CTD+ + RIPS+ESLKSV P+ DSS++VVL+DRR+D +L L+N SS
K+V
Sbjct 231
CTDLQDNGRIPSIESLKSVDPNADSSMEVVLIDRRSDPNLKELQNRVHGISCSSITTKEV
290

Query 239
INQLAKLVSSRMGGTTSN-EENLLPRWKESSEAIKSSAGSIVLHLGKLPIGLCKHRSLLF
297
++QLAKLV +RMGG S E++ + WKE S+ +K SIV+ +G L
IGLC+HR+LLF
Sbjct 291
VDQLAKLVCNRMGGPASRGEDDFISIWKECSDNLKDCLESIVVPIGSLSIGLCRHRALLF
350

Query 298
KMLADKVNIPCRLVKGCKYCKAEDASSCVVRFGLEREYLVDLFGAPGQLSDPDSFVNGP
Y 357
K+LAD +++PCR+ KGCKYCK +D SSC+VRFGL+REYLVDL G PG L +PDS
+NGP
Sbjct 351
KVLADTIDLPCRIAKGCKYCKRDDGSSCLVRFGLDREYLVDLVGRPGFLCEPDSLLNGPS
410

Query 358

SLSVPSPLRPPKFRSLEITSNFSSVAKQYFSDCHSLNLLFSDASTGASNGAAVAVDQMYS
417
S+S+ SPLR P+ +S E T +F +AKQYF DC SLNL+F DASTG + MY
Sbjct 411
SISISSPLRFPRIKSTESTVDFRQLAKQYFLDCQSLNLVFDDASTGTVHDGEAPGFSMYP
470

Query 418
KKHDAGDGIANSWVPVKGQAIANSDIILPEAPREVLPLMSPSNLTADKKKEFQLIEGNQY
477
KK D D ++ V + + S + LP+ + SN K+ EG+Q
Sbjct 471
KKTDRTDSEISNHVQLPSNSNEISQLPLPQKVSRI------SNHVQLPSKDSMFSEGSQL 524

Query 478
LRSTVS-DLSLAVDDLIIPWSELVLKEKIGAGSFGTVHRADWHGSDVAVKILMEQDYHLD
536
L S +LSL +D
IPW++LVLKE+IGAGSFGTVHRADWHGSDVAVKILMEQD+H D
Sbjct 525
LSGKTSKELSLDAEDSDIPWNDLVLKERIGAGSFGTVHRADWHGSDVAVKILMEQDFHAD
584

Query 537
RFKEFMREVAIMKSLRHPNIVLFMGAVTEPPNLSIVTEYLSRGSLYKLLHRSGAREVLDE
596
RFKEF+REVAIMK LRHPNIVLFMGAVT+PPNLSIVTEYLSRGSLY+LL
+SGAREVLDE
Sbjct 585
RFKEFLREVAIMKRLRHPNIVLFMGAVTQPPNLSIVTEYLSRGSLYRLLRKSGAREVLDE
644

Query 597
RRRLNMAFDVAKGMNYLHRRSPPIVHRDLKSPNLLVDKKYTVKVCDFGLSRLKANTYLS
S 656

RRRLNMA+DVAKGMNYLH+R+PPIVHRDLKSPNLLVDKKYTVKVCDFGLSR KANT+LSS
Sbjct 645
RRRLNMAYDVAKGMNYLHKRNPPIVHRDLKSPNLLVDKKYTVKVCDFGLSRFKANTFLS
S 704

Query 657
KSLAGTPEWMAPEVLRDEPSNEKSDVYSFAVILWELMTLQQPWCNLNPAQVVAAVGFKG
R 716

KS AGTPEWMAPEVLRDE SNEKSDVYSF VILWEL TLQQPW NLN AQVVAAVGFKG+
Sbjct 705 KSAAGTPEWMAPEVLRDELSNEKSDVYSFGVILWELATLQQPWSNLNAAQVVAAVGFKGK 764

Query 717 RLEIPKELNPQVAALIESCWANEPWRRPSFANIMETLRPLINKVPVPQ 764
RLEIP++LNP VAALIE+CWANEPW+RPSFA+IM++LR LI K P PQ
Sbjct 765 RLEIPRDLNPHVAALIEACWANEPWKRPSFASIMDSLRSLI-KPPTPQ 811

>PREDICTED: serine/threonine-protein kinase CTR1 isoform X2 [Populus euphratica]
Sequence ID: XP_011018886.1 Length: 818
Range 1: 106 to 808

Score:854 bits(2207), Expect:0.0,
Method:Compositional matrix adjust.,
Identities:454/733(62%), Positives:541/733(73%), Gaps:41/733(5%)

Query 43 SYGAGSSVSASSDY-------PFHLPPAVAAAGGGGGTPSPCKSWAQQAEETYQLQLALA 95
S G G+ + + Y P PP +A G S KSWAQQ EE+YQLQLALA
Sbjct 106 STGGGNEIDQAYGYEDGNFVRPAARPPVDVSANNG----SSGKSWAQQTEESYQLQLALA 161

Query 96 LRLCADAACAADPGFLDP-GDSGGSKMGGGGGGSGSGRAFPLAPPSPTAEALSHRFWVNG 154
LRL ++A CA DP FLDP D + + S + EALSHRFWVNG
Sbjct 162 LRLSSEATCADDPHFLDPVPDESALR----------------SSTSNSPEALSHRFWVNG 205

Query 155 SLSYSNTIPDGFYLIQGMDPFVWSMCTDVHEENRIPSVESLKSVRPD-DSSIQVVLVDRR 213
LSY N IPDGFYLI GMDP+VW++CTD+ + RIPS+ESLKSV P+ DSS++VVL+DRR
Sbjct 206 CLSYFNKIPDGFYLIHGMDPYVWTVCTDLQDNGRIPSIESLKSVDPNADSSMEVVLIDRR 265

Query 214
ADFDLGMLENYASSFLSSSSDMKDVINQLAKLVSSRMGG-TTSNEENLLPRWKESSEAIK
272
+D +L L+N SS K+V++QLA+LV +RMGG E+ + WKE S+
+K
Sbjct 266
SDPNLKELQNRVHGISCSSITTKEVVDQLARLVCNRMGGPACRGEDEFISIWKECSDNLK
325

Query 273
SSAGSIVLHLGKLPIGLCKHRSLLFKMLADKVNIPCRLVKGCKYCKAEDASSCVVRFGLE
332
SIV+ +G L IGLC+HRSLLFK+LAD +++PCR+ KGCKYCK +D SSC+V+FG
+
Sbjct 326
DCLESIVVPIGSLSIGLCRHRSLLFKVLADTIDLPCRIAKGCKYCKRDDGSSCLVQFGFD
385

Query 333
REYLVDLFGAPGQLSDPDSFVNGPYSLSVPSPLRPPKFRSLEITSNFSSVAKQYFSDCHS
392
REYLVDL G PG L +PDSF+NGP S+S+ SPLR P+ +S E T +F +AKQYF DC S
Sbjct 386
REYLVDLIGRPGFLCEPDSFLNGPSSISISSPLRFPRIKSTESTVDFRQLAKQYFMDCQS
445

Query 393
LNLLFSDASTGASNGAAVAVDQMYSKKHDAGDGIANSWVPVKGQAIANSDIILPEAPREV
452
LNL+F DASTG + MY KK D D ++ V + + S + LP
Sbjct 446
LNLVFDDASTGTVHDGEAPGFSMYPKKTDRTDSEISNHVQLPSNSNEISQLPLP------ 499

Query 453
LPLMSPSNLTADKKKEFQLIEGNQYLRSTVS-DLSLAVDDLIIPWSELVLKEKIGAGSFG
511
+ S ++ D K+ EG+Q L S +L L +D IPW++LVLKE+IGAGSFG
Sbjct 500
---LKVSRISVDASKDSMFSEGSQLLSGKRSKELPLDAEDSDIPWNDLVLKERIGAGSFG
556

Query 512
TVHRADWHGSDVAVKILMEQDYHLDRFKEFMREVAIMKSLRHPNIVLFMGAVTEPPNLSI
571

TVHRADWHGSDVAVKILMEQD+H +RFKEF+REVAIMK LRHPNIVLFMGAVT+PPNLSI
Sbjct 557 TVHRADWHGSDVAVKILMEQDFHAERFKEFLREVAIMKRLRHPNIVLFMGAVTQPPNLSI 616

Query 572 VTEYLSRGSLYKLLHRSGAREVLDERRRLNMAFDVAKGMNYLHRRSPPIVHRDLKSPNLL 631

VTEYLSRGSLY+LLH+SGAREVLDERRRLNMA+DVAKGMNYLH+R+PPIVHRDLKSPNLL
Sbjct 617 VTEYLSRGSLYRLLHKSGAREVLDERRRLNMAYDVAKGMNYLHKRNPPIVHRDLKSPNLL 676

Query 632 VDKKYTVKVCDFGLSRLKANTYLSSKSLAGTPEWMAPEVLRDEPSNEKSDVYSFAVILWE 691
VDKKYTVKVCDFGLSR KANT+LSSKS AGTPEWMAPEVLRDE SNEKSDVYSF VILWE
Sbjct 677 VDKKYTVKVCDFGLSRFKANTFLSSKSAAGTPEWMAPEVLRDELSNEKSDVYSFGVILWE 736

Query 692 LMTLQQPWCNLNPAQVVAAVGFKGRRLEIPKELNPQVAALIESCWANEPWRRPSFANIME 751
L TLQQPW NLN AQVVAAVGFKG+RLEIP++LNP VAALIE+CWANEPW+RPSFA+IM+
Sbjct 737 LATLQQPWSNLNAAQVVAAVGFKGKRLEIPRDLNPHVAALIEACWANEPWKRPSFASIMD 796

Query 752 TLRPLINKVPVPQ 764
+LR LI K P PQ
Sbjct 797 SLRSLI-KPPTPQ 808

>hypothetical protein PHAVU_007G223500g [Phaseolus vulgaris]
Sequence ID: XP_007145256.1 Length: 836
>hypothetical protein PHAVU_007G223500g [Phaseolus vulgaris]
Sequence ID: ESW17250.1 Length: 836

Range 1: 1 to 826

Score:854 bits(2206), Expect:0.0,
Method:Compositional matrix adjust.,
Identities:480/854(56%), Positives:588/854(68%), Gaps:118/854(13%)

Query 1 MELPAAGGGRRTSYSLLSQFPDD----------------------------------AA 25
ME+PA RR++YSLLSQFPDD A
Sbjct 1
MEMPA----RRSNYSLLSQFPDDQFSGASAPSSSGDGKTGRAGKLDRVSEWDLIADHRAG
56

Query 26 AAGA-------SPAVLQRQSSGGSS-----------YGAGSSVSASSDYP----FH---- 59
GA S LQRQSSG S Y S +A+SD FH
Sbjct 57
QQGANRIGNLYSSVGLQRQSSGSSYGESSLSGGGDFYAPTLSTAAASDVDAFGYFHDERS
116

Query 60
-LPPAVAAAGGGGGTPSPCKSWAQQAEETYQLQLALALRLCADAACAADPGFLDPG-DSG
117
A A G G KSWAQQ EE+YQLQLALALRL +DA CA DP FLDP D
G
Sbjct 117
KFSDAPARIAGSFG-----KSWAQQTEESYQLQLALALRLSSDATCADDPNFLDPMPDDG
171

Query 118
GSKMGGGGGGSGSGRAFPLAPPSPTAEALSHRFWVNGSLSYSNTIPDGFYLIQGMDPFVW
177
++ S +AEA+SHRFWVNG LSYS+ IPDGFYLI GMD
FVW
Sbjct 172 ALRL-----------------SSSAEAVSHRFWVNGCLSYSDKIPDGFYLIHGMDSFVW
213

Query 178
SMCTDVHEENRIPSVESLKSVRPDD-SSIQVVLVDRRADFDLGMLENYASSFLSSSSDMK
236
++CTD+HE RIPSV+ LKSV P SS++VVLVD+ +D L L+N A + SS
Sbjct 214
TVCTDLHENGRIPSVDMLKSVNPSIVSSVEVVLVDQNSDPSLRDLQNRAHNISCSSITTS
273

Query 237

DVINQLAKLVSSRMGGTTS-NEENLLPRWKESSEAIKSSAGSIVLHLGKLPIGLCKHRSL 295

DV++QL+KLV +RMGG+ S E+NL+ W++ S +K GS+V+ +G L +GLC+HR++

Sbjct 274 DVVDQLSKLVCNRMGGSASVGEDNLVSIWRDCSNDLKDCLGSVVIPIGSLSVGLCRHRAI 333

Query 296 LFKMLADKVNIPCRLVKGCKYCKAEDASSCVVRFGLEREYLVDLFGAPGQLSDPDSFVNG 355

LFK+LAD +++PCR+ KGCKYCK +DASSC+VRFGLEREYLVDL G PG LS+PDS +NG

Sbjct 334 LFKVLADAIDLPCRIAKGCKYCKRDDASSCLVRFGLEREYLVDLIGKPGHLSEPDSLLNG 393

Query 356 PYSLSVPSPLRPPKFRSLEITSNFSSVAKQYFSDCHSLNLLFSDASTGASNGA------- 408

P S+S SPLR P+ + E T +F S+AKQYFSDC SL L+F ++S +G

Sbjct 394 PTSISFSSPLRFPRLKPAEPTIDFRSLAKQYFSDCLSLELVFDNSSAEQFDGKYKDRNNP 453

Query 409 -AVAVDQMYSKK-----HDAG----DGIANSWVPVKG-QAIANSDI------ILPEAPRE 451

++ D S D+ + + +++P Q I +S + I + P

Sbjct 454 RPISTDSNRSSHLPLHPQDSHLSTREQFSETYLPCDAPQNIVDSTVGKYPPPIKHKRPVG 513

Query 452 VLPLMSPSNLTADKKKEFQLIEGNQYLRST-VSDLSLAVDDLIIPWSELVLKEKIGAGSF 510

+ ++ +N D + + IEG+Q + S +L+L ++DL IPWS+LVL+EKIG+GSF

Sbjct 514 IPTPLALTNTNDDMIEGKRFIEGSQLIPSKHTRELTLDMEDLDIPWSDLVLREKIGSGSF 573

Query 511 GTVHRADWHGSDVAVKILMEQDYHLDRFKEFMREVAIMKSLRHPNIVLFMGAVTEPPNLS 570

GTVHRA+W+GSDVAVKILMEQD+H +RF+EF+REV+IMK LRHPNIVLFMGAVT+PPNLS

Sbjct 574
GTVHRAEWNGSDVAVKILMEQDFHAERFQEFLREVSIMKRLRHPNIVLFMGAVTQPPNLS
633

Query 571
IVTEYLSRGSLYKLLHRSGAREVLDERRRLNMAFDVAKGMNYLHRRSPPIVHRDLKSPNL
630

IVTEYLSRGSLY+LLHRSGA+EVLDERRRL+MA+DVAKGMNYLH+R+PPIVHRDLKSPNL
Sbjct 634
IVTEYLSRGSLYRLLHRSGAKEVLDERRRLSMAYDVAKGMNYLHKRNPPIVHRDLKSPNL
693

Query 631
LVDKKYTVKVCDFGLSRLKANTYLSSKSLAGTPEWMAPEVLRDEPSNEKSDVYSFAVILW
690

LVDKKYTVKVCDFGLSRLKANT+LSSKS
AGTPEWMAPEVLRDEPSNEKSD+YSF VILW
Sbjct 694
LVDKKYTVKVCDFGLSRLKANTFLSSKSAAGTPEWMAPEVLRDEPSNEKSDIYSFGVILW
753

Query 691
ELMTLQQPWCNLNPAQVVAAVGFKGRRLEIPKELNPQVAALIESCWANEPWRRPSFANIM
750

EL TLQQPW NLNPAQVVAAVGFKG+RLEIP+++NPQVAALIE
CWANEPW+RPSFA+IM
Sbjct 754
ELATLQQPWVNLNPAQVVAAVGFKGKRLEIPRDVNPQVAALIEGCWANEPWKRPSFASIM
813

Query 751 ETLRPLINKVPVPQ 764
++LRPL+ K P PQ
Sbjct 814 DSLRPLL-KPPTPQ 826

>Serine/threonine-protein kinase [Actinidia chinensis var. chinensis]
Sequence ID: PSS34902.1 Length: 824
Range 1: 1 to 819

Score:854 bits(2206), Expect:0.0,
Method:Compositional matrix adjust.,

Identities:472/844(56%), Positives:586/844(69%), Gaps:100/844(11%)

Query 1 MELPAAGGGRRTSYSLLSQFPDD----------AAAAGASP-----------------AV 33
ME+P GRR++Y+LLSQ PDD A A GAS AV
Sbjct 1
MEIP----GRRSNYTLLSQVPDDHHHPQPPKFSAEAGGASERGFDWELVDHRAGRVGSAV
56

Query 34
---LQRQSSGGSSYGAGSSVSASSDYPFHLPPAVAAAGGGG-----------GTPSPCKS 79
LQRQSSG S++G S S +Y H G GG G+ S KS
Sbjct 57
SIGLQRQSSG-STFGESS---ISGEYYTHEDVFKVGGGSGGEARTRAAVEGSGSWSASKS
112

Query 80
WAQQAEETYQLQLALALRLCADAACAADPGFLDPG-DSGGSKMGGGGGGSGSGRAFPL
AP 138
WAQQ EE+YQLQLALALRL ++A CA DP FLDP D S+ G
Sbjct 113
WAQQTEESYQLQLALALRLSSEATCADDPNFLDPVLDESESRSLGS-------------- 158

Query 139
PSPTAEALSHRFWVNGSLSYSNTIPDGFYLIQGMDPFVWSMCTDVHEENRIPSVESLKSV
198
+ +AE++SHRFWVNG LSY + IPDGFYLI GMDP+VW++CTD+ E
RIPS+ESLK++
Sbjct 159
-TNSAESISHRFWVNGCLSYFDRIPDGFYLIHGMDPYVWTICTDLQENGRIPSIESLKAI
217

Query 199
RPD-DSSIQVVLVDRRADFDLGMLENYASSFLSSSSDMKDVINQLAKLVSSRMG---GTT
254
P DSSI+V+L+DRR D L L+N S S ++VI+QLAK+V +RMG
+
Sbjct 218
DPSMDSSIEVILIDRRTDTSLKELQNMIHSVSCSCVTTEEVIHQLAKIVCNRMGIRGAAS
277

Query 255
SNEENLLPRWKESSEAIKSSAGSIVLHLGKLPIGLCKHRSLLFKMLADKVNIPCRLVKGC
314
+ E++ +P WKE +K SIVL +G L +GLC+HR+LLFK+LAD +++PCR+ KGC

Sbjct 278
TGEDDFVPMWKECCNNLKECLRSIVLPIGSLSVGLCRHRALLFKVLADTIDLPCRVAKGC
337

Query 315
KYCKAEDASSCVVRFGLEREYLVDLFGAPGQLSDPDSFVNGPYSLSVPSPLRPPKFRSLE
374
KYC + A+SC+VRFG +REYLVDL G PG L +PDS +NGP S+ + SPL P+FR +E
Sbjct 338
KYCMTDGAASCLVRFGPDREYLVDLIGKPGCLCEPDSLLNGPSSILISSPLCFPRFRQVE
397

Query 375
ITSNFSSVAKQYFSDCHSLNLLFSDASTGASNGAAVAVDQMYSKKH-------------- 420
+F S+AKQ+FSD SLN++F D+S+GA A D Y K+
Sbjct 398
PLVDFRSLAKQFFSDFESLNIVFDDSSSGAGLDGD-AGDSTYPKQSVRQFMDIHSHVPSS
456

Query 421
-DAGDGIANSWVPVKG----QAIANSDIILPEAPR---------EVLPLMSPSNLTADKK 466
D+ + + VK I++++++ PR EV PL+ S+ +
Sbjct 457
SDSNETYDRDFQLVKSCNPHTVISSTNVVKNPVPRKHFQPIGHREVQPLLGFSDPRGNTG
516

Query 467
KEFQLIEGNQYLRSTVS-DLSLAVDDLIIPWSELVLKEKIGAGSFGTVHRADWHGSDVAV
525
K+ + IEG+Q + S ++SL V+DL
IPWS+LVLKE+IG+GSFGTVHRADWHGS+VAV
Sbjct 517
KDLRFIEGSQMVPIKPSKEISLDVEDLDIPWSDLVLKERIGSGSFGTVHRADWHGSEVAV
576

Query 526
KILMEQDYHLDRFKEFMREVAIMKSLRHPNIVLFMGAVTEPPNLSIVTEYLSRGSLYKLL
585
KILM+QD+H +R KEF+REVAIMK
LRHPNIVLFMGAVT+PPNLSIVTEYLSRGSLY+LL
Sbjct 577
KILMDQDFHPERIKEFLREVAIMKRLRHPNIVLFMGAVTQPPNLSIVTEYLSRGSLYRLL
636

Query 586
HRSGAREVLDERRRLNMAFDVAKGMNYLHRRSPPIVHRDLKSPNLLVDKKYTVKVCDFG
L 645
H+ GAREVLDERRRL+MA+DVAKGMNYLH
R+PPIVHRDLKSPNLLVDKKYTVKVCDFGL
Sbjct 637
HKPGAREVLDERRRLSMAYDVAKGMNYLHNRNPPIVHRDLKSPNLLVDKKYTVKVCDF
GL 696

Query 646
SRLKANTYLSSKSLAGTPEWMAPEVLRDEPSNEKSDVYSFAVILWELMTLQQPWCNLNPA
705
SRLKANT+LSSKS AGTPEWMAPEVLRDEPSNEKSDVYSF VILWE+
TLQQPW NLNPA
Sbjct 697
SRLKANTFLSSKSAAGTPEWMAPEVLRDEPSNEKSDVYSFGVILWEIATLQQPWSNLNPA
756

Query 706
QVVAAVGFKGRRLEIPKELNPQVAALIESCWANEPWRRPSFANIMETLRPLINKVPVPQL
765
QVVAAVGFK +RL+IP+ LNP VA++IE+CWANEP +RPSF++IME+LRPLI K P+ Q
Sbjct 757
QVVAAVGFKCKRLDIPRNLNPHVASIIEACWANEPLKRPSFSSIMESLRPLI-KPPMSQP
815

Query 766 IRSD 769
+R+D
Sbjct 816 VRAD 819

>serine/threonine-protein kinase CTR1-like isoform X1 [Lolium perenne]
Sequence ID: XP_051217156.1 Length: 773
Range 1: 11 to 769

Score:854 bits(2206), Expect:0.0,
Method:Compositional matrix adjust.,
Identities:457/803(57%), Positives:562/803(69%), Gaps:85/803(10%)

Query 4 PAAGGGRRTS---YSLLSQFP-------------------DDAAAAGASP---------A 32
P GGGRR + YSLL+ P DDAA++ P A
Sbjct 11

PMMGGGRRAAAPNYSLLATSPPTSISNDACSPHYPPPPSHDDAASSFEPPQRHSPQLGVA
70

Query 33
VLQRQSSGGSSYGAGSSVSASSDYPFHLPPAVAAAGGGGGTPSP------CKSWAQQAEE 86
QRQSSG SS G G S + A + A G P KSWAQQAEE
Sbjct 71
DWQRQSSG-SSVG-GDDADGYSSVSTTVANAESKANNAGAADRPPDSSSGGKSWAQQAE
E 128

Query 87
TYQLQLALALRLCADAACAADPGFLDPGDSGGSKMGGGGGGSGSGRAFPLAPPSPTAEA
L 146
YQLQLALALRLC+DAA AADP FLD + P TA +L
Sbjct 129 AYQLQLALALRLCSDAASAADPNFLDSSSA------------------PEHHHHHTATSL
170

Query 147
SHRFWVNGSLSYSNTIPDGFYLIQGMDPFVWSMCTDVHEENRIPSVESLKSVRPDDSSIQ
206
SHRFWVNG LSYS+ + DGFYLIQGMDPF+W++CTDV + RIPS++SL+++ P
+SS++
Sbjct 171
SHRFWVNGCLSYSDKLSDGFYLIQGMDPFIWTLCTDVQDGGRIPSIDSLRALNPTESSLE
230

Query 207
VVLVDRRADFDLGMLENYASSFLSSSSDMKDVINQLAKLVSSRMGG--TTSNEENLLPRW
264
VV+VD+ AD+DL + A + +D K++ +LA +VS +MGG T++ E
L+PRW
Sbjct 231
VVIVDKVADYDLRQHISTAIDVSRTCADSKEITARLAGIVSVKMGGSLTSTEEHELVPRW
290

Query 265
KESSEAIKSSAGSIVLHLGKLPIGLCKHRSLLFKMLADKVNIPCRLVKGCKYCKAEDASS
324
+ + +K ++ S+VL +GKL +GLC HR+LLFK +AD +N+PCR+V+GCKYCKA
A+S
Sbjct 291
TDCAGFLKITSASLVLPIGKLSVGLCCHRTLLFKAIADSINLPCRIVRGCKYCKAAGAAS
350

Query 325
CVVRFGLEREYLVDLFGAPGQLSDPDSFVNGPYSLSVPSPLRPPKFRSLEITSNFSSVAK
384
C+VRF +REYLVDL G PG LS+PDS +NG S+S+ SPLRPPK S+++ NF
S+AK
Sbjct 351
CLVRFSTDREYLVDLIGNPGLLSEPDSLLNGLSSMSISSPLRPPKHNSVDVVHNFKSLAK
410

Query 385
QYFSDCHSLNLLFSDASTGASNGAAVAVDQMYSKKHDAGDGIANSWVPVKGQAIANSDII
444
QYF DC SLNL+F+D + AA+ ++ + H + + +KG A + + I
Sbjct 411
QYFLDCKSLNLMFNDPAAAIDLDAAMGSNRGPNSSHATNSDLQTTLSHIKGDAGRDGNFI
470

Query 445
LPEAPREVLPLMSPSNLTADKKKEF--QLIEGNQYLRSTVSDLSLAVDDLIIPWSELVLK
502
+++ F ++ G R SD+SL+++DLIIPWSEL ++
Sbjct 471 --------------------RQRSFPEDILSGQ---RDPFSDVSLSIEDLIIPWSELAVR 507

Query 503
EKIGAGSFGTVHRADWHGSDVAVKILMEQDYHLDRFKEFMREVAIMKSLRHPNIVLFMG
A 562
EKIGAGSFGTVHRADW+GSDVAVKILM+QD H +R
KEF+REVAIMKSLRHPNIVL MGA
Sbjct 508
EKIGAGSFGTVHRADWNGSDVAVKILMDQDLHPERLKEFLREVAIMKSLRHPNIVLLMGA
567

Query 563
VTEPPNLSIVTEYLSRGSLYKLLHRSGAREVLDERRRLNMAFDVAKGMNYLHRRSPPIVH
622
VT+PPNLSIVTEYLSRGSLY+LLHR GARE LDERR
L+MAFDVAKGMNYLH+R+PPIVH
Sbjct 568
VTQPPNLSIVTEYLSRGSLYRLLHRHGARENLDERRCLSMAFDVAKGMNYLHKRNPPIVH
627

Query 623
RDLKSPNLLVDKKYTVKVCDFGLSRLKANTYLSSKSLAGTPEWMAPEVLRDEPSNEKSD
V 682

RDLKSPNLLVDKKYTVKVCDFGLSRLKANT+LSSK+
AGTPEWMAPEVLRDEPSNEKSDV
Sbjct 628
RDLKSPNLLVDKKYTVKVCDFGLSRLKANTFLSSKTAAGTPEWMAPEVLRDEPSNEKSD
V 687

Query 683
YSFAVILWELMTLQQPWCNLNPAQVVAAVGFKGRRLEIPKELNPQVAALIESCWANEPWR
742
YSFAVILWELMTLQ+PW NLNPAQVVAAVGF+GRR EIP
++P+VAA+IESCWA EPWR
Sbjct 688
YSFAVILWELMTLQKPWSNLNPAQVVAAVGFRGRRPEIPSSVDPKVAAIIESCWAKEPWR
747

Query 743 RPSFANIMETLRPLINKVPVPQL 765
RPSFA IME+L+PLI +P PQL
Sbjct 748 RPSFATIMESLKPLIKTLP-PQL 769

>serine/threonine-protein kinase CTR1 [Citrus sinensis]
Sequence ID: KAH9738416.1 Length: 838
Range 1: 140 to 828

Score:854 bits(2206), Expect:0.0,
Method:Compositional matrix adjust.,
Identities:435/705(62%), Positives:530/705(75%), Gaps:34/705(4%)

Query 78
KSWAQQAEETYQLQLALALRLCADAACAADPGFLDPGDSGGSKMGGGGGGSGSGRAFP
LA 137
KSWAQQ EE+YQLQLALALRL ++A CA DP FLDP + G
Sbjct 140
KSWAQQTEESYQLQLALALRLSSEATCADDPNFLDPVPDESALRSG-------------- 185

Query 138
PPSPTAEALSHRFWVNGSLSYSNTIPDGFYLIQGMDPFVWSMCTDVHEENRIPSVESLKS
197
P+ + EA+SHRFWVNG LSY + +PDGFYLI G++P+VW++CTD++E
RIPS+ESL+S
Sbjct 186
-PASSPEAISHRFWVNGCLSYFDKVPDGFYLIHGVNPYVWTVCTDMNENGRIPSIESLRS

244

Query 198
VRPD-DSSIQVVLVDRRADFDLGMLENYASSFLSSSSDMKDVINQLAKLVSSRMGGT-TS
255
V P DS I+VVL+DRR+D L L+N + + ++V++QLAKLV +RMGG+
T+
Sbjct 245
VDPSSDSLIEVVLIDRRSDPSLKELQNRVVNISCTCITTQEVVDQLAKLVCNRMGGSATA
304

Query 256
NEENLLPRWKESSEAIKSSAGSIVLHLGKLPIGLCKHRSLLFKMLADKVNIPCRLVKGCK
315
E++ +P W+E S+ IK GS+V+ +G L IGLC+HR+LLFK+LAD +++PCR+ KGCK
Sbjct 305
GEDDFVPIWRECSDDIKDCLGSVVVPIGSLSIGLCRHRTLLFKVLADAIDLPCRIAKGCK
364

Query 316
YCKAEDASSCVVRFGLEREYLVDLFGAPGQLSDPDSFVNGPYSLSVPSPLRPPKFRSLEI
375
YCK EDASSC+VRFGL+REYLVDL G PG L DPDS +NGP S+S+ SPLR P+ R E
Sbjct 365
YCKREDASSCLVRFGLDREYLVDLIGKPGHLCDPDSLLNGPSSISIASPLRFPRLRQAEP
424

Query 376
TSNFSSVAKQYFSDCHSLNLLFSDASTGASNGAAVAVDQM-------YSKKHDAGDGIAN
428
T +F +AKQ+FSDC SLNL+F +AST Q+ + HD +
Sbjct 425
TIDFRLLAKQFFSDCQSLNLVFEEASTELGVVKLPHDPQLPLPPKGGRTSGHDRDFELFK
484

Query 429
SWVPVKGQAIANSDI--------ILPEAPREVLPLMSPSNLTADKKKEFQLIEGNQYLRS 480
S P+ +++ I P R+ P +S + D K++ E ++
Sbjct 485
SCNPTQNMTHSINMVKDPNPLKHIQPIGHRDAQPGLSSIDQRVDASKDLRFTESGRLVPG
544

Query 481
TVS-DLSLAVDDLIIPWSELVLKEKIGAGSFGTVHRADWHGSDVAVKILMEQDYHLDRFK

539
S + + VDDL
IPW++LVLKEKIGAGSFGTVHRADWHGSDVAVKILMEQ++H +RFK
Sbjct 545
KPSKEFTFDVDDLDIPWNDLVLKEKIGAGSFGTVHRADWHGSDVAVKILMEQEFHAERFK
604

Query 540
EFMREVAIMKSLRHPNIVLFMGAVTEPPNLSIVTEYLSRGSLYKLLHRSGAREVLDERRR
599
EF+REVAIMK LRHPNIVLFMGAVT+PPNLSIVTEYLSRGSLY+LLH+ G
RE+LDERRR
Sbjct 605
EFLREVAIMKRLRHPNIVLFMGAVTQPPNLSIVTEYLSRGSLYRLLHKPGVREMLDERRR
664

Query 600
LNMAFDVAKGMNYLHRRSPPIVHRDLKSPNLLVDKKYTVKVCDFGLSRLKANTYLSSKS
L 659

LNMA+DVAKGMNYLHRR+PPIVHRDLKSPNLLVDKKYTVKVCDFGLSRLKANT+LSSKS
Sbjct 665
LNMAYDVAKGMNYLHRRNPPIVHRDLKSPNLLVDKKYTVKVCDFGLSRLKANTFLSSKS
A 724

Query 660
AGTPEWMAPEVLRDEPSNEKSDVYSFAVILWELMTLQQPWCNLNPAQVVAAVGFKGRRL
E 719
AGTPEWMAPEVLRDEPSNEKSD+YSF VILWEL TLQQPW
NLNPAQVVAAVGFKG+RLE
Sbjct 725
AGTPEWMAPEVLRDEPSNEKSDIYSFGVILWELATLQQPWGNLNPAQVVAAVGFKGKRLE
784

Query 720 IPKELNPQVAALIESCWANEPWRRPSFANIMETLRPLINKVPVPQ 764
IP+ +NP VA++IE+CWANEPW+RPSF+ IME LRPLI K P PQ
Sbjct 785 IPRNVNPHVASIIEACWANEPWKRPSFSTIMELLRPLI-KSPTPQ 828

>serine/threonine-protein kinase CTR1 isoform X3 [Ziziphus jujuba]
Sequence ID: XP_048321301.1 Length: 818
Range 1: 1 to 817

Score:853 bits(2205), Expect:0.0,
Method:Compositional matrix adjust.,
Identities:478/846(57%), Positives:579/846(68%), Gaps:106/846(12%)

Query 1 MELPAAGGGRRTSYSLLSQFPDD------AAAAGASPAV-------------------- 33
ME+P GRR++Y+LLSQ PDD AA AG
Sbjct 1
MEMP----GRRSNYTLLSQVPDDQFGGGSAAMAGTGATTSYYESVSGEGKNNKGKVERGF
56

Query 34 ---------------------LQRQSSGGSS---------YGAGSSVSASSD---YPFH 59
LQRQSSG S Y S +A++D Y +
Sbjct 57
DWDAVGDHRANQQGNRMFSSIGLQRQSSGSSFGESSLSGEYYAPTLSTTAANDMDAYGY
L 116

Query 60
LPPAVAAAGGGG-------------GTPSPCKSWAQQAEETYQLQLALALRLCADAACAA 106
GGGG G S KSWAQQ EE+YQLQLALALRL ++A
CA
Sbjct 117
HEDVFKIGGGGGDLRAKGVDGAVGTGGSSWGKSWAQQTEESYQLQLALALRLSSEATCA
D 176

Query 107
DPGFLDPGDSGGSKMGGGGGGSGSGRAFPLAPPSPTAEALSHRFWVNGSLSYSNTIPDGF
166
DP FLDP + + S +AEA+SHRFWVNG LSY + +PDGF
Sbjct 177
DPNFLDPVPD---------------ESALRSSSSSSAEAVSHRFWVNGCLSYFDKVPDGF 221

Query 167
YLIQGMDPFVWSMCTDVHEENRIPSVESLKSVRPD-DSSIQVVLVDRRADFDLGMLENYA
225
YLI GM+P++W++CTD+ E RIPS+ESLKSV P +SSI+ +LVDRR+D L L+N
Sbjct 222
YLIHGMNPYIWTVCTDLQENGRIPSLESLKSVDPSIESSIEAILVDRRSDPSLKELQNRV
281

Query 226
SSFLSSSSDMKDVINQLAKLVSSRMGGT-TSNEENLLPRWKESSEAIKSSAGSIVLHLGK
284
S + K+V++QLAKLV +RMGG+ T E++ + W+ESS+ +K GS+V+ LG

Sbjct 282
HGISSGCINTKEVVDQLAKLVCNRMGGSATIGEDDFVSLWRESSDDLKECLGSVVVPLGS
341

Query 285
LPIGLCKHRSLLFKMLADKVNIPCRLVKGCKYCKAEDASSCVVRFGLEREYLVDLFGAPG
344
L IGLC+HR+LLFK+LAD +++PCR+ KGCKYC DASSC+VRFG++REYLVDL
G PG
Sbjct 342
LSIGLCRHRALLFKVLADTIDLPCRIAKGCKYCTRYDASSCLVRFGIDREYLVDLIGKPG
401

Query 345
QLSDPDSFVNGPYSLSVPSPLRPPKFRSLEITSNFSSVAKQYFSDCHSLNLLFSDASTGA
404
L +PDS +NGP S+ + SPLR P+ + +E +F S+AKQYFSDC SLNL+F +ASTG
Sbjct 402
CLCEPDSLLNGPSSILISSPLRFPRLKPVEPIIDFRSLAKQYFSDCQSLNLVFDEASTGP 461

Query 405
SNGAAVAVDQMYSKKHDAGDGIANSWVPVKGQAIANSDIILPEAPREVLPLMSPSNLTAD
464
+ + MY K+ D S++ V GQ+ +S +I P R + D
Sbjct 462
ATDGDDSKVSMYPKQIDR-KCSEESFLLVTGQS-DDSSLIDPAIGRR-------EAMRVD 512

Query 465
KKKEFQLIEGNQYLRST-VSDLSLAVDDLIIPWSELVLKEKIGAGSFGTVHRADWHGSDV
523
K +L EG+Q + S + +L ++DL IPW
+LVLKE+IGAGSFGTVHRADWHGSDV
Sbjct 513
SSKSSRLFEGSQLIPSKPTKEFTLDIEDLNIPWCDLVLKERIGAGSFGTVHRADWHGSDV
572

Query 524
AVKILMEQDYHLDRFKEFMREVAIMKSLRHPNIVLFMGAVTEPPNLSIVTEYLSRGSLYK
583
AVKILMEQD+H +RFKEF+REVAIMK
LRHPNIVLFMGAVTEPPNLSIVTEYLSRGSLY+
Sbjct 573
AVKILMEQDFHAERFKEFLREVAIMKRLRHPNIVLFMGAVTEPPNLSIVTEYLSRGSLYR
632

Query 584
LLHRSGAREVLDERRRLNMAFDVAKGMNYLHRRSPPIVHRDLKSPNLLVDKKYTVKVCD
F 643

LLH+SGARE+LDERRRL+MA+DVAKGMNYLHRR+PPIVHRDLKSPNLLVDKKYTVKVCD
F
Sbjct 633
LLHKSGAREMLDERRRLSMAYDVAKGMNYLHRRNPPIVHRDLKSPNLLVDKKYTVKVC
DF 692

Query 644
GLSRLKANTYLSSKSLAGTPEWMAPEVLRDEPSNEKSDVYSFAVILWELMTLQQPWCNL
N 703
GLSRLKANT+LSSKS AGTPEWMAPEVLRDEPSNEKSDVYSF VILWEL
TLQQPW NLN
Sbjct 693
GLSRLKANTFLSSKSAAGTPEWMAPEVLRDEPSNEKSDVYSFGVILWELATLQQPWSNLN
752

Query 704
PAQVVAAVGFKGRRLEIPKELNPQVAALIESCWANEPWRRPSFANIMETLRPLINKVPVP
763
PAQVVAAVGFKG+R EIP++LN VA++IE+CWANEPW+RPSFA+IME+LRPLI
K P
Sbjct 753
PAQVVAAVGFKGKRPEIPRDLNSHVASIIEACWANEPWKRPSFASIMESLRPLI-KAPTA
811

Query 764 QLIRSD 769
Q R D
Sbjct 812 QPGRPD 817

>protein kinase superfamily protein [Actinidia rufa]
Sequence ID: GFY82708.1 Length: 824
Range 1: 1 to 819

Score:853 bits(2204), Expect:0.0,
Method:Compositional matrix adjust.,
Identities:472/844(56%), Positives:586/844(69%), Gaps:100/844(11%)

Query 1 MELPAAGGGRRTSYSLLSQFPDD----------AAAAGASP-----------------AV 33
ME+P GRR++Y+LLSQ PDD A A GAS AV
Sbjct 1
MEIP----GRRSNYTLLSQVPDDHHHPQPPKFSAEAGGASERGFDWELVDHRAGRVGSAV
56

Query 34
---LQRQSSGGSSYGAGSSVSASSDYPFHLPPAVAAAGGGG-----------GTPSPCKS 79
LQRQSSG S++G S S +Y H G GG G+ S KS
Sbjct 57
SIGLQRQSSG-STFGESS---ISGEYYTHEDVFKVGGGSGGEARTKAAAEGSGSWSASKS
112

Query 80
WAQQAEETYQLQLALALRLCADAACAADPGFLDPG-DSGGSKMGGGGGGSGSGRAFPL
AP 138
WAQQ EE+YQLQLALALRL ++A CA DP FLDP D S+ G
Sbjct 113
WAQQTEESYQLQLALALRLSSEATCADDPNFLDPVLDESTSRSLGS-------------- 158

Query 139
PSPTAEALSHRFWVNGSLSYSNTIPDGFYLIQGMDPFVWSMCTDVHEENRIPSVESLKSV
198
+ +AE++SHRFWVNG LSY + IPDGFYLI GMDP+VW++CTD+ E
RIPS+ESLK++
Sbjct 159
-TNSAESISHRFWVNGCLSYFDRIPDGFYLIHGMDPYVWTVCTDLQENGRIPSIESLKAI
217

Query 199
RPD-DSSIQVVLVDRRADFDLGMLENYASSFLSSSSDMKDVINQLAKLVSSRMG---GTT
254
P DSSI+V+L+DRR D L L+N S S ++VI+QLAK+V +RMG
+
Sbjct 218
DPSMDSSIEVILIDRRTDTSLKELQNMIHSVSCSCVTTEEVIHQLAKIVCNRMGIRGAAS
277

Query 255
SNEENLLPRWKESSEAIKSSAGSIVLHLGKLPIGLCKHRSLLFKMLADKVNIPCRLVKGC
314
+ E++ +P WKE + +K SIVL +G L +GLC+HR+LLFK+LAD +++PCR+ KGC
Sbjct 278
TGEDDFVPMWKECCDNLKECLRSIVLPIGSLSVGLCRHRALLFKVLADTIDLPCRVAKGC

337

Query 315
KYCKAEDASSCVVRFGLEREYLVDLFGAPGQLSDPDSFVNGPYSLSVPSPLRPPKFRSLE
374
KYC + A+SC+VRFG +REYLVDL G PG L PDS +NGP S+ + SPL P+FR +E
Sbjct 338
KYCMTDGAASCLVRFGPDREYLVDLIGKPGCLCAPDSLLNGPSSILISSPLCFPRFRQVE
397

Query 375
ITSNFSSVAKQYFSDCHSLNLLFSDASTGASNGAAVAVDQMYSKKH-------------- 420
+F S+AKQ+FSD SLN++F D+S+GA A D Y K+
Sbjct 398
PLVDFRSLAKQFFSDFESLNIVFDDSSSGAGLDGD-AGDSTYPKQSVRQFMDIHSHVPSS
456

Query 421
-DAGDGIANSWVPVKG----QAIANSDIILPEAPR---------EVLPLMSPSNLTADKK 466
D+ + + VK I++++++ PR EV PL+ S+ +
Sbjct 457
SDSNETYDRDFQLVKSCNPHAVISSTNVVKNPVPRKHFQPIGHREVQPLLGFSDPRGNTG
516

Query 467
KEFQLIEGNQYLRSTVS-DLSLAVDDLIIPWSELVLKEKIGAGSFGTVHRADWHGSDVAV
525
K+ + IEG+Q + S ++SL V+DL
IPWS+LVLKE+IG+GSFGTVHRADWHGS+VAV
Sbjct 517
KDLRFIEGSQMVPIKPSKEISLDVEDLDIPWSDLVLKERIGSGSFGTVHRADWHGSEVAV
576

Query 526
KILMEQDYHLDRFKEFMREVAIMKSLRHPNIVLFMGAVTEPPNLSIVTEYLSRGSLYKLL
585
KILM+QD+H +R KEF+REVAIMK
LRHPNIVLFMGAVT+PPNLSIVTEYLSRGSLY+LL
Sbjct 577
KILMDQDFHPERIKEFLREVAIMKRLRHPNIVLFMGAVTQPPNLSIVTEYLSRGSLYRLL
636

Query 586
HRSGAREVLDERRRLNMAFDVAKGMNYLHRRSPPIVHRDLKSPNLLVDKKYTVKVCDFG

L 645
H+ GAREVLDERRRL+MA+DVAKGMNYLH R+PPIVHRDLKSPNLLVDKKYTVKVCDFGL
Sbjct 637 HKPGAREVLDERRRLSMAYDVAKGMNYLHNRNPPIVHRDLKSPNLLVDKKYTVKVCDFGL 696

Query 646 SRLKANTYLSSKSLAGTPEWMAPEVLRDEPSNEKSDVYSFAVILWELMTLQQPWCNLNPA 705
SRLKANT+LSSKS AGTPEWMAPEVLRDEPSNEKSDVYSF VILWE+ TLQQPW NLNPA
Sbjct 697 SRLKANTFLSSKSAAGTPEWMAPEVLRDEPSNEKSDVYSFGVILWEIATLQQPWSNLNPA 756

Query 706 QVVAAVGFKGRRLEIPKELNPQVAALIESCWANEPWRRPSFANIMETLRPLINKVPVPQL 765
QVVAAVGFK +RL+IP+ LNP VA++IE+CWANEP +RPSF++IME+LRPLI K P+ Q
Sbjct 757 QVVAAVGFKCKRLDIPRNLNPHVASIIEACWANEPLKRPSFSSIMESLRPLI-KPPMSQP 815

Query 766 IRSD 769
+R+D
Sbjct 816 VRAD 819

>serine/threonine-protein kinase CTR1 isoform X2 [Elaeis guineensis]
Sequence ID: XP_019703234.1 Length: 754
Range 1: 1 to 746

Score:852 bits(2202), Expect:0.0,
Method:Compositional matrix adjust.,
Identities:453/775(58%), Positives:562/775(72%), Gaps:47/775(6%)

Query 1 MELPAAGGGRRTSYSLLSQFPDDAAAAGASPAVLQRQSSGGSSYGAGSSVSASSDYPFHL 60
ME+P RR YSLLSQFPD+ AAA + LQRQSS S YP
Sbjct 1

MEMPE----RRPGYSLLSQFPDEPAAAAVALDWLQRQSS---GSSVRESSMLGDCYP--- 50

Query 61
PPAVAAAG--GGGGTPSPCKSWAQQAEETYQLQLALALRLCADAACAADPGFLDPGDSGG
118
PP ++ G+ SP KSWAQQAEETYQLQLALALRLC++AA AADP FLD GD
Sbjct 51
PPTLSTVCEETAIGSSSPNKSWAQQAEETYQLQLALALRLCSEAASAADPNFLDAGDQLA
110

Query 119
SKMGGGGGGSGSGRAFPLAPPSPTAEALSHRFWVNGSLSYSNTIPDGFYLIQGMDPFVWS
178
+A++LSHRFWVNG LSY + I DGFYLI+GMDP VW+
Sbjct 111 VVQNA------------------SAKSLSHRFWVNGCLSYYDKILDGFYLIRGMDPLVWT
152

Query 179
MCTDVHEENRIPSVESLKSVRPDDSSIQVVLVDRRADFDLGMLENYASSFLSSSSDMKDV
238
+CTDV E+RIPS+ESL++++P DSSI+V ++DR D+DL L+ S +
KDV
Sbjct 153
LCTDVQIESRIPSIESLRTIQPIDSSIEVAVIDRLGDYDLRQLQKTVLSISYNHGTRKDV 212

Query 239
INQLAKLVSSRMGGTTSNEEN-LLPRWKESSEAIKSSAGSIVLHLGKLPIGLCKHRSLLF
297
I++LA+LV ++MGG S+EE+ LLPRWK+ SE +K+S+GS+V LGKL
+GLC+HR+LLF
Sbjct 213
IDKLARLVCTQMGGVASDEESELLPRWKDCSEFLKASSGSVVFPLGKLSVGLCRHRALLF
272

Query 298
KMLADKVNIPCRLVKGCKYCKAEDASSCVVRFGLEREYLVDLFGAPGQLSDPDSFVNGP
Y 357
KMLAD +N+PCR+ KGCKYCK+ DASSC+V G ERE+L+DL PG + +PDS
+NG
Sbjct 273
KMLADTINLPCRVAKGCKYCKSNDASSCLVLLGPEREFLIDLIRNPGSVCEPDSLLNGLS
332

Query 358

SLSVPSPLRPPKFRSLEITSNFSSVAKQYFSDCHSLNLLFSDASTGASNGAAVAVDQMYS
417
S+ + SPL PKF+ + IT N S+AKQYF C SLNL+F DAS G + A Q ++
Sbjct 333
SILISSPLHIPKFKMVGITDNLRSLAKQYFLVCQSLNLMFRDASAGGDAEVSQAT-QPHA
391

Query 418
KKHDAGDGIANSWVPVK----GQAIANSDIILPEAPR----------EVLPLMSPSNLTA 463
++ DG + + + Q + +S+ IL E V P ++ S+ A
Sbjct 392
ERVAQPDGQDGNLLLHELHNPSQNVVSSEQILREQSHVKQIAQVGHLNVSPSLAFSDSRA
451

Query 464
DKKKEFQLIEGNQYLRST-VSDLSLAVDDLIIPWSELVLKEKIGAGSFGTVHRADWHGSD
522
+K + + I+ + + ++ D SLA+DDL+IPW +LVLKEKIGAGSFG VHRADW+
SD
Sbjct 452
EKNNDLRFIKEHHFGQTKPTKDTSLAMDDLMIPWRDLVLKEKIGAGSFGIVHRADWNDS
D 511

Query 523
VAVKILMEQDYHLDRFKEFMREVAIMKSLRHPNIVLFMGAVTEPPNLSIVTEYLSRGSLY
582
VAVKILMEQD H + KEF+REVAIM+SLRHPNIVL
MGAVT+PPNL+IVTEYLSRGSLY
Sbjct 512
VAVKILMEQDNHPECLKEFLREVAIMRSLRHPNIVLLMGAVTQPPNLAIVTEYLSRGSLY
571

Query 583
KLLHRSGAREVLDERRRLNMAFDVAKGMNYLHRRSPPIVHRDLKSPNLLVDKKYTVKVC
D 642
+LLHR ARE LDE+
RLNMAFDVAKGMNYLH+R+PPIVHRDLKSPNLLVDK+YTVKV D
Sbjct 572
RLLHRPDARENLDEKCRLNMAFDVAKGMNYLHQRNPPIVHRDLKSPNLLVDKEYTVKV
GD 631

Query 643
FGLSRLKANTYLSSKSLAGTPEWMAPEVLRDEPSNEKSDVYSFAVILWELMTLQQPWCNL
702

FGLSRLK NT+L S++ AGTPEWMAPE+ RDE SNEKSDVYSF VILWELM+LQQPW NL
Sbjct 632 FGLSRLKENTFLCSRTAAGTPEWMAPEIFRDELSNEKSDVYSFGVILWELMSLQQPWSNL 691

Query 703 NPAQVVAAVGFKGRRLEIPKELNPQVAALIESCWANEPWRRPSFANIMETLRPLI 757
NPAQVVAAVGFKGRRLEIP +++P+VA +I+SCWANEPW+RPSFA+IME+L+PLI
Sbjct 692 NPAQVVAAVGFKGRRLEIPSDVDPRVAGIIKSCWANEPWKRPSFASIMESLKPLI 746
