# Supplementary material for: Genome-wide evolutionary analysis of TKL_CTR1-DRK-2 gene family and functional characterization reveals that TaCTR1 positively regulates flowering time in wheat
Source: BMC Genomics. 2024 May 14;25:474. doi: 10.1186/s12864-024-10383-2 (PMC11092142; doi:10.1186/s12864-024-10383-2)

T.ae TKL_CTR1−DRK−2 I subfamily exon−intron and kinase domain diagram (all)

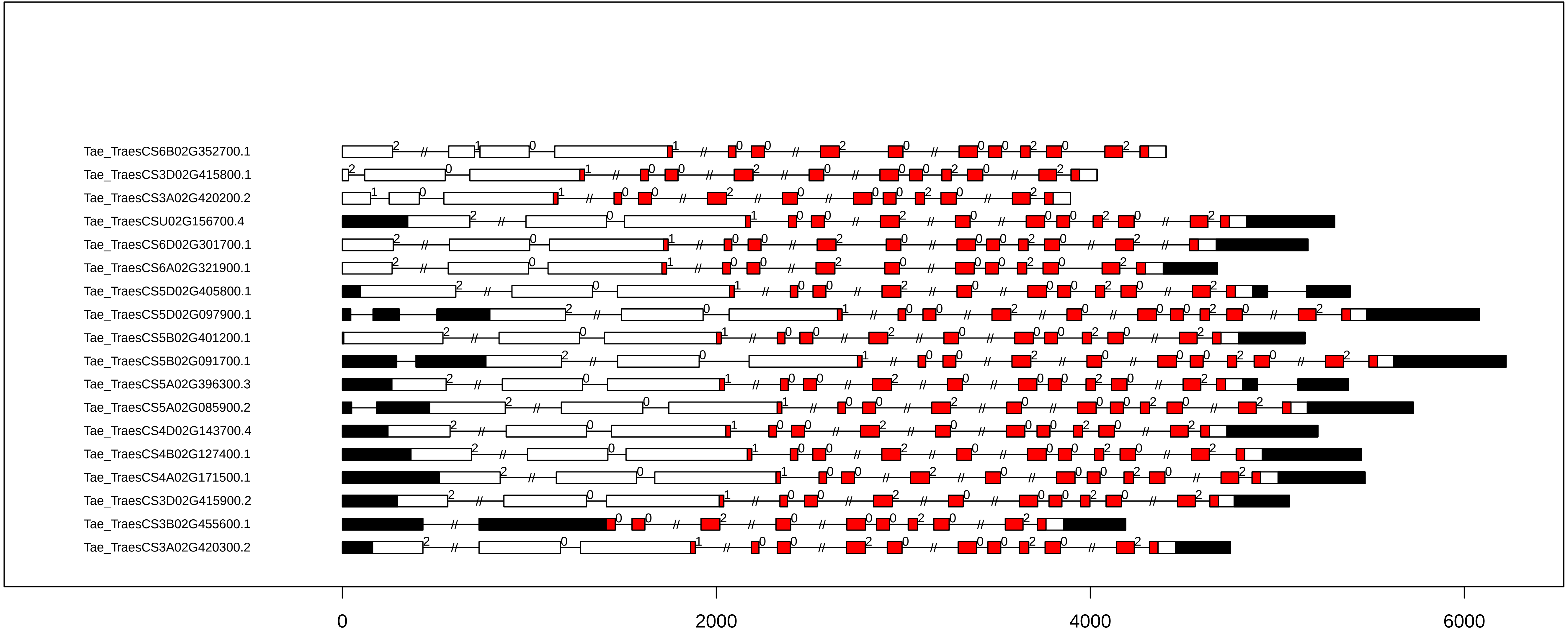

T.ae TKL_CTR1−DRK−2 II subfamily exon−intron and kinase domain diagram (all)

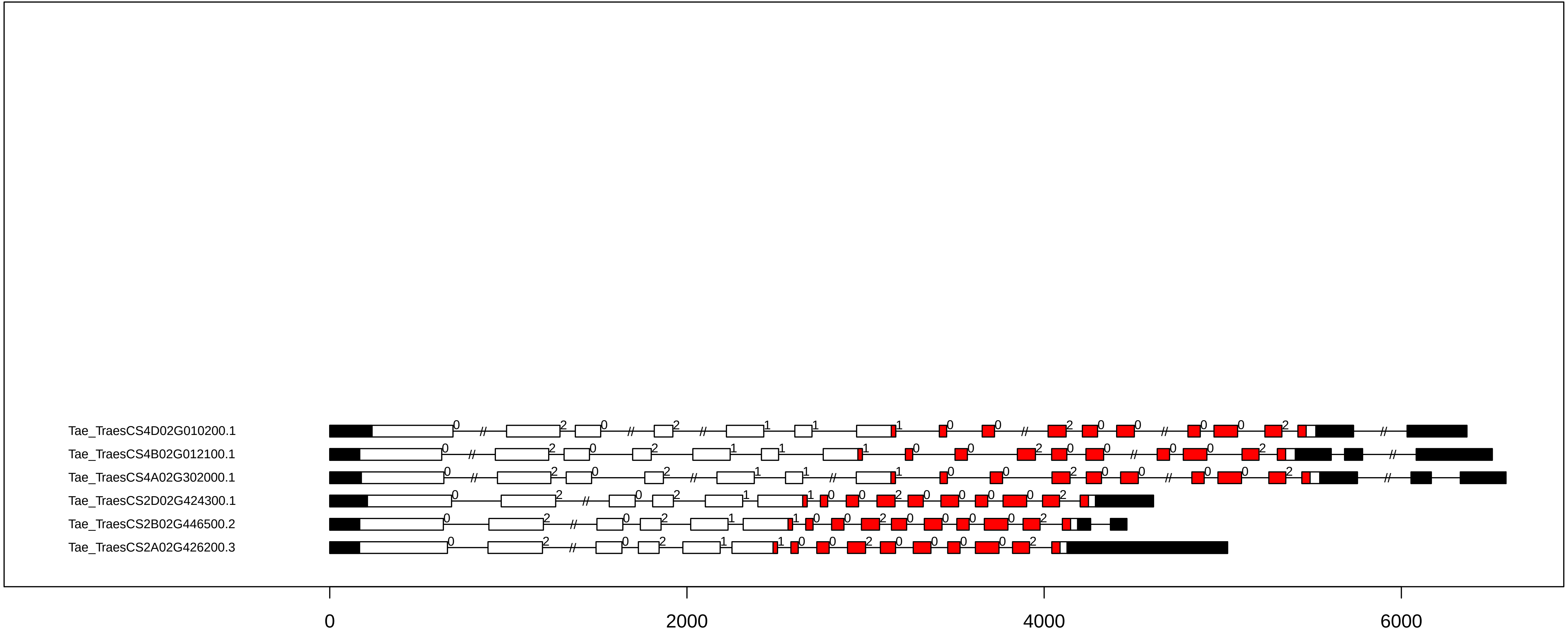

T.ae TKL_CTR1−DRK−2 III subfamily exon−intron and kinase domain diagram (all)

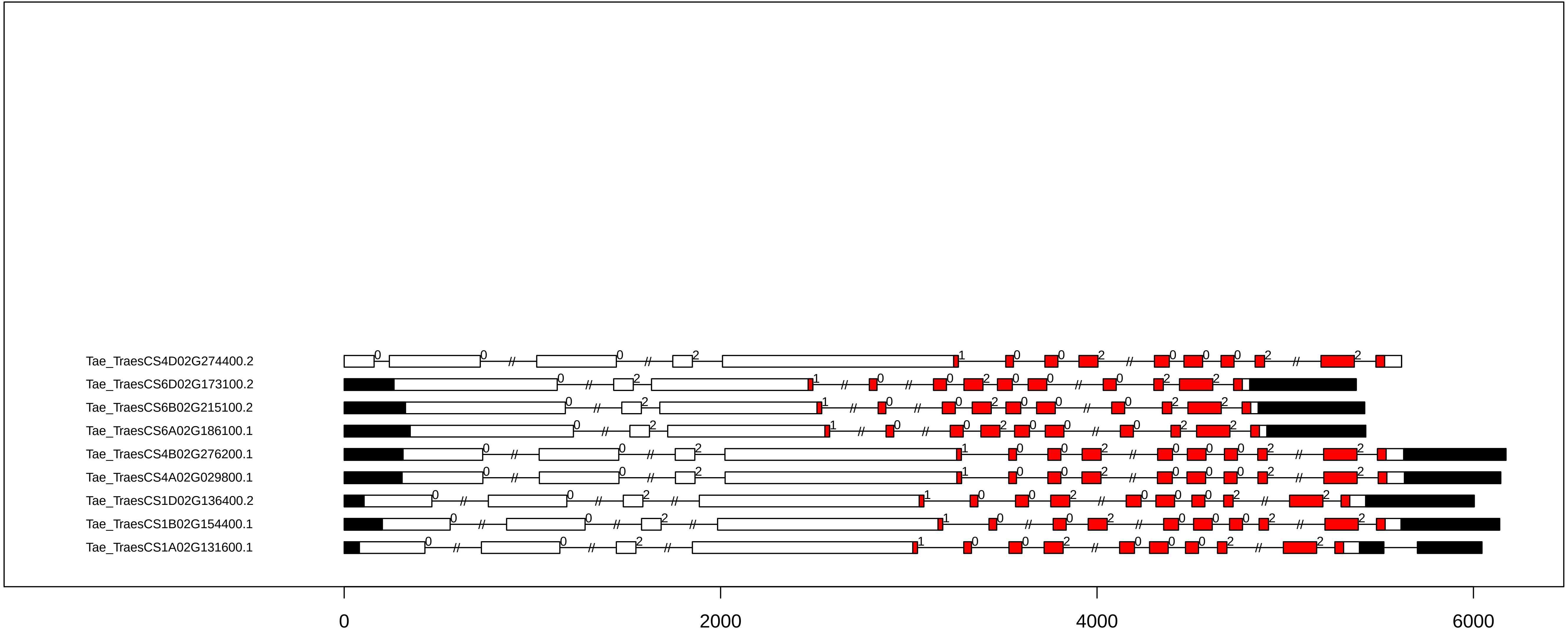

T.ae TKL_CTR1−DRK−2 IV subfamily exon−intron and kinase domain diagram (all)

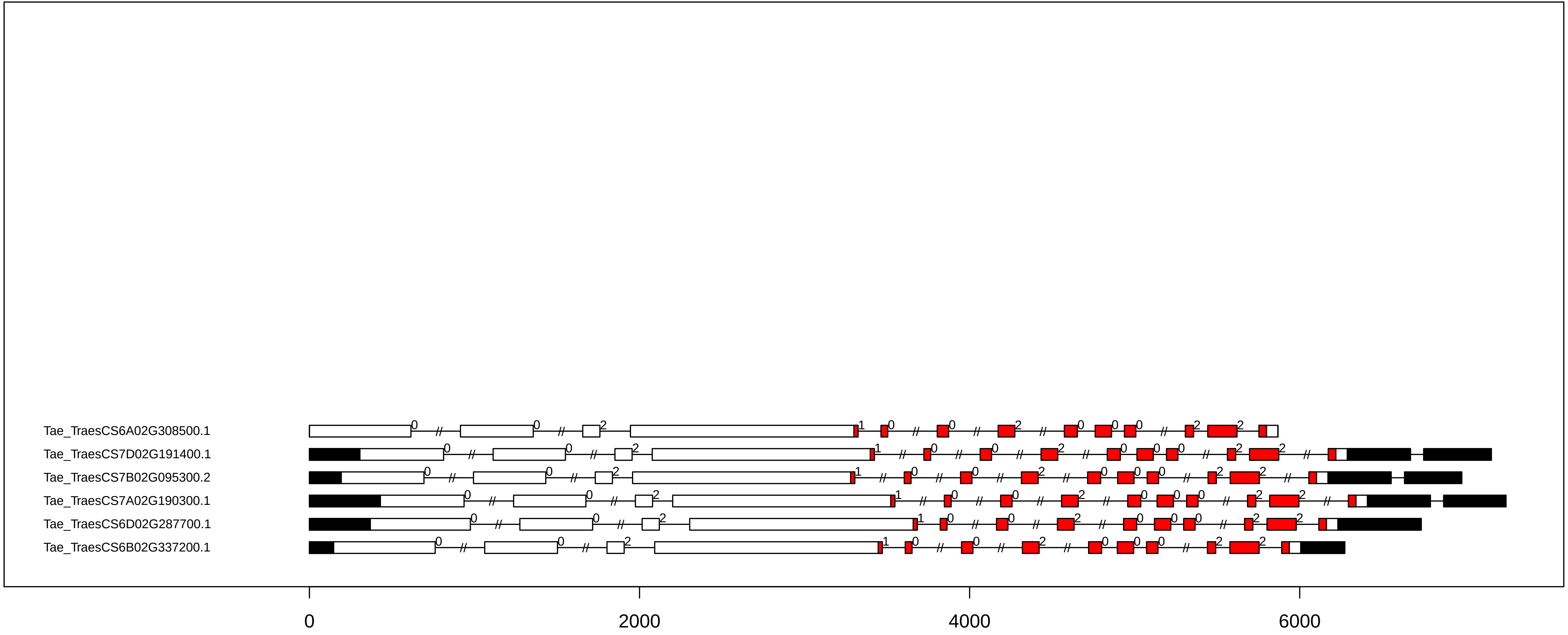

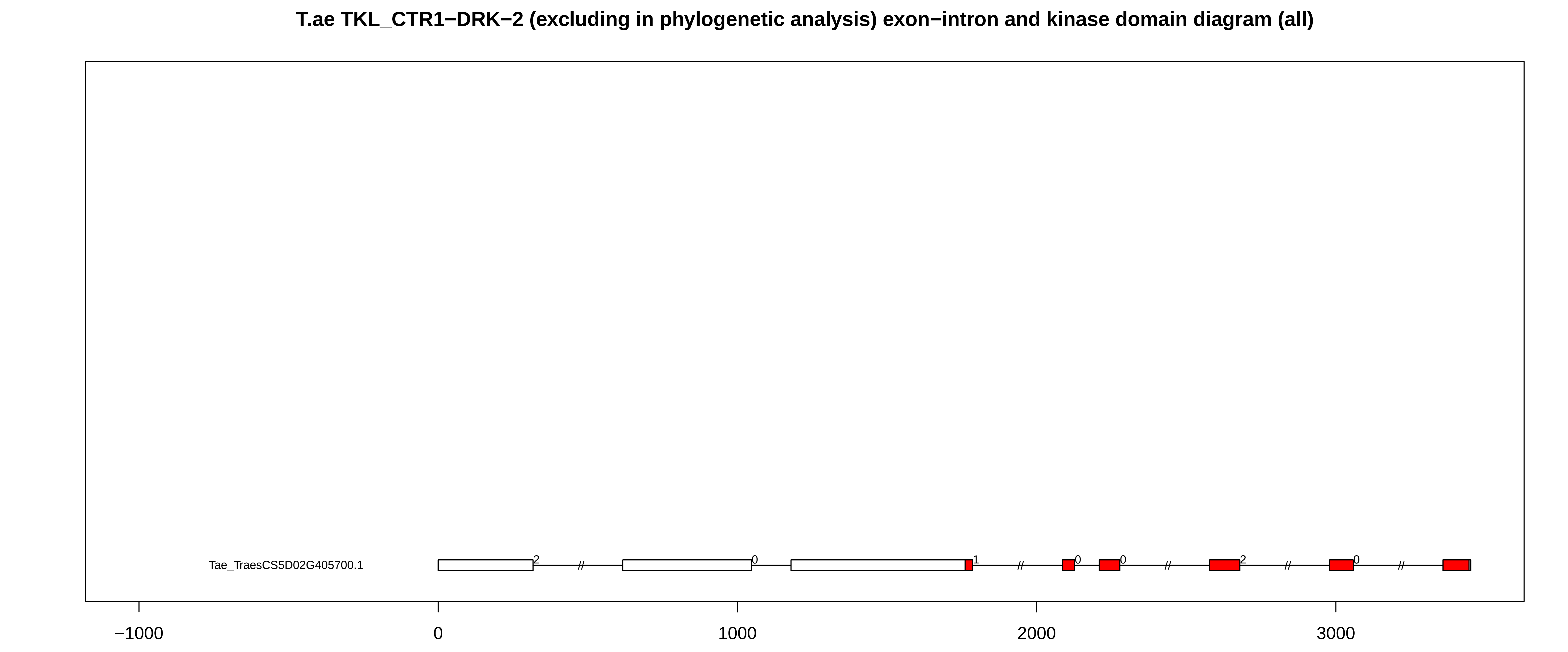
T.ae TKL_CTR1−DRK−2 (excluding in phylogenetic analysis) exon−intron and kinase domain diagram (all)
Tae_TraesCS5D02G405700.1
2
0
1
0
0
2
0
−1000
0
1000
2000
3000

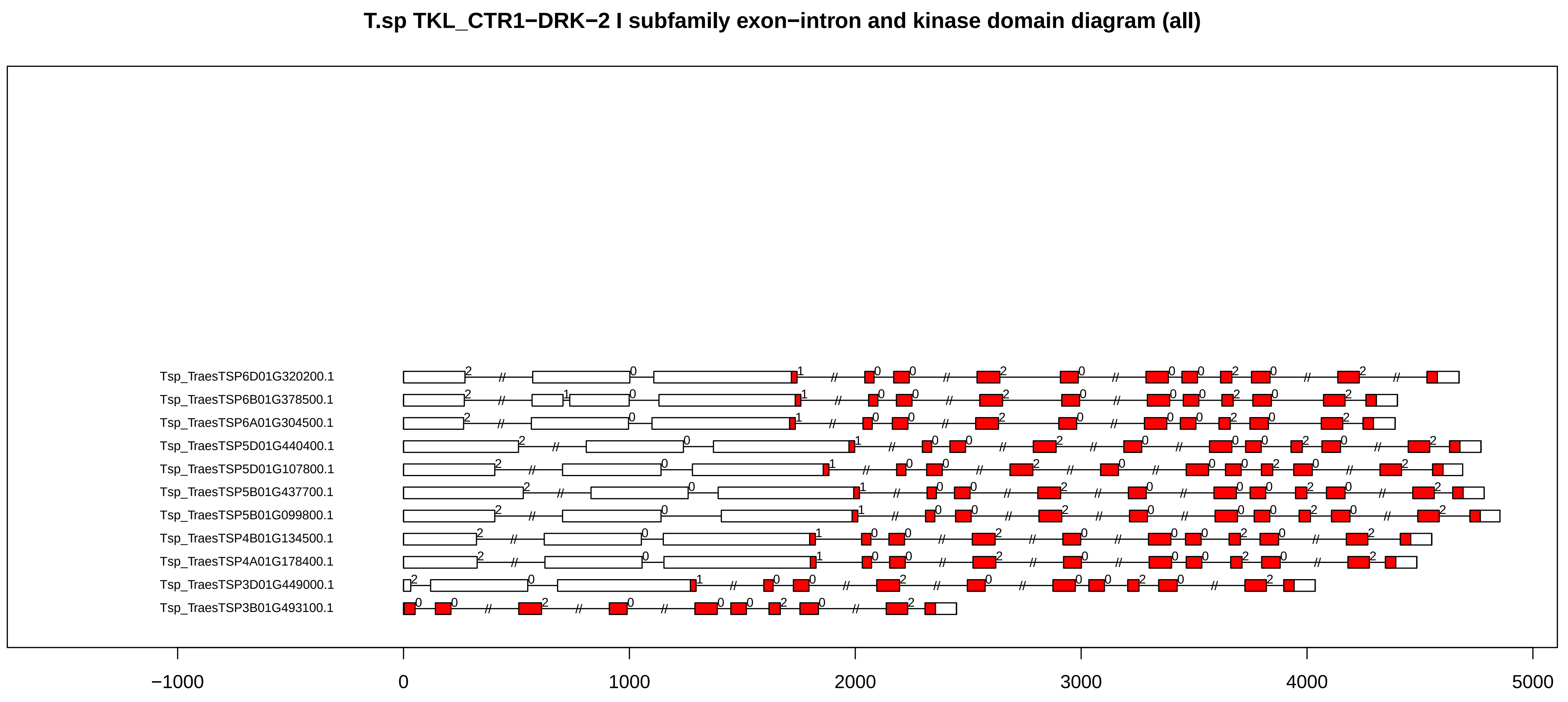

T.sp TKL_CTR1−DRK−2 I subfamily exon−intron and kinase domain diagram (all)
Tsp_TraesTSP6D01G320200.1
Tsp_TraesTSP6B01G378500.1
Tsp_TraesTSP6A01G304500.1
Tsp_TraesTSP5D01G440400.1
Tsp_TraesTSP5D01G107800.1
Tsp_TraesTSP5B01G437700.1
Tsp_TraesTSP5B01G099800.1
Tsp_TraesTSP4B01G134500.1
Tsp_TraesTSP4A01G178400.1
Tsp_TraesTSP3D01G449000.1
Tsp_TraesTSP3B01G493100.1
−1000
0
1000
2000
3000
4000
5000

T.sp TKL_CTR1−DRK−2 II subfamily exon−intron and kinase domain diagram (all)

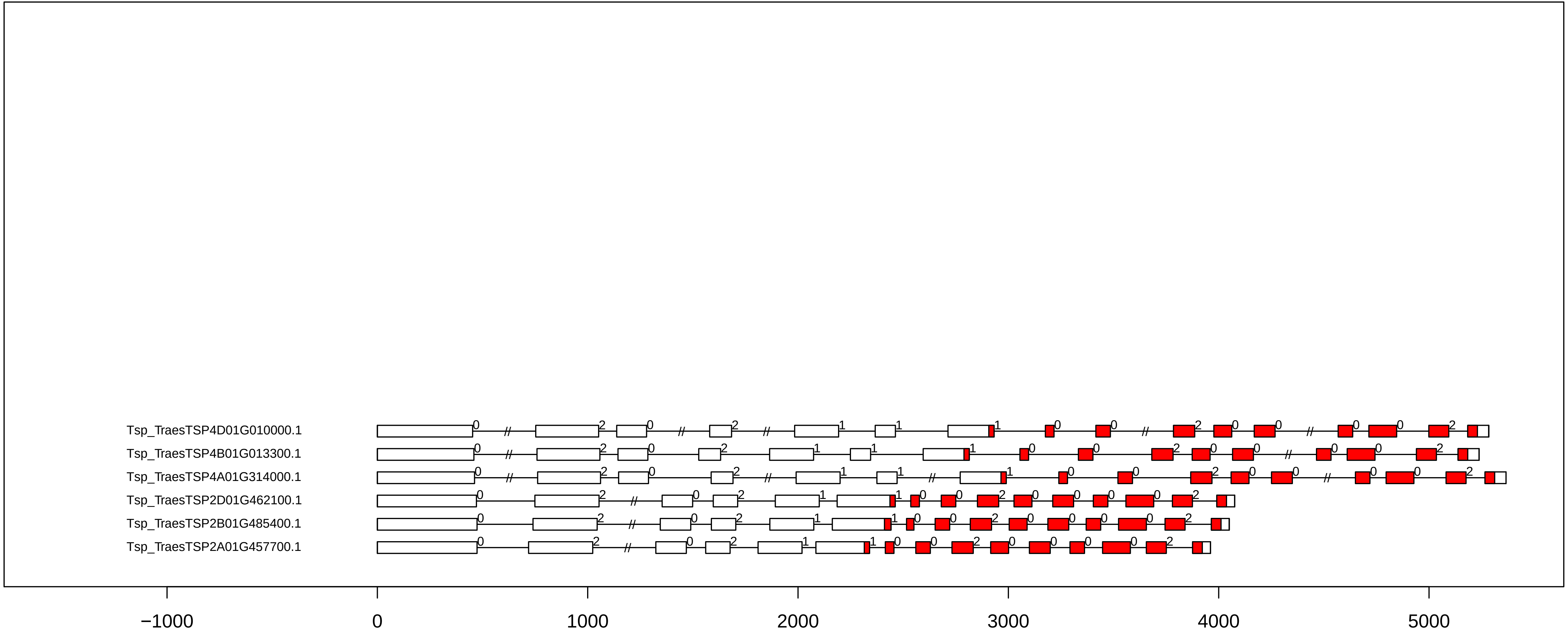

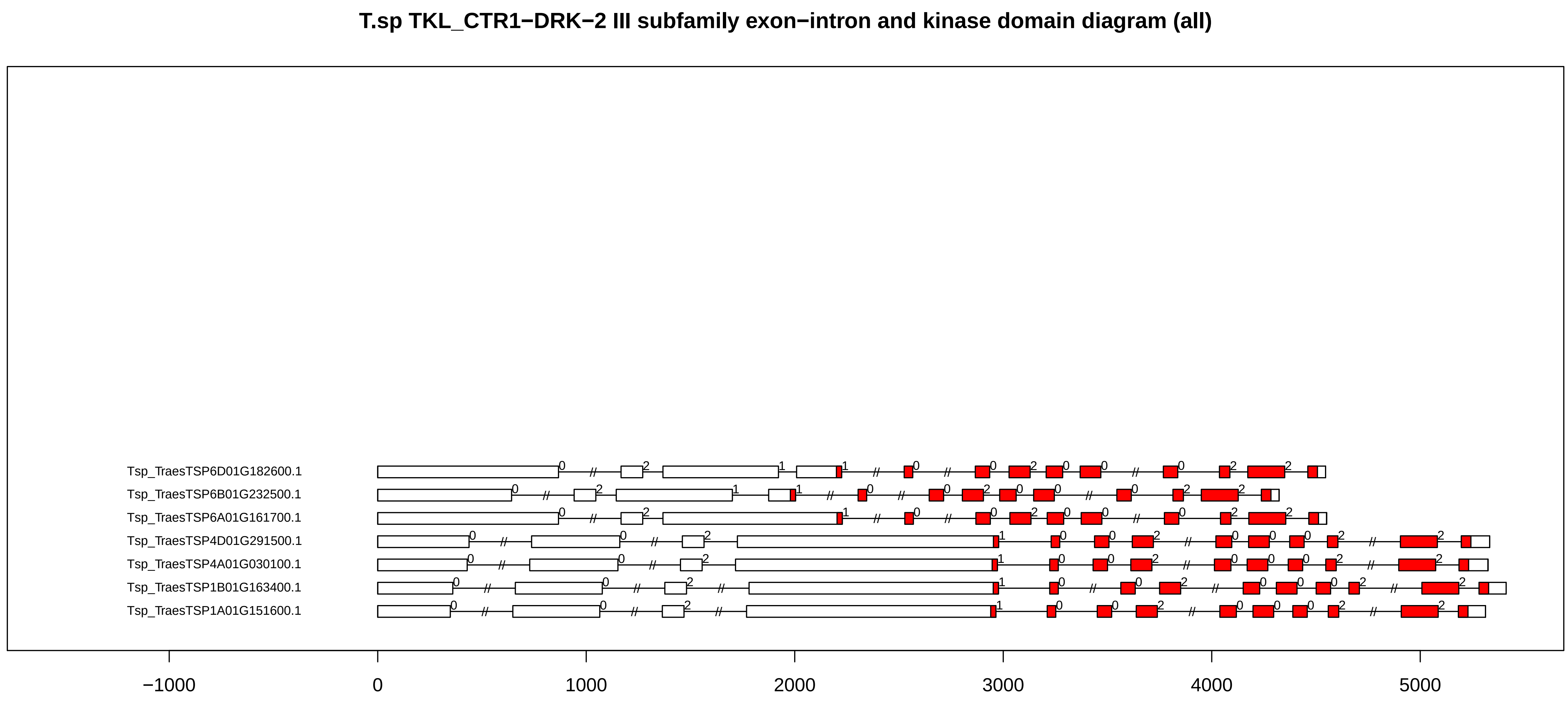

T.sp TKL_CTR1−DRK−2 III subfamily exon−intron and kinase domain diagram (all)
Tsp_TraesTSP6D01G182600.1
Tsp_TraesTSP6B01G232500.1
Tsp_TraesTSP6A01G161700.1
Tsp_TraesTSP4D01G291500.1
Tsp_TraesTSP4A01G030100.1
Tsp_TraesTSP1B01G163400.1
Tsp_TraesTSP1A01G151600.1
−1000
0
1000
2000
3000
4000
5000

T.sp TKL_CTR1−DRK−2 IV subfamily exon−intron and kinase domain diagram (all)

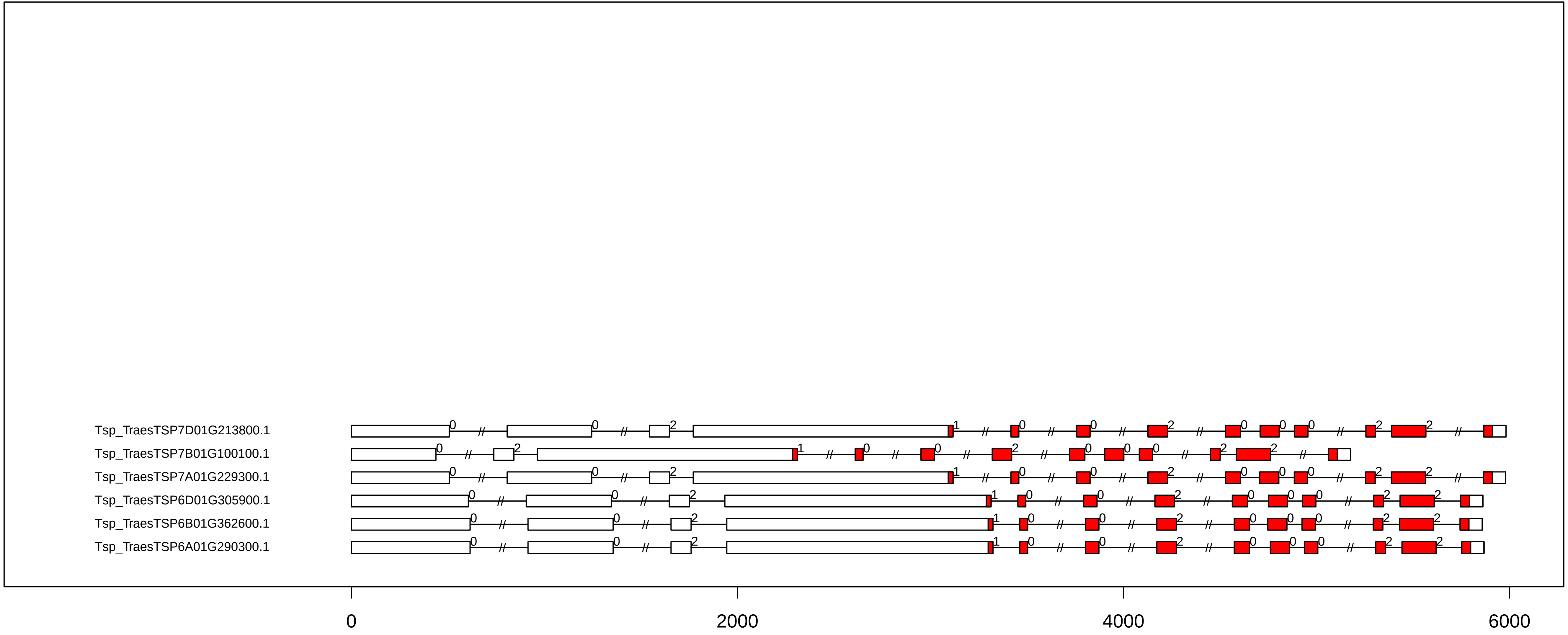

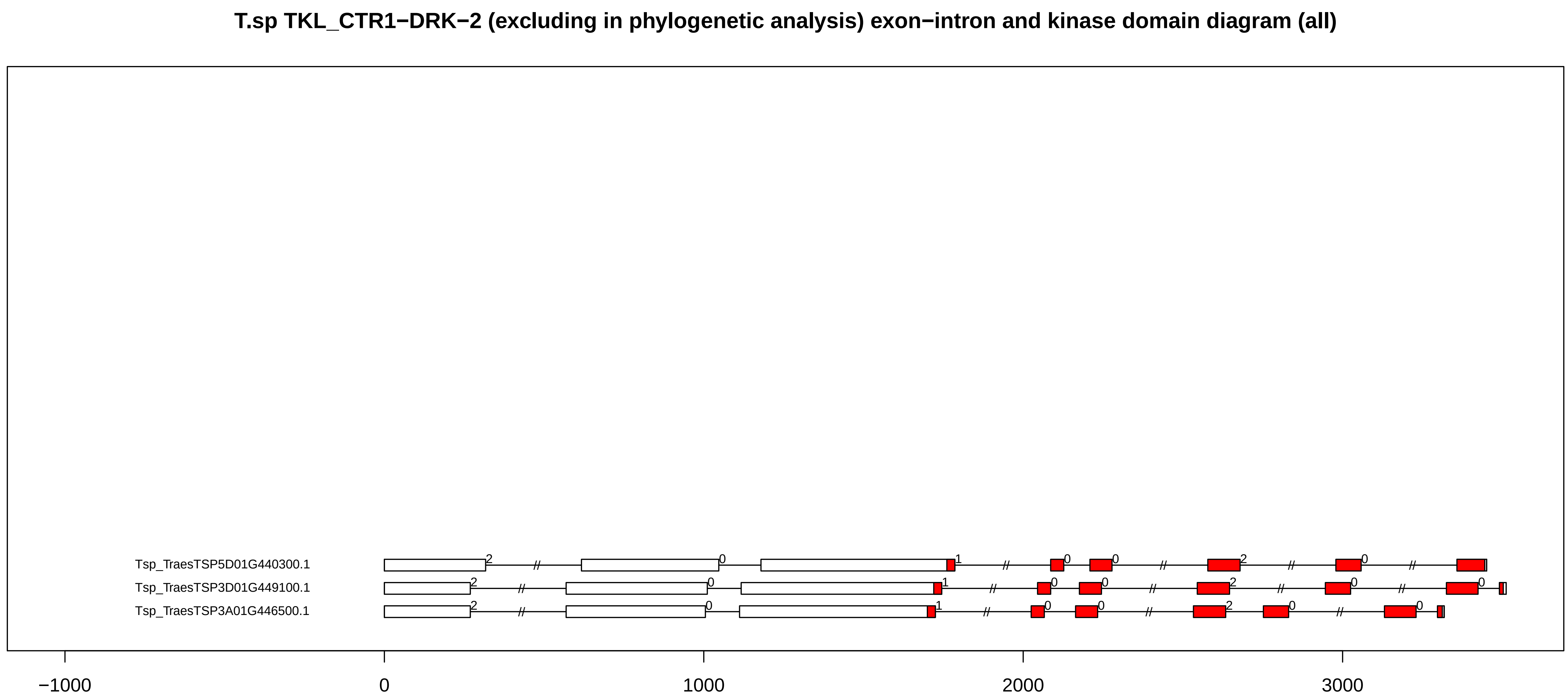
T.sp TKL_CTR1−DRK−2 (excluding in phylogenetic analysis) exon−intron and kinase domain diagram (all)
Tsp_TraesTSP5D01G440300.1
Tsp_TraesTSP3D01G449100.1
Tsp_TraesTSP3A01G446500.1
−1000
0
1000
2000
3000

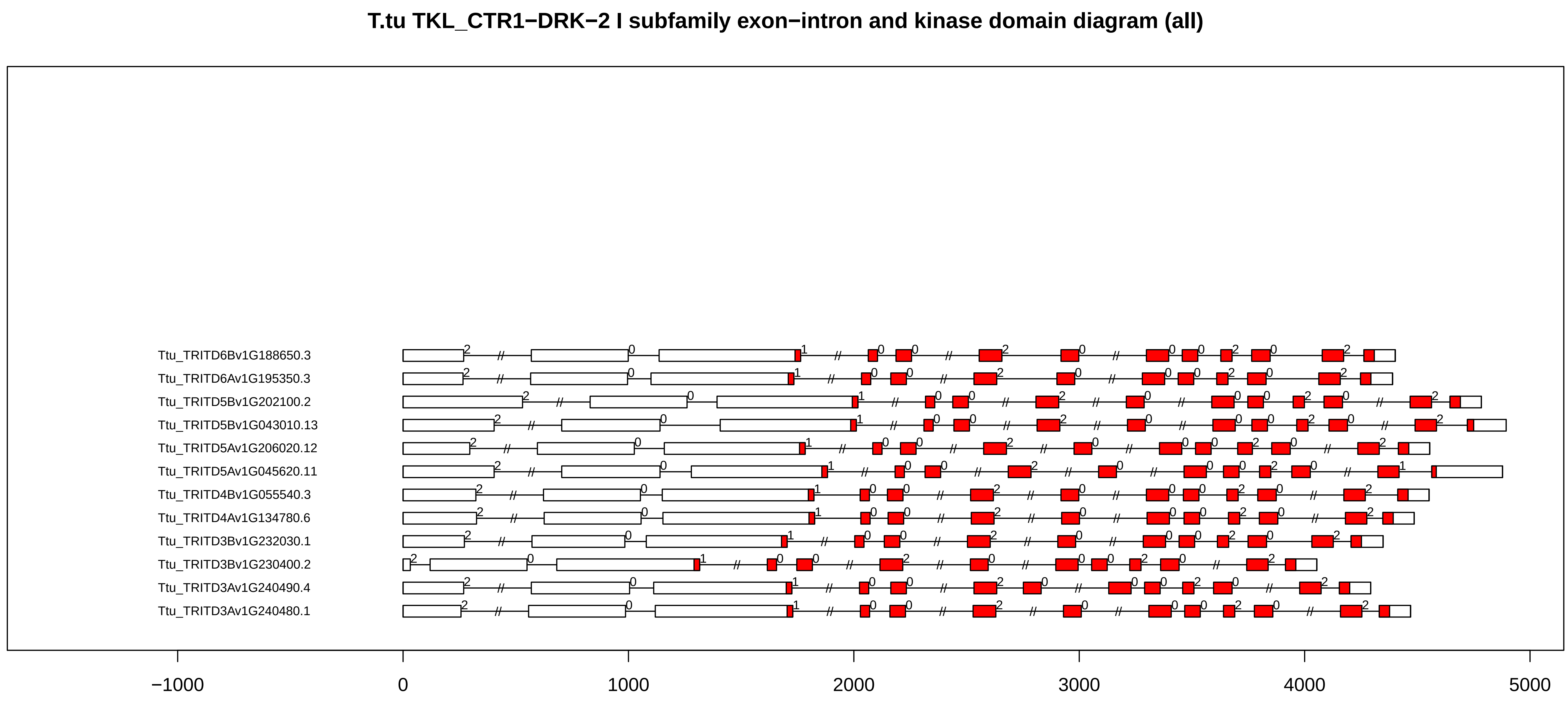

T.tu TKL_CTR1−DRK−2 I subfamily exon−intron and kinase domain diagram (all)
Ttu_TRITD6Bv1G188650.3
Ttu_TRITD6Av1G195350.3
Ttu_TRITD5Bv1G202100.2
Ttu_TRITD5Bv1G043010.13
Ttu_TRITD5Av1G206020.12
Ttu_TRITD5Av1G045620.11
Ttu_TRITD4Bv1G055540.3
Ttu_TRITD4Av1G134780.6
Ttu_TRITD3Bv1G232030.1
Ttu_TRITD3Bv1G230400.2
Ttu_TRITD3Av1G240490.4
Ttu_TRITD3Av1G240480.1
−1000
0
1000
2000
3000
4000
5000

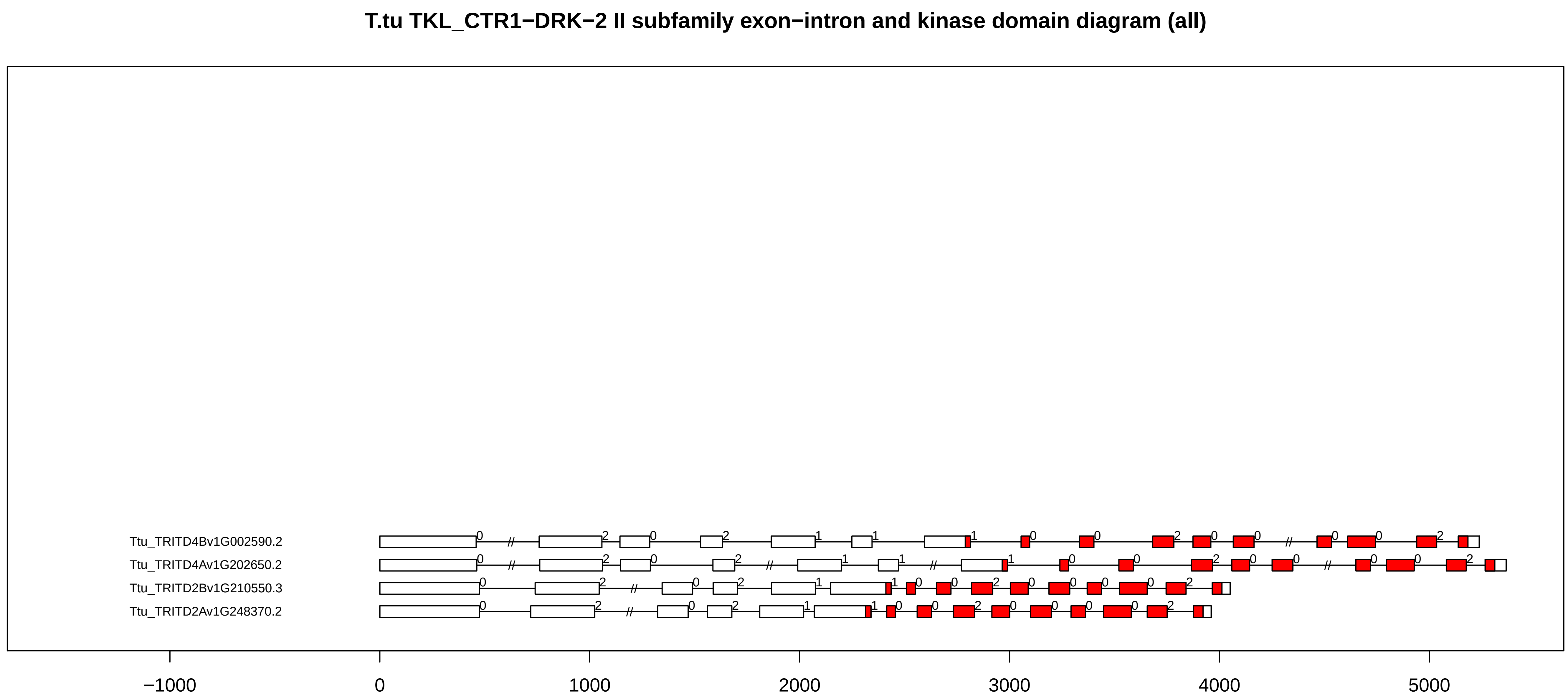
T.tu TKL_CTR1−DRK−2 II subfamily exon−intron and kinase domain diagram (all)
Ttu_TRITD4Bv1G002590.2
Ttu_TRITD4Av1G202650.2
Ttu_TRITD2Bv1G210550.3
Ttu_TRITD2Av1G248370.2
−1000
0
1000
2000
3000
4000
5000

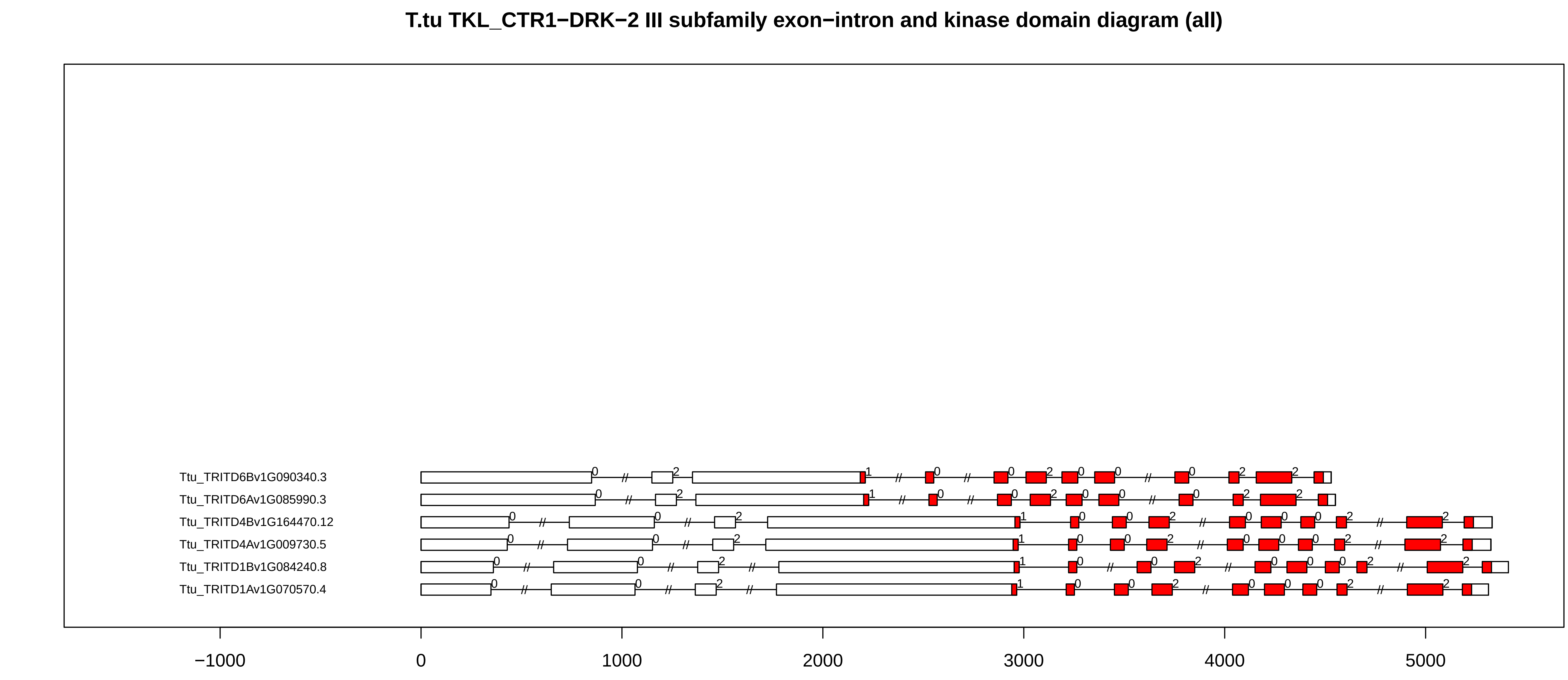

T.tu TKL_CTR1−DRK−2 III subfamily exon−intron and kinase domain diagram (all)
Ttu_TRITD6Bv1G090340.3
Ttu_TRITD6Av1G085990.3
Ttu_TRITD4Bv1G164470.12
Ttu_TRITD4Av1G009730.5
Ttu_TRITD1Bv1G084240.8
Ttu_TRITD1Av1G070570.4
−1000
0
1000
2000
3000
4000
5000

T.tu TKL_CTR1−DRK−2 IV subfamily exon−intron and kinase domain diagram (all)

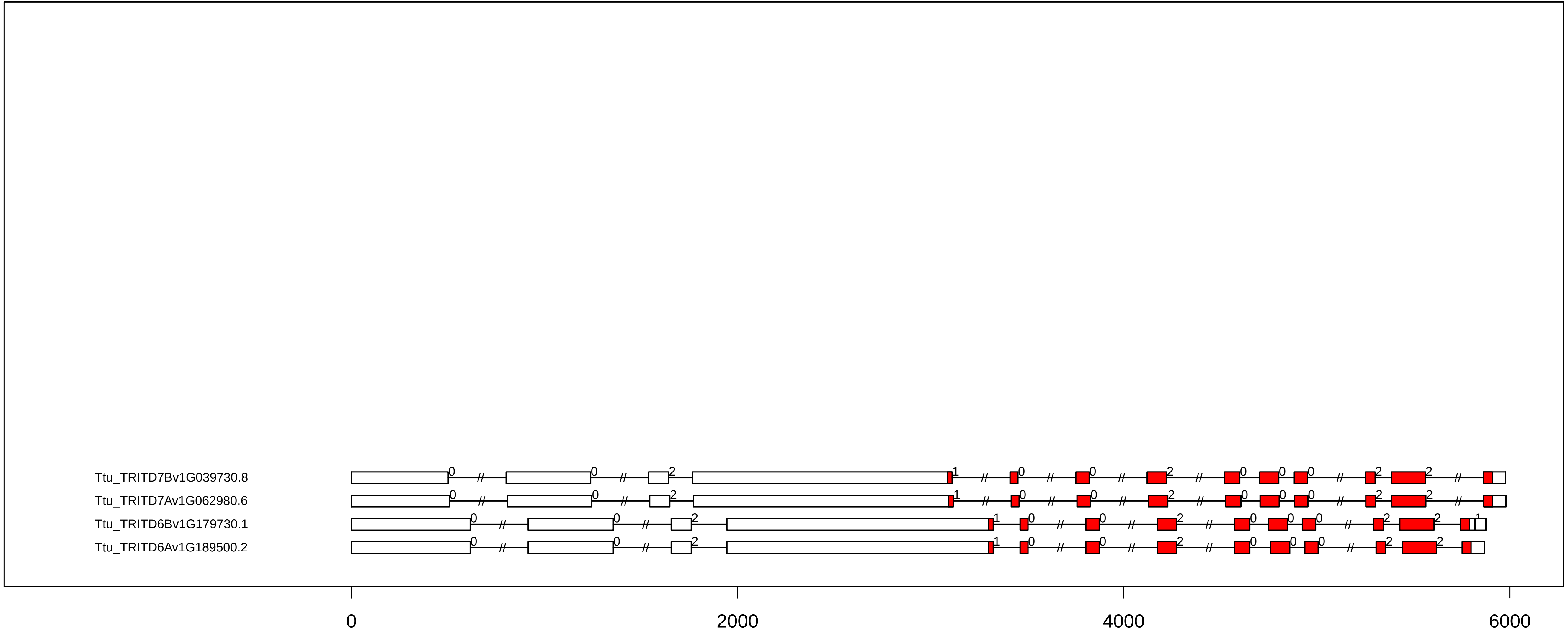

T.di TKL_CTR1−DRK−2 I subfamily exon−intron and kinase domain diagram (all)

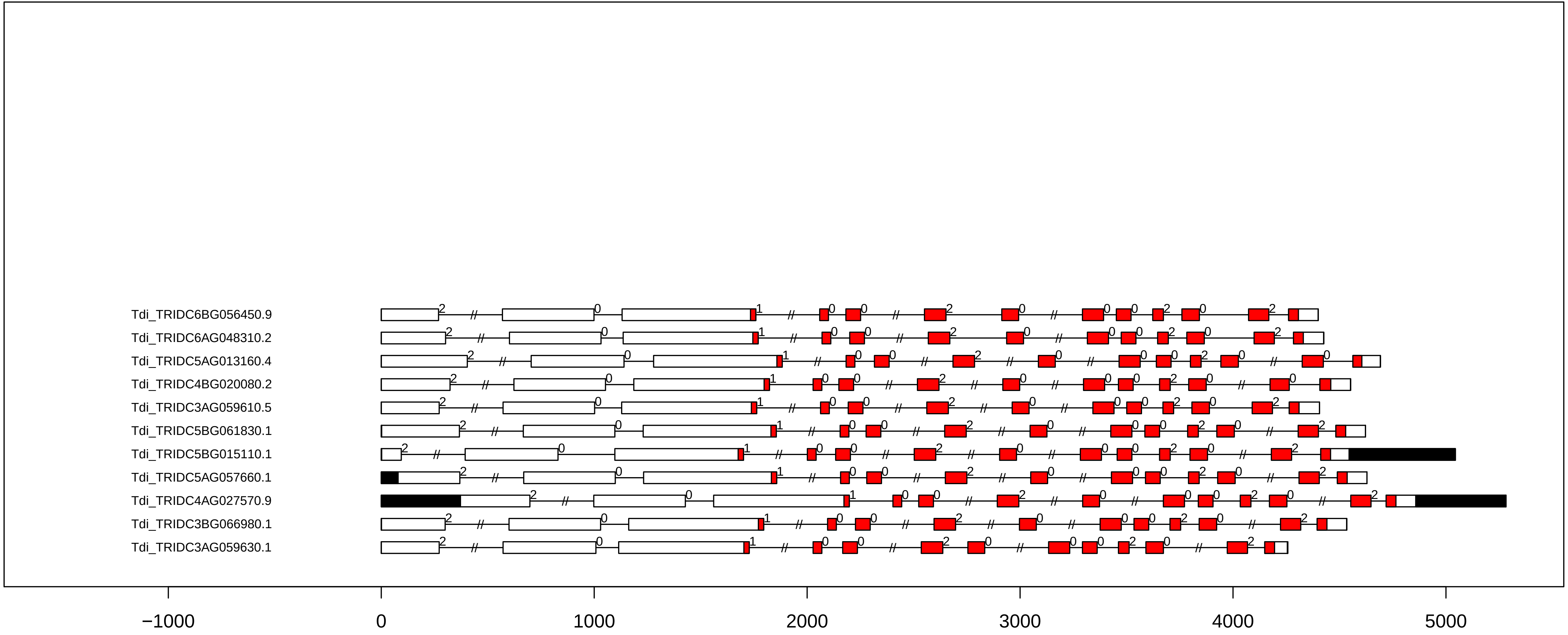

T.di TKL_CTR1−DRK−2 II subfamily exon−intron and kinase domain diagram (all)

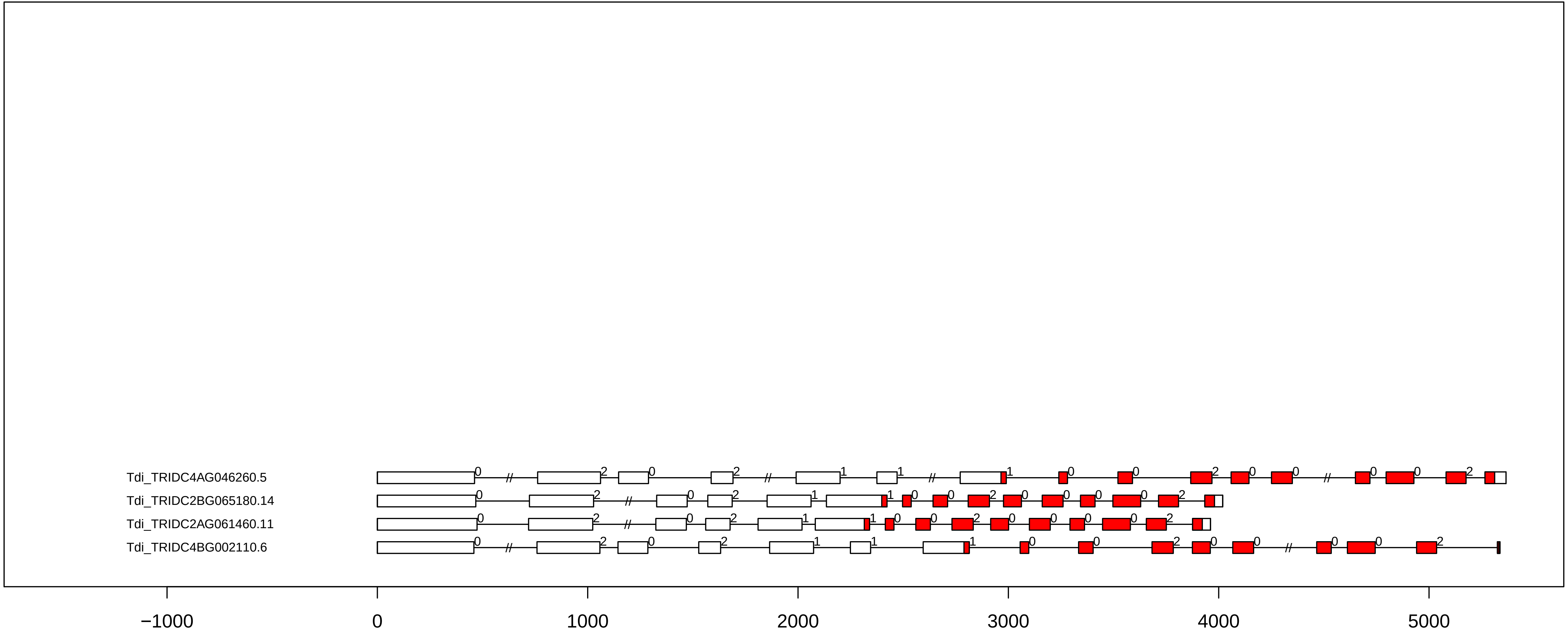

T.di TKL_CTR1−DRK−2 III subfamily exon−intron and kinase domain diagram (all)

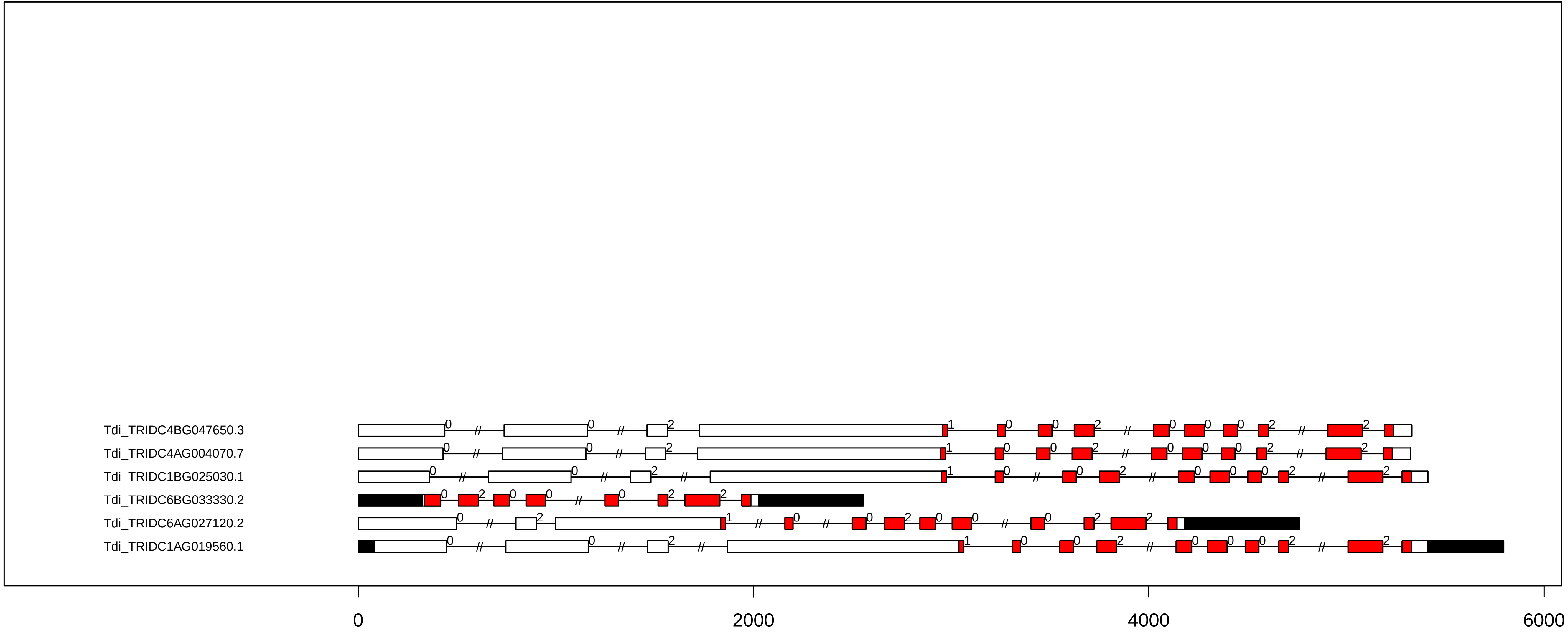

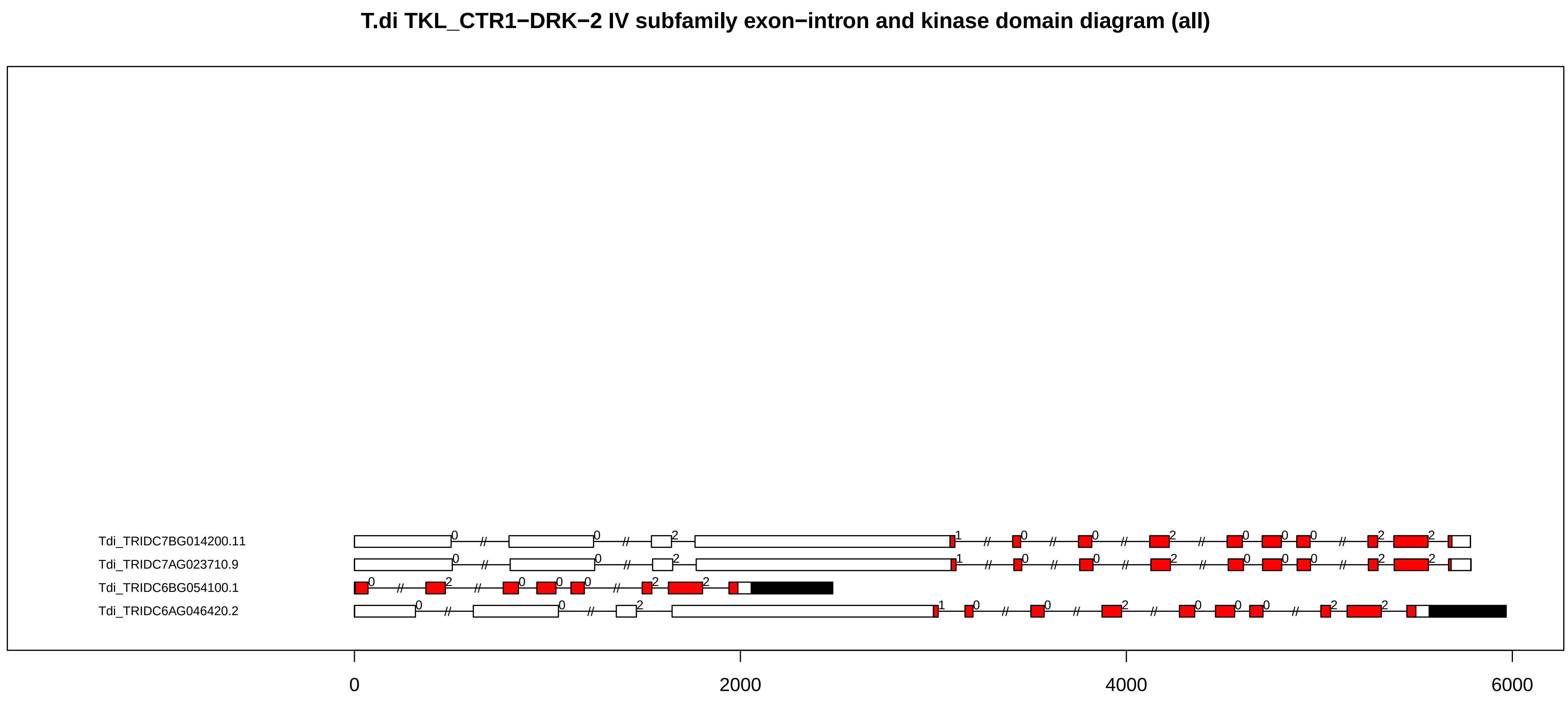

T.di TKL_CTR1−DRK−2 IV subfamily exon−intron and kinase domain diagram (all)
Tdi_TRIDC7BG014200.11
Tdi_TRIDC7AG023710.9
Tdi_TRIDC6BG054100.1
Tdi_TRIDC6AG046420.2
0
2000
4000
6000

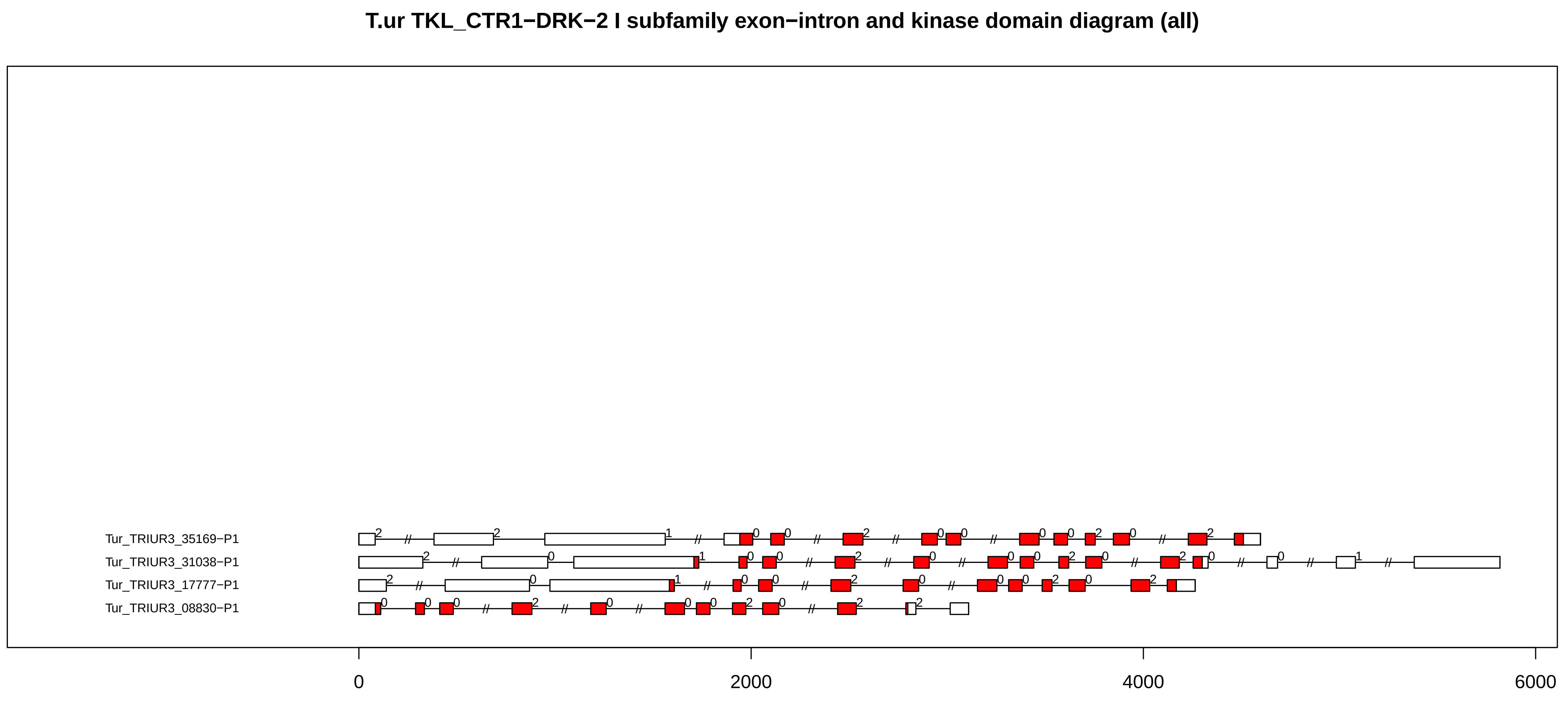

T.ur TKL_CTR1−DRK−2 I subfamily exon−intron and kinase domain diagram (all)
Tur_TRIUR3_35169−P1
Tur_TRIUR3_31038−P1
Tur_TRIUR3_17777−P1
Tur_TRIUR3_08830−P1
0
2000
4000
6000

T.ur TKL_CTR1−DRK−2 II subfamily exon−intron and kinase domain diagram (all)

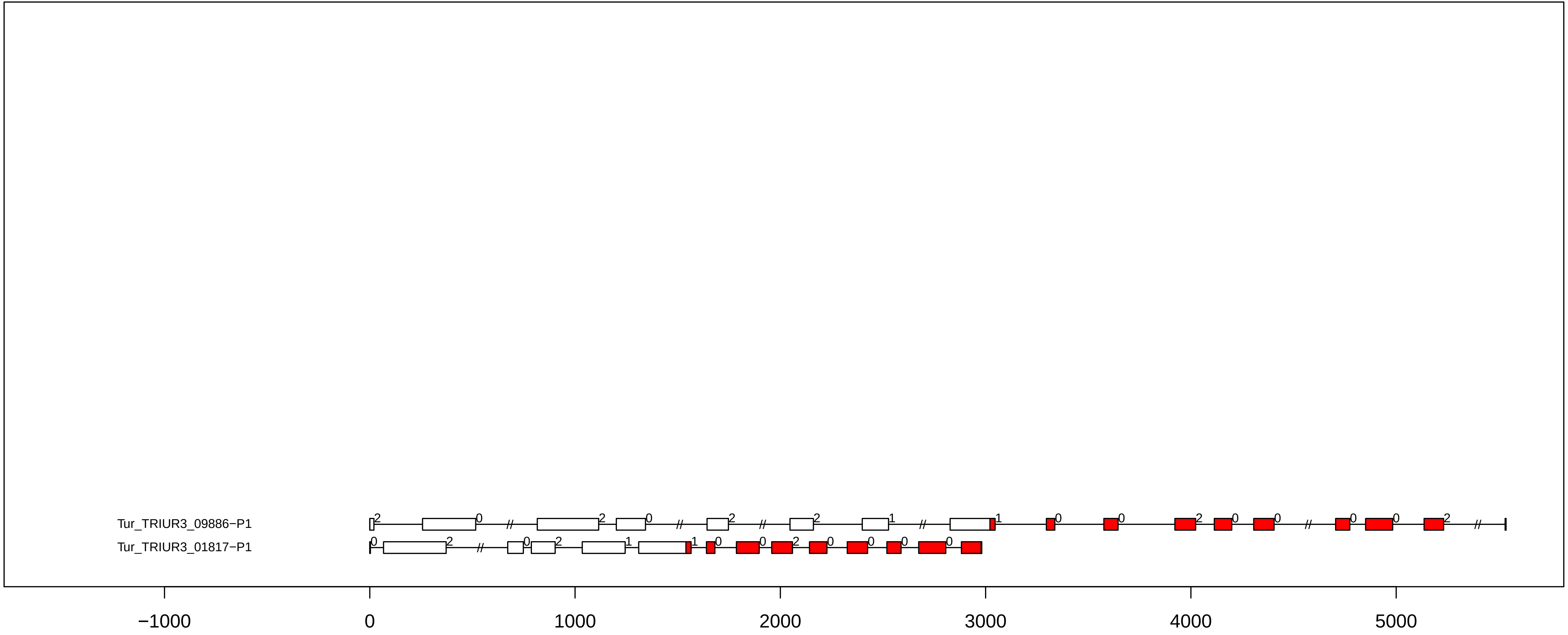

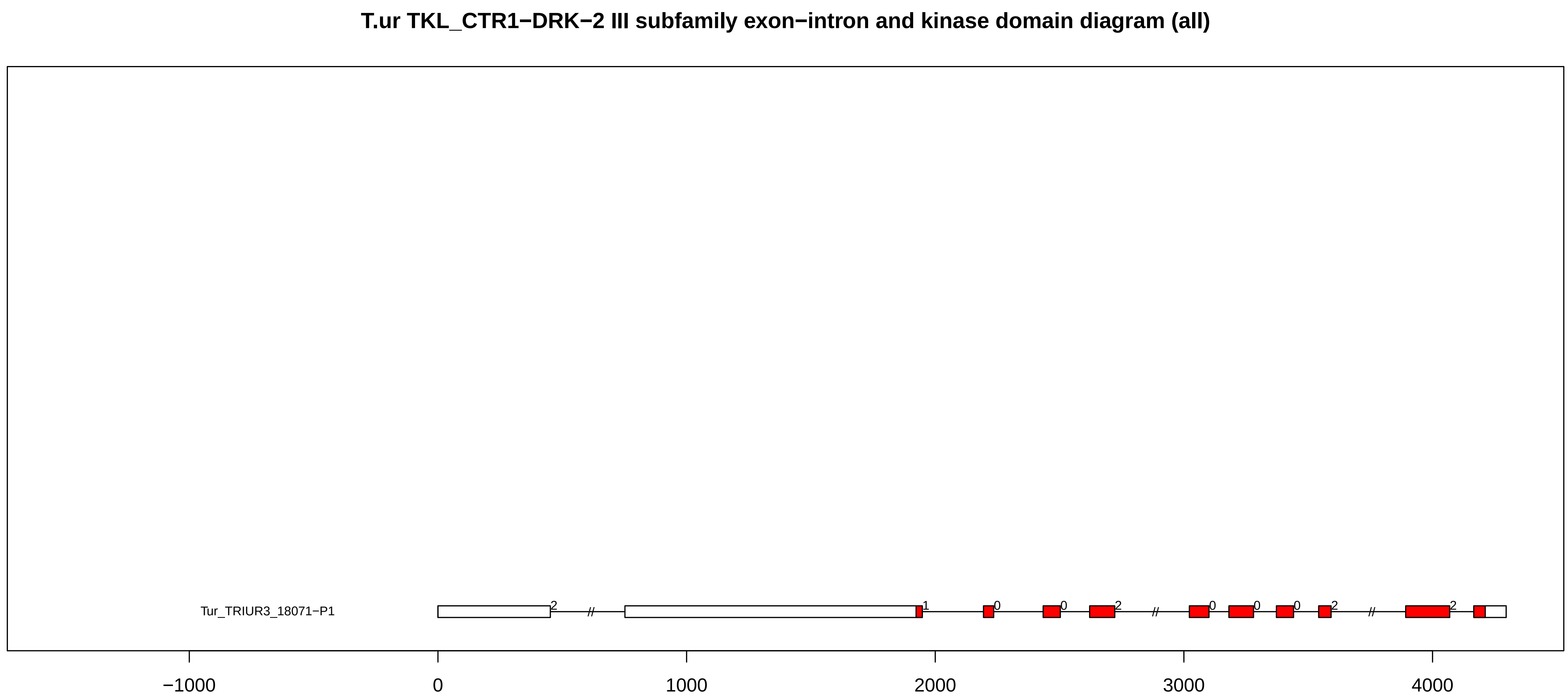
T.ur TKL_CTR1−DRK−2 III subfamily exon−intron and kinase domain diagram (all)
Tur_TRIUR3_18071−P1
2
1
0
0
2
0
0
0
2
2
−1000
0
1000
2000
3000
4000

T.ur TKL_CTR1−DRK−2 IV subfamily exon−intron and kinase domain diagram (all)

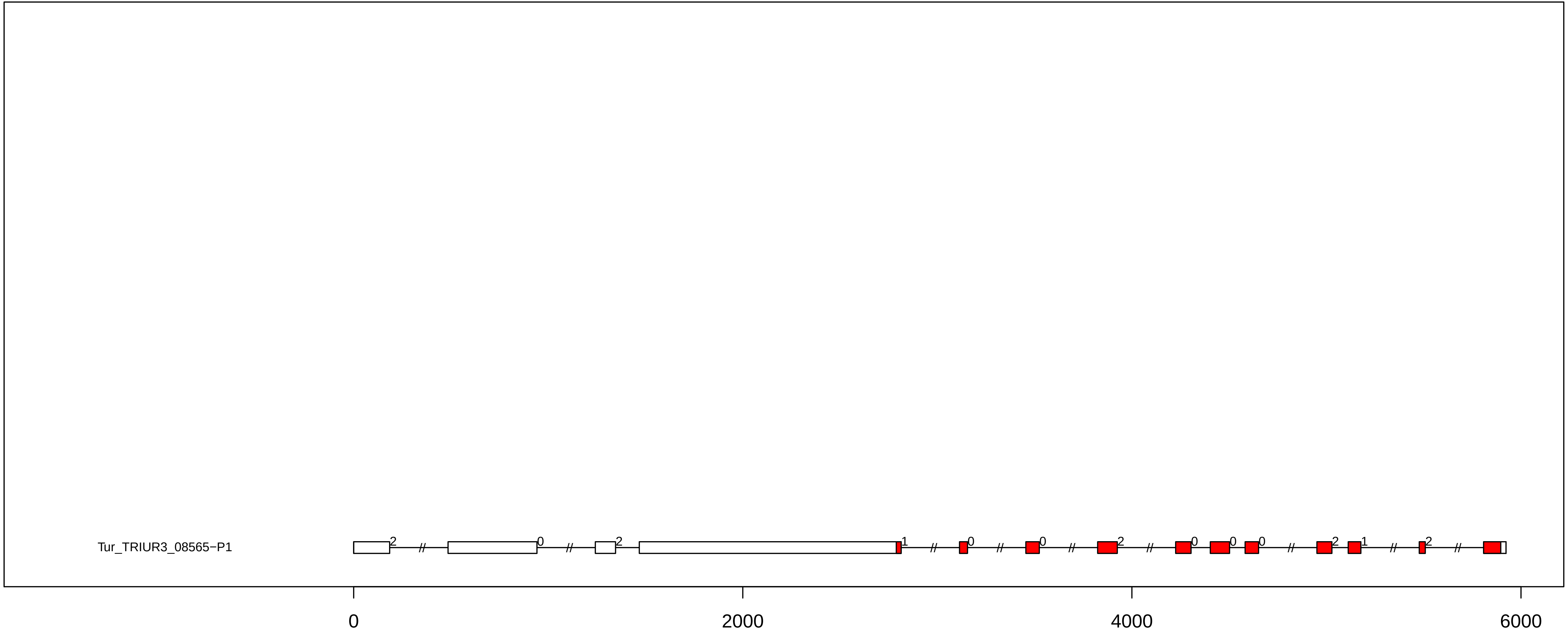

T.ur TKL_CTR1−DRK−2 (excluding in phylogenetic analysis) exon−intron and kinase domain diagram (all)

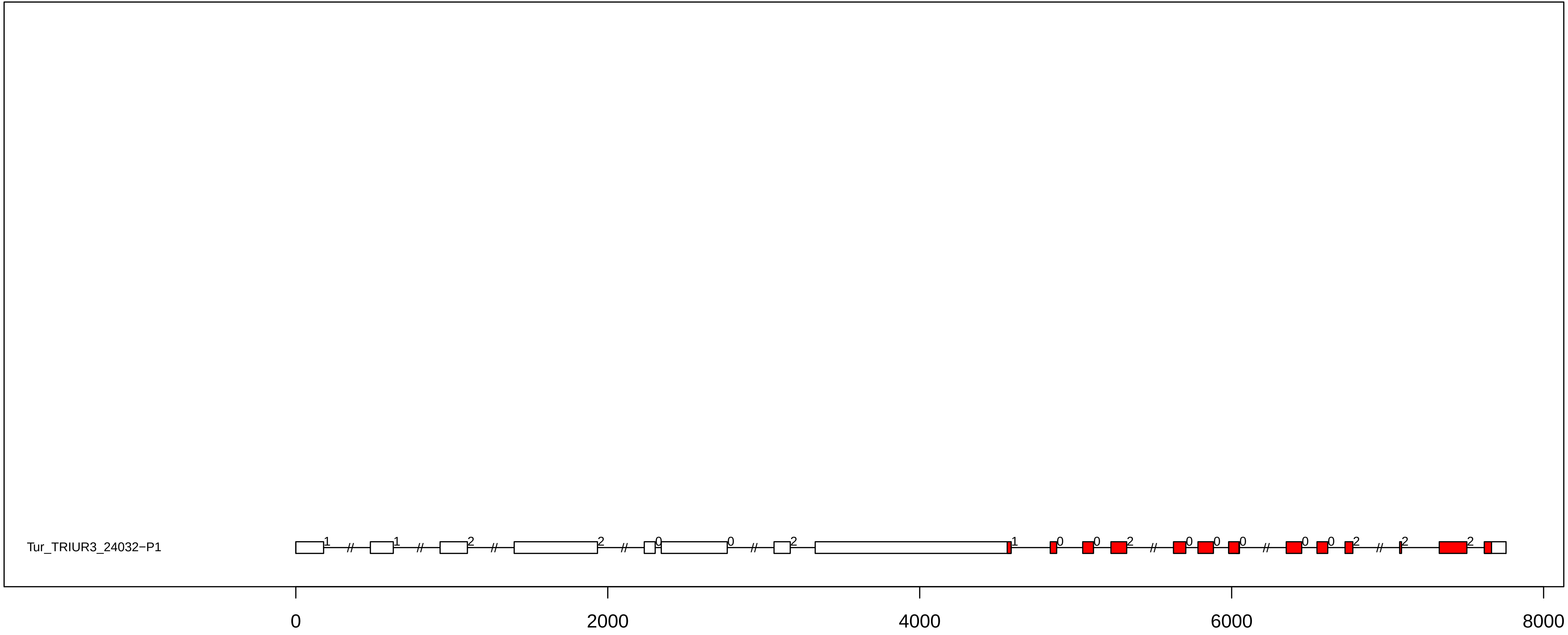

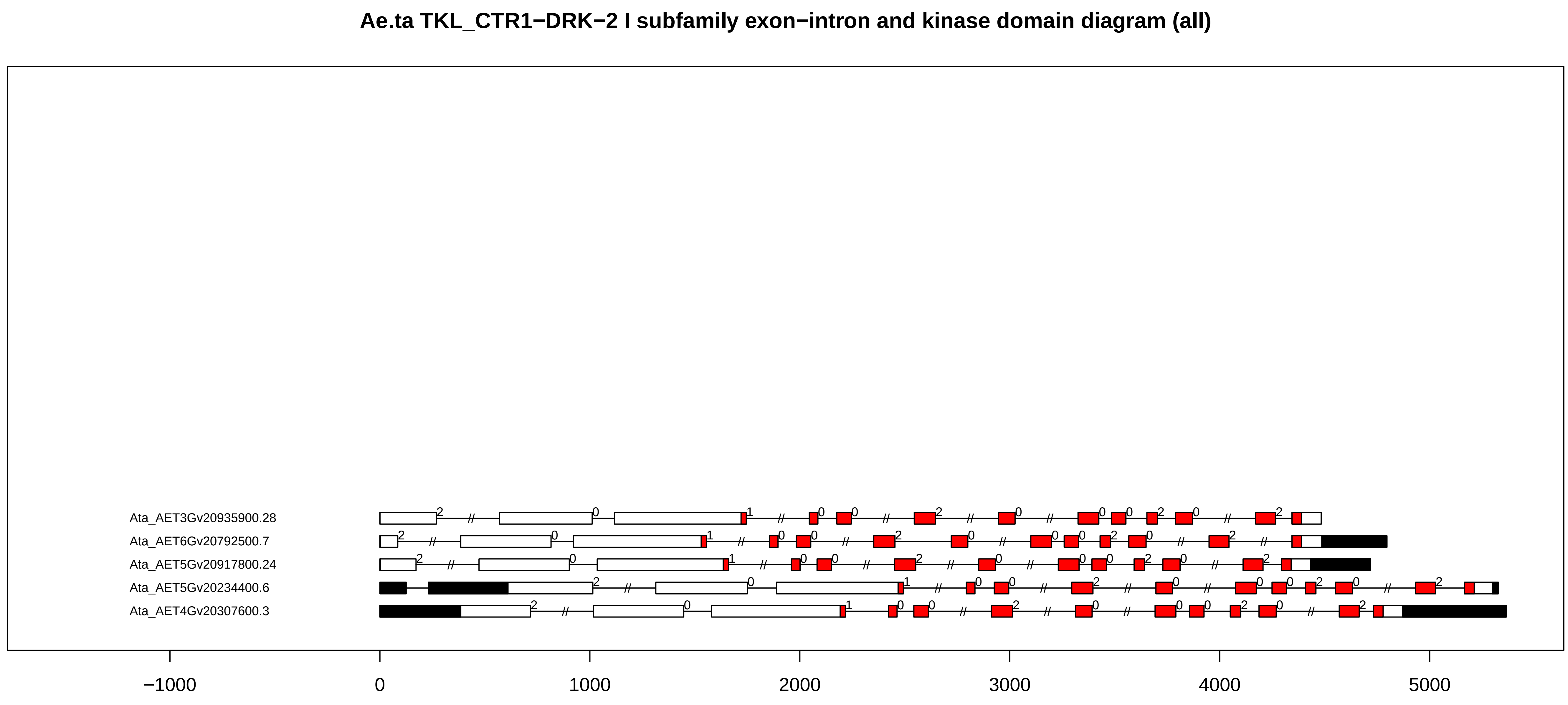

Ae.ta TKL_CTR1−DRK−2 I subfamily exon−intron and kinase domain diagram (all)
Ata_AET3Gv20935900.28
Ata_AET6Gv20792500.7
Ata_AET5Gv20917800.24
Ata_AET5Gv20234400.6
Ata_AET4Gv20307600.3
−1000
0
1000
2000
3000
4000
5000

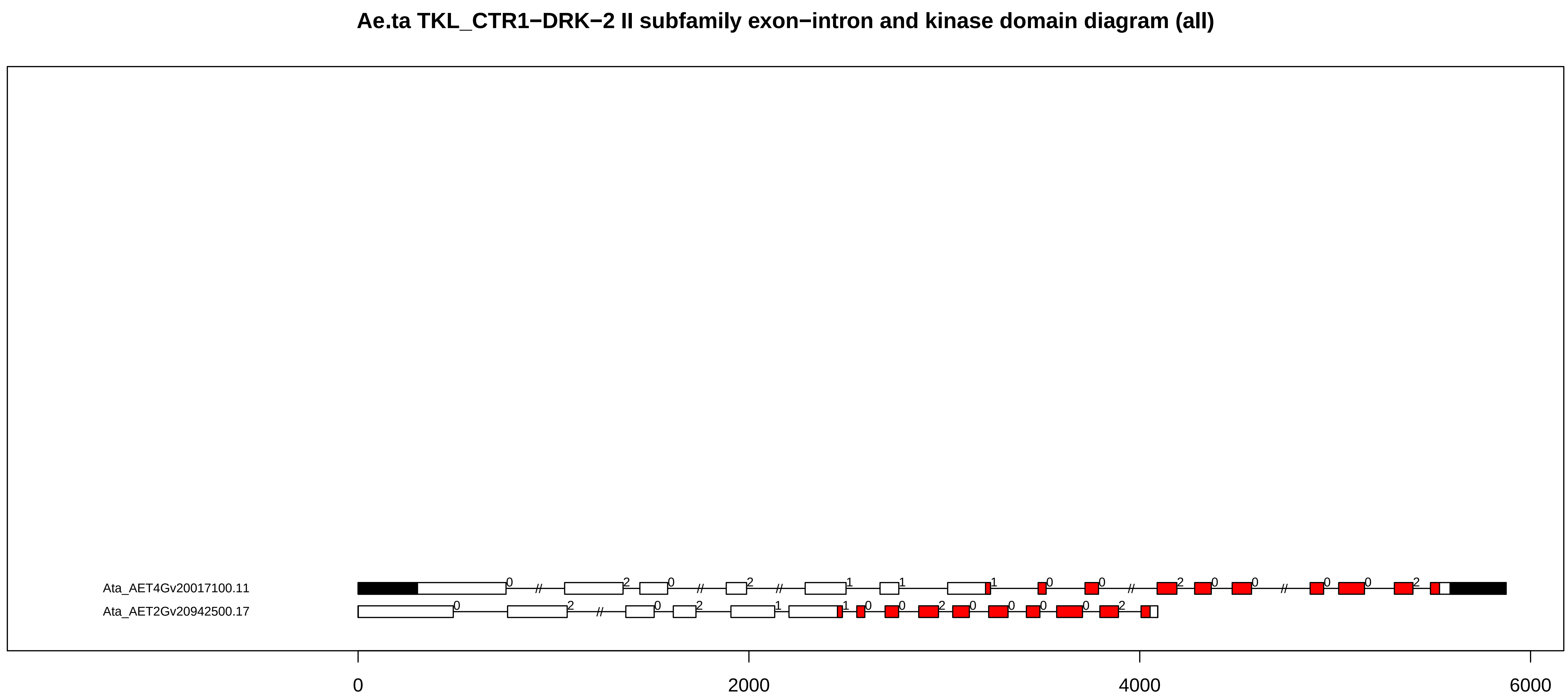

Ae.ta TKL_CTR1−DRK−2 II subfamily exon−intron and kinase domain diagram (all)
Ata_AET4Gv20017100.11
Ata_AET2Gv20942500.17
0
2000
4000
6000

Ae.ta TKL_CTR1−DRK−2 III subfamily exon−intron and kinase domain diagram (all)

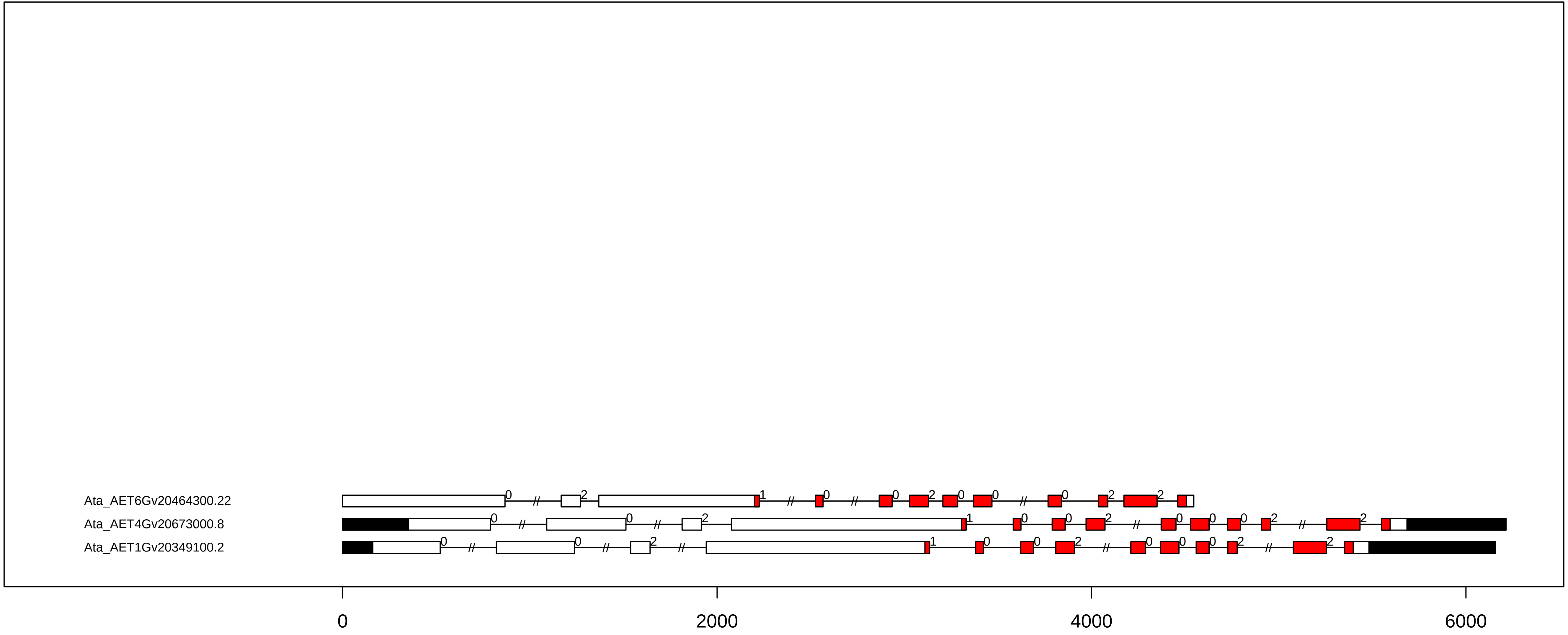

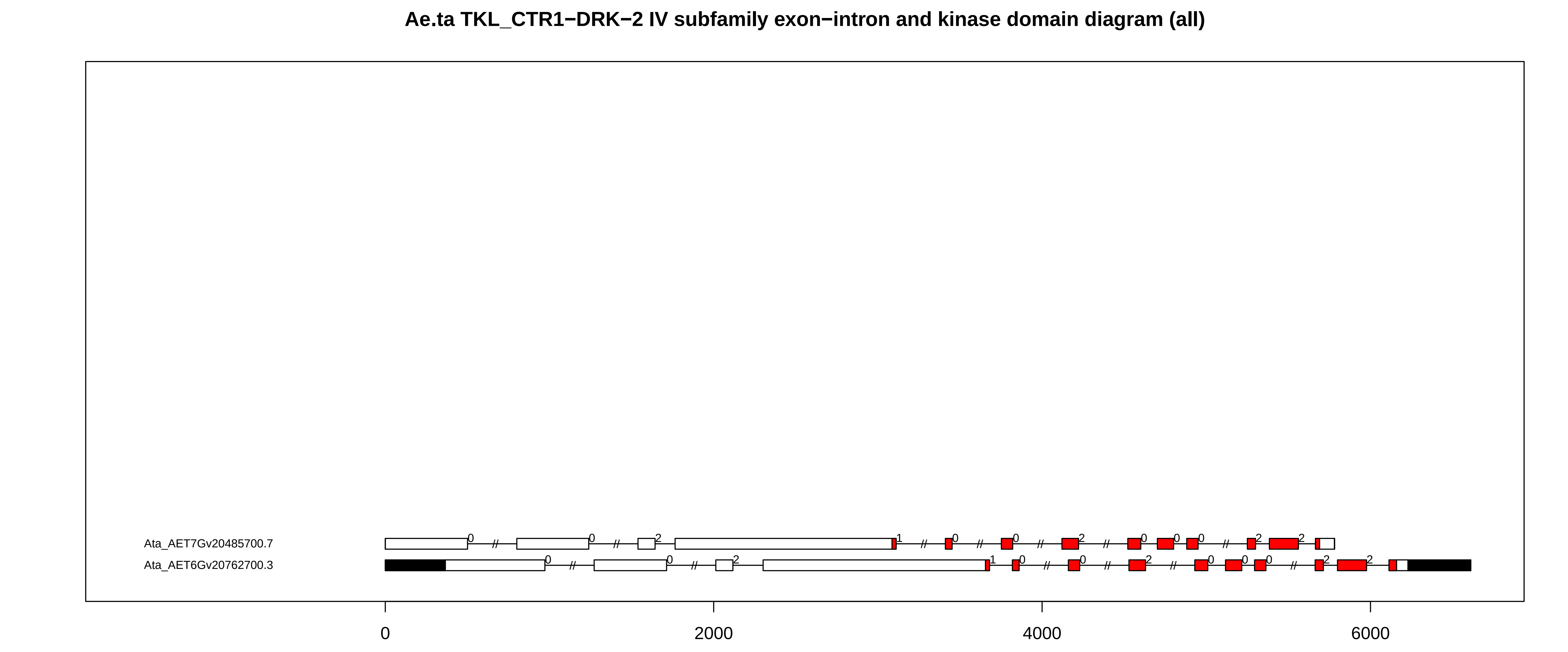

Ae.ta TKL_CTR1−DRK−2 IV subfamily exon−intron and kinase domain diagram (all)
Ata_AET7Gv20485700.7
Ata_AET6Gv20762700.3
0
2000
4000
6000

B.di TKL_CTR1−DRK−2 I subfamily exon−intron and kinase domain diagram (all)

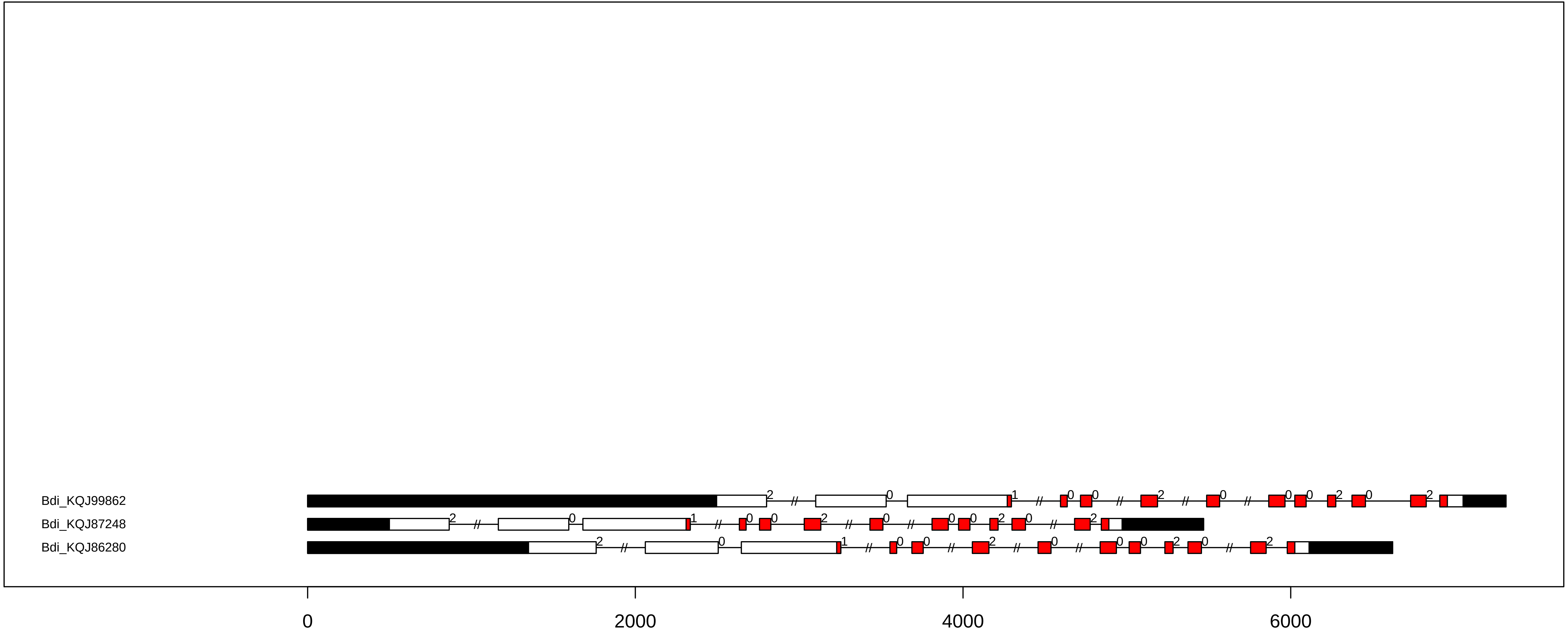

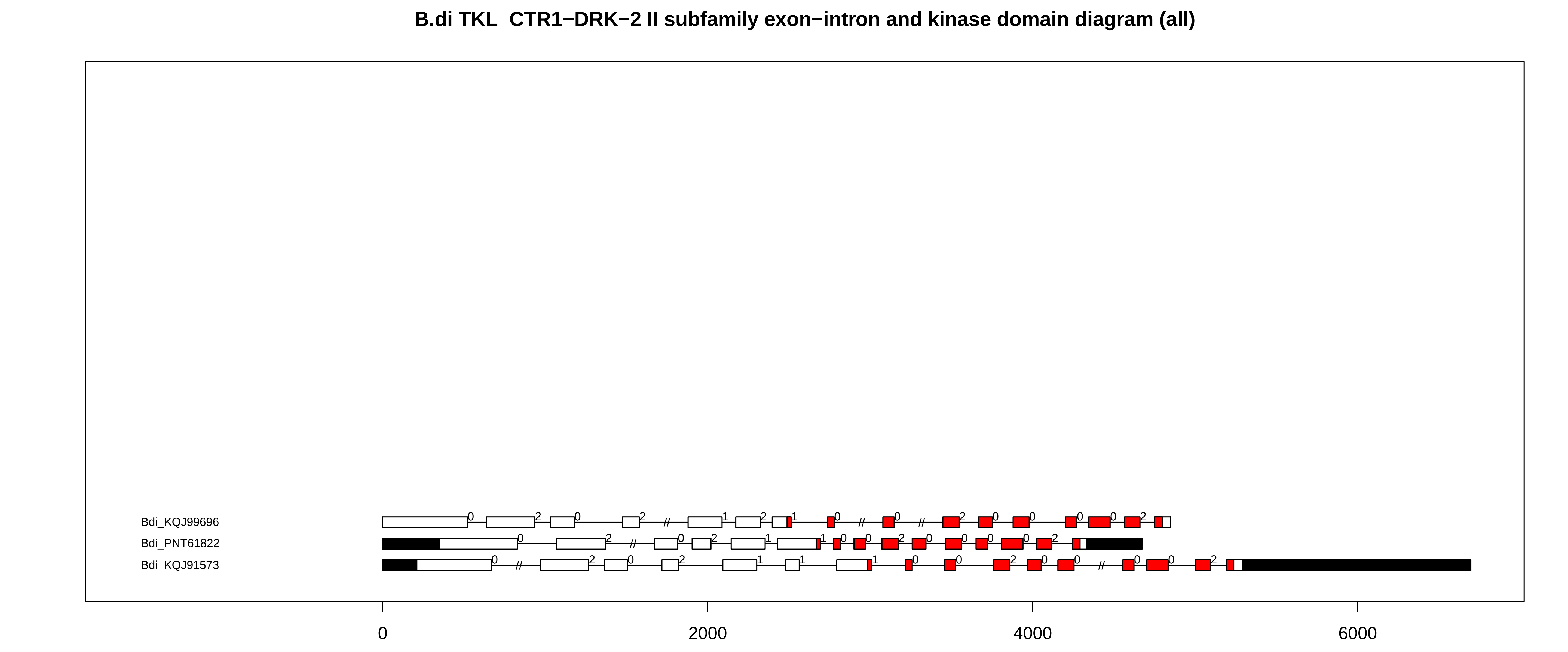

B.di TKL_CTR1−DRK−2 II subfamily exon−intron and kinase domain diagram (all)
Bdi_KQJ99696
Bdi_PNT61822
Bdi_KQJ91573
0
2000
4000
6000

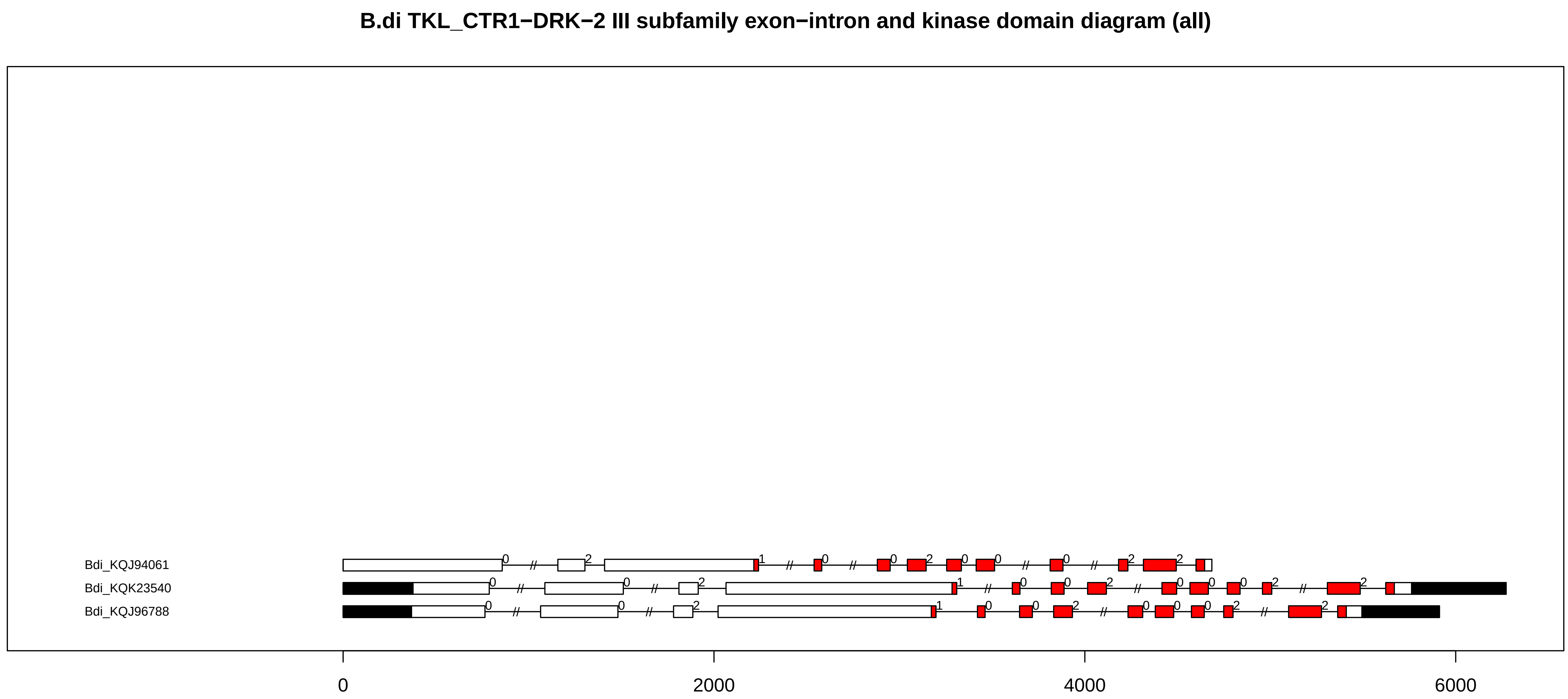

B.di TKL_CTR1−DRK−2 III subfamily exon−intron and kinase domain diagram (all)
Bdi_KQJ94061
Bdi_KQK23540
Bdi_KQJ96788
0
2000
4000
6000

B.di TKL_CTR1−DRK−2 IV subfamily exon−intron and kinase domain diagram (all)

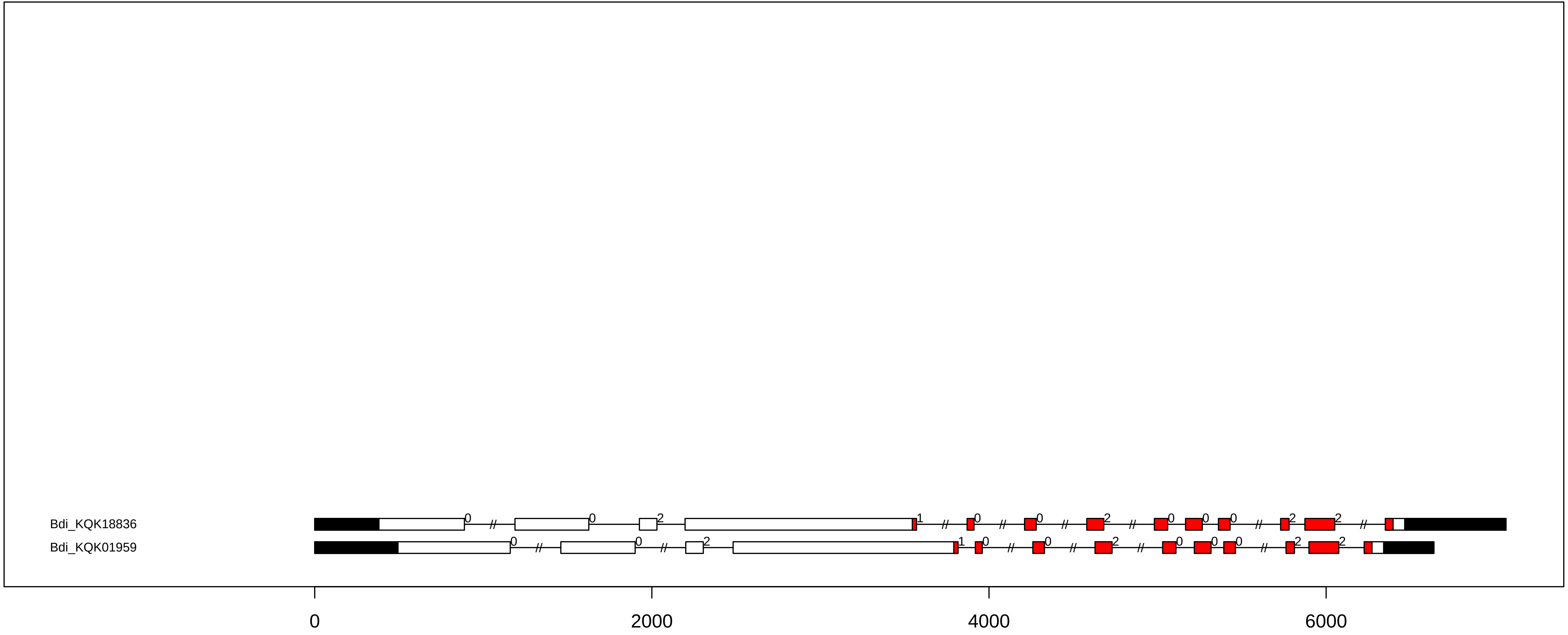

Z.ma TKL_CTR1−DRK−2 I subfamily exon−intron and kinase domain diagram (all)

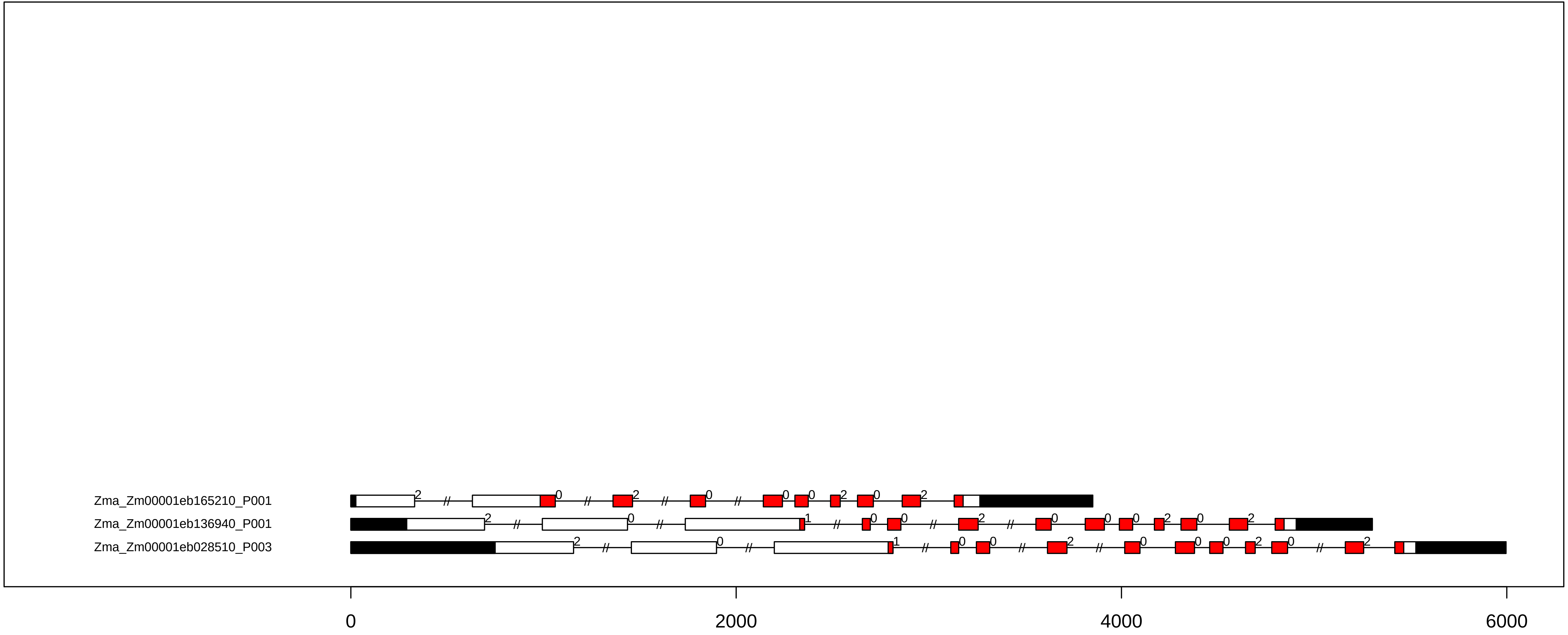

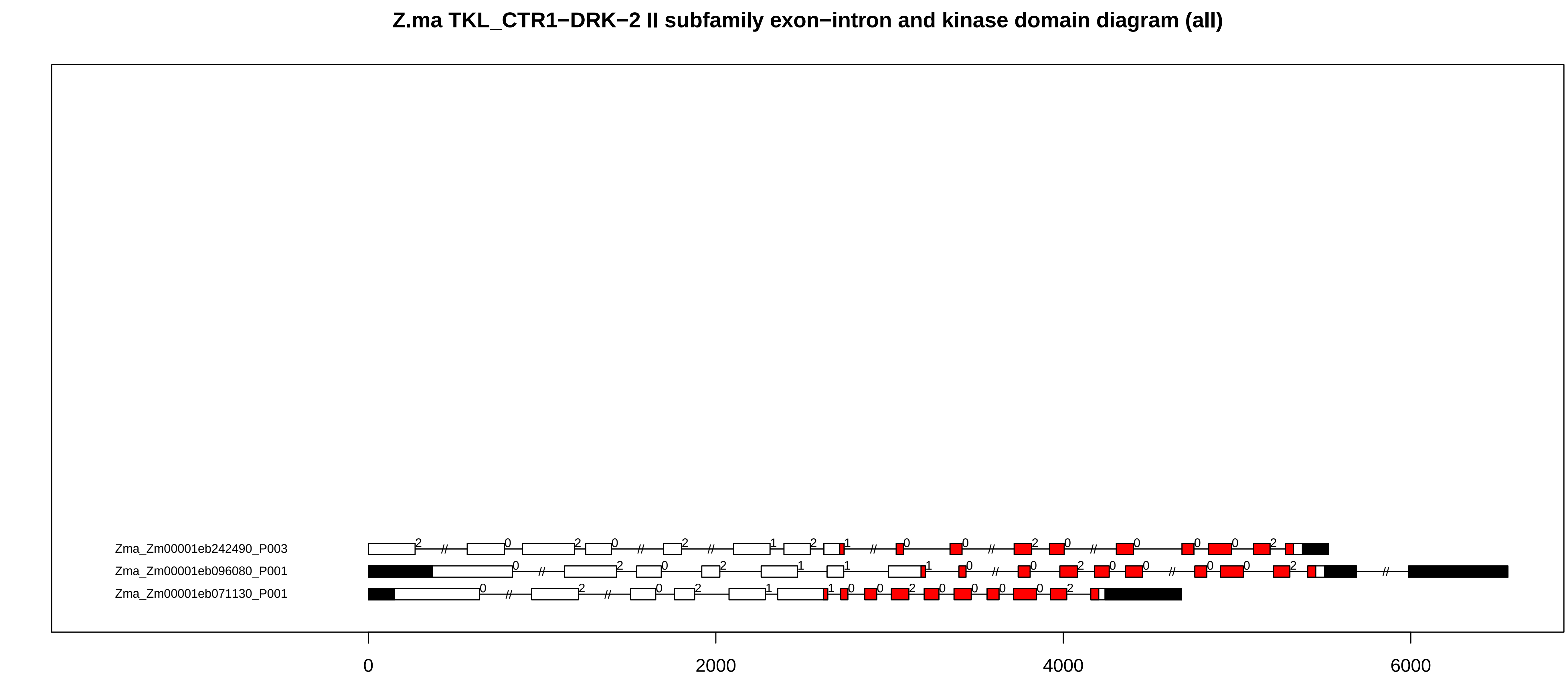
Z.ma TKL_CTR1−DRK−2 II subfamily exon−intron and kinase domain diagram (all)
Zma_Zm00001eb242490_P003
Zma_Zm00001eb096080_P001
Zma_Zm00001eb071130_P001
0
2000
4000
6000

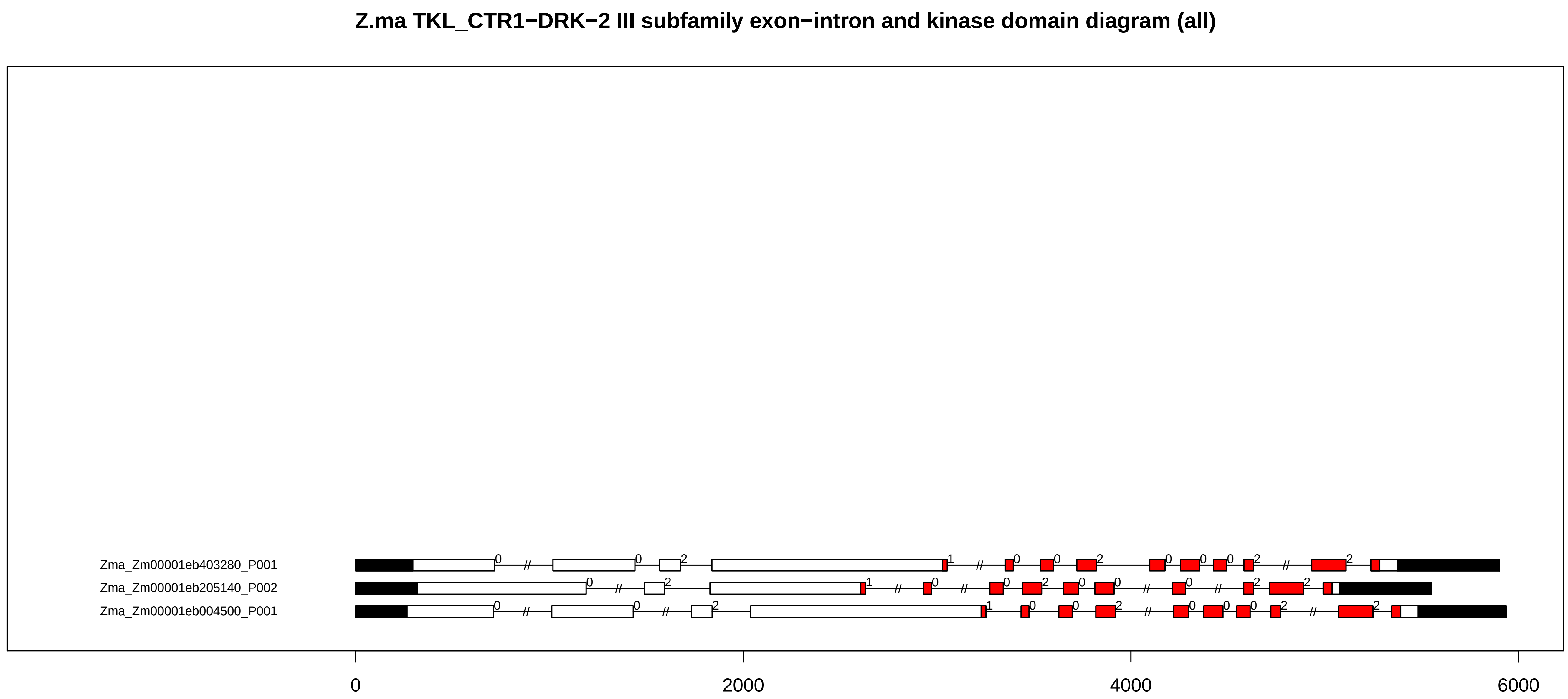
Z.ma TKL_CTR1−DRK−2 III subfamily exon−intron and kinase domain diagram (all)
Zma_Zm00001eb403280_P001
Zma_Zm00001eb205140_P002
Zma_Zm00001eb004500_P001
0
2000
4000
6000

Z.ma TKL_CTR1−DRK−2 IV subfamily exon−intron and kinase domain diagram (all)

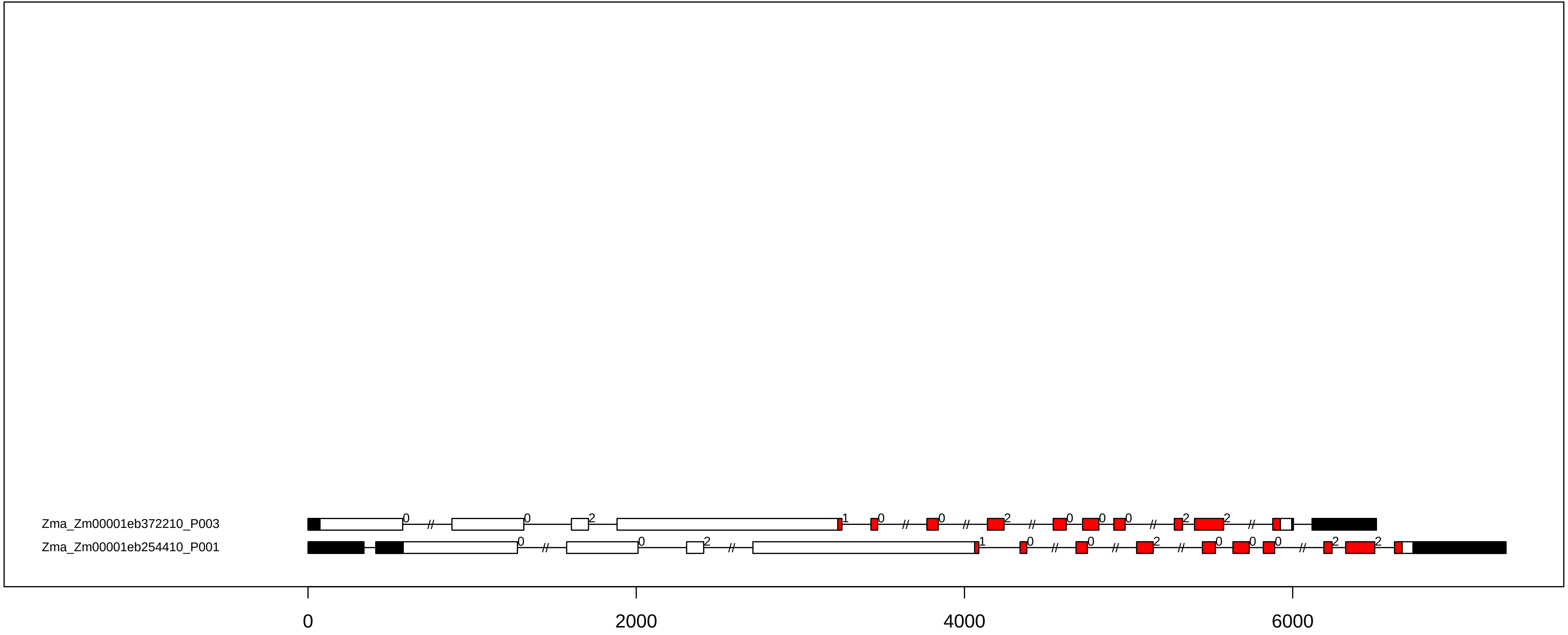

O.sa TKL_CTR1−DRK−2 II subfamily exon−intron and kinase domain diagram (all)

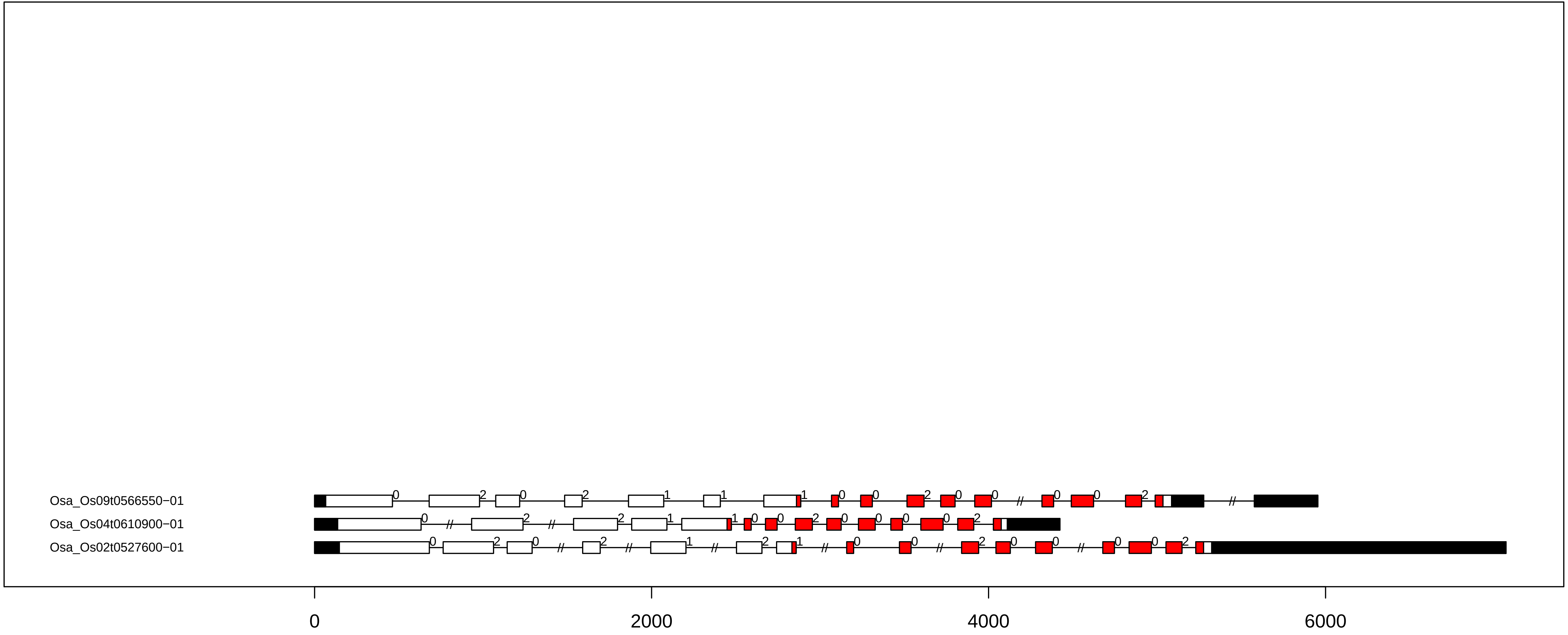

O.sa TKL_CTR1−DRK−2 III subfamily exon−intron and kinase domain diagram (all)

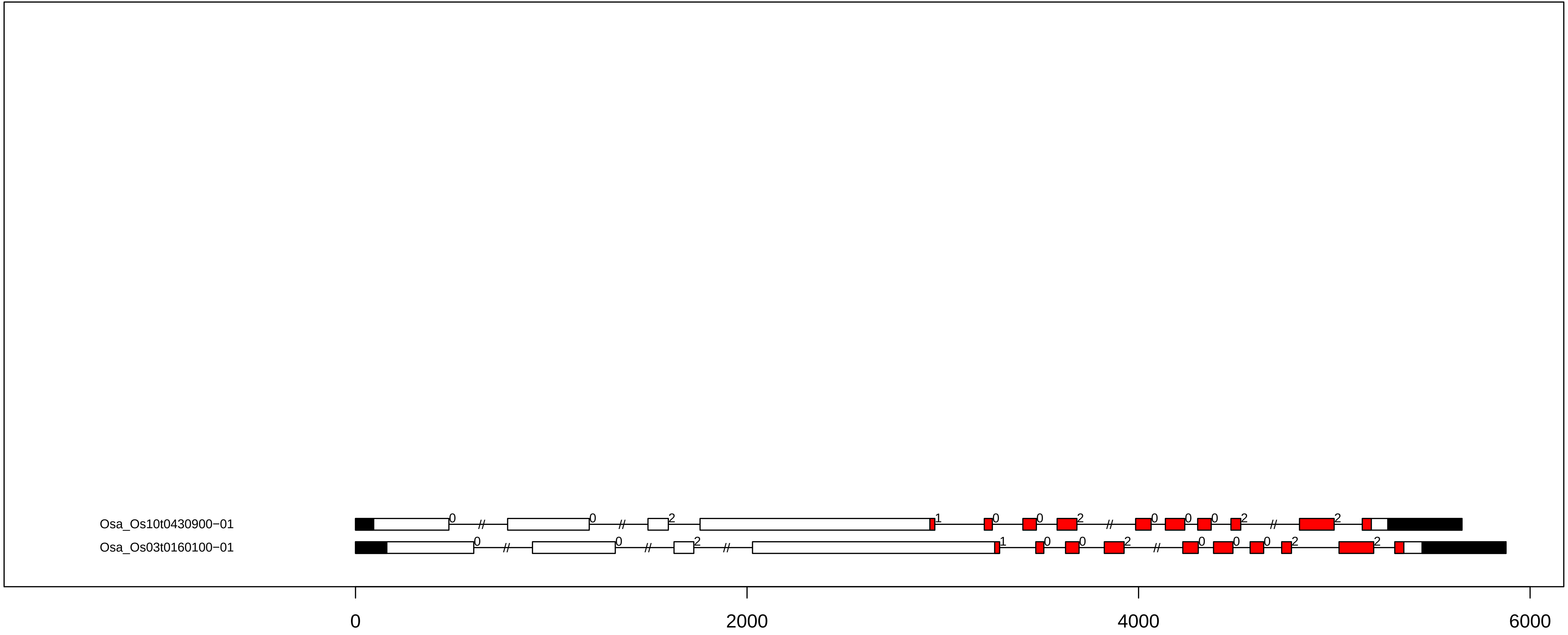

O.sa TKL_CTR1−DRK−2 IV subfamily exon−intron and kinase domain diagram (all)

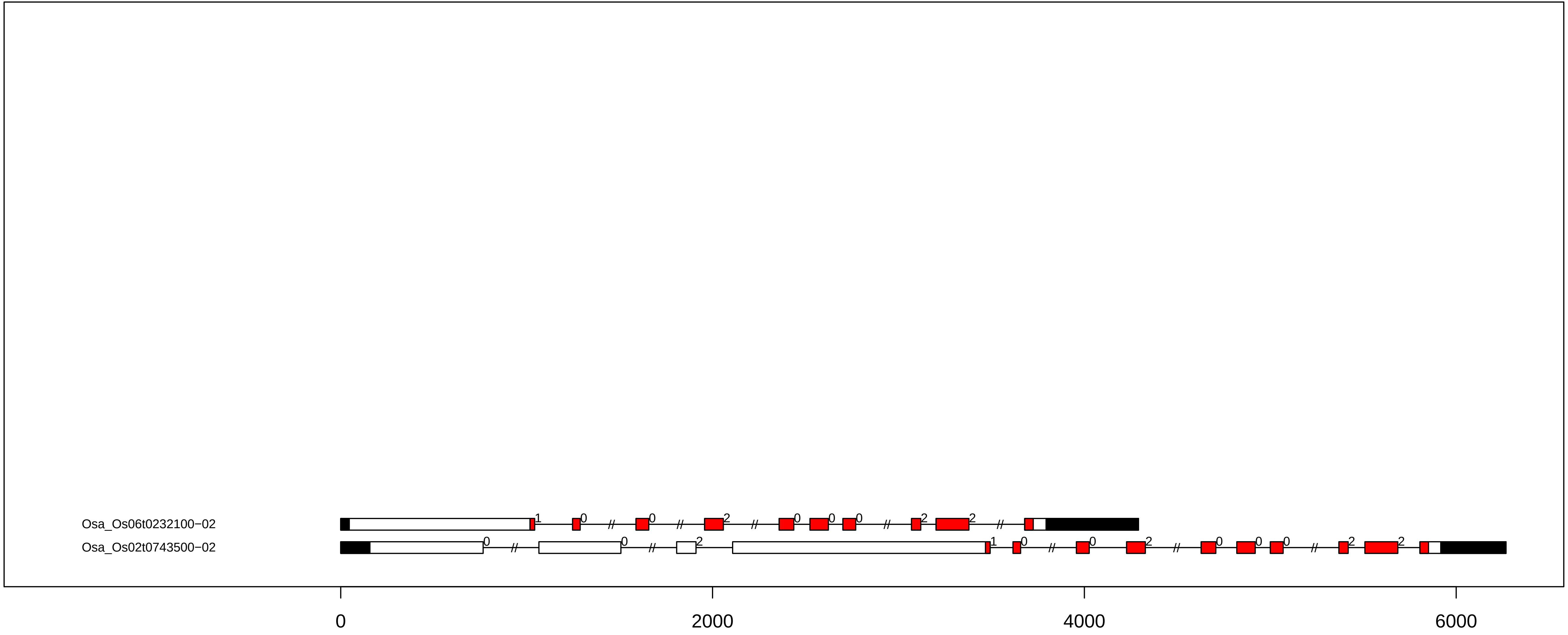

O.sa TKL_CTR1−DRK−2 (excluding in phylogenetic analysis) exon−intron and kinase domain diagram (all)

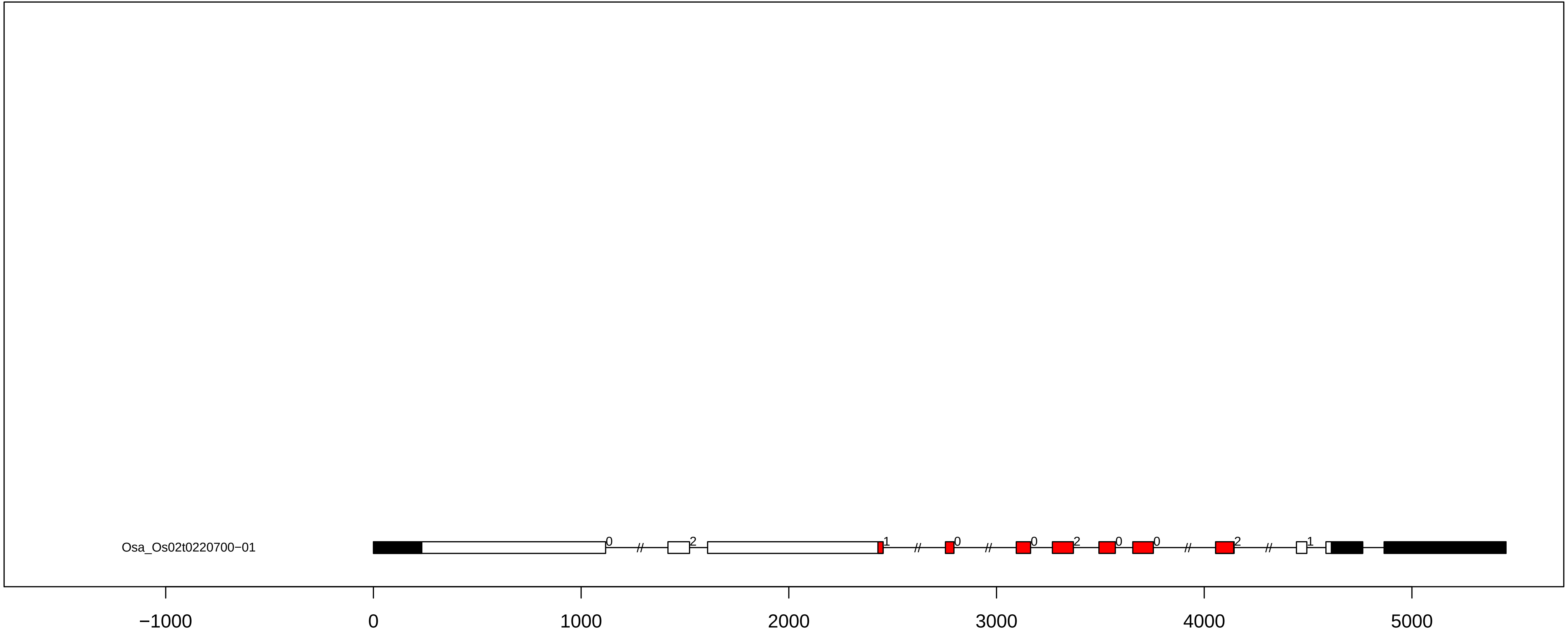

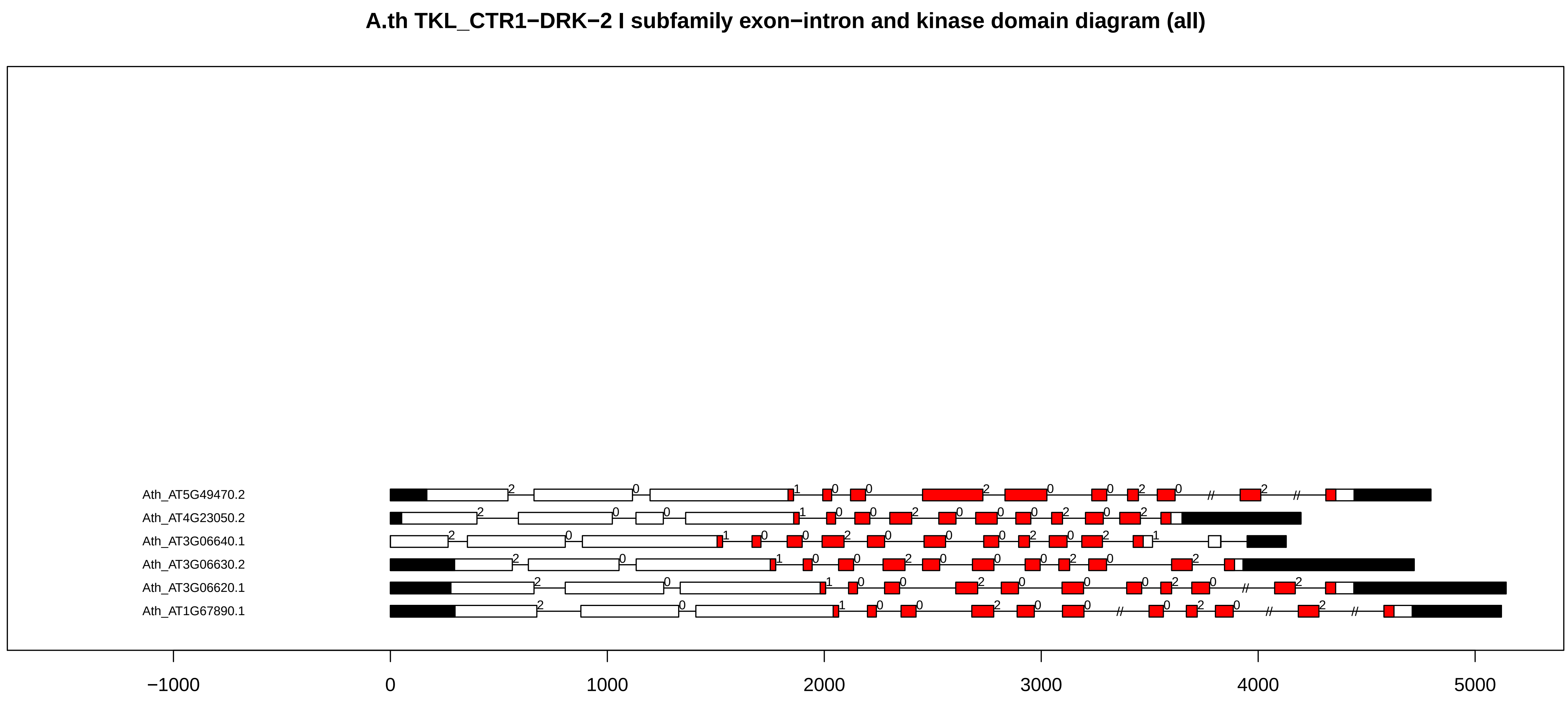

A.th TKL_CTR1−DRK−2 I subfamily exon−intron and kinase domain diagram (all)
Ath_AT5G49470.2
Ath_AT4G23050.2
Ath_AT3G06640.1
Ath_AT3G06630.2
Ath_AT3G06620.1
Ath_AT1G67890.1
−1000
0
1000
2000
3000
4000
5000

A.th TKL_CTR1−DRK−2 II subfamily exon−intron and kinase domain diagram (all)

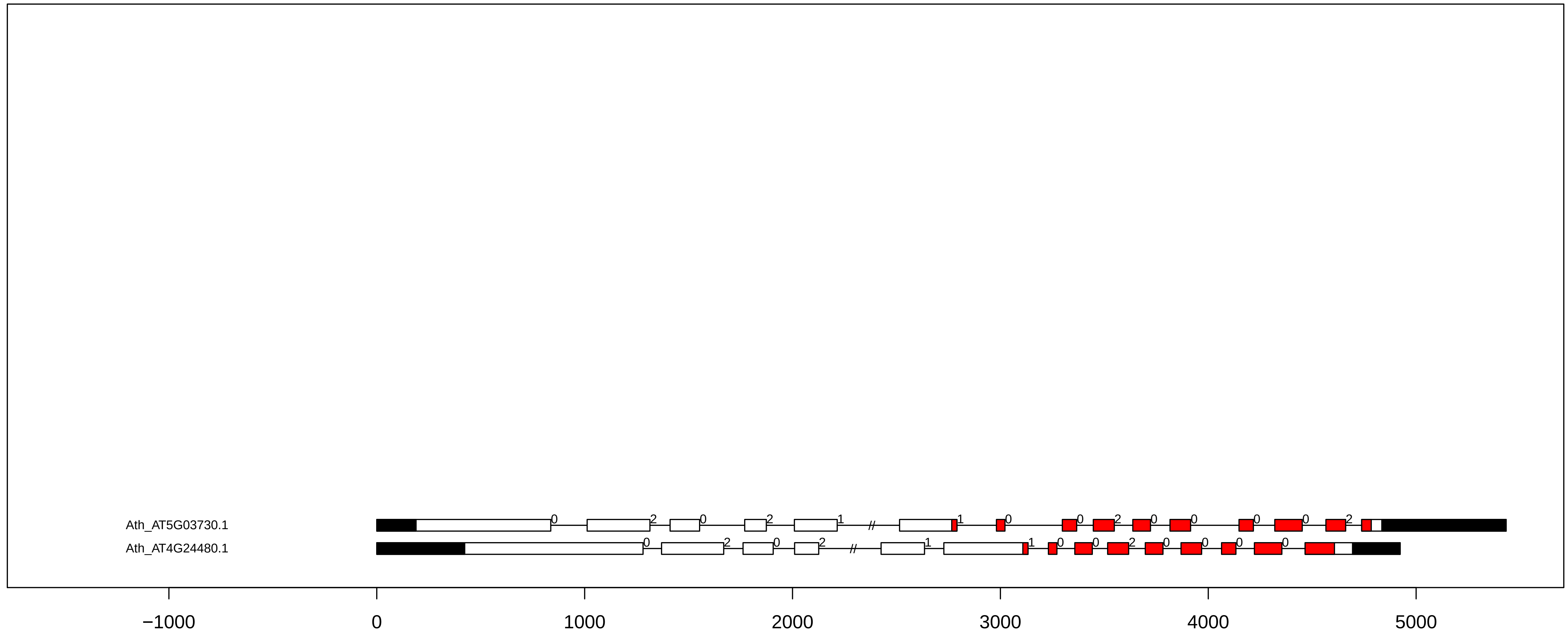

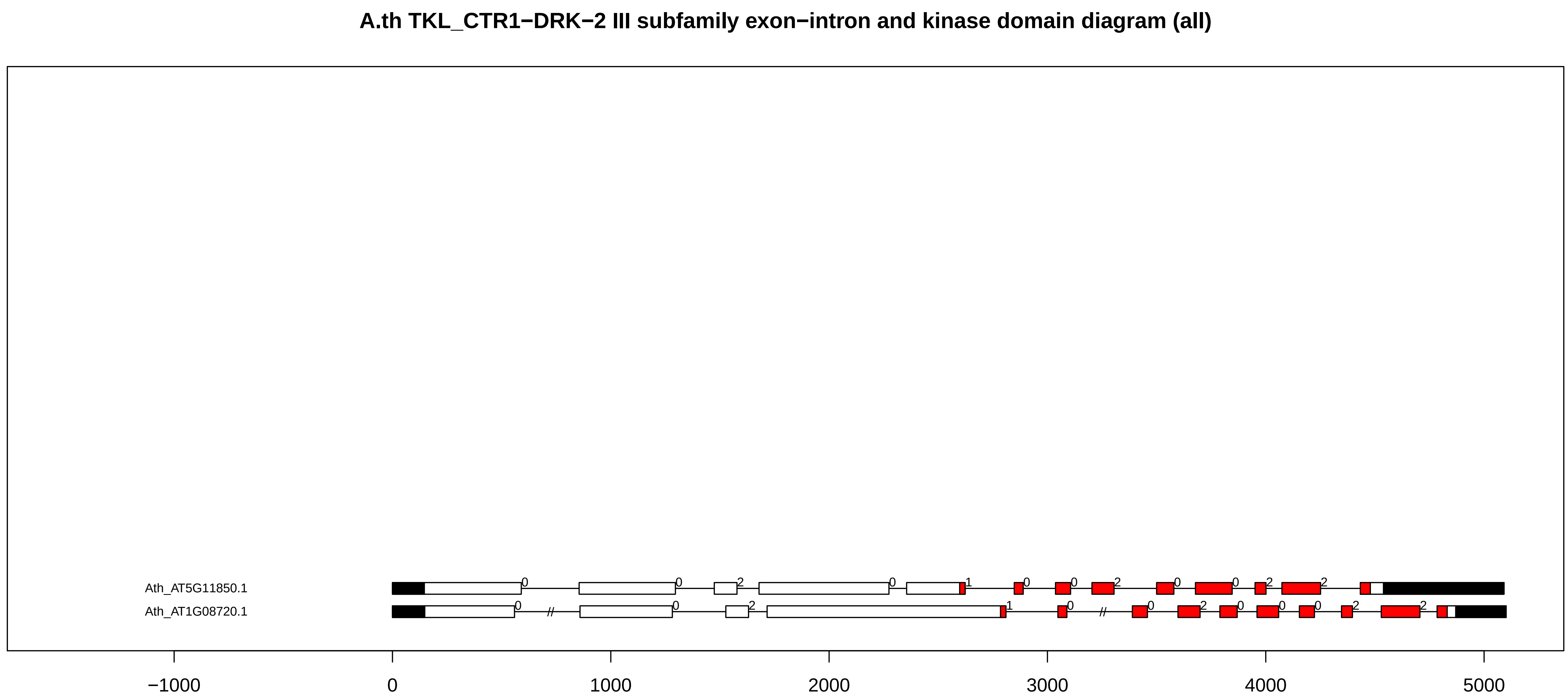
A.th TKL_CTR1−DRK−2 III subfamily exon−intron and kinase domain diagram (all)
Ath_AT5G11850.1
Ath_AT1G08720.1
−1000
0
1000
2000
3000
4000
5000

A.th TKL_CTR1−DRK−2 IV subfamily exon−intron and kinase domain diagram (all)

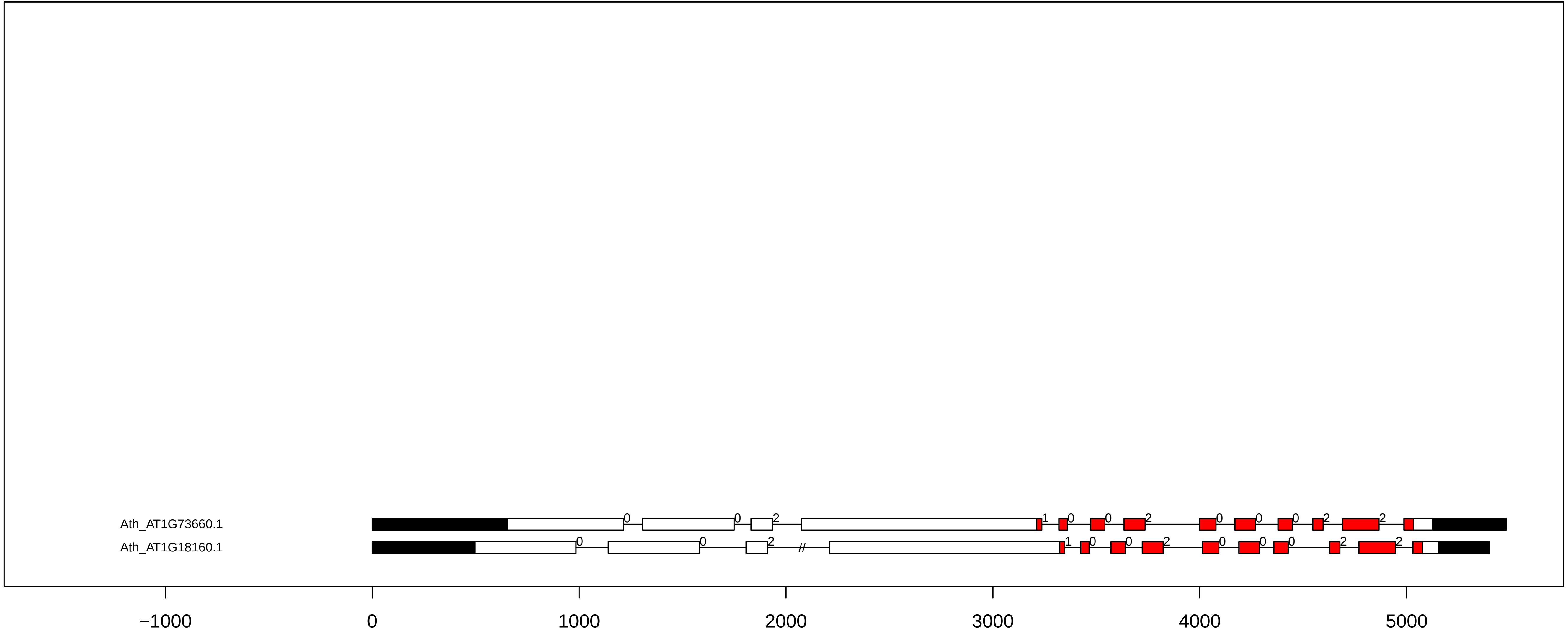

V.vi TKL_CTR1−DRK−2 I subfamily exon−intron and kinase domain diagram (all)

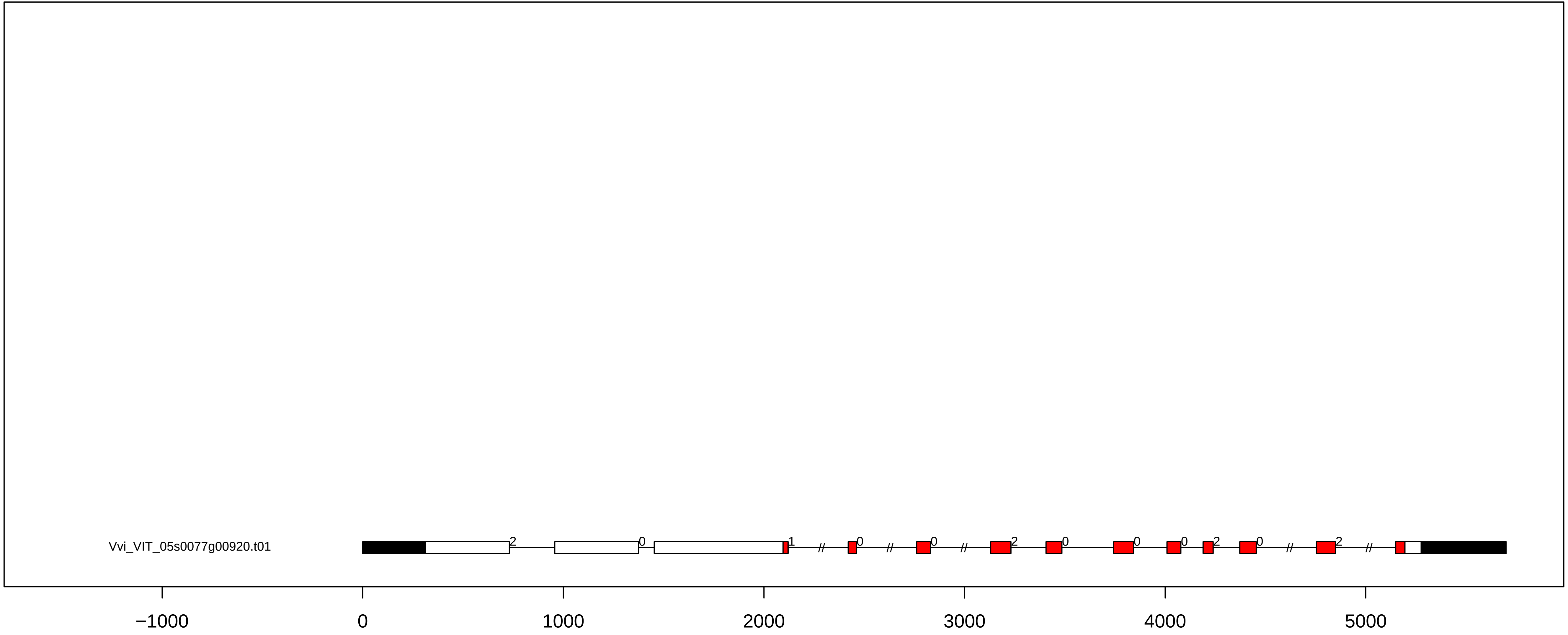

V.vi TKL_CTR1−DRK−2 II subfamily exon−intron and kinase domain diagram (all)

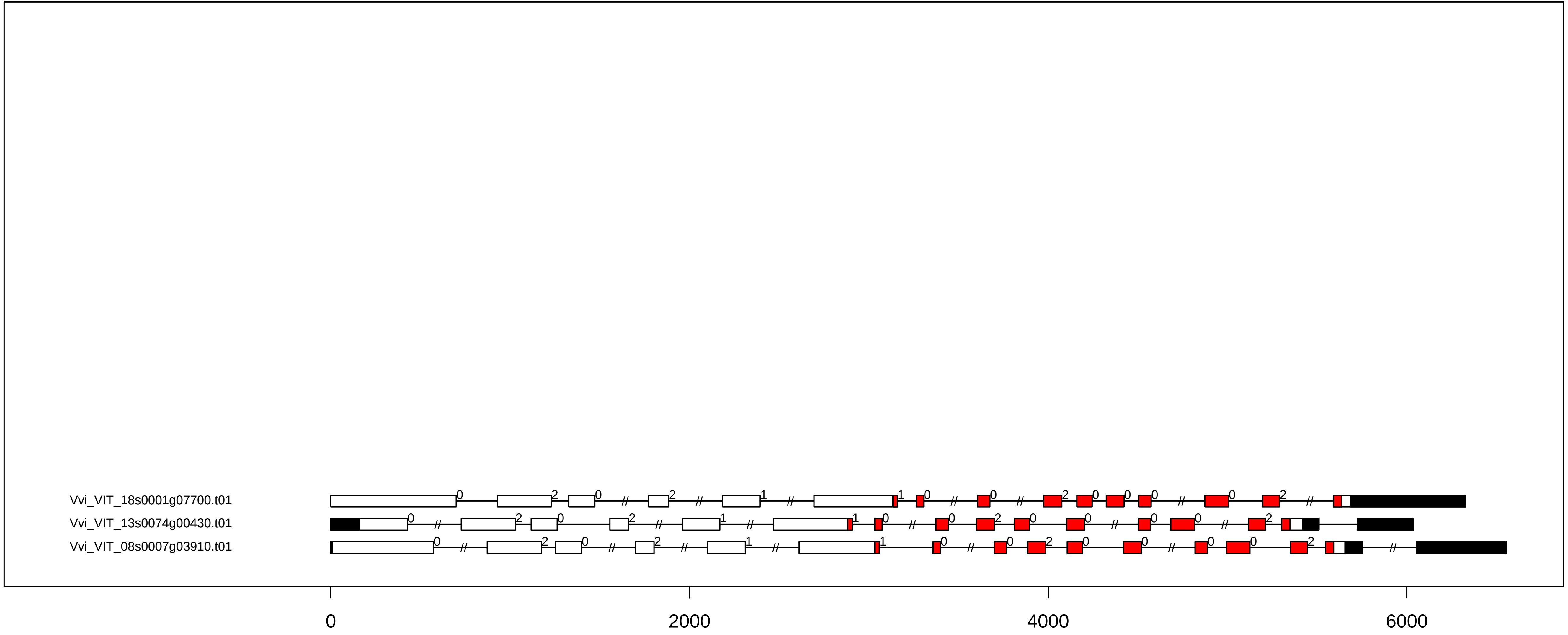

V.vi TKL_CTR1−DRK−2 III subfamily exon−intron and kinase domain diagram (all)

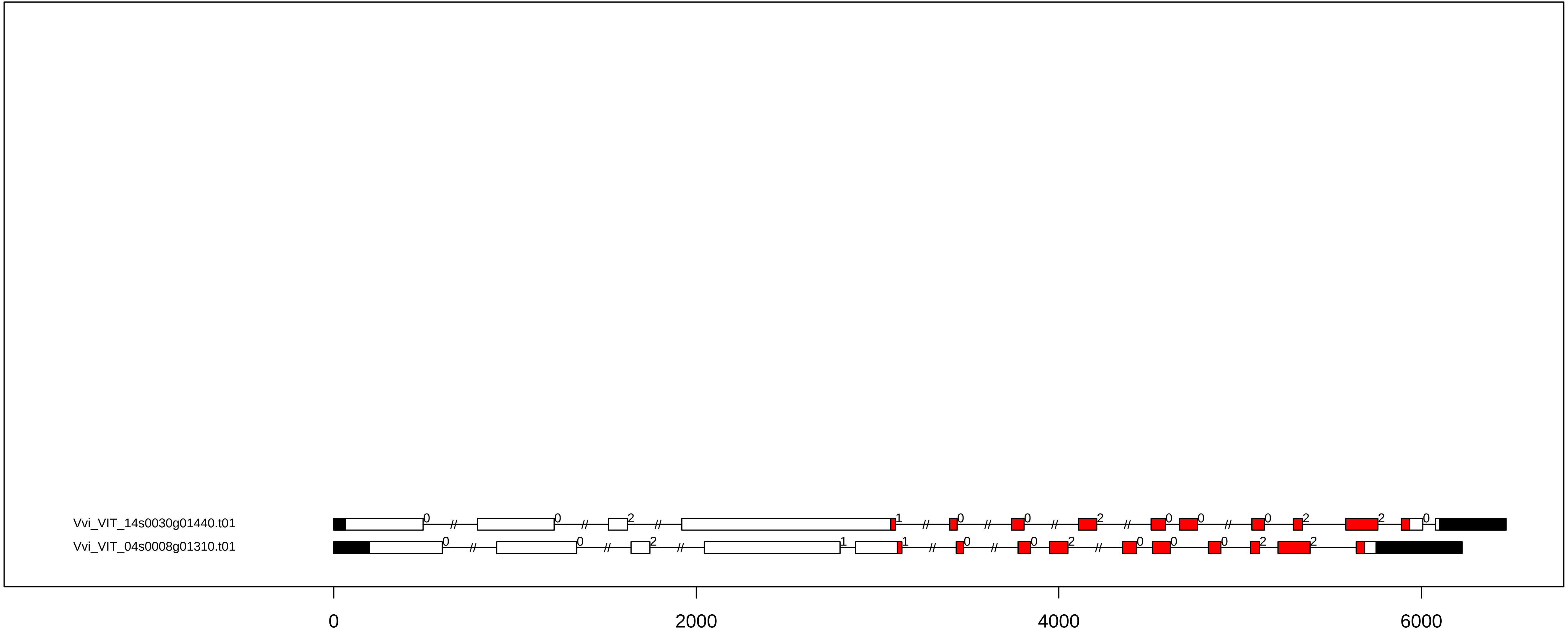

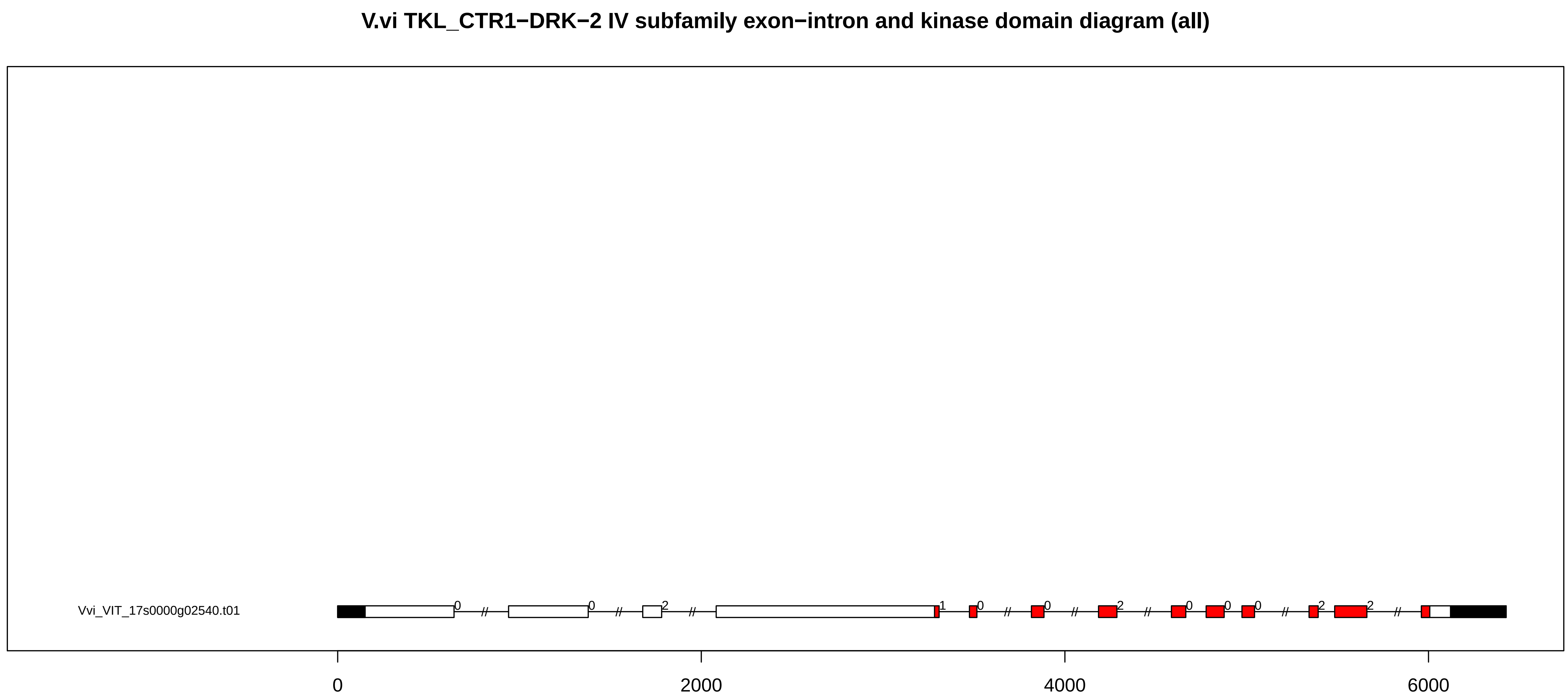
V.vi TKL_CTR1−DRK−2 IV subfamily exon−intron and kinase domain diagram (all)
Vvi_VIT_17s0000g02540.t01
0
0
2
1
0
0
2
0
0
0
2
2
0
2000
4000
6000

V.vi TKL_CTR1−DRK−2 (excluding in phylogenetic analysis) exon−intron and kinase domain diagram (all)

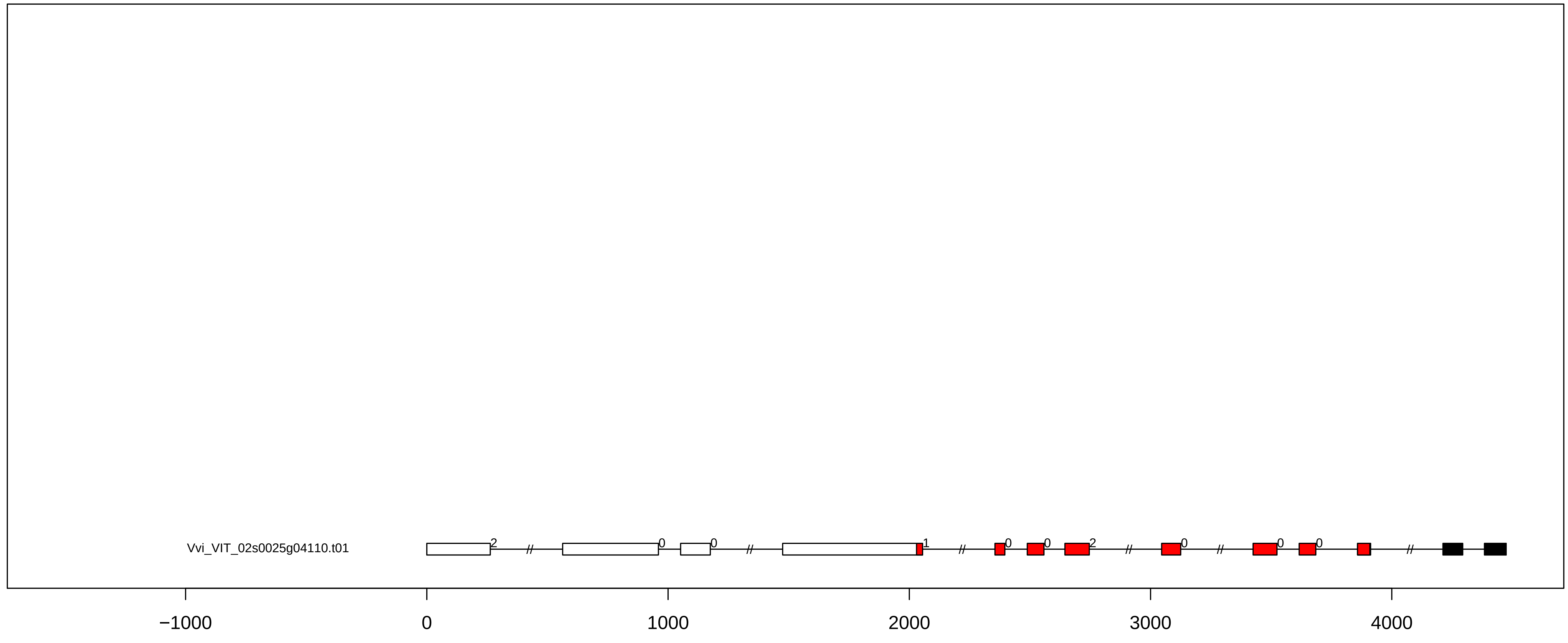

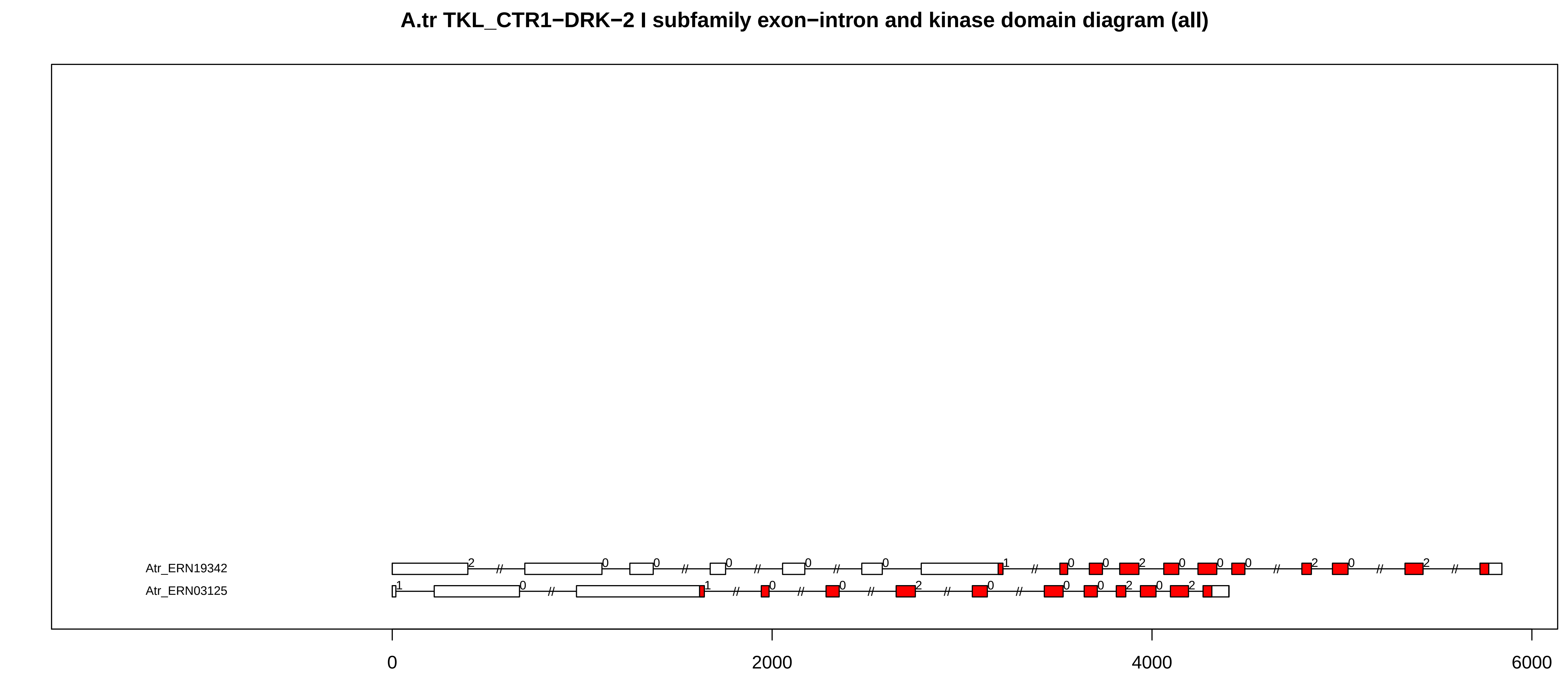
A.tr TKL_CTR1−DRK−2 I subfamily exon−intron and kinase domain diagram (all)
Atr_ERN19342
Atr_ERN03125
0
2000
4000
6000

A.tr TKL_CTR1−DRK−2 II subfamily exon−intron and kinase domain diagram (all)

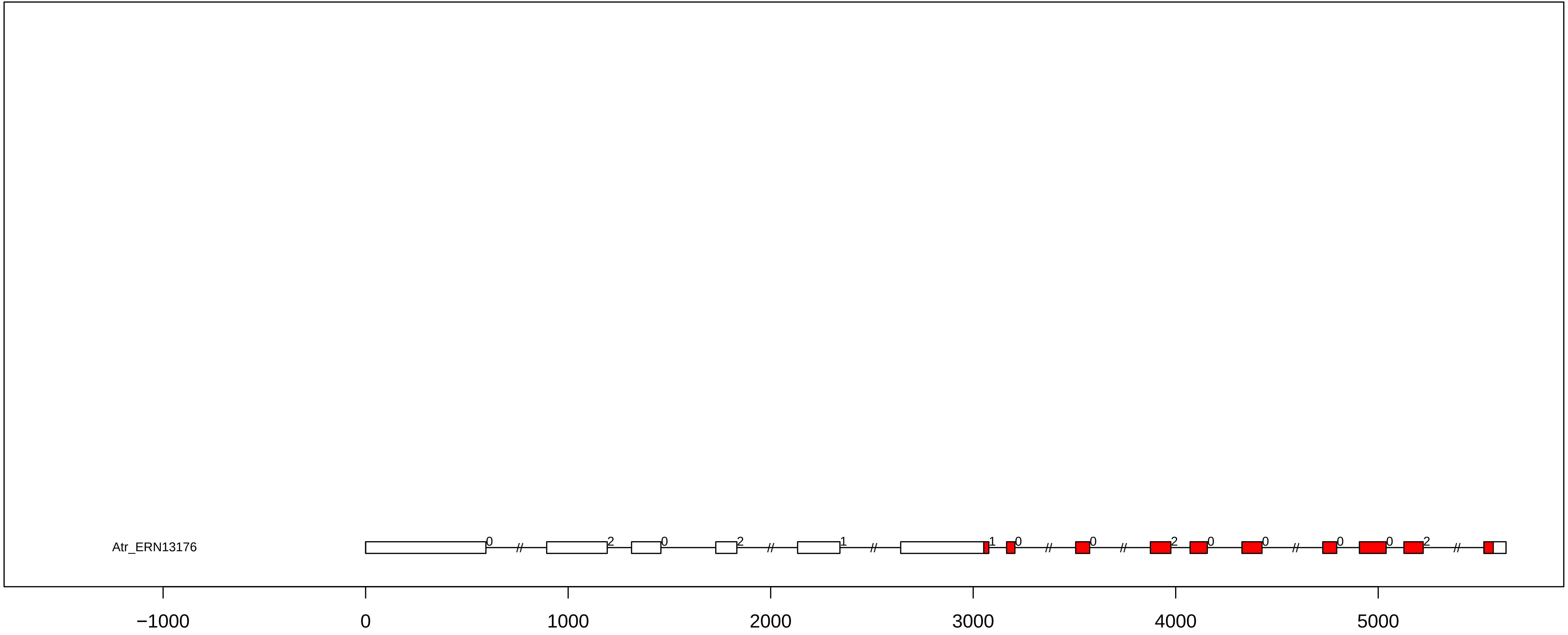

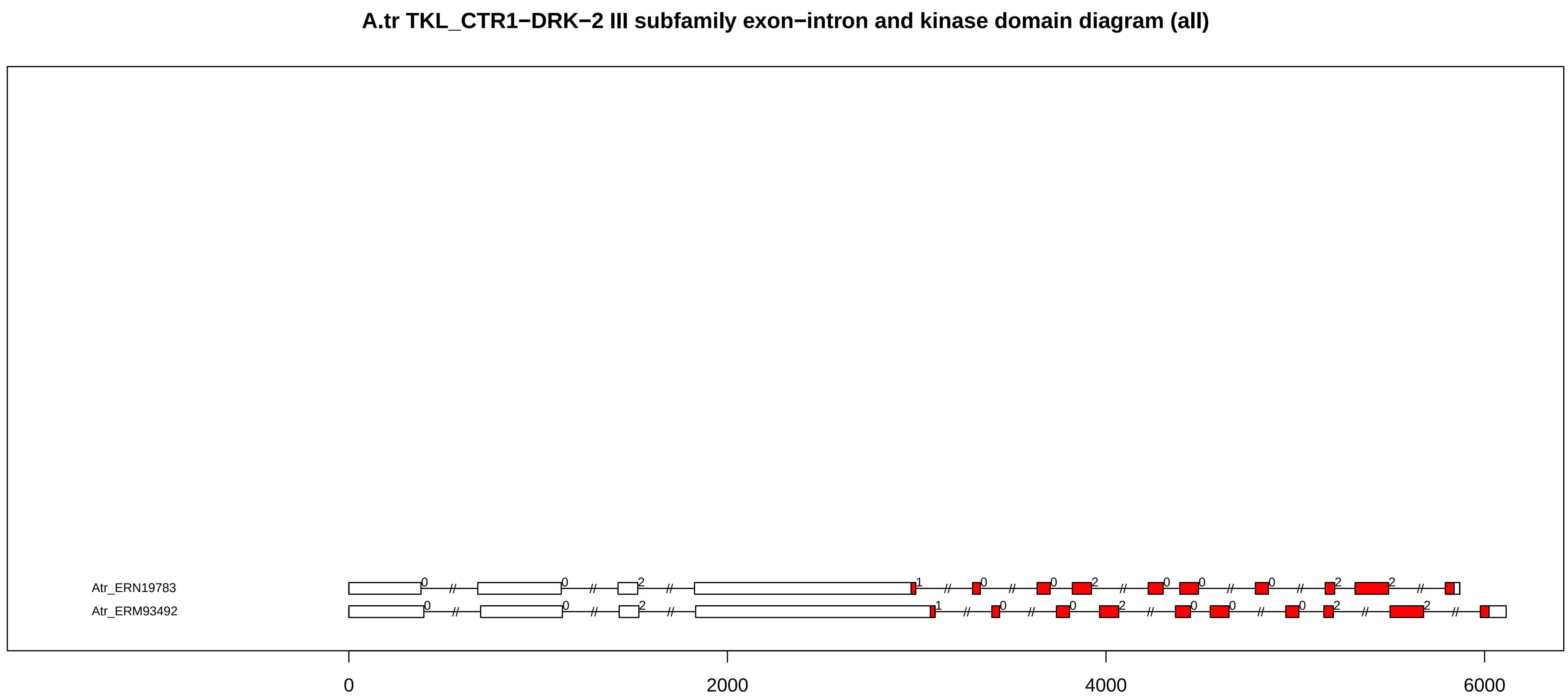
A.tr TKL_CTR1−DRK−2 III subfamily exon−intron and kinase domain diagram (all)
Atr_ERN19783
Atr_ERM93492
0
2000
4000
6000

A.tr TKL_CTR1−DRK−2 IV subfamily exon−intron and kinase domain diagram (all)

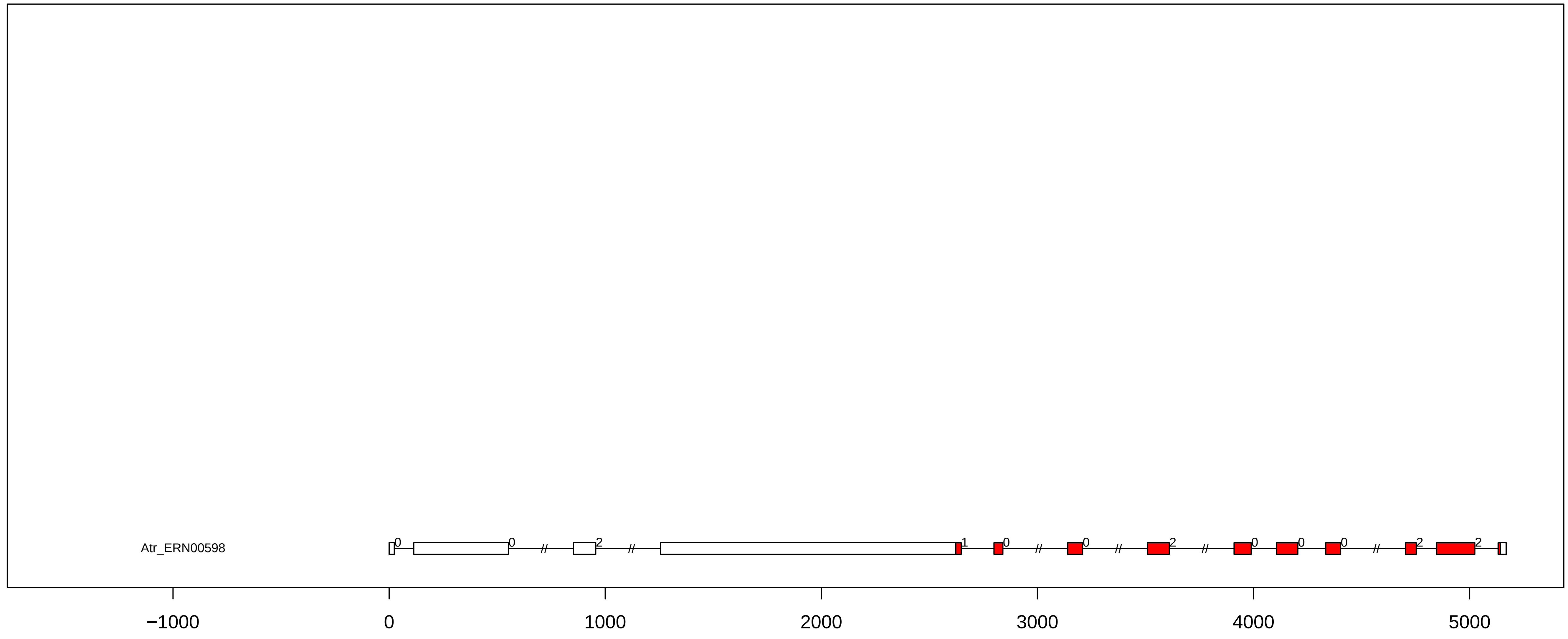

A.tr TKL_CTR1−DRK−2 (excluding in phylogenetic analysis) exon−intron and kinase domain diagram (all)

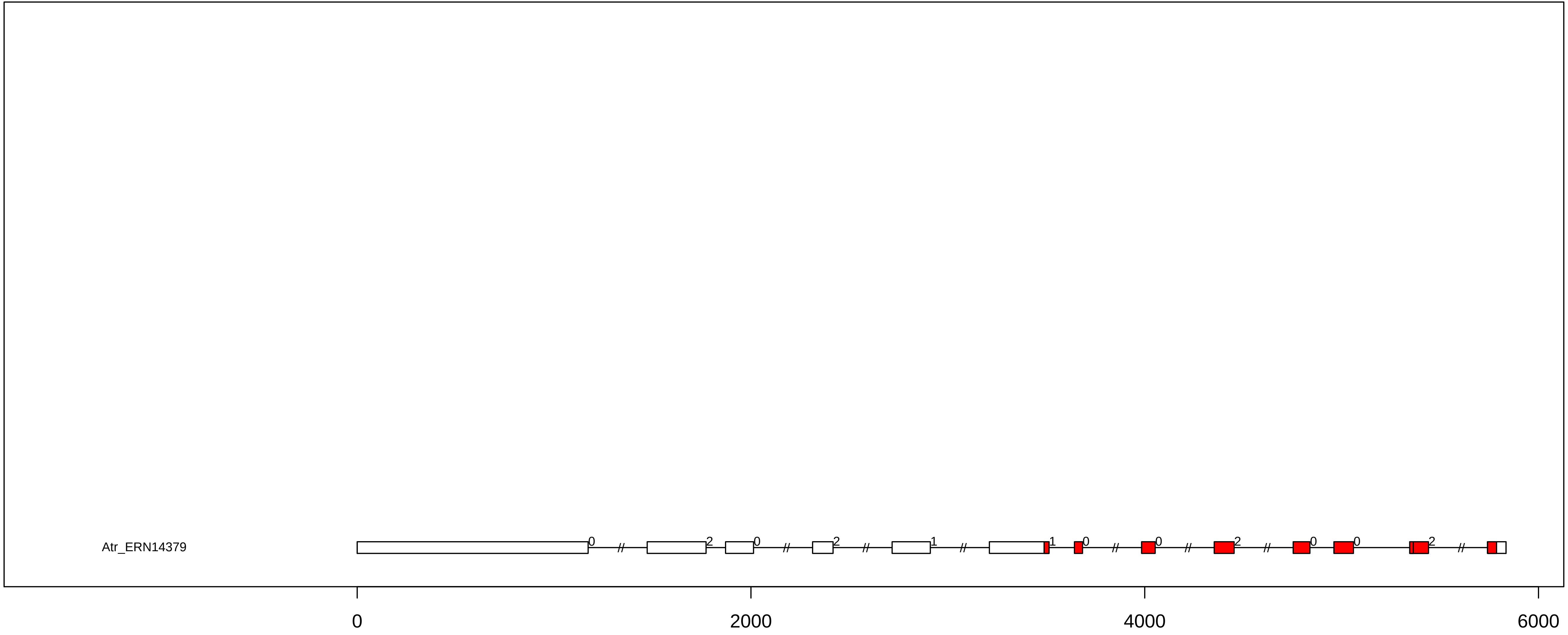

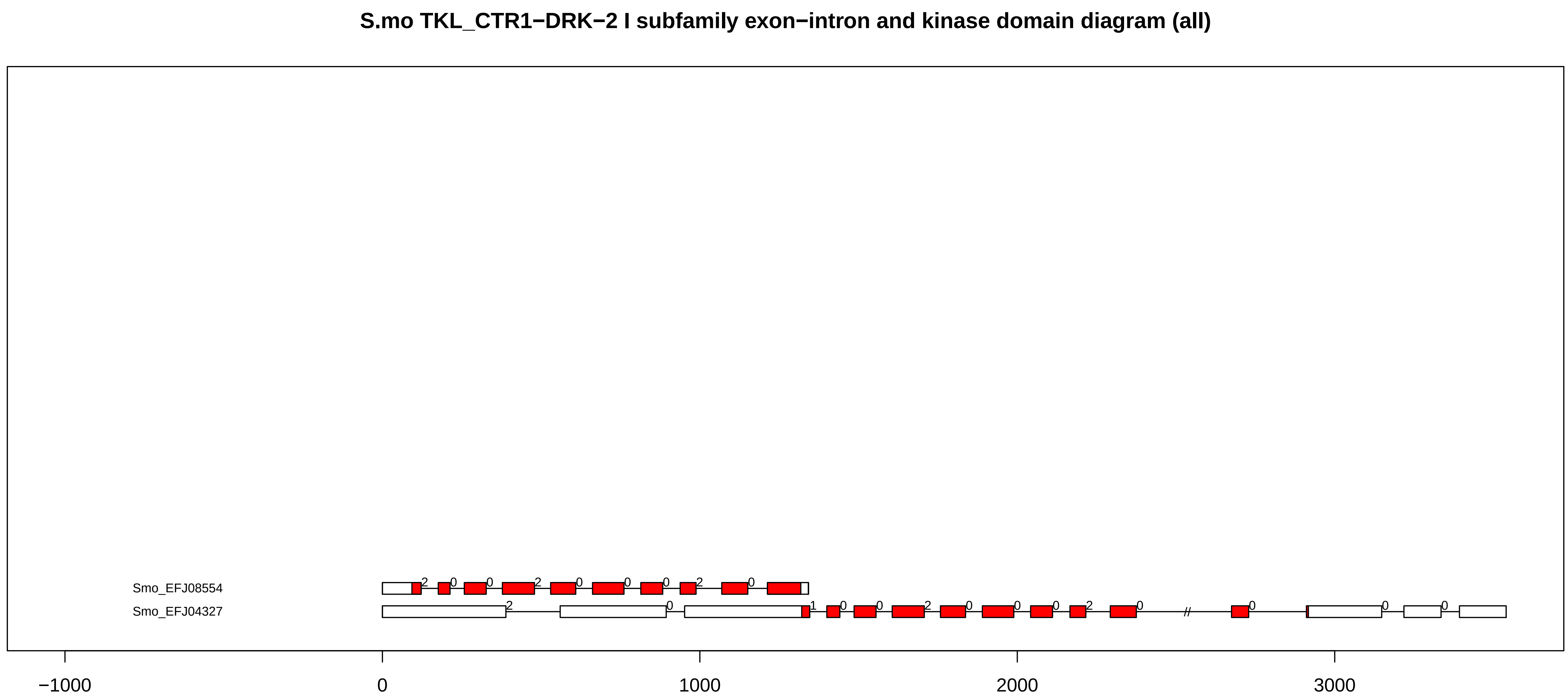
S.mo TKL_CTR1−DRK−2 I subfamily exon−intron and kinase domain diagram (all)
Smo_EFJ08554
Smo_EFJ04327
−1000
0
1000
2000
3000

S.mo TKL_CTR1−DRK−2 II subfamily exon−intron and kinase domain diagram (all)

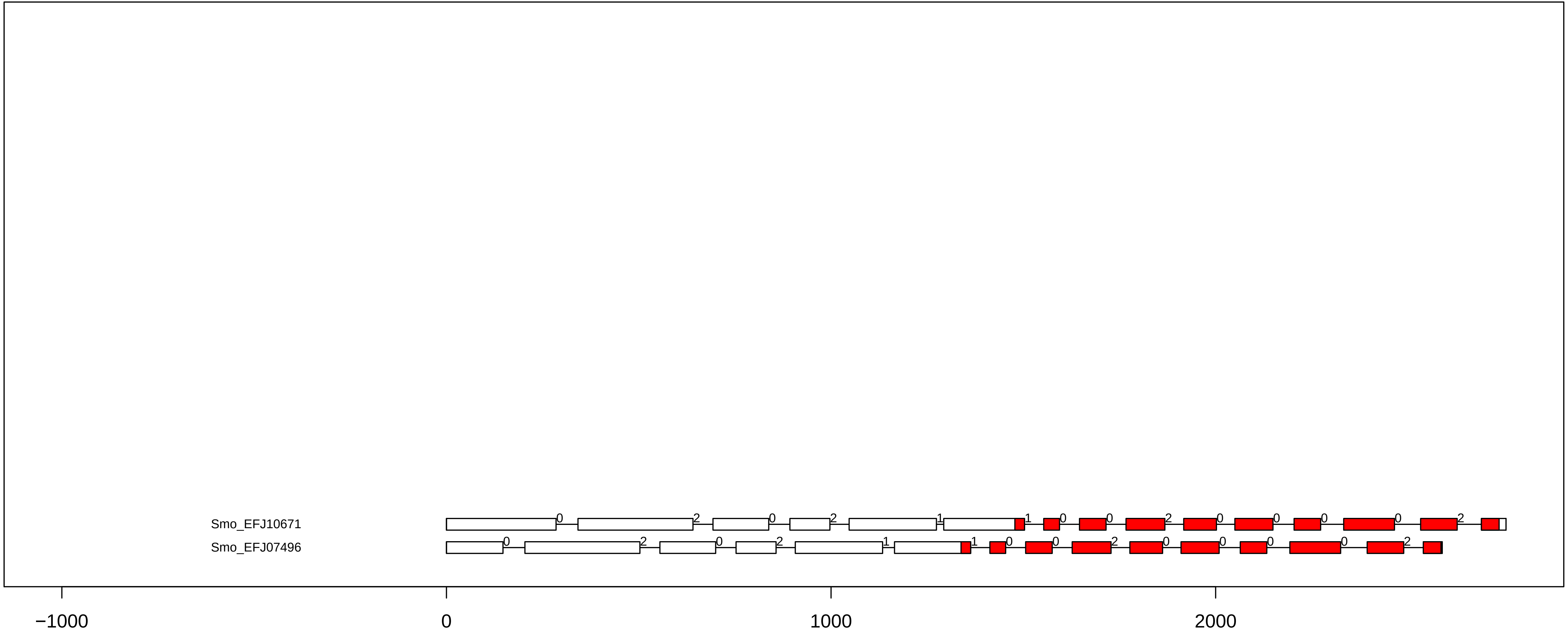

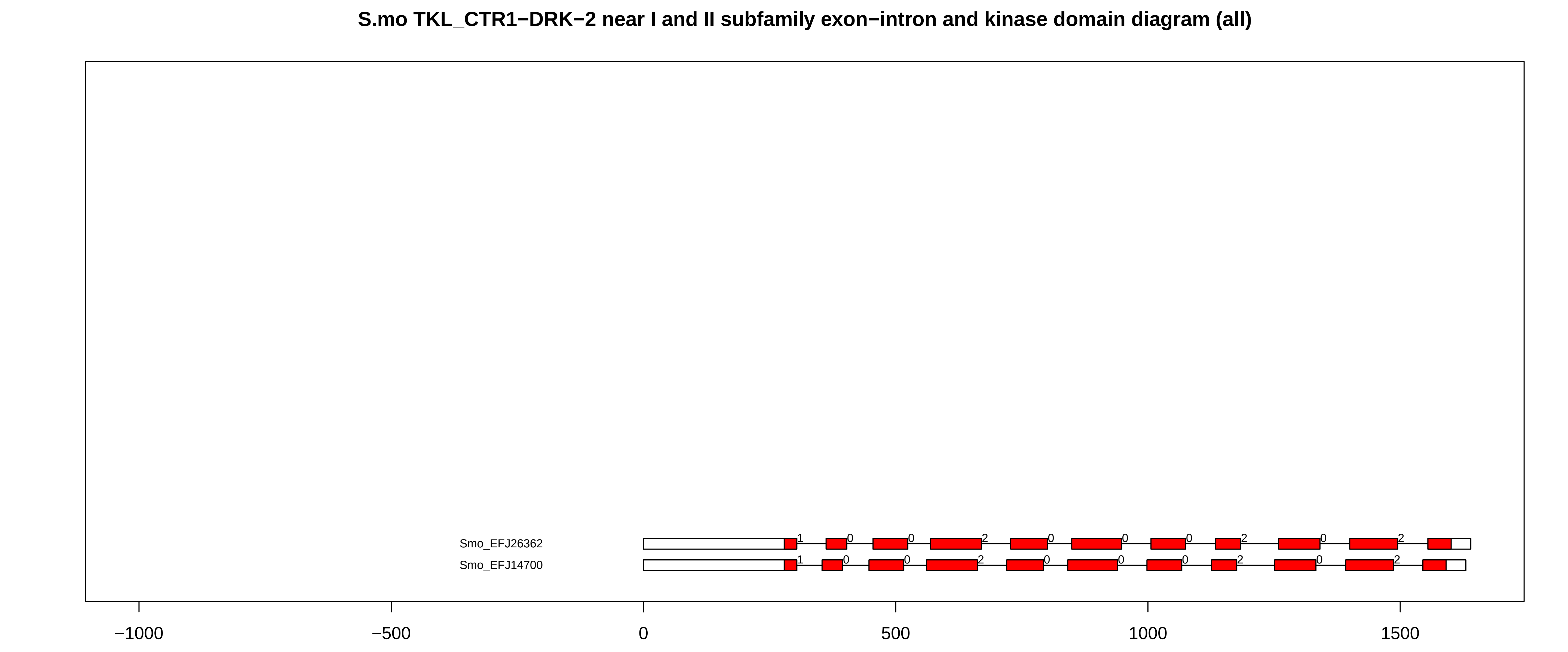

S.mo TKL_CTR1−DRK−2 near I and II subfamily exon−intron and kinase domain diagram (all)
Smo_EFJ26362
Smo_EFJ14700
1
0
0
2
0
0
0
2
0
2
−1000
−500
0
500
1000
1500

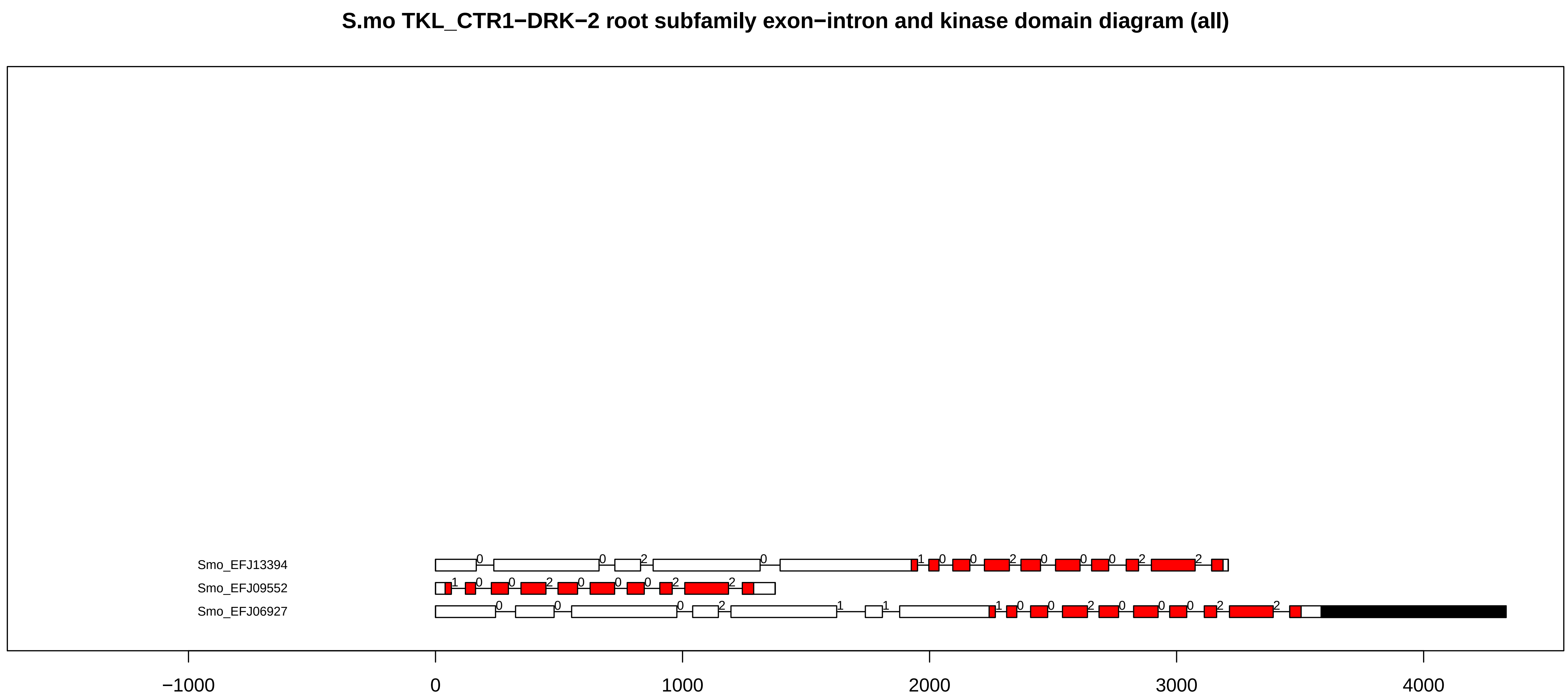
S.mo TKL_CTR1−DRK−2 root subfamily exon−intron and kinase domain diagram (all)
Smo_EFJ13394
Smo_EFJ09552
Smo_EFJ06927
−1000
0
1000
2000
3000
4000

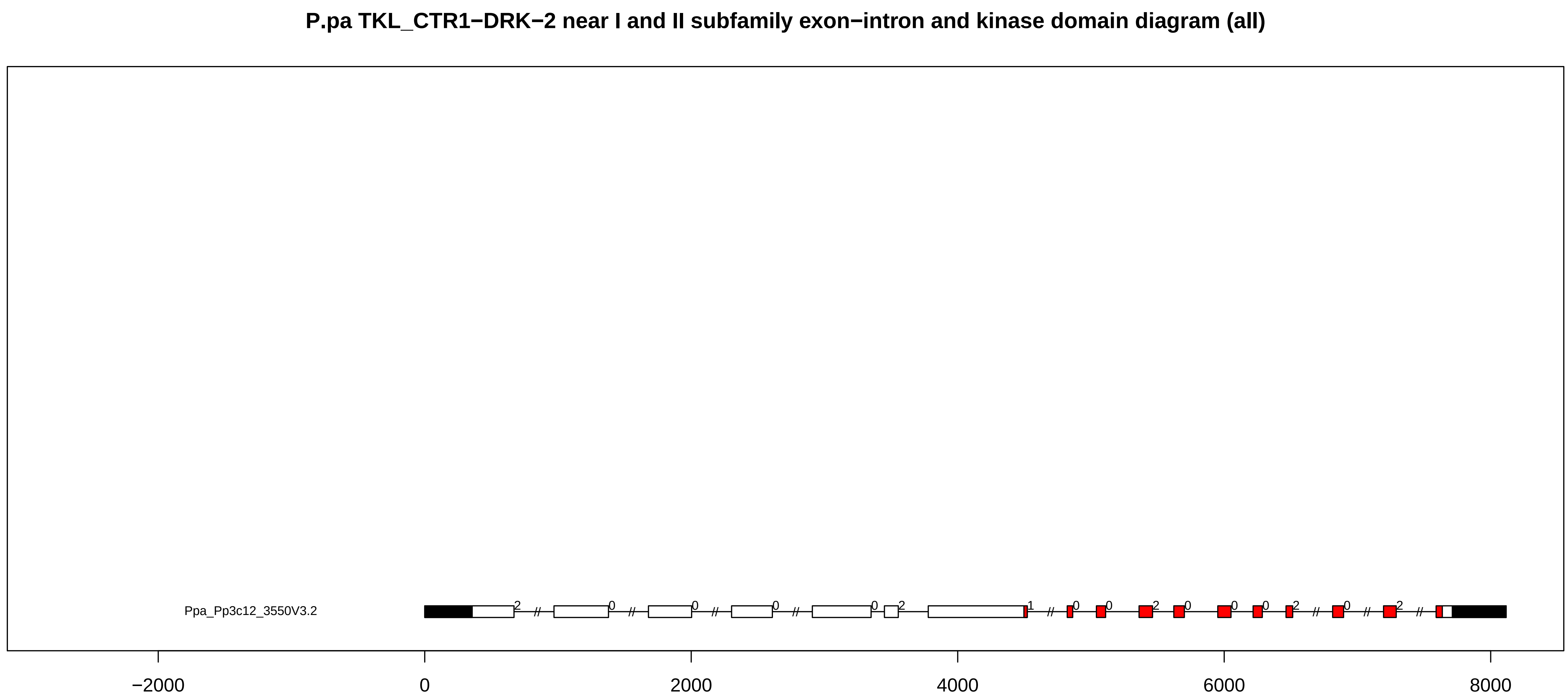

P.pa TKL_CTR1−DRK−2 near I and II subfamily exon−intron and kinase domain diagram (all)
Ppa_Pp3c12_3550V3.2
2
0
0
0
0
2
1
0
0
2
0
0
0
2
0
2
−2000
0
2000
4000
6000
8000

C.re TKL_CTR1−DRK−2 root subfamily exon−intron and kinase domain diagram (all)

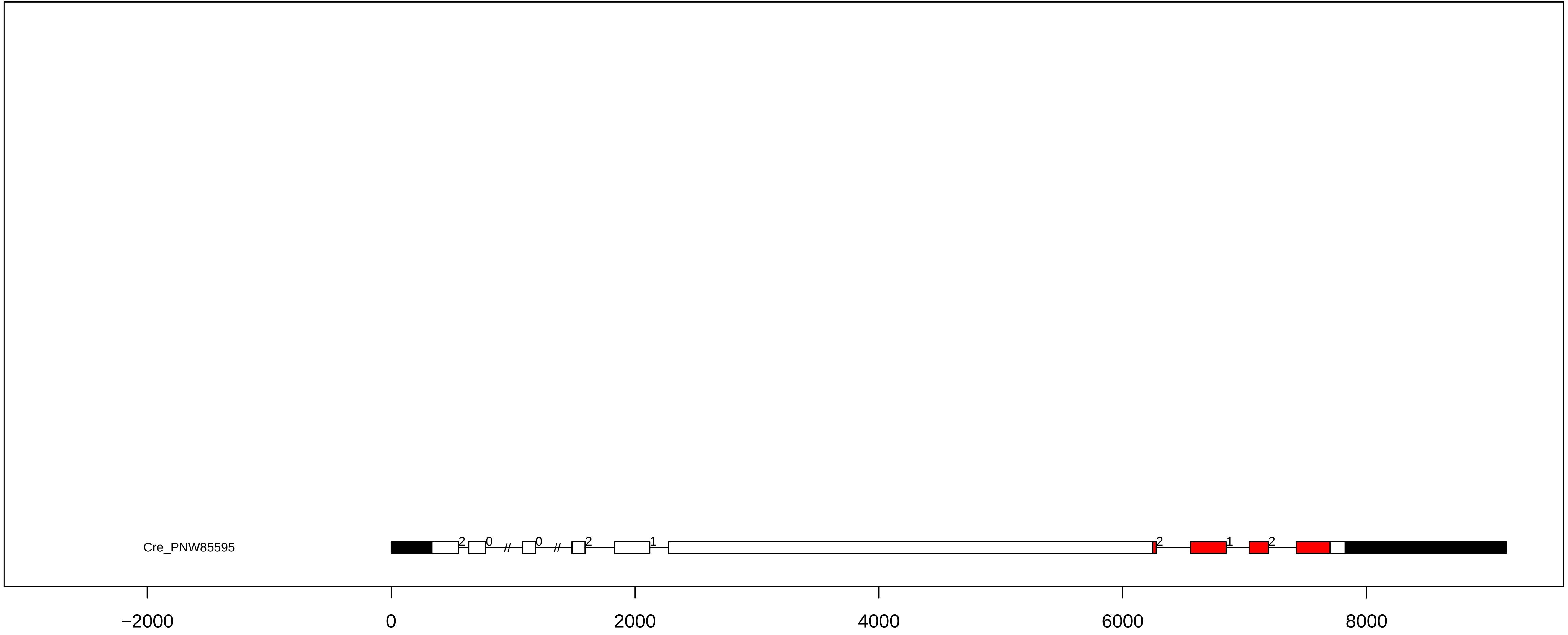

Supplement: Supplementary file 10 — Supplementary Material 10 [file 12864_2024_10383_MOESM10_ESM.pdf]
