# Supplementary material for: Genome-wide evolutionary analysis of TKL_CTR1-DRK-2 gene family and functional characterization reveals that TaCTR1 positively regulates flowering time in wheat
Source: BMC Genomics. 2024 May 14;25:474. doi: 10.1186/s12864-024-10383-2 (PMC11092142; doi:10.1186/s12864-024-10383-2)

TKL_CTR1−DRK−2 I−IV subfamilies exon−intron and kinase domain diagram (part 1)

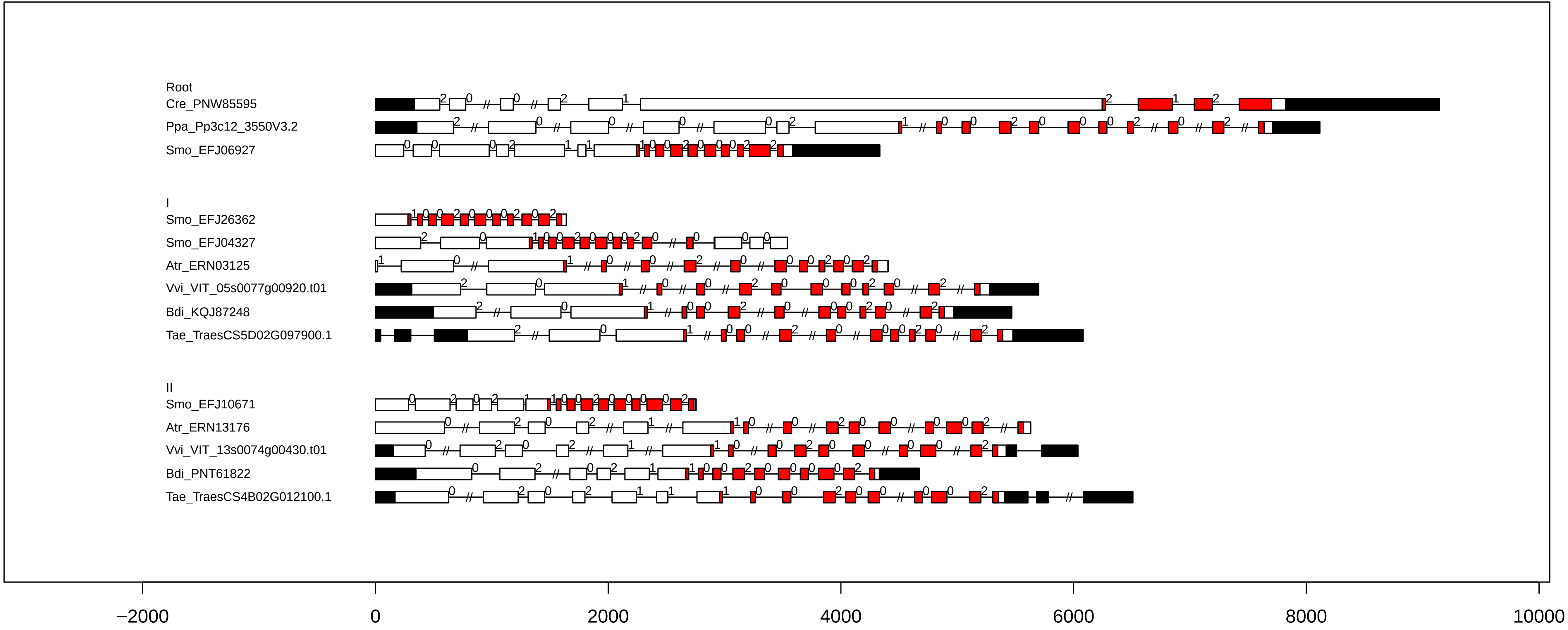

TKL_CTR1−DRK−2 I−IV subfamilies exon−intron and kinase domain diagram (part 2)

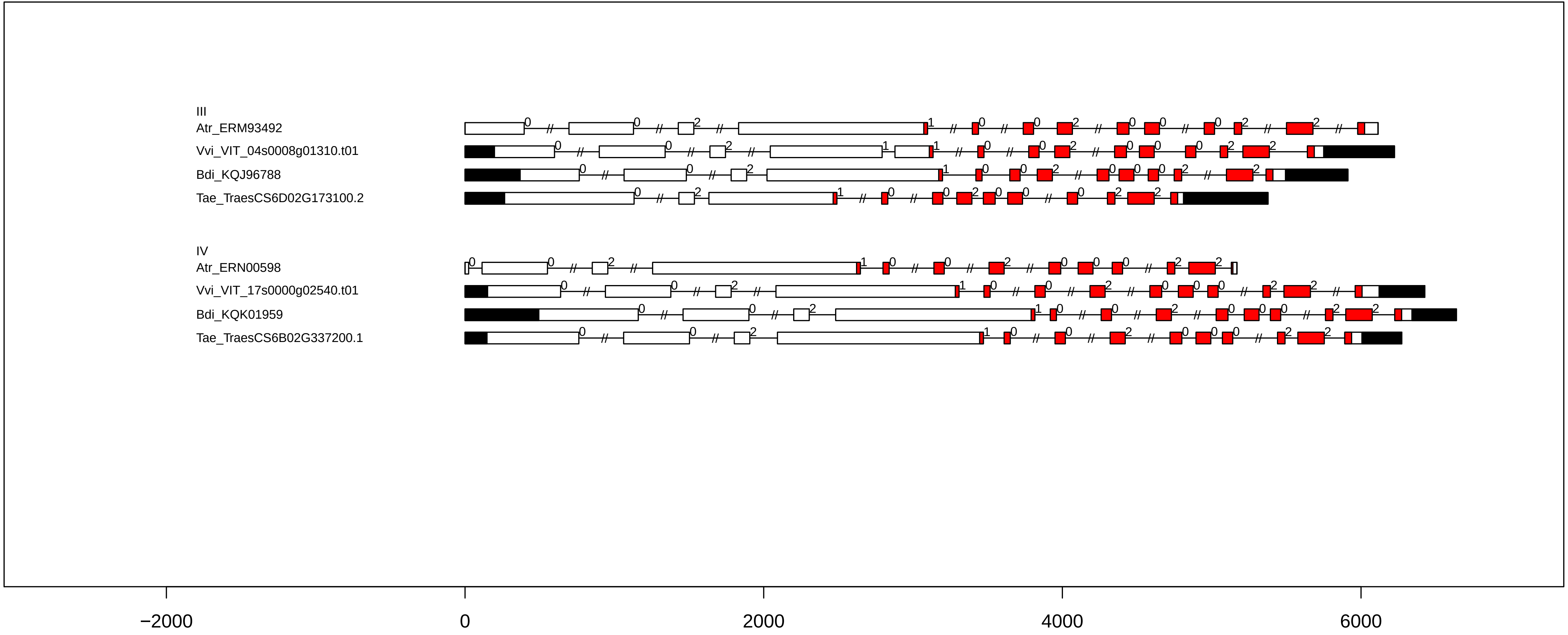

Supplement: Supplementary file 11 — Supplementary Material 11 [file 12864_2024_10383_MOESM11_ESM.pdf]
