# Supplementary material for: Genome-wide evolutionary analysis of TKL_CTR1-DRK-2 gene family and functional characterization reveals that TaCTR1 positively regulates flowering time in wheat
Source: BMC Genomics. 2024 May 14;25:474. doi: 10.1186/s12864-024-10383-2 (PMC11092142; doi:10.1186/s12864-024-10383-2)
Supplement: Supplementary file 12 — Supplementary Material 12 [file 12864_2024_10383_MOESM12_ESM.pdf]

		I	II	III	IV	excluding members	total number
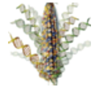	*Zea mays*	3	3	3	2	0	11
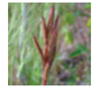	*Brachypodium distachyon*	3	3	3	2	0	11
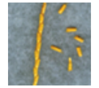	*Aegilops tauschii* （D genome）	5	2	3	2	0	12
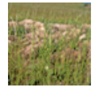	*Triticum urartu* （A genome）	4	2	1	1	1	9
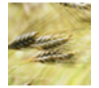	*Triticum dicoccoides* (AB subgenome）	11	4	6	4	0	25
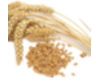	*Triticum turgidum* (AB subgenome）	12	4	6	4	0	26
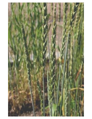	*Triticum spelta* （ABD subgenome）	11	6	7	6	3	33
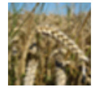	*Triticum aestivum* （ABD subgenome）	18	6	9	6	1	40
