# Supplementary material for: Genome-wide evolutionary analysis of TKL_CTR1-DRK-2 gene family and functional characterization reveals that TaCTR1 positively regulates flowering time in wheat
Source: BMC Genomics. 2024 May 14;25:474. doi: 10.1186/s12864-024-10383-2 (PMC11092142; doi:10.1186/s12864-024-10383-2)
Supplement: Supplementary file 13 — Supplementary Material 13 [file 12864_2024_10383_MOESM13_ESM.pdf]

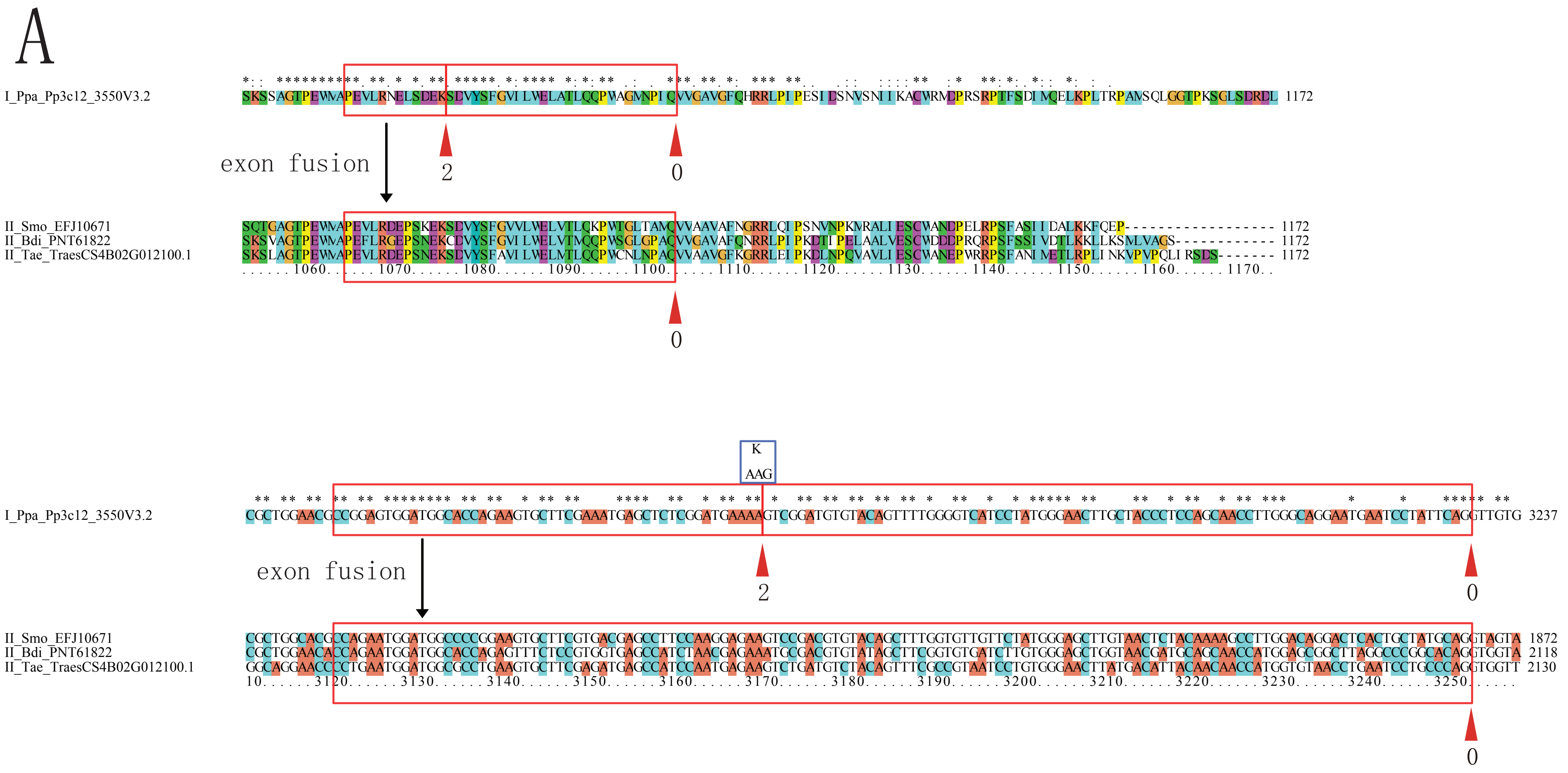
A
I_Ppa_Pp3c12_3550V3.2
exon fusion
2
0
II_Smo_EFJ10671
II_Bdi_PNT61822
II_Tae_TraesCS4B02G012100.1
K
AAG

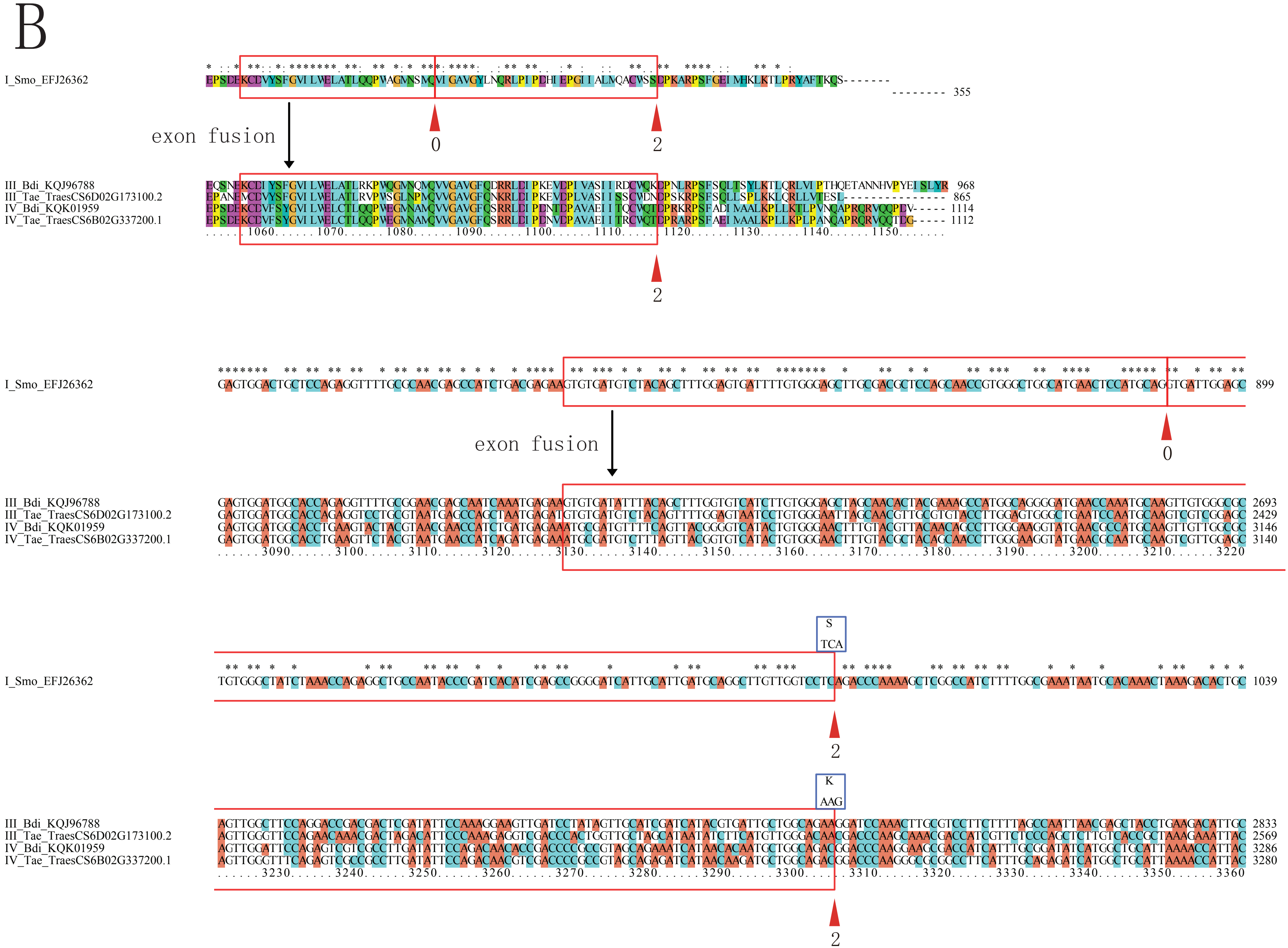

B
I_Smo_EFJ26362
exon fusion
III_Bdi_KQJ96788
III_Tae_TraesCS6D02G173100.2
IV_Bdi_KQK01959
IV_Tae_TraesCS6B02G337200.1
S
TCA
K
AAG
