# Supplementary material for: Genome-wide evolutionary analysis of TKL_CTR1-DRK-2 gene family and functional characterization reveals that TaCTR1 positively regulates flowering time in wheat
Source: BMC Genomics. 2024 May 14;25:474. doi: 10.1186/s12864-024-10383-2 (PMC11092142; doi:10.1186/s12864-024-10383-2)
Supplement: Supplementary file 14 — Supplementary Material 14 [file 12864_2024_10383_MOESM14_ESM.pdf]

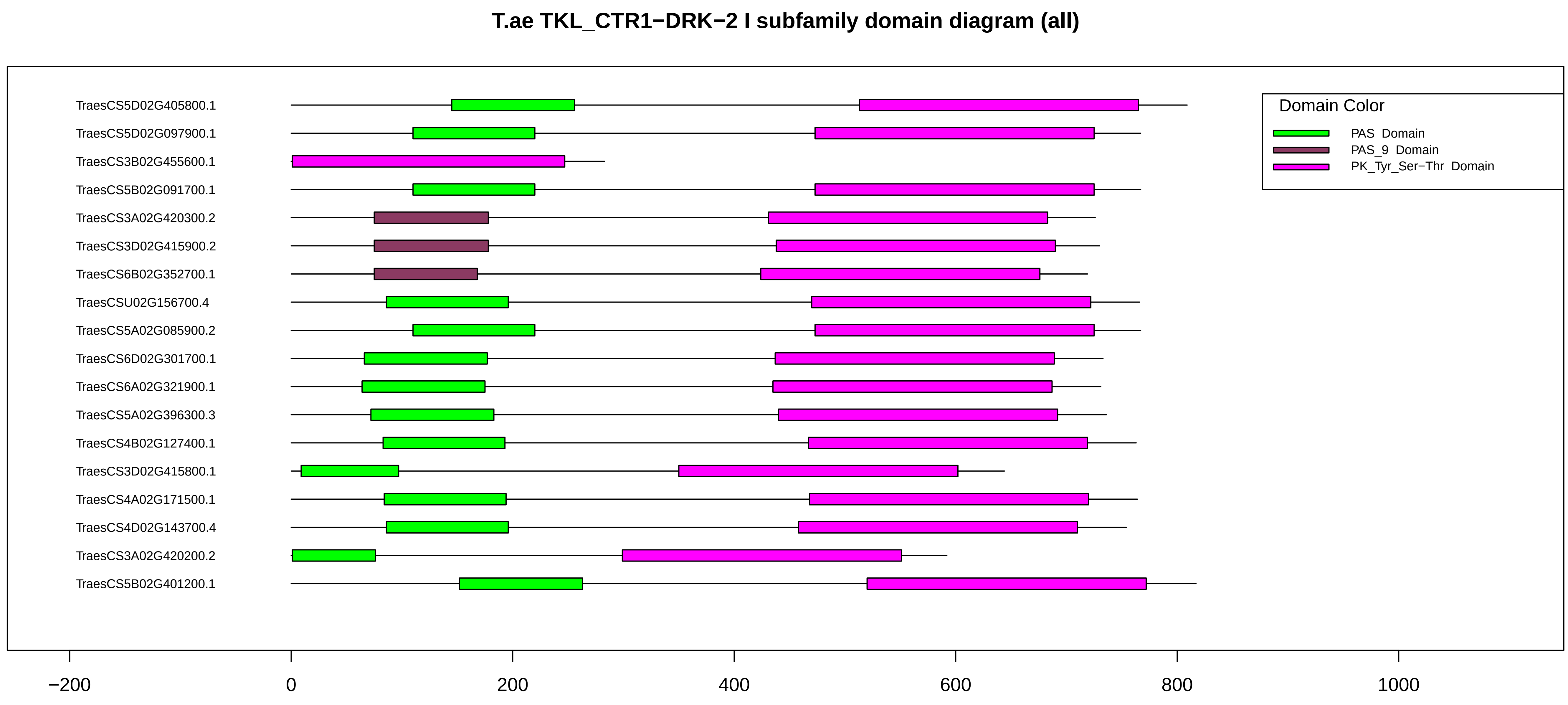

T.ae TKL_CTR1−DRK−2 I subfamily domain diagram (all)
TraesCS5D02G405800.1
TraesCS5D02G097900.1
TraesCS3B02G455600.1
TraesCS5B02G091700.1
TraesCS3A02G420300.2
TraesCS3D02G415900.2
TraesCS6B02G352700.1
TraesCSU02G156700.4
TraesCS5A02G085900.2
TraesCS6D02G301700.1
TraesCS6A02G321900.1
TraesCS5A02G396300.3
TraesCS4B02G127400.1
TraesCS3D02G415800.1
TraesCS4A02G171500.1
TraesCS4D02G143700.4
TraesCS3A02G420200.2
TraesCS5B02G401200.1
Domain Color
PAS Domain
PAS_9 Domain
PK_Tyr_Ser−Thr Domain
−200
0
200
400
600
800
1000

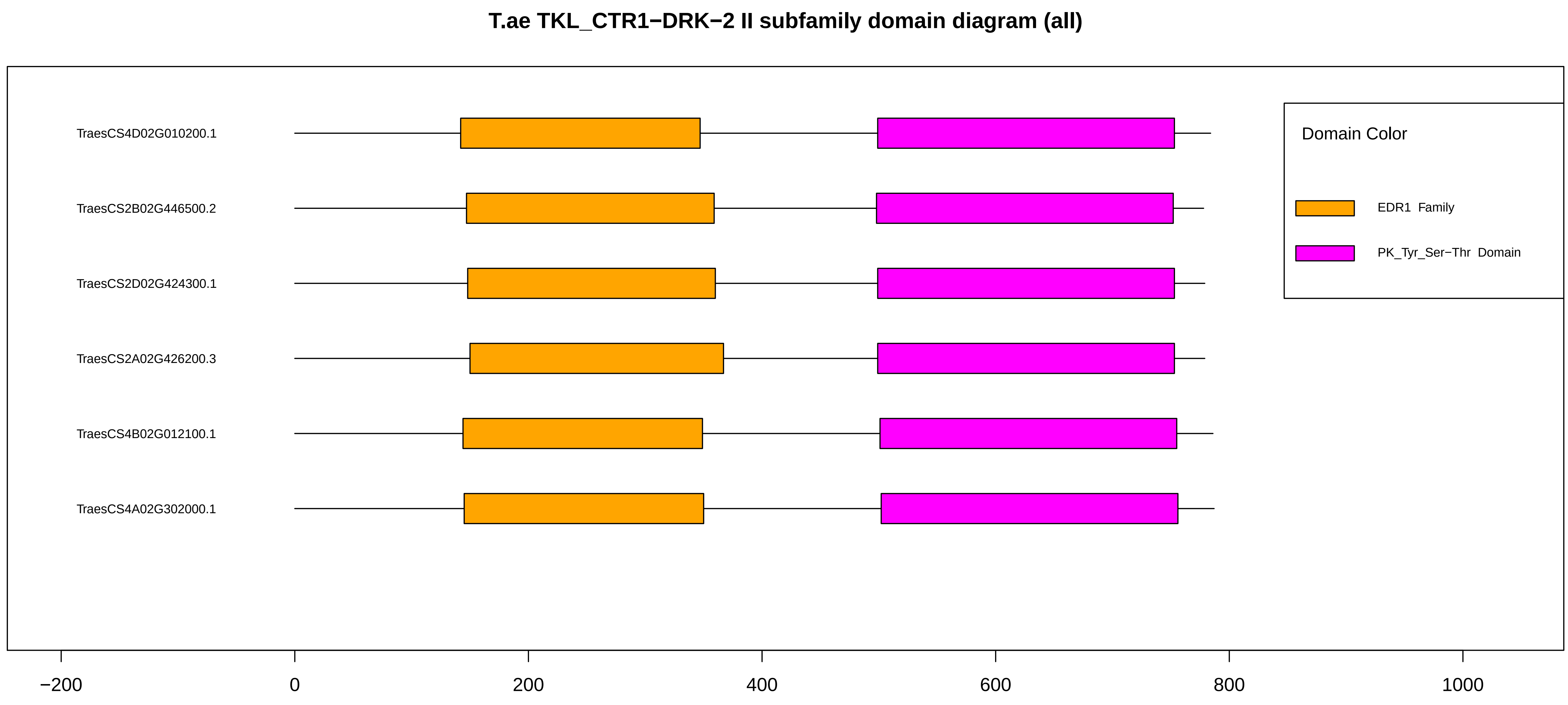

T.ae TKL_CTR1−DRK−2 II subfamily domain diagram (all)
TraesCS4D02G010200.1
TraesCS2B02G446500.2
TraesCS2D02G424300.1
TraesCS2A02G426200.3
TraesCS4B02G012100.1
TraesCS4A02G302000.1
Domain Color
EDR1 Family
PK_Tyr_Ser−Thr Domain
−200
0
200
400
600
800
1000

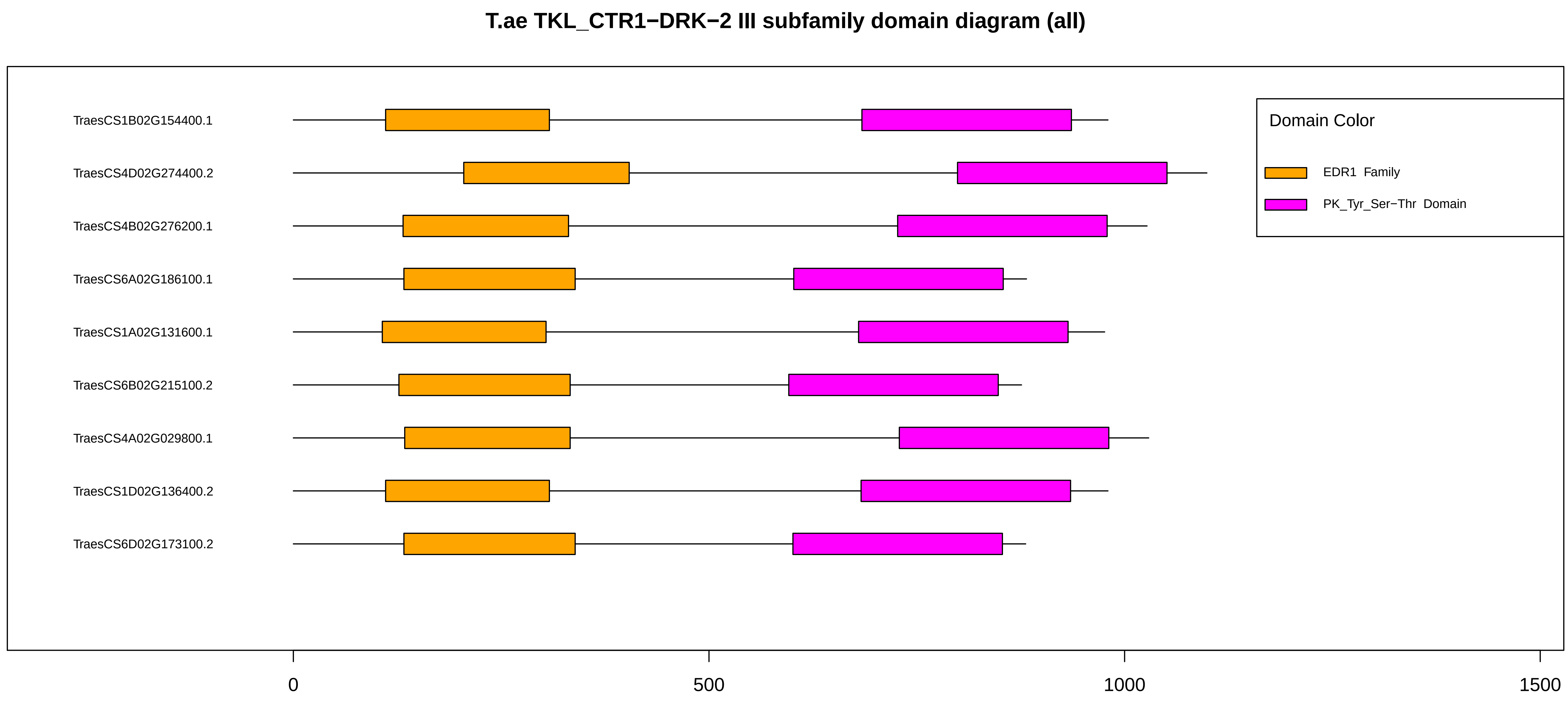
T.ae TKL_CTR1−DRK−2 III subfamily domain diagram (all)
TraesCS1B02G154400.1
TraesCS4D02G274400.2
TraesCS4B02G276200.1
TraesCS6A02G186100.1
TraesCS1A02G131600.1
TraesCS6B02G215100.2
TraesCS4A02G029800.1
TraesCS1D02G136400.2
TraesCS6D02G173100.2
Domain Color
EDR1 Family
PK_Tyr_Ser−Thr Domain
0
500
1000
1500

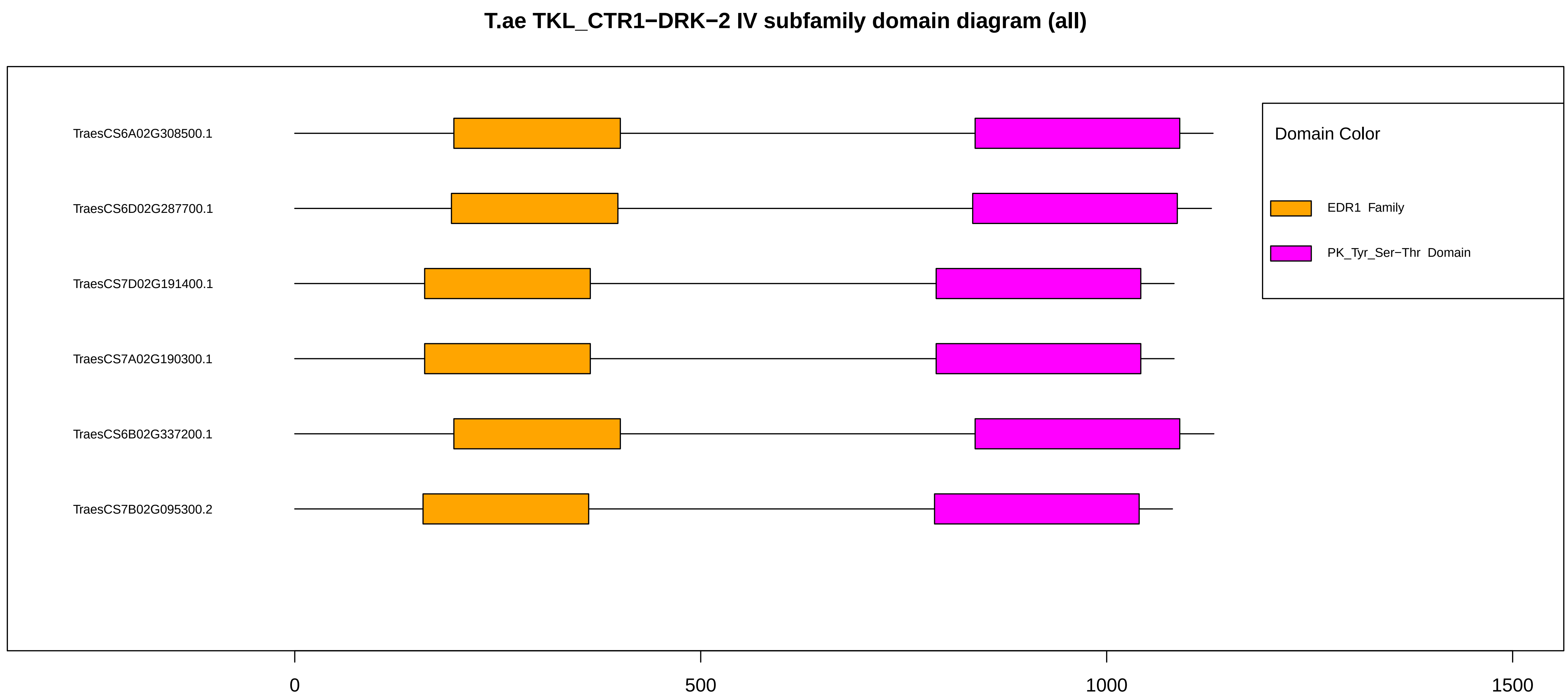

T.ae TKL_CTR1−DRK−2 IV subfamily domain diagram (all)
TraesCS6A02G308500.1
TraesCS6D02G287700.1
TraesCS7D02G191400.1
TraesCS7A02G190300.1
TraesCS6B02G337200.1
TraesCS7B02G095300.2
Domain Color
EDR1 Family
PK_Tyr_Ser−Thr Domain
0
500
1000
1500

T.ae TKL_CTR1−DRK−2 (excluding in phylogenetic analysis) domain diagram (all)

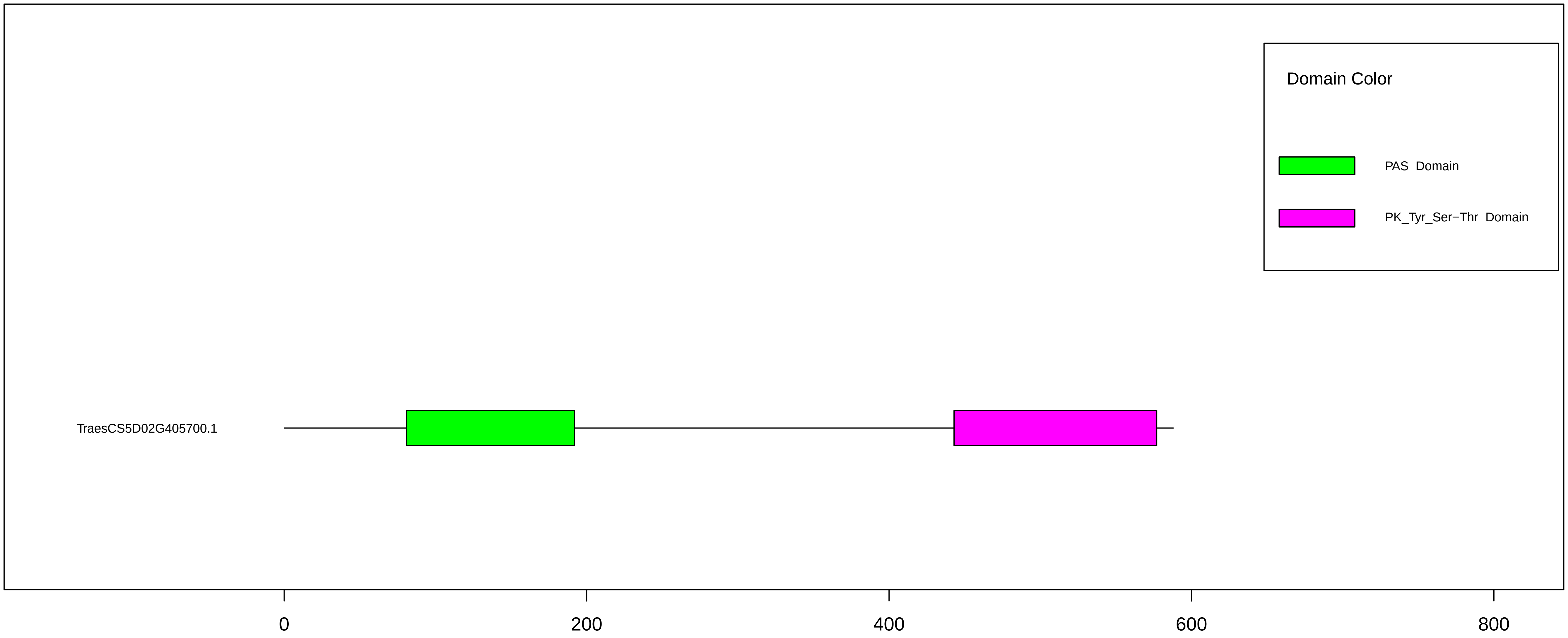

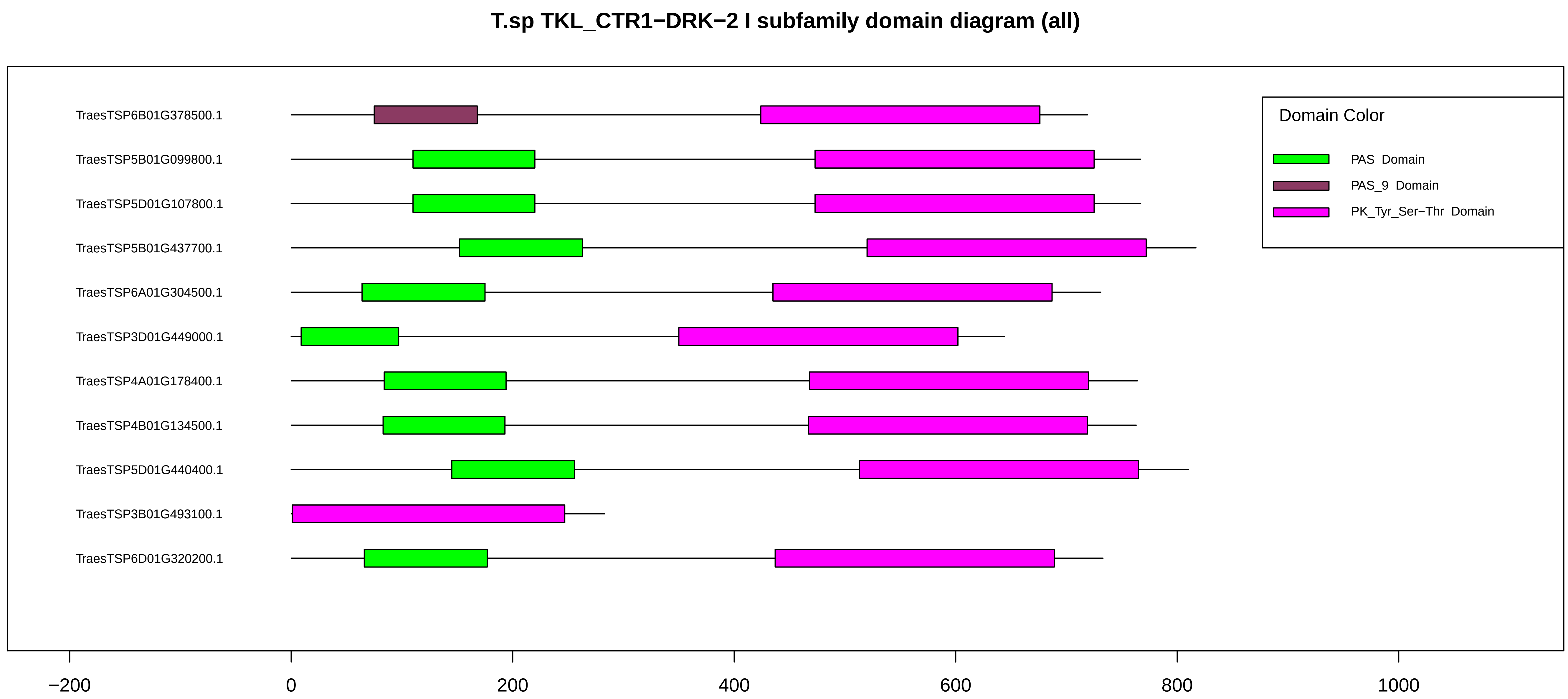
T.sp TKL_CTR1−DRK−2 I subfamily domain diagram (all)
TraesTSP6B01G378500.1
TraesTSP5B01G099800.1
TraesTSP5D01G107800.1
TraesTSP5B01G437700.1
TraesTSP6A01G304500.1
TraesTSP3D01G449000.1
TraesTSP4A01G178400.1
TraesTSP4B01G134500.1
TraesTSP5D01G440400.1
TraesTSP3B01G493100.1
TraesTSP6D01G320200.1
Domain Color
PAS Domain
PAS_9 Domain
PK_Tyr_Ser−Thr Domain
−200
0
200
400
600
800
1000

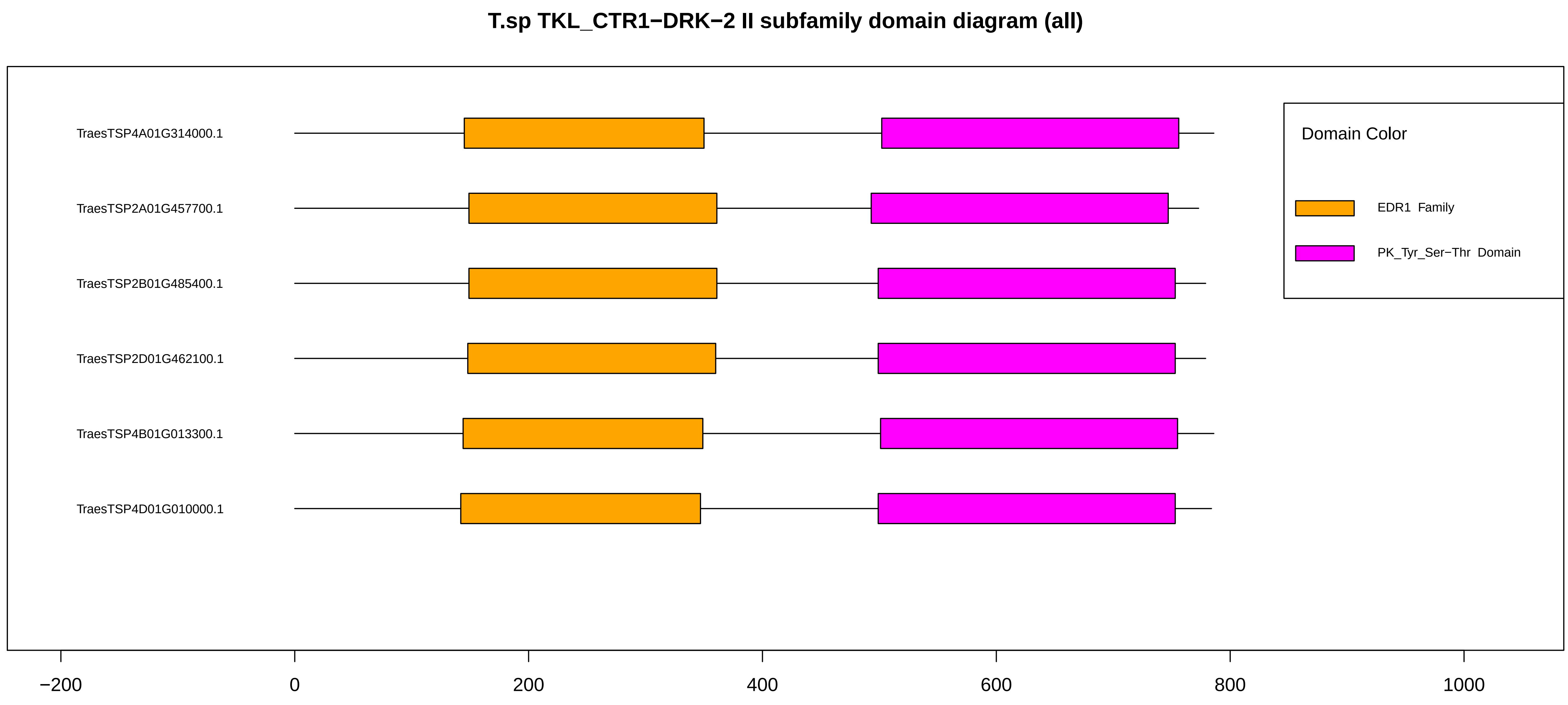
T.sp TKL_CTR1−DRK−2 II subfamily domain diagram (all)
TraesTSP4A01G314000.1
TraesTSP2A01G457700.1
TraesTSP2B01G485400.1
TraesTSP2D01G462100.1
TraesTSP4B01G013300.1
TraesTSP4D01G010000.1
Domain Color
EDR1 Family
PK_Tyr_Ser−Thr Domain
−200
0
200
400
600
800
1000

T.sp TKL_CTR1−DRK−2 III subfamily domain diagram (all)

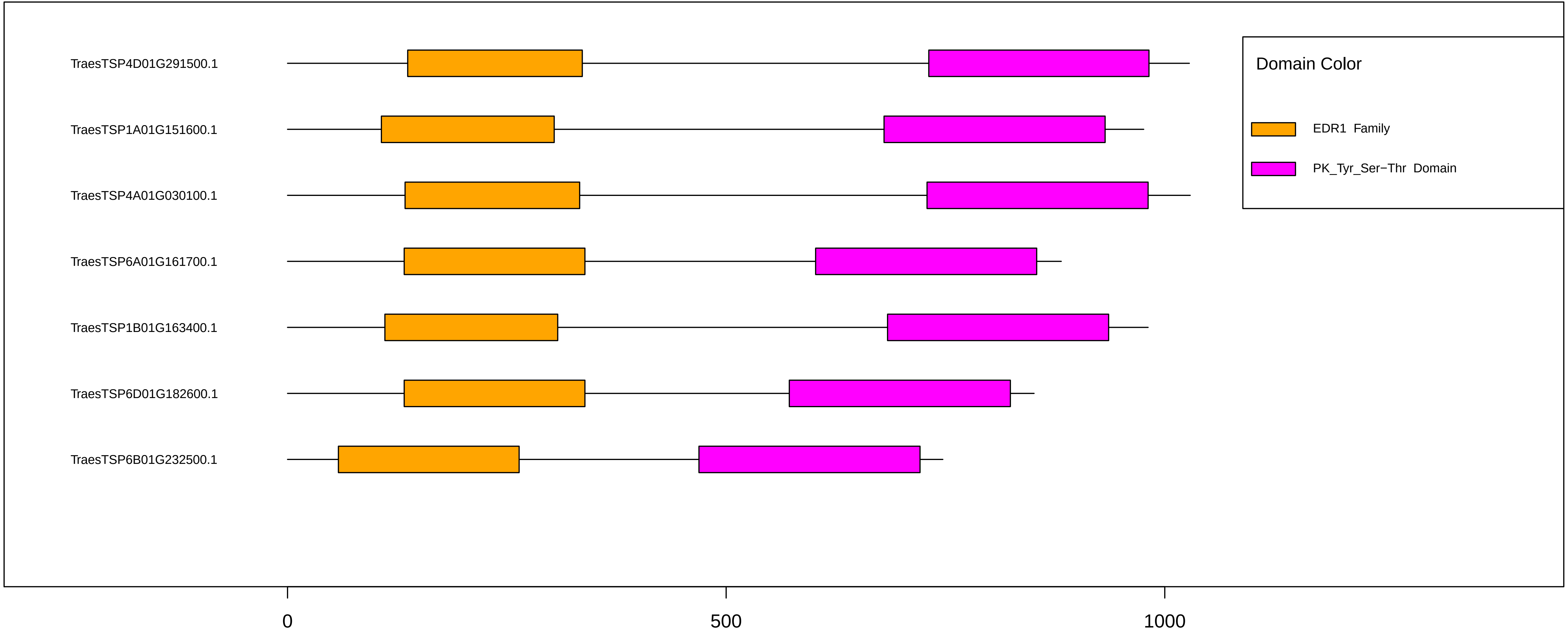

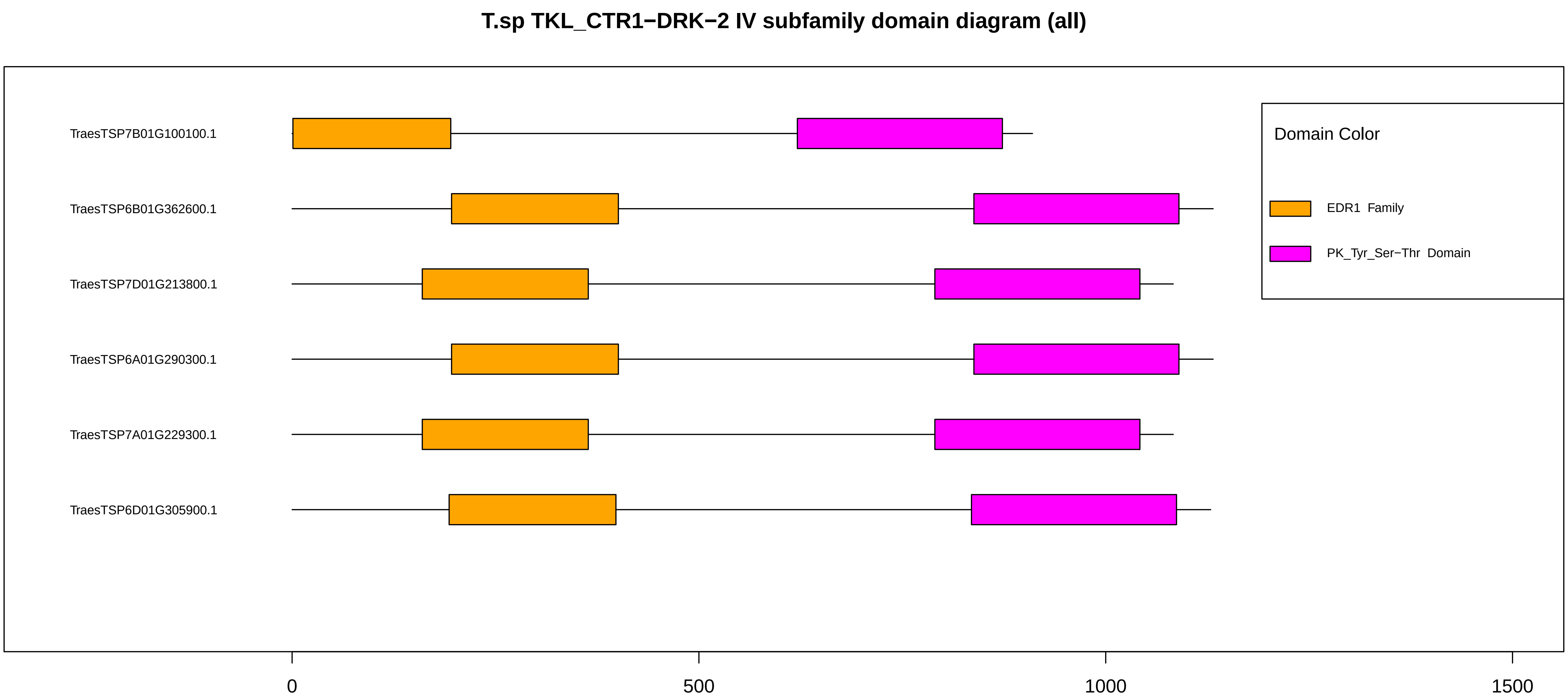

T.sp TKL_CTR1−DRK−2 IV subfamily domain diagram (all)
TraesTSP7B01G100100.1
TraesTSP6B01G362600.1
TraesTSP7D01G213800.1
TraesTSP6A01G290300.1
TraesTSP7A01G229300.1
TraesTSP6D01G305900.1
Domain Color
EDR1 Family
PK_Tyr_Ser−Thr Domain
0
500
1000
1500

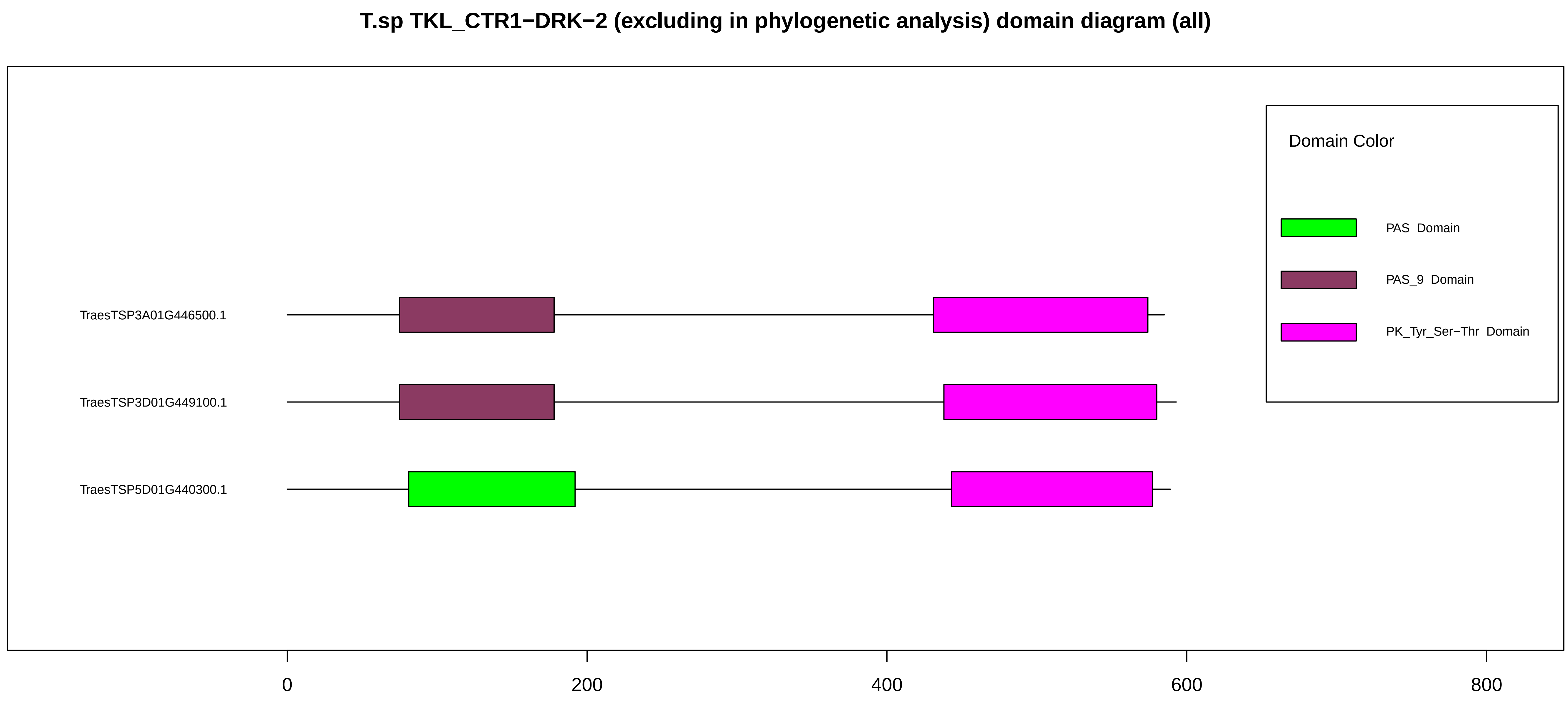
T.sp TKL_CTR1−DRK−2 (excluding in phylogenetic analysis) domain diagram (all)
Domain Color
PAS Domain
PAS_9 Domain
PK_Tyr_Ser−Thr Domain
TraesTSP3A01G446500.1
TraesTSP3D01G449100.1
TraesTSP5D01G440300.1
0
200
400
600
800

T.tu TKL_CTR1−DRK−2 I subfamily domain diagram (all)

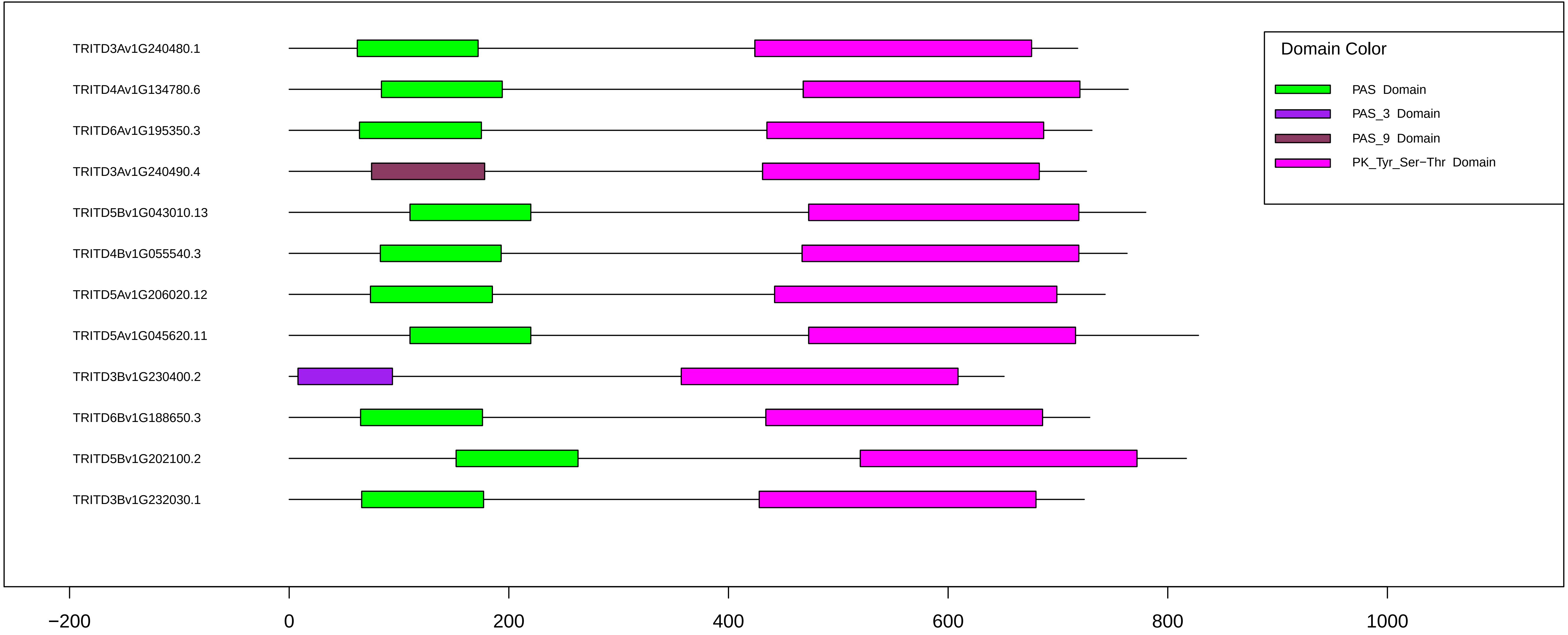

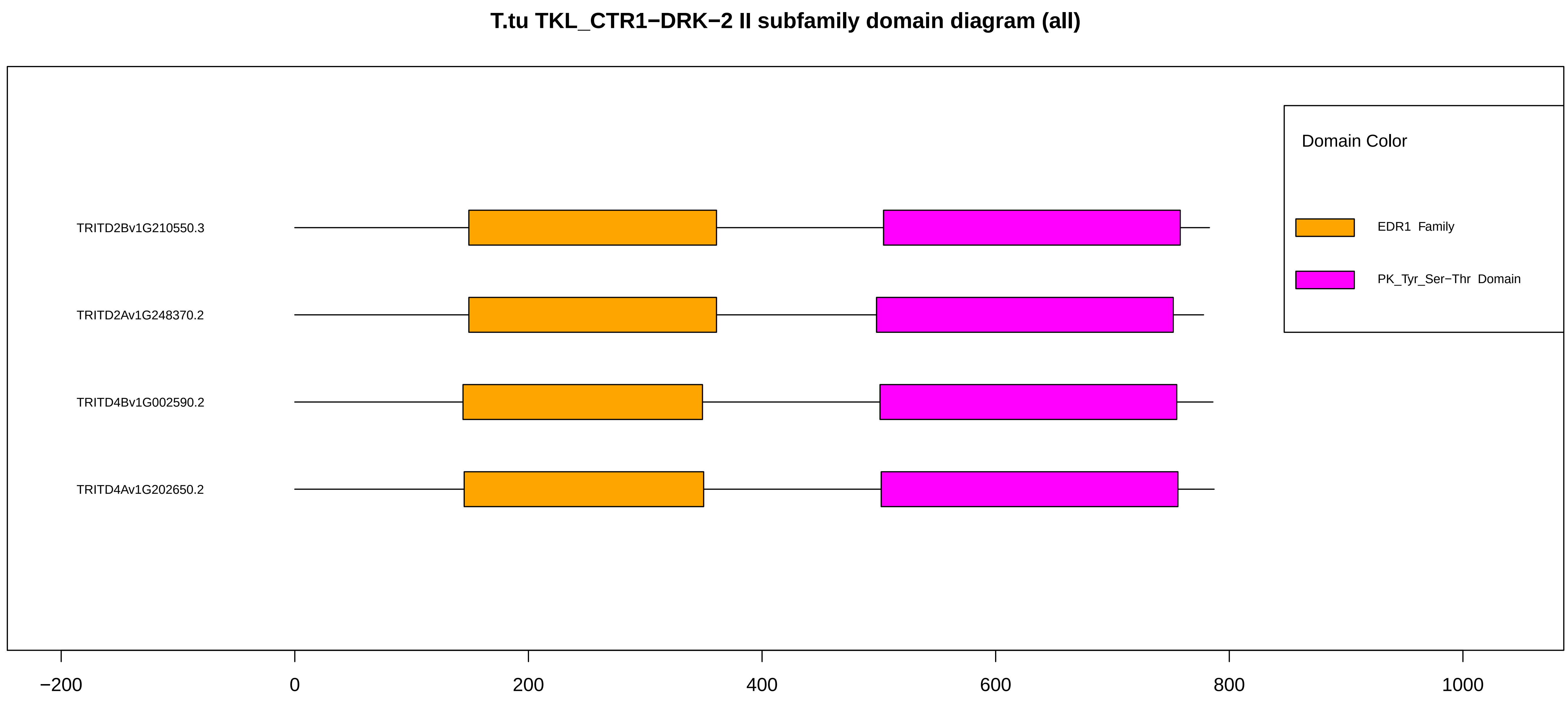
T.tu TKL_CTR1−DRK−2 II subfamily domain diagram (all)
TRITD2Bv1G210550.3
TRITD2Av1G248370.2
TRITD4Bv1G002590.2
TRITD4Av1G202650.2
Domain Color
EDR1 Family
PK_Tyr_Ser−Thr Domain
−200
0
200
400
600
800
1000

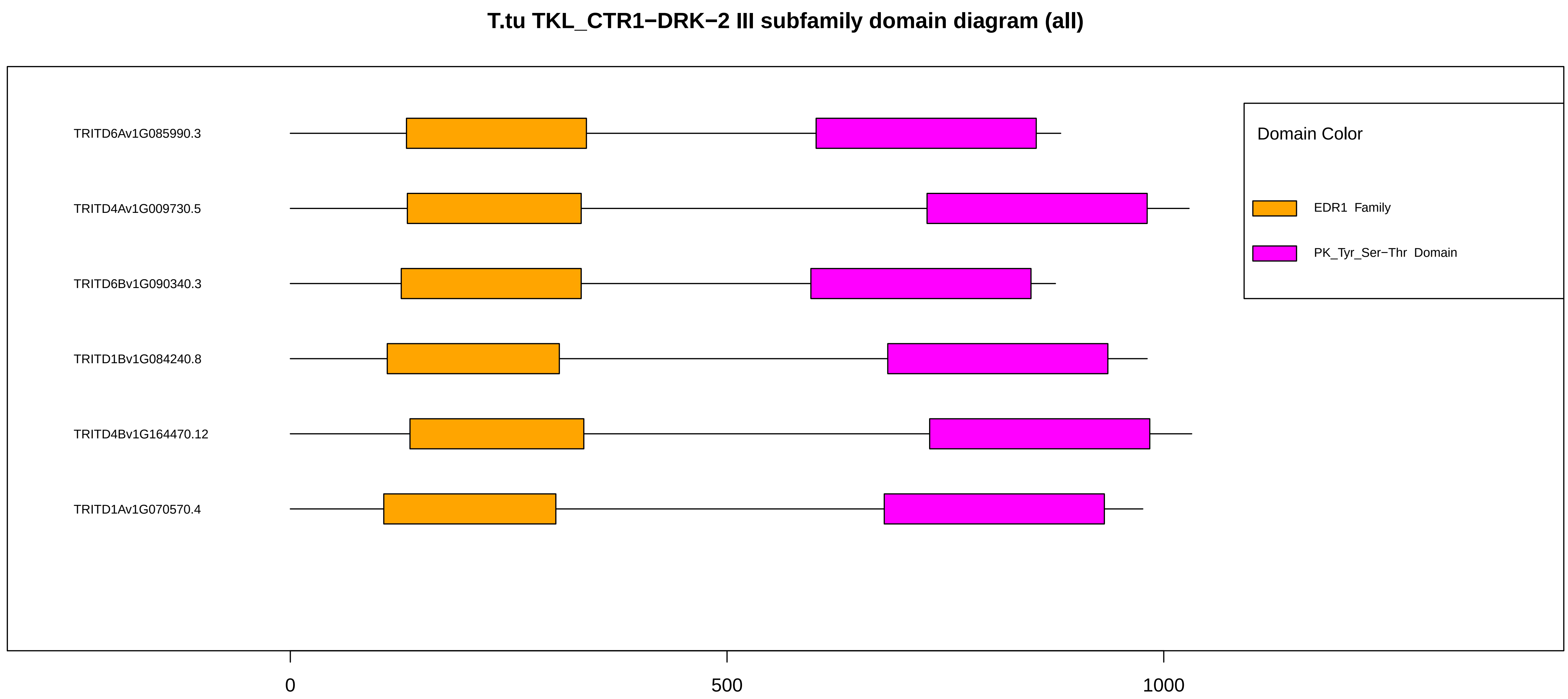
T.tu TKL_CTR1−DRK−2 III subfamily domain diagram (all)
TRITD6Av1G085990.3
TRITD4Av1G009730.5
TRITD6Bv1G090340.3
TRITD1Bv1G084240.8
TRITD4Bv1G164470.12
TRITD1Av1G070570.4
Domain Color
EDR1 Family
PK_Tyr_Ser−Thr Domain
0
500
1000

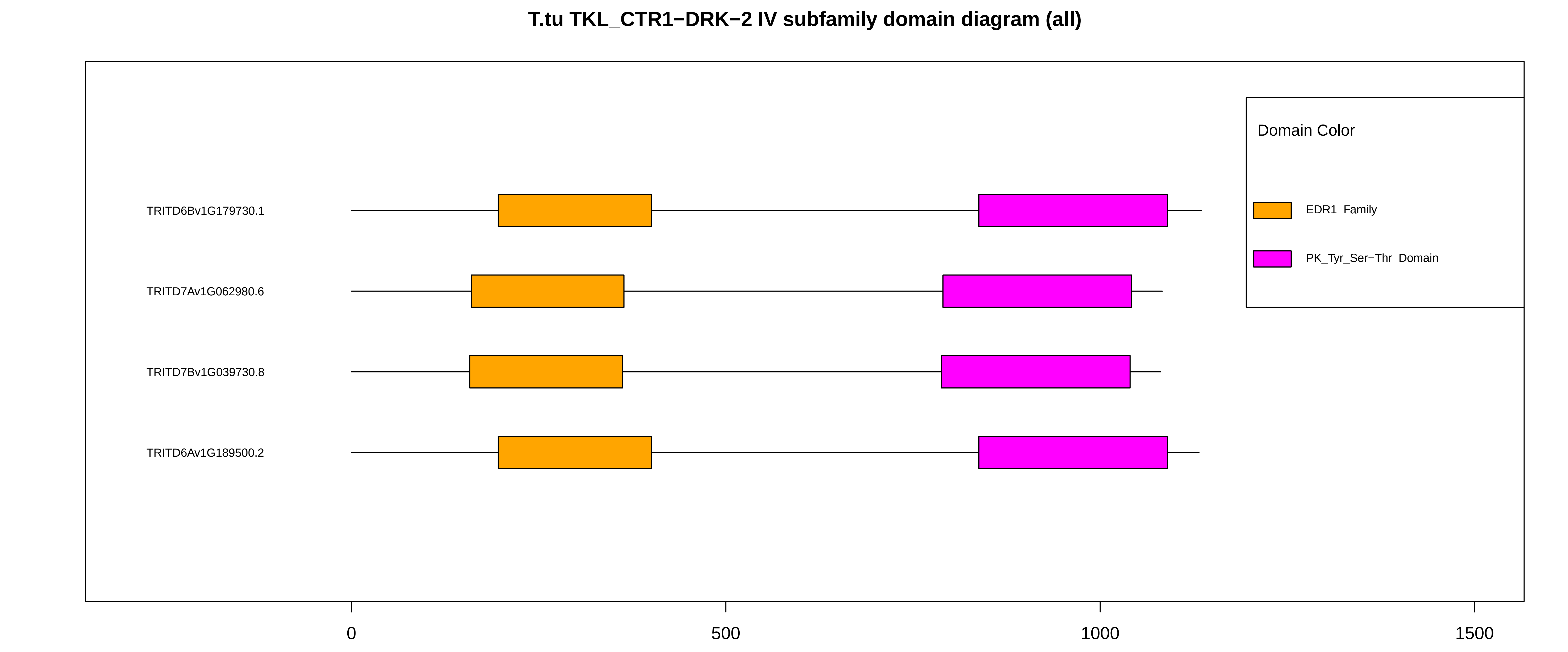

T.tu TKL_CTR1−DRK−2 IV subfamily domain diagram (all)
Domain Color
EDR1 Family
PK_Tyr_Ser−Thr Domain
TRITD6Bv1G179730.1
TRITD7Av1G062980.6
TRITD7Bv1G039730.8
TRITD6Av1G189500.2
0
500
1000
1500

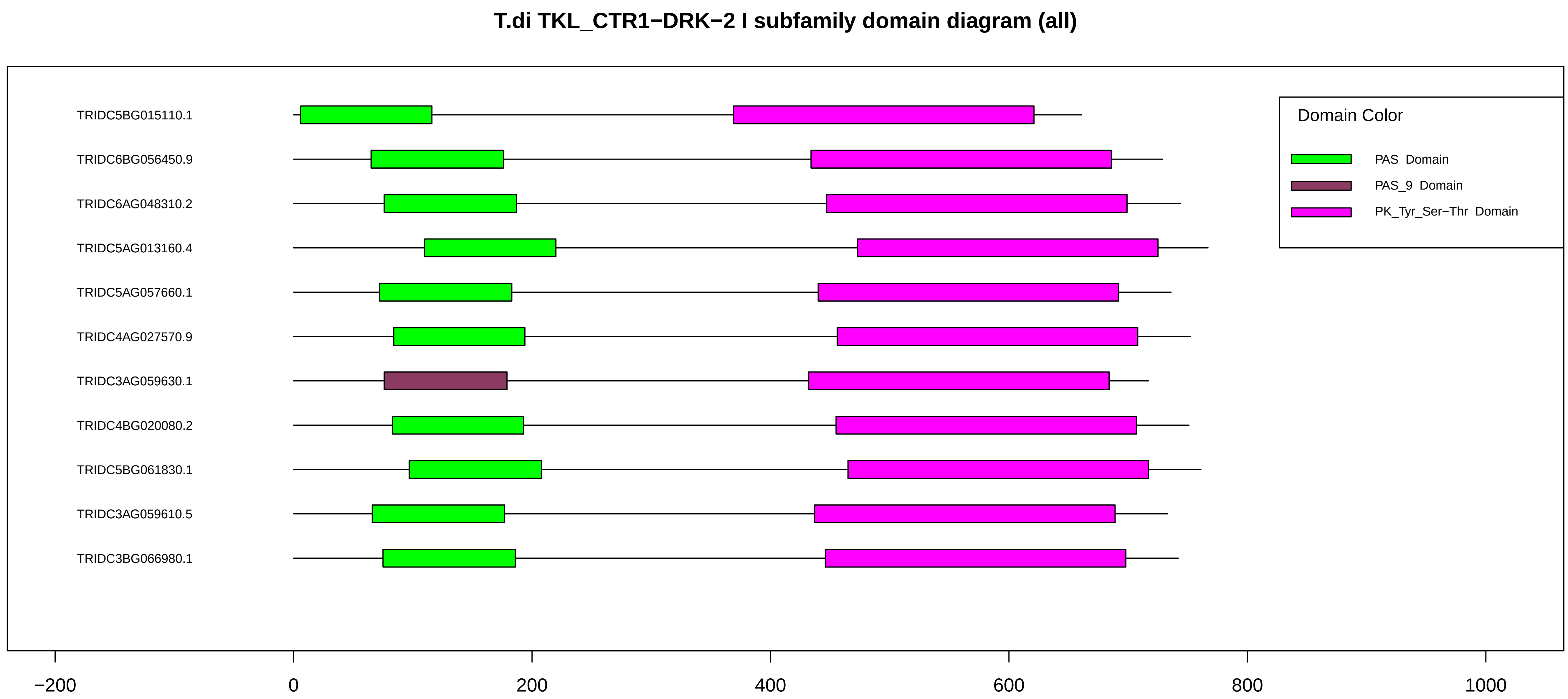
T.di TKL_CTR1−DRK−2 I subfamily domain diagram (all)
TRIDC5BG015110.1
TRIDC6BG056450.9
TRIDC6AG048310.2
TRIDC5AG013160.4
TRIDC5AG057660.1
TRIDC4AG027570.9
TRIDC3AG059630.1
TRIDC4BG020080.2
TRIDC5BG061830.1
TRIDC3AG059610.5
TRIDC3BG066980.1
Domain Color
PAS Domain
PAS_9 Domain
PK_Tyr_Ser−Thr Domain
−200
0
200
400
600
800
1000

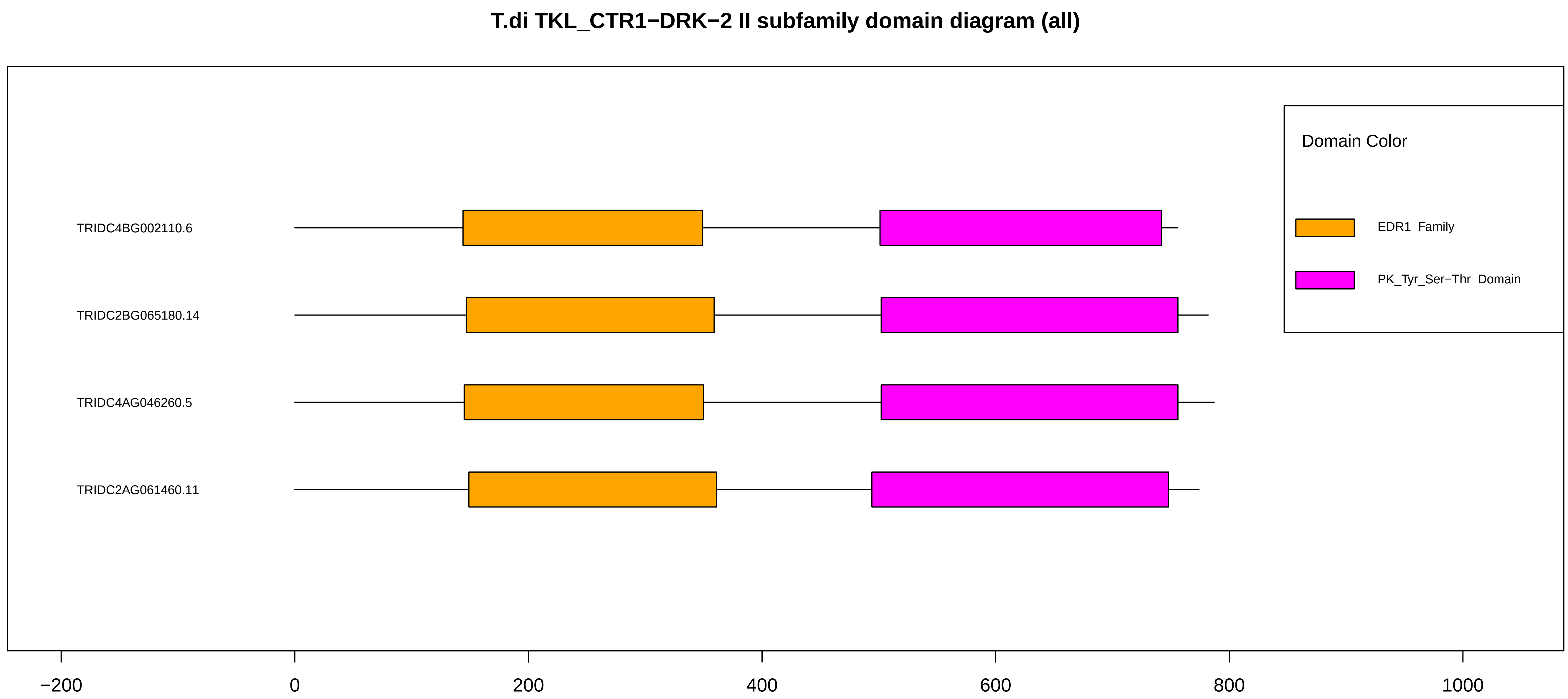
T.di TKL_CTR1−DRK−2 II subfamily domain diagram (all)
TRIDC4BG002110.6
TRIDC2BG065180.14
TRIDC4AG046260.5
TRIDC2AG061460.11
Domain Color
EDR1 Family
PK_Tyr_Ser−Thr Domain
−200
0
200
400
600
800
1000

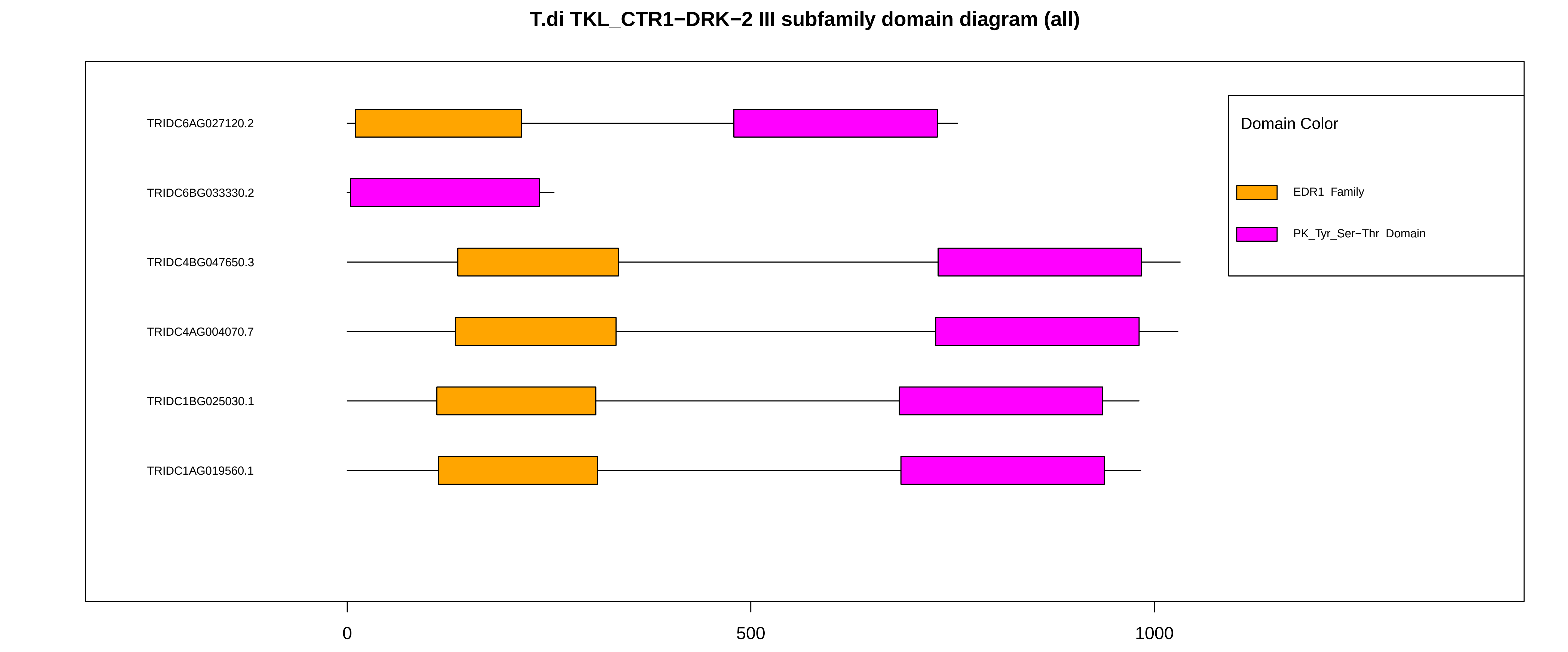
T.di TKL_CTR1−DRK−2 III subfamily domain diagram (all)
TRIDC6AG027120.2
TRIDC6BG033330.2
TRIDC4BG047650.3
TRIDC4AG004070.7
TRIDC1BG025030.1
TRIDC1AG019560.1
Domain Color
EDR1 Family
PK_Tyr_Ser−Thr Domain
0
500
1000

T.di TKL_CTR1−DRK−2 IV subfamily domain diagram (all)

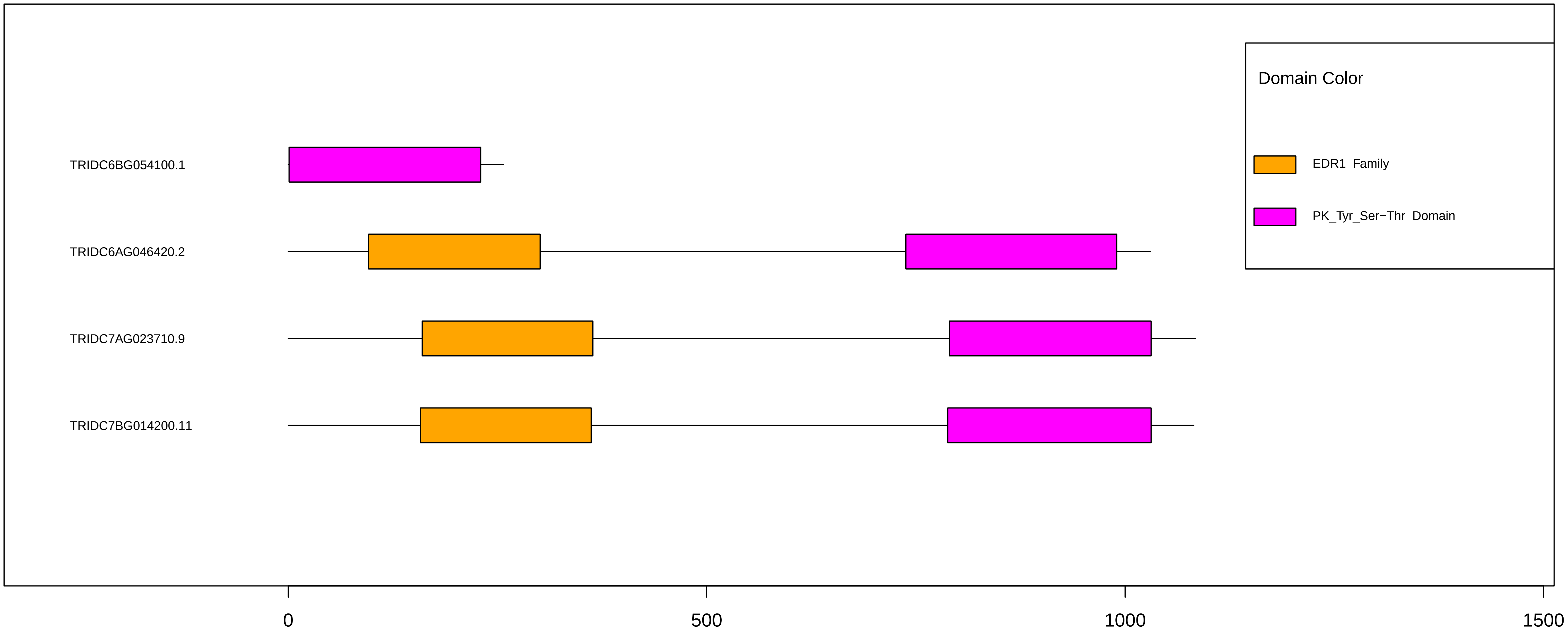

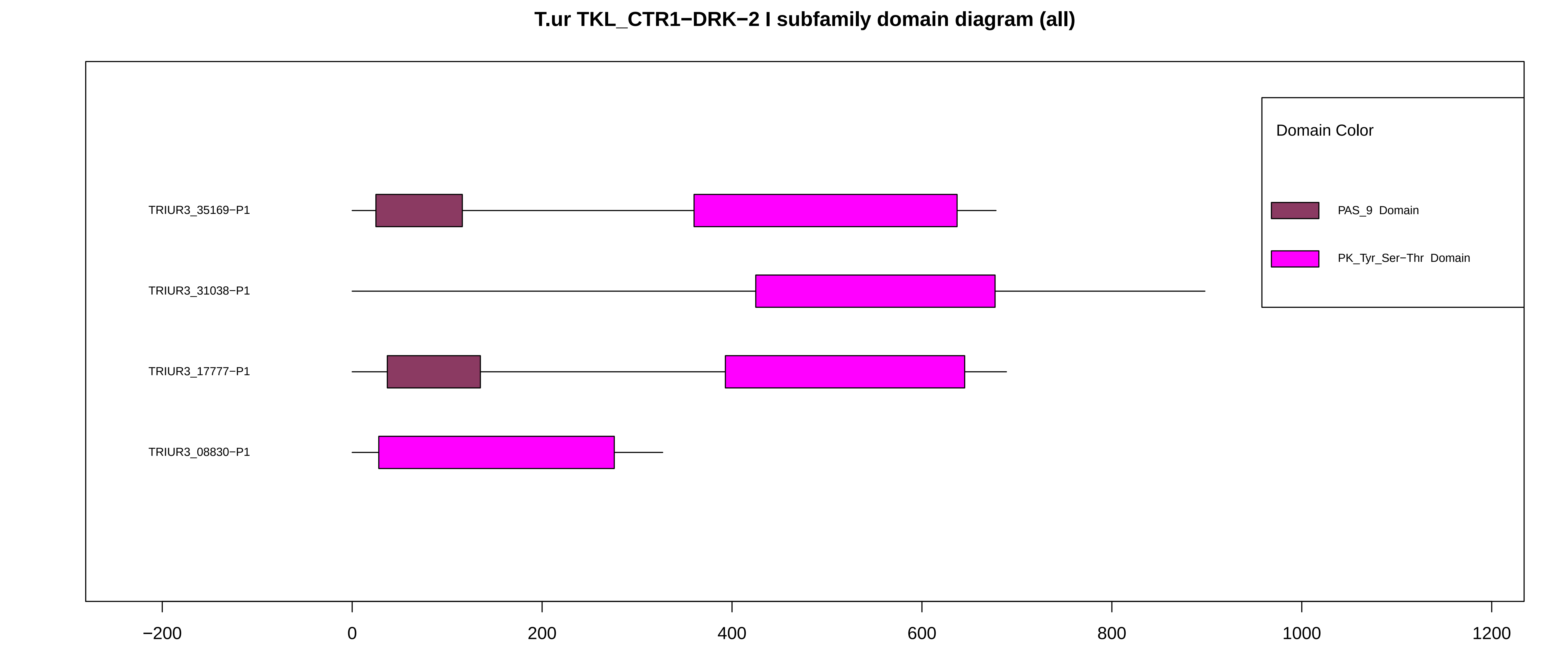
T.ur TKL_CTR1−DRK−2 I subfamily domain diagram (all)
TRIUR3_35169−P1
TRIUR3_31038−P1
TRIUR3_17777−P1
TRIUR3_08830−P1
Domain Color
PAS_9 Domain
PK_Tyr_Ser−Thr Domain
−200
0
200
400
600
800
1000
1200

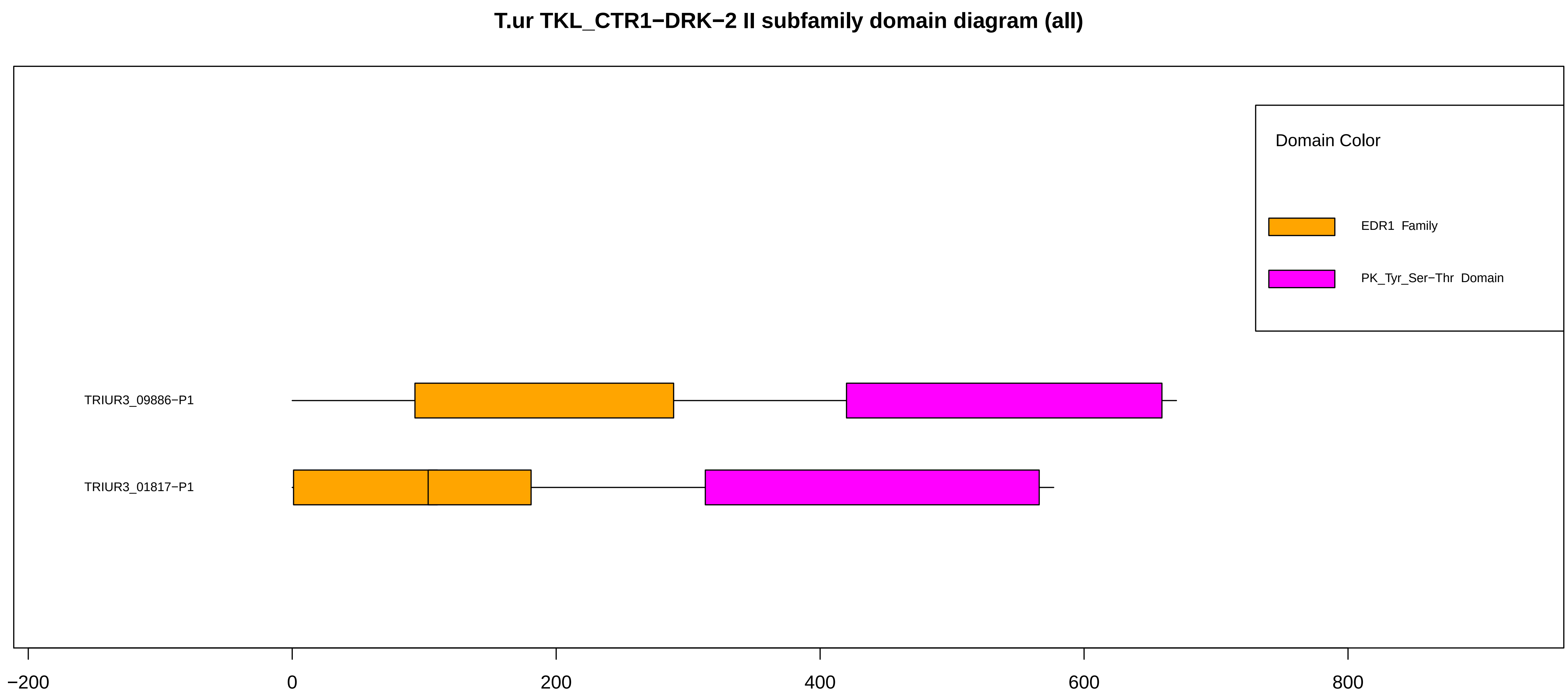
T.ur TKL_CTR1−DRK−2 II subfamily domain diagram (all)
Domain Color
EDR1 Family
PK_Tyr_Ser−Thr Domain
TRIUR3_09886−P1
TRIUR3_01817−P1
−200
0
200
400
600
800

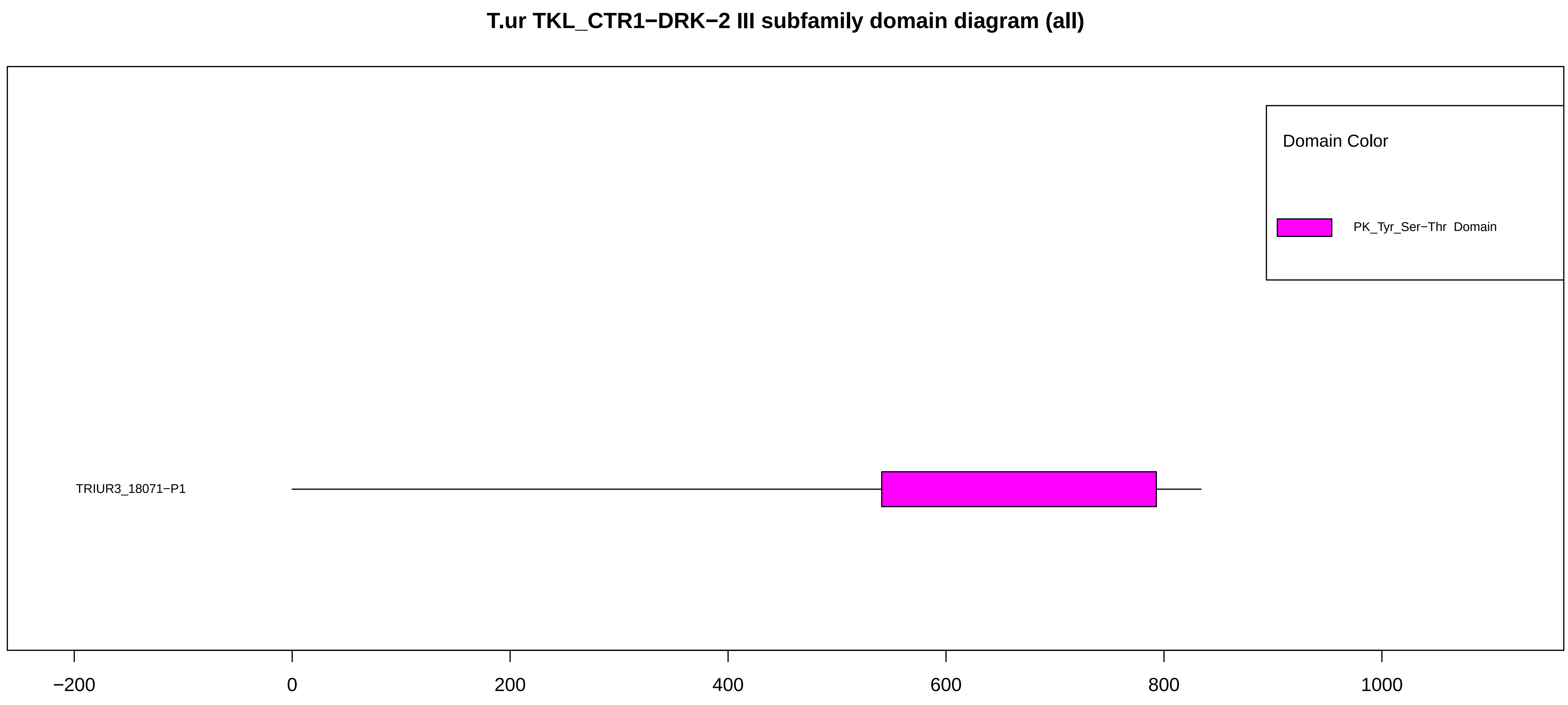
T.ur TKL_CTR1−DRK−2 III subfamily domain diagram (all)
Domain Color
PK_Tyr_Ser−Thr Domain
TRIUR3_18071−P1
−200
0
200
400
600
800
1000

T.ur TKL_CTR1−DRK−2 IV subfamily domain diagram (all)

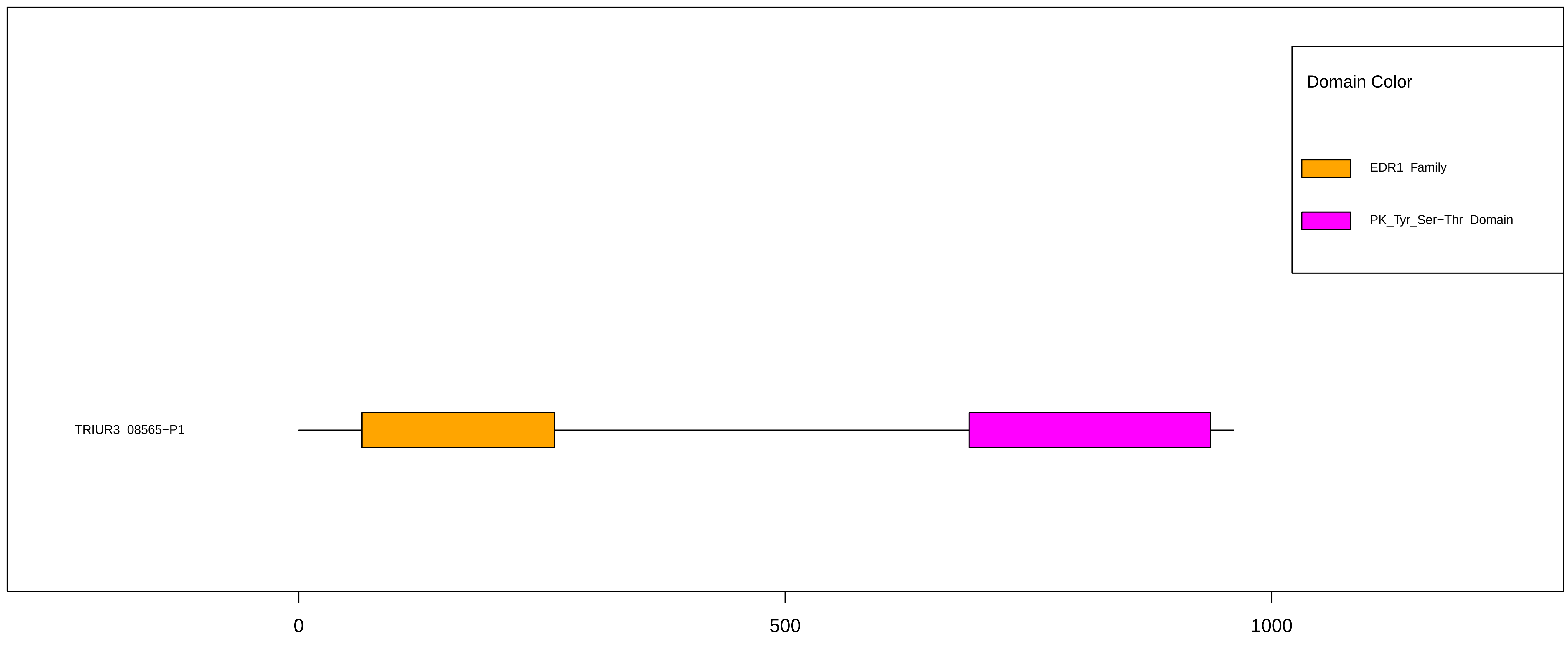

T.ur TKL_CTR1−DRK−2 (excluding in phylogenetic analysis) domain diagram (all)

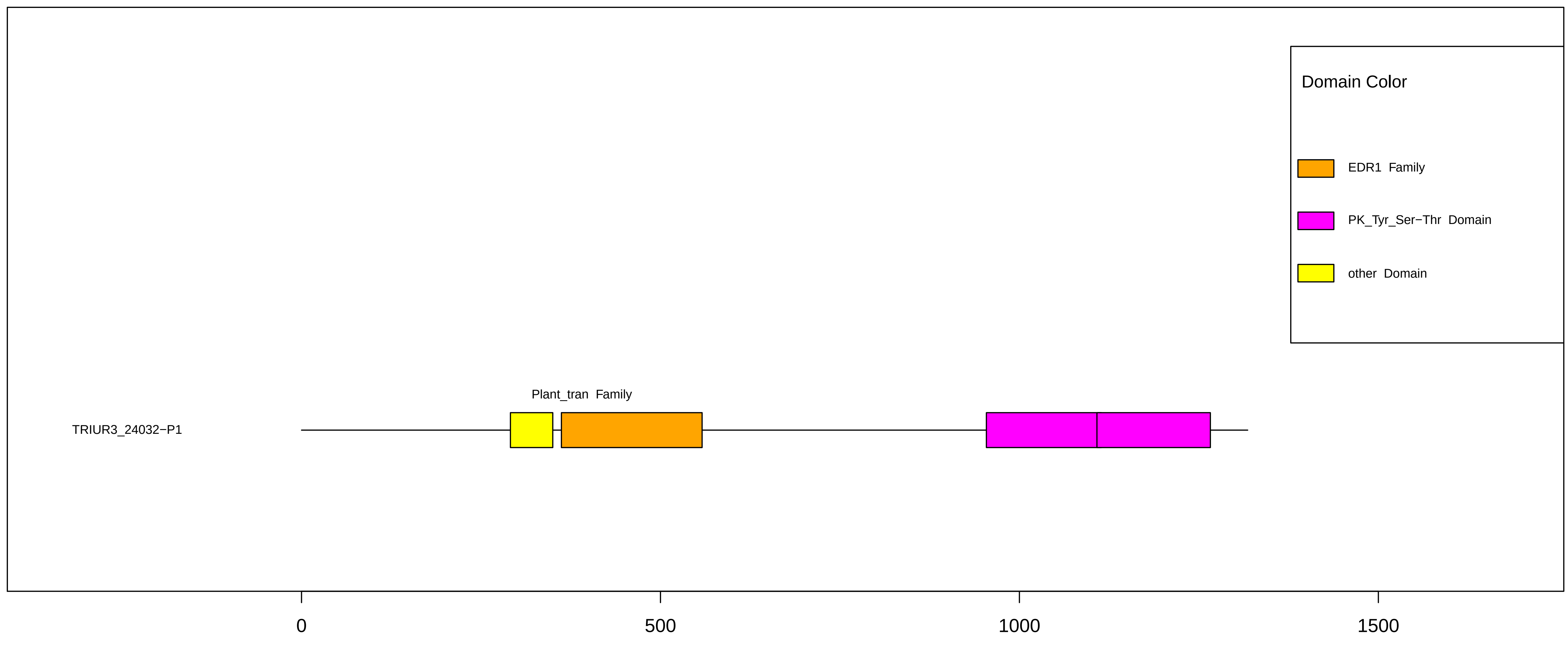

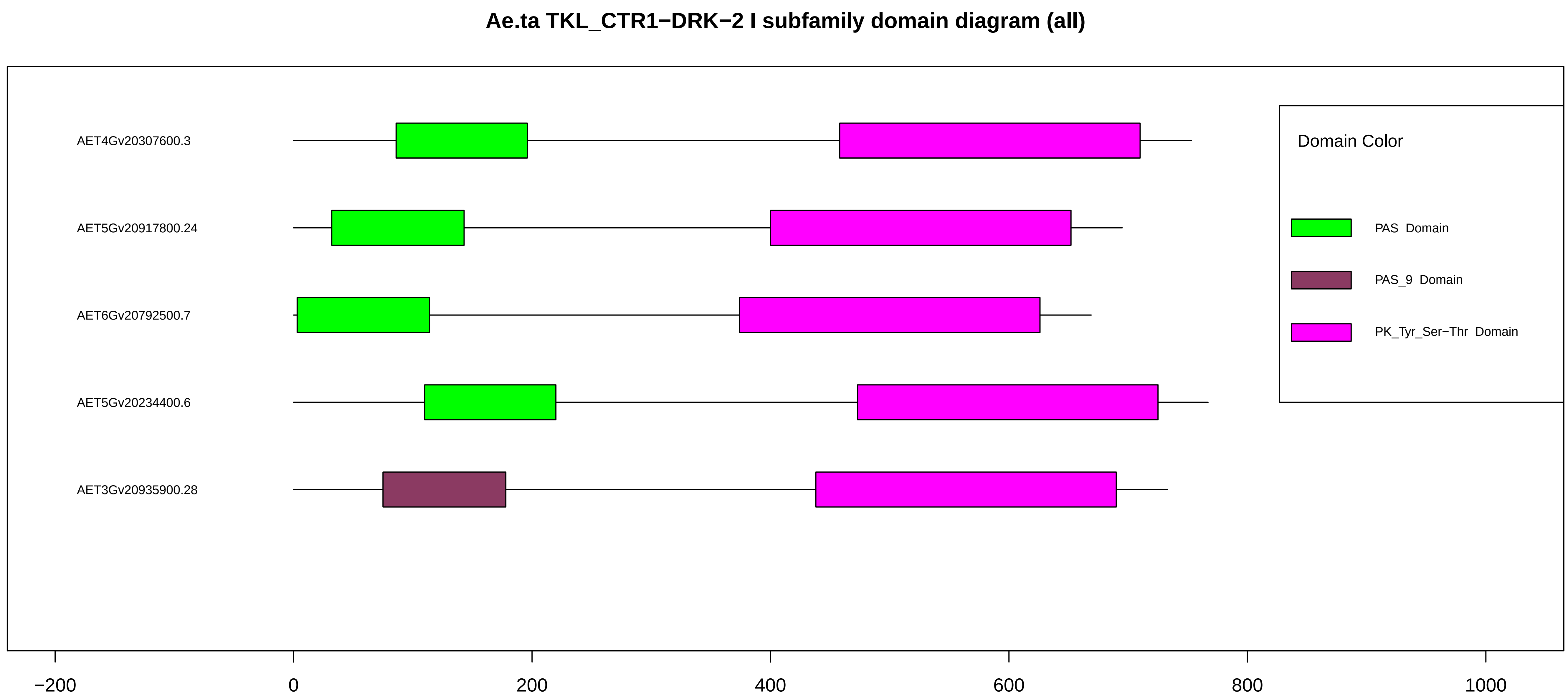

Ae.ta TKL_CTR1−DRK−2 I subfamily domain diagram (all)
AET4Gv20307600.3
AET5Gv20917800.24
AET6Gv20792500.7
AET5Gv20234400.6
AET3Gv20935900.28
Domain Color
PAS Domain
PAS_9 Domain
PK_Tyr_Ser−Thr Domain
−200
0
200
400
600
800
1000

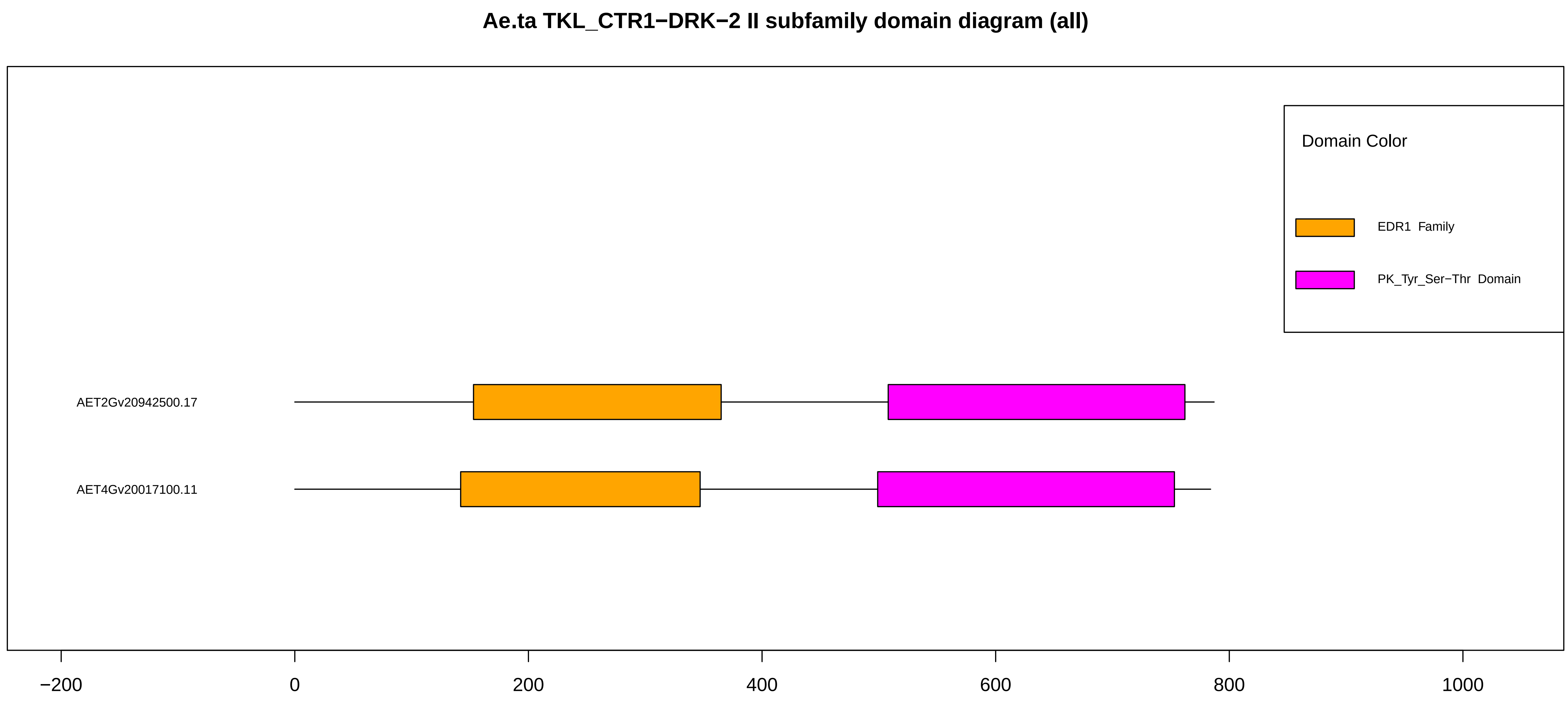
Ae.ta TKL_CTR1−DRK−2 II subfamily domain diagram (all)
Domain Color
EDR1 Family
PK_Tyr_Ser−Thr Domain
AET2Gv20942500.17
AET4Gv20017100.11
−200
0
200
400
600
800
1000

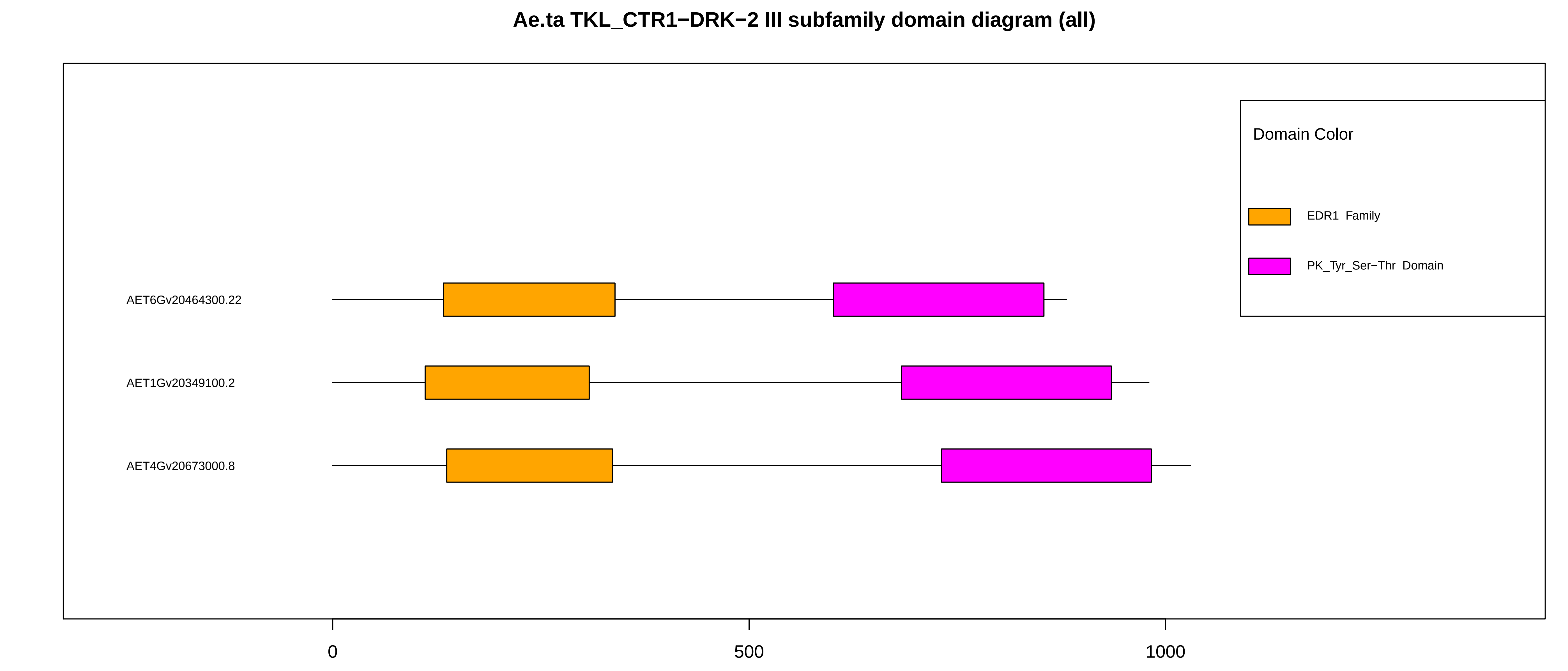

Ae.ta TKL_CTR1−DRK−2 III subfamily domain diagram (all)
Domain Color
EDR1 Family
PK_Tyr_Ser−Thr Domain
AET6Gv20464300.22
AET1Gv20349100.2
AET4Gv20673000.8
0
500
1000

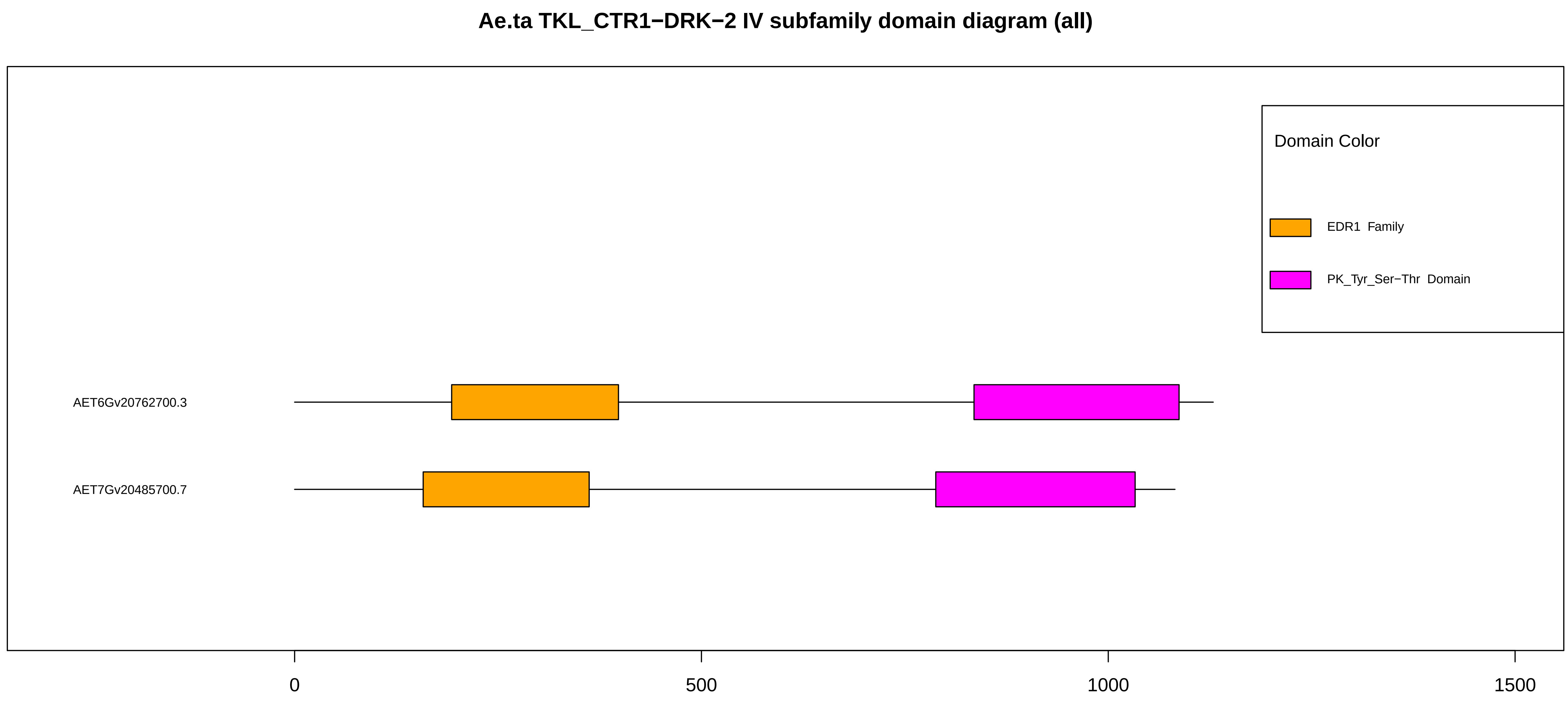
Ae.ta TKL_CTR1−DRK−2 IV subfamily domain diagram (all)
Domain Color
EDR1 Family
PK_Tyr_Ser−Thr Domain
AET6Gv20762700.3
AET7Gv20485700.7
0
500
1000
1500

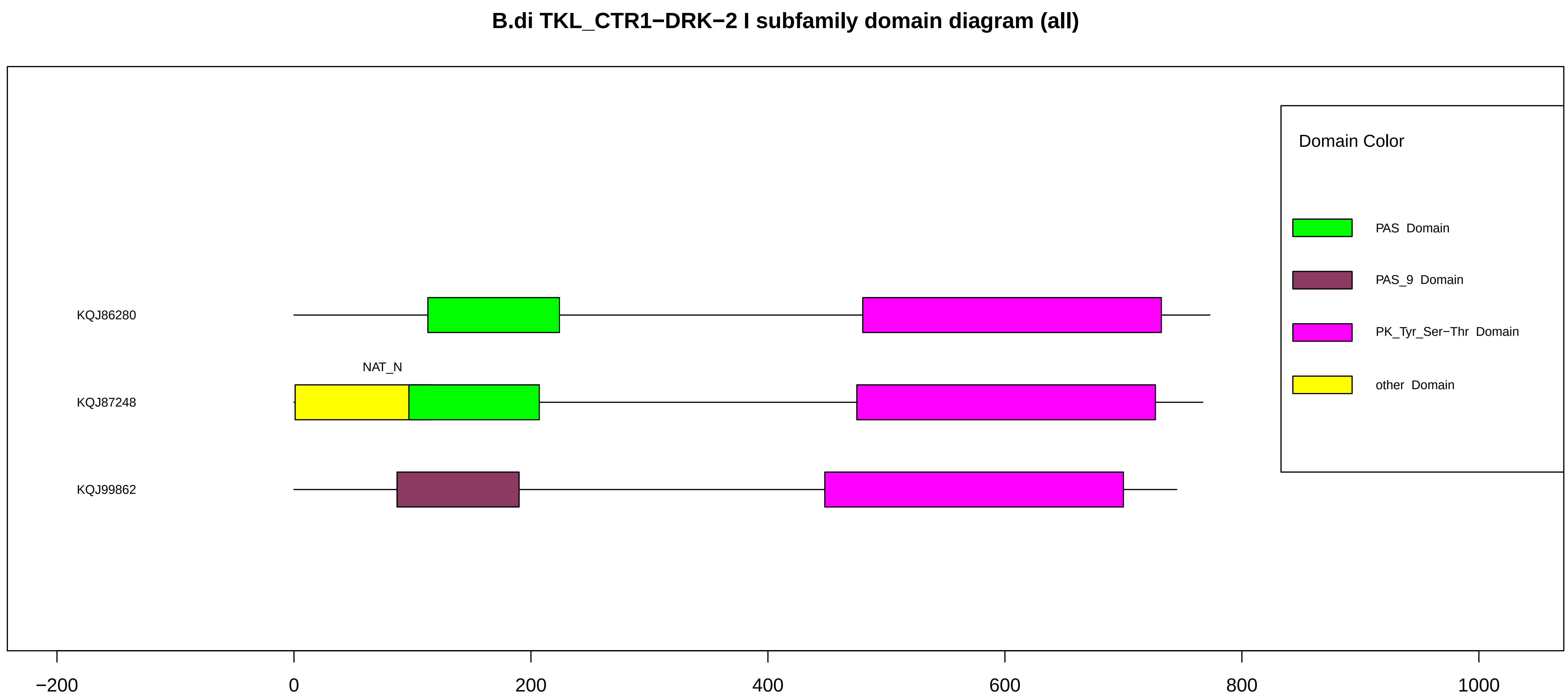
B.di TKL_CTR1−DRK−2 I subfamily domain diagram (all)
KQJ86280
KQJ87248
KQJ99862
NAT_N
Domain Color
PAS Domain
PAS_9 Domain
PK_Tyr_Ser−Thr Domain
other Domain
−200
0
200
400
600
800
1000

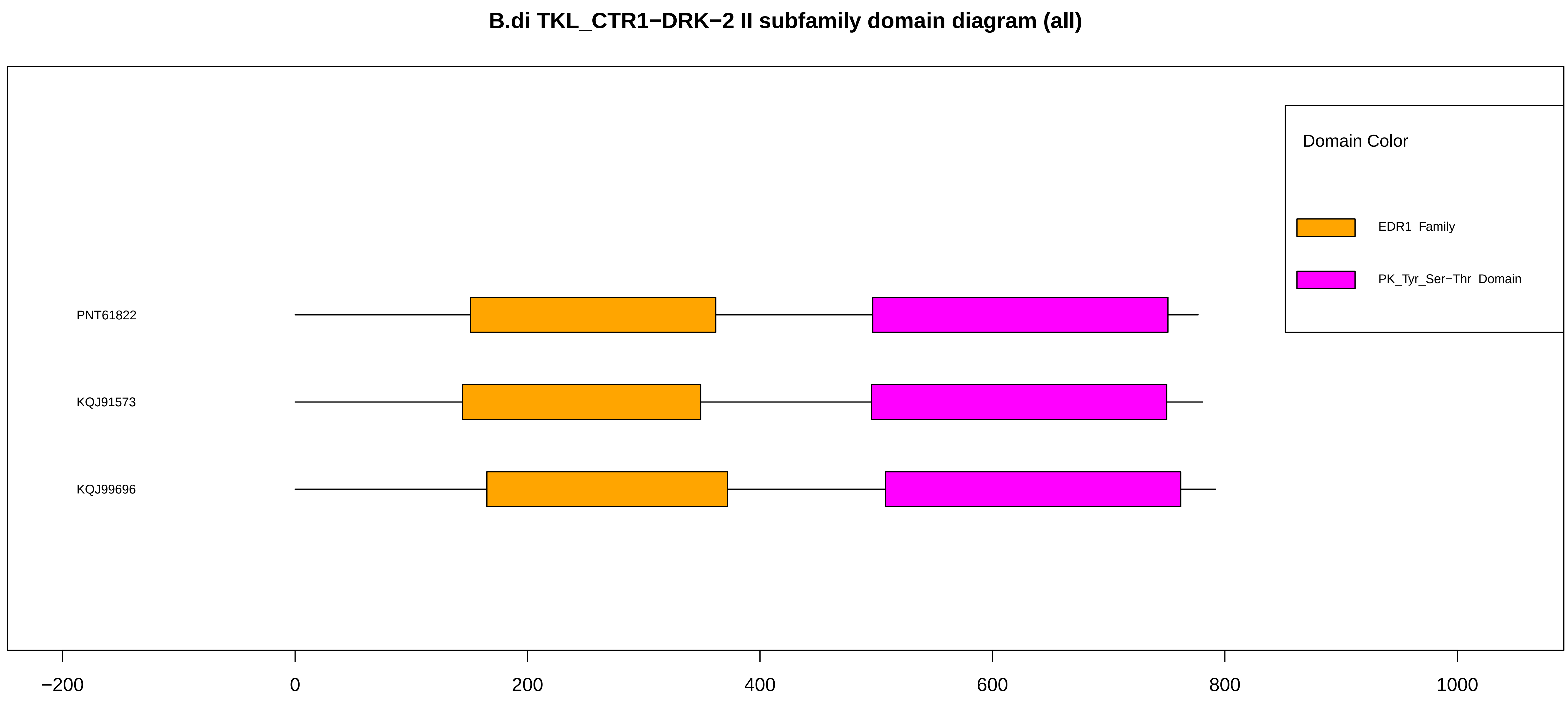
B.di TKL_CTR1−DRK−2 II subfamily domain diagram (all)
Domain Color
EDR1 Family
PK_Tyr_Ser−Thr Domain
PNT61822
KQJ91573
KQJ99696
−200
0
200
400
600
800
1000

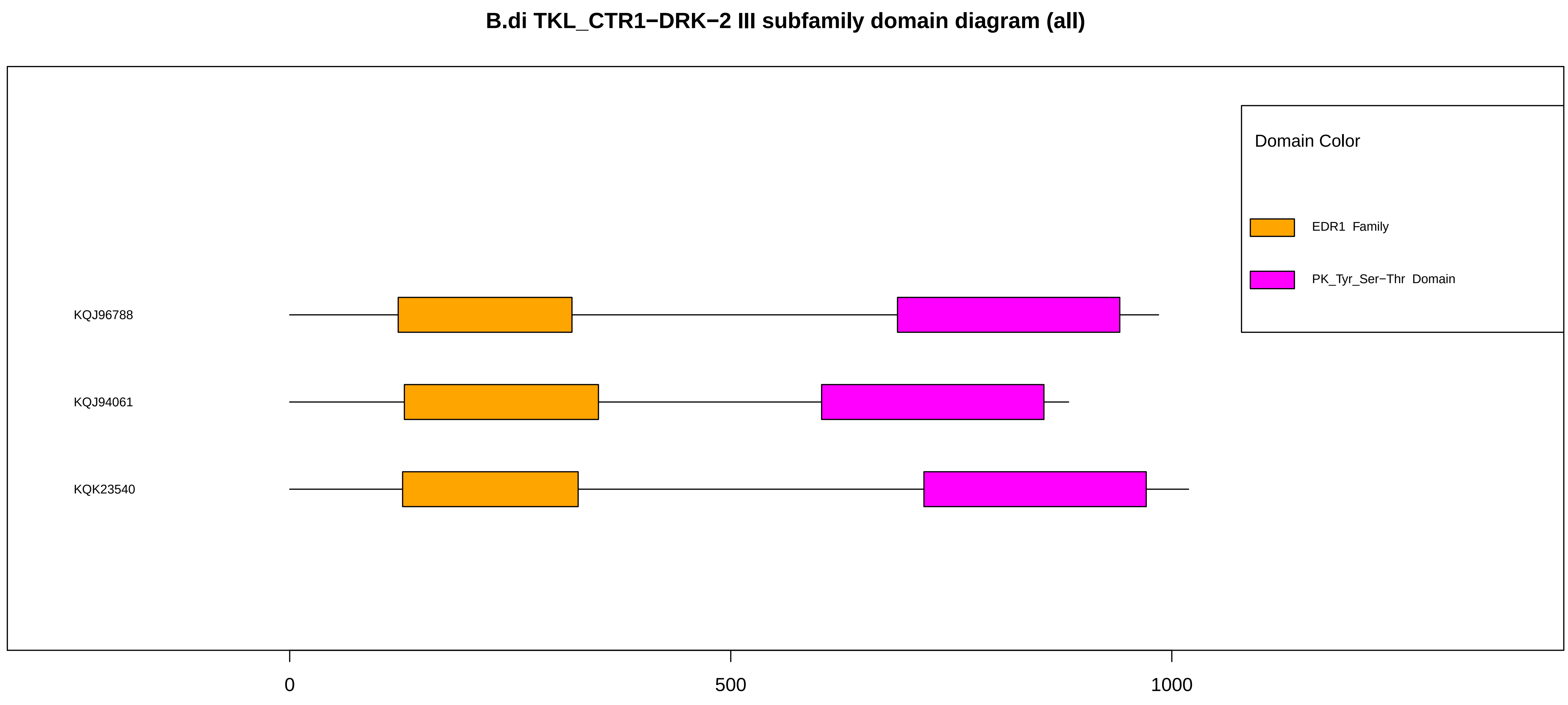
B.di TKL_CTR1−DRK−2 III subfamily domain diagram (all)
Domain Color
EDR1 Family
PK_Tyr_Ser−Thr Domain
KQJ96788
KQJ94061
KQK23540
0
500
1000

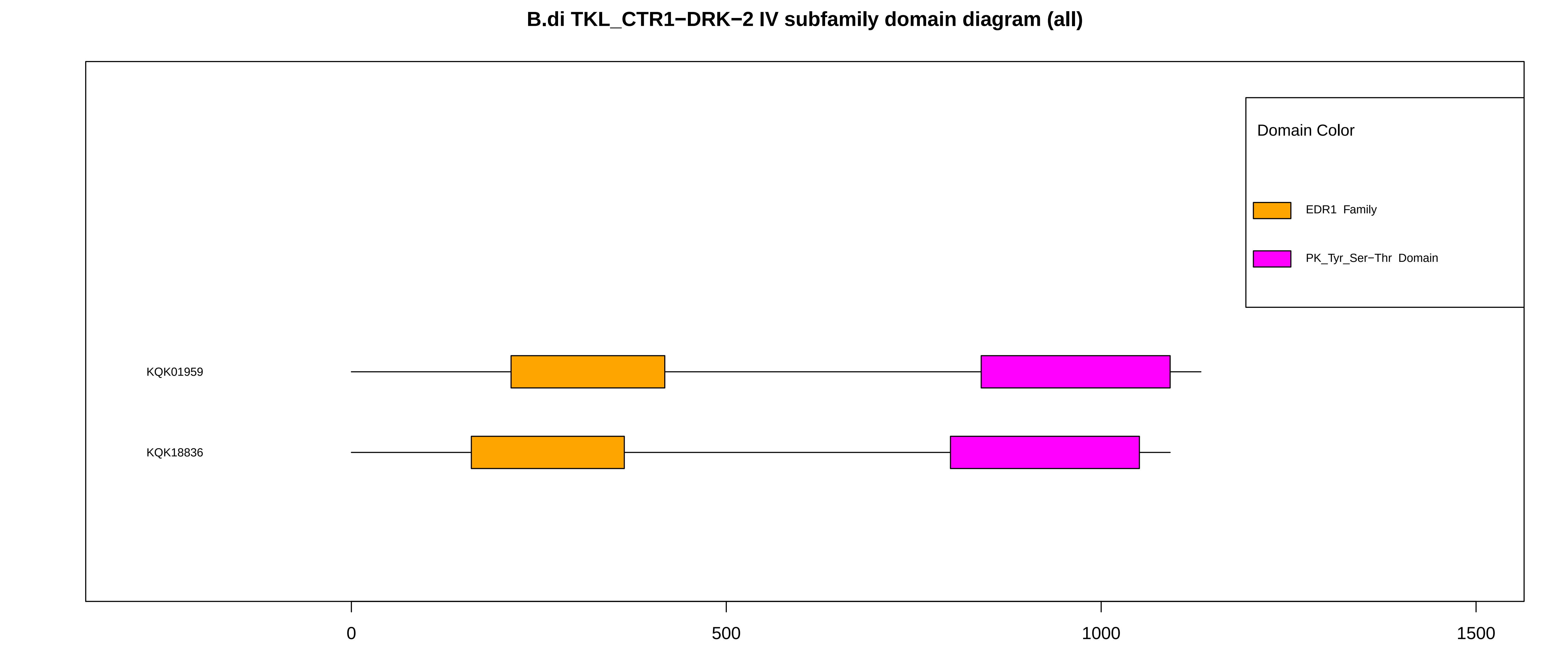

B.di TKL_CTR1−DRK−2 IV subfamily domain diagram (all)
Domain Color
EDR1 Family
PK_Tyr_Ser−Thr Domain
KQK01959
KQK18836
0
500
1000
1500

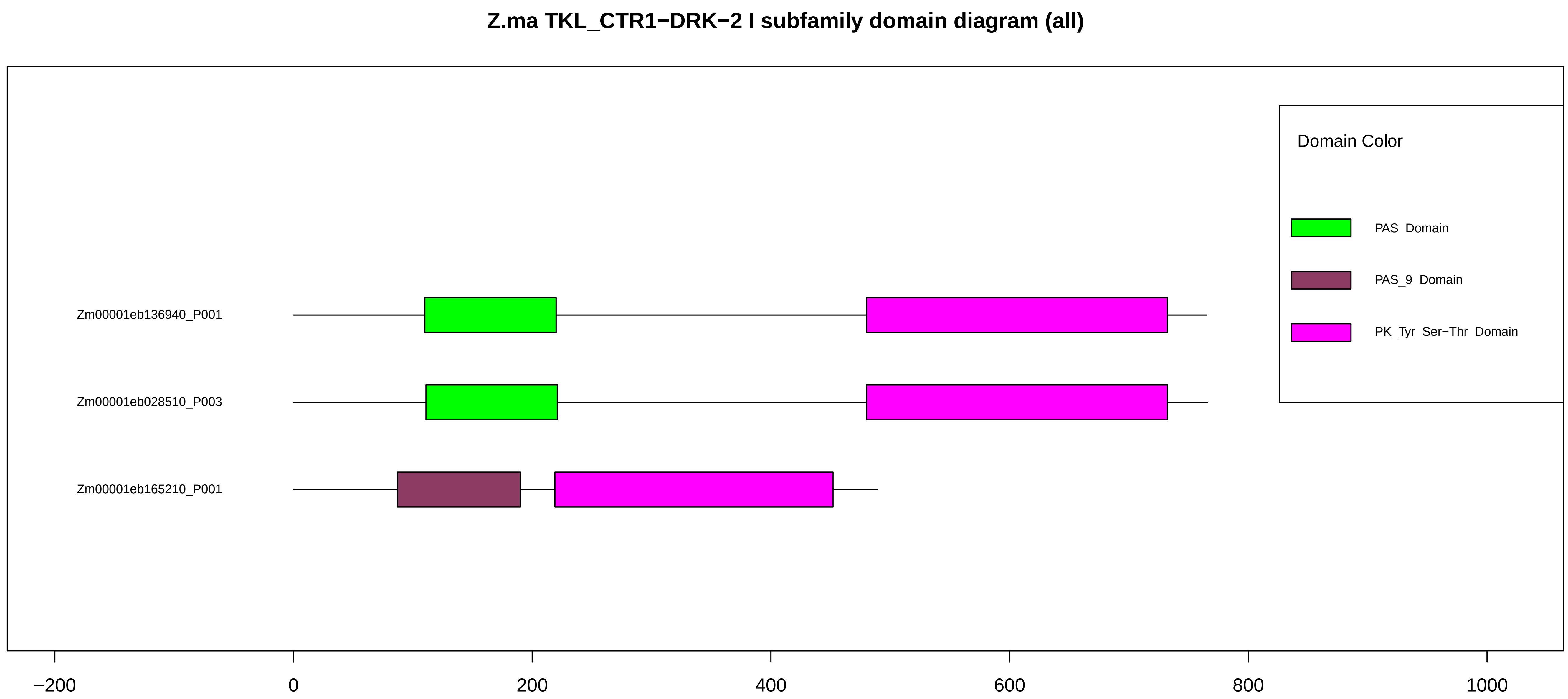
Z.ma TKL_CTR1−DRK−2 I subfamily domain diagram (all)
Domain Color
PAS Domain
PAS_9 Domain
PK_Tyr_Ser−Thr Domain
Zm00001eb136940_P001
Zm00001eb028510_P003
Zm00001eb165210_P001
−200
0
200
400
600
800
1000

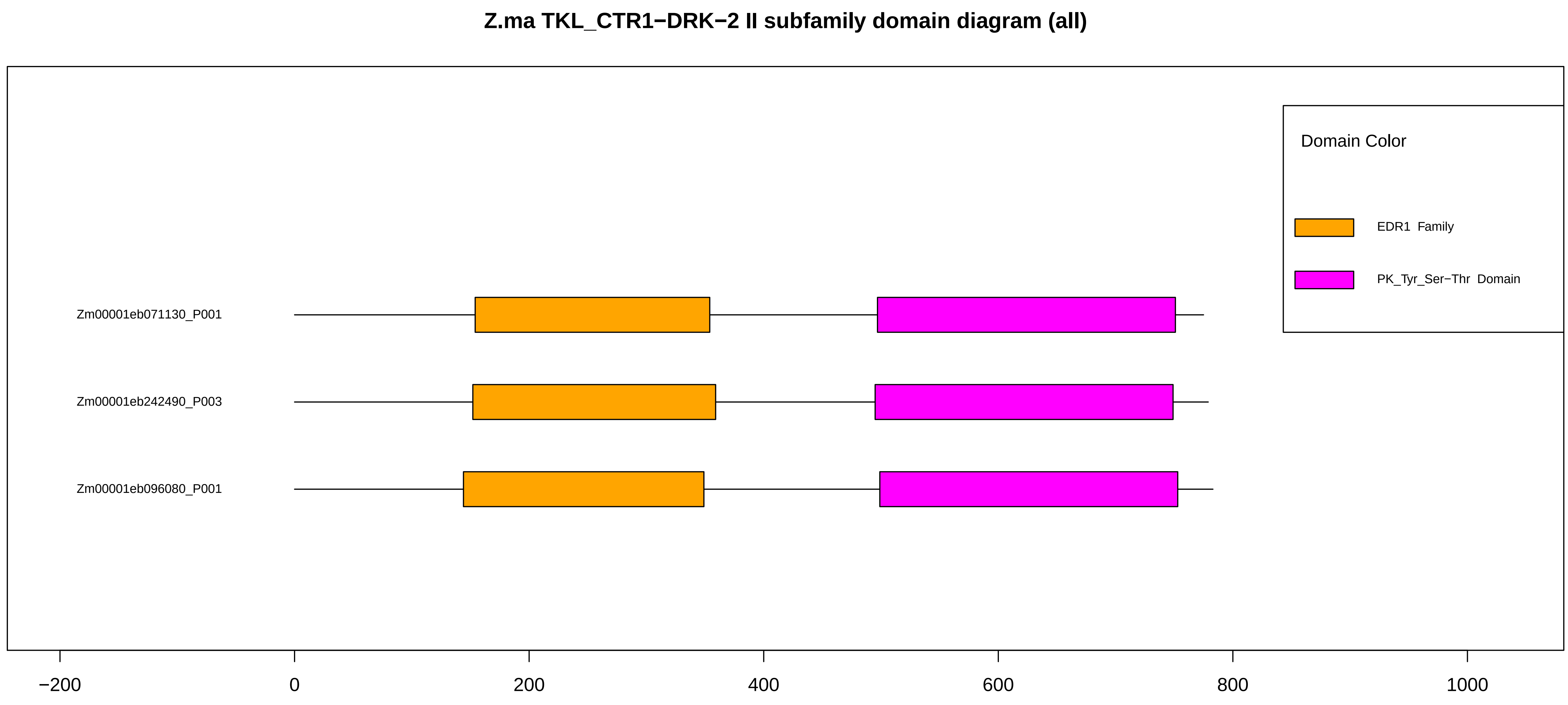
Z.ma TKL_CTR1−DRK−2 II subfamily domain diagram (all)
Domain Color
EDR1 Family
PK_Tyr_Ser−Thr Domain
Zm00001eb071130_P001
Zm00001eb242490_P003
Zm00001eb096080_P001
−200
0
200
400
600
800
1000

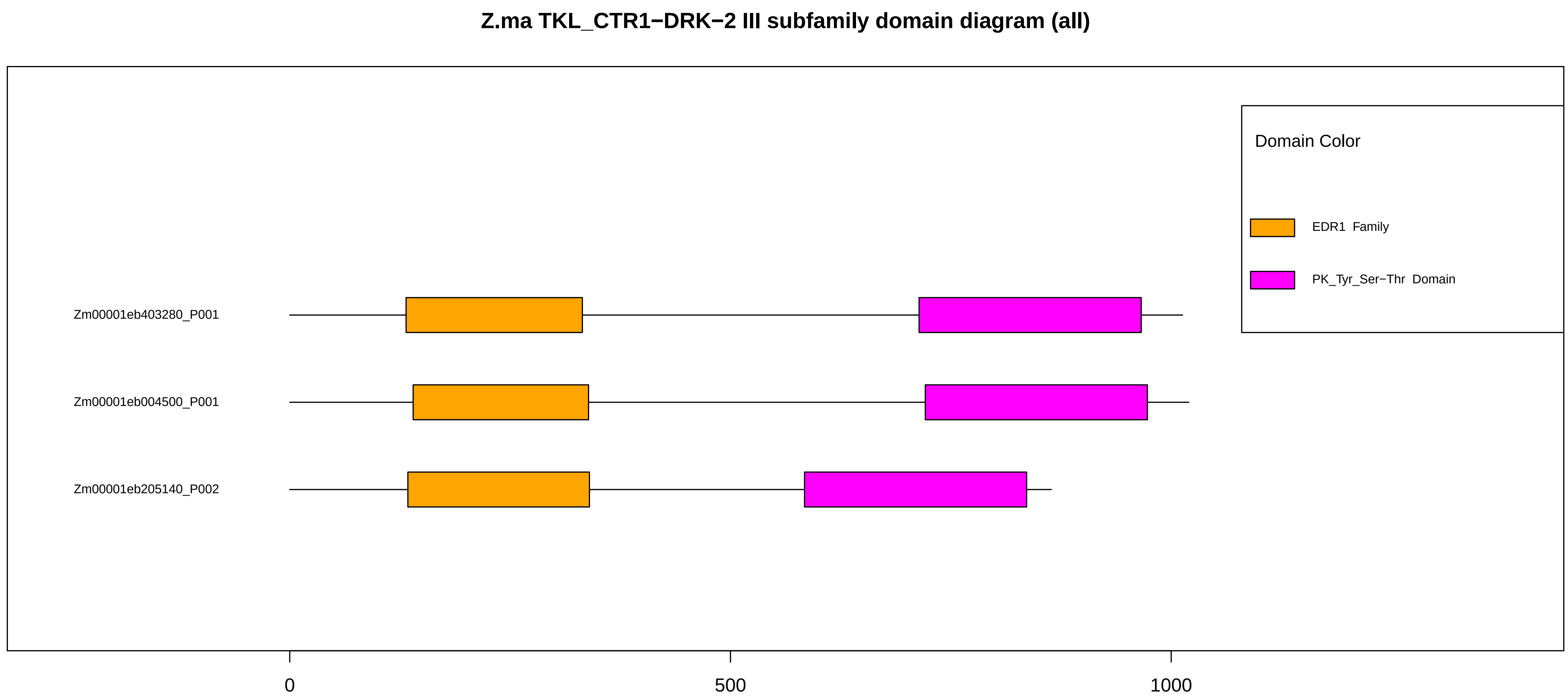
Z.ma TKL_CTR1−DRK−2 III subfamily domain diagram (all)
Domain Color
EDR1 Family
PK_Tyr_Ser−Thr Domain
Zm00001eb403280_P001
Zm00001eb004500_P001
Zm00001eb205140_P002
0
500
1000

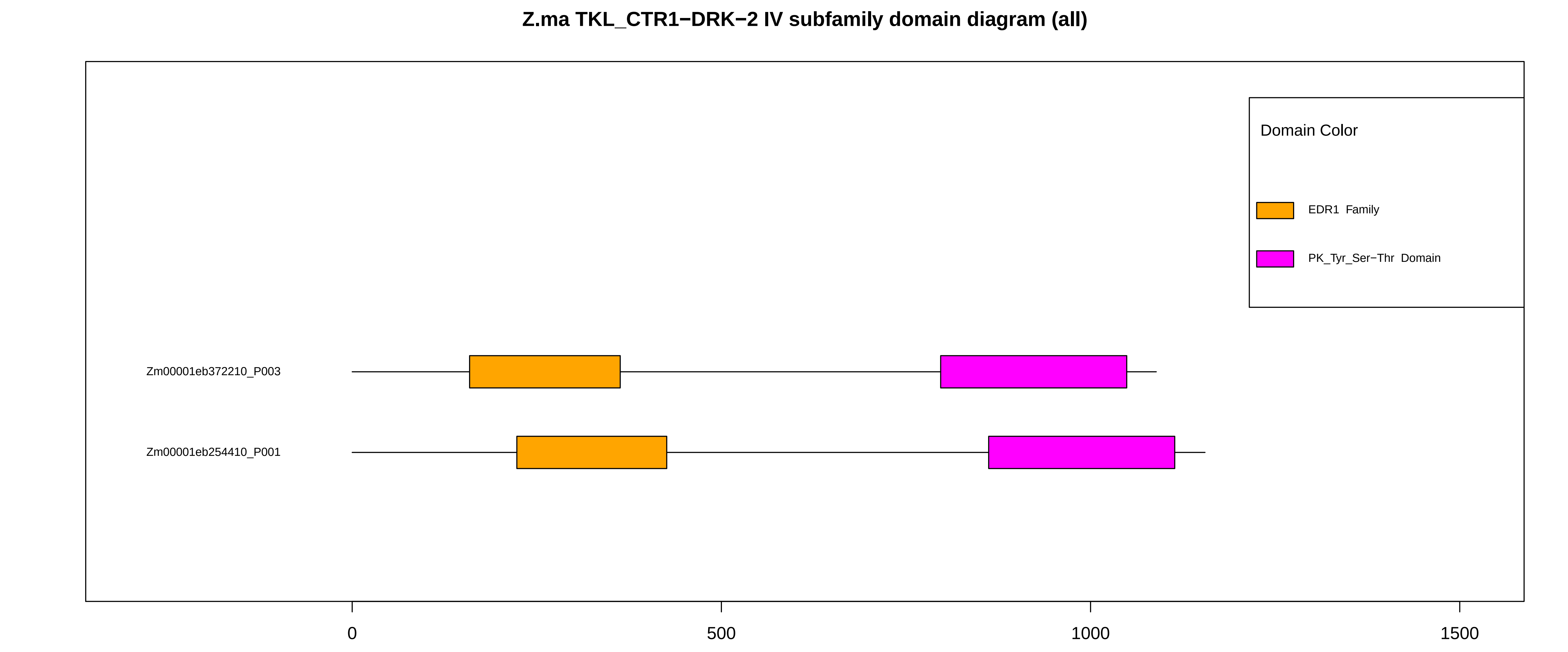
Z.ma TKL_CTR1−DRK−2 IV subfamily domain diagram (all)
Domain Color
EDR1 Family
PK_Tyr_Ser−Thr Domain
Zm00001eb372210_P003
Zm00001eb254410_P001
0
500
1000
1500

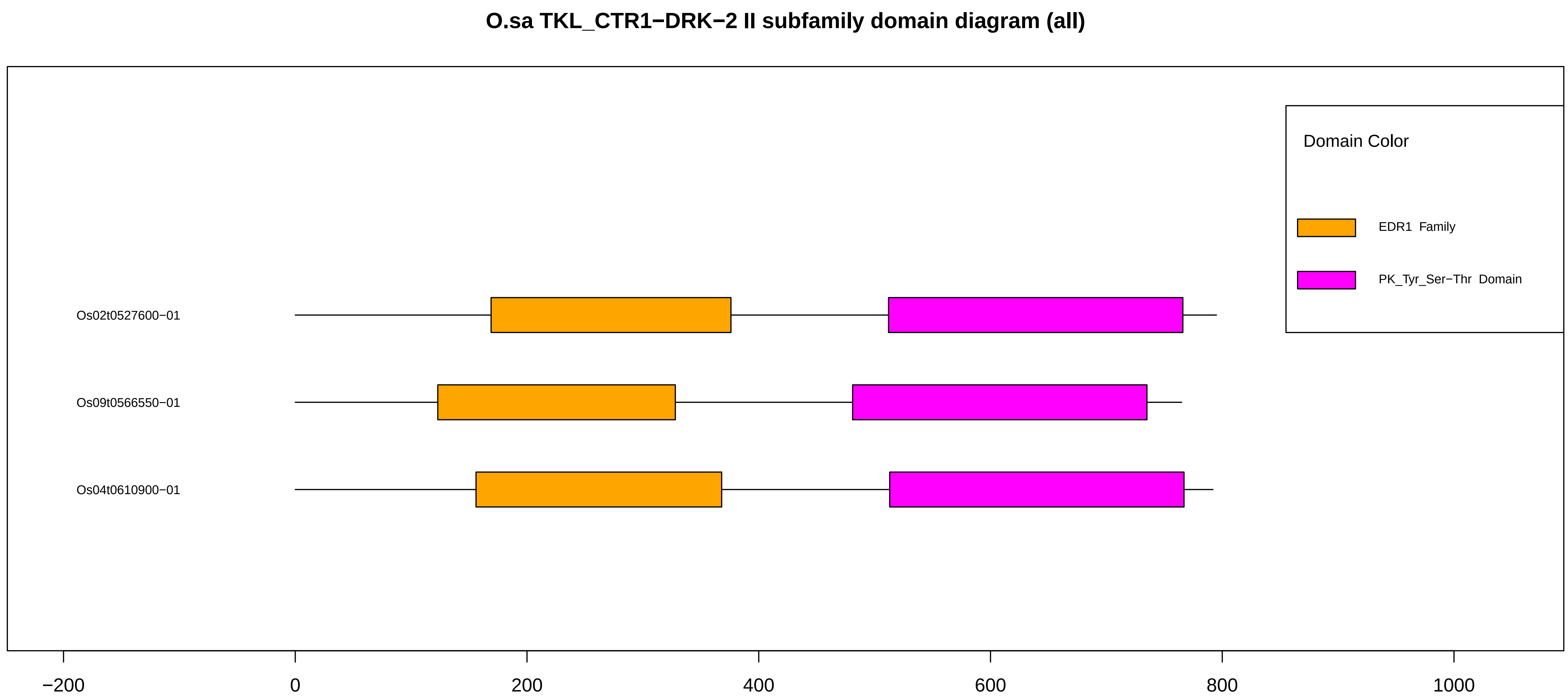
O.sa TKL_CTR1−DRK−2 II subfamily domain diagram (all)
Domain Color
EDR1 Family
PK_Tyr_Ser−Thr Domain
Os02t0527600−01
Os09t0566550−01
Os04t0610900−01
−200
0
200
400
600
800
1000

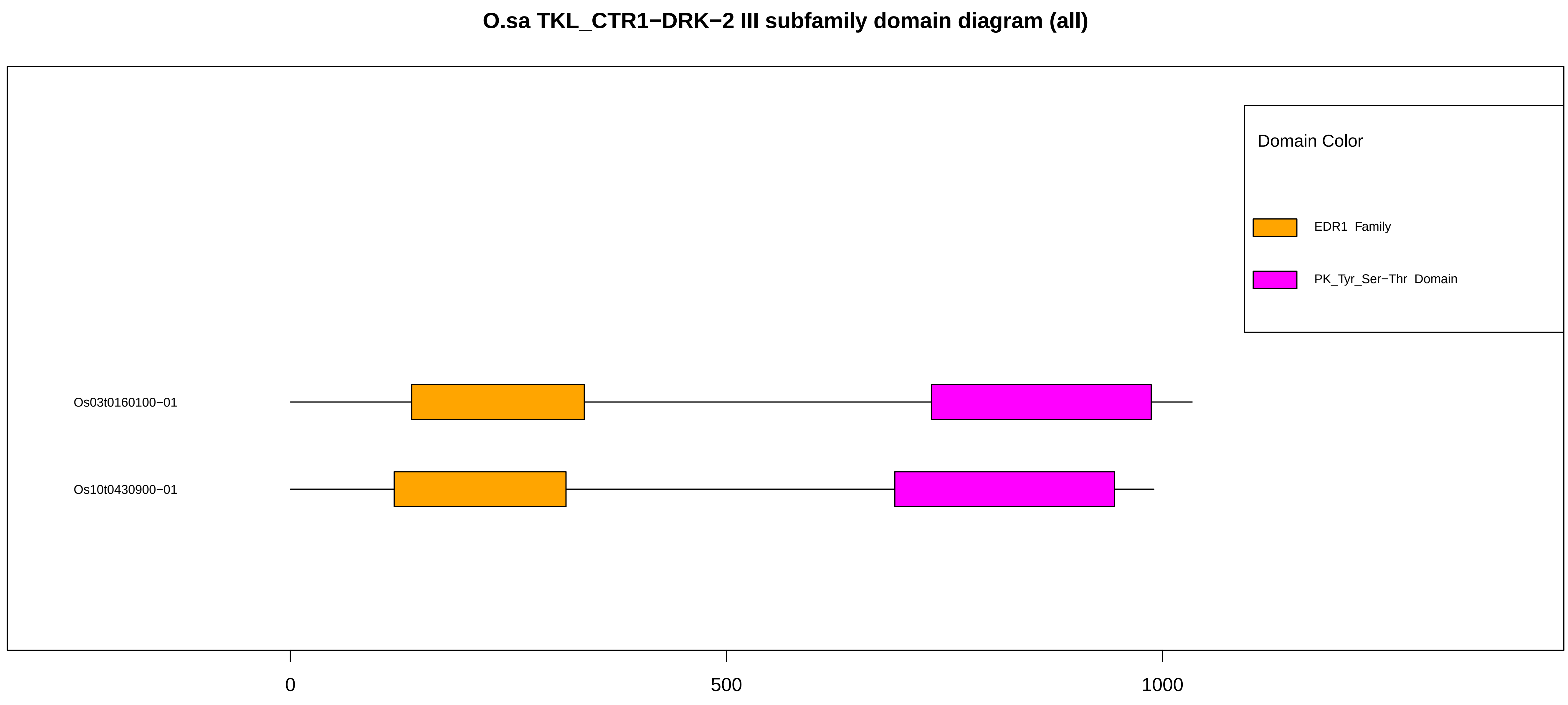
O.sa TKL_CTR1−DRK−2 III subfamily domain diagram (all)
Domain Color
EDR1 Family
PK_Tyr_Ser−Thr Domain
Os03t0160100−01
Os10t0430900−01
0
500
1000

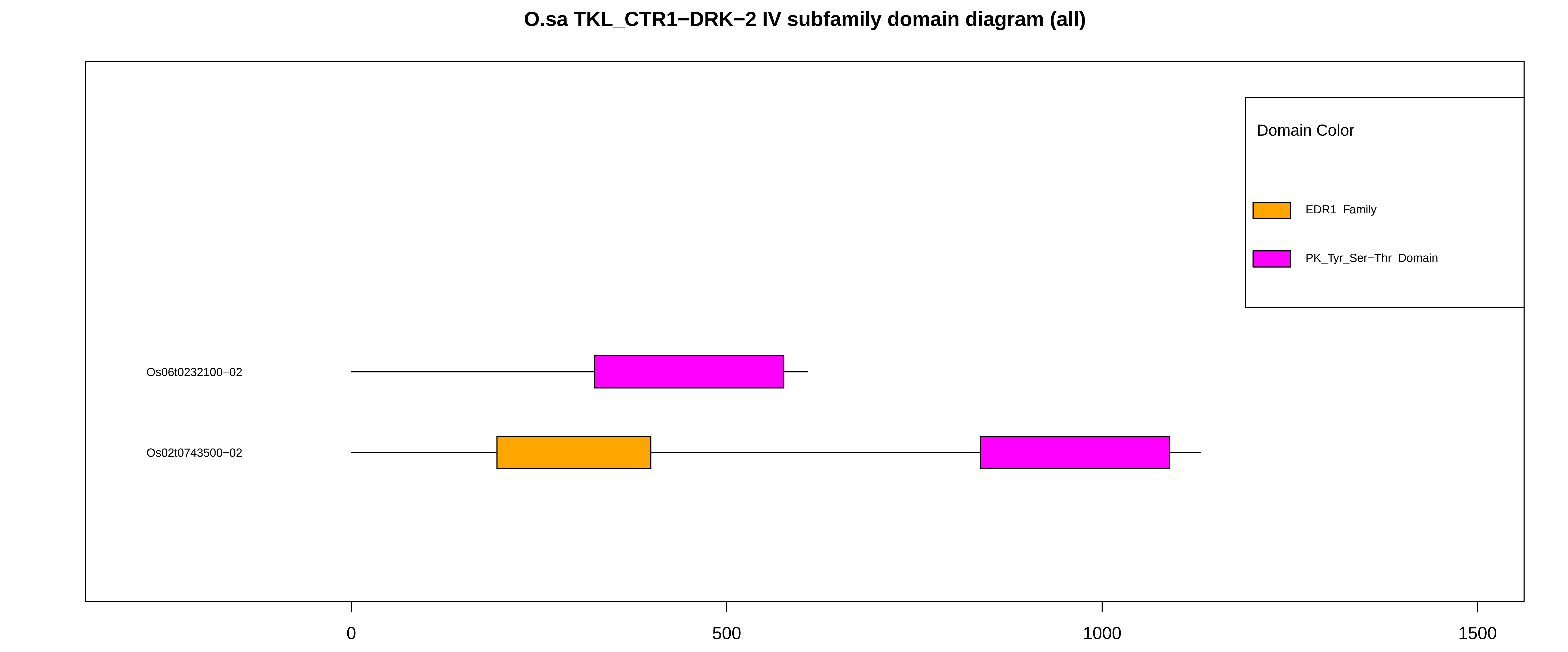

O.sa TKL_CTR1−DRK−2 IV subfamily domain diagram (all)
Domain Color
EDR1 Family
PK_Tyr_Ser−Thr Domain
Os06t0232100−02
Os02t0743500−02
0
500
1000
1500

O.sa TKL_CTR1−DRK−2 (excluding in phylogenetic analysis) domain diagram (all)

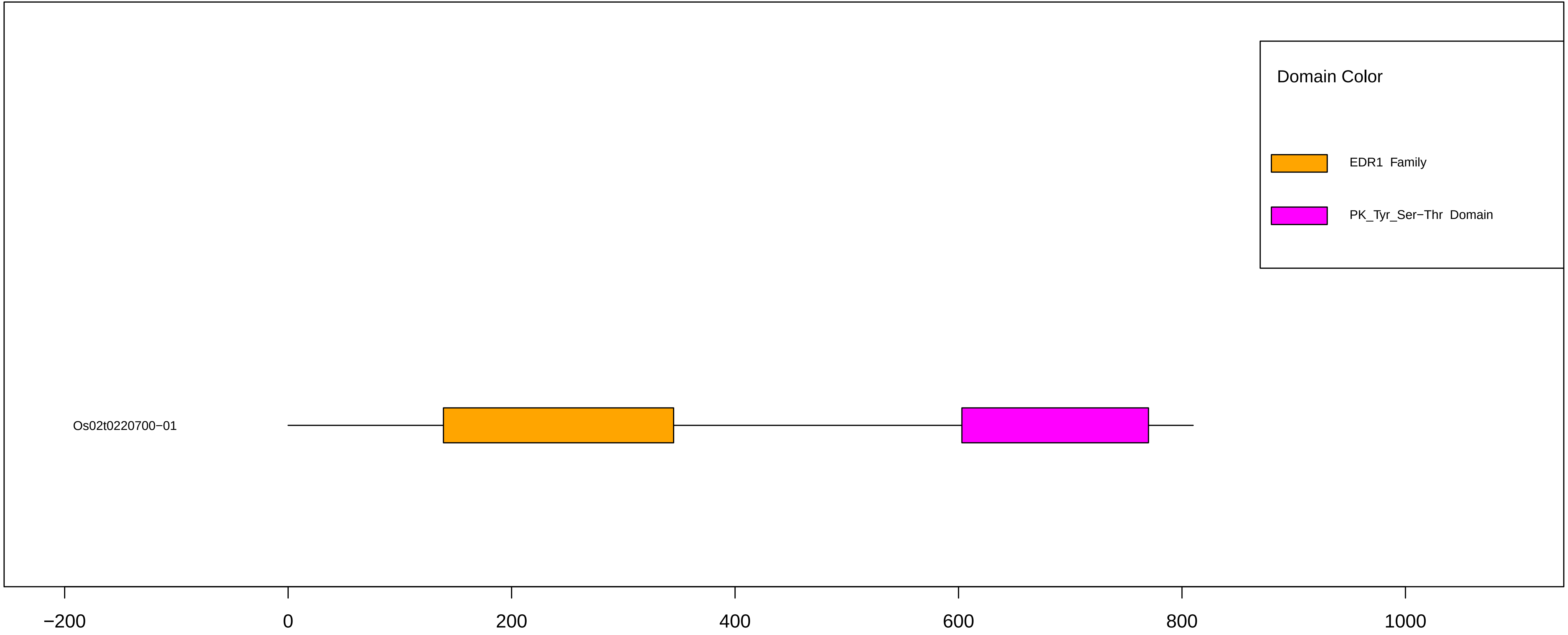

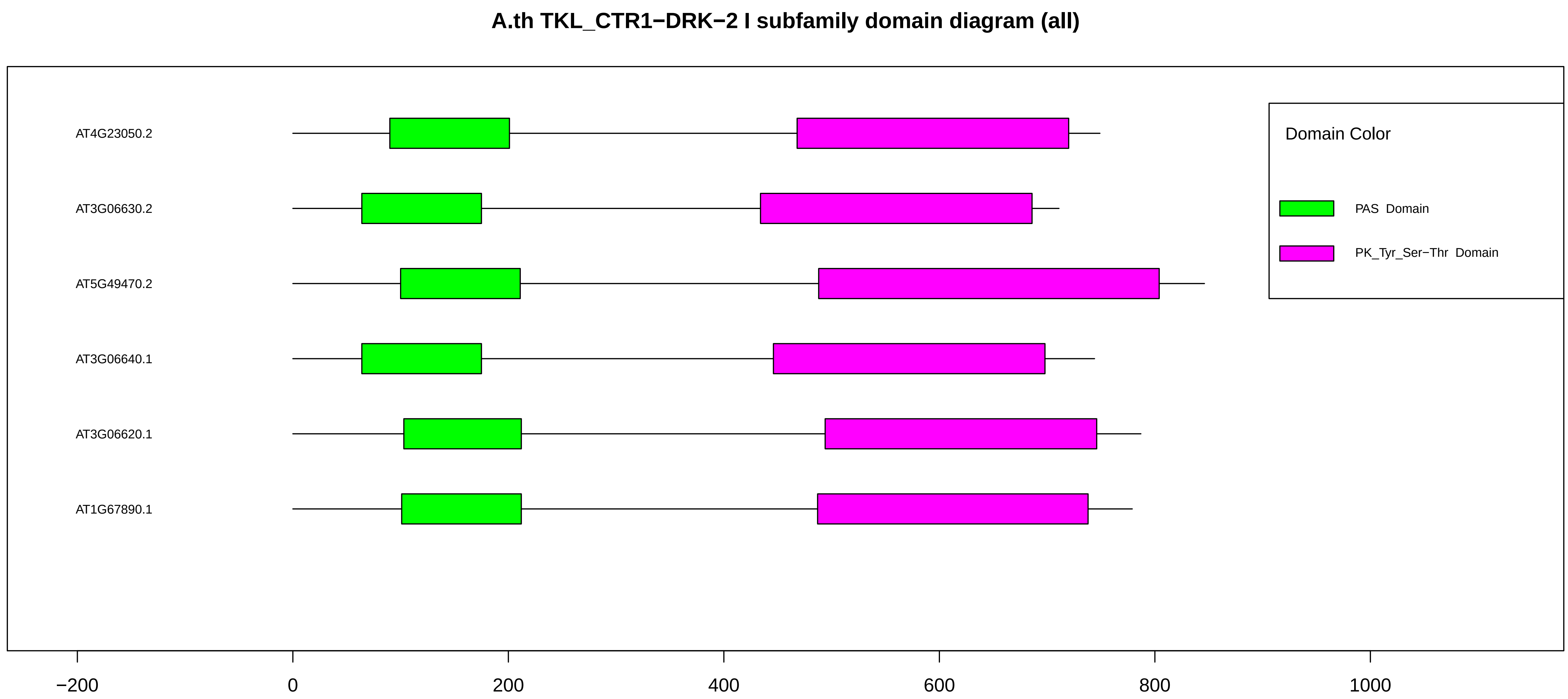
A.th TKL_CTR1−DRK−2 I subfamily domain diagram (all)
AT4G23050.2
AT3G06630.2
AT5G49470.2
AT3G06640.1
AT3G06620.1
AT1G67890.1
Domain Color
PAS Domain
PK_Tyr_Ser−Thr Domain
−200
0
200
400
600
800
1000

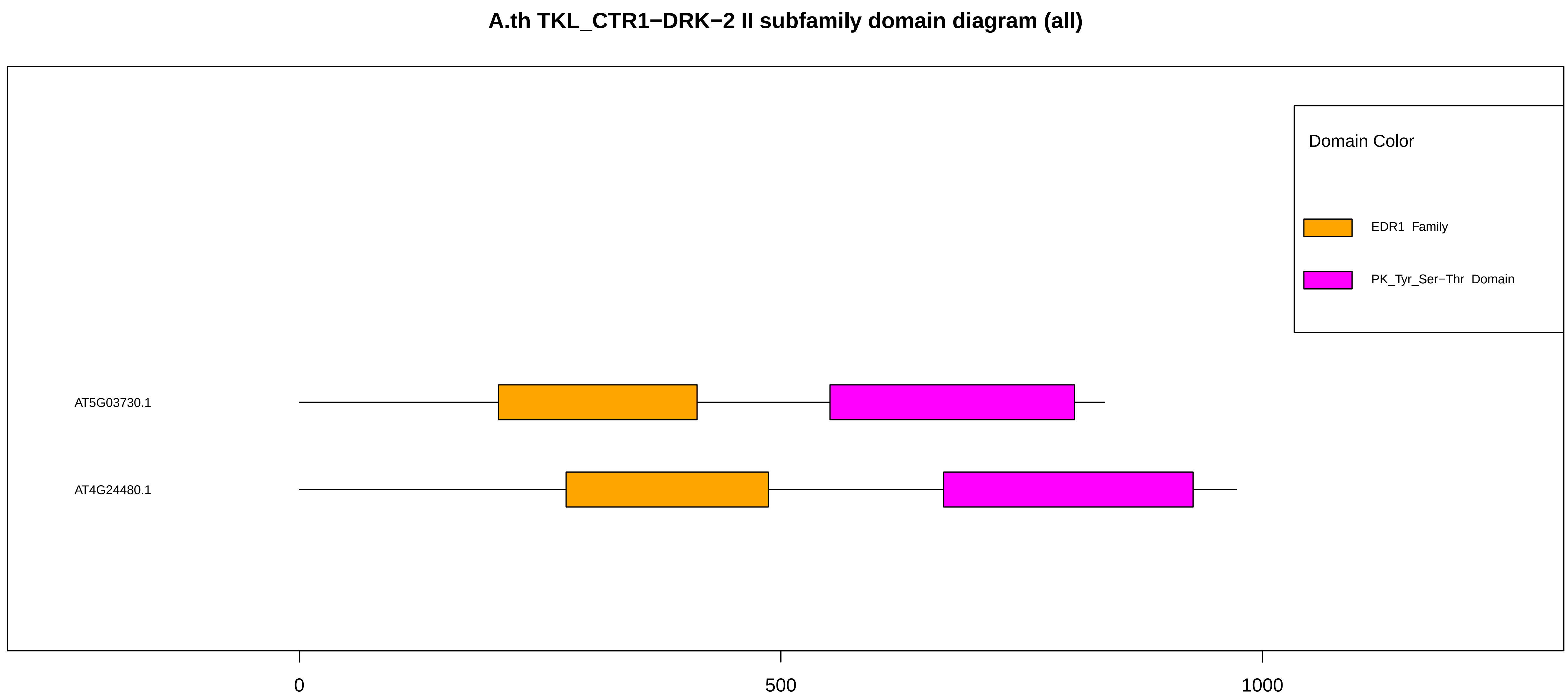
A.th TKL_CTR1−DRK−2 II subfamily domain diagram (all)
Domain Color
EDR1 Family
PK_Tyr_Ser−Thr Domain
AT5G03730.1
AT4G24480.1
0
500
1000

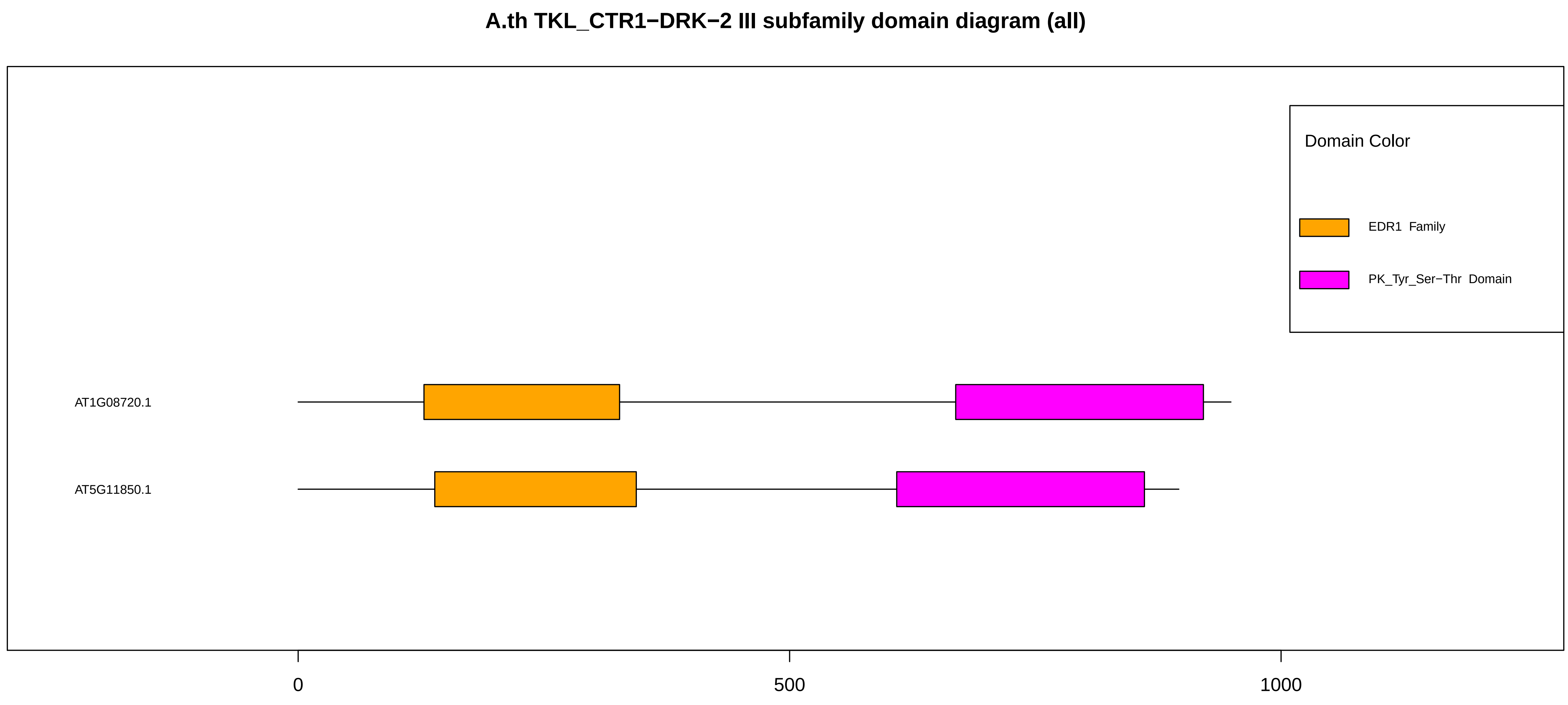
A.th TKL_CTR1−DRK−2 III subfamily domain diagram (all)
Domain Color
EDR1 Family
PK_Tyr_Ser−Thr Domain
AT1G08720.1
AT5G11850.1
0
500
1000

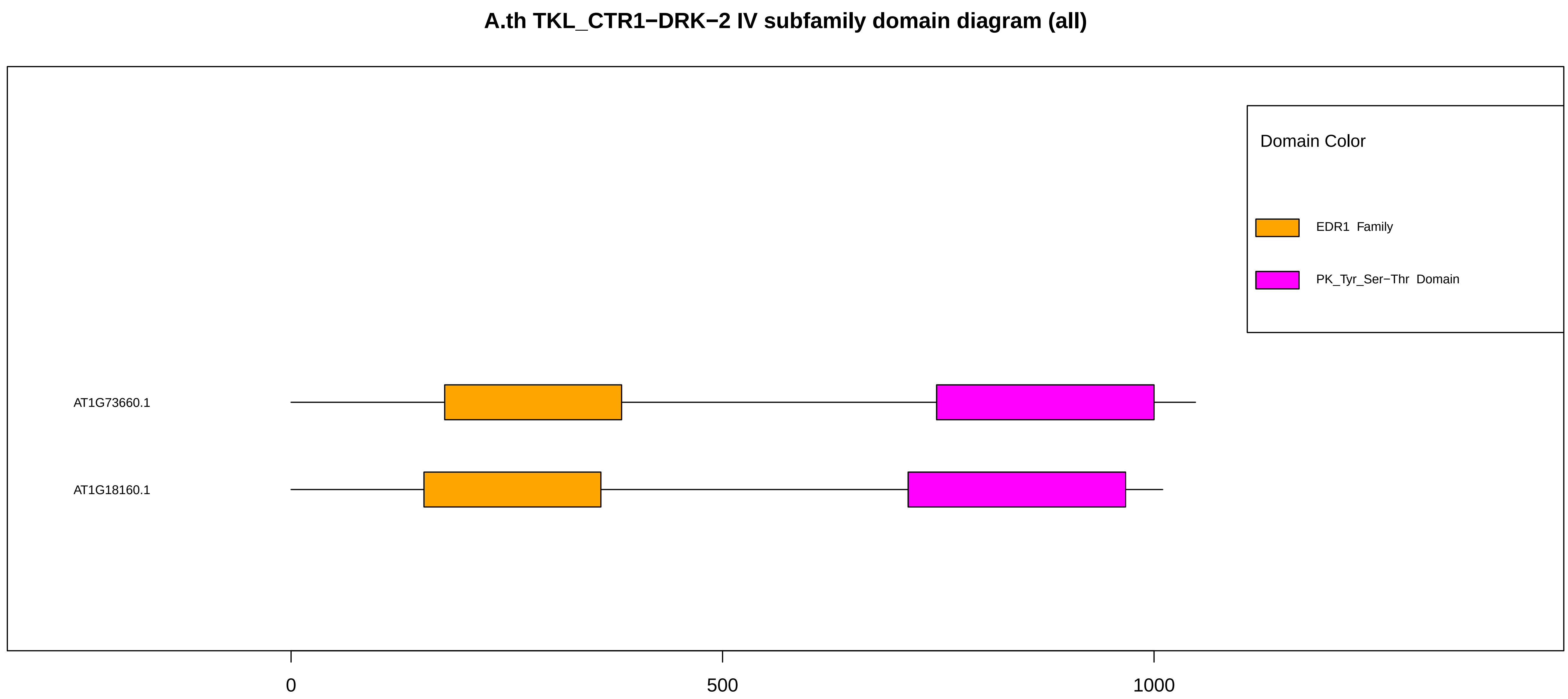

A.th TKL_CTR1−DRK−2 IV subfamily domain diagram (all)
Domain Color
EDR1 Family
PK_Tyr_Ser−Thr Domain
AT1G73660.1
AT1G18160.1
0
500
1000

V.vi TKL_CTR1−DRK−2 I subfamily domain diagram (all)

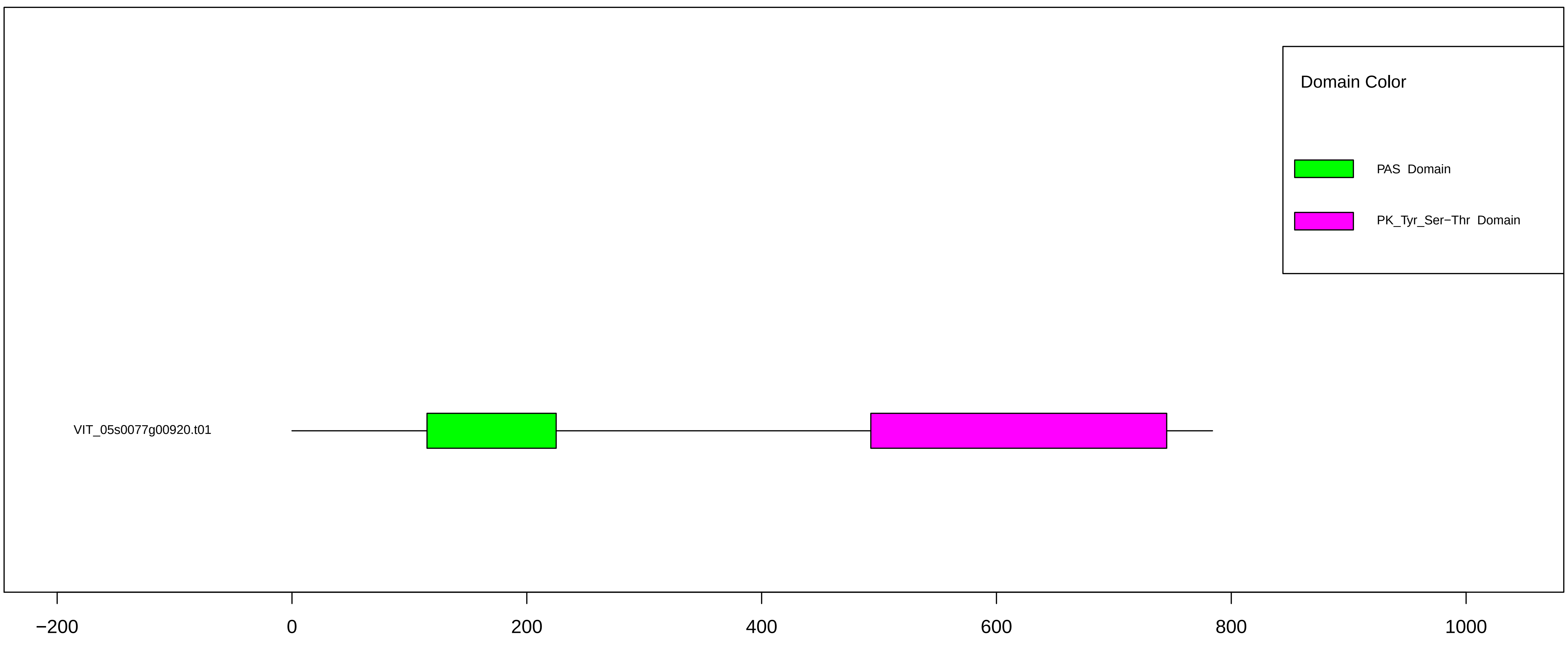

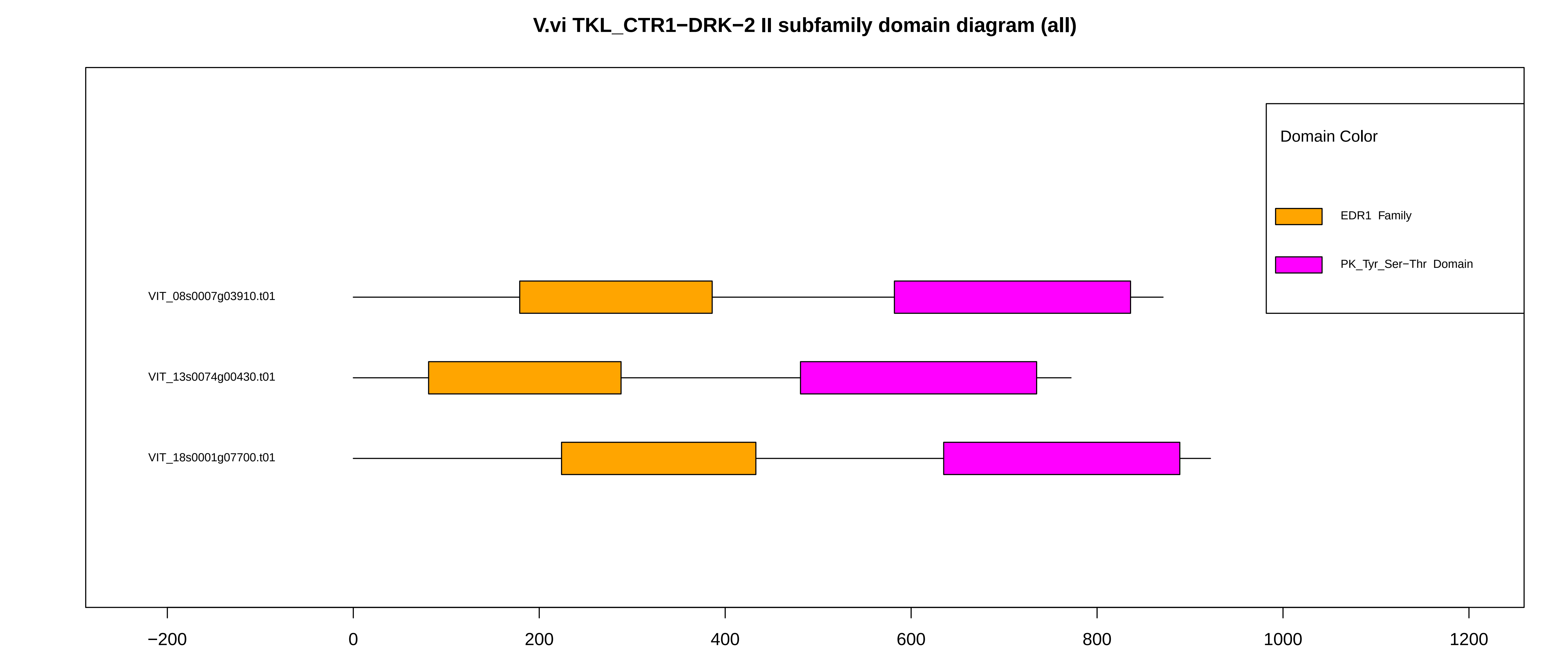

V.vi TKL_CTR1−DRK−2 II subfamily domain diagram (all)
Domain Color
EDR1 Family
PK_Tyr_Ser−Thr Domain
VIT_08s0007g03910.t01
VIT_13s0074g00430.t01
VIT_18s0001g07700.t01
−200
0
200
400
600
800
1000
1200

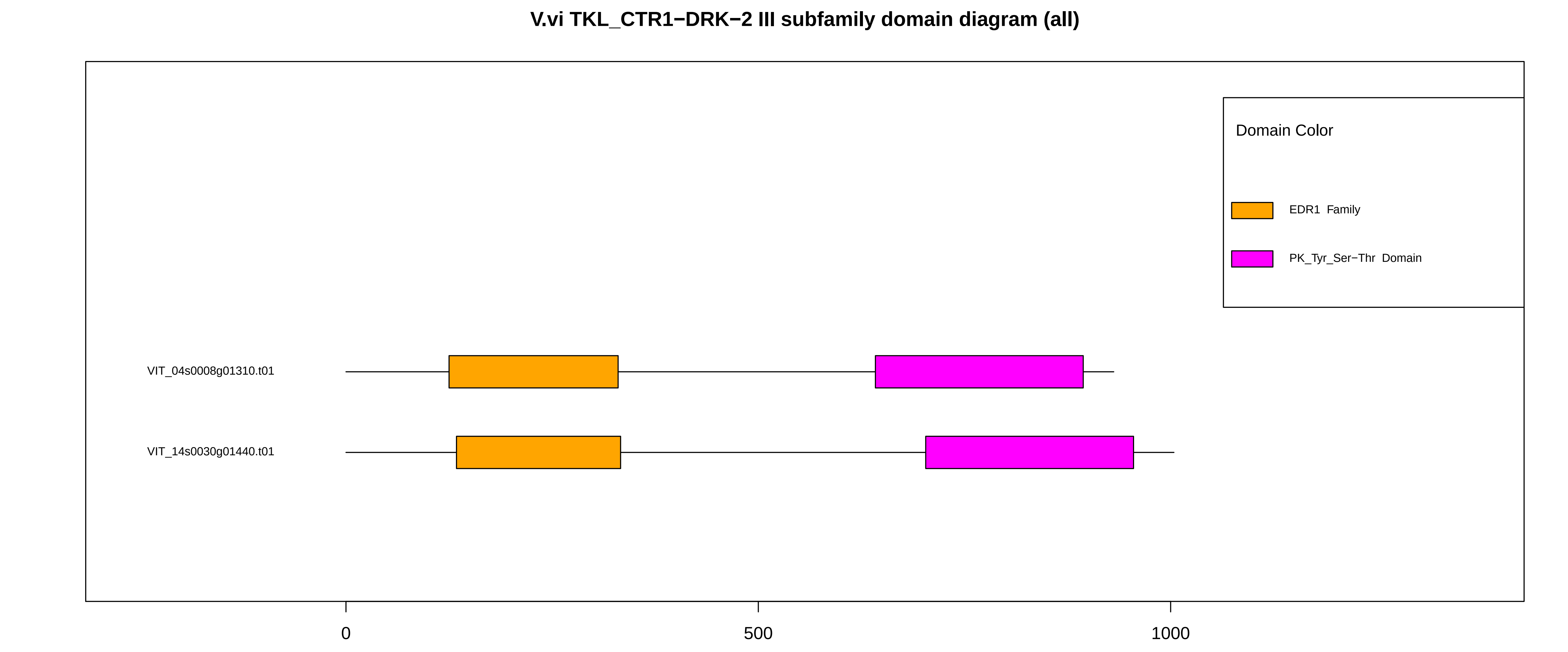
V.vi TKL_CTR1−DRK−2 III subfamily domain diagram (all)
Domain Color
EDR1 Family
PK_Tyr_Ser−Thr Domain
VIT_04s0008g01310.t01
VIT_14s0030g01440.t01
0
500
1000

V.vi TKL_CTR1−DRK−2 IV subfamily domain diagram (all)

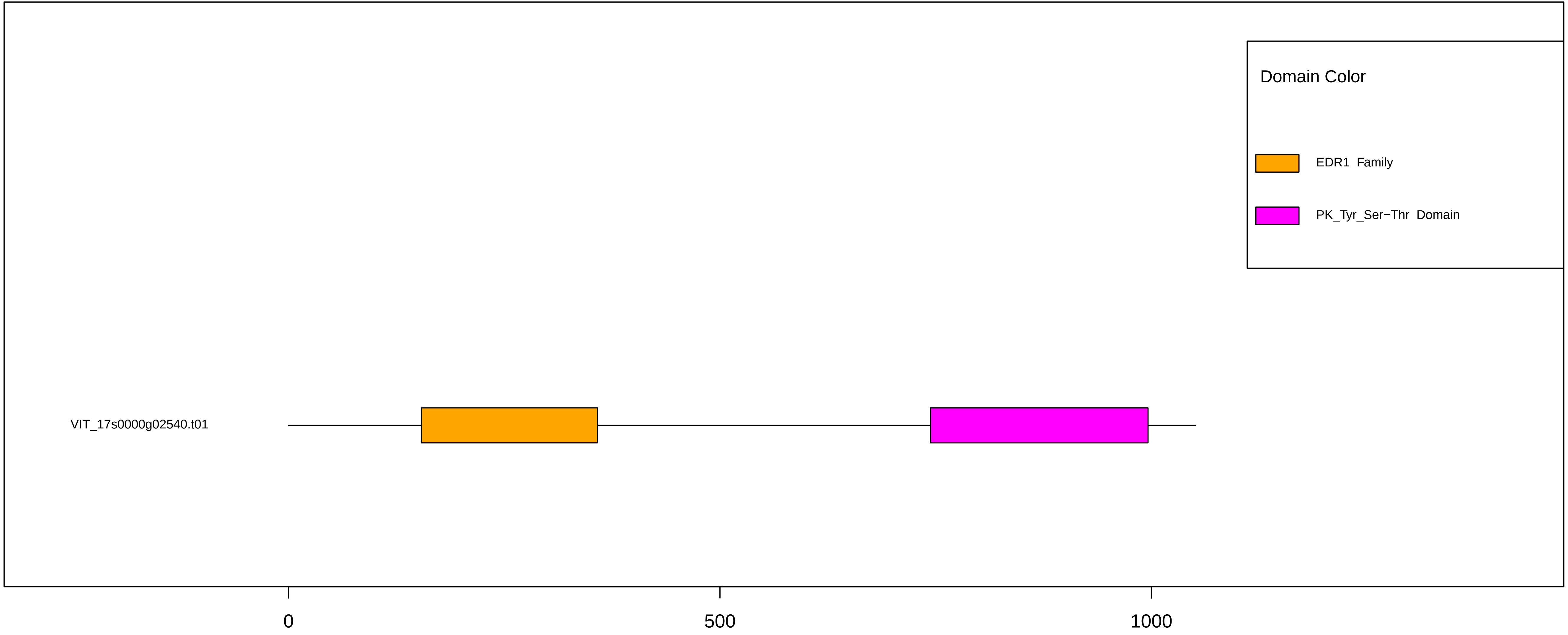

V.vi TKL_CTR1−DRK−2 (excluding in phylogenetic analysis) domain diagram (all)

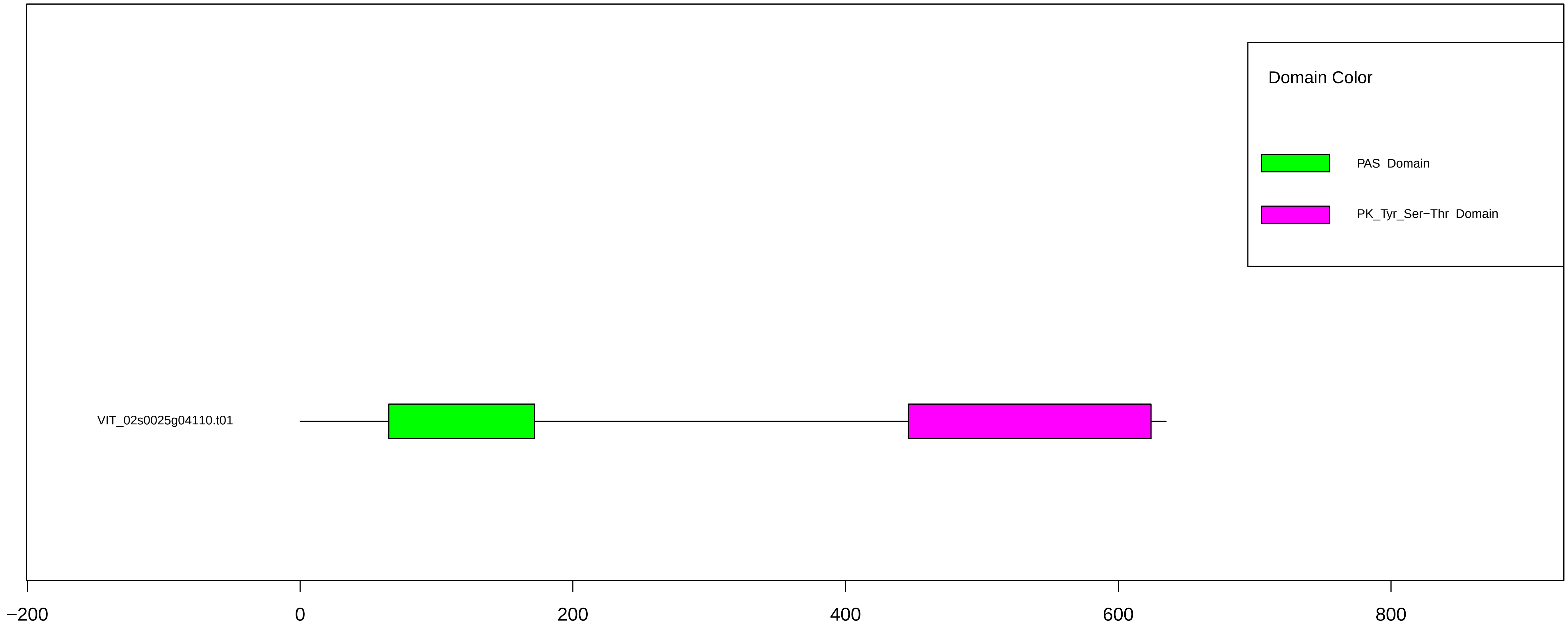

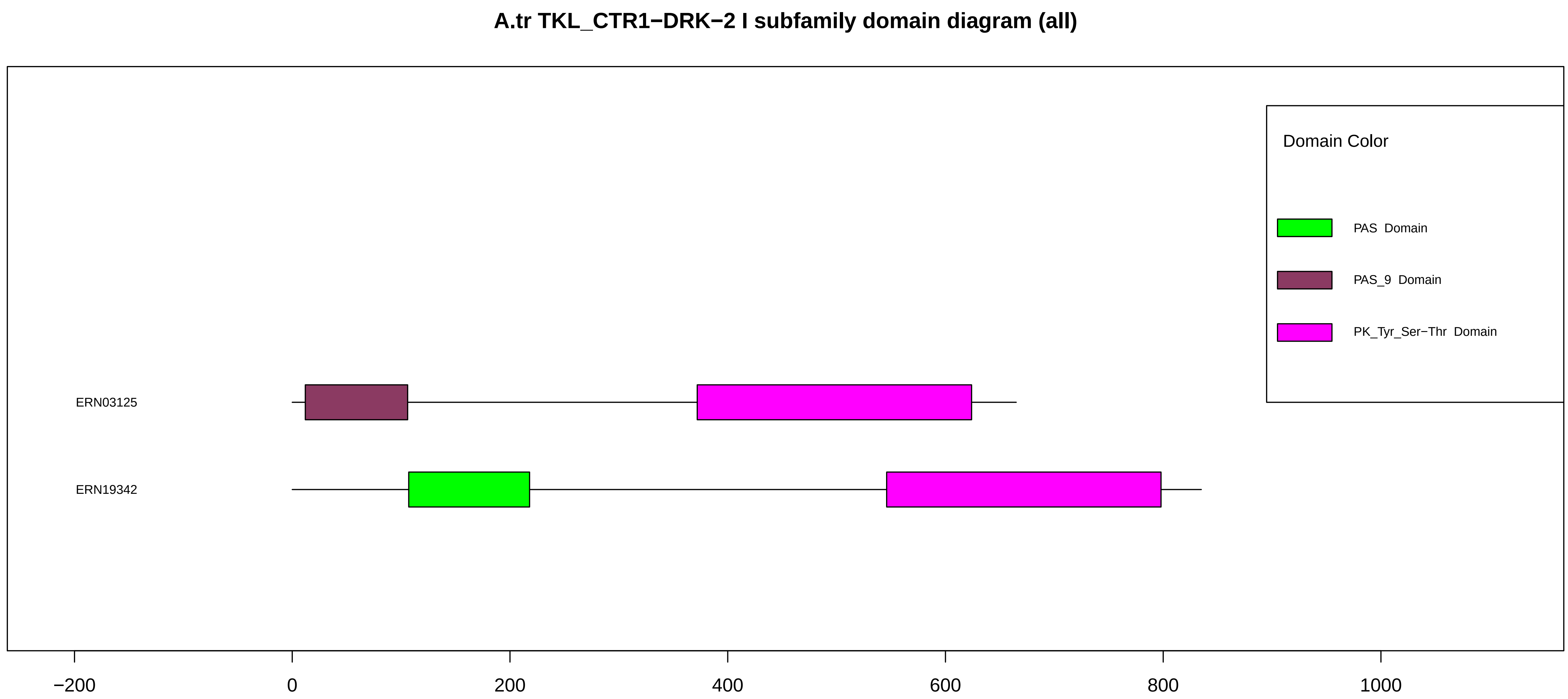
A.tr TKL_CTR1−DRK−2 I subfamily domain diagram (all)
Domain Color
PAS Domain
PAS_9 Domain
PK_Tyr_Ser−Thr Domain
ERN03125
ERN19342
−200
0
200
400
600
800
1000

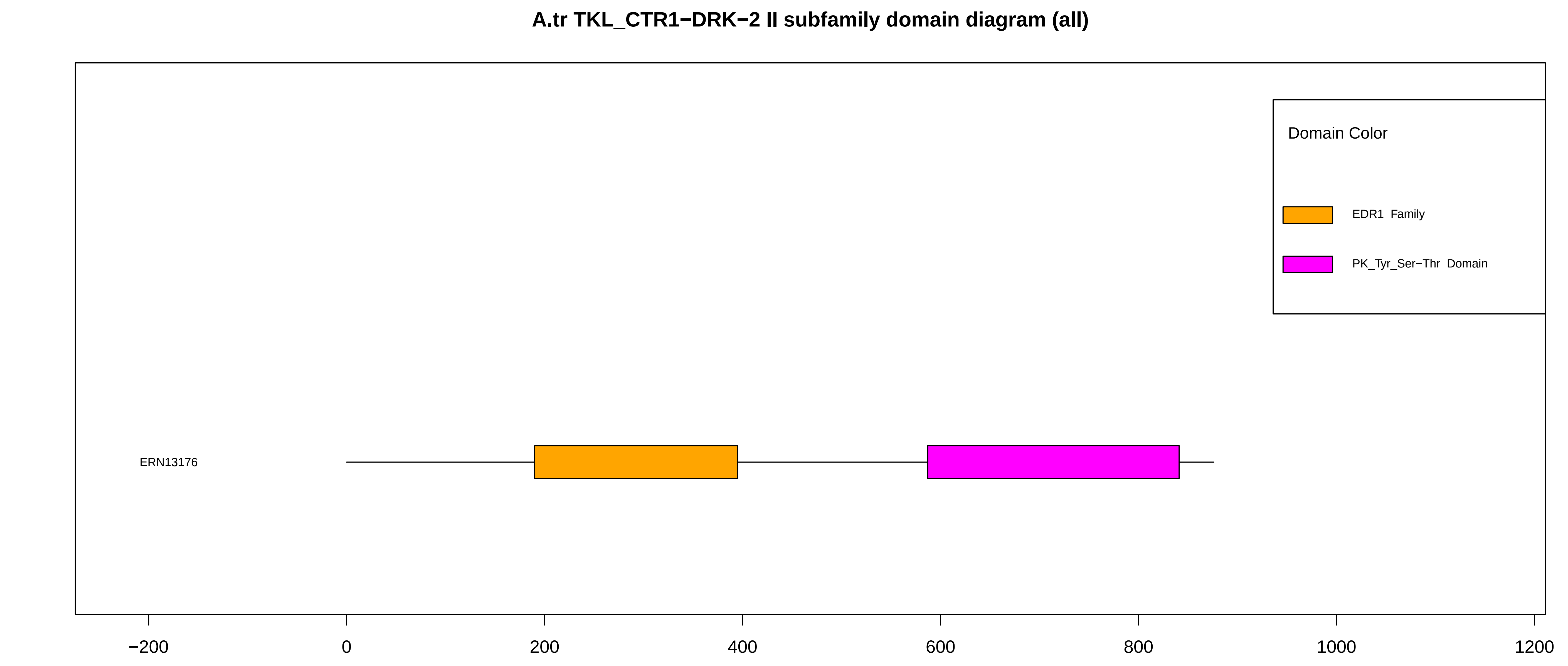
A.tr TKL_CTR1−DRK−2 II subfamily domain diagram (all)
Domain Color
EDR1 Family
PK_Tyr_Ser−Thr Domain
ERN13176
−200
0
200
400
600
800
1000
1200

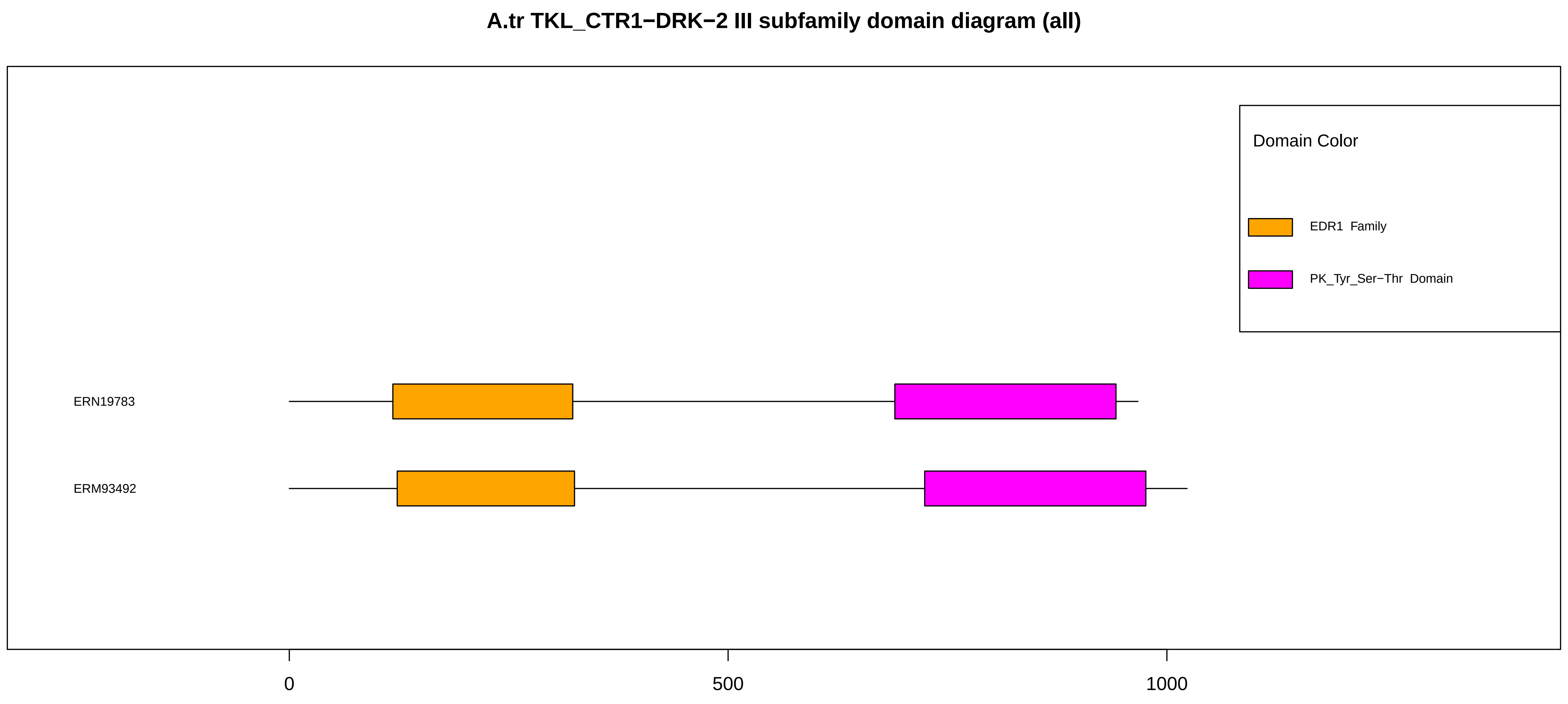
A.tr TKL_CTR1−DRK−2 III subfamily domain diagram (all)
Domain Color
EDR1 Family
PK_Tyr_Ser−Thr Domain
ERN19783
ERM93492
0
500
1000

A.tr TKL_CTR1−DRK−2 IV subfamily domain diagram (all)

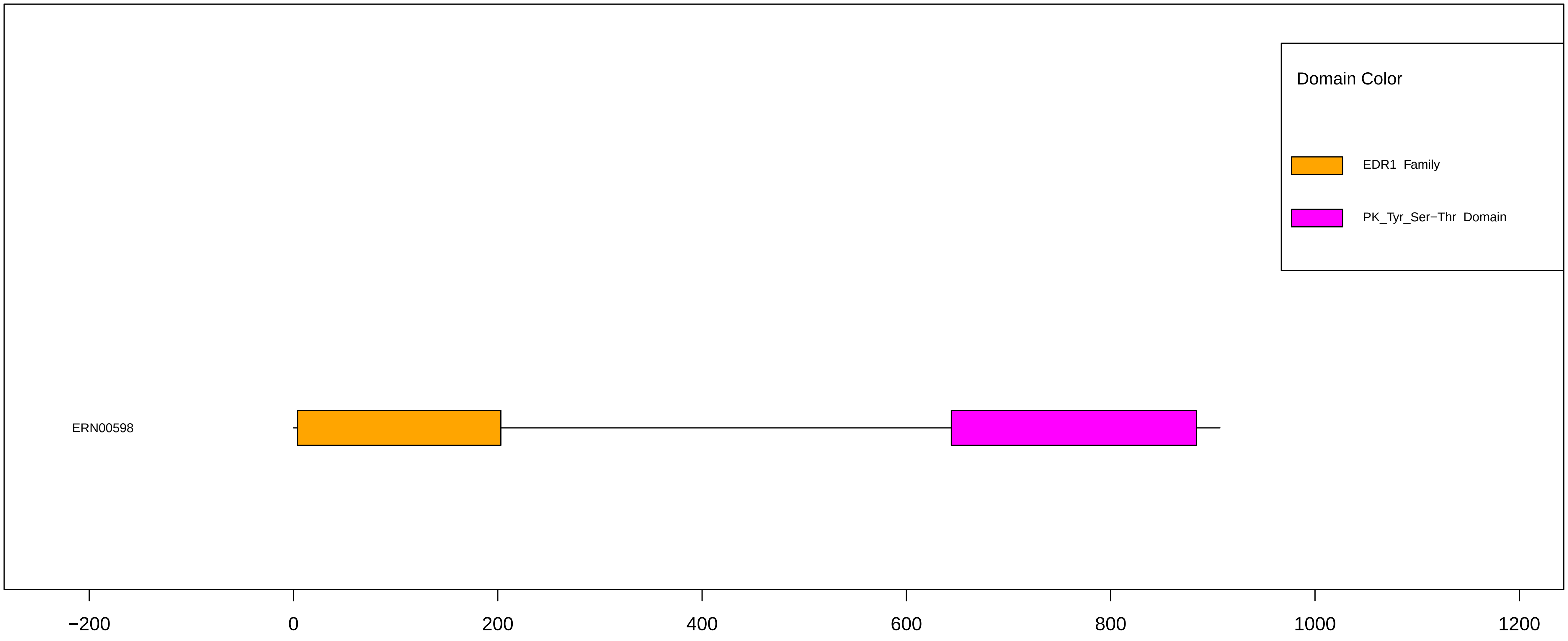

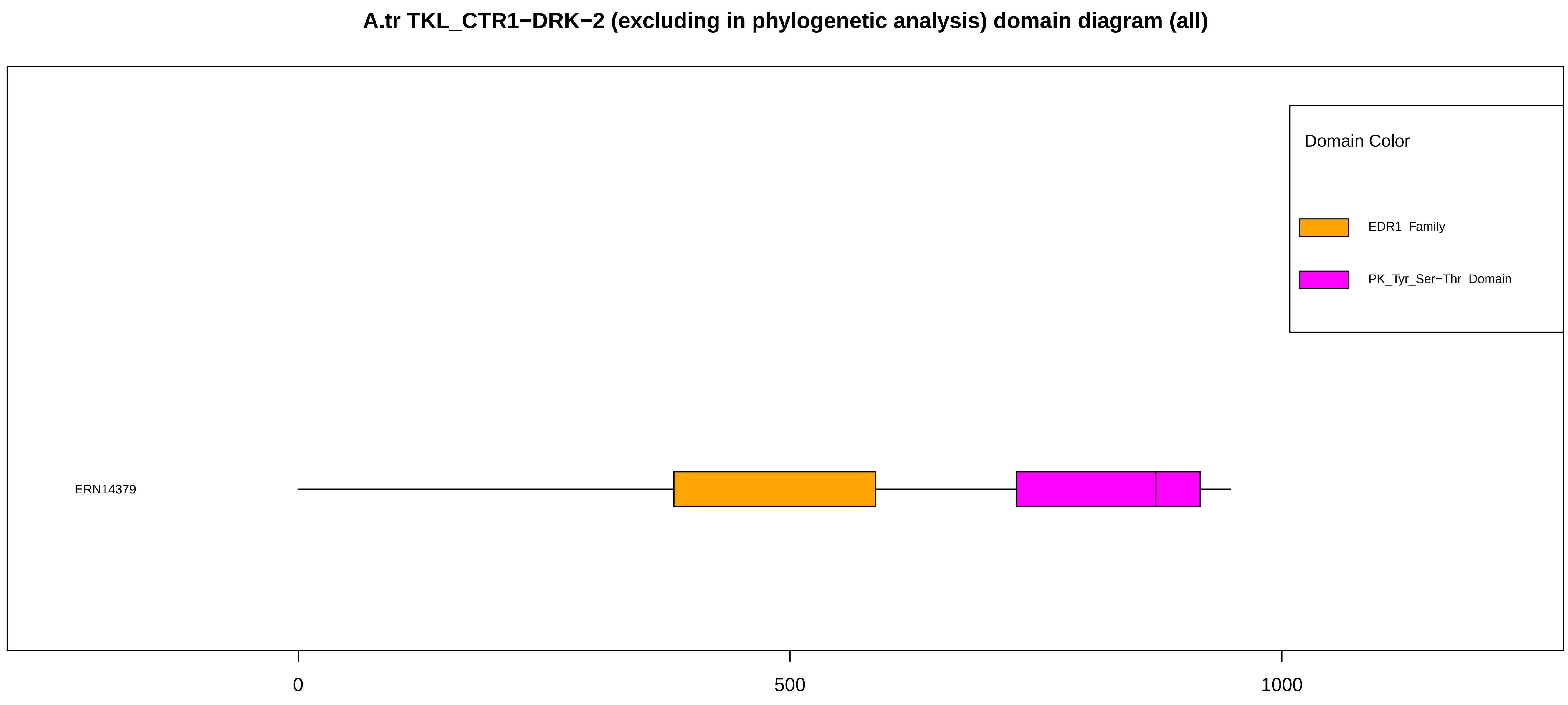
A.tr TKL_CTR1−DRK−2 (excluding in phylogenetic analysis) domain diagram (all)
Domain Color
EDR1 Family
PK_Tyr_Ser−Thr Domain
ERN14379
0
500
1000

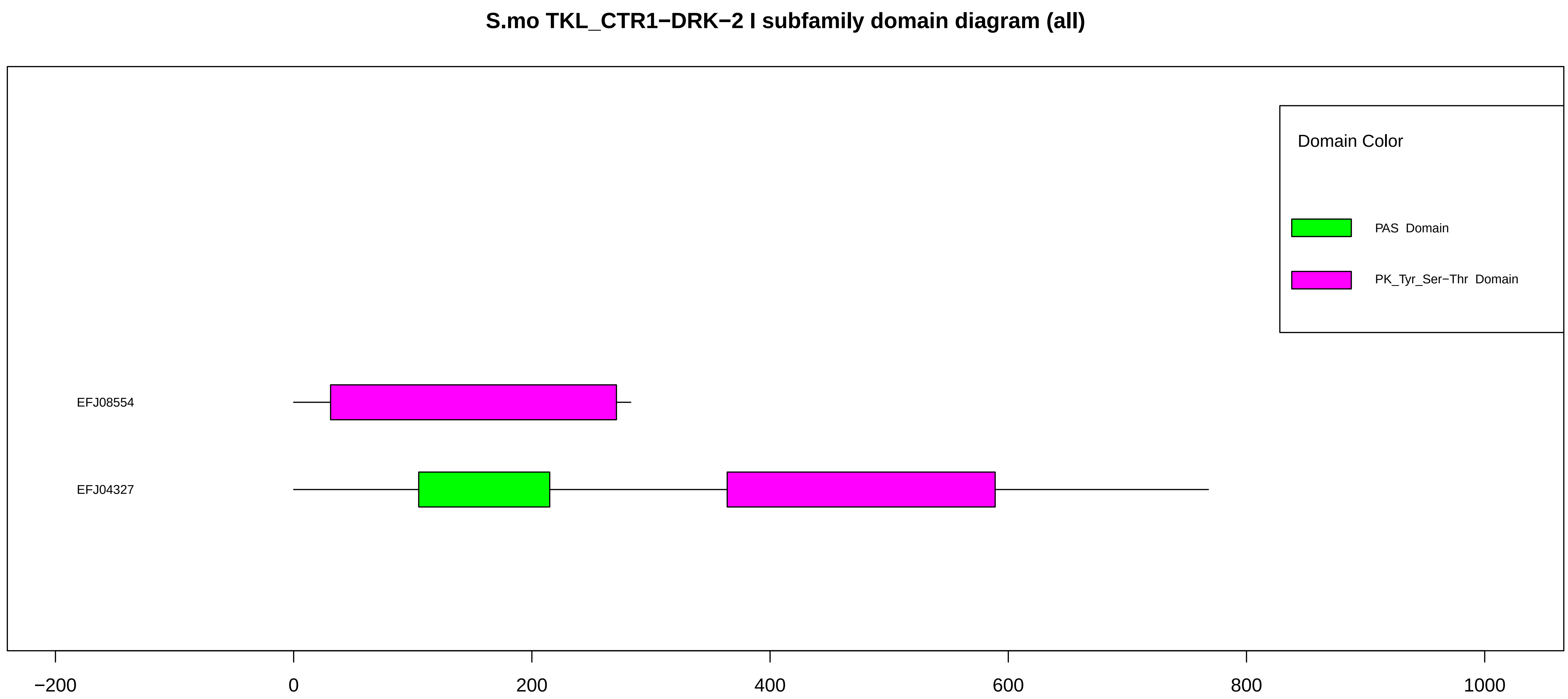

S.mo TKL_CTR1−DRK−2 I subfamily domain diagram (all)
Domain Color
PAS Domain
PK_Tyr_Ser−Thr Domain
EFJ08554
EFJ04327
−200
0
200
400
600
800
1000

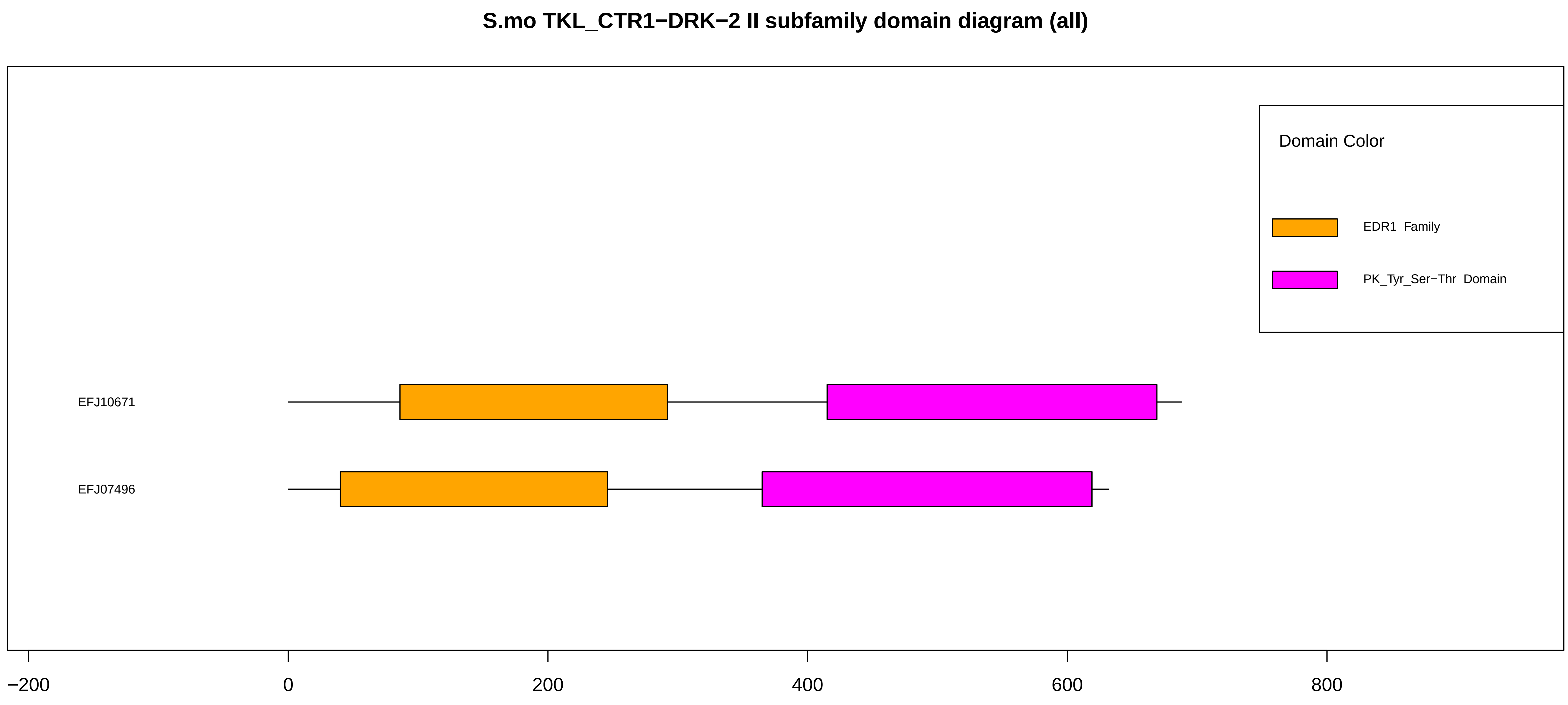
S.mo TKL_CTR1−DRK−2 II subfamily domain diagram (all)
Domain Color
EDR1 Family
PK_Tyr_Ser−Thr Domain
EFJ10671
EFJ07496
−200
0
200
400
600
800

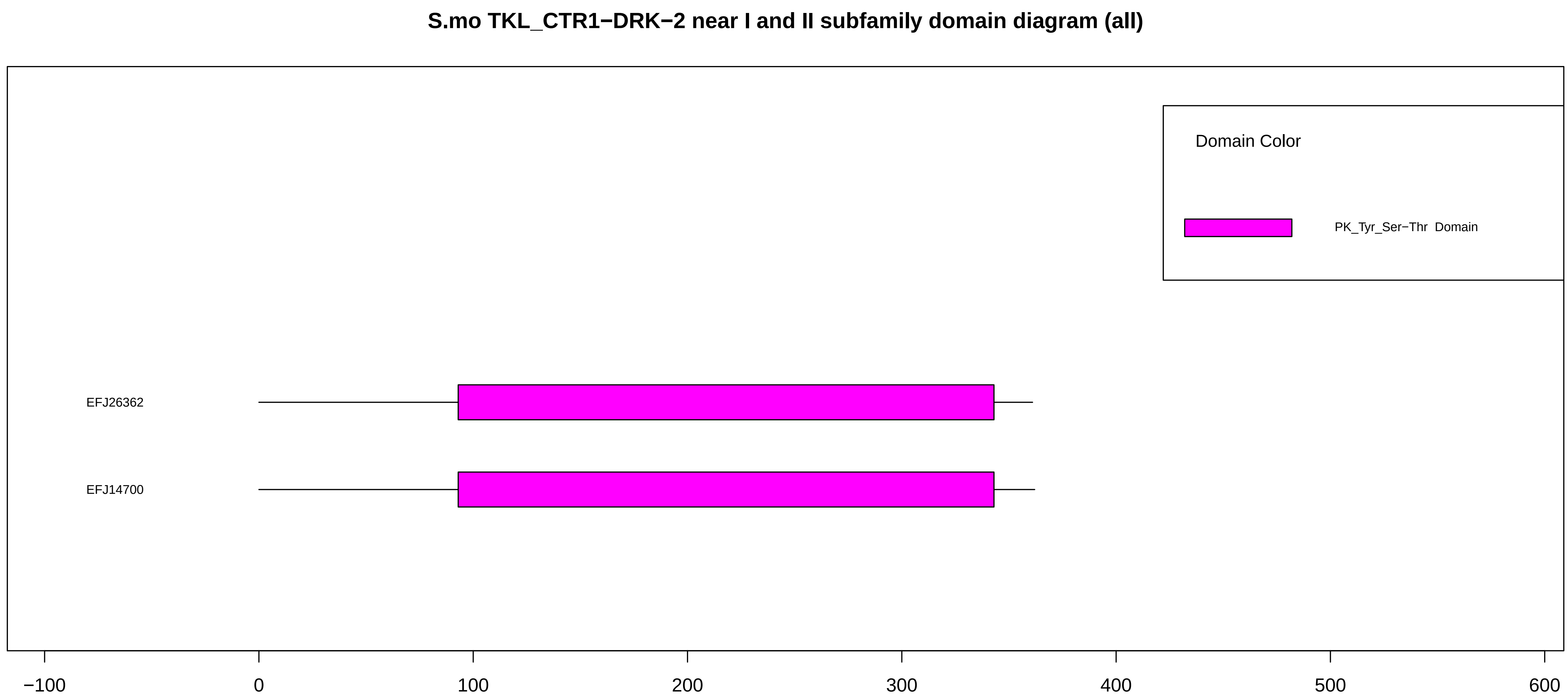
S.mo TKL_CTR1−DRK−2 near I and II subfamily domain diagram (all)
Domain Color
PK_Tyr_Ser−Thr Domain
EFJ26362
EFJ14700
−100
0
100
200
300
400
500
600

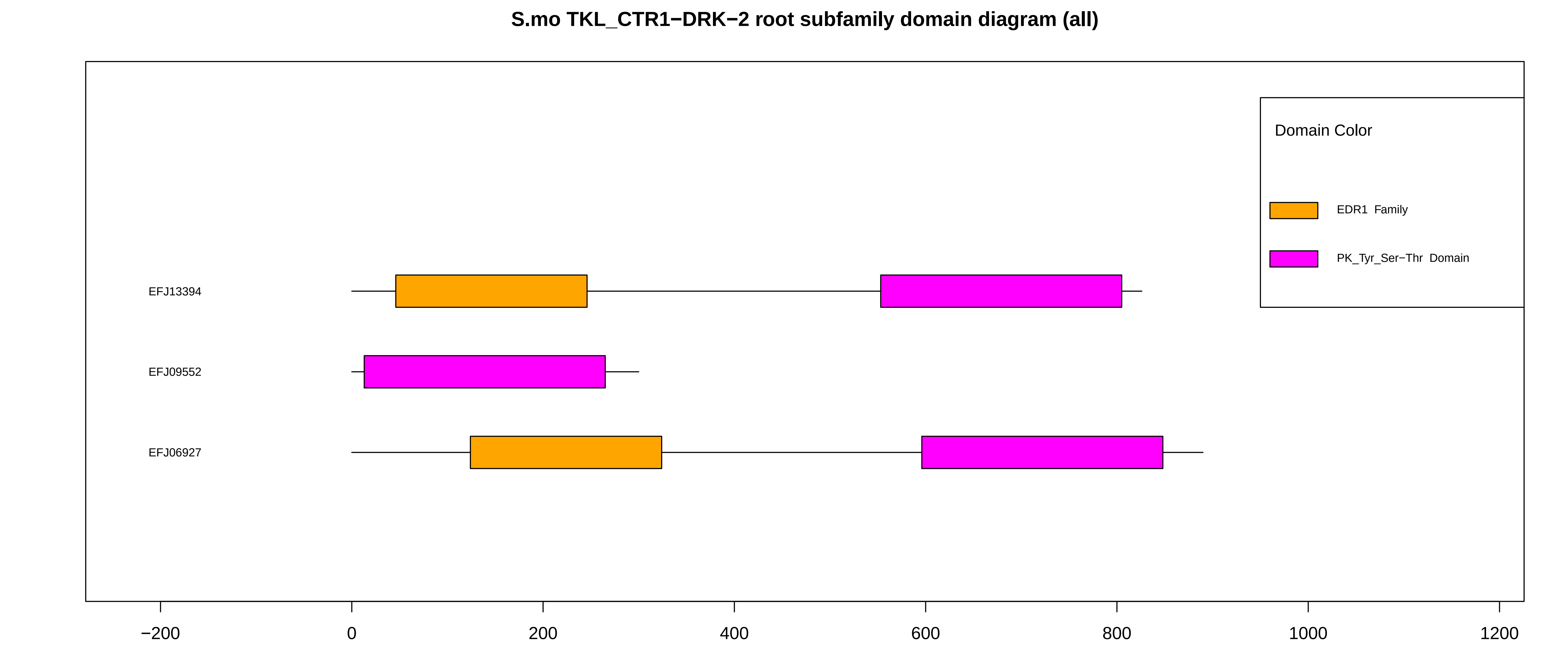
S.mo TKL_CTR1−DRK−2 root subfamily domain diagram (all)
Domain Color
EDR1 Family
PK_Tyr_Ser−Thr Domain
EFJ13394
EFJ09552
EFJ06927
−200
0
200
400
600
800
1000
1200

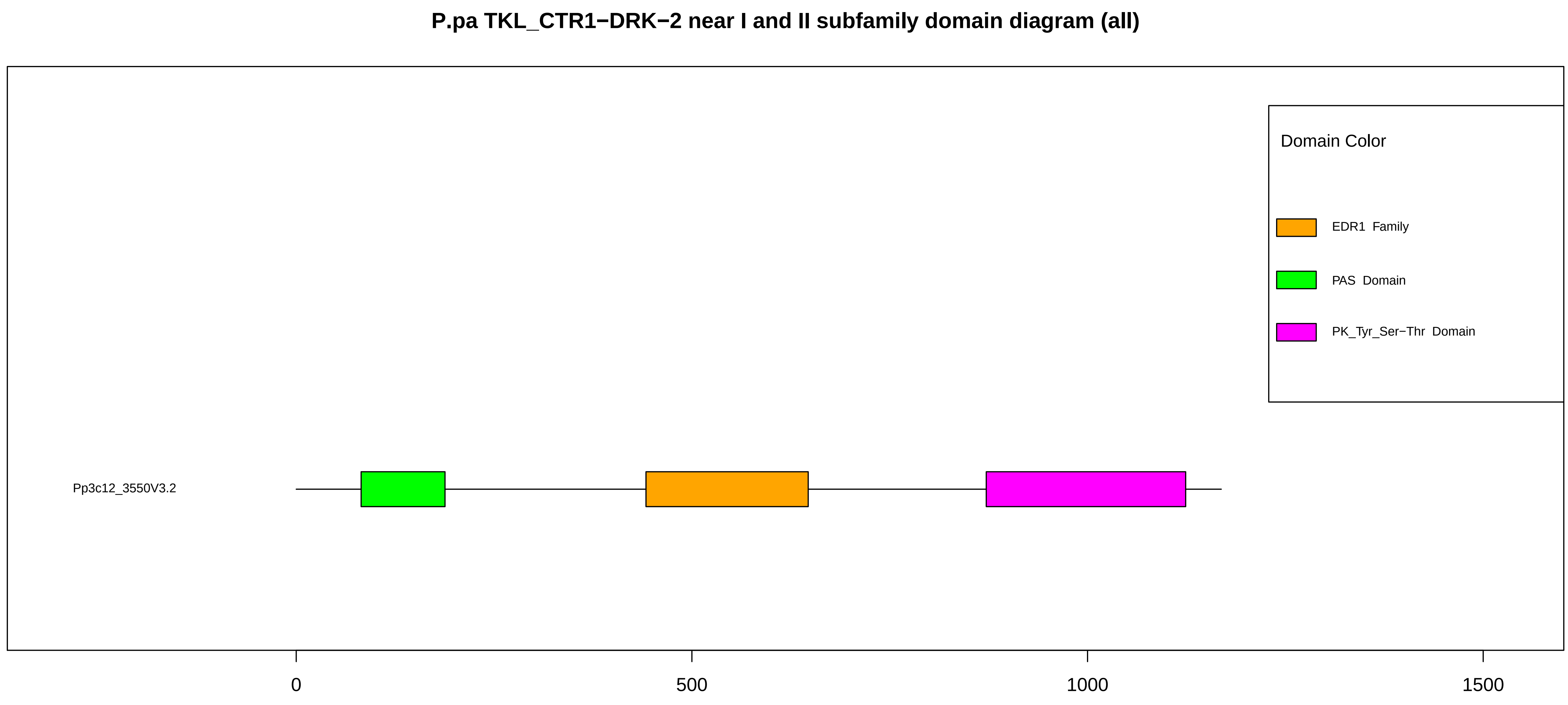
P.pa TKL_CTR1−DRK−2 near I and II subfamily domain diagram (all)
Domain Color
EDR1 Family
PAS Domain
PK_Tyr_Ser−Thr Domain
Pp3c12_3550V3.2
0
500
1000
1500

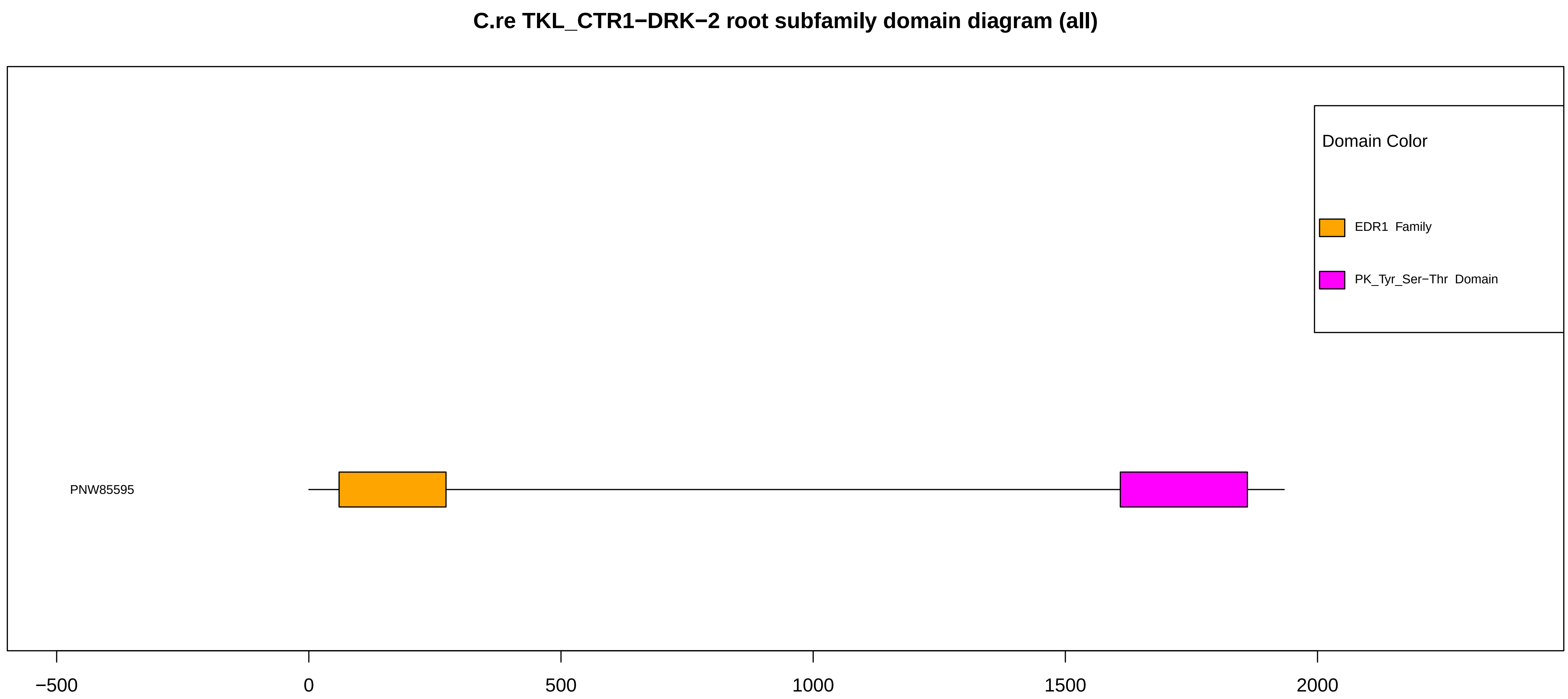
C.re TKL_CTR1−DRK−2 root subfamily domain diagram (all)
Domain Color
EDR1 Family
PK_Tyr_Ser−Thr Domain
PNW85595
−500
0
500
1000
1500
2000
